# The Mechanism of Ruthenium‐Catalyzed Directed C─H Arylation of Arenes: The Key Role of Bis‐Cyclometalated Intermediates

**DOI:** 10.1002/anie.202506707

**Published:** 2025-05-06

**Authors:** Pablo Domingo‐Legarda, Samuel E. Neale, Ambre Carpentier, Claire L. McMullin, Michael Findlay, Igor Larrosa, Stuart A. Macgregor

**Affiliations:** ^1^ Department of Chemistry University of Manchester Oxford Road Manchester M13 9PL UK; ^2^ Institute of Chemical Sciences Heriot‐Watt University Edinburgh EH14 4AS UK; ^3^ EaStCHEM School of Chemistry North Haugh University of St. Andrews St. Andrews KY16 9ST UK

**Keywords:** C‒X activation, C‒H functionalization, DFT calculations, Kinetic studies, Mechanistic elucidation, Ruthenium catalysis

## Abstract

The mechanism of Ru‐catalyzed *N*‐directed C‐H *ortho*‐arylation with haloarenes has been under intense scrutiny over the last decade, with conflicting proposals concerning the relevance of various catalytic intermediates and the nature of the key steps. This work presents experimental and computational studies that address these long‐standing questions. Stoichiometric, catalytic, and mechanistic kinetic studies, supported by DFT calculations, reveal that bis‐cyclometallated ruthenium species are key intermediates in these reactions. These studies also show that oxidative addition with bromoarenes proceeds via a concerted oxidative addition pathway, as demonstrated by DFT and experimental kinetic orders. Bromoarene activation does not proceed at mono‐cyclometalated species. In the catalytic process, zero order kinetics are observed on both reaction substrates, an observation that is rationalized by DFT calculations which predict a rate‐limiting step within the product‐release stage. These results showcase how detailed experimental and DFT studies can combine to probe mechanistic questions, as well as resolving opposing views around the mechanism of these Ru‐catalyzed arylations that form the basis of promising mild C─H functionalizations.

## Graphical abstract

The mechanism of Ru‐catalyzed N‐directed C─H *ortho*‐arylation with haloarenes is addressed through experimental stoichiometric, catalytic kinetic, and DFT studies. These reveal a key role for bis‐cyclometallated ruthenium species and show bromoarene activation proceeds via a concerted oxidative addition. Bromoarene activation at mono‐cyclometalated species is ruled out.

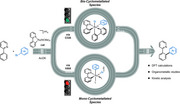

## Introduction

**Figure 1:**
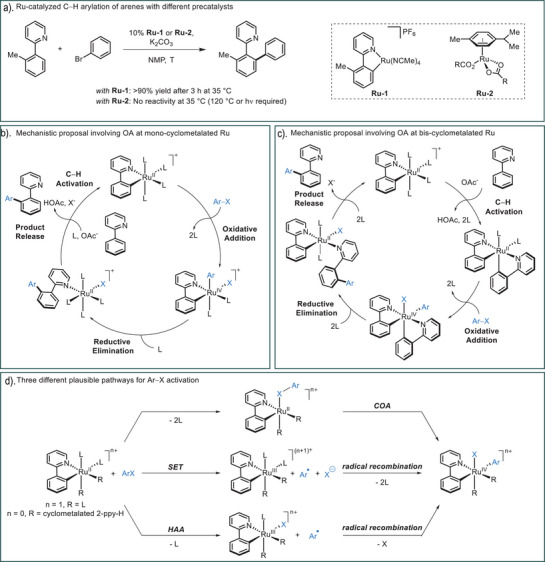
Ruthenium(II)‐catalyzed C─H arylations. a) Catalytic activity comparison between mono‐cyclometalated ruthenium complex **Ru‐1** and *p*‐cymene‐ligated complex **Ru‐2**. b) A mechanistic proposal involving oxidative addition of Ar─X at a mono‐cyclometalated Ru(II) intermediate. c) A mechanistic proposal involving oxidative addition of Ar─X at a bis‐cyclometalated‐Ru(II). d) Potential pathways for oxidative addition of Ar─X at a Ru(II) intermediate: concerted oxidative addition (COA), single‐electron transfer (SET), or halogen atom abstraction (HAA).

Biaryl motifs represent one of the most common scaffolds in industrially important fine chemicals and are found in a wide variety of pharmaceuticals,^[^ ^1^, ^2^ ^]^ agrochemicals,^[^ ^3^ ^]^ and functional materials.^[^ ^4^ ^]^ Although the synthesis of these molecules has traditionally relied on the use of transition‐metal‐catalyzed cross‐coupling chemistry, in the last two decades the direct C─H arylation of arenes has emerged as a more sustainable and streamlined approach.^[^ ^5^, ^6^, ^7^, ^8^, ^9^, ^10^, ^11^, ^12^, ^13^, ^14^, ^15^, ^16^, ^17^, ^18^, ^19^, ^20^, ^21^, ^22^, ^23^, ^24^, ^25^ ^]^ Since Oi and Inoue's seminal report on the use of air‐ and moisture‐stable Ru(II) complexes for direct C─H arylation procedures,^[^ ^26^ ^]^ significant progress has been made, greatly improving the scope and efficiency of Ru‐catalyzed C─H arylation reactions.^[^ ^27^, ^28^, ^29^, ^30^, ^31^ ^]^ The mechanisms through which these catalytic reactions proceed have been under intense scrutiny, with various proposals put forward.^[^ ^32^, ^33^, ^34^ ^]^ Although for many years a matter of debate, it is now currently accepted that the *p*‐cymene ligand in typical Ru precatalysts of general structure [Ru(*p*‐cymene)X_2_]_n_ must be removed as a preactivation step and that a mono‐cyclometalated species of the type [Ru(C∩N)L_4_]X must initially be formed. Indeed, we have shown that **Ru‐1** can catalyze the arylation reaction of 2‐*ortho*‐tolylpyridine with bromobenzene at 35 °C (Figure 1a).^[^ ^35^ ^]^ In contrast, when using Ru(*p*‐cymene)‐based precatalysts such as **Ru‐2** either temperatures of 120 °C or light‐mediated *p*‐cymene dissociation is typically required.^[^ ^36^, ^37^ ^]^ On the other hand, the nature of the *p*‐cymene‐free Ru species that undergo oxidative addition with Ar–X electrophiles, as well as the mechanism of oxidative addition itself, remain a matter of debate, with several conflicting proposals and studies. Historically, the most commonly proposed Ru‐*ortho*‐arylation mechanisms involve the oxidative addition of Ar–X at a mono‐cyclometalated Ru species, followed by reductive elimination (Figure 1b).^[^ ^32^, ^33^, ^34^, ^36^, ^37^ ^]^ However, in 2018, the Larrosa group showed that mono‐cyclometalated species such as **Ru‐1** react sluggishly and only at high temperatures with Ar–I, which is inconsistent with the proposed oxidative addition. Instead, it was proposed that a second molecule of the 2‐phenylpyridine substrate (**2‐ppy‐H**) must undergo cyclometalation to form a bis‐cyclometalated Ru(2‐ppy)_2_L_2_ species prior to reaction with Ar–I (Figure 1c).^[^ ^35^ ^]^ We have also shown that bis‐aryl intermediates are key to Ar–Br activation in the catalytic arylation of pentafluorobenzene.^[^ ^38^ ^]^ In contrast, more recent calculations by Ackermann on the arylation of **2‐ppy‐H** with Ph–Br suggested that bis‐cyclometalated Ru‐species are less reactive and that Ar–X activation proceeds at a mono‐cyclometalated species (Figure 1b).^[^ ^39^ ^]^

Adding to the complexity of this picture is the ability of Ru to undergo both one‐electron and two‐electron processes. A number of pathways for the formal oxidative addition process have therefore been proposed, including: i) a concerted oxidative addition (COA); ii) single‐electron transfer (SET) followed by radical recombination; or iii) halogen atom abstraction (HAA) followed by radical recombination (Figure 1d). In their more recent study, Ackermann and coworkers proposed a HAA pathway and found this was clearly favored over a COA transition state that was 23.8 kcal mol^−1^ higher in energy.^[^ ^39^ ^]^ However, the overall barrier reported for the arylation reaction in this study was 29.2 kcal mol^−1^, which is much larger than one would expect for reactions that run smoothly at 35 °C.^[^ ^40^ ^]^ In addition, the potential role of bis‐cyclometalated Ru species was not fully explored computationally, with only HAA being considered for arylation, and no experimental studies were carried out to validate the proposed mono‐cyclometalated pathway. Taken together, these results prompted us to re‐examine the mechanism of Ar−X oxidative addition at both mono‐ and bis‐cyclometalated‐Ru species.

Herein we report a combined DFT and experimental study on the mechanism of the arylation of 2‐phenylpyridines with Ar−Br catalyzed by cationic [Ru(2‐ppy)L_4_]^+^. In stark contrast to the latest proposals, our computed results show that bis‐cyclometalated Ru‐species offer a significantly lower energy pathway than mono‐cyclometalated Ru. Importantly, this conclusion is supported by experimental organometallic and kinetic studies that confirm the need for formation of bis‐cyclometalated species, providing excellent agreement between experiment and computation.

## Results and Discussion

**Figure 2:**
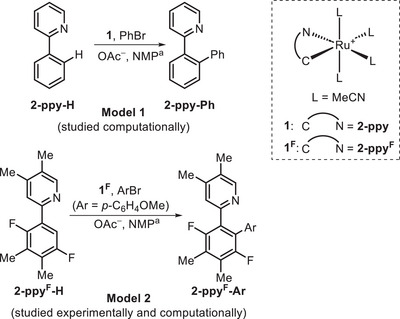
Models used in this study for computational and experimental work. ^a^NMP (*ε* = 32.2) is not available within Gaussian16 and so MeOH (*ε* = 32.6) was used in the calculations.

To draw comparison with literature work,^[^ ^39^ ^]^ computational studies considered the *ortho*‐arylation of 2‐phenylpyridine (**2‐ppy‐H**) with PhBr (Model 1, Figure 2) and used a PBE(def2‐tzvp, MeOH, D3‐BJ)//BP86(MeOH, SDD, 6–31g**) protocol which was adopted after extensive testing. For practical reasons the parallel experimental studies considered the arylation of a fluorinated substrate (**2‐ppy^F^‐H**) with 4‐bromoanisole (Model 2). Selected calculations were therefore also performed with Model 2 and showed that the key conclusions drawn from Model 1 are unaffected (see Supporting Information which also gives details of functional testing).

### Formation of Bis‐Cyclometalated Species

We began our study by examining this process computationally using Model 1, where the catalyst precursor used in previous experimental studies,^[^ ^35^ ^]^ **Ru‐1**, is employed, denoted as **1** in the computational study. All free energies are quoted relative to **1** set to 0.0 kcal mol^−1^, including any species necessary to maintain mass balance. Each stationary point in this study has several isomers and speciation studies were conducted throughout to identify the most favored pathways that are presented in the main text. Full details of alternative isomers and pathways are provided in the Supporting Information.

**Figure 3:**
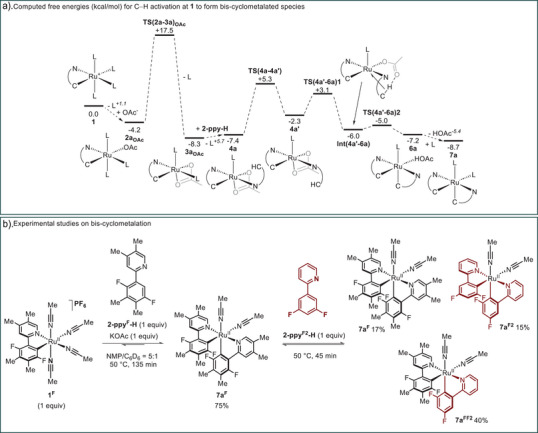
Studies on the formation of bis‐cyclometalated complexes. a) Computed free energies (kcal mol^−1^, L = MeCN) for C─H activation of **2‐ppy‐H** at **1** to form bis‐cyclometalated species **7a**. Method: PBE(def2‐tzvp, MeOH, D3‐BJ)//BP86(MeOH, SDD, 6–31G**); see Figures S13–S18 for speciation studies and alternative pathways. b) Experimental formation of bis‐cyclometalated complexes. Yields reported by quantitative in situ ^19^F NMR analysis with an internal standard.

The lowest energy pathway for the cyclometalation of **2‐ppy‐H** at **1** is shown in Figure 3a and proceeds via a series of ligand substitutions to form **4a** at −7.4 kcal mol^−1^. In some cases, substitution had to be treated as purely dissociative in nature, for example, loss of a MeCN ligand (L) *trans* to the aryl C in **1** gave a 5‐coordinate intermediate at +1.1 kcal mol^−1^ without a transition state; similarly, OAc^–^ addition to this intermediate is “barrierless” and forms **2a_OAc_** at −4.2 kcal mol^−1^. Such cases are shown with a dashed arrow in Figure 3a with the energy of the 5‐coordinate intermediate indicated. Substitution transition states can be located when ligand loss is accompanied by a change in binding mode of one of the other ligands, for example a κ^1^ to κ^2^ rearrangement of the OAc^–^ ligand in **TS(2a‐3a)_OAc_** at +17.5 kcal mol^−1^. Once formed, C─H activation at **4a** may occur either *trans* to aryl or *trans* to L. The former is markedly disfavored as it places two strong *trans*‐influence ligands opposite one another and involves a coplanar arrangement of cyclometalated ligands (see Figure S18a). C─H activation *trans* to L requires initial rotation of the **2‐ppy‐H** ligand to give rotamer **4a′** at −2.3 kcal mol^−1^. **4a′** can then access a two‐step AMLA/CMD process^[^ ^41^ ^]^ to give bis‐cyclometalated **6a** at −7.2 kcal mol^−1^ from which HOAc/L substitution forms **7a** at −8.7 kcal mol^−1^. C─H activation at **1** is therefore predicted to form **7a** directly with the largest barrier of 21.7 kcal mol^−1^ corresponding to the loss of MeCN in **2_OAc_** to form κ^2^‐OAc complex **3_OAc_**. In the absence of any onward reaction, protodemetallation to reform **4a** is accessible (Δ*G* ^‡^ = 14.0 kcal mol^−1^; Δ*G* = +1.3 kcal mol^−1^) and so C─H activation should be reversible.

To confirm the predicted formation of bis‐cyclometalated species of the type **7a**, we monitored the stoichiometric reaction of mono‐cyclometalated‐Ru species **1^F^** with substituted phenylpyridine **2‐ppy^F^‐H** (1 equiv) by in situ ^19^F NMR spectroscopy (Figure 3b). In full accordance with the calculations, **7a^F^** was formed in 2 h at 50 °C simply in the presence of 1 equiv of KOAc, demonstrating that bis‐cyclometalated species are not only energetically accessible under the reaction conditions but also the only species formed in observable quantities. Furthermore, addition of 1 equiv of the related phenylpyridine **2‐ppy^F2^‐H** to this mixture resulted in scrambling of the 2‐ppy ligands in **7a** as expected for a system in equilibrium.^[^ ^42^ ^]^

Key steps along the reaction profile shown in Figure 3 were recomputed with Model 2, featuring the substituted 2‐phenylpyridine **2‐ppy^F^‐H** as substrate and installed as the cyclometalated ligand in the catalyst **1^F^** (Figure S48). Model 2 shows the same pattern to Model 1 with each stationary point stabilized by ca. 4 kcal mol^−1^. The overall barrier via **TS(2a^F^‐3a^F^)_OAc_** is now 21.6 kcal mol^−1^ and leads to **7a^F^** at −12.8 kcal mol^−1^.

### Ph−Br Activation at Mono‐ and Bis‐Cyclometalated Ru Species: Experimental Studies

**Figure 4:**
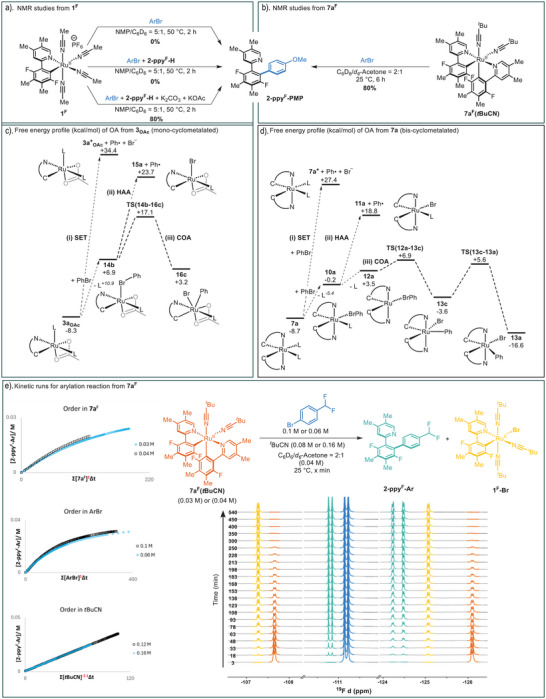
Experimental and computational studies on the oxidative addition step. a) Stoichiometric reaction of mono‐cyclometalated‐Ru **1^F^** with 4‐bromoanisole. b) Stoichiometric reaction of bis‐cyclometalated‐Ru **7a^F^** with 4‐bromoanisole. c) Lowest energy reaction profiles (free energies, kcal mol^−1^) computed for Ph─Br activation at mono‐cyclometalated species computed with Model 1. Method: PBE(def2‐tzvp, MeOH, D3‐BJ)//BP86(MeOH, SDD, 6–31G**). d) Lowest energy reaction profiles for Ph─Br activation at bis‐cyclometalated **7a** (Model 1). e) Kinetic studies of the reaction of bis‐cyclometalated‐Ru **7a^F^** with 4‐bromoanisole.

We began by testing the hypothesis of oxidative addition to a mono‐cyclometalated species (Figure 4a). In situ NMR monitoring of the reaction of **1^F^** with 4‐bromoanisole in NMP/C_6_D_6_, both in the presence and absence of KOAc, at 50 °C, over 2 h, revealed no arylation product formation and returned only unreacted starting materials. Similarly, heating up **1^F^** in the presence of an additional 1 equiv of **2‐ppy^F^‐H** led to no formation of arylation product, despite the observation of small amounts of a new complex that calculations indicate may arise from MeCN/**2‐ppy^F^‐H** substitution in **1^F^** (see Figure S48). These results strongly suggest that oxidative addition to mono‐cyclometalated species as proposed in Figure 1b is not a feasible process at 50 °C. However, when KOAc and K_2_CO_3_ were added to the mixture of **1^F^**, **2‐ppy^F^‐H** and 4‐bromoanisole the formation of bis‐cyclometalated species was observed, followed by its consumption to form arylation product **2‐ppy^F^‐Ar**. Importantly, no **2‐ppy^F^‐Ar** was observed before formation of bis‐cyclometalated‐Ru species **7a^F^** (see Supporting Information, Section 6) consistent with the latter being an intermediate en route to **2‐ppy^F^‐Ar**. We then moved to study the direct reaction of **7a^F^** with 4‐bromoanisole (Figure 4b). In this case, no additives were found to be necessary for reactivity, with **7a^F^** reacting smoothly at room temperature to give arylation product **2‐ppy^F^‐Ar** in 80% yield. This reaction is extremely clean, with no other species observed. When the reactivity of **7a^F^** toward 4‐bromoanisole was compared in the presence and absence of KOAc identical kinetics were observed (see Figures S10–S12), indicating that KOAc does not play a role in this oxidative addition process.

### Computational Studies

The computed energetics of Ph─Br activation at bis‐cyclometalated **7a** via the COA, SET, and HAA mechanisms are summarized in Figure 4d. SET from **7a** to PhBr gives the Ru(III) cation, **7a^+^**, along with initial formation of a [PhBr]^–^ radical anion. The latter, however, does not correspond to a local minimum but undergoes Ph─Br cleavage to form a Ph radical and Br^−^.^[^ ^43^, ^44^ ^]^ The overall free energy change for this process, Δ*G*, is +36.1 kcal mol^−1^. HAA requires initial L/PhBr dissociative substitution to form **10a** from which Ph─Br bond homolysis forms the neutral Ru(III) intermediate **11a** and a phenyl radical (Δ*G* = +27.5 kcal mol^−1^). In contrast to these high energy processes, COA proceeds from 5‐coordinate **12a** via **TS(12a‐13c)** at +6.9 kcal mol^−1^ to give a Ru(IV) intermediate, **13c**, at −3.6 kcal mol^−1^. **13c** displays a narrow *trans*‐C_aryl_–Ru–C_Ph_ angle of 142° that is bent toward the Br ligand and reflects a Jahn–Teller distortion away from an octahedral geometry associated with a d^4^ Ru(IV) center.^[^ ^38^, ^45^ ^]^ Isomerization via **TS(13c‐13a)** leads to **13a** at −16.6 kcal mol^−1^ with a similar *trans*‐C_aryl_–Ru–C_Ph_ angle of 145° but now with this unit bent away from Br. The Ru−Br distance shortens in this process from 2.92 to 2.62 Å due to reduced steric pressure and more efficient Br→Ru π‐donation. Of the three Ph─Br activation mechanisms, COA with an overall barrier relative to **7a** of 15.6 kcal mol^−1^ is clearly the most accessible kinetically and, after isomerization to **13a**, is thermodynamically downhill by 7.9 kcal mol^−1^. In contrast, the thermodynamics of SET (Δ*G* = +36.1 kcal mol^−1^) and HAA (Δ*G* = +27.5 kcal mol^−1^) mean that both these processes can be ruled out.

Ph─Br activation at the mono‐cyclometalated species **1** and **3_OAc_** was also computed to model reactivity in the absence and presence of OAc^–^, respectively. In both cases COA is the favored mechanism but for **1** this entails an overall barrier of 38.5 kcal mol^−1^ (see Figure S34). COA via **3_OAc_** is more accessible (Figure 4c) and involves a 6‐coordinate intermediate **14b** reacting via **TS(14b‐16c)** with an overall barrier of 25.4 kcal/mol;^[^ ^46^ ^]^ SET (Δ*G* = +42.7 kcal mol^−1^) and HAA (Δ*G* = +32.0 kcal mol^−1^) are again significantly higher in energy. Ph─Br activation is therefore much less accessible via a mono‐cyclometalated mechanism (Δ*G* ^‡^ = 25.4 kcal mol^−1^) than from bis‐cyclometalated **7a** (Δ*G* ^‡^ = 15.6 kcal mol^−1^). With Model 2 these barriers become 27.0  and 18.9 kcal mol^−1^. Both models therefore predict Ar─Br activation to be clearly more accessible via a bis‐cyclometalated species, the difference in the activation barriers, ΔΔ*G* ^‡^, being 9.8 kcal mol^−1^ (Model 1) and 8.1 kcal mol^−1^ (Model 2). The barrier of 27.0 kcal mol^−1^ computed with Model 2 is also consistent with the lack of room temperature reaction seen experimentally between **1^F^** and 4‐bromoanisole in the presence of acetate.

A kinetic study of the reaction of bis‐cyclometalated **7a^F^** (isolated as the ^t^BuCN adduct) with 4‐bromo(difluoromethyl)benzene revealed kinetic orders of 1 on both **7a^F^** and the bromoarene (Figure 4e). Remarkably, a kinetic order of −2 was also measured for *^t^* BuCN, the ligand on **7a^F^**. These experimental results are only consistent with the calculated COA pathway, which requires both supporting nitrile ligands to dissociate before accessing **TS(12a‐13a)**. In contrast, the lowest energy pathways obtained for HAA and SET would display orders on *^t^* BuCN of −1 and 0, respectively.

### Computational Modeling of C─C Bond Formation and Product Release

**Figure 5:**
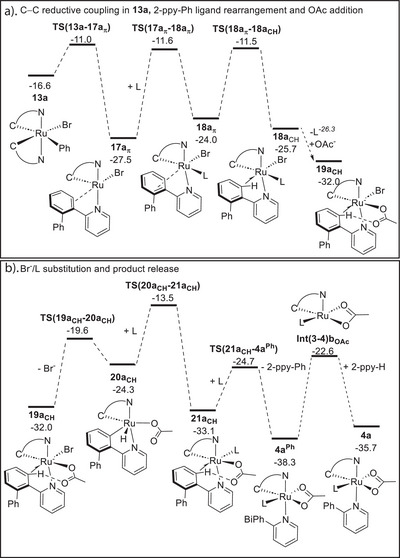
Computed free energies (kcal mol^−1^) for: a) C─C coupling in **13a** and formation of **19a_CH_**; and b) Subsequent release of the **2‐ppy‐Ph** product with reformation of **4a**. Method: PBE(def2‐tzvp, MeOH, D3‐BJ)//BP86(MeOH, SDD, 6–31G**).

Following isomerization of **13c** to **13a** (Figure 4d) C─C reductive coupling proceeds via **TS(13a‐17a_π_)** at −11.0 kcal mol^−1^ (Figure 5a). This forms **17a_π_** (−27.5 kcal mol^−1^) that features the arylation product, **2‐ppy‐Ph**, bound via N and a π‐interaction with the newly formed biphenyl moiety. Displacement of **2‐ppy‐Ph** and Br^–^ by OAc^–^ and L would reform **4a** that could then re‐enter the catalytic cycle. This may also involve rearrangement of the π‐bound biphenyl to a C─H agostic form that we find is more readily displaced by incoming ligands.^[^ ^47^ ^]^ Several pathways with different permutations of these steps were explored (see Figures S42–S47) and these identified **4a^Ph^** at −38.3 kcal mol^−1^ as the most stable κ^1^‐*N*‐bound **2‐ppy‐Ph** complex. Figure 5 shows the lowest energy pathway to this intermediate. Initial MeCN addition to **17a_π_** promotes the π‐ to C─H agostic rearrangement via **TS(18a_π_‐18a_CH_)** to form **18a_CH_** in which L/OAc^−^ substitution gives **19a_CH_** at −32.0 kcal mol^−1^ (Figure 5a). Br^−^/L substitution then proceeds via **20a_CH_** and **21a_CH_** with addition of MeCN to the latter leading to **4a^Ph^**. Dissociative **2‐ppy‐Ph**/**2‐ppy‐H** substitution then releases the product and forms **4a** (Figure 5b). Along this profile both **19a_CH_** and **21a_CH_** feature strong agostic C─H interactions and H‐bonding to the κ^1^‐OAc ligands. These species are therefore set up for C─H activation that could lead on to a second C─H arylation process. Although we have not studied it here, this would be consistent with the double‐arylated products commonly seen experimentally.^[^ ^26^, ^27^, ^28^, ^29^, ^30^, ^31^, ^32^, ^33^, ^34^, ^35^, ^36^, ^37^ ^]^ In contrast, the intervening intermediate, **20a_CH_**, shows Ru─hydride character (Ru─H = 1.55 Å, C⋯H = 1.99 Å; H⋯O = 2.39 Å), with Br^–^ dissociation from **19a_CH_** inducing C─H bond cleavage, and MeCN addition then reforming the agostic C─H bond in **21a_CH_**. The highest energy span along this profile corresponds to this net Br^–^/L substitution via **TS(20a_CH_‐21a_CH_)** with an overall barrier relative to **19a_CH_** of 18.5 kcal mol^−1^.

### Overall Profile

**Figure 6:**
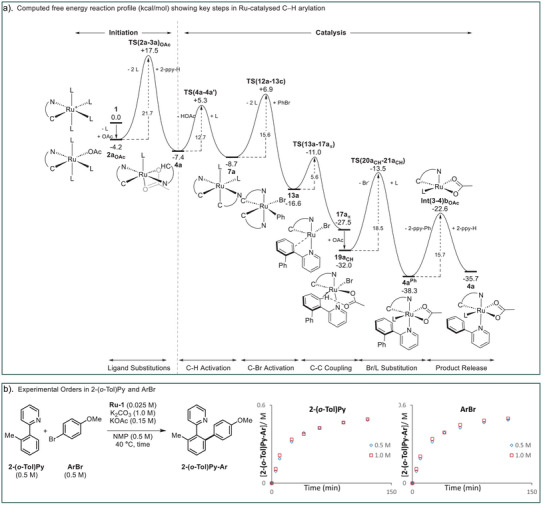
a) Computed reaction profile (free energies, kcal mol^−1^) summarizing key steps in the Ru‐catalyzed C─H arylation of **2‐ppy‐H** at catalyst **1** via bis‐cyclometalated intermediates. Method: PBE(def2‐tzvp, MeOH, D3‐BJ)//BP86(MeOH, SDD, 6–31G**); see Figures 3, 4, 5 for full details. b) Kinetic analysis of a catalytic arylation reaction revealing zero order on both substrates of the reaction.

Figure 6a summarizes the overall pathway computed for the catalytic C─H arylation of **2‐ppy‐H** by [Ru(2‐ppy)(MeCN)_4_]^+^, **1**, and highlights the key intermediates and transition states that define the free energy spans within each component of the reaction. Catalysis initiation from **1** entails substitution of three MeCN ligands by OAc^–^ and **2‐ppy‐H** to form **4a** at −7.4 kcal mol^−1^. The overall barrier for this initiation process is 21.7 kcal mol^−1^ and corresponds to MeCN dissociation in **2a_OAc_** via **TS(2a‐3a)_OAc_**. Catalysis then proceeds via C─H activation in **4a** followed by HOAc/MeCN substitution to give bis‐cyclometalated **7a** at −8.7 kcal mol^−1^. C─Br activation proceeds via the COA transition state **TS(12a‐13c)** (+6.9 kcal mol^−1^) to form Ru(IV) intermediate **13a**. This C─Br activation has an overall barrier of 15.6 kcal mol^−1^ relative to **7a** and first requires the displacement of two MeCN ligands by Ph─Br. This is consistent with the inverse second order in the nitrile ligands determined experimentally via stoichiometric reactions of **7a^F^**. Facile C─C reductive coupling then forms the **2‐ppy‐Ph** product bound in a κ‐*N*, π‐fashion to Ru in **17a_π_** (−27.5 kcal mol^−1^). The subsequent product release is then a multiple step process in which the largest energy span of 18.5 kcal mol^−1^ is associated with Br^–^/L substitution in **19a_CH_** via **TS(20a_CH_‐21a_CH_)**. Dissociative substitution of **2‐ppy‐Ph** in **4a^Ph^** by further **2‐ppy‐H** substrate proceeds via **Int(3–4)b_OAc_** at −22.6 kcal/mol^−1^ to regenerate **4a**. Overall, the catalytic arylation of **2‐ppy‐H** to **2‐ppy‐Ph** is exergonic by 28.3 kcal mol^−1^. Within the catalytic cycle, Br^–^/L substitution in **19a_CH_** represents the largest energy span with a barrier of 18.5 kcal mol^−1^. The barrier for this step reflects the difficulty in dissociating a ligand *trans* to a low *trans*‐influence agostic interaction; similarly MeCN loss via **TS(2a‐3a)_OAc_** in the initiation process occurs *trans* to a relatively weak *trans*‐influence pyridyl nitrogen.

The rate‐limiting Br^–^/L substitution step predicted by our computed reaction profile indicates that the catalytic arylation reaction should display kinetic orders of zero for both substrates, 2‐arylpyridine and ArBr. This is in stark contrast to previously calculated reaction profiles for ruthenium arylation, where oxidative addition was invariably shown as the largest barrier to overcome, with a predicted kinetic order for Ar─Br of one. In order to test our computational prediction, we carried out a kinetic study of the reaction of 2‐*ortho*‐tolylpyridine with 4‐bromoanisole using the mono‐cyclometalated **Ru‐1** as catalyst under standard reaction conditions (Figure 6b).^[^ ^35^ ^]^ Remarkably, we obtained kinetic orders of zero for both substrates, in full consistency with the computed reaction profile.

## Conclusions

In conclusion, we report a combination of experimental and computational mechanistic studies that unravel the essential role of bis‐cyclometalated Ru species in the Ru(II)‐mediated C─H arylation of 2‐phenylpyridines with bromoarenes. Our work conclusively reveals that these reactions proceed via the concerted oxidative addition of the bromoarene at a bis‐cyclometalated Ru(II) complex, rather than the previously proposed COA or HAA processes on mono‐cyclometalated Ru(II).

Modeling studies on the full catalytic cycle show the key bis‐cyclometalated precursor to Ar─Br activation is formed via a facile CMD/AMLA C─H activation and that the subsequent C─C coupling is also a low energy process. The overall rate‐limiting step in the catalytic phenylation of 2‐phenylpyridine corresponds to a Br^–^/MeCN substitution step that is part of a multistep product release process. This is also corroborated by the zero‐order kinetics determined for both substrates. Our study identifies the key role of bis‐cyclometalated species in promoting Ar─Br activation and provides the detailed mechanistic insight that will underpin the design of more efficient Ru arylation catalysts. It also highlights how the detailed interplay of computational and experimental mechanistic studies is necessary to provide reliable mechanistic insight in this multifaceted system that exhibits mechanistic and speciation diversity.

## Supporting Information

The data that support the findings of this study are available in the Supporting Information of this article.

## Conflict of Interests

The authors declare no conflict of interest.

## Supporting information



Supporting Information

## Data Availability

The data that support the findings of this study are available in the Supporting Information of this article.
