# Supplementary material for: The Mechanism of Ruthenium‐Catalyzed Directed C─H Arylation of Arenes: The Key Role of Bis‐Cyclometalated Intermediates
Source: Angew Chem Int Ed Engl. 2025 May 6;64(26):e202506707. doi: 10.1002/anie.202506707 (PMC12184301; doi:10.1002/anie.202506707)
Supplement: Supplementary file 1 — Supporting Information [file ANIE-64-e202506707-s001.pdf]

Supporting Information for:

The Mechanism of Ruthenium-Catalyzed Directed C–H Arylation of Arenes: The Key Role of Bis-Cyclometalated Intermediates

Pablo Domingo-Legarda,[a] Samuel E. Neale,[b] Ambre Carpentier,[b] Claire L. McMullin,[b,†] Michael Findlay,[a] Igor Larrosa*,[a] and Stuart A. Macgregor*,[c]

[a] School of Chemistry, University of Manchester, Oxford Road, Manchester M13 9PL, U.K.

[b] Institute of Chemical Sciences, Heriot-Watt University, Edinburgh EH14 4AS, U.K.

[c] EaStCHEM School of Chemistry, North Haugh, University of St. Andrews, KY16 9ST, U.K.

[†]Present Address: Department of Chemistry, University of Bath, Claverton Down, Bath, BA2 7AY U.K.

Contents

1. General Information 4

2. Preparation of 2-(2,5-difluoro-3,4-dimethylphenyl)-4,5-dimethylpyridine (2-ppy^{F}-H) 4

3. Preparation of 2-(3,5-difluorophenyl)pyridine (2-ppy^{F2}-H) 4

4. General procedures 5

4.1. General procedure A: synthesis of monocyclometalated species 1^{F} and 1^{F2} 5

4.2. General procedure B: synthesis of biscyclometalated species 7a^{F} and 7a^{F2} 6

4.3. General procedure C: C-H arylation of 2-ppy^{F}-H 7

5. Determination of order in reagents 8

5.1. Determination of the order in 7a^{F} 9

5.2. Determination of the order in Arylbromide 10

5.3. Determination of the order in pivalonitrile 11

6. Kinetic analysis from 1^{F} 12

6.1. Reaction in presence of 2-ppy^{F}-H, KOAc and K_2CO_3 and then, addition of 4-bromoanisole 12

6.2. Reaction in presence of 2-ppy^{F}-H in absence of KOAc and K_2CO_3, and then, addition of 4-bromoanisole 13

6.3. Reaction in presence of 2-ppy^{F}-H, KOAc, K_2CO_3 and 4-bromoanisole 14

6.4. Reaction between complex 1^{F} and 4-bromoanisole, and then addition of 2-ppy^{F}-H 15

6.5. Reaction between complex 1^{F} and 4-bromoanisole in the presence of AcOK, and then addition of 2-ppy^{F}-H 16

7. Scrambling Experiment 17

8. Effect of AcOK in the reaction kinetics 18

9. NMR Spectra of pure compounds 21

10. Computational Details 46

11. C–H Arylation via bis-cyclometalated intermediates. 47

11.1. Formation of isomers of 4 from 1. 47

11.2 C–H activation pathways to form bis-cyclometalated intermediates. 53

11.3 Ph–Br activation at bis-cyclometalated intermediates 57

12. C–H Arylation via mono-cyclometalated intermediates 64

12.1. Ph–Br activation in the presence of OAc^- 65

12.2. Ph–Br activation in the absence of OAc^- 71

13. C–C coupling and product release. 76

13.1. Initial reactions of 17a$_{\pi}$. 79

13.2. Product release mechanisms 80

14. Calculations with Model 2. 86

14.1 C–H Activation 86

14.2 C–Br Activation 87

15. Computed energies (au) and Cartesian coordinates of all species. 89

15.1 Model 1. 89

15.2 Model 2. 257

16. References 272

1. General Information

NMR experiments were carried out using J-Young type NMR tubes, and the samples were prepared inside an Argon-filled glove box. All the arylation reactions were carried out in an Argon-filled glove box with oven-dried crimp-cap microwave vials (10 mL). All the solvents were purchased from Acros (Fisher), Aldrich (Merck), Alfa Aesar (Fisher), Fluorochem, Generon, Manchester Organics, Molekula, MP Biomedicals and TCI, and used without further purification. The solvents used in NMR studies (NMP, d_6-Benzene and d_6-Acetone) were dried over 4Å activated molecular sieves for a period of at least 72 hours. Column chromatography was carried out on silica gel, particle size 40-63 µm, using flash techniques. High resolution mass spectra were performed by the School of Chemistry Mass Spectrometry Service (University of Manchester) employing a Thermo Finnigan MAT95XP spectrometer. Mass spectra for the characterization of ruthenium complexes were performed by the School of Chemistry Mass Spectrometry Service (University of Manchester) employing a Waters SQD2 spectrometer. ^{1}H NMR, ^{19}F NMR and ^{13}C NMR spectra were recorded at 400 or 500 MHz on Bruker machines. Kinetic analysis spectra were recorded at 700 MHz Bruker machine. ^{1}H NMR are referenced to the residual solvent peak at 7.26 ppm ($CDCl_3$), 7.16 ppm (C_6D_6), 1.94 ppm (CD_3CN), and quoted in ppm to 2 decimal places with coupling constants (J) to the nearest 0.1 Hz. ^{13}C NMR spectra, recorded at 100 MHz or 126 MHz, are referenced to the solvent peak at 77.00 ppm ($CDCl_3$), 128.16 ppm (C_6D_6), 1.32 ppm (CD_3CN), and quoted in ppm to 1 decimal place with coupling constants (J) to the nearest 0.1 Hz. ^{19}F NMR spectra were recorded at 376 or 471 MHz in $CDCl_3$, C_6D_6, CD_3CN, and quoted in ppm to 2 decimal places and with coupling constants (J) to the nearest 0.1 Hz. For the kinetic analysis spectra 1,3,5-trifluorobenzene and trifluoromethoxybenzene were used as internal standards.

2. Preparation of 2-(2,5-difluoro-3,4-dimethylphenyl)-4,5-dimethylpyridine (2-ppy^{F}-H)

The synthesis protocol described by Larrosa and co-workers[1], was followed to synthesise **2-ppy^{F}-H**. The spectroscopical data match with the one described in the literature.

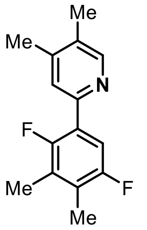

^{1}H NMR (400 MHz, $CDCl_3$) δ 8.42 (s, 1H), 7.56 (d, J = 2.3 Hz, 1H), 7.46 (dd, J = 10.2, 6.4 Hz, 1H), 2.34 (s, 3H), 2.30 (s, 3H), 2.27 (d, J = 2.6 Hz, 3H), 2.25 (d, J = 2.1 Hz, 3H). **^{13}C NMR** (101 MHz, $CDCl_3$) δ 157.1 (dd, J = 237.5, 2.1 Hz), 154.4 (dd, J = 240.9, 2.1 Hz), 150.7, 149.9, 145.9, 131.2, 125.8 (dd, J = 19.6, 4.5 Hz), 125.6 – 125.3 (m, 2C), 125.1 (d, J = 9.4 Hz), 113.3 (dd, J = 27.1, 4.1 Hz), 19.4, 16.2, 11.4 (dd, J = 6.6, 2.1 Hz), 11.3 (dd, J = 4.5, 2.0 Hz). **^{19}F NMR** (376 MHz, $CDCl_3$) δ -122.33 (d, J = 18.5 Hz), -125.80 (d, J = 18.5 Hz).

3. Preparation of 2-(3,5-difluorophenyl)pyridine (2-ppy^{F2}-H)

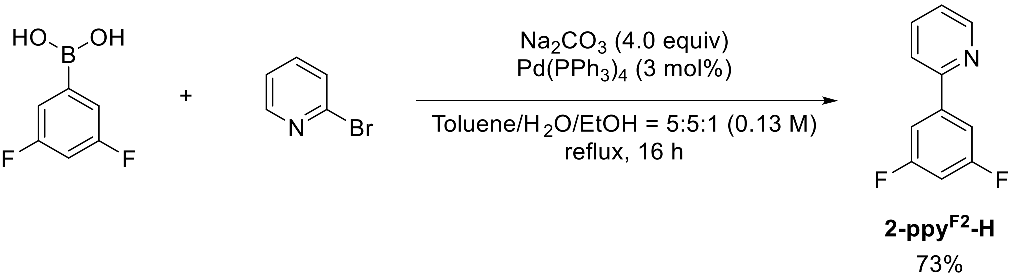

2-ppy^{F2}-H
73%

Scheme S1. Synthesis of **2-ppy^{F2}-H**.

A 250 mL two-necked flask was charged with Na_2CO_3 (8.4 g, 80.0 mmol, 4.0 equiv), 3,5-difluorophenyl boronic acid (3.5 g, 22.0 mmol, 1.1 equiv), $Pd(PPh_3)_4$ (694 mg, 0.60 mmol, 0.03 equiv.) and a stirrer bar, under N_2 atmosphere. Then, previously deoxygenated toluene (70.0 mL), water (70.0 mL) and ethanol (14.0 mL) were added to the mixture, followed by 2-bromopyridine (1.9 mL, 20.0 mmol, 1.0 equiv). The mixture was stirred under reflux for 16 hours. Then, the reaction was cooled down to room temperature and quenched with 30 mL of a saturated solution of NH_4Cl. The aqueous phase was extracted with ethyl acetate (3×50 mL), and the combined organic phase was washed with brine (50 mL), dried over $MgSO_4$ and concentrated under reduced pressure. The crude mixture was purified by flash column chromatography on silica gel using a 9:1 mixture of *n*-hexane/EtOAc as eluent, affording 2-(3,5-difluorophenyl)pyridine as a white crystalline solid (2.8 gr, 73%). Spectroscopy data match perfectly with the one described in the literature.[2] **^{1}H NMR** (400 MHz, $CDCl_3$) δ 8.70 (ddd, J = 4.9, 1.8, 0.9 Hz, 1H), 7.79 (td, J = 7.7, 1.8 Hz, 1H), 7.69 (dt, J = 8.0, 1.1 Hz, 1H), 7.60 – 7.50 (m, 2H), 7.29 (ddd, J = 7.5, 4.8, 1.1 Hz, 1H), 6.85 (tt, J = 8.7, 2.4 Hz, 1H). **^{13}C NMR** (101 MHz, $CDCl_3$) δ 163.4 (dd, *J* = 247.9, 12.7 Hz, 2C), 154.8 (t, *J* = 3.0 Hz), 149.8, 142.7 (t, *J* = 9.3 Hz), 137.0, 123.2, 120.5, 110.4 – 108.2 (m, 2C), 104.1 (t, *J* = 25.6 Hz). **^{19}F NMR** (376 MHz, $CDCl_3$) δ -109.59.

4. General procedures

4.1. General procedure A: synthesis of monocyclometalated species 1^F and 1^{F2}

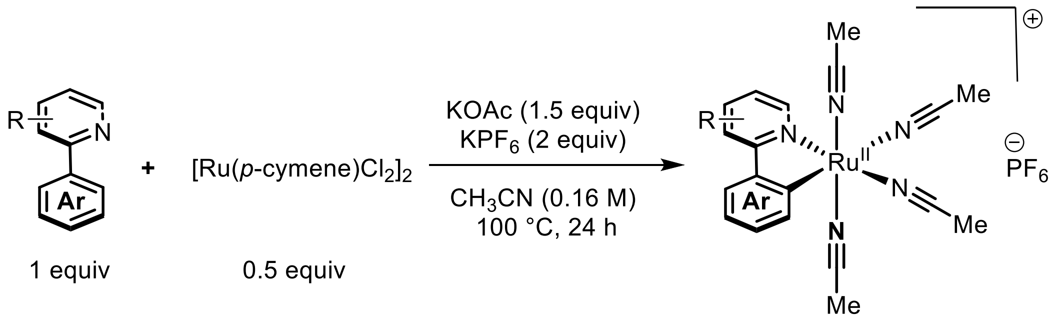

Scheme S2. General procedure A: Synthesis of monocyclometalated species.

The procedure described by Larrosa and co-workers was used:[1]

Liquid reagents and solvents were dried over 4 Å molecular sieves and degassed with 3 freeze-pump-thaw cycles prior to use. KOAc was dried at 140 °C in a vacuum oven for 48 h prior to use. To an oven dried 100 mL ace pressure tube equipped with a stirring bar, inside to an argon filled glovebox, 490 mg of $[RuCl_2(p\text{-cymene})]_2$ (489.9, 0.8 mmol, 0.5 equiv), 236 mg of KOAc (2.4 mmol, 1.5 equiv), 590 mg of KPF_6 (3.2 mmol, 2 equiv), the corresponding 2-Arylpyridine (1.6 mmol, 1 equiv) and MeCN (10 mL, 0.16 M) were added. The tube was sealed, transferred out of the glovebox and heated at 100 °C for 24 h. Upon completion, the reaction crude was purified via flash chromatography, using aluminium oxide (Al_2O_3, neutral) conditioned with DCM as solid phase and quickly eluted with MeCN/DCM (1:1) using N_2 in replacement of air collecting the yellow/orange band. The solution was concentrated under reduced pressure and precipitated with Et_2O affording the desired Ru-complexes. **These complexes have to be promptly transferred to a glove box as they decompose if exposed to air.**

The General procedure A was applied, using 2-(2,5-difluoro-3,4-dimethylphenyl)-4,5 dimethylpyridine

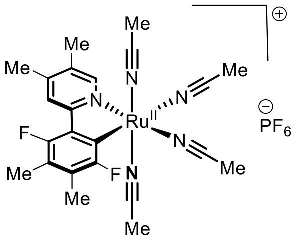

(396 mg, 1.6 mmol, 1.0 equiv), affording complex **1^{F}** as a yellow solid (915 mg, 87%). Spectroscopy data match perfectly with the one described in the literature.[1] **^{1}H NMR** (400 MHz, CD_3CN) δ 8.65 (s, 1H), 8.01 (d, J = 2.5 Hz, 1H), 2.43 (s, 3H), 2.39 (s, 3H), 2.31 (s, 3H), 2.22 (d, J = 2.3 Hz, 3H), 2.20 (d, J = 2.5 Hz, 3H), 2.05 (s, 6H), 1.96 (s, 3H). **^{13}C NMR** (176 MHz, CD_3CN) δ 167.7 (d, *J* = 224.6 Hz), 164.4 (d, *J* = 7.5 Hz), 160.4 (dd, *J* = 51.3, 2.5 Hz), 156.0 (dd, *J* = 248.1, 1.6 Hz), 152.6, 148.1 (d, *J* = 1.4 Hz), 133.8 (dd, *J* = 19.5, 6.2 Hz), 131.6 (d, *J* = 1.4 Hz), 124.8 (dd, *J* = 26.2, 4.8 Hz), 124.1, 124.0, 123.9, 122.5, 121.9, 19.7, 16.5, 11.9 (dd, *J* = 5.0, 1.9 Hz), 11.0 (dd, *J* = 7.3, 2.2 Hz), 4.0, 3.9 (signals for both carbons of one of the MeCN ligands were not observed). **^{19}F NMR** (376 MHz, CD_3CN) δ -72.93 (d, J = 706.4 Hz), -106.67 (dq, J = 23.0, 2.4 Hz), -124.46 (dp, J = 23.0, 2.7 Hz).

The General procedure A was applied, using 2-(3,5-difluorophenyl)pyridine (305 mg, 1.6 mmol, 1.0

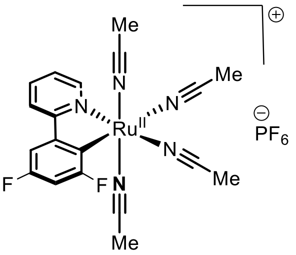

equiv), affording complex **1^{F2}** as an orange solid (873 mg, 91%). Decomposes at 180 °C. **^{1}H NMR** (400 MHz, CD_3CN) δ 8.97 (dt, J = 5.6, 1.2 Hz, 1H), 7.89 (dt, J = 8.2, 1.2 Hz, 1H), 7.80 (td, J = 7.5, 1.5 Hz, 1H), 7.46 (dd, J = 10.1, 2.4 Hz, 1H), 7.24 (ddd, J = 7.5 5.6, 1.5 Hz, 1H), 6.69 (ddd, J = 10.1, 8.2, 2.4 Hz, 1H), 2.44 (s, 3H), 2.04 (s, 6H), 1.96 (s, 3H). **^{13}C NMR** (101 MHz, CD_3CN) δ 173.9 (dd, *J* = 234.5, 11.1 Hz), 168.1 (d, *J* = 10.2, 2.8 Hz), 160.7 (dd, *J* = 234.5, 12.2 Hz), 156.3 (dd, *J* = 94.0, 6.0 Hz), 153.8, 150.8 (dd, *J* = 21.7, 8.4 Hz), 137.5, 124.5, 123.3, 122.8, 120.0, 107.4 (dd, *J* = 21.0, 3.7 Hz), 103.7 (dd, *J* = 34.7, 23.9 Hz), 4.0, 3.9 (signals for both carbons of one of the MeCN ligands were not observed). **^{19}F NMR** (376 MHz, CD_3CN) δ -72.86 (d, *J* = 706.5 Hz), -96.04 (dd, *J* = 8.2, 5.9 Hz), -123.90 (dd, *J* = 10.0, 5.9 Hz). **HRMS** calculated for $[M-CH_3CN-PF_6]^{+}$: 415.0308. found: 415.0312.

4.2. General procedure B: synthesis of biscyclometalated species 7a^{F} and 7a^{F2}

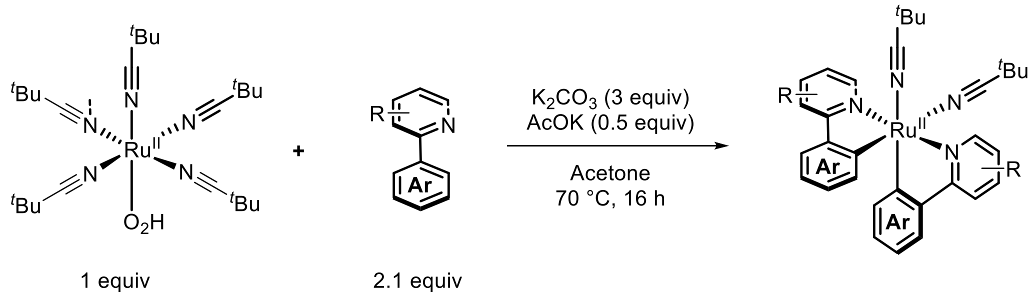

Scheme S3. General procedure B: Synthesis of biscyclometalated species.

A modification of the method used by Larrosa and co-workers was used:[3]

In an argon filled glovebox, to a microwave vial fitted with a magnetic stirrer bar, 142 mg of RuAqua (0.20 mmol, 1.00 equiv), the corresponding 2-Arylpyridine (0.42 mmol, 2.10 equiv), 83 mg of K_2CO_3 (0.60 mmol, 3.00 equiv), 10 mg of AcOK (0.60 mmol, 3.00 equiv) and 4.0 mL of a dry Acetone were added. The resulting mixture was capped and stirred at 70 °C for 16 hours. Upon completion, the reaction crude was diluted with acetone and filtered through 2 PFT filters. The solution was concentrated under reduced pressure till dryness and the resulting residue was redissolved in a minimum amount of benzene. The complexes were then precipitated after the addition of hexane as

deep red solids. **Biscycloruthenated complexes must be kept in a glovebox as they quickly decompose if exposed to air.**

The General procedure B was applied, using 2-(2,5-difluoro-3,4-dimethylphenyl)-4,5 dimethylpyridine (104 mg, 0.42 mmol, 2.10 equiv), affording complex **7a^F** as a deep red solid (88 mg, 0.12 mmol, 58%). Spectroscopy data match perfectly with the one described in the literature.[3] **^{1}H NMR** (400 MHz, C_6D_6) δ 9.27 (s, 2H), 8.51 (s, 2H), 2.04 (s, 12H), 2.01 (s, 6H), 1.82 (d, *J* = 2.3 Hz, 6H), 0.67 (s, 18H). **^{13}C NMR** (176 MHz, C_6D_6) δ 170.5 (d, *J* = 54.9 Hz), 167.4 (d, *J* = 224.2 Hz), 164.9 (d, *J* = 7.4 Hz), 156.5 (d, *J* = 247.4 Hz), 151.1, 141.7, 133.1 (dd, *J* = 21.5, 4.7 Hz), 128.3, 127.3, 123.3 (d, *J* = 22.1 Hz), 122.3 (dd, *J* = 26.8, 4.6 Hz), 113.8 (dd, *J* = 20.0, 3.9 Hz), 28.1, 19.3, 16.5, 11.8 (dd, *J* = 4.6, 1.7 Hz), 10.9 (dd, *J* = 7.3, 2.2 Hz). **^{19}F NMR** (376 MHz, C_6D_6) δ -107.60 (d, *J* = 23.8 Hz), -125.37 (d, *J* = 23.8 Hz).

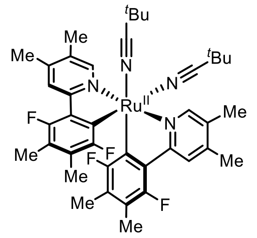

The General procedure B was applied, using 2-(3,5-difluorophenyl)pyridine (80.2 mg, 0.42 mmol, 2.10 equiv), affording complex **7a^{F2}** as a deep red solid (71 mg, 0.11 mmol, 55%). **^{1}H NMR** (400 MHz, C_6D_6) δ 9.39 (d, *J* = 5.6 Hz, 2H), 7.43 – 7.35 (m, 4H), 7.11 (t, *J* = 7.8 Hz, 2H), 6.75 (t, *J* = 6.6 Hz, 2H), 6.46 (ddd, *J* = 10.1, 7.8, 2.3 Hz, 2H), 0.55 (s, 18H). **^{13}C NMR** (176 MHz, C_6D_6) δ 172.8 (dd, *J* = 233.9, 10.6 Hz), 167.5 (d, *J* = 5.1 Hz), 164.8 (d, *J* = 49.0 Hz), 158.7 (dd, *J* = 233.0, 11.8 Hz), 151.3, 149.5 (d, *J* = 56.4 Hz), 148.7 (dd, *J* = 23.7, 8.1 Hz), 131.6, 119.6, 117.8, 105.6 (dd, *J* = 19.9, 3.5 Hz), 102.0 (dd, *J* = 35.1, 23.3 Hz), 27.5. **^{19}F NMR** (376 MHz, C_6D_6) δ -96.72 (dd, *J* = 7.7, 5.2 Hz), -125.58 (td, *J* = 10.0, 5.2 Hz). **HRMS**. (*pivalonitriles were exchanged by the acetonitrile in the mobile phase*) $[M(CH_3CN)]^+$: $[M-2xCH_3CN]^+$: calculated: 481.9975. found: 481.9955.

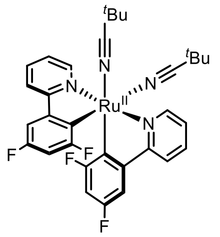

4.3. General procedure C: C-H arylation of 2-ppy^F-H

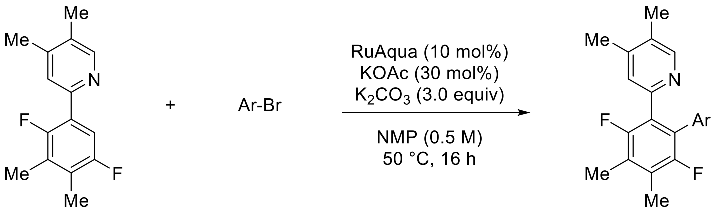

Scheme S4. General procedure C: Synthesis of arylated 2-arylpyridines.

10 ml Schlenk tube with a stirring bar was charged with RuAqua (28.4 mg, 0.04 mmol, 10.0 mol%), KOAc (12.0 mg, 0.12 mmol, 30.0 mol%), K_2CO_3 (166 mg, 1.20 mmol, 3.00 equiv) and **2-ppy^F-H** (99.0 mg, 0.40 mmol, 1.00 equiv), after 3 x 5 min evacuation/refill cycles, the appropriate degassed aryl halide (0.80 mmol, 2.0 equiv) and degassed NMP (0.8 mL, 0.5 M with respect to **2-ppy^F-H**) were added to the Schlenk tube. The reaction was then stirred at 50 degrees for 16 hours. Upon completion, the crude mixture was purified by flash column chromatography on silica gel, using a 9:1 mixture of hexane/AcOEt as eluent.

2-(3,6-difluoro-4'-methoxy-4,5-dimethyl-[1,1'-biphenyl]-2-yl)-4,5-dimethylpyridine (2-ppy^F-PMP).

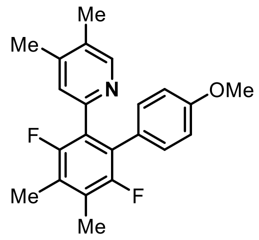

Genaral procedure C was followed, using 4-bromoanisole as the arylating agent (138 µL, 0.80 mmol, 2.00 equiv). The desired product was obtained as a white solid. 92.0 mg, 65% yield. **mp** = 89-92 °C. **^{1}H NMR** (400 MHz, $CDCl_3$) δ 8.29 (s, 1H), 7.05 (d, J = 8.8 Hz, 2H), 6.79 (s, 1H), 6.75 (d, J = 8.8 Hz, 2H), 3.77 (s, 3H), 2.31-2.28 (m, 6H), 2.21 (s, 3H), 2.13 (s, 3H) **^{13}C NMR** (101 MHz, $CDCl_3$) δ 158.7, 154.5 (dd, J = 241.8, 4.6 Hz), 154.1 (dd, J = 239.8, 3.9 Hz), 151.5 (d, J = 2.4 Hz), 149.5, 145.3, 131.8 (d, J = 1.3 Hz), 130.5, 126.9, 126.4 (dd, J = 10.5, 3.9 Hz), 126.3 (d, J = 2.1 Hz), 126.2 (dd, J = 14.8, 3.6 Hz), 125.1 (dd, J = 19.2, 5.5 Hz), 124.3 (dd, J = 18.9, 5.0 Hz), 113.2, 55.2, 19.2, 16.3, 11.6 (dd, J = 5.5, 2.4 Hz), 11.4 (dd, J = 5.3, 2.3 Hz). **^{19}F NMR** (376 MHz, $CDCl_3$) δ -123.95 (d, J = 18.0 Hz), -124.09 (d, J = 18.0 Hz). **HRMS** calcd for $C_{22}H_{22}F_2NO$ [M+H]: 354.1664, found 354.1652.

2-(4'-(difluoromethyl)-3,6-difluoro-4,5-dimethyl-[1,1'-biphenyl]-2-yl)-4,5-dimethylpyridine (2-ppy^F-Ar).

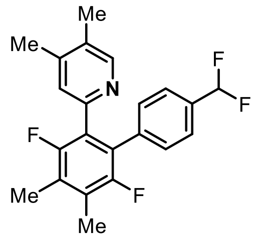

Genaral procedure C was followed, using (4-difluoromethyl)bromobenzene as the arylating agent (103 µL, 0.80 mmol, 2.00 equiv). The desired product was obtained as a white solid. 106 mg, 71% yield. **mp** = 101-103 °C. **^{1}H NMR** (400 MHz, $CDCl_3$) δ 8.25 (s, 1H), 7.36 (d, J = 7.8 Hz, 2H), 7.23 (d, J = 7.8 Hz, 2H), 6.83 (s, 1H), 6.60 (t, J = 56.6 Hz, 1H), 2.30 (t, J = 2.3 Hz, 6H), 2.20 (s, 3H), 2.13 (s, 3H). **^{13}C NMR** (101 MHz, $CDCl_3$) δ 154.4 (dd, J = 241.5, 3.9 Hz), 153.8 (dd, J = 241.2, 3.9 Hz), 150.8 (d, J = 1.7 Hz), 149.5, 145.4, 136.7 (d, J = 1.9 Hz), 132.8 (t, J = 22.3 Hz), 130.9, 130.8, 126.7, 126.1 (dd, J = 14.9, 7.8 Hz), 125.6 (dd, J = 17.1, 5.5 Hz), 125.51 – 125.10 (m, 2C), 124.9 (t, J = 6.0 Hz), 114.7 (t, J = 238.7 Hz), 19.1, 16.2, 11.5 (dd, J = 5.3, 3.0 Hz), 11.4 (dd, J = 4.9, 3.2 Hz). **^{19}F NMR** (376 MHz, $CDCl_3$) δ -123.72 (d, J = 18.0 Hz), -123.78 (d, J = 18.0 Hz). **HRMS** calcd for $C_{22}H_{20}F_4N$ [M+H]: 374.1513, found 374.1526.

5. Determination of order in reagents

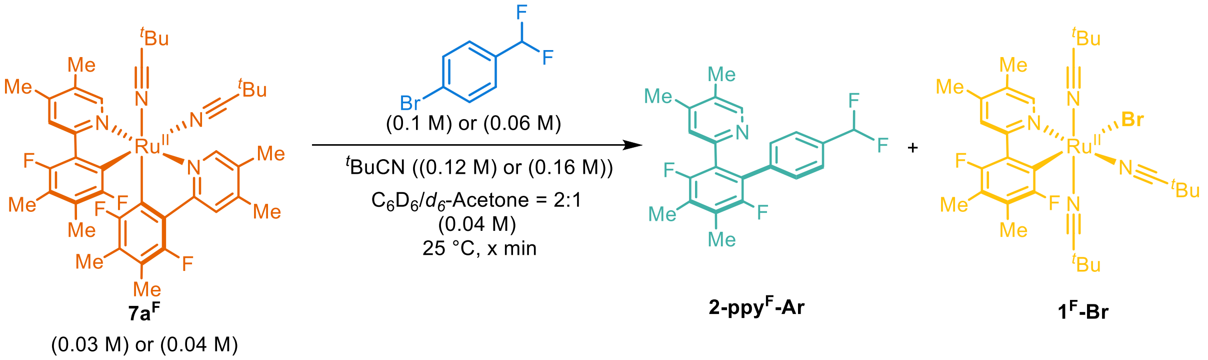

The orders in **7a^F**, aryl bromide and pivalonitrile were determined using normalized time scale analysis.[4-6] **7a^F** was weighed out in an argon-filled glove box into a Young's Tap NMR tube (19.0 mg, 0.025 mmol, 1.00 equiv or 14.0 mg, 0.019 mmol, 0.75 equiv). Then, a stock solution of pivalonitrile in *d_6*-benzene was added (0.075 mmol, 3.00 equiv or 0.10 mmol, 4.00 equiv), followed by the addition of 100 µL of a stock solution of trifluoromethoxybenzene (0.025 mmol, 1.00 equiv), *d_6*-benzene (to fill to a total volume of 300 µL) and 150 µL *d_6*-acetone. Then, a NMR spectrum was recorded, and the tube was introduced again inside the glove box, when 50 µL of *d_6*-acetone and a stock solution of 4-(difluoromethyl)bromobenzene were added (0.0375 mmol, 1.50 equiv or 0.0625 mmol, 2.50 equiv). The tube was frozen in a dry ice/acetone bath to prevent the reaction to occur before the NMR

recording take place. Then, the reaction was monitored by NMR, at 25 degrees, using trifluoromethoxybenzene as internal standard.

5.1. Determination of the order in 7a^F

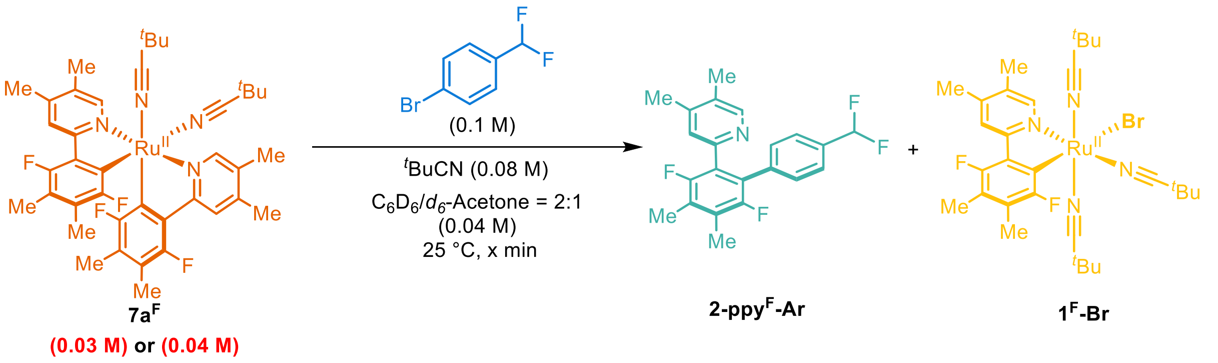

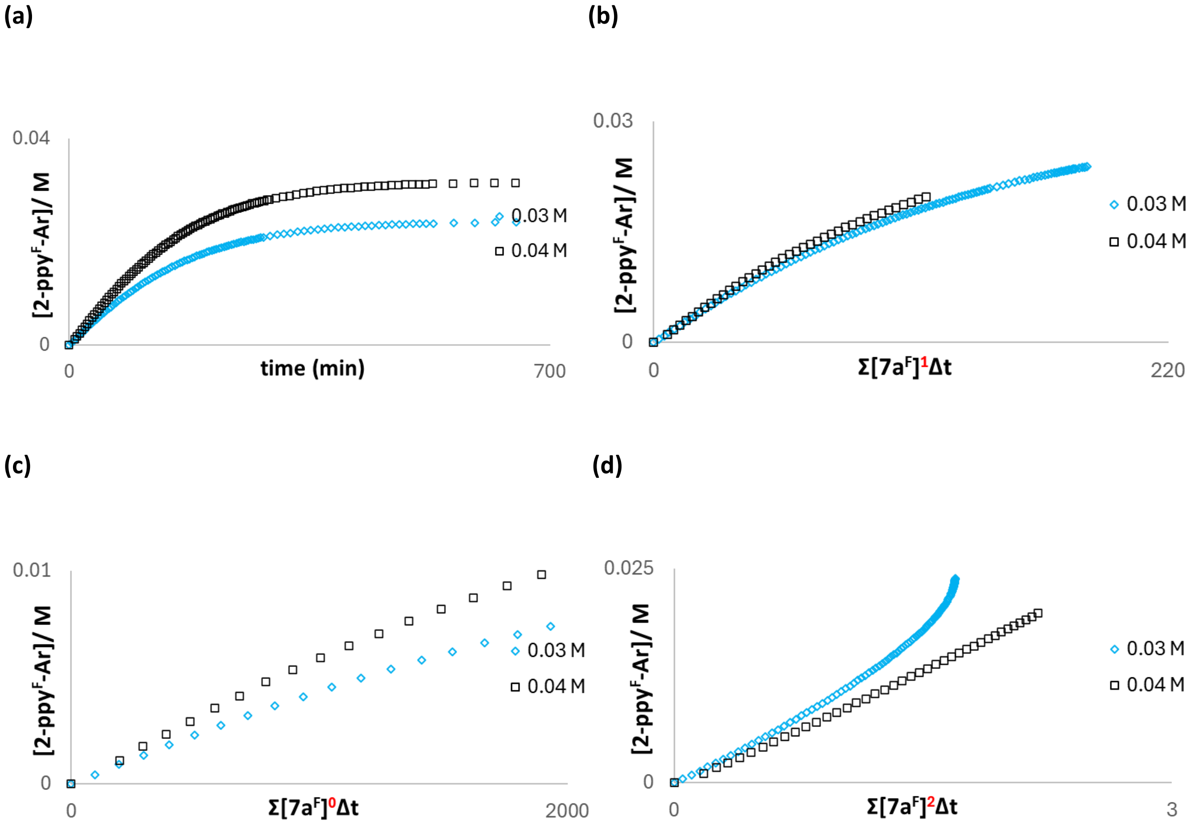

Figure S1. Determination of order in **7a^F**. (a) Temporal reaction profiles of reactions carried out with 0.025/0.019 mmol of **7a^F**; (b) Normalized time scale profiles for order 1.0 in [**7a^F**]; (c) Normalized time scale profiles for order 0 in [**7a^F**]; (d) Normalized time scale profiles for order 2.0 in [**7a^F**].

The overlap between normalised time scale reaction profiles for these two reactions with differing concentrations of **7a^F** shows an order of 1.

5.2. Determination of the order in Arylbromide

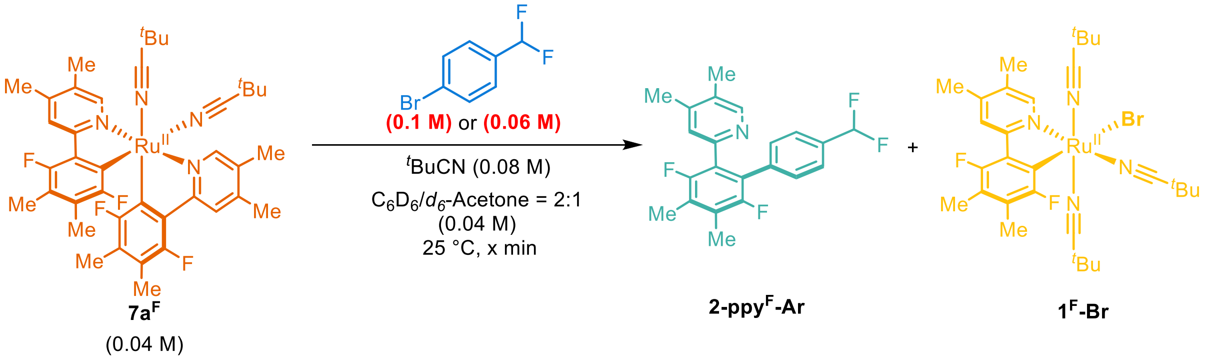

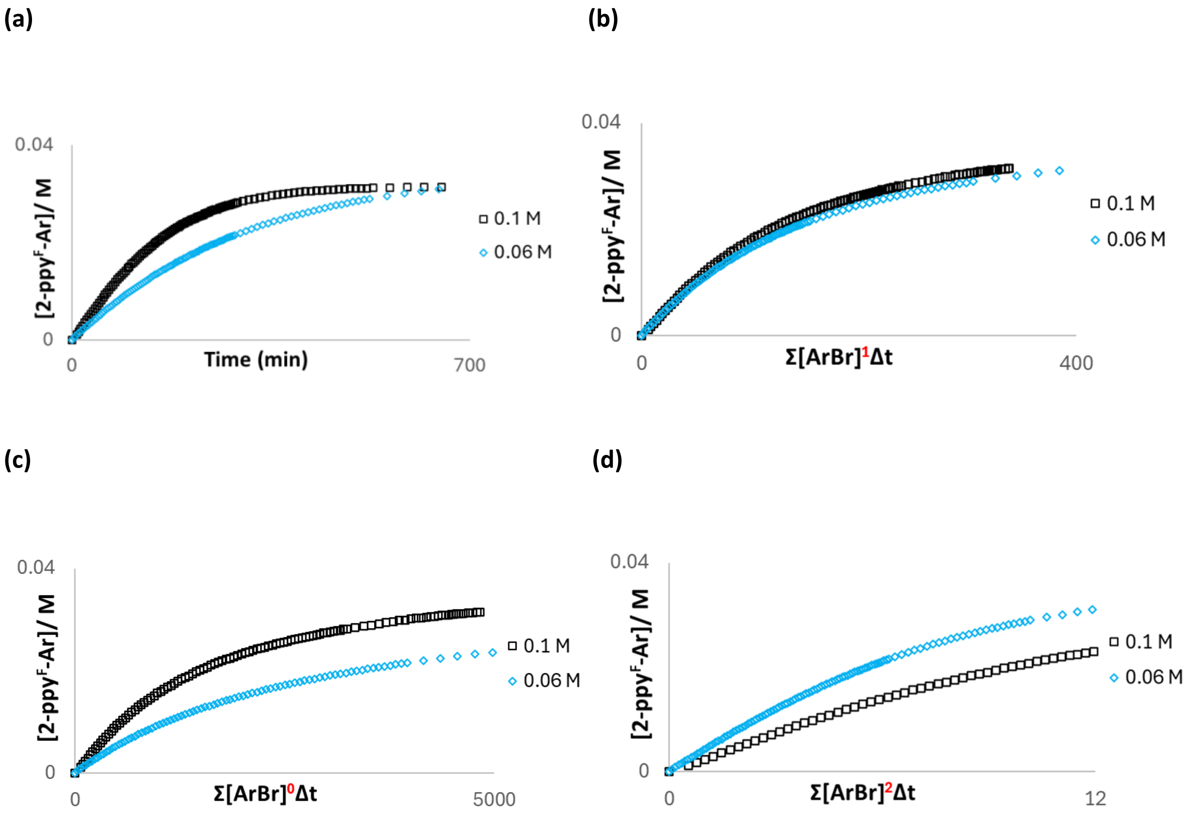

Figure S2. Determination of order in **3a**.

(a) Temporal reaction profiles of reactions carried out with 0.05/0.1 mmol of **ArBr**; (b) Normalized time scale profiles for order 1.0 in [**ArBr**]; (c) Normalized time scale profiles for order 0 in [**ArBr**]; (d) Normalized time scale profiles for order 2.0 in [**ArBr**].

The overlap between normalised time scale reaction profiles for these two reactions with differing concentrations of **ArBr** shows an order of 1.

5.3. Determination of the order in pivalonitrile

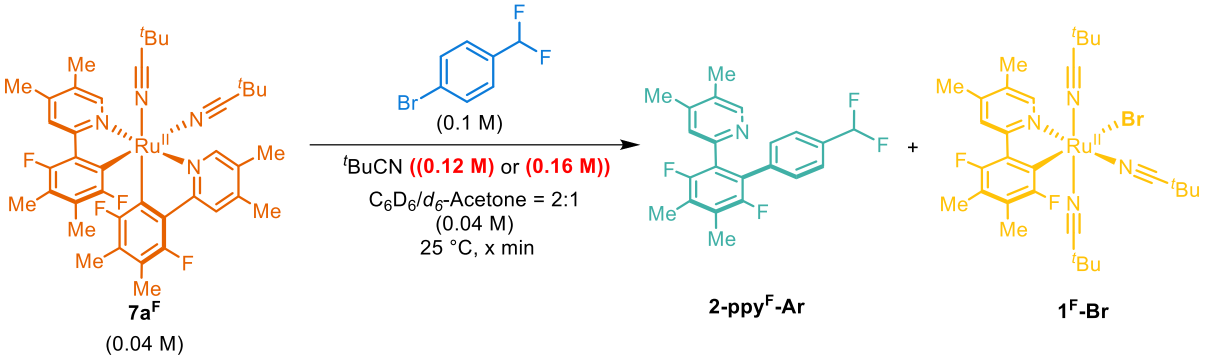

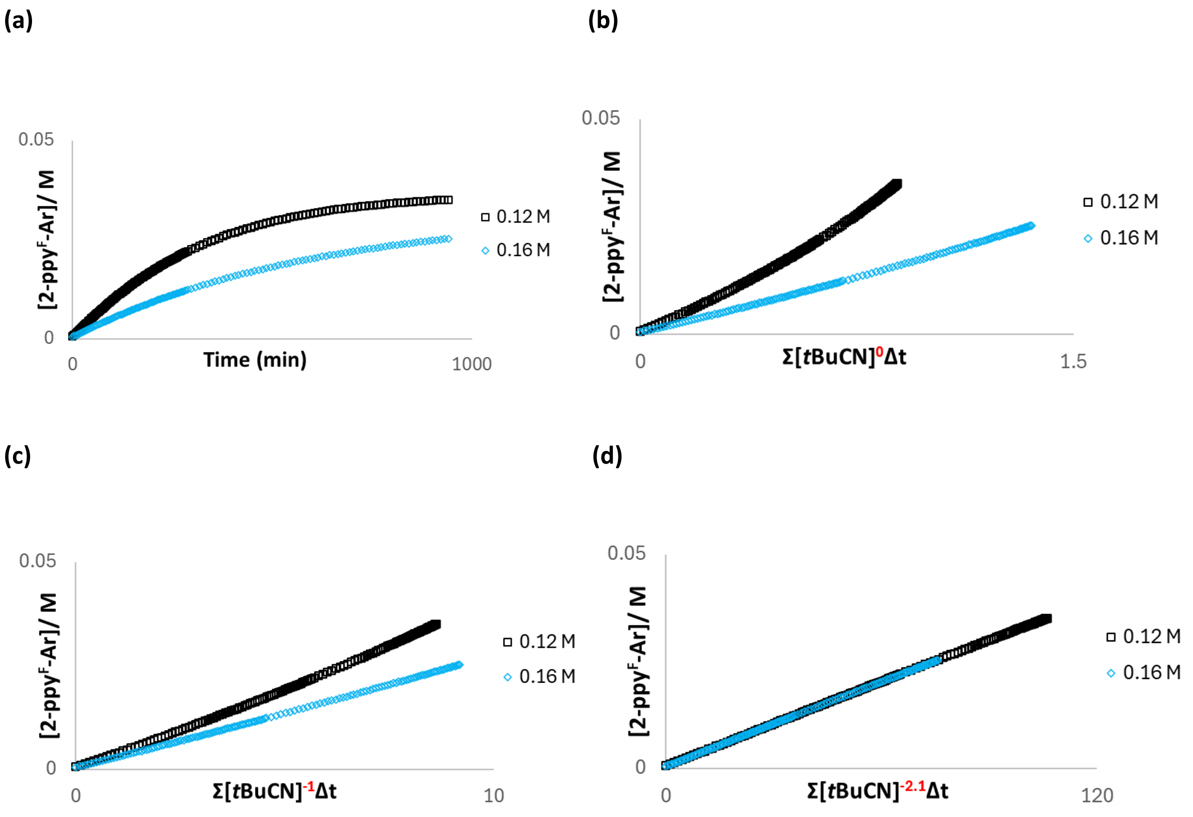

Figure S3. Determination of order in **pivalonitrile**.

(a) Temporal reaction profiles of reactions carried out with 0.05/0.1 mmol of **^{t}BuCN**; (b) Normalized time scale profiles for order 0 in [**^{t}BuCN**]; (c) Normalized time scale profiles for order -1.0 in [**^{t}BuCN**]; (d) Normalized time scale profiles for order -2.1 in [**^{t}BuCN**].

The overlap between normalised time scale reaction profiles for these two reactions with differing concentrations of **^{t}BuCN** shows an order of -2.1.

6. Kinetic analysis from 1^F

6.1. Reaction in presence of 2-ppy^F-H, KOAc and K_2CO_3 and then, addition of 4-bromoanisole

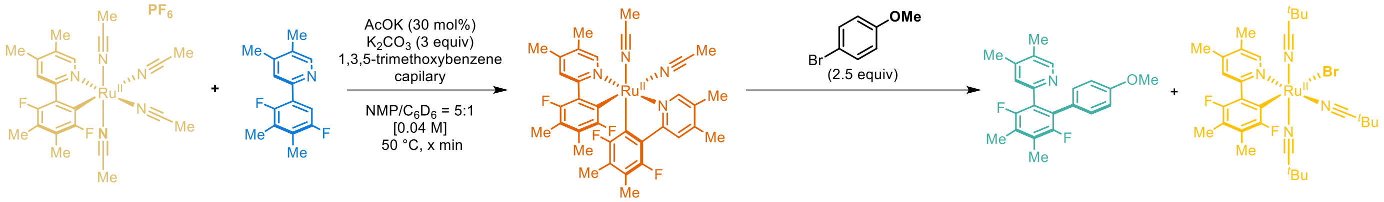

Inside an argon-filled glovebox, a solution of mono-cyclometalated complex **1^F^** (17.0 mg, 0.025 mmol), **2-ppy^F^-H** (6.2 mg, 0.025 mmol) KOAc (0.8 mg, 0.0075 mmol) and K_2CO_3 (10.0 mg, 0.075 mmol) was prepared in a mixture 5:1 of NMP/*d*$_6$-Benzene (0.04 M) in a Young's tap NMR tube, with a capillary with a solution of 1,3,5-trifluorobenzene in *d*$_6$-Benzene as internal standard. The reaction mixture was heated at 50 °C in an ultrasound bath, to facilitate the mixture of all reaction components, and NMR spectra were recorded after 0, 10, 20, 30, 40, 50, 60, 90 and 120 minutes inside the heating bath, until consumption of complex **1^F^**. Following this, 100 µL of a stock solution of 4-bromoanisole (2.50 equiv, 0.0625 mmol) was added to the tube (at room temperature, inside an argon-filled glove box), and the reaction mixture was then heated at 50 °C in an ultrasound bath. NMR spectra were recorded after 0, 5, 10, 15, 35 and 55 minutes inside the heating bath.

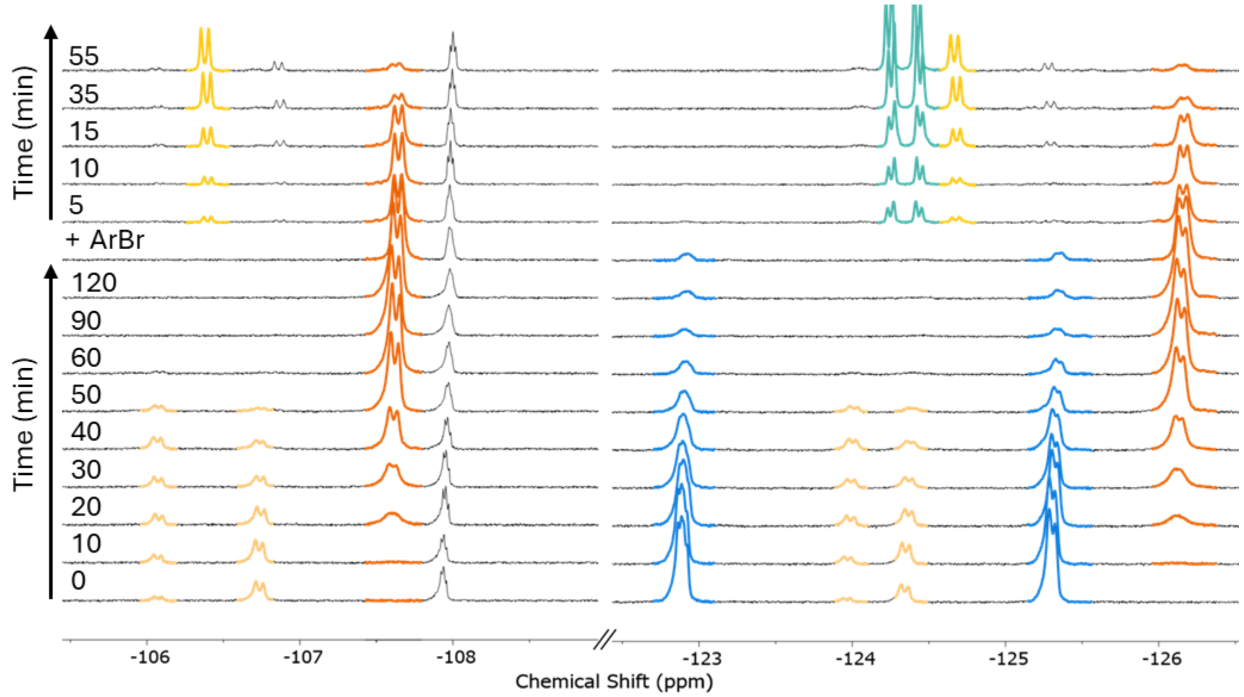

Figure S4. ^{19}F-NMR stacked spectra for reaction in presence of 2-ppy^F-H, KOAc and K_2CO_3 and then, addition of 4-bromoanisole.

6.2. Reaction in presence of 2-ppy^F-H in absence of KOAc and K_2CO_3, and then, addition of 4-bromoanisole

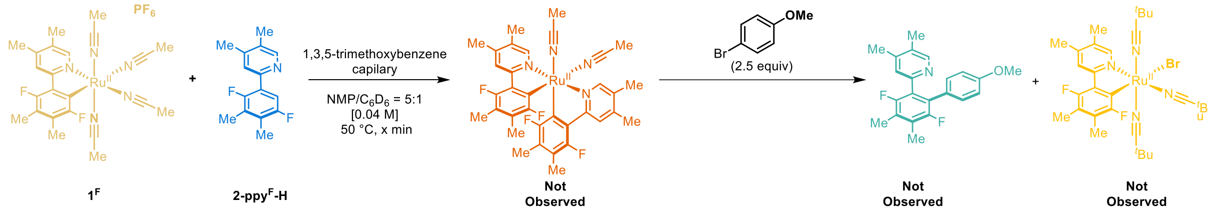

Inside an argon-filled glovebox, a solution of mono-cyclometalated complex **1^F** (17.0 mg, 0.025 mmol) and **2-ppy^F-H** (6.2 mg, 0.025 mmol) was prepared in a mixture 5:1 of NMP/*d$_6$*-Benzene (0.04 M) in a Young's tap NMR tube, with a capillary with a solution of 1,3,5-trifluorobenzene in *d$_6$*-Benzene as internal standard. The reaction mixture was heated at 50 °C in an ultrasound bath, to facilitate the mixture of all reaction components, and NMR spectra were recorded after 0, 10, 20, 30, 40, 50, 60, 90 and 120 minutes inside the heating bath. Then, 100 µL of a stock solution of 4-bromoanisole (2.50 equiv, 0.0625 mmol) was added to the tube (at room temperature, inside the glovebox), and the reaction mixture was then heated at 50 °C in an ultrasound bath. NMR spectra were recorded after 0, 10, 20, 30, 40, 50, 60 and 90 minutes inside the heating bath. Nor Bis-cyclometalated species **7a^F**, neither product **2-ppy^F-Ar** were observed during the analysis.

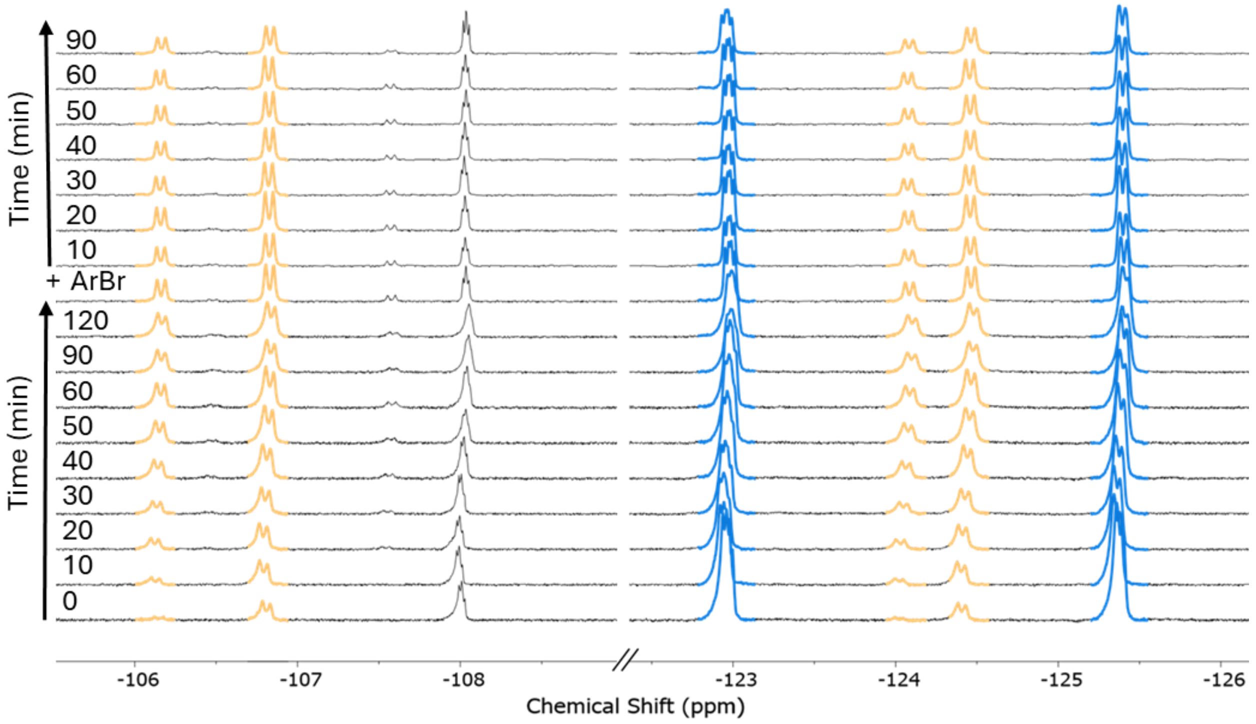

Figure S5. ^{19}F-NMR stacked spectra for reaction in presence of 2-ppy^F-H in absence of KOAc and K_2CO_3, and then, addition of 4-bromoanisole.

6.3. Reaction in presence of 2-ppy^F-H, KOAc, K_2CO_3 and 4-bromoanisole

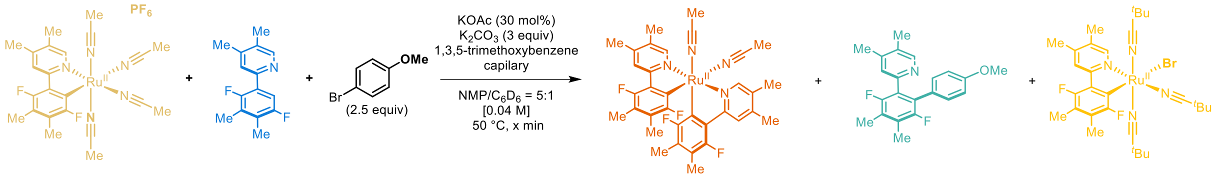

Inside an argon-filled glovebox, a solution of mono-cyclometalated complex **1^F** (17.0 mg, 0.025 mmol), **2-ppy^F-H** (6.2 mg, 0.025 mmol) KOAc (0.8 mg, 0.0075 mmol), K_2CO_3 (10.0 mg, 0.075 mmol) and 4-Bromoanisole (100 µL of stock solution 0.63 M in NMP, 0.0625 mmol) was prepared in a mixture 5:1 of NMP/d_6-Benzene (0.04 M) in a Young's tap NMR tube, with a capillary with a solution of 1,3,5-trifluorobenzene in d_6-Benzene as internal standard. The reaction mixture was heated at 50 °C in an ultrasound bath, to facilitate the mixture of all reaction components, and NMR spectra were recorded after 0, 10, 20, 30, 40, 50, 60, 90 and 120 minutes inside the heating bath.

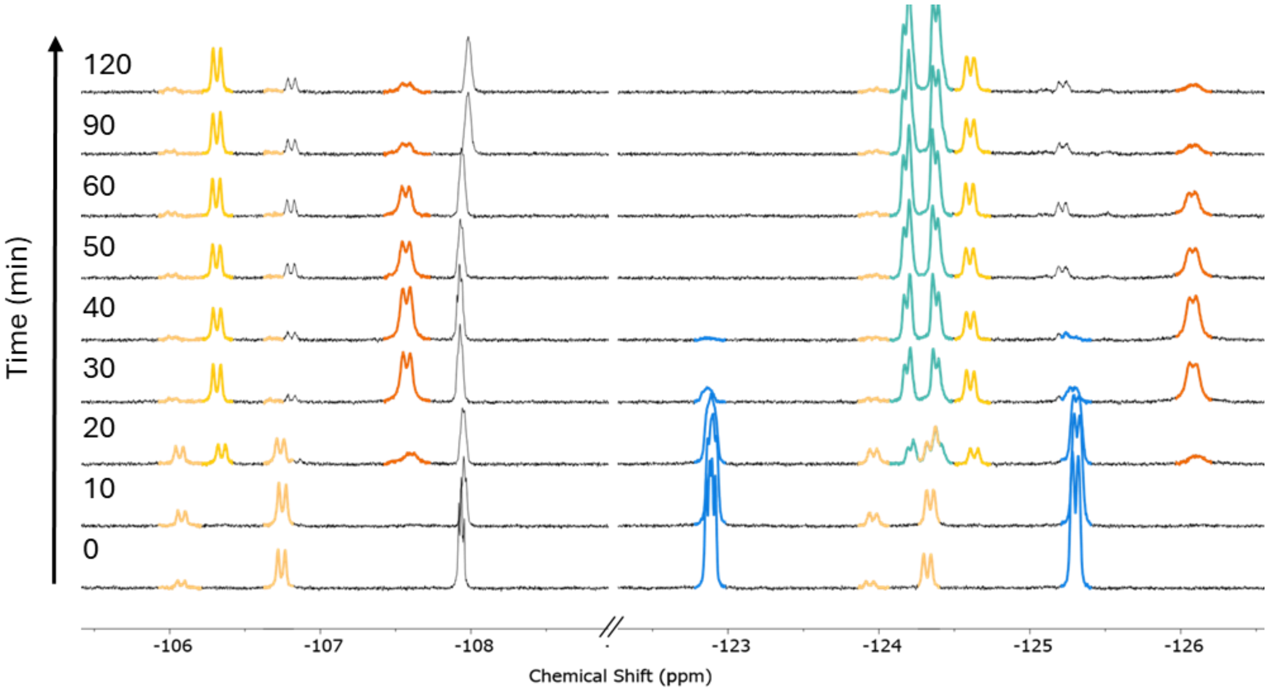

Figure S6. ^{19}F-NMR stacked spectra for reaction in presence of 2-ppy^F-H, KOAc, K_2CO_3 and 4-bromoanisole.

6.4. Reaction between complex 1^F^ and 4-bromoanisole, and then addition of 2-ppy^F^-H

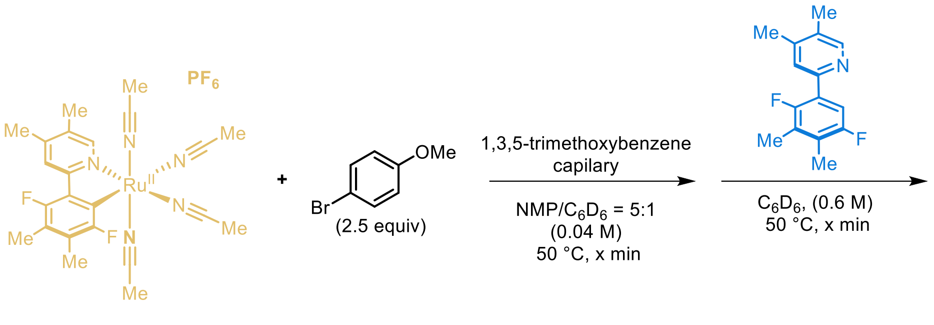

Inside an argon-filled glovebox, a solution of mono-cyclometalated complex **1^F^** (17.0 mg, 0.025 mmol) and 4-Bromoanisole (100 µL of stock solution 0.63 M in NMP, 0.0625 mmol) was prepared in a mixture 5:1 of NMP/d_6-Benzene (0.04 M) in a Young's tap NMR tube, with a capillary with a solution of 1,3,5-trifluorobenzene in d_6-Benzene as internal standard. The reaction mixture was heated at 50 °C in an ultrasound bath, to facilitate the mixture of all reaction components, and NMR spectra were recorded after 0, 10, 20, 30, 60, 90 and 120 minutes inside the heating bath. Then, 100 µL of a stock solution of **2-ppy^F^-H** (1.00 equiv, 0.025 mmol) was added to the tube (at room temperature, inside the glove box), and the reaction mixture was then heated at 50 °C in an ultrasound bath. NMR spectrum was recorded after 60 minutes inside the heating bath. Nor bis-cyclometalated species **7a^F^**, neither product **2-ppy^F^-PMP** were observed during the analysis.

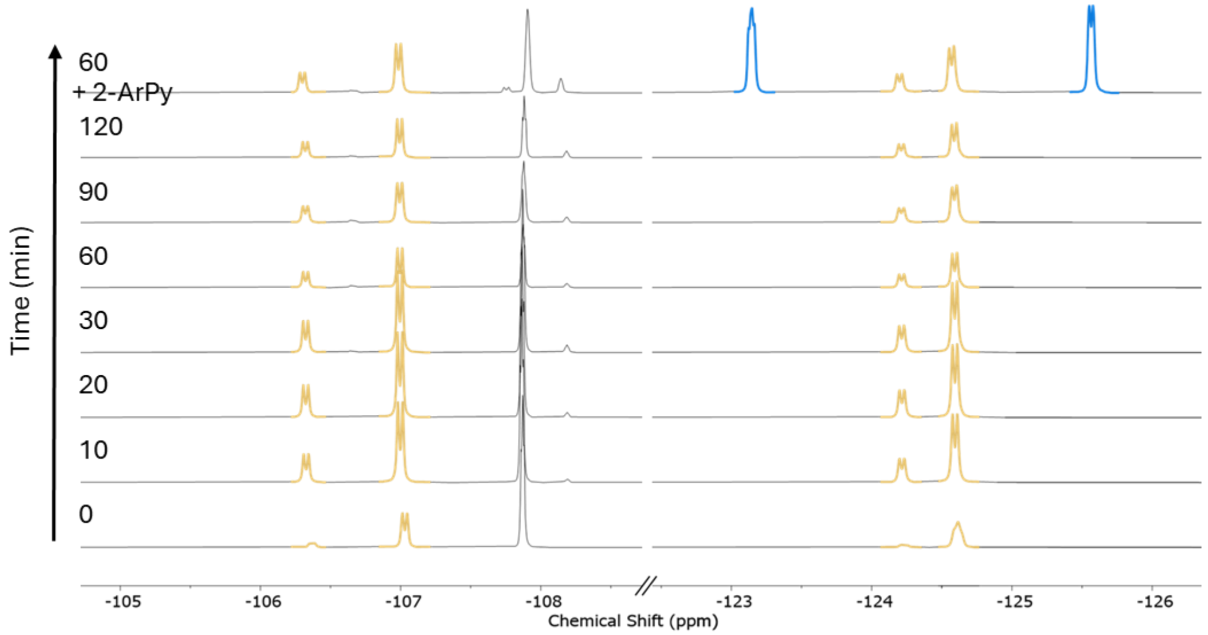

Figure S7. ^{19}F-NMR stacked spectra for reaction between complex 1^F^ and 4-bromoanisole, and then addition of 2-ppy^F^-H.

6.5. Reaction between complex 1^F and 4-bromoanisole in the presence of AcOK, and then addition of 2-ppy^F-H

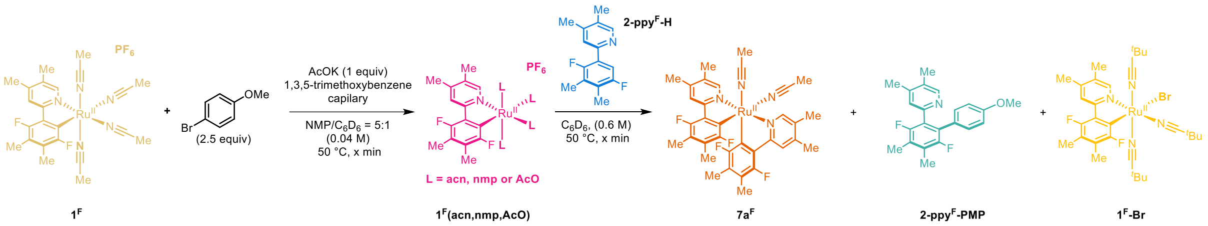

Inside an argon-filled glovebox, a solution of mono-cyclometalated complex $\mathbf{1^F}$ (17 mg, 0.025 mmol), AcOK (4 mg, 0.025 mmol) and 4-Bromoanisole (100 µL of stock solution 0.63 M in NMP, 0.0625 mmol) was prepared in a mixture 5:1 of NMP/d_6-Benzene (0.04 M) in a Young's tap NMR tube, with a capillary with a solution of 1,3,5-trifluorobenzene in d_6-Benzene as internal standard. The reaction mixture was heated at 50 °C in an ultrasound bath, to facilitate the mixture of all reaction components, and NMR spectra were recorded after 0, 10, 20, 30, 60, 90 and 120 minutes inside the heating bath. Then, 100 µL of a stock solution of **2-ppy^F-H** (1.0 equiv, 0.025 mmol) was added to the tube (at room temperature, inside the glove box), and the reaction mixture was then heated at 50 °C in an ultrasound bath. NMR spectrum was recorded after 60 minutes inside the heating bath. Both bis-cyclometalated species $\mathbf{7a^F}$ and product **2-ppy^F-PMP** were observed during the analysis, along with ligand exchange of the acetonitrile ligands in $\mathbf{1^F}$ with NMP and acetate.

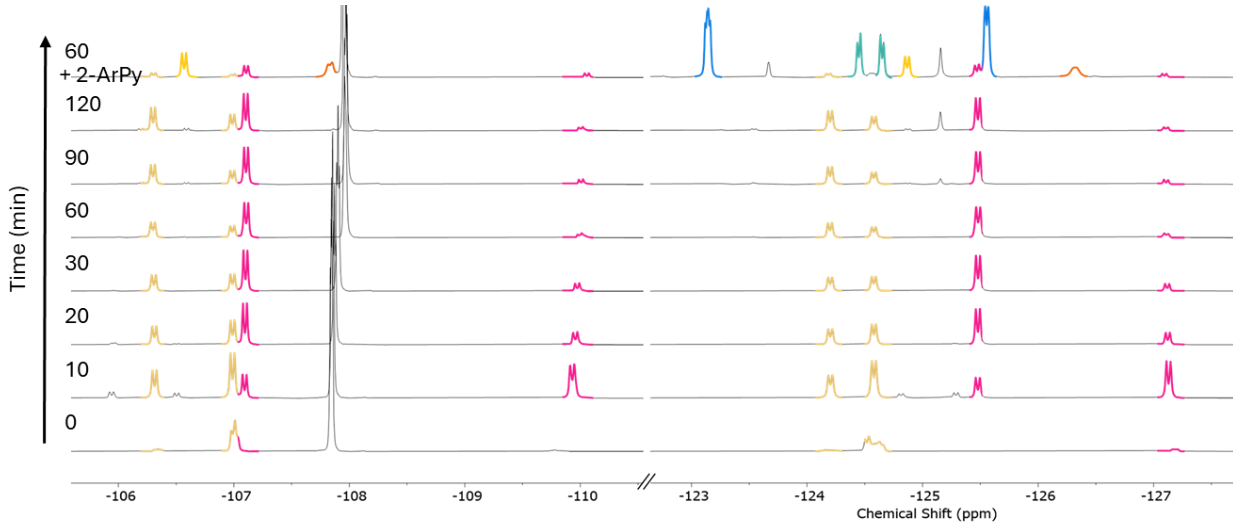

Figure S8. ^{19}F-NMR stacked spectra for reaction between complex 1^F and 4-bromoanisole in the presence of AcOK, and then addition of 2-ppy^F-H.

7. Scrambling Experiment

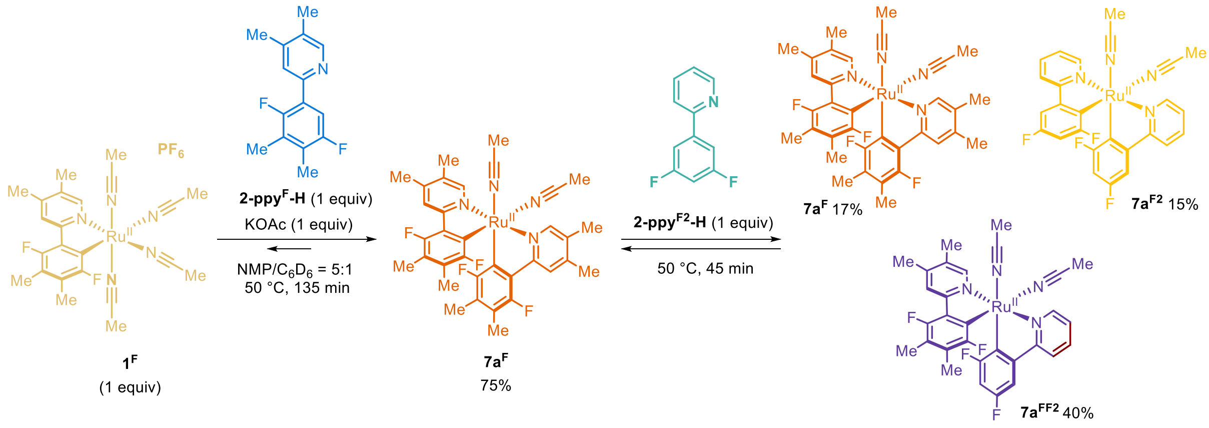

Inside an argon-filled glovebox, a solution of mono-cyclometalated complex **1^F** (17.0 mg, 0.025 mmol), **2-ppy^F-H** (6.2 mg, 0.025 mmol, 1.00 equiv), KOAc (0.8 mg, 0.025 mmol, 1.00 equiv) and trifluoromethoxybenzene (0.025 mmol, 1.00 equiv) was prepared in a mixture 5:1 of NMP/*d$_6$*-Benzene (0.04 M) in a Young's tap NMR tube. The reaction mixture was heated at 50 °C in an ultrasound bath, to facilitate the mixture of all reaction components, and NMR spectra were recorded after 0, 15, 45 and 75 minutes inside the heating bath. Following this, 100 μL of a stock solution of **2-ppy^{F2}-H** (1.0 equiv, 0.025 mmol) was added to the tube (at room temperature, inside an argon-filled glove box), and the reaction mixture was then heated at 50 °C in an ultrasound bath. NMR spectra were recorded after 15, 45, 75, and 105 minutes inside the heating bath. Trifluoromethoxybenzene was used as internal standard.

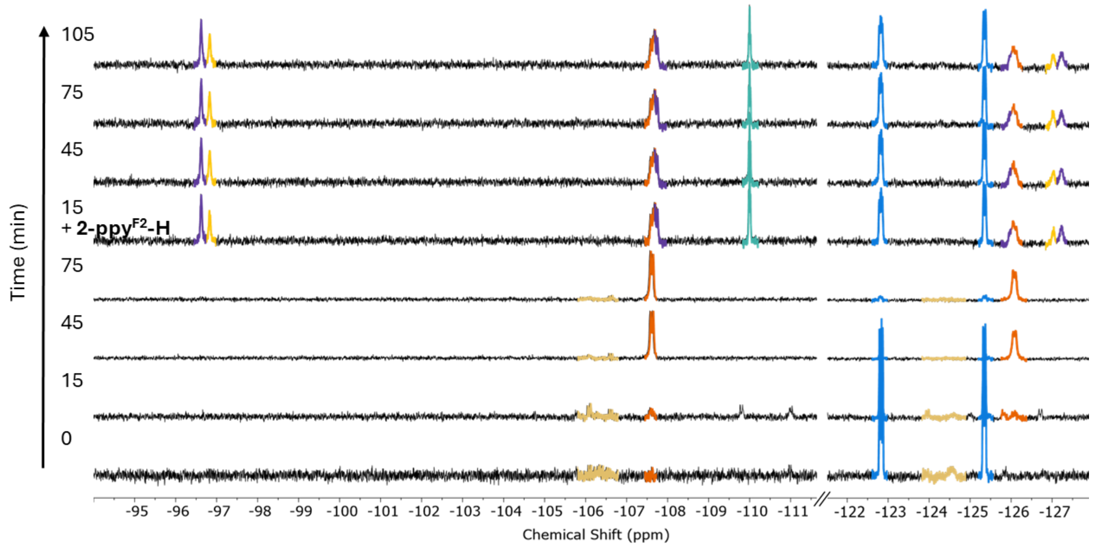

Figure S9. ^{19}F-NMR stacked spectra for scrambling experiment.

8. Effect of AcOK in the reaction kinetics

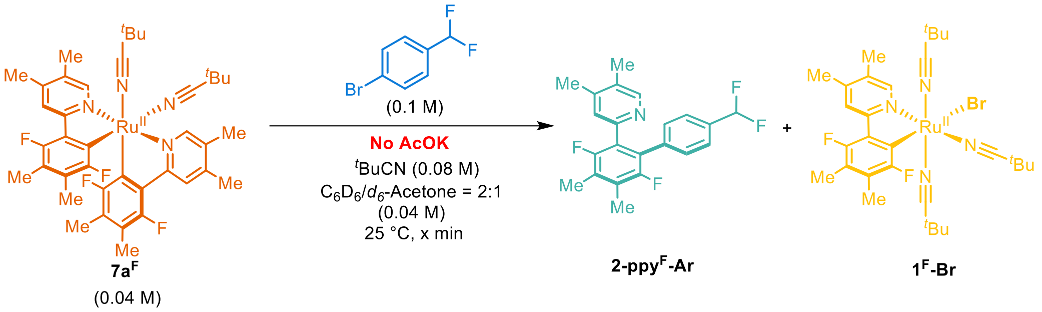

7a^F was weighed out in an argon-filled glove box into a Young's Tap NMR tube (19.0 mg, 0.025 mmol, 1.00 equiv). Then, 100 µL of a stock solution of pivalonitrile in d_6-benzene was added (0.05 mmol, 2.00 equiv), followed by the addition of 100 µL of a stock solution of trifluoromethoxybenzene (0.025 mmol, 1.00 equiv), 100 µL of d_6-benzene and 150 µL of d_6-acetone. Then, a NMR spectrum was recorded, and the tube was introduced again inside the glove box, when 50 µL of d_6-acetone and 100 µL of a stock solution of 4-(difluoromethyl)bromobenzene were added (0.0625 mmol, 2.5 equiv). The tube was frozen in a dry ice/acetone bath to prevent the reaction to occur before the NMR experiments take place. Then, the reaction was monitored by NMR, at 25 degrees, using trifluoromethoxybenzene as internal standard.

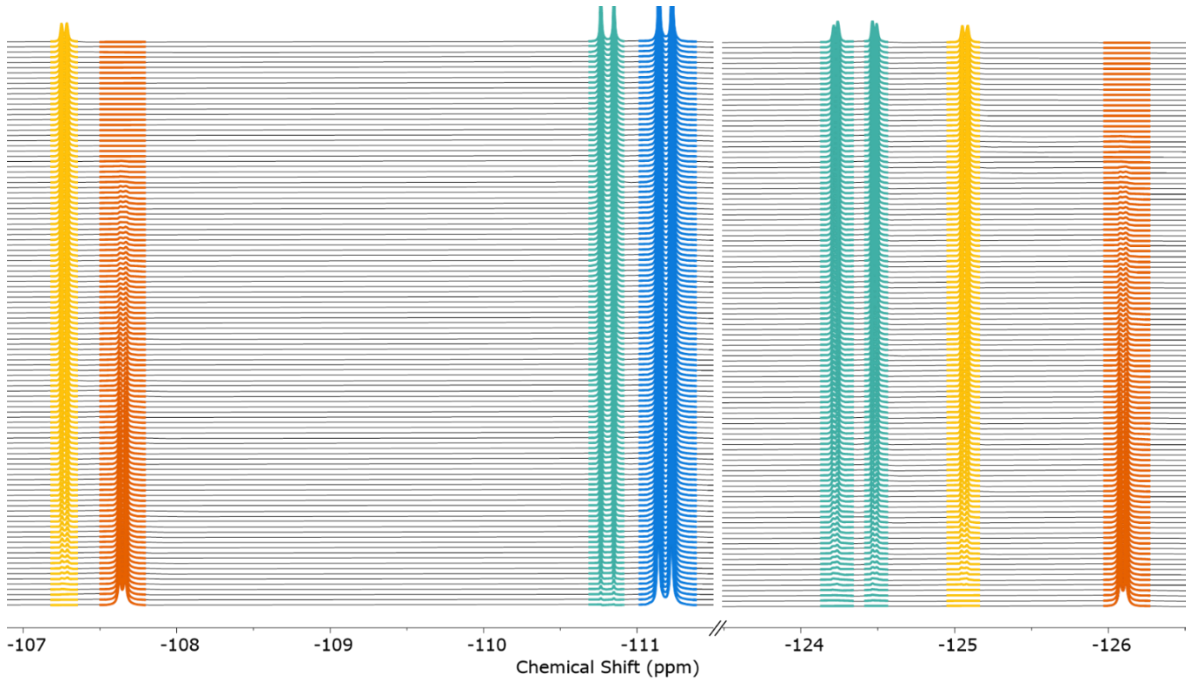

Figure S10. ^{19}F-NMR stacked spectra for reaction between **7a^F** and 2-bromoanisole in the absence of AcOK.

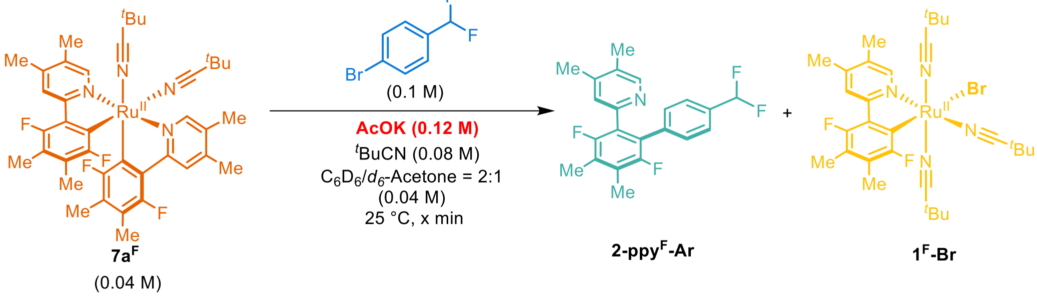

7a^{F} (19 mg, 0.025 mmol, 1.00 equiv) and AcOK (7.5 mg, 0.075 mmol, 3.00 equiv) were weighed out in an argon-filled glove box into a Young's Tap NMR tube. Then, 100 µL of a stock solution of pivalonitrile in d_6-benzene was added (0.05 mmol, 2.00 equiv), followed by the addition of 100 µL of a stock solution of trifluoromethoxybenzene (0.025 mmol, 1.00 equiv), 100 µL of d_6-benzene and 150 µL of d_6-acetone. Then, a NMR spectrum was recorded, and the tube was introduced again inside the glove box, when 50 µL of d_6-acetone and 100 µL of a stock solution of 4-(difluoromethyl)bromobenzene were added (0.0625 mmol, 2.50 equiv). The tube was frozen in a dry ice/acetone bath to prevent the reaction to occur before the NMR experiments take place. Then, the reaction was monitored by NMR, at 25 degrees, using trifluoromethoxybenzene as internal standard.

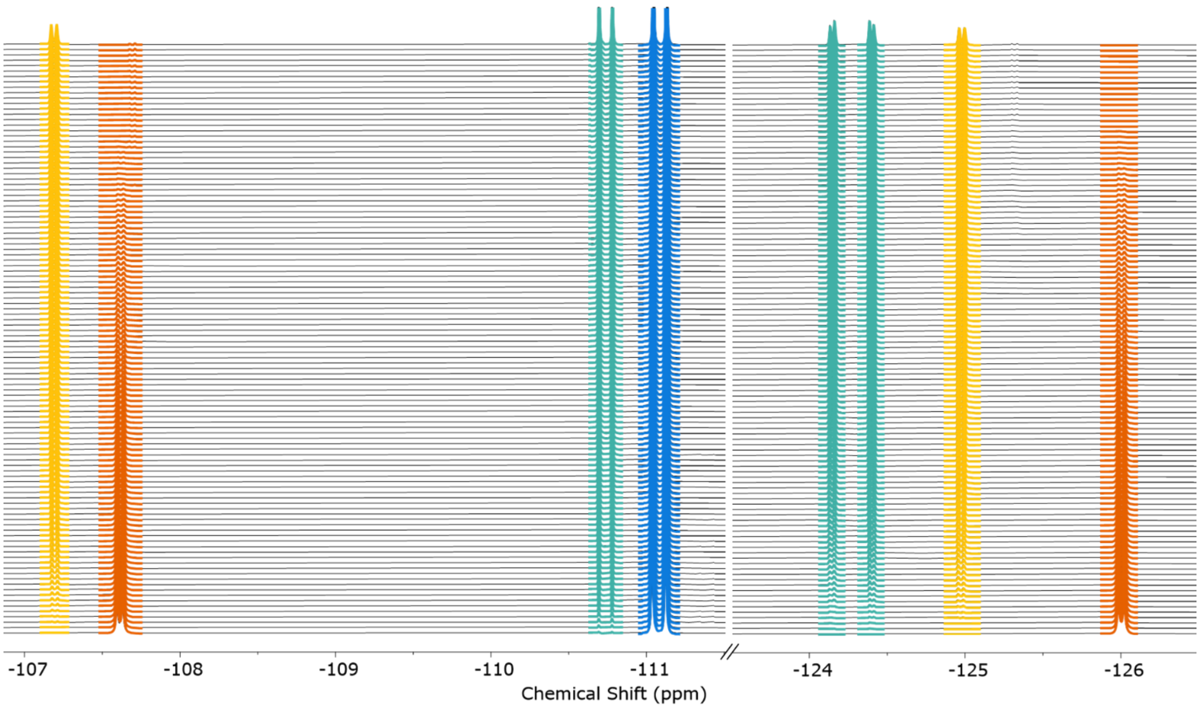

Figure S11. ^{19}F-NMR stacked spectra for reaction between **7a**^{F} and 2-bromoanisole in the presence of 3 equivalents of AcOK.

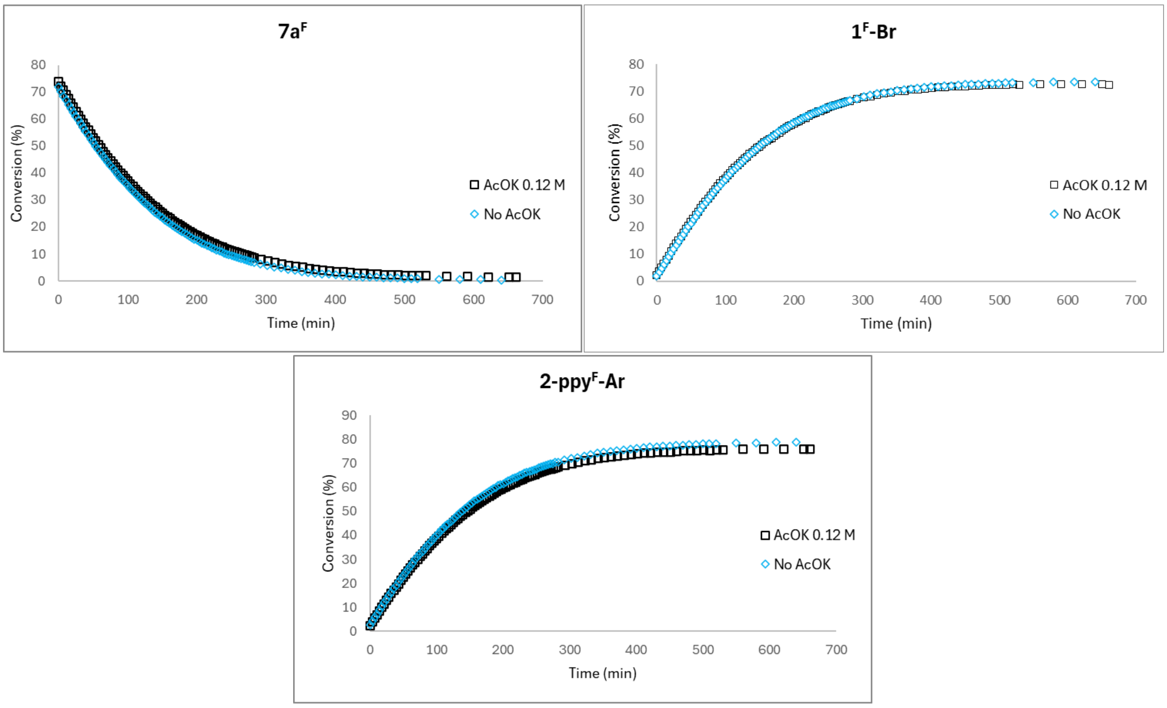

Figure S12. Comparison of reaction profiles between the reactions in the absence and presence of AcOK.

9. NMR Spectra of pure compounds

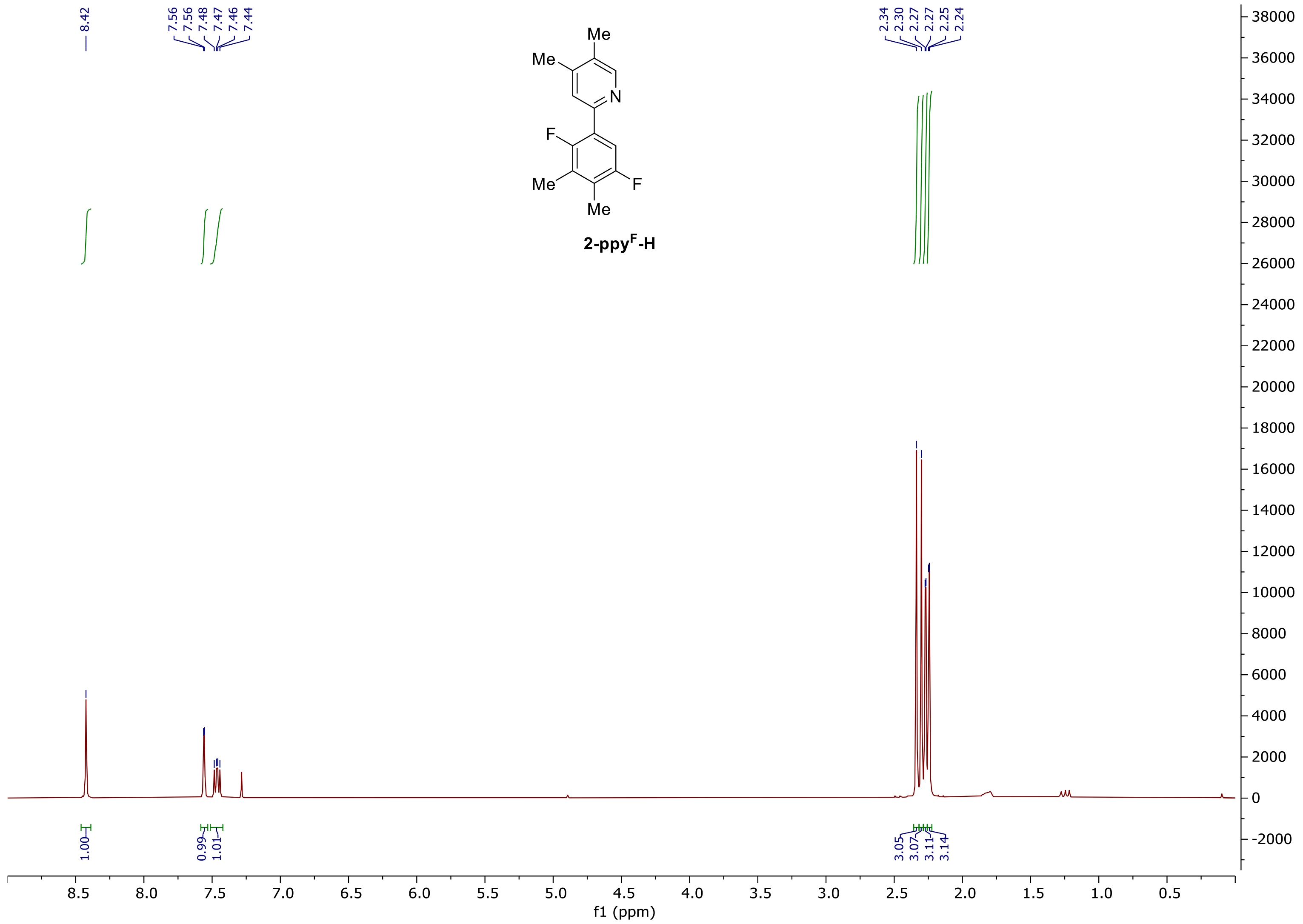

^{1}H-NMR spectrum (400 MHz, $CDCl_3$) of **2-ppy^F-H**

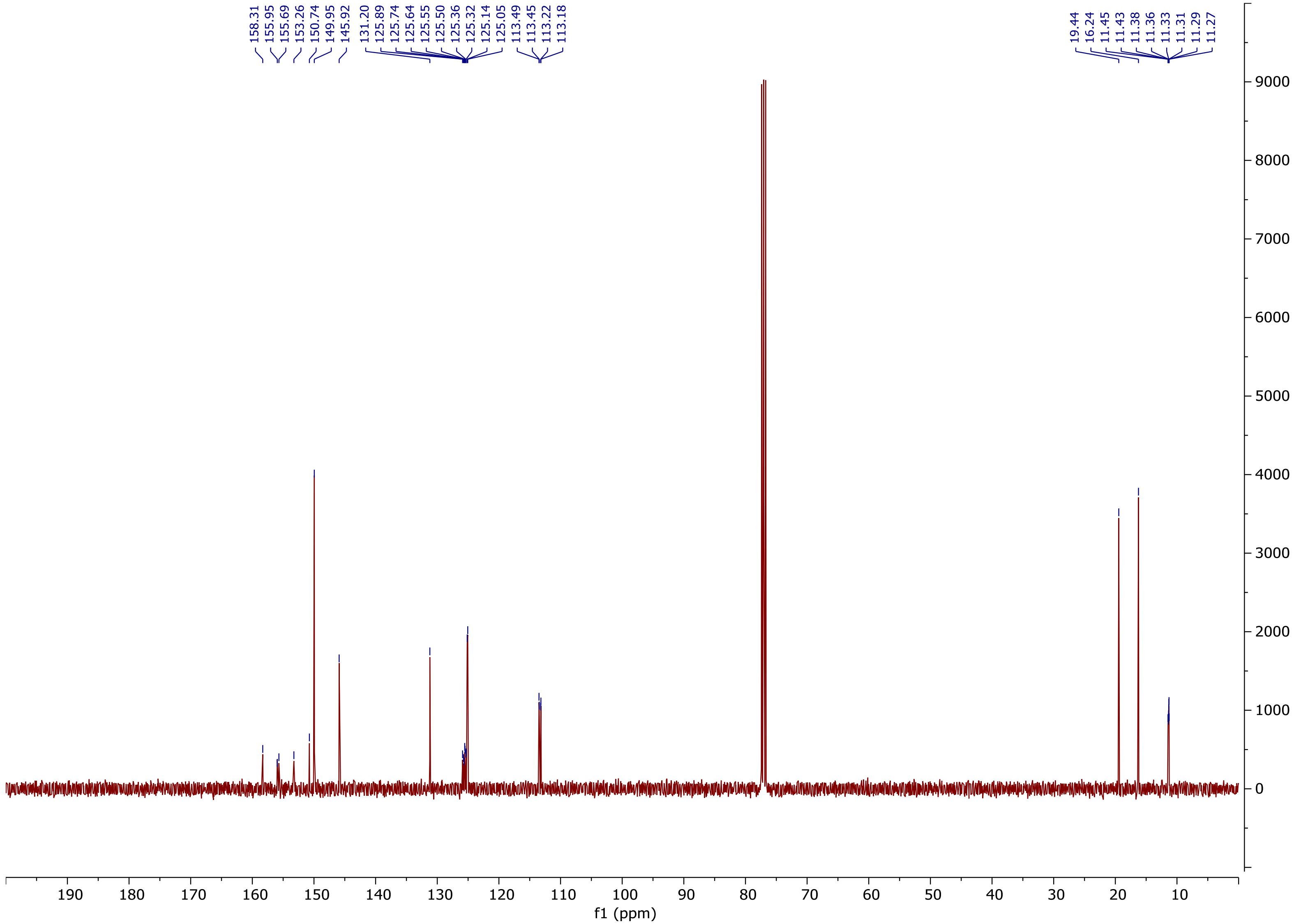

^{13}C-NMR spectrum (101 MHz, $CDCl_3$) of **2-ppy^{F}-H**

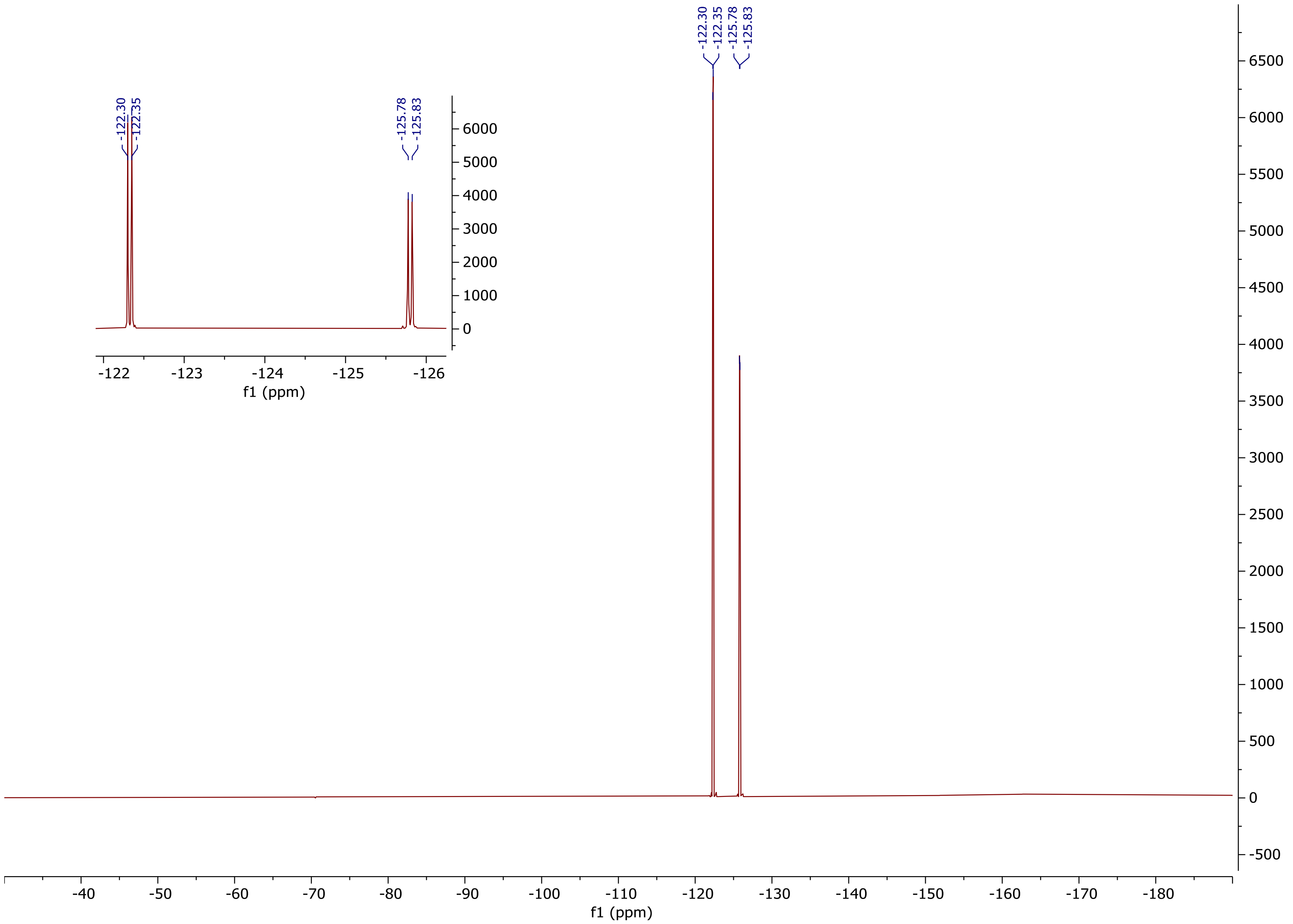

^{19}F-NMR spectrum (376 MHz, $CDCl_3$) of **2-ppy^{F}-H**

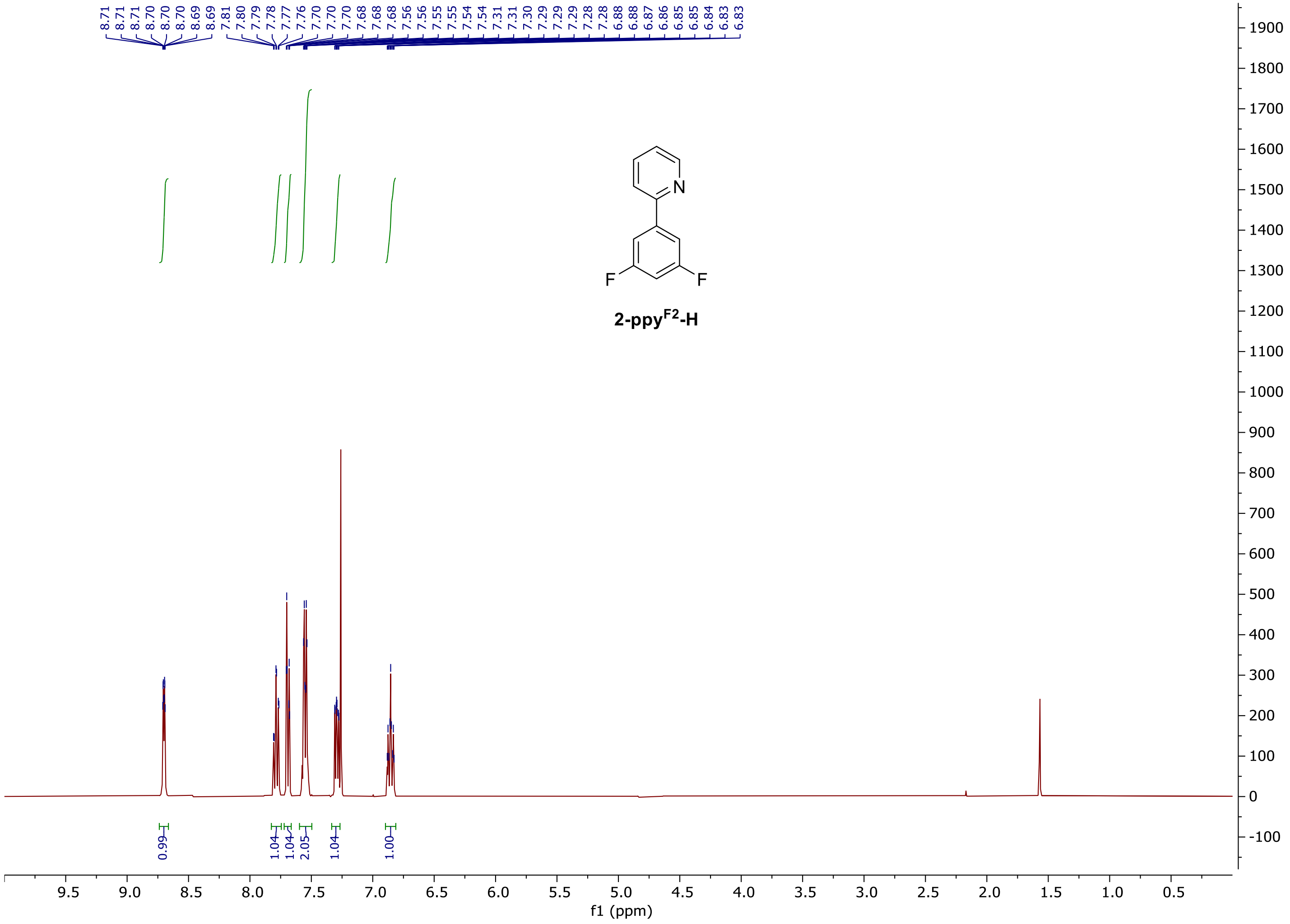

^{1}H-NMR spectrum (400 MHz, $CDCl_3$) of **2-ppy^{F2}-H**

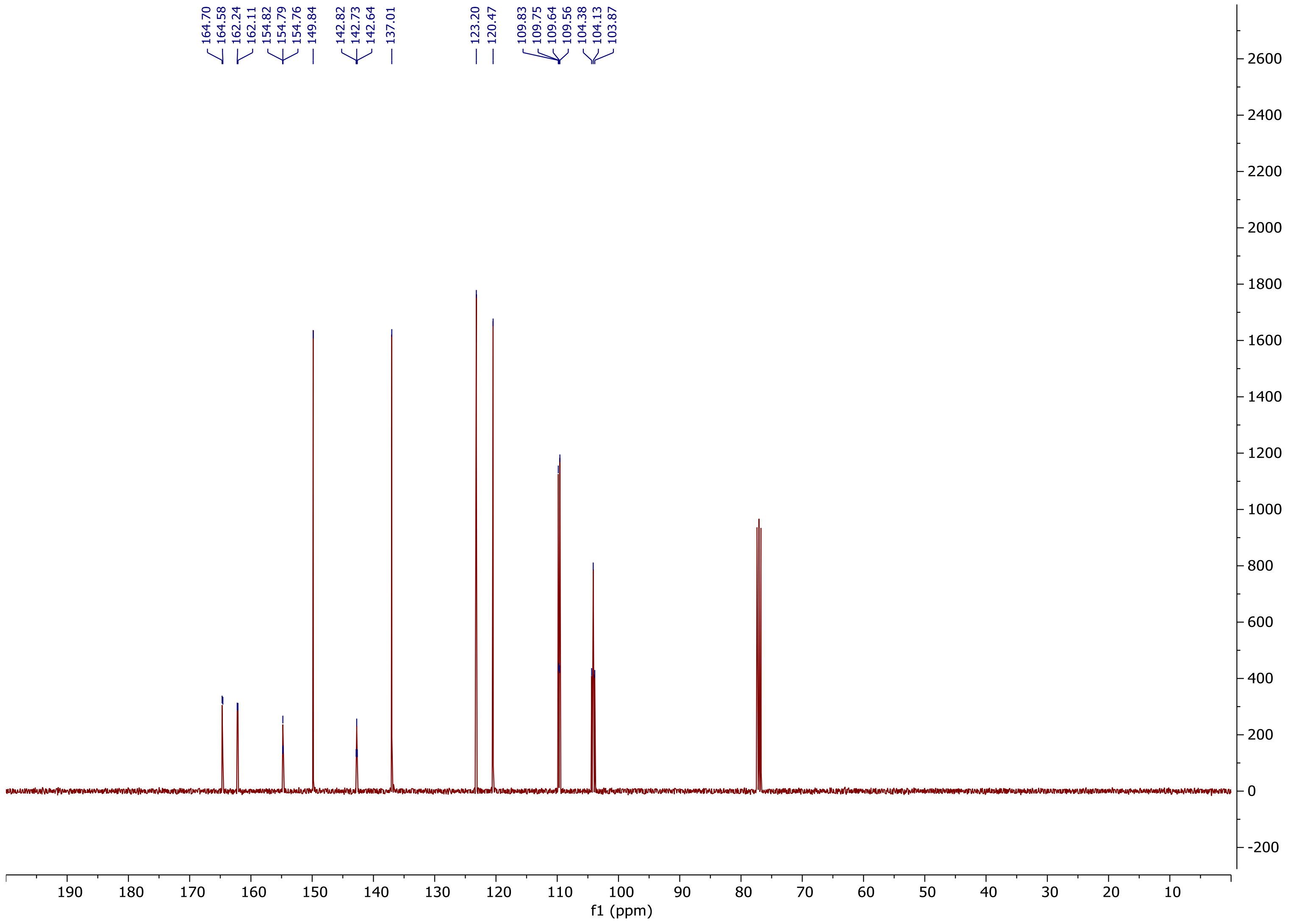

^{13}C-NMR spectrum (101 MHz, $CDCl_3$) of **2-ppy^{F2}-H**

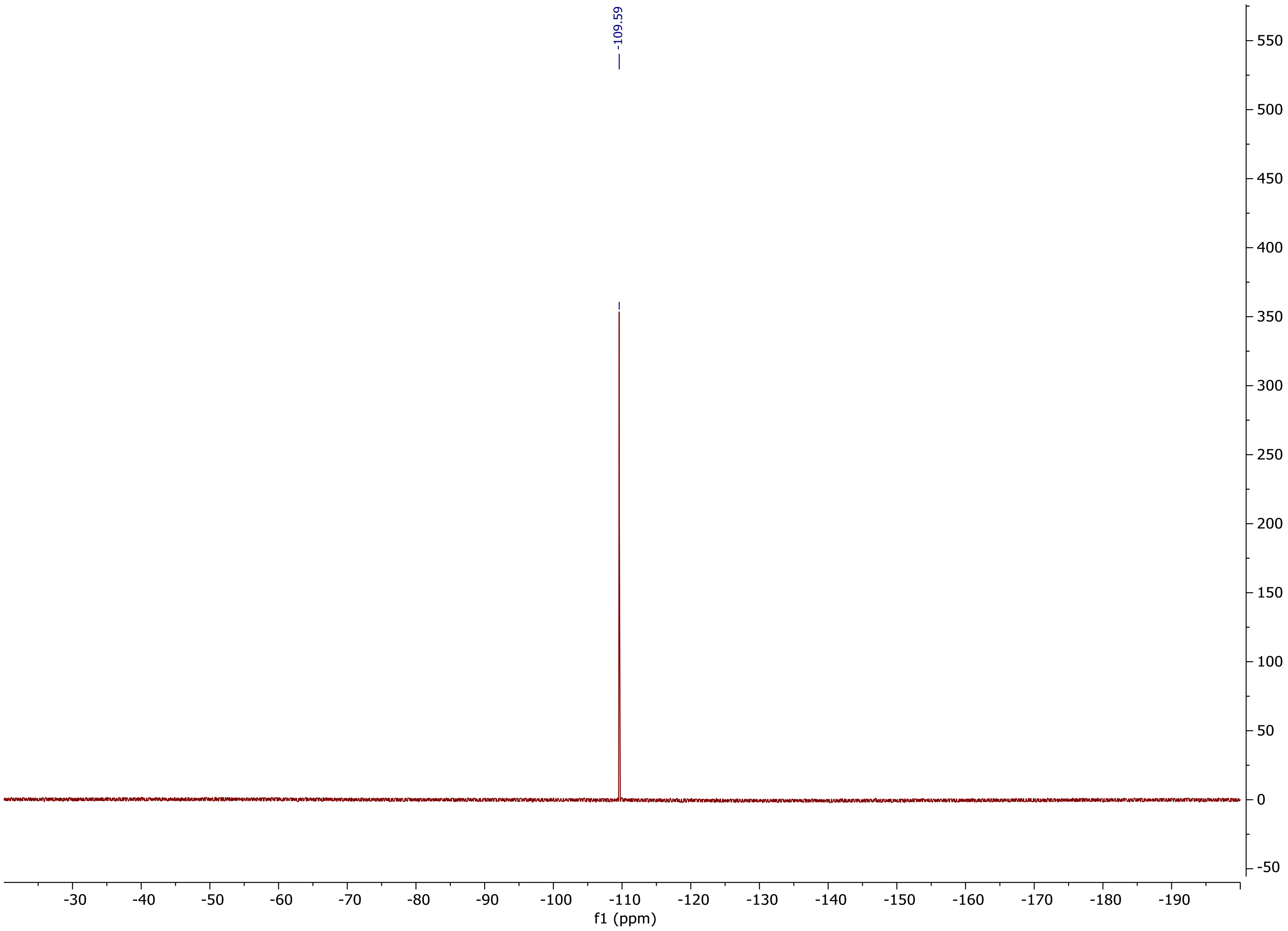

^{19}F-NMR spectrum (376 MHz, $CDCl_3$) of **2-ppy^{F2}-H**

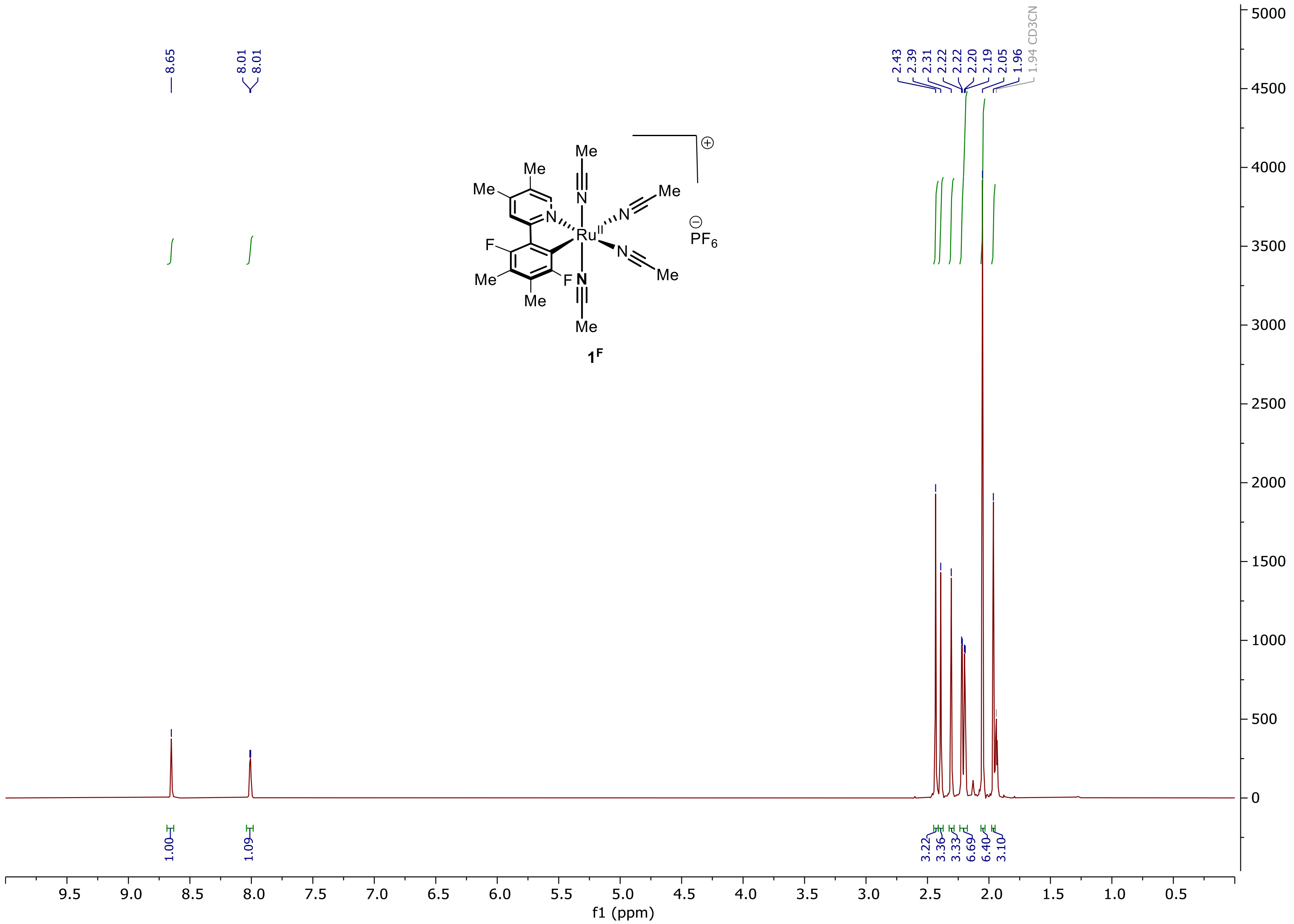

^{1}H-NMR spectrum (400 MHz, CD_3CN) of **1^{F}**

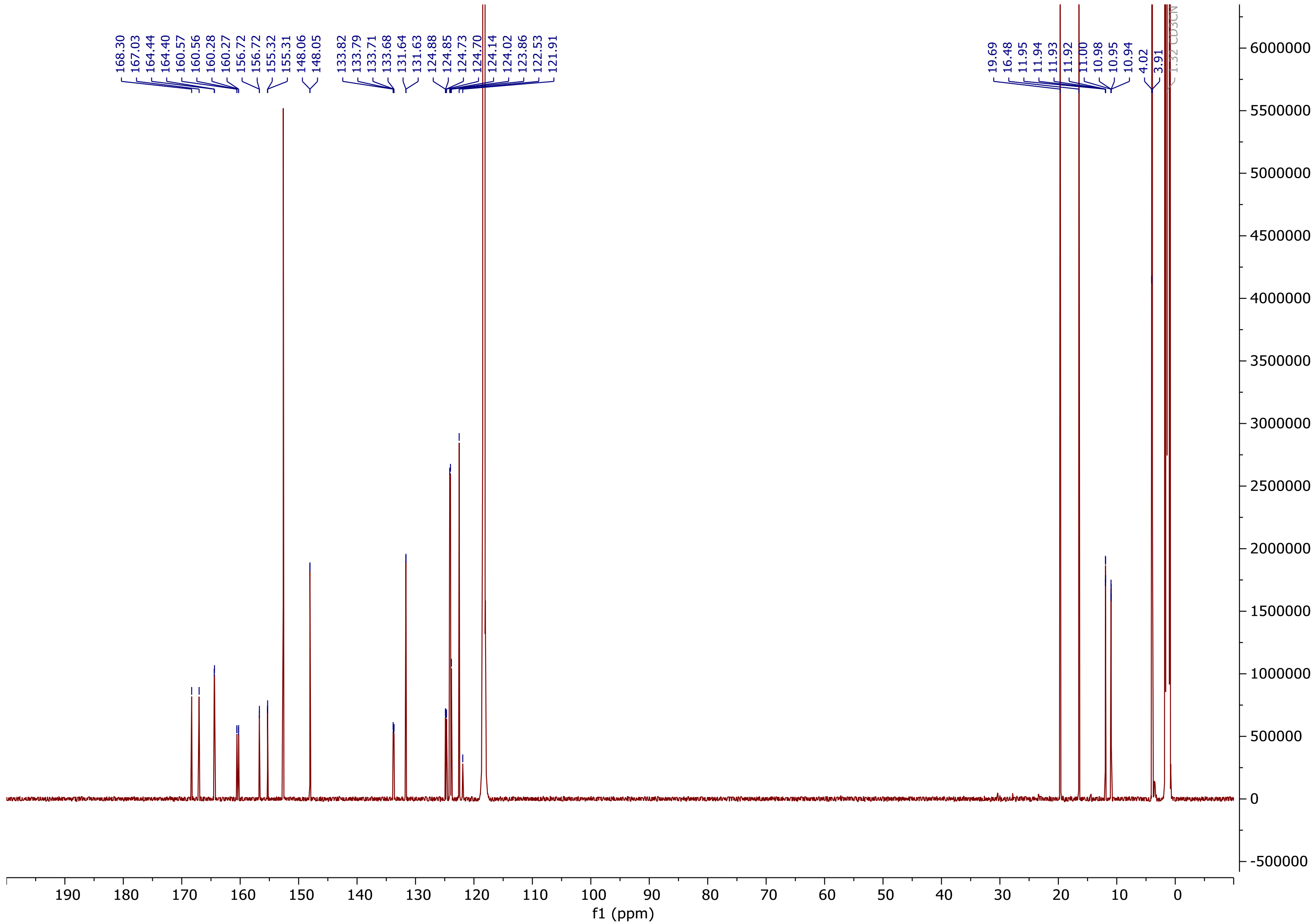

^{13}C-NMR spectrum (176MHz, CD_3CN) of $\mathbf{1^F}$

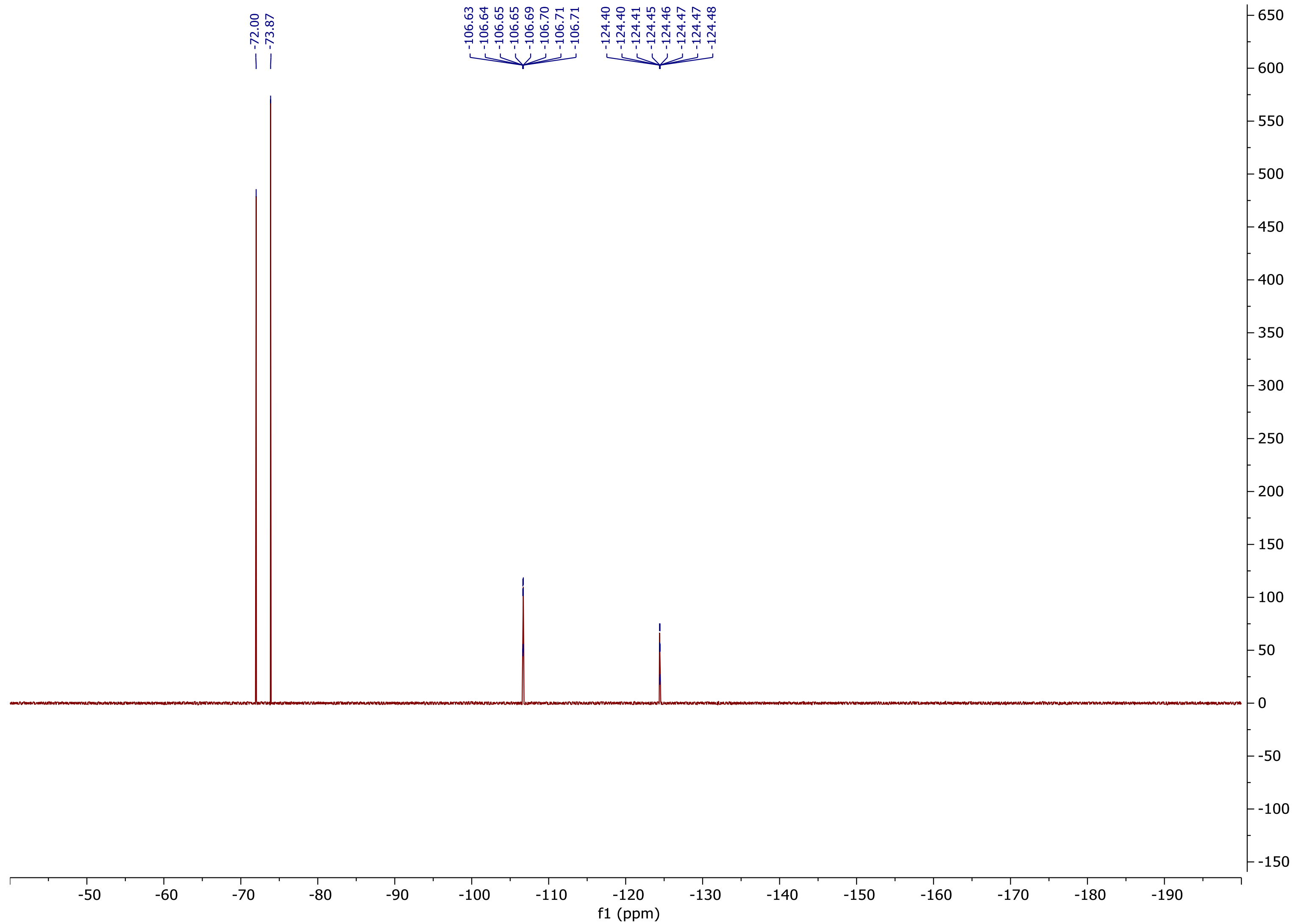

^{19}F-NMR spectrum (376 MHz, CD_3CN) of **1^{F}**

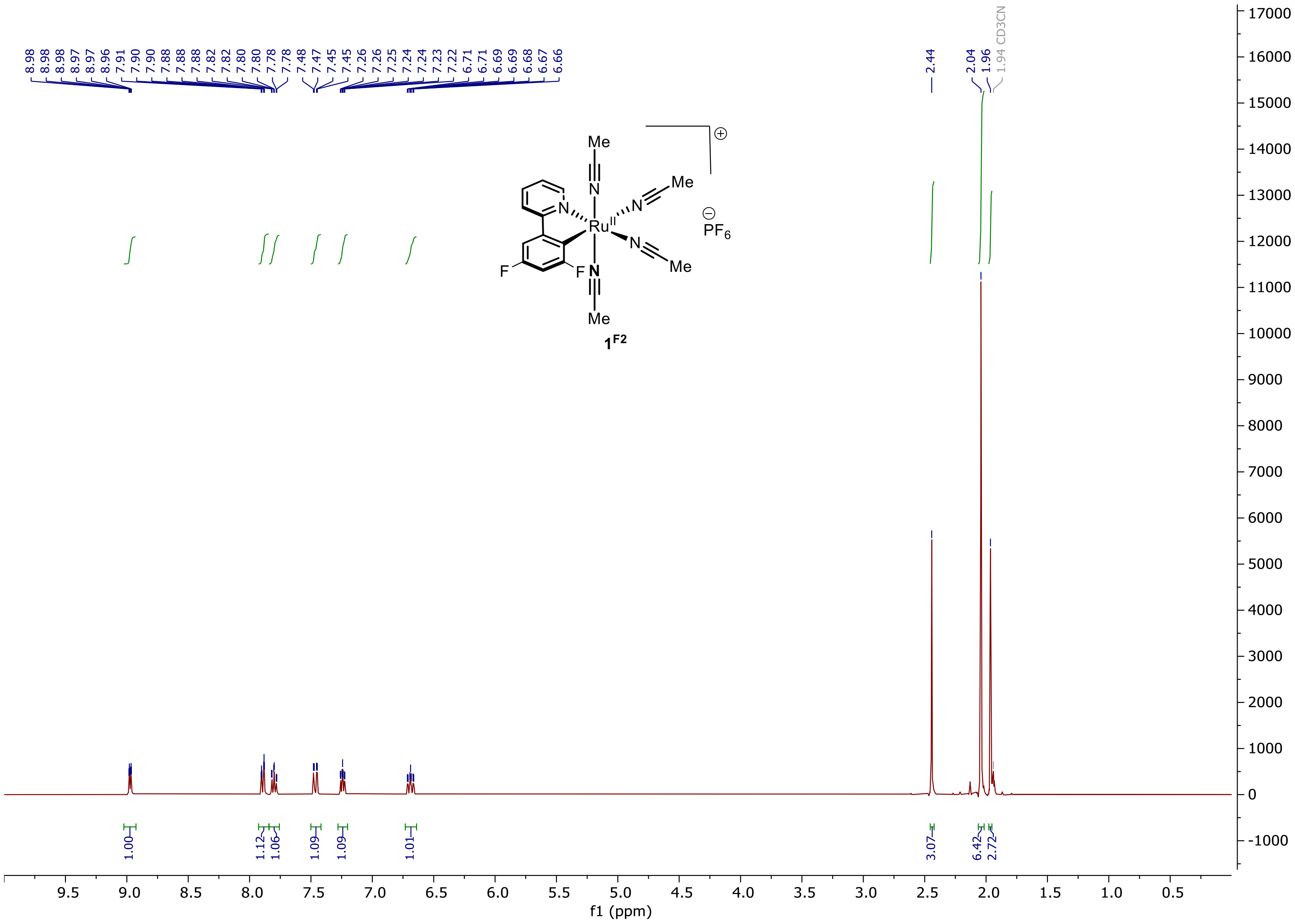

^{1}H-NMR spectrum of $\mathbf{1^{F2}}$

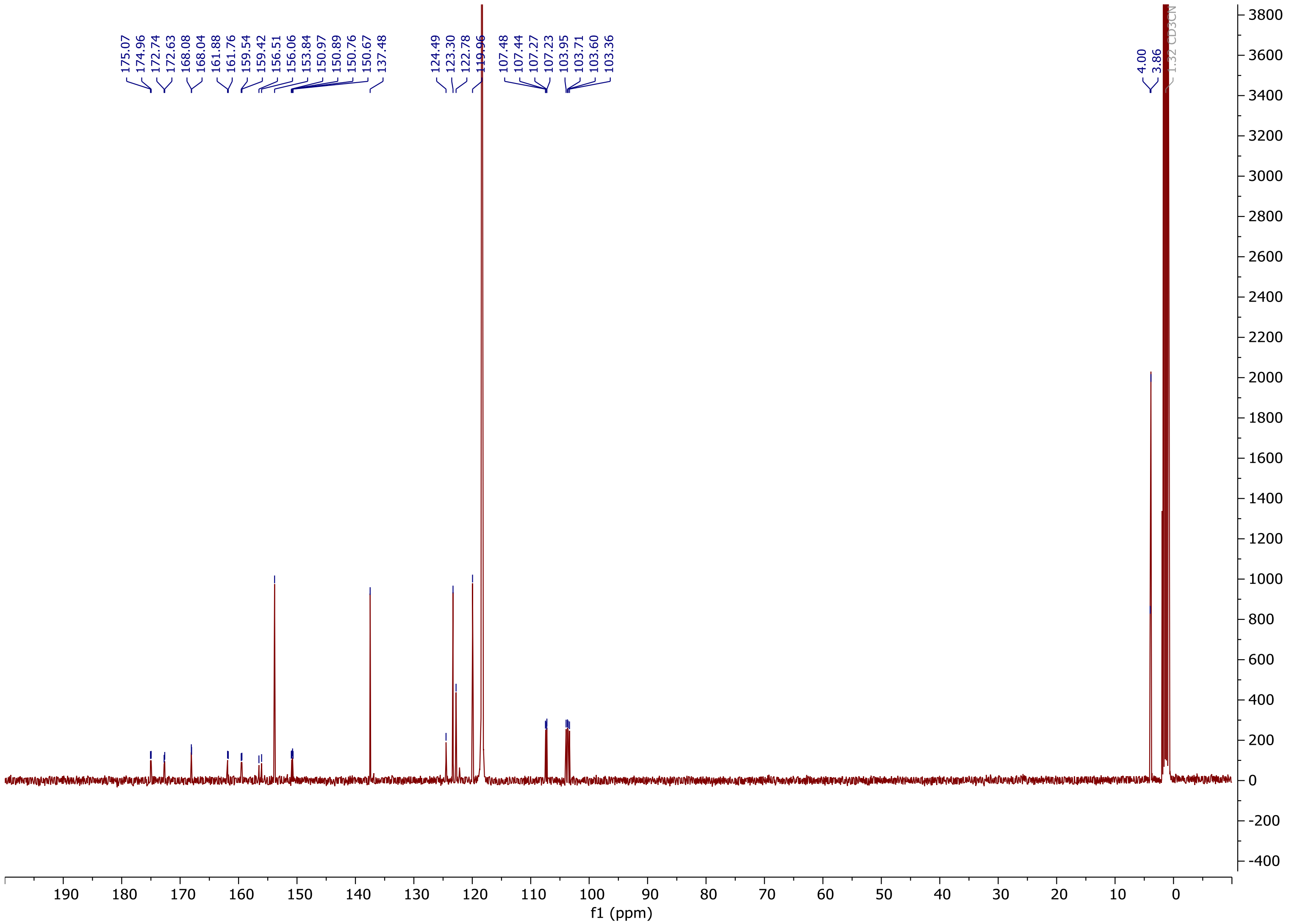

^{13}C-NMR spectrum of **1^{F2}**

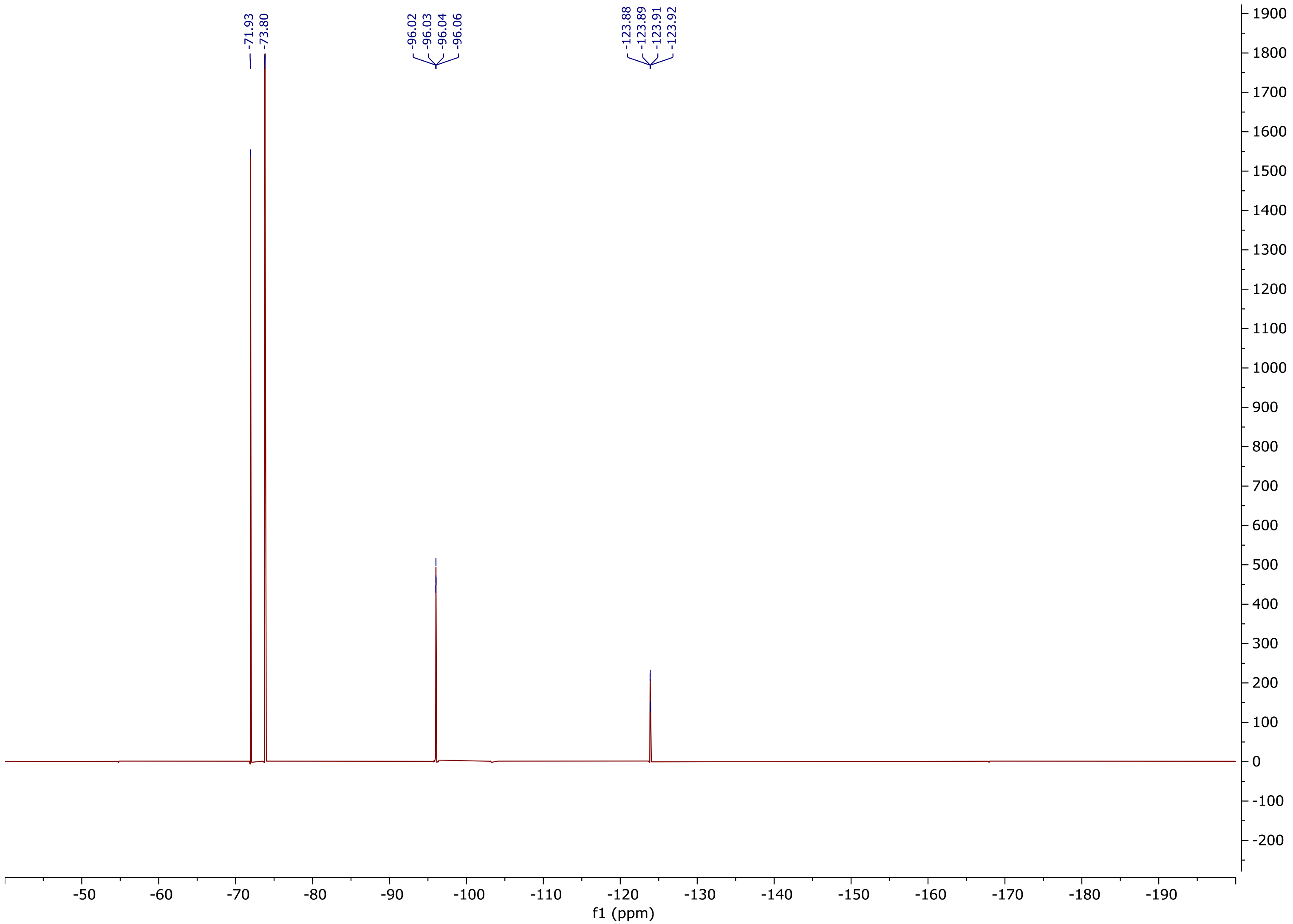

^{19}F-NMR spectrum of **1^{F2}**

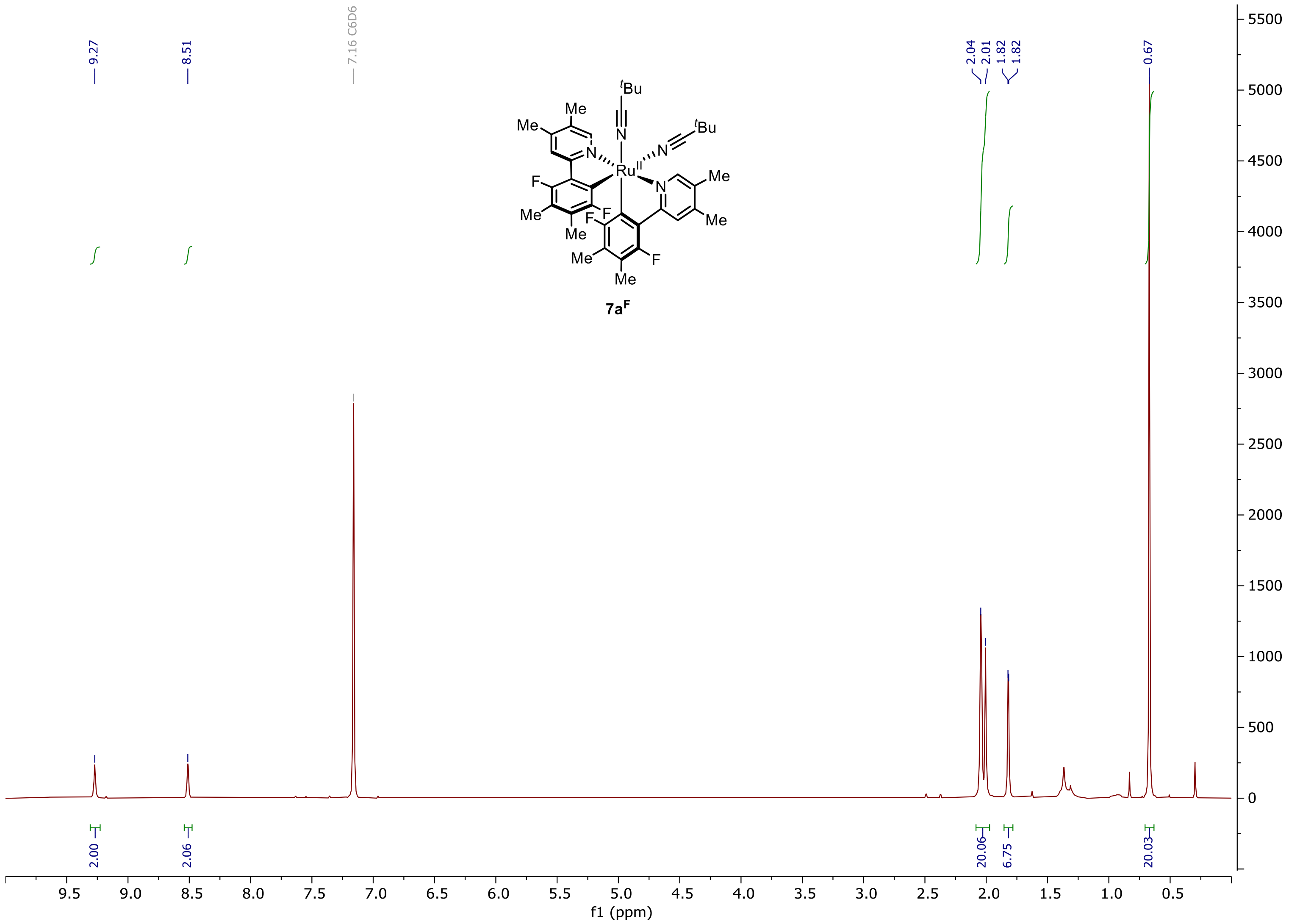

^{1}H-NMR spectrum (400 MHz, C_6D_6) of **7a**$^{\mathbf{F}}$

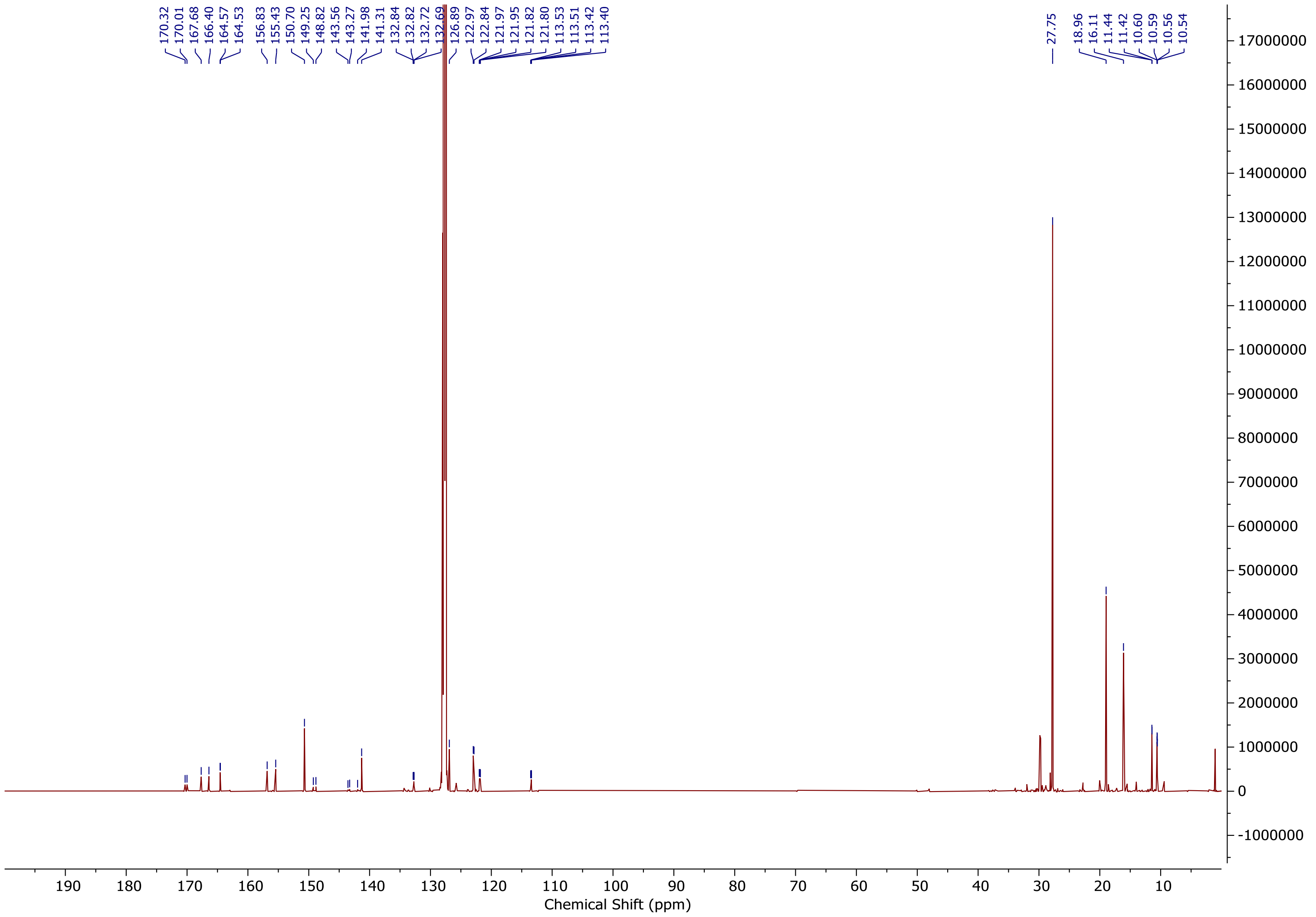

^{13}C-NMR spectrum (176 MHz, C_6D_6) of **7a^{F}**

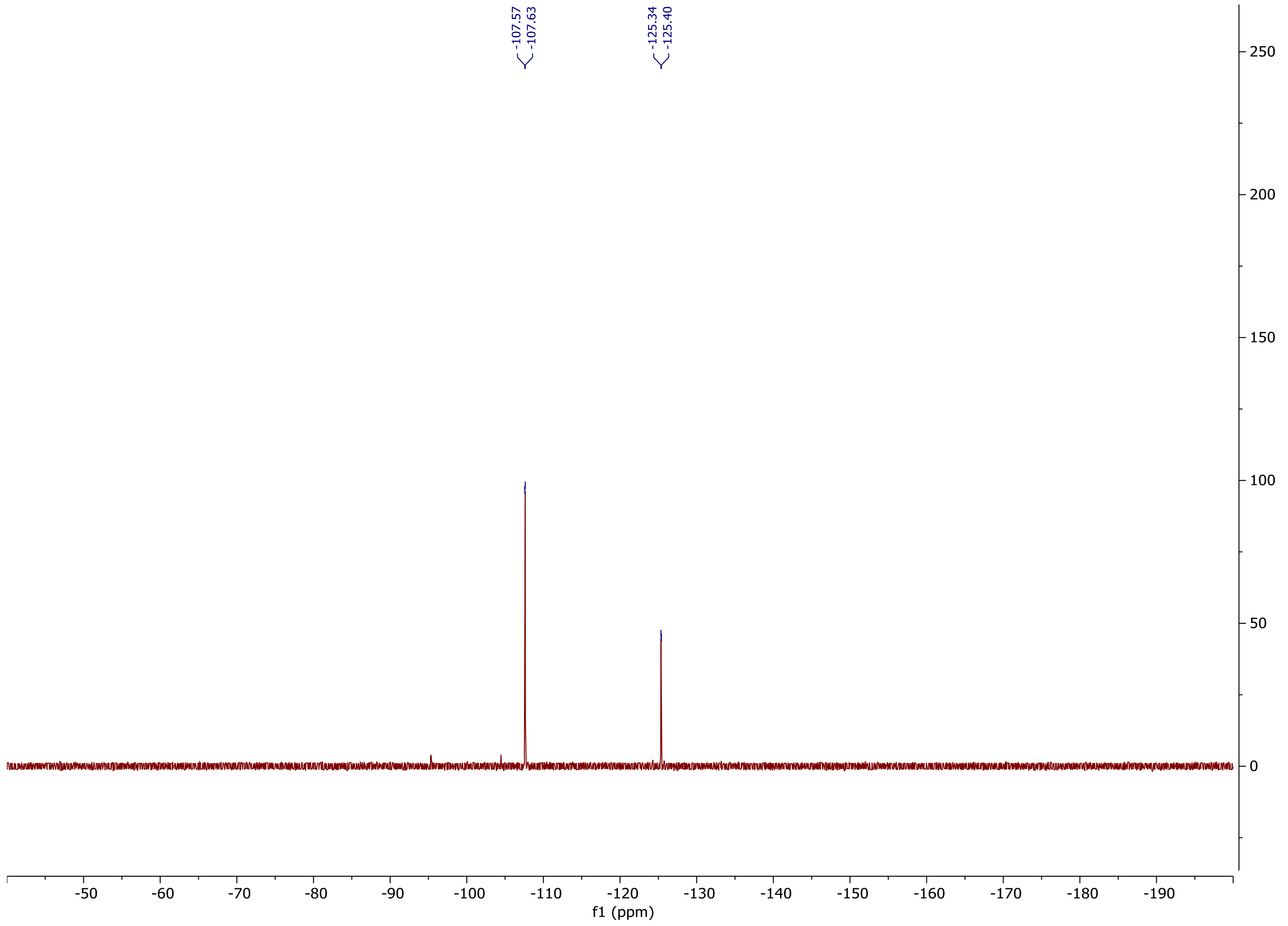

^{19}F-NMR spectrum (376 MHz, C_6D_6) of **7a^{F}**

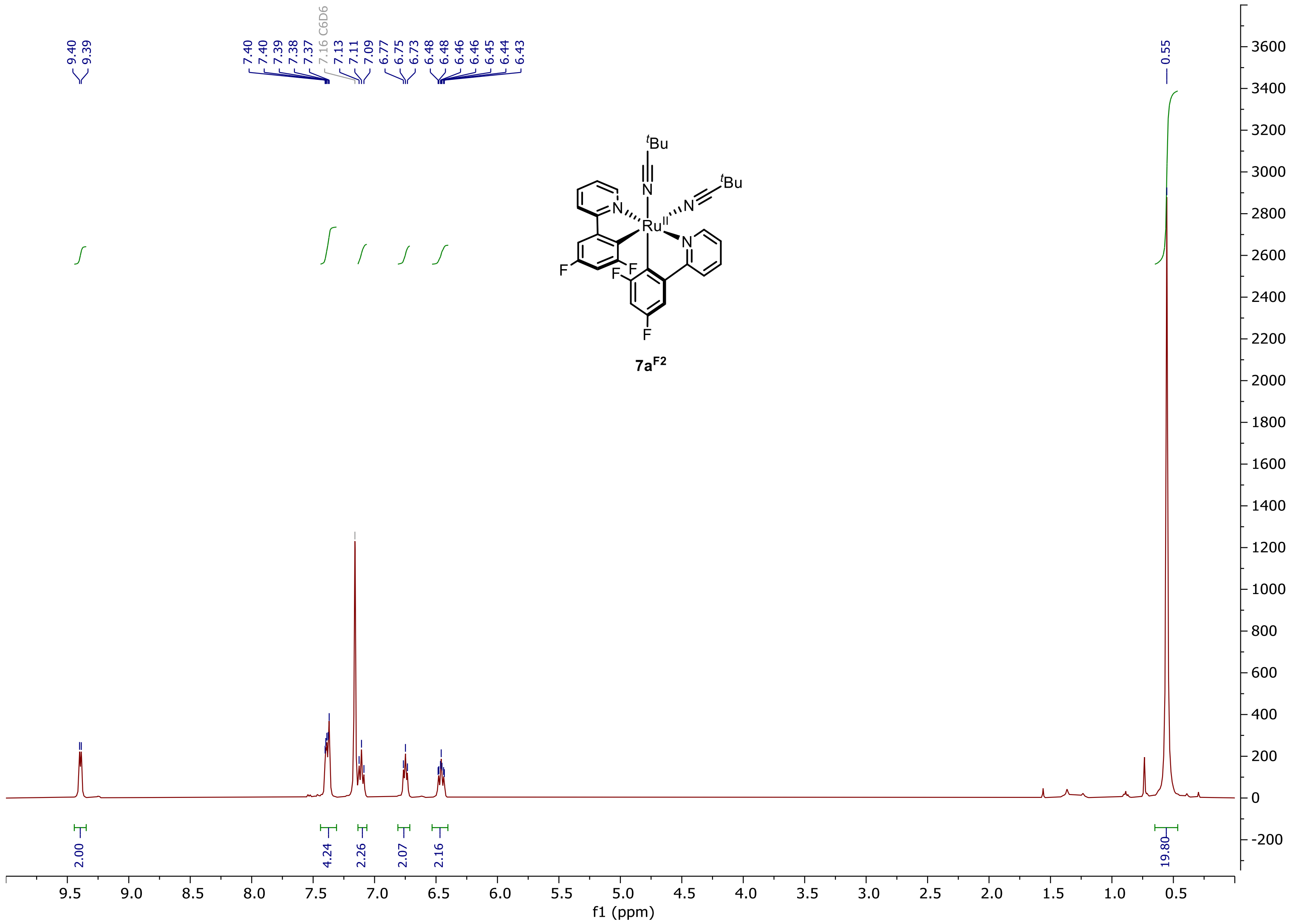

^{1}H-NMR spectrum (400 MHz, C_6D_6) of **7a^{F2}**

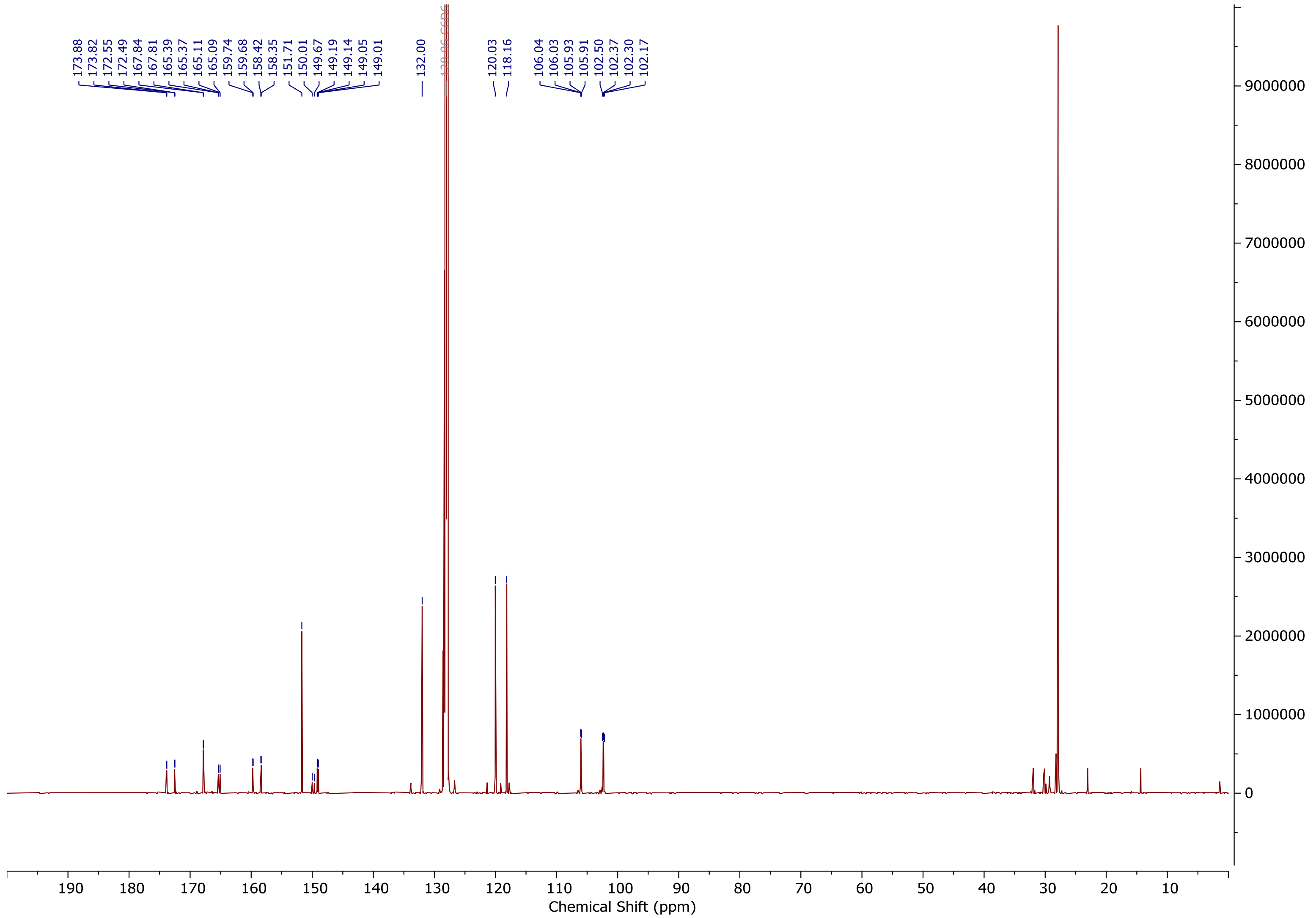

^{13}C-NMR spectrum (176 MHz, C_6D_6) of **7a^{F2}**

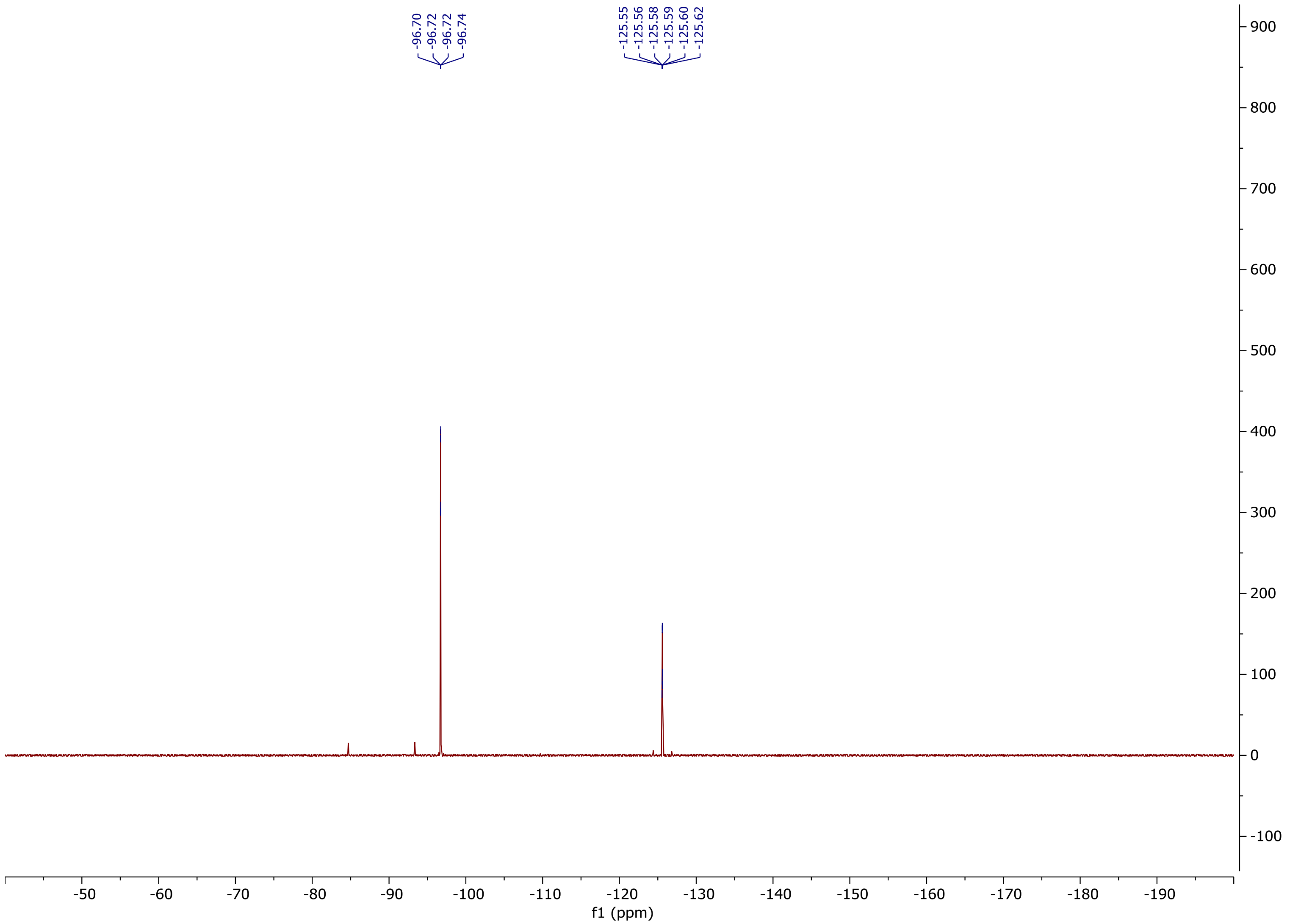

^{19}F-NMR spectrum (376 MHz, C_6D_6) of **7a^{F2}**

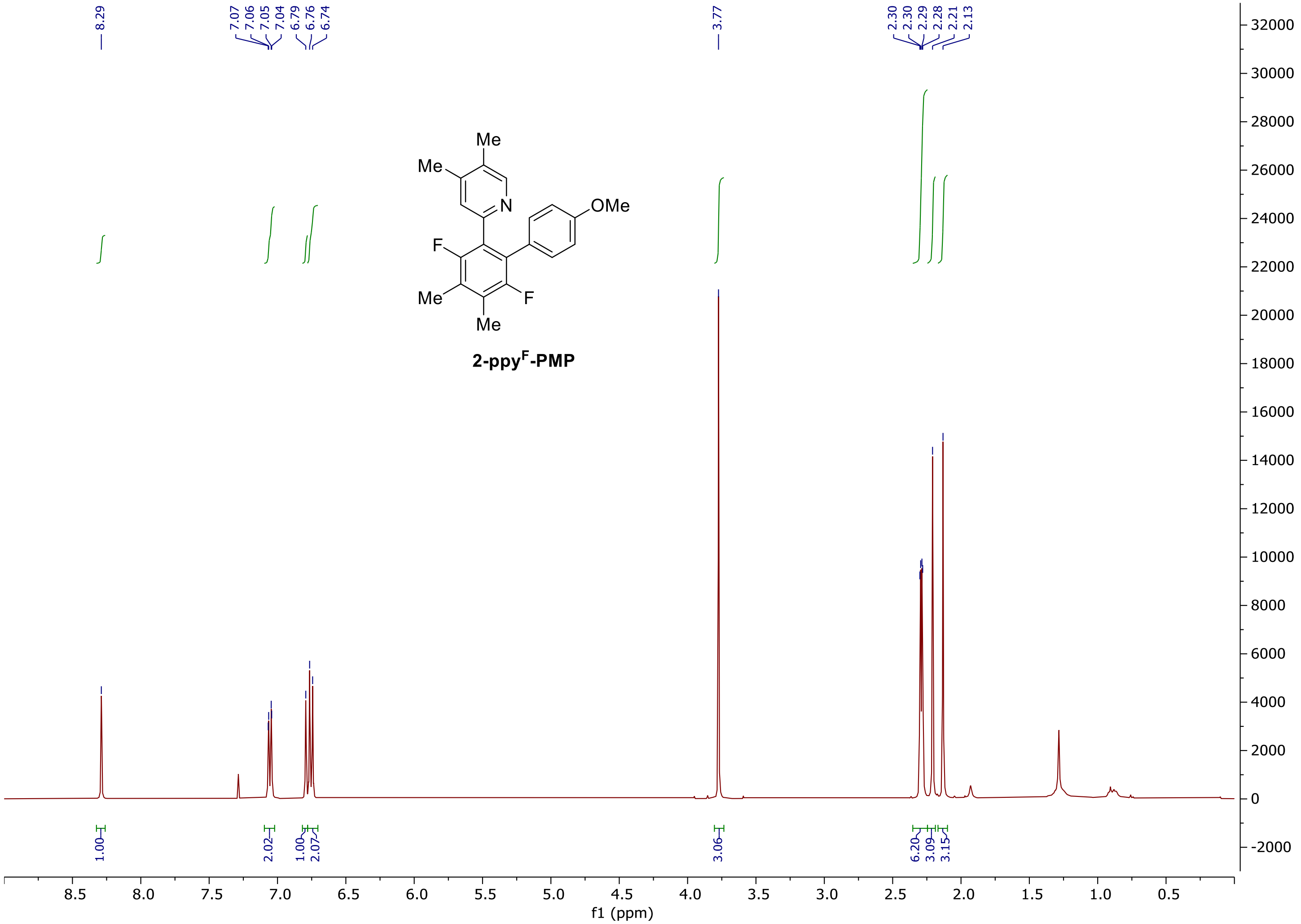

^{1}H-NMR spectrum (400 MHz, $CDCl_3$) of **2-ppy^{F}-PMP**

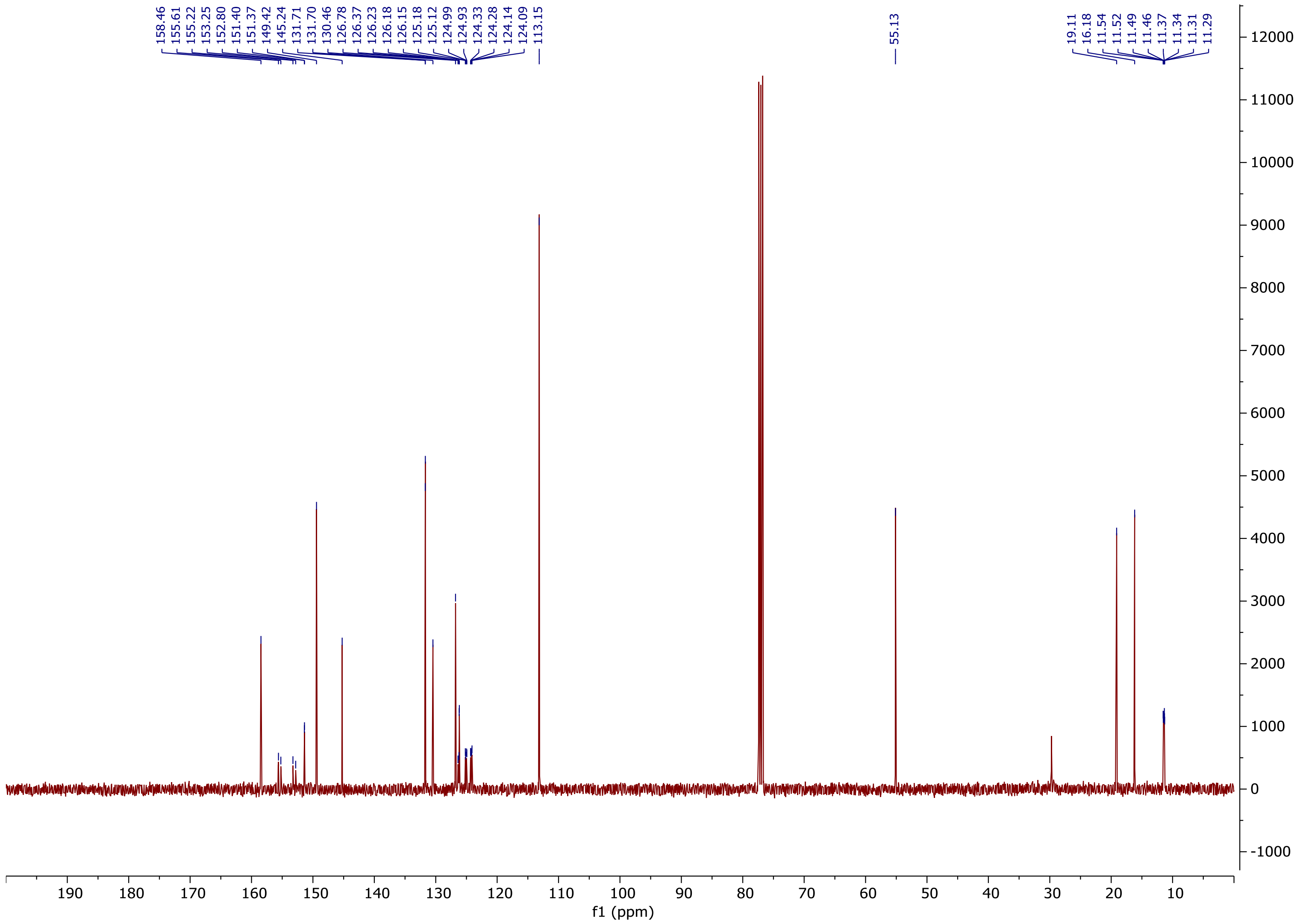

^{13}C-NMR spectrum (101 MHz, $CDCl_3$) of **2-ppy^{F}-PMP**

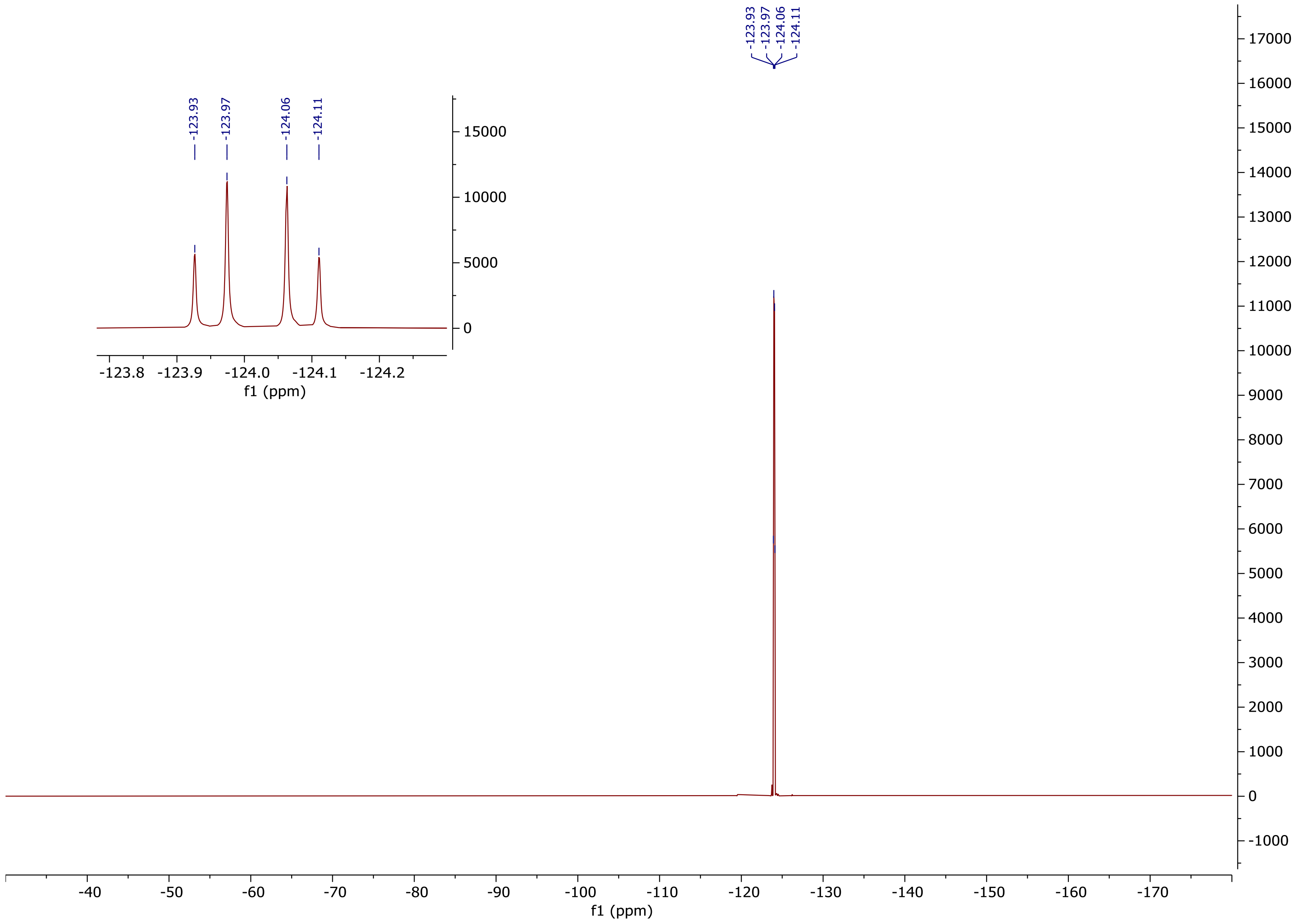

^{19}F-NMR spectrum (376 MHz, $CDCl_3$) of **2-ppy^{F}-PMP**

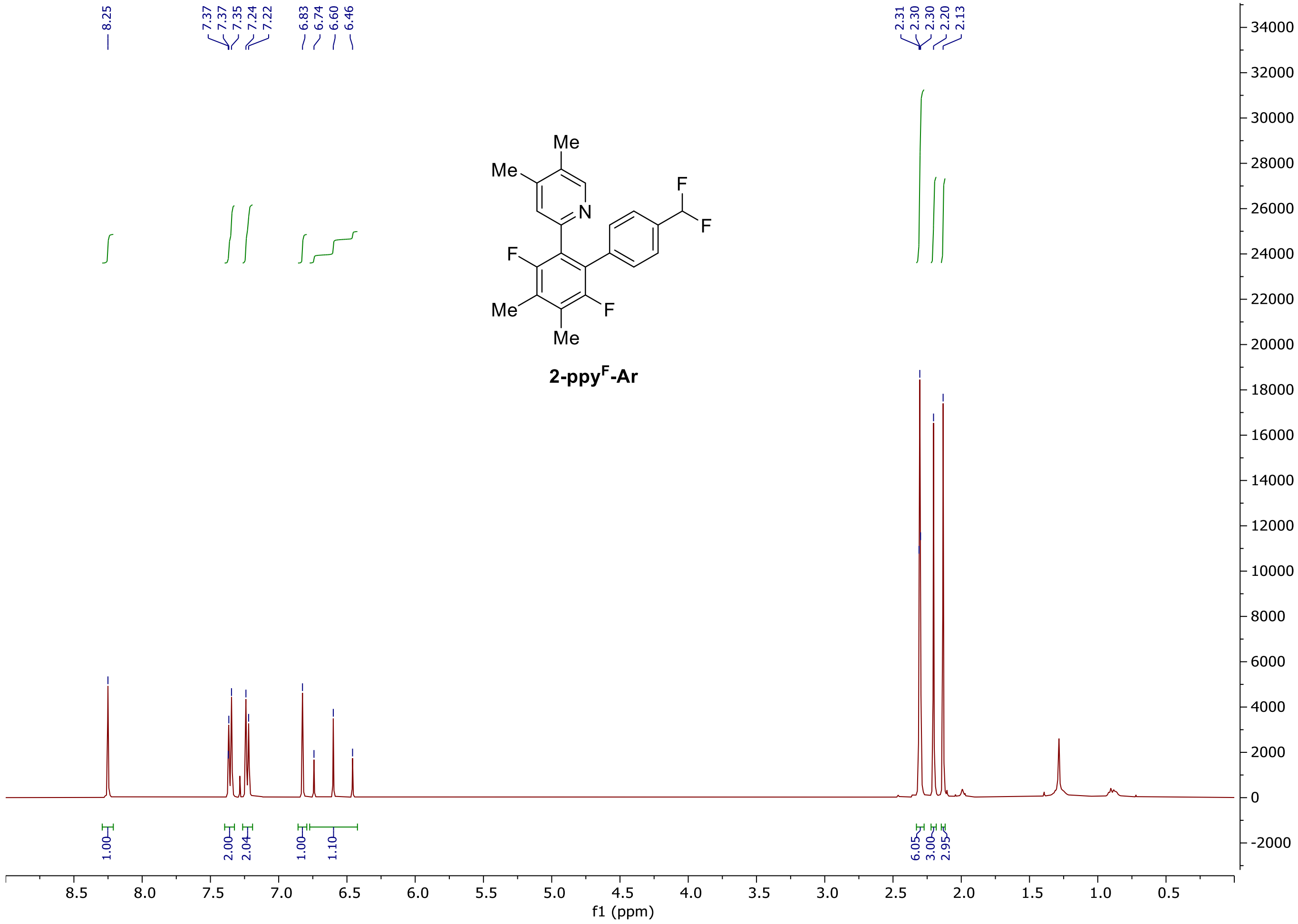

^{1}H-NMR spectrum of (400 MHz, $CDCl_3$) **2-ppy^{F}-Ar**

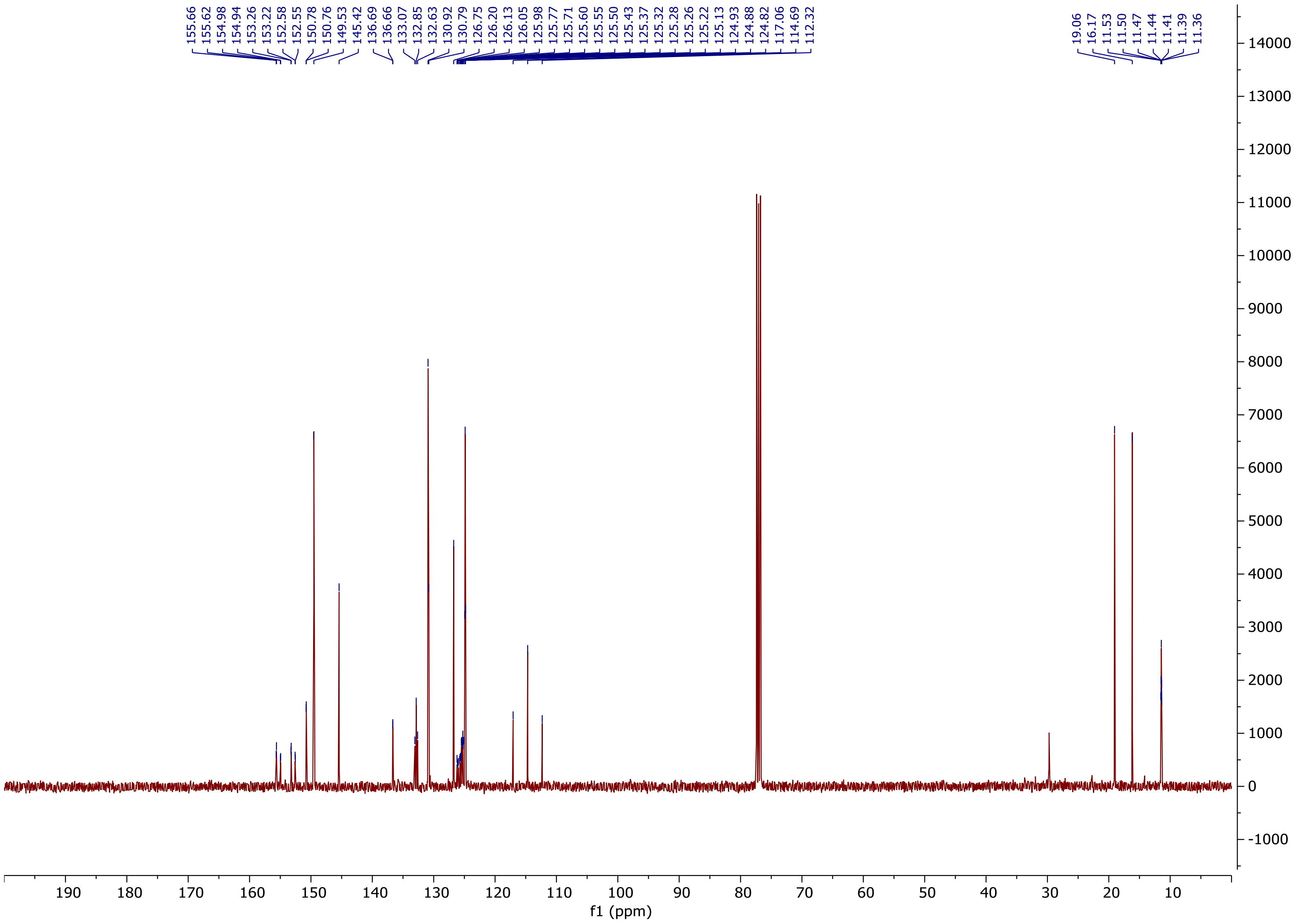

^{13}C-NMR spectrum (101 MHz, $CDCl_3$) of **2-ppy^{F}-Ar**

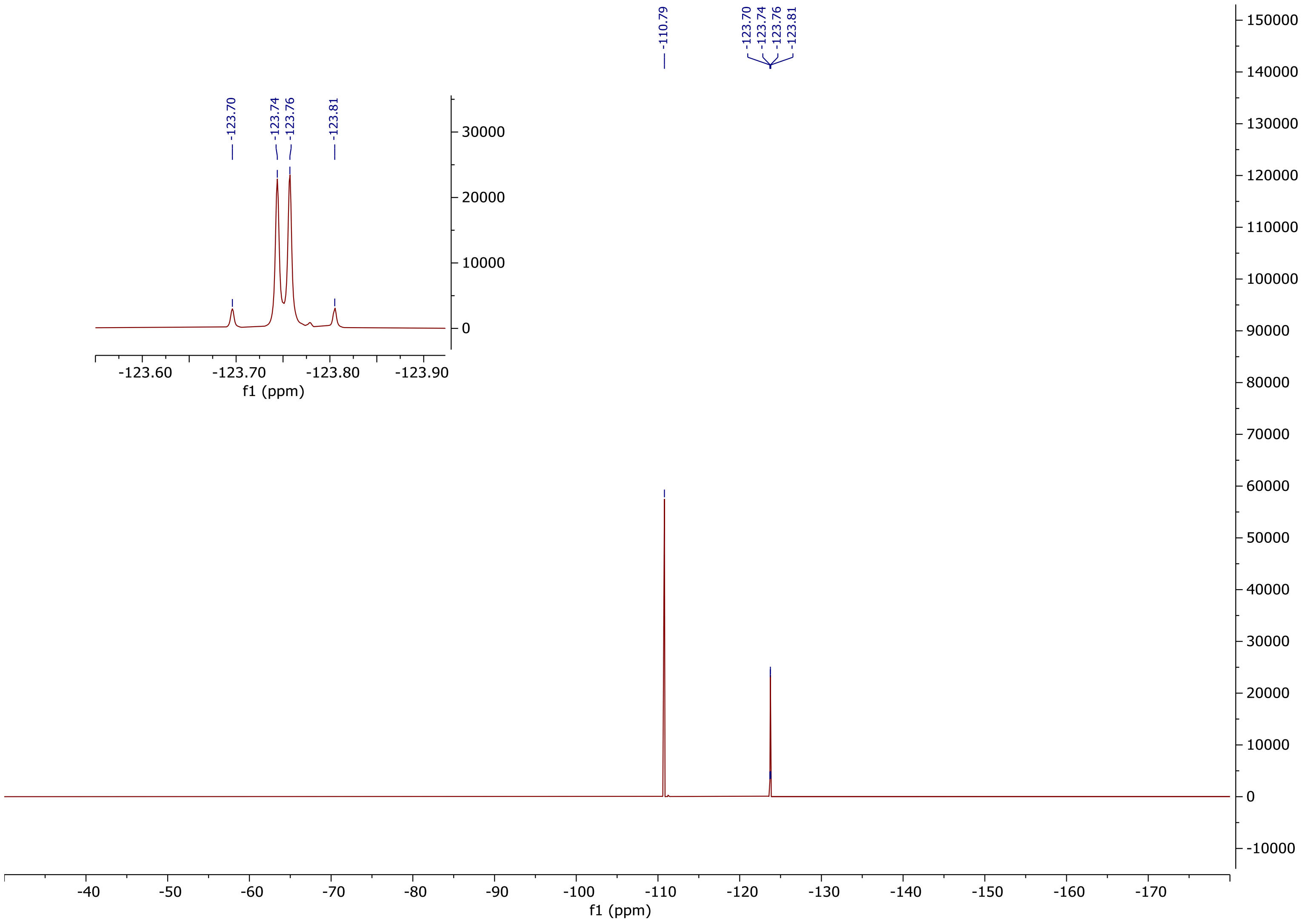

^{19}F-NMR spectrum (376 MHz, $CDCl_3$) of **2-ppy^F-A**

10. Computational Details

DFT calculations were run with Gaussian 16 (Revision A.03).[7] Geometry optimizations and thermodynamic corrections were obtained with the BP86 functional[8,9] and included a correction for solvation effects using a PCM approach[10] within the optimisation procedure. MeOH (ε = 32.6) was chosen as the solvent due to its similar dielectric to the NMP (ε = 32.2) solvent used experimentally. Ru centres were described by a Stuttgart RECP and the associated basis set[11] and 6-31G** basis sets were used for all other atoms.[12,13] All stationary points were fully characterized via analytical frequency calculations as either minima (all positive frequencies) or transition states (one negative frequency) and the latter were characterized via IRC calculations and subsequent geometry optimizations to confirm the adjacent minima. Free energies were computed within the quasi-harmonic approximation in which all low energy vibrational modes were set to 100 cm^{-1} as proposed by Cramer and Truhlar[14] and implemented in the Goodvibes code.[15] Electronic energies were recomputed with the PBE functional[16] using def2-TZVP basis sets[17,18] and including corrections for MeOH solvent and dispersion effects using Grimme's D3 parameter set[19] with Becke-Johnson damping.[20] This protocol was chosen on the basis of functional testing on selected key stationary points where electronic energies with the def2-TZVP basis set were recomputed in MeOH with the BLYP,[8,21]B3LYP,[22] PBE0,[23] B97D [21] B97D3,[24] ωB97x-D,[25] M06,[26] M06L, M06-2X and TPSS[27] functionals. For the BLYP, B3LYP, PBE0, and TPSS functionals these single points included the D3-BJ dispersion correction. All computed structures are provided below as well as in separate files of XYZ coordinates also supplied.

Most stationary points in this study have several possible geometric isomers and these were explored in each case. The isomers are labelled a, b, c etc from the most stable upwards. Alternative pathways were also considered and full details are given in the following. In some cases, this led to all isomers of a plausible species being ruled out on energetic grounds. Such species therefore do not appear in the full manuscript but create a gap in the numerical labelling; for example species **5**, Ru(2-ppy)(κ^1-OAc)(2-ppy-H)$(L)_2$ is not energetically relevant to the initial C–H activation and so does not feature in Figure 3A.

The energies of selected stationary points were recomputed with the full set of functionals described above. This was generally done to assess potentially competing processes, for example Ar–Br activation via COA, SET and HAA at a given intermediate, or the competition between the lowest energy mon- and bis-cyclometalated species. Details are shown in Figures S17, S19, S25, S26, S37, S38, S48 and S19.

11. C-H Arylation via bis-cyclometalated intermediates.

11.1. Formation of isomers of 4 from 1.

Figure S13 shows initial L/OAc$^-$ substitution is easiest trans to the aryl carbon to form $\mathbf{2_{OAc}}$. This can then access $\mathbf{3_{OAc}}$ via $\mathbf{TS(2a\text{-}3a)_{OAc}}$ at +17.5 kcal/mol. Reactions via **Int(1-2)b** or **Int(1-2)c** (and the adducts that would be formed upon subsequent OAc addition) are disfavoured by the high isomerisation barriers from **Int(1-2)a**. Dissociative L/2-ppy-H substitution trans to the pyridyl N then gives **4a** at -7.4 kcal/mol (Figure S14). Isomers of **5** which feature a κ^1-OAc ligand were assessed but were significantly higher in energy.

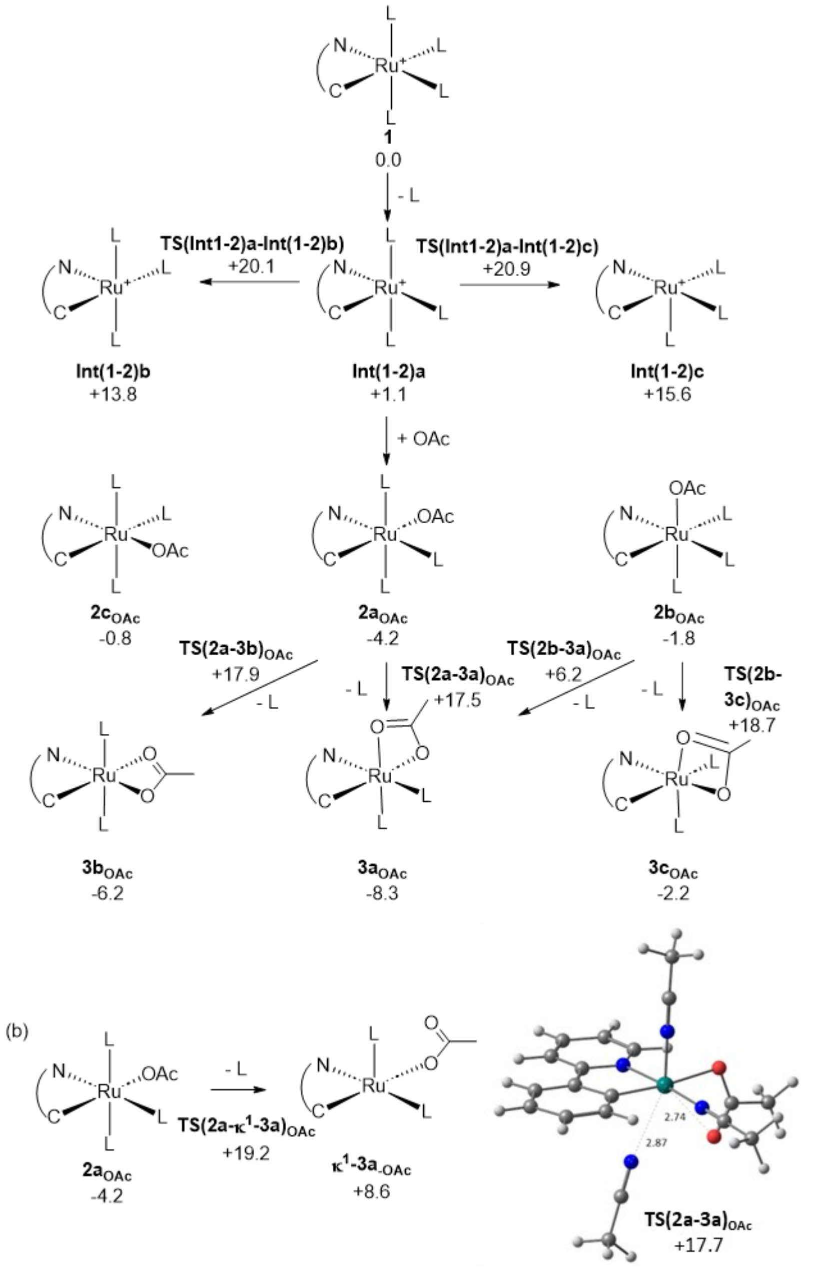

Figure S13. (a) Computed free energies (kcal/mol) for stationary points implicated in the formation of isomers of Ru(2-ppy)(κ^2-OAc)L$_2$, **3$_{OAc}$** (L = MeCN); (b) alternative MeCN dissociation from **2a$_{OAc}$** maintaining a κ^1-OAc ligand; (c) geometry of key transition state **TS(2a-3a)$_{OAc}$** is also shown. Method: PBE(def2-tzvp,MeOH,D3BJ)//BP86(MeOH,SDD,6-31G**).

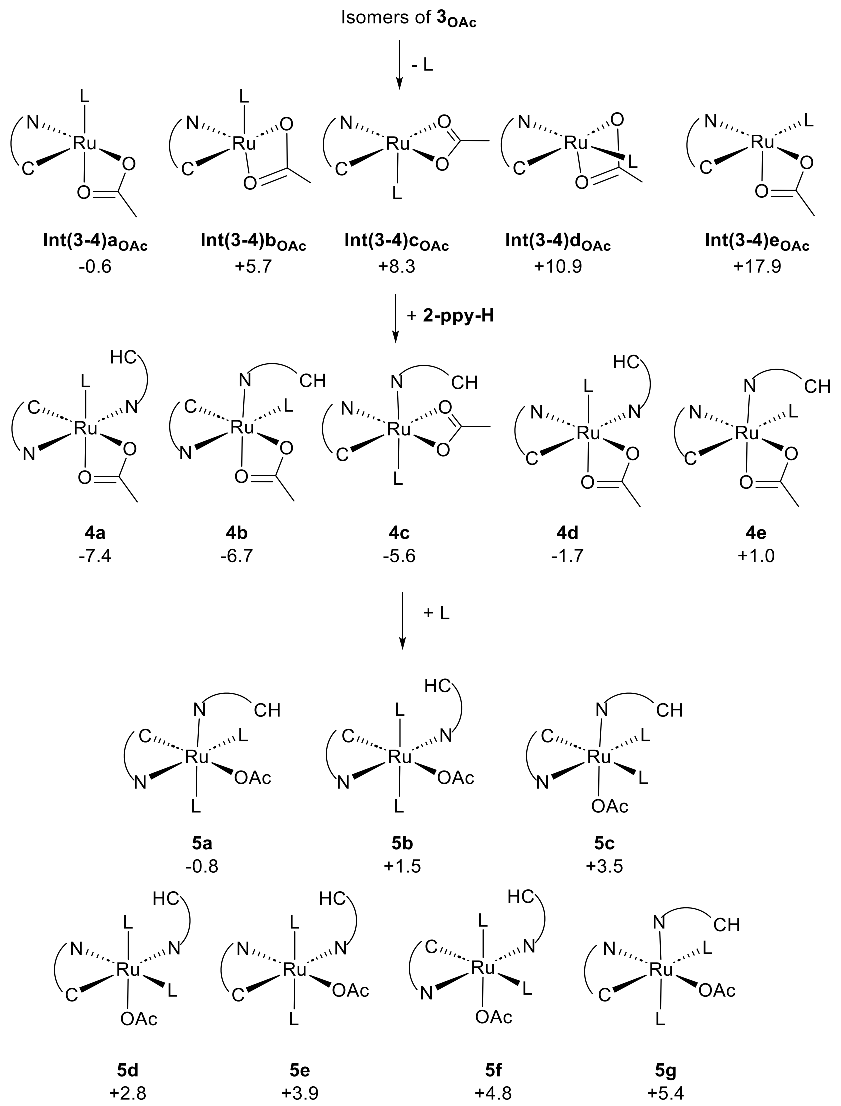

Figure S14. Computed free energies (kcal/mol) for stationary points implicated in the formation of Ru(2-ppy)(κ^2-OAc)(2-ppy-H)L, **4**, and Ru(2-ppy)(κ^1-OAc)(2-ppy-H)L_2, **5**, (L = MeCN). Method: PBE(def2-tzvp,MeOH,D3BJ)// BP86(MeOH,SDD,6-31G**).

The formation **4** from **1** via initial L/2-ppy-H substitution was also assessed and first involves **2a$_{2\text{-ppy-H}}$** at +2.4 kcal/mol (Figure S15). Loss of L trans to the pyridyl N then leads to **3f$_{2\text{-ppy-H}}$** at +9.4 kcal/mol via **TS(2a-3f)$_{2\text{-ppy-H}}$** at +23.3 kcal/mol. Isomerisation of **3f$_{2\text{-ppy-H}}$** to **3b$_{2\text{-ppy-H}}$** places the pyridyl N atoms trans to each other. L loss then gives **Int(3-4)$_{2\text{-ppy-H}}$** for which 9 isomers were located with C–H agostic interactions. Two π-bound isomers were also located with other π-bound or agostic geometries isomerising to one of those shown in Figure S15. OAc^- addition to **Int(3-4)b$_{2\text{-ppy-H}}$** then leads to **4a**.

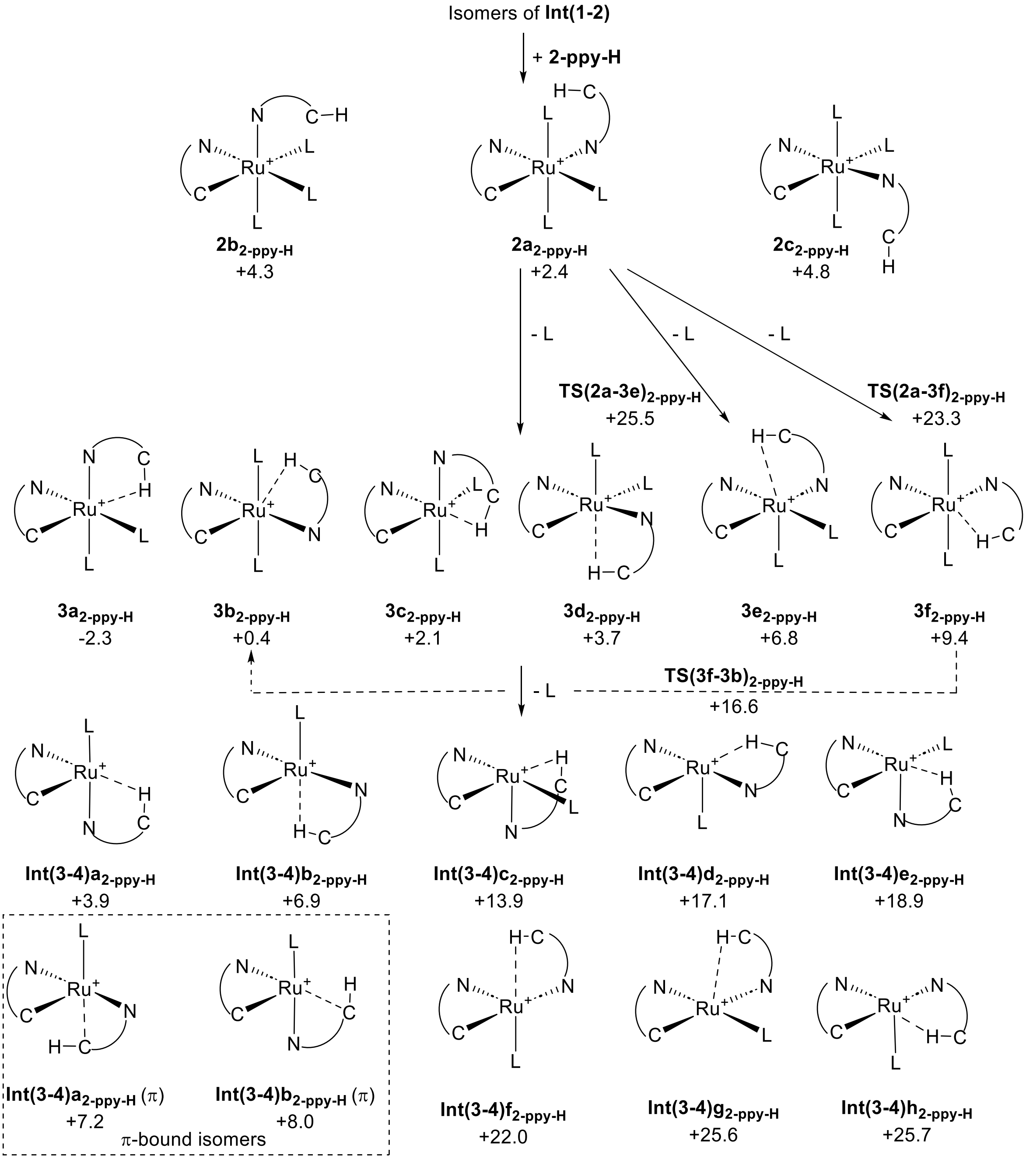

Figure S15. Computed free energies (kcal/mol) for stationary points implicated in the formation of Ru(2-ppy)(κ^2-OAc)(2-ppy-H)L, **4**, and Ru(2-ppy)(κ^1-OAc)(2-ppy-H)L$_2$, **5**, (L = MeCN). Method: PBE(def2-tzvp,MeOH,D3BJ)// BP86(MeOH,SDD,6-31G**).

The most accessible pathways for the formation of **4** either via initial L/OAc⁻ or L/2-ppy-H substitution are compared in Figures S16A and S16B respectively. The former pathway is kinetically favoured with an overall energy span of 21.7 kcal/mol compared to 23.3 kcal/mol for the latter.

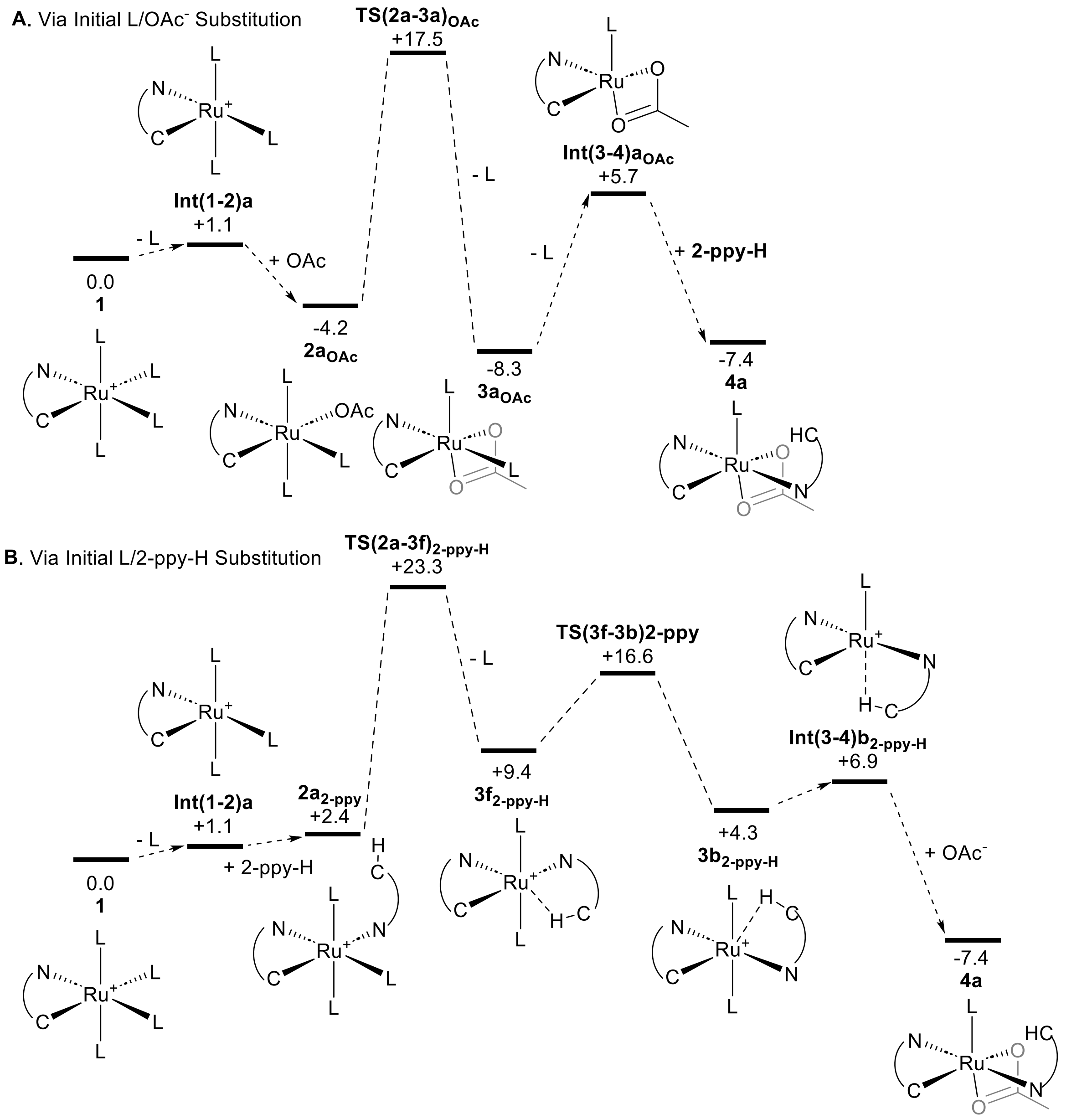

Figure S16. Comparison of the lowest energy pathways (kcal/mol) for the formation of **4a** via (A) initial L/OAc⁻ substitution and (B) initial L/2-ppy-H substitution. Method: PBE(def2-tzvp,MeOH,D3BJ)// BP86(MeOH,SDD,6-31G**).

Recomputing these energy spans with different functionals shows the preferred pathway is sensitive to functional choice (Figure S17). However, in all cases, these initial ligand substitutions have higher barriers than the subsequent C–H activation (see Figure S18a). The overall conclusion, that initial ligand substitution is the slow step of the initiation process that forms the active bis-cyclometalated species, is therefore independent of functional choice.

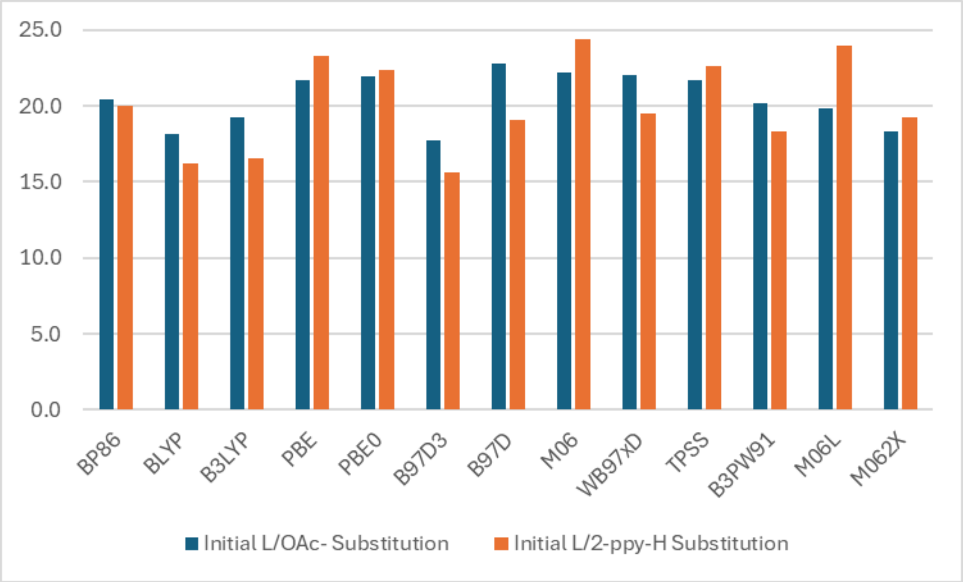

Figure S17. Free energy spans (kcal/mol) computed with different functionals for competing mechanisms for the formation of **4a**.

11.2 C-H activation pathways to form bis-cyclometalated intermediates.

C–H activation from all isomers of **4** was assessed (Figures S18a and S18b). The lowest energy pathway stems from rotamer **4a'**, accessed via **TS(4a-4a')** at +5.3 kcal/mol. C–H activation is then a two-step AMLA-CMD process via **Int(4a’-6a)** to form first **6a** at -7.2 kcal/mol and then **7a** via HOAc/L substitution. The overall energy span is 10.5 kcal/mol, with 2-ppy-H rotation via **TS(4a-4a’)** being the highest transition state.

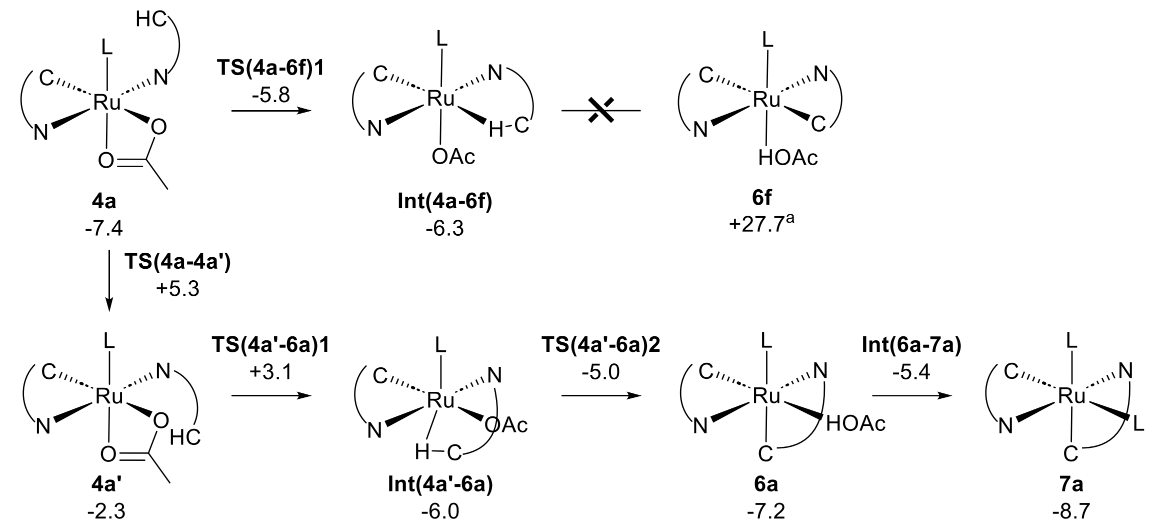

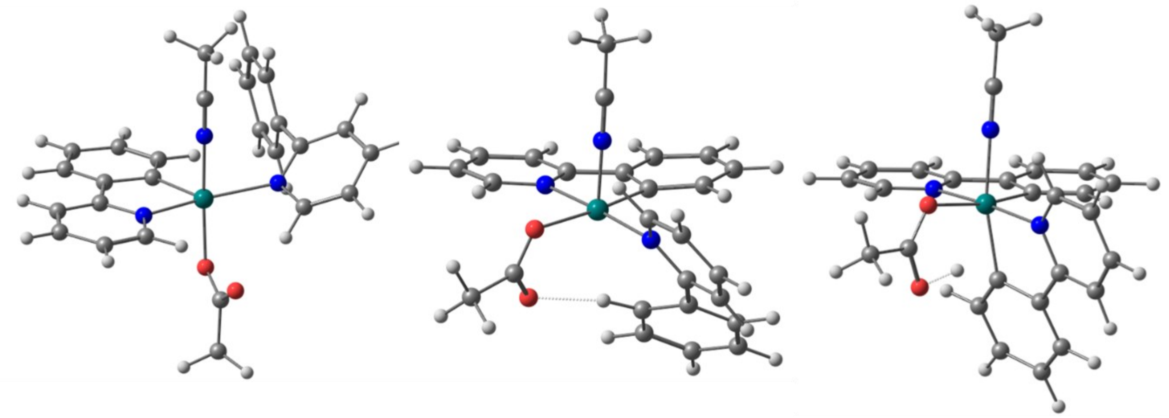

TS(4a-4a') +5.3 **TS(4a'-6a)1** +3.1 **TS(4a'-6a)2** -5.0

Figure S18a. Computed free energies (kcal/mol) for C–H activation from **4a** with key transition states also shown. Method: PBE(def2-tzvp,MeOH,D3BJ)//BP86(MeOH,SDD,6-31G**). [a]**6f** could only be located as a local minimum if the HOAc proton is oriented away from the *cis*-C_{aryl} ligand.

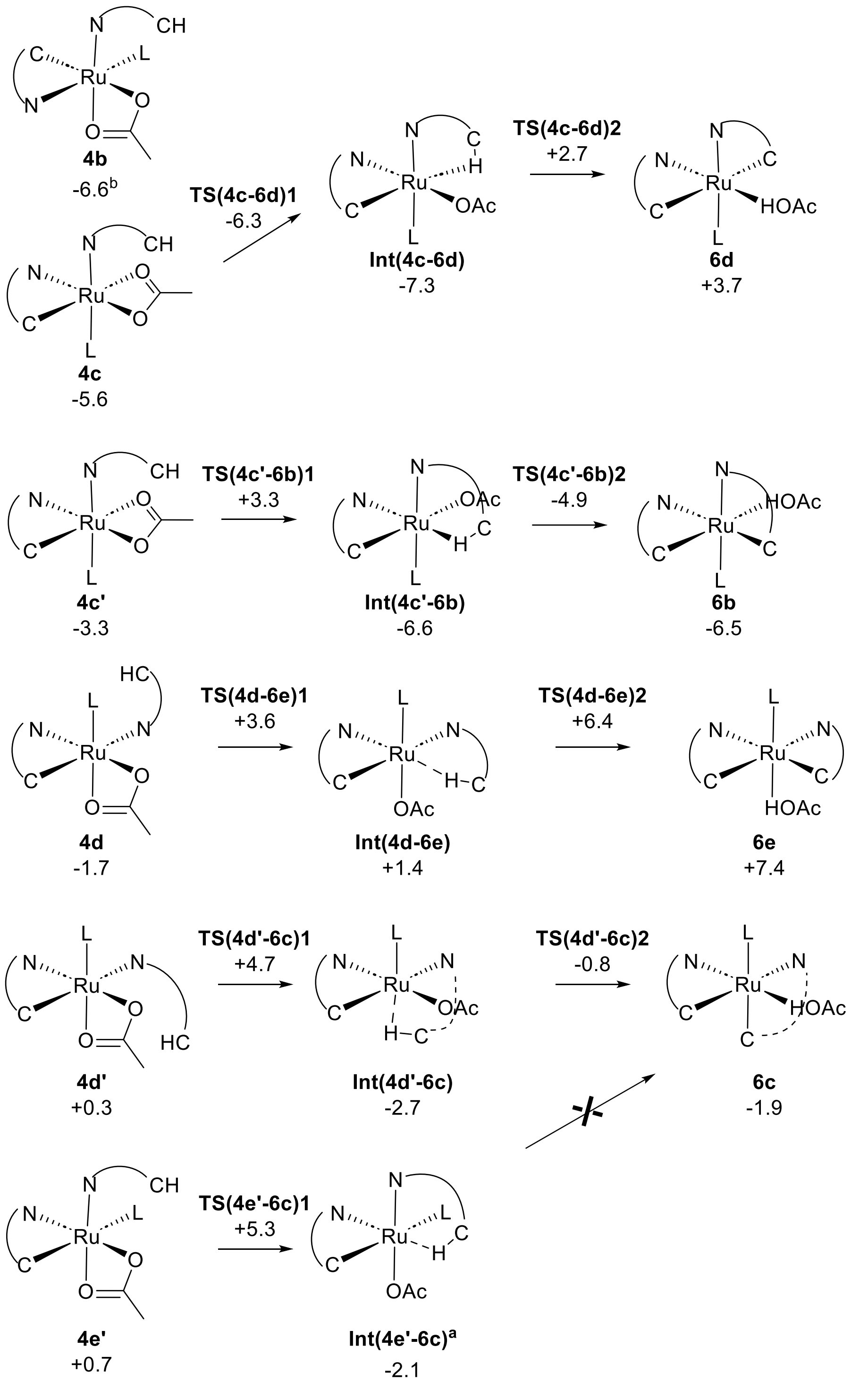

Figure S18b. Computed free energies (kcal/mol) for alternative C–H activation processes from **4b-e**. Method: PBE(def2-tzvp,MeOH,D3BJ)//BP86(MeOH,SDD,6-31G**). [a]a transition state for C–H activation in **Int(4e'-6d)** could not be located; however the energy of **TS1(4e'-6c)** is sufficient to rule out this pathway. [b]C–H activation from **4b** is enantiomeric with that from **4c**.

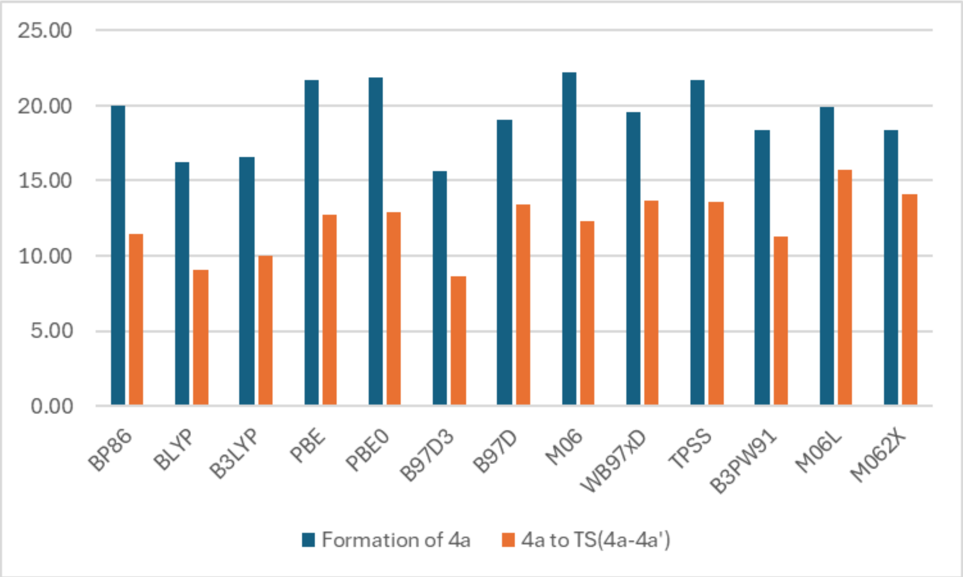

Figure S19. Free energies (kcal/mol) computed with different functionals comparison energy spans for the formation of **4** from **1** and for C–H activation from **4**. For the formation of **4** the lowest energy pathway (i.e. L/OAc⁻ substitution or L/2-ppy-H substitution) was used, as appropriate; for C–H activation the energy span corresponds to 2-ppy-H rotation via **TS(4a-4a')**.

11.3 Ph-Br activation at bis-cyclometalated intermediates

A speciation study of bis-cyclometalated intermediates that could be formed prior to Ph–Br activation was performed (see Figure S20). All isomers of **7** were assessed and those based on the *cis,trans*- and *cis,cis*-Ru(2-ppy)$_2$ fragments were considerably favoured (compare **7a/7b** *cf*. **7c-7e**). Only these low energy isomers of **8** and **9** were considered, along with selected π-bound isomers of PhBr complexes.

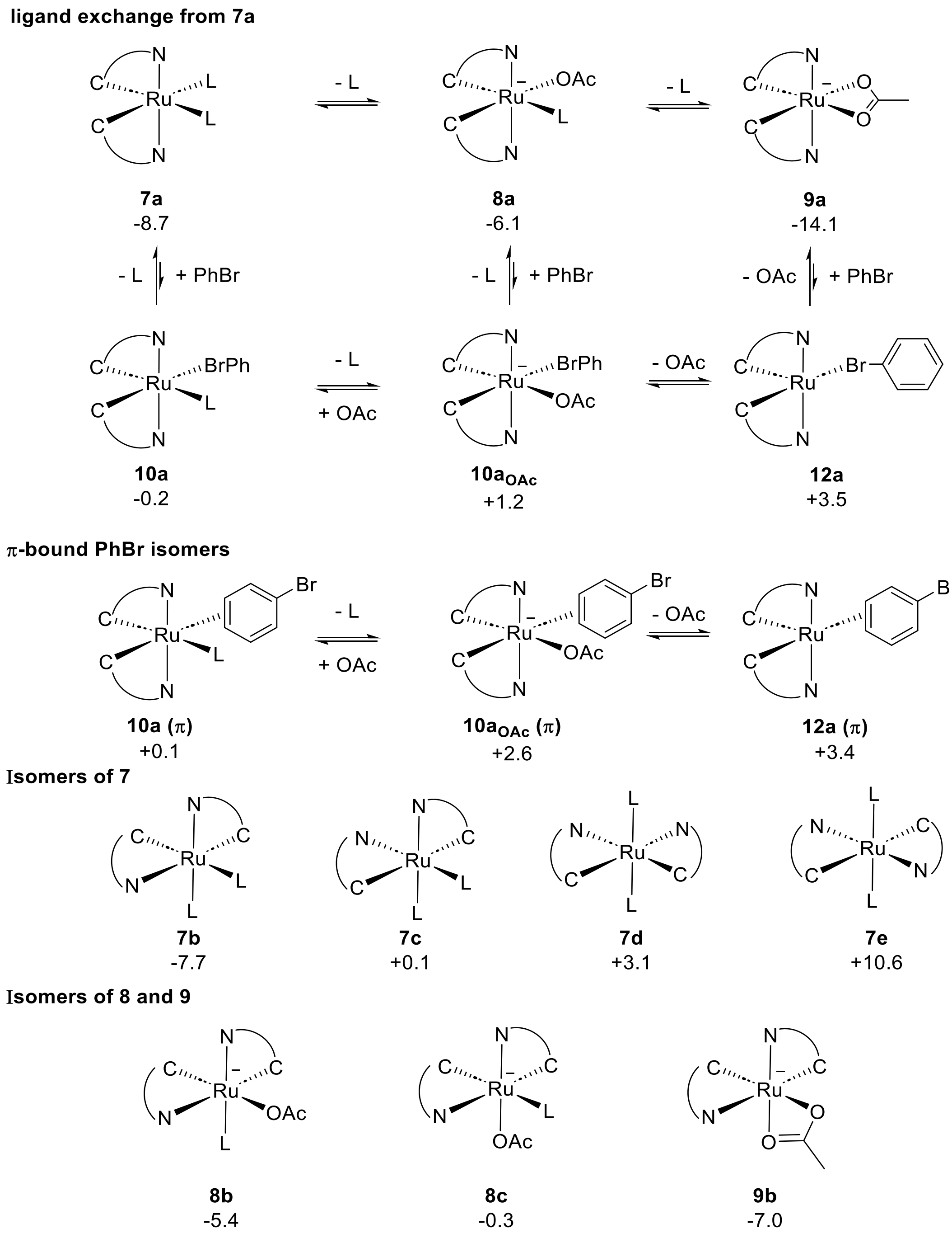

Figure S20. Computed free energies (kcal/mol) for bis-cyclometalated intermediate **7**, *trans,cis*-Ru(2-ppy)$_2$(MeCN)$_2$ and species **8, 9** and **12** formed via substitution of one or both MeCN ligands. Method: PBE(def2-tzvp,MeOH,D3BJ)//BP86(MeOH,SDD,6-31G**).

11.3.1. Concerted oxidative addition (COA) from isomers of 5-coordinate 12

COA of PhBr in isomers **12a-d** was assessed with reaction of **12a** being clearly the most accessible via **TS(12a-13c)** at +6.9 kcal/mol (Figure S21). This forms Jahn-Teller distorted Ru(IV) species **13**, the most stable of which, **13a**, is formed via isomerisation of **13c**.

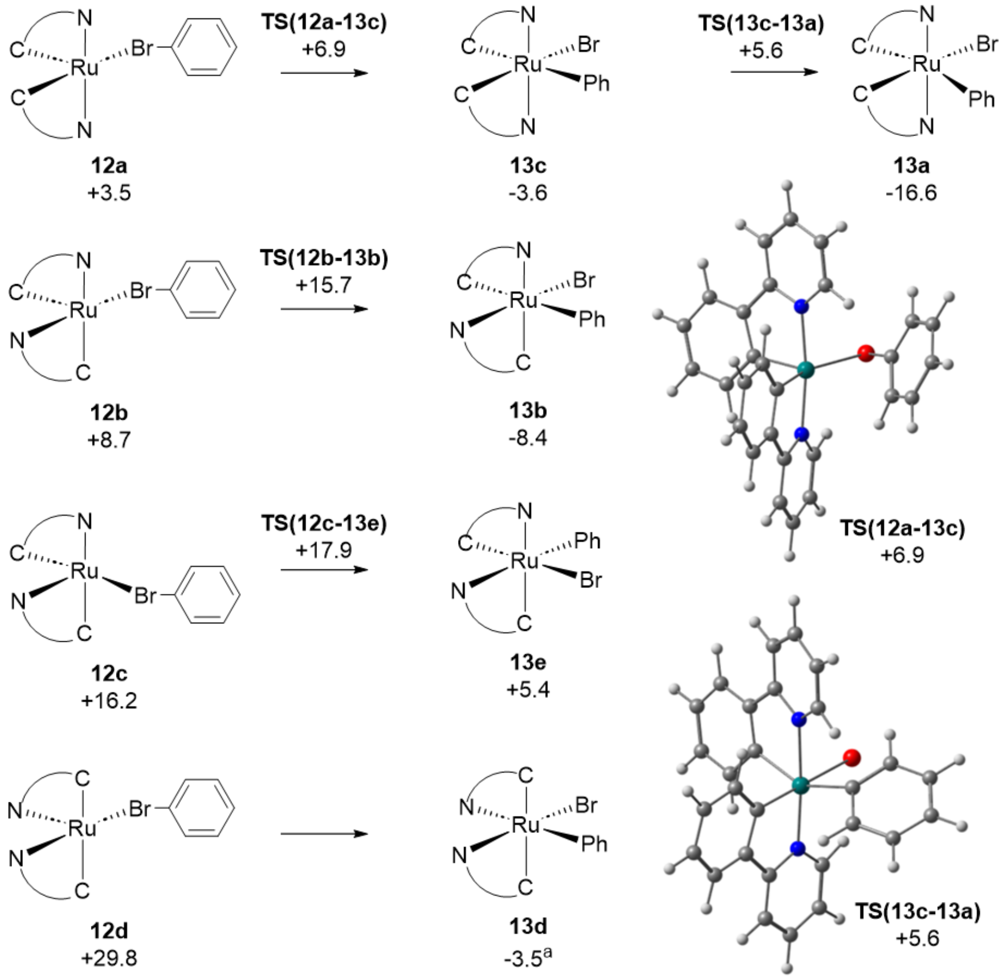

Figure S21. Ph–Br activation pathways via concerted oxidative addition from isomers of **12** with key transition states also shown. Method: PBE(def2-tzvp,MeOH,D3BJ)//BP86(MeOH,SDD,6-31G**) [a]Location of a transition state for Ph–Br activation from the high energy isomer **12d** was not attempted.

11.3.2 Single electron transfer (SET)

Figure S22 gives the thermodynamics of SET from **7a**, **8a** and **9a** leading to formation of the corresponding Ru(III) species and, in principle, a [PhBr]$^{-}$ radical anion. However, the latter does not correspond to a local minimum but rather undergoes C–Br cleavage to form Ph· and Br^{-}. These were therefore computed as separate species and as such will maximise any entropic stabilisation. The overall free energies in Figure S22 are therefore a lower limit to the thermodynamics of this step.

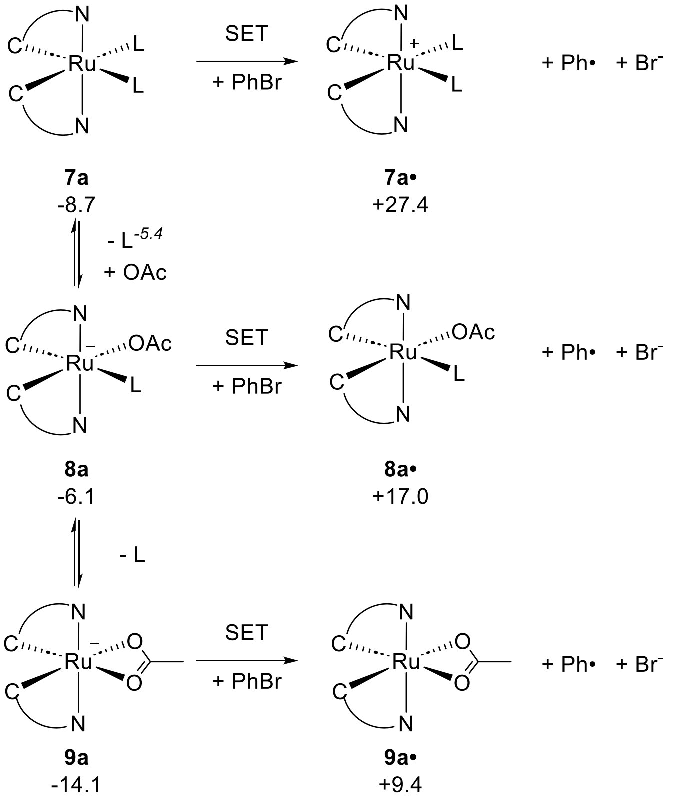

Figure S22. Computed free energies (kcal/mol) for single electron transfer (SET) to PhBr from **7a**, **8a** and **9a**. Method: PBE(def2-tzvp,MeOH,D3BJ)//BP86(MeOH,SDD,6-31G**)

11.3.3 Halogen atom abstraction (HAA)

Figure S23 gives the thermodynamics of HAA from **8a**, **10a$_{OAc}$** and **12a** to form the corresponding Ru(III) species and a phenyl radical. These product species are computed separately, maximising any entropic stabilisation and hence providing a lower limit to the thermodynamics of this step. Linear transits probing the cleavage of the Ph–Br bond on the open-shell singlet pathway (broken symmetry mode) generally tended towards a limit and implied a minimal activation barrier for the reverse radical recombination.

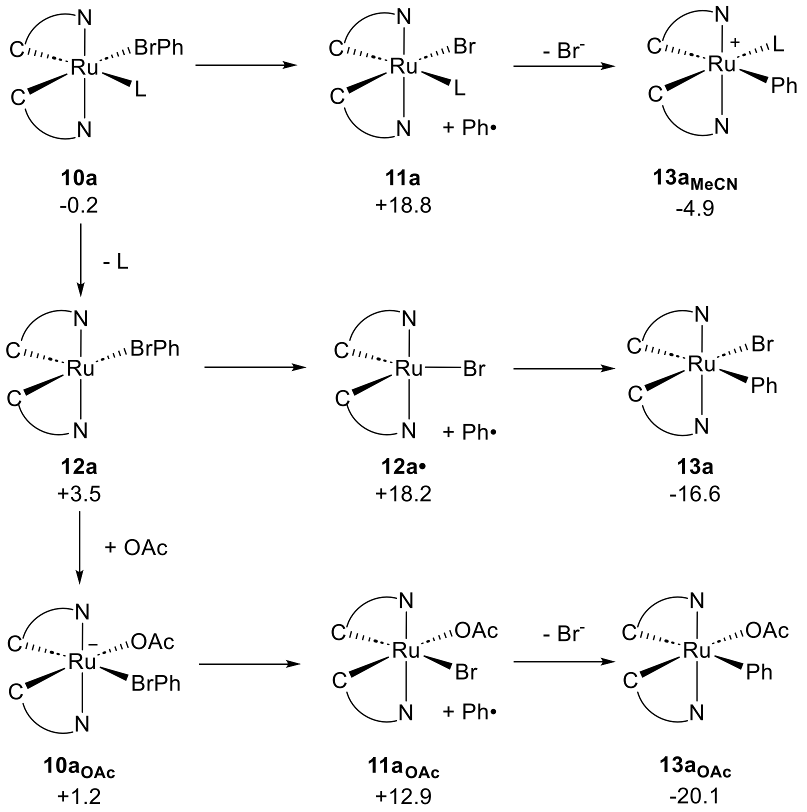

Figure S23. Computed free energies (kcal/mol) for halogen atom abstraction (HAA) from **8a**, **10a$_{OAc}$** and **12a**. The product/s of radical recombination after Br$^-$ loss (where necessary) are also provided. Method: PBE(def2-tzvp,MeOH,D3BJ)//BP86(MeOH,SDD,6-31G**).

11.3.4 Concerted oxidative addition (COA) from isomers of 6-coordinate 8 and 10

This possibility was considered by computing possible isomers for the 7-coordinate Ru(IV) species, **16bis**, formed upon COA in 6-coordinate **8** and **10** (see Figure S24). C–Br activation transition states were then located by scanning backwards from the 7-coordinate species. The most stable transition state located is **TS(8a-16a^{bis})** but at +17.1 kcal/mol, this is considerable higher than **TS(12a-13c)** at +6.9 kcal/mol.

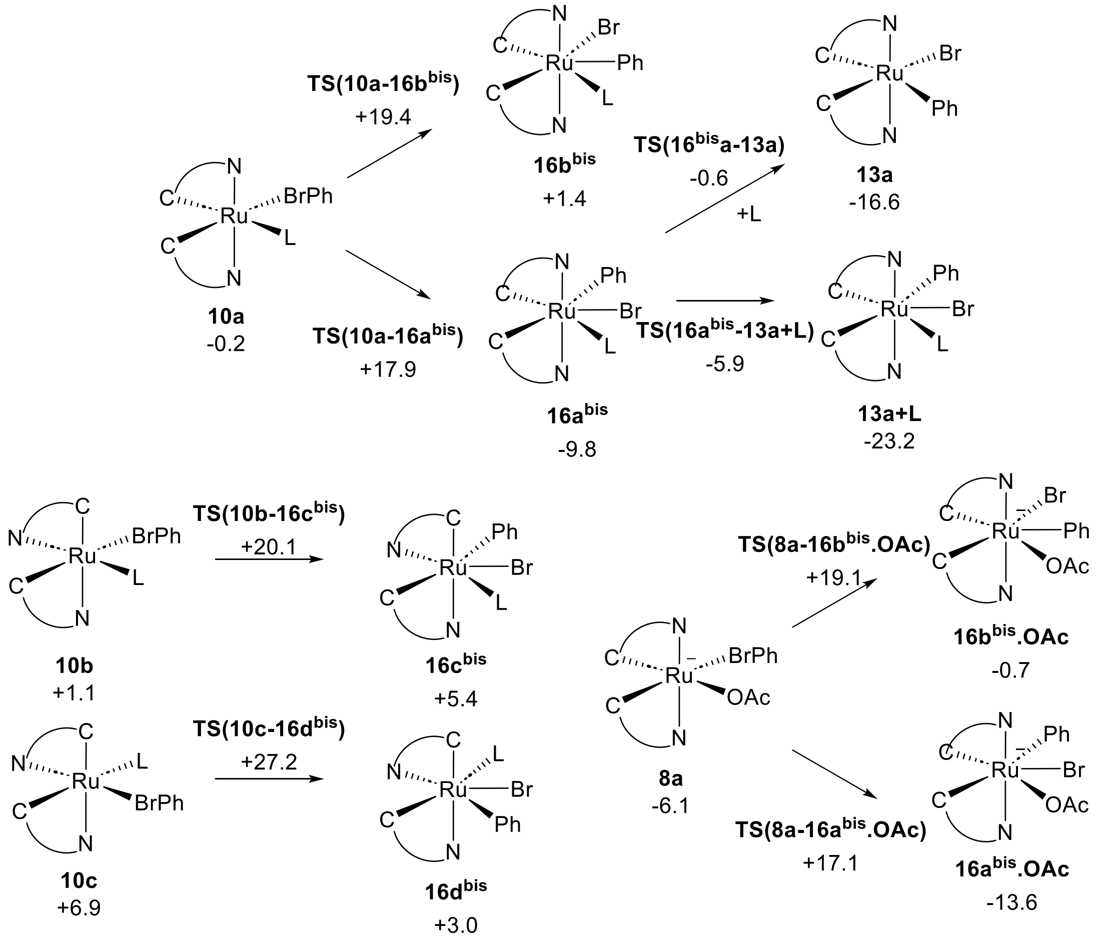

Figure S24. Computed free energies (kcal/mol) for Ph–Br activation via concerted oxidative addition from 6-coordinate **8a** and isomers of **10**. Method: PBE(def2-tzvp,MeOH,D3BJ)//BP86(MeOH,SDD,6-31G**). Transition states and products of L loss and C–C reductive coupling in **16a^{bis}** are also shown.

The functional dependence of the competing COA, SET and HAA reactions at **7a** was assessed and is shown in Figure S25. For COA the energy span from **7a** to **TS(12a-13c)** is given while data for HAA and SET represent the thermodynamic change associated with these steps (see above). Although absolute values vary considerably, COA is clearly the most accessible route, independent of the functional employed.

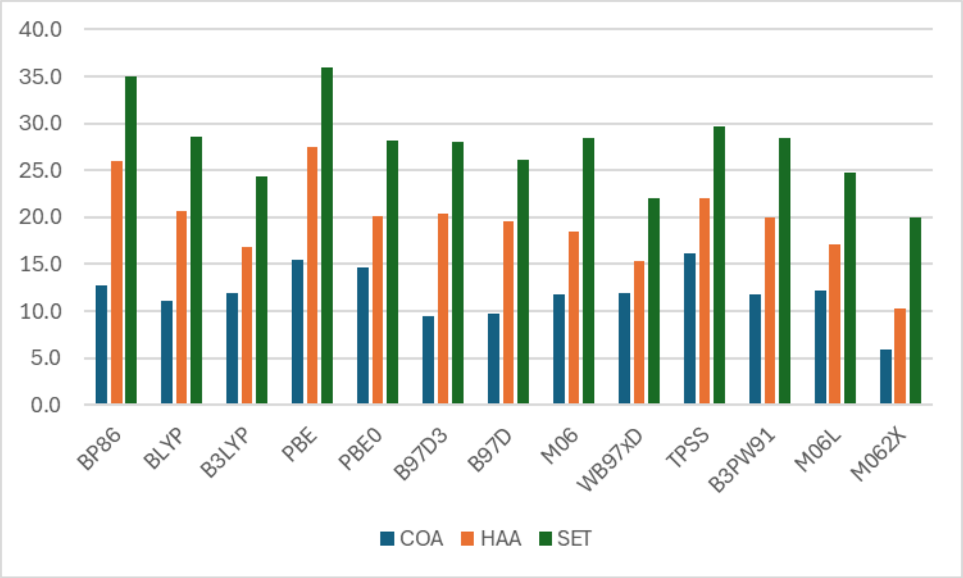

Figure S25. Free energies (kcal/mol) computed with different functionals for Ph–Br activation at **7a** via concerted oxidative addition (COA), halogen atom abstraction (HAA) and single electron transfer (SET).

Experimentally, the addition of KOAc had no effect on the kinetics of arylation (see Figure S12) and so computed results for Ph–Br activation have been based on the reactions of **7a**. However, computationally the displacement of two MeCN ligands in **7a** by OAc^- was found to be thermodynamically favorable, giving **9a** with ΔG = -5.4 kcal/mol at the PBE(def2-tzvp,MeOH,D3BJ)//BP86(MeOH,SDD,6-31G**) level (see Figure S20). For completeness therefore, COA, SET and HAA were modelled from **9a**, with the results provided across Figures S21-S23. Here the lowest energy pathways are compared with the full range of functionals in Figure S26 which also shows the energy of formation of **9a** from **7a**.

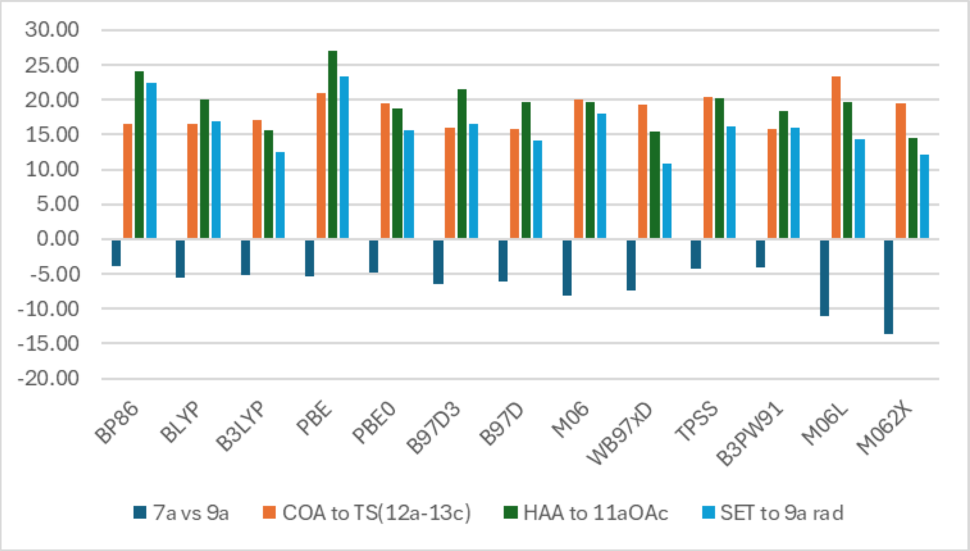

Figure S26. Free energies (kcal/mol) computed with different functionals for Ph–Br activation at **9a** via concerted oxidative addition (COA), halogen atom abstraction (HAA) and single electron transfer (SET).

The formation of **9a** from **7a** is found to be favored with all functionals, in particular the Minnesota functionals tested and ωB97x-D. The lowest energy pathways for COA is still via **TS(12a-13c)** and so barriers for this process have all risen by the difference in energy between these two species. SET now proceeds via formation of the **9a** radical cation (Figure S22) while for HAA the formation of $\mathbf{11a}_{\mathbf{OAc}}$ is the most accessible process (Figure S23). The thermodynamics of SET and HAA are far more accessible from these anionic OAc^- complexes and as result both SET and HAA can move below COA, with SET becoming the favored pathway with certain functional (in particular, the Minnesota functionals and ωB97x-D for the reason mentioned above). It should be reiterated that only the thermodynamics of SET and HAA have been considered here and so barriers are likely to be higher. However, the lowest energy pathways computed from bis-cyclometalated **9a** are all lower in energy than those from mono-cyclometalated $\mathbf{3a}_{\mathbf{OAc}}$, and so the conclusion that reaction is preferred from the bis-cyclometalated species is independent of the model and functional employed.

12. C-H Arylation via mono-cyclometalated intermediates

A speciation study of possible mono-cyclometalated species with PhBr as a ligand was conducted (see Figure S27), including isomers of cationic species **2_{PhBr}** and **5_{PhBr}-OAc**, and neutral **5_{PhBr}** (with κ^1-OAc ligands) and **14** (with κ^2-OAc ligands). All these species were much higher in energy than **1** with **$5a_{PhBr}$** (+5.8 kcal/mol) and **14a** (+6.8 kcal/mol) being the most accessible. π-bound isomers (including η^6-**14a-OAc**) were also considered but are disfavoured.

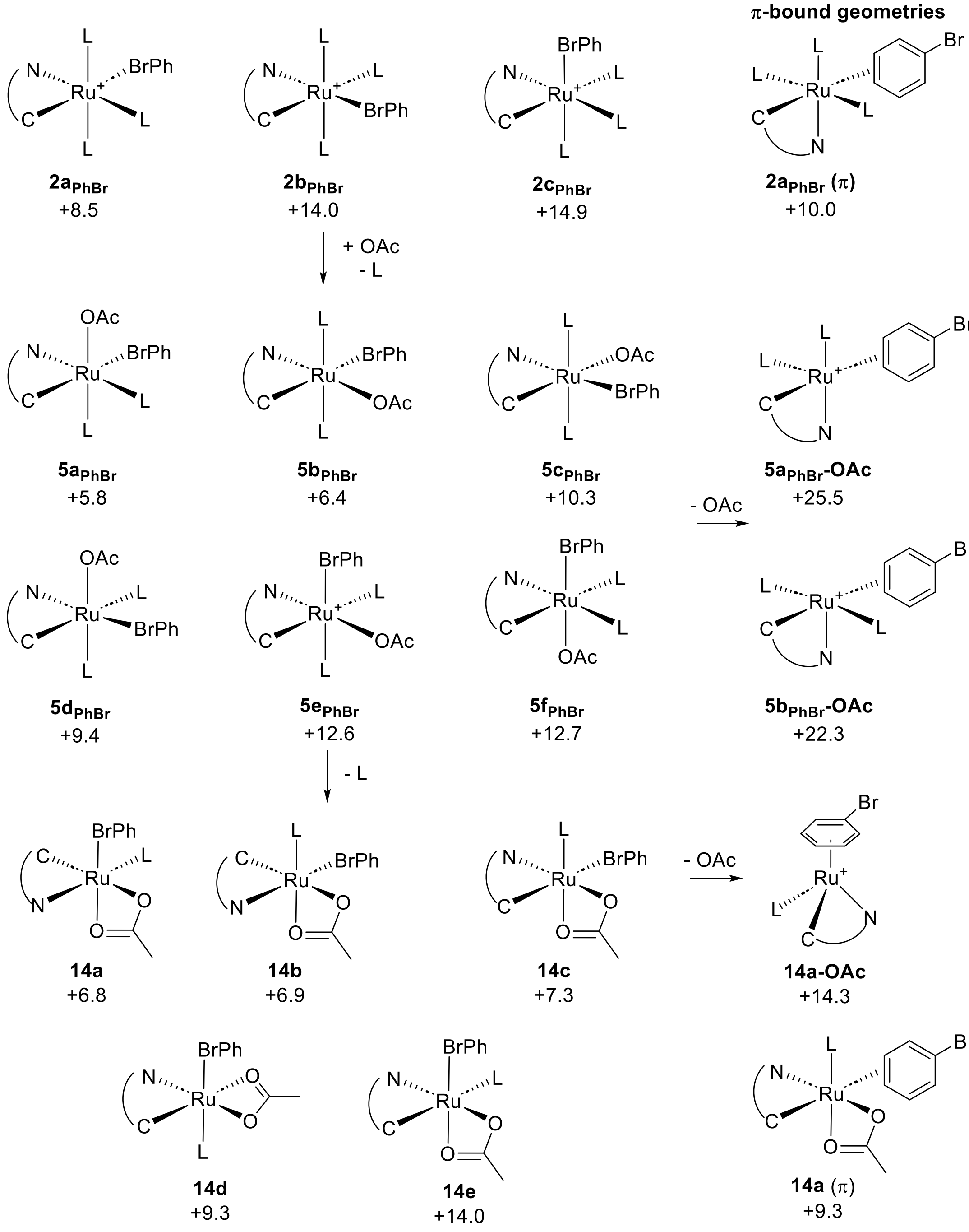

Figure S27. Computed free energies (kcal/mol) for mono-cyclometalated intermediates featuring PhBr as a ligand. Method: PBE(def2-tzvp,MeOH,D3BJ)//BP86(MeOH,SDD,6-31G**).

12.1. Ph-Br activation in the presence of OAc$^-$

12.1.1 COA in 14 to give 7-coordinate Ru(IV), 16bis.

COA in isomers of **14** would form 7-coordinate Ru(IV) intermediates, **16** for which 18 isomers were located (Figure S28). Other potential isomers rearranged to one of those shown.

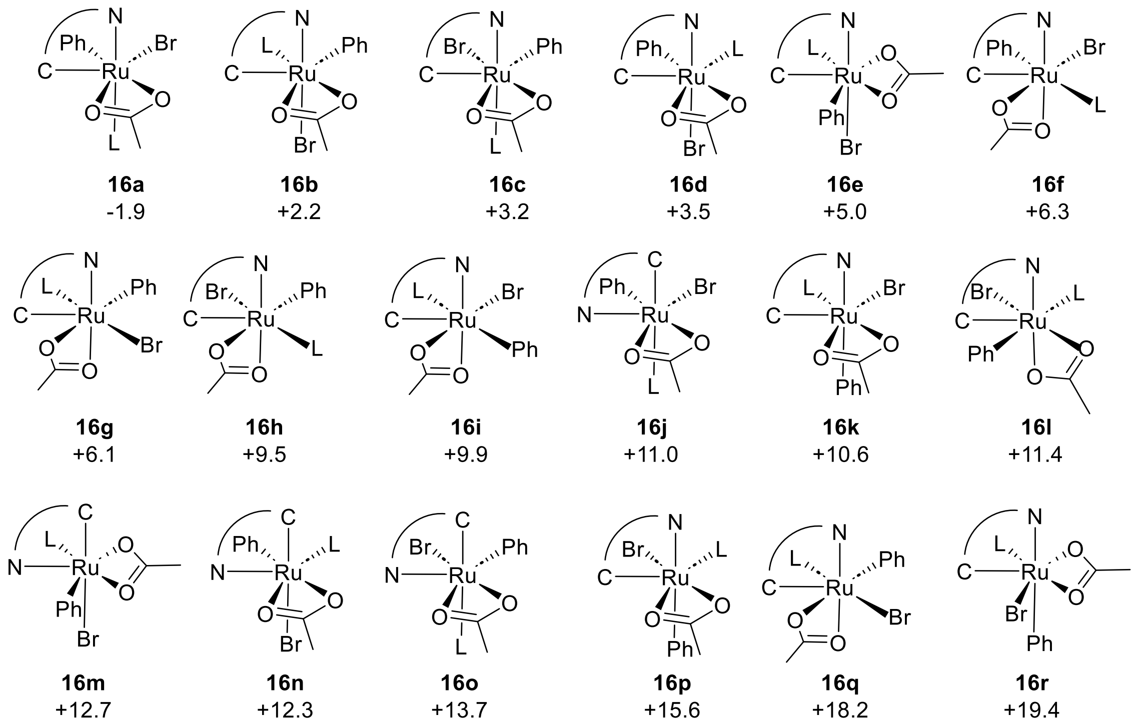

Figure S28. Isomers of **16** potentially formed via COA in **14**. Method: PBE(def2-tzvp,MeOH,D3BJ)//BP86(MeOH,SDD,6-31G**).

Transition states for Ph–Br activation (Figure S29) were then located via scans from the isomers of **16** that probed the microscopic reverse Ph–Br coupling process. The lowest energy process stems from **14b** via **TS(14b-16b)** at +17.1 kcal/mol.

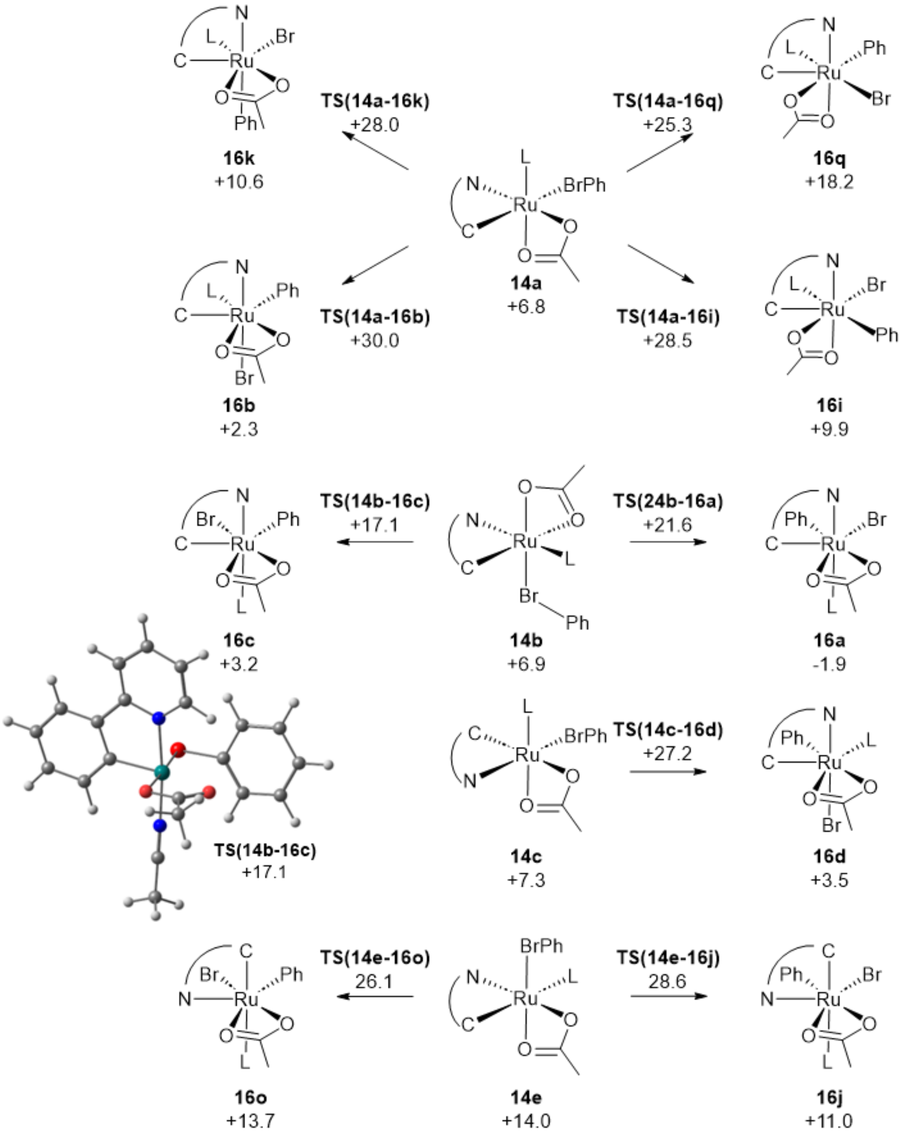

Figure S29. Computed free energies (kcal/mol) for Ph–Br activation via concerted oxidative addition, from isomers of 6-coordinate **14**, with the geometry of the most accessible transition state also shown Method: PBE(def2-tzvp,MeOH,D3BJ)//BP86(MeOH,SDD,6-31G**).

12.1.2 COA in Ru(2-ppy)(L)(κ^1-OAc)(BrPh), 24, to give 6-coordinate Ru(IV), 25.

COA in isomers of **24** would form 6-coordinate Ru(IV) intermediates, **25** for which 11 isomers are possible (Figure S30a). These were computed as both the singlet and triplet electronic states. Large singlet-triplet gaps were computed when trans C_{aryl} ligands are present that are mutually cis to Br. This allows for maximum π-stabilisation that favors the low-spin state. Transition states for Ph–Br activation (Figure S30b) were located via scans from isomers of **25** that probed the microscopic reverse Ph–Br coupling. The lowest energy process stems from **24b** via **TS(24b-25a')** at +20.6 kcal/mol.

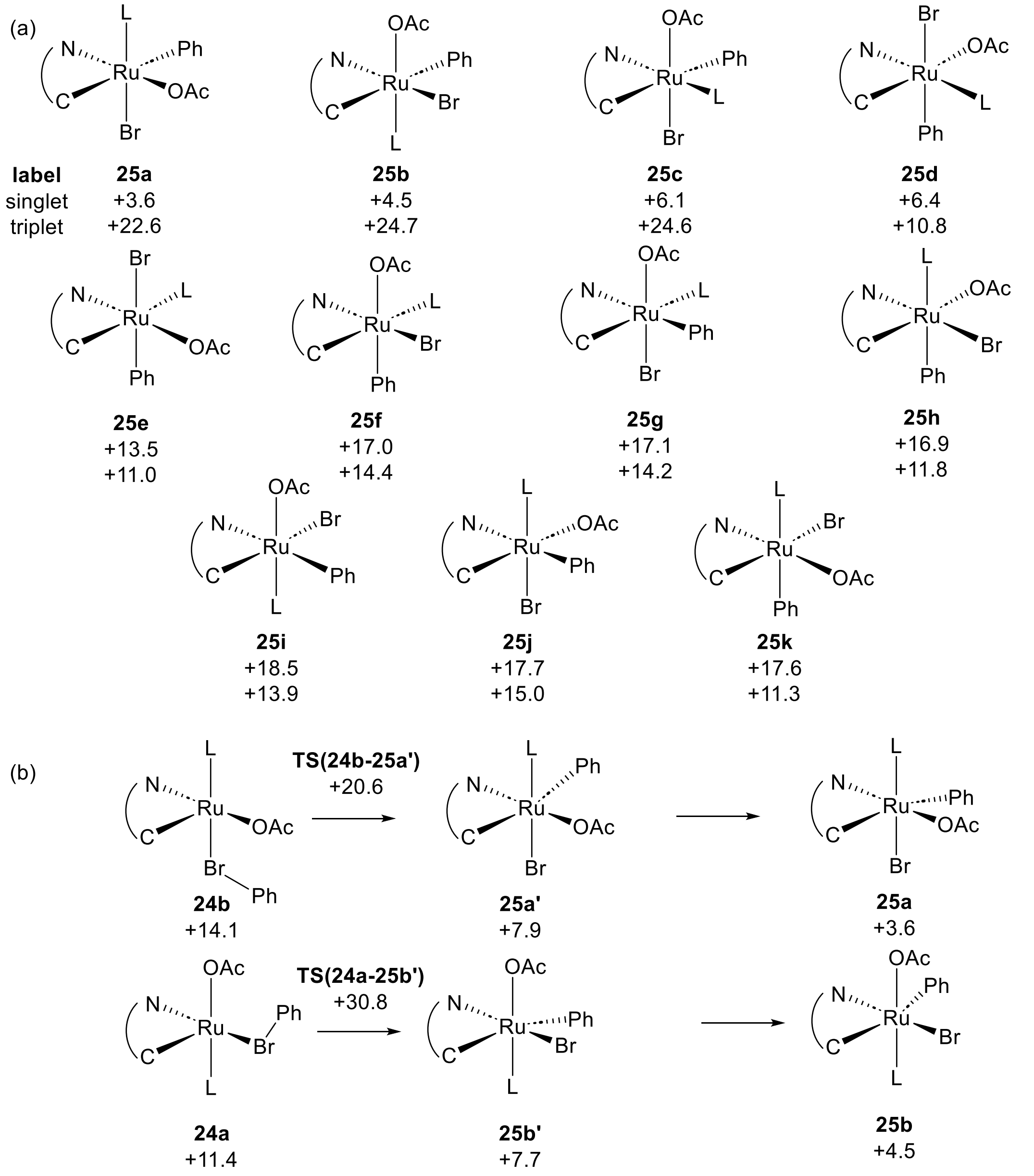

Figure S30. (a) Computed free energies (kcal/mol) for isomers of **25** formed via concerted oxidative addition of PhBr at isomers of **24**. Data for geometries optimised in both the singlet and the triplet electronic states are given. (b) Computed free energies for Ph–Br activation at isomers **24a** and **24b**.

12.1.3 COA in Ru(2-ppy)(κ^2-OAc)(BrPh), 26, to give 6-coordinate Ru(IV), 27.

COA in isomers of **26** would form 6-coordinate Ru(IV) intermediates, **27** for which 6 isomers are possible (Figure S31a). These were computed as both the singlet and triplet electronic states, with singlets again favoured by trans C_{aryl} ligands that are cis to Br. Transition states for Ph–Br activation (Figure S31b) were located via scans from isomers of **27** that probed the microscopic reverse Ph–Br coupling. The lowest energy process stems from **26a** via **TS(26a-27a')** at +22.6 kcal/mol.

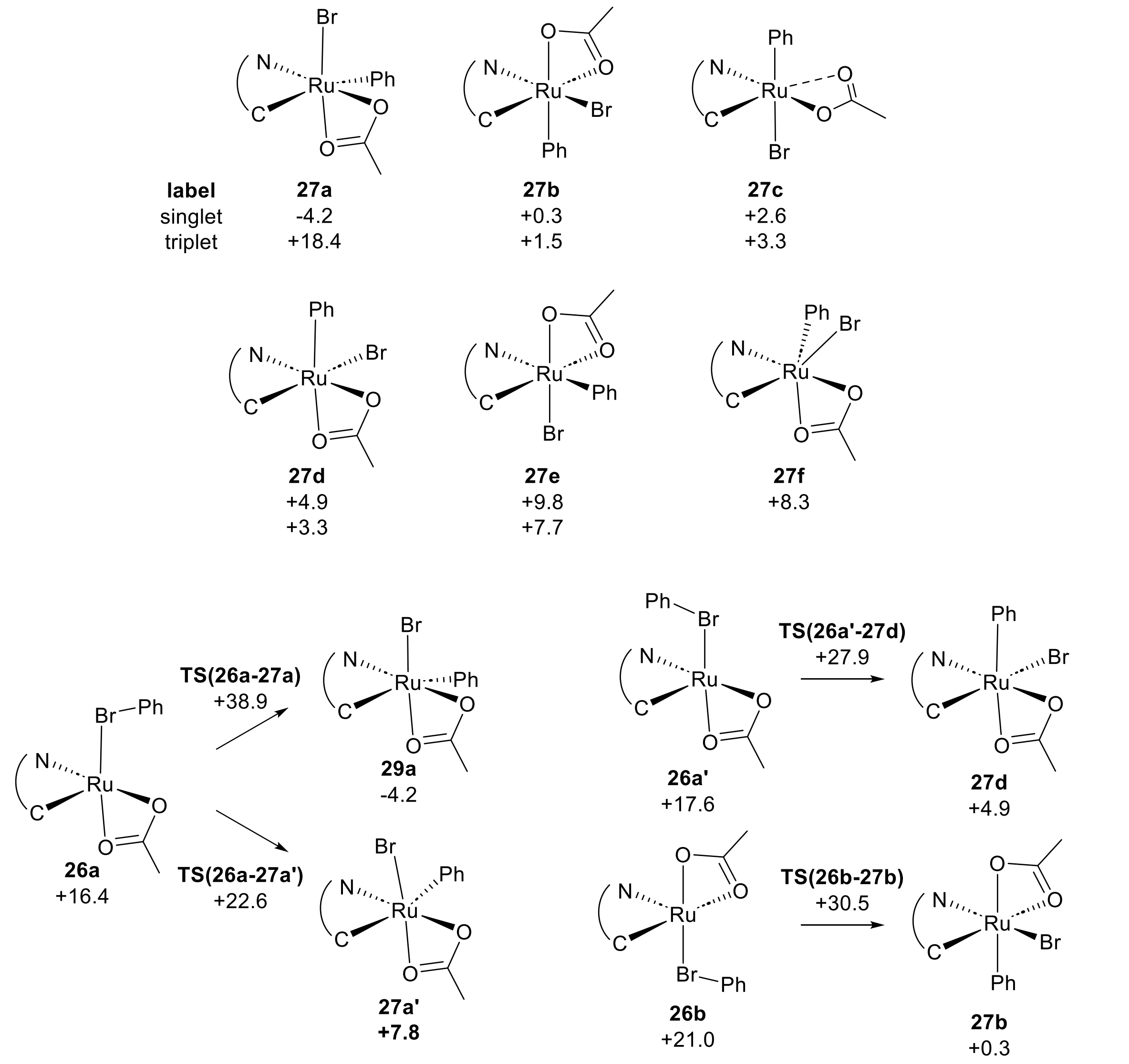

Figure S31. (a) Computed free energies (kcal/mol) for isomers of **27** formed via oxidative addition of PhBr at isomers of **26**. Data for geometries optimised in both the singlet and the triplet electronic states are given. (b) Computed free energies for Ph–Br activation at isomers **26a** and **26b**.

12.1.4. Single electron transfer (SET)

Figure S32 gives the thermodynamics of the most accessible SET processes from low energy neutral mono-cyclometalated intermediates of **2**$_{\mathbf{OAc}}$, **3**$_{\mathbf{OAc}}$, **4** and **5**, to form of the corresponding cationic Ru(III) species along with Ph· and Br$^-$ (the [PhBr]$^-$ radical anion not being a local minimum). This maximises any entropic stabilisation meaning the overall free energies in Figure S32 are a lower limit to the thermodynamics of this step.

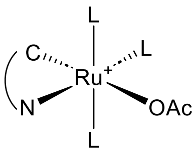

2a$_{\mathbf{OAc}}$**•**
+39.7

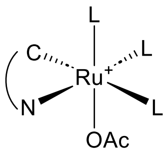

2b$_{\mathbf{OAc}}$**•**
+39.5

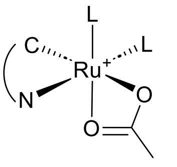

3a$_{\mathbf{OAc}}$**•**
+34.4

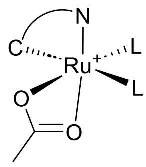

3c$_{\mathbf{OAc}}$**•**
40.7

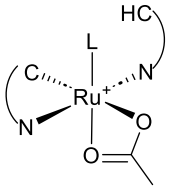

4•
+32.8

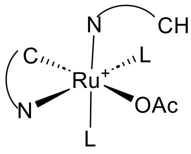

5a•
+40.9

Figure S32. Computed free energies (kcal/mol) for single electron transfer (SET) to PhBr from low energy isomers of the mono-cyclometalated intermediates **2**$_{\mathbf{OAc}}$, **3**$_{\mathbf{OAc}}$, **4** and **5** and **2a**$_{\mathbf{2\text{-}ppy}}$. Method: PBE(def2-tzvp,MeOH,D3BJ)//BP86(MeOH,SDD,6-31G**) .

12.1.5. Halogen atom abstraction (HAA)

Figure S33 gives the thermodynamics of HAA from neutral precursors **2_{PhBr}**, **5_{PhBr}** and **14** to form the corresponding Ru(III) species and a phenyl radical. These product species are computed separately, maximising any entropic stabilisation and hence providing a lower limit to the thermodynamics of this step.

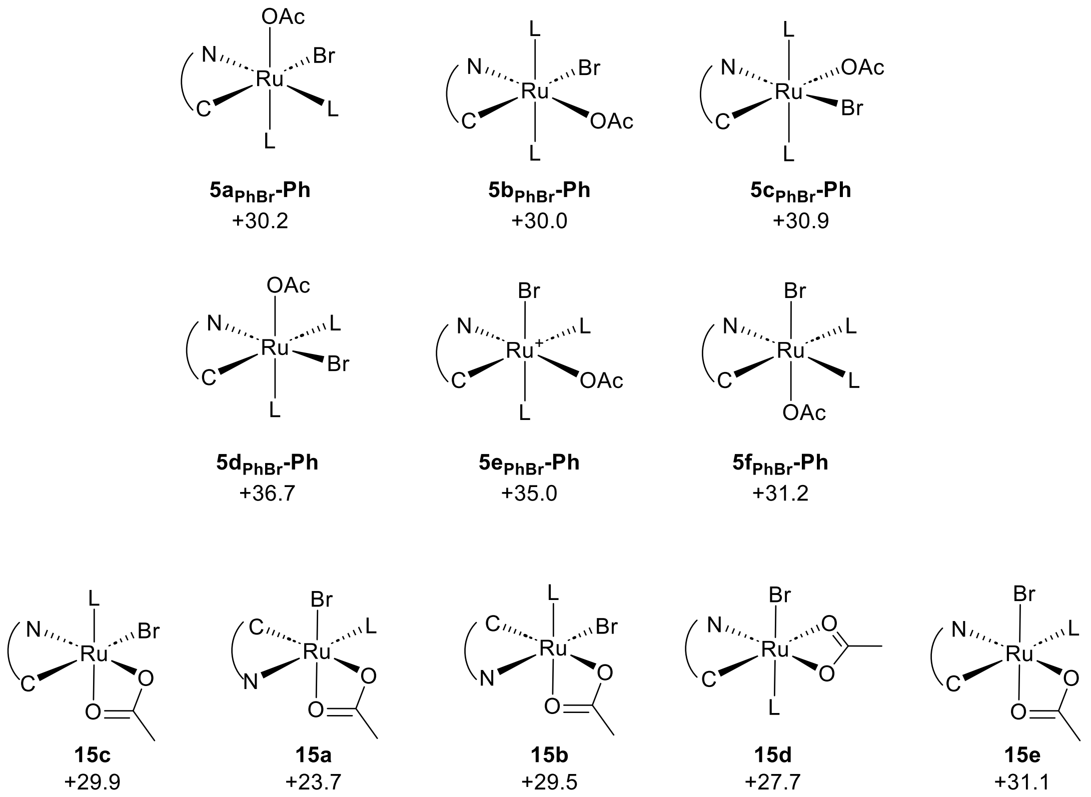

Figure S33. Computed free energies (kcal/mol) for halogen atom abstraction (HAA) from isomers of **2_{PhBr}**, **5_{PhBr}** and **14** to form the corresponding Ru(III) species and a phenyl radical. Method: PBE(def2-tzvp,MeOH,D3BJ)//BP86(MeOH,SDD,6-31G**).

12.2. Ph-Br activation in the absence of OAc⁻

12.2.1. COA in 2_{PhBr} to give 7-coordinate Ru(IV), 22.

COA in isomers of **2**$_{\mathbf{PhBr}}$ would form 7-coordinate Ru(IV) intermediates, **22** (Figure S34). Geometries of **22** were initiated from a series of pentagonal bipyramidal geometries with the cyclometalated 2-ppy ligand occupying equatorial sites. Isomerisation to structures with N_{py} occupying an axial site was generally seen, although in some cases with Ph cis to C_{aryl} C–C coupling was seen during the optimisation. 6 isomers of **22** were located but the high energy of these Ru(IV) species precludes their involvement in a competitive mechanism. For completeness one transition state for Ph–Br activation was located in **2b**$_{\mathbf{PhBr}}$, for which a free energy of 38.5 kcal/mol was determined.

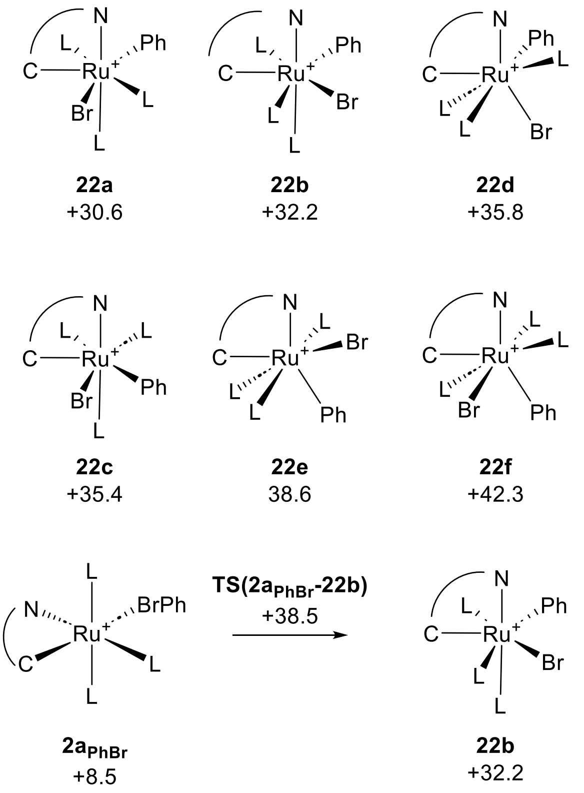

Figure S34. Computed free energies (kcal/mol) for isomers of 7-coordinate **22** formed via oxidative addition of PhBr at isomer of **2**$_{\mathbf{PhBr}}$. Method: PBE(def2-tzvp,MeOH,D3BJ)//BP86(MeOH,SDD,6-31G**).

12.2.2 COA in Ru(2-ppy)(L)$_2$(BrPh), 22, to give 6-coordinate Ru(IV), 23.

COA in isomers of **22** would form 6-coordinate Ru(IV) intermediates, **23** for which 7 isomers are possible (Figure S35a). These were computed as both the singlet and triplet electronic states with singlets favored by trans C_{aryl} ligands that are mutually cis to Br. Transition states for Ph–Br activation (Figure S35b) were located via scans from isomers of **23** that probed the microscopic reverse Ph–Br coupling. The lowest energy of these proceeded from **22a** via a transition state at +38.2 kcal/mol.

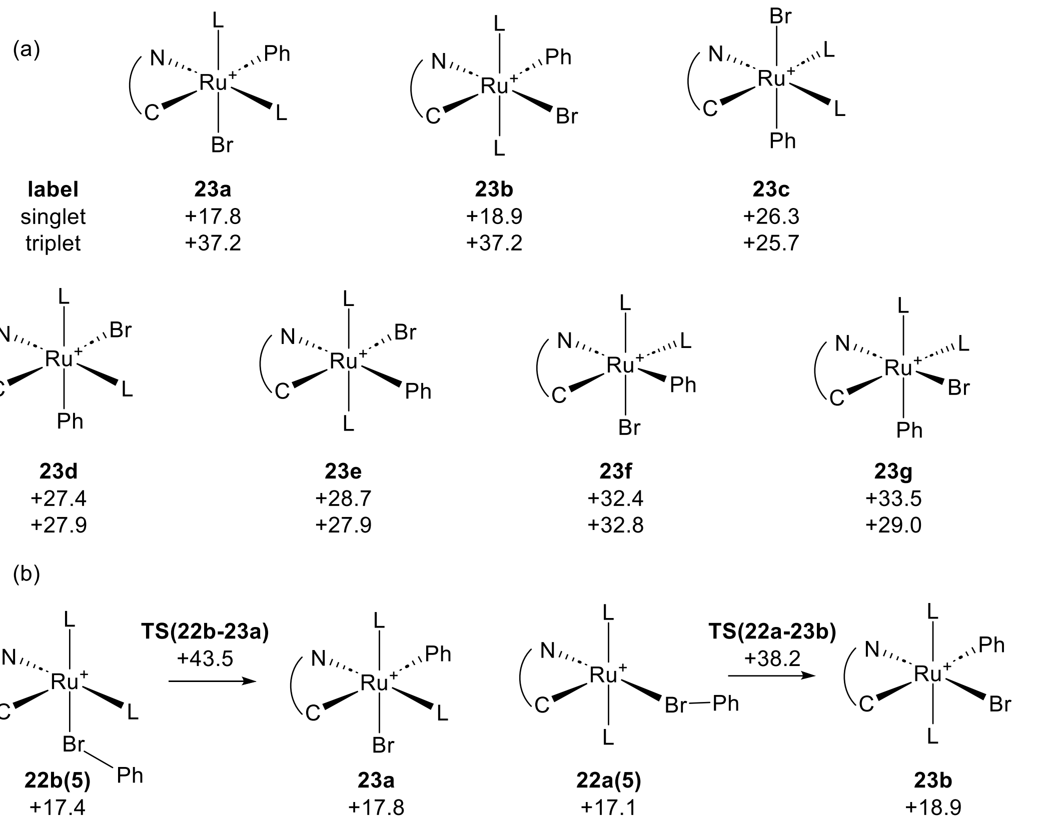

Figure S35. (a) Computed free energies (kcal/mol) for isomers of 6-coordinate **23** formed via concerted oxidative addition of PhBr at isomers of **22**, computed in both the singlet and the triplet electronic states. (b) Computed free energies for Ph–Br activation at isomers **22a** and **22b**. Method: PBE(def2-tzvp,MeOH,D3BJ)//BP86(MeOH,SDD,6-31G**).

12.2.3 Lowest energy SET and HAA processes.

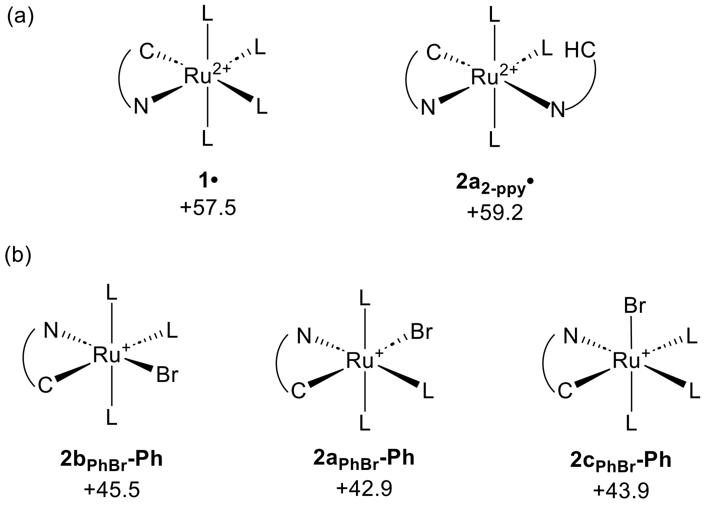

Figure S36. Computed free energies (kcal/mol) for (a) single electron transfer (SET) to PhBr from the lowest energy cationic precursors, **1** and **2a$_{2\text{-ppy-H}}$** and (b) HAA from isomers of **2$_{PhBr}$**. Method: PBE(def2-tzvp,MeOH,D3BJ)//BP86(MeOH,SDD,6-31G**).

The lowest energy pathways established for Ph–Br activation at bis-cyclometalated intermediates (COA at **7a** via **TS(12a-13c)**) and via a mono-cyclometalated intermediate (COA from $\mathbf{3a_{OAc}}$ via **TS(14b-16c)**) were recomputed with different functionals (Figure S37). In each case a clear preference for reaction via bis-cyclometalated species is seen.

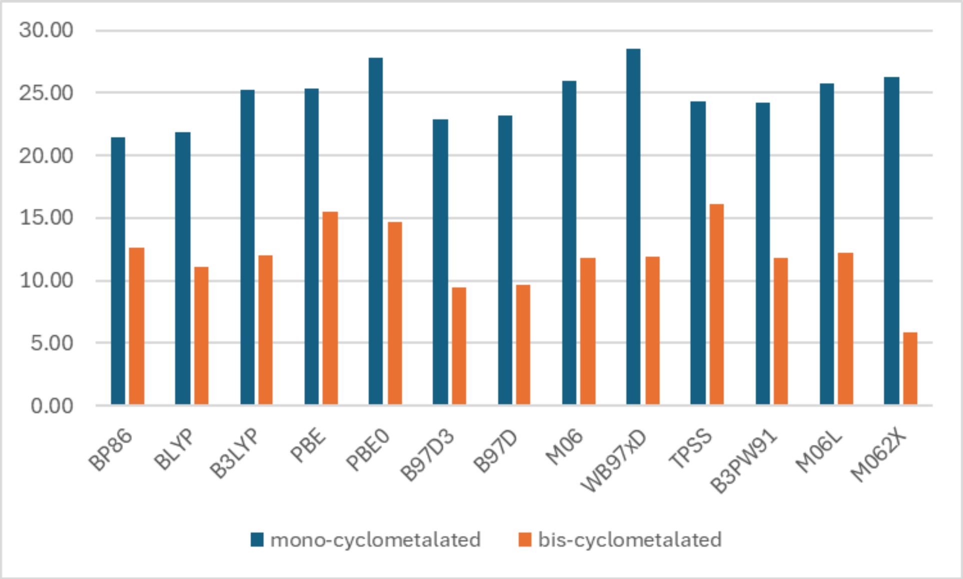

Figure S37. Free energies (kcal/mol) computed with different functionals for the lowest energy pathways for Ph–Br activation at bis-cyclometalated **7a** (COA via **TS(12a-13c)**) and via mono-cyclometalated 3aOAc (COA via **TS(14b-16c)**).

The lowest energy pathways computed from bis-cyclometalated **9a** are all lower in energy than those from mono-cyclometalated **3a**$_{\mathbf{OAc}}$, and so the conclusion that reaction is preferred from the bis-cyclometalated species is independent of the model and functional employed.

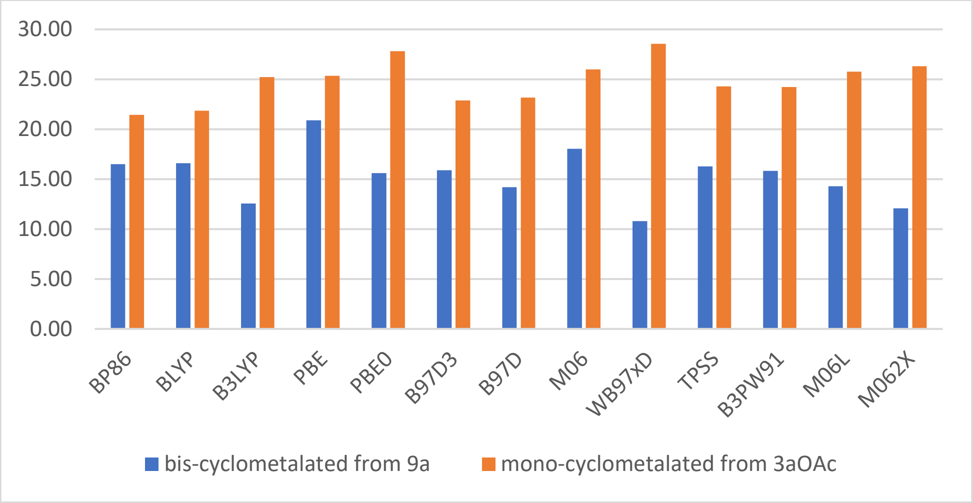

Figure S38. Free energies (kcal/mol) computed with different functionals for the lowest energy pathways for Ph–Br activation at bis-cyclometalated **9a** (see Figure S26 and discussion for the preferred pathways) and via mono-cyclometalated **3a**$_{\mathbf{OAc}}$ (COA via **TS(14b-16c)**).

13. C–C coupling and product release.

C–C coupling in **13a** forms **17a**$_{\pi}$, from which product release requires (i) displacement of the π-interaction of the arene ring of the 2-ppy-Ph product and (ii) addition of sufficient L and/or OAc^{-} ligands to form 6-coordinate intermediates from which the **2-ppy-Ph** product can dissociate. Ligand addition may occur either with or without Br^{-} loss.

In exploring the many possible pathways encompassing these processes two general principles were adopted: (i) the new **2-ppy-H** substrate required in the next catalytic cycle only binds after the **2-ppy-Py** product has dissociated (ii) the mer-arrangement of the {Ru(2-ppy)(2-ppy-Ph)} core with trans-N ligands and (when present) Br cis to the Ru-aryl is retained throughout. With this approach seven 6-coordinate intermediates with the product bound could be identified, as shown Figure S39.

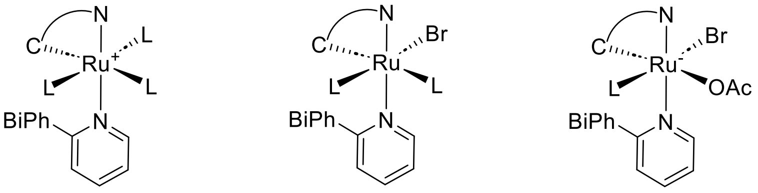

	**2c$_{2\text{-ppy-Ph}}$**	**24a**	**29a**
	-26.8	-26.1	-25.6
**$\Delta G^{\ddagger}_{span}$**	24.1	26.3	26.6

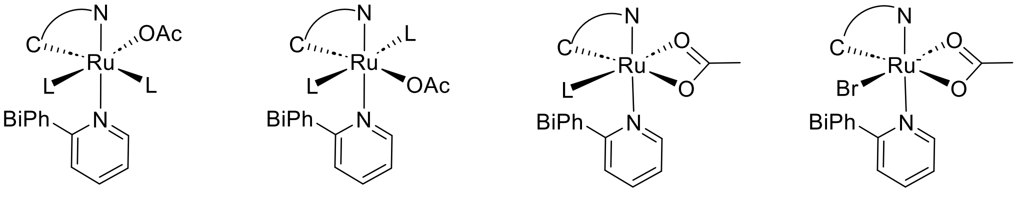

	**5f^{Ph}**	**5b^{Ph}**	**4a^{Ph}**	**31a**
	-27.7	-31.6	-38.3	-23.4
**$\Delta G^{\ddagger}_{span}$**	27.9	25.3	18.5	14.9

Figure S39. Computed free energies (kcal/mol) for complexes featuring the 2-ppy-Ph product bound through the pyridyl nitrogen. Method: PBE(def2-tzvp,MeOH,D3BJ)//BP86(MeOH,SDD,6-31G**).

Figure S39 also shows the overall free energy span, $\Delta G^{\ddagger}_{span}$, required to access each intermediate and full details of the pathways involved are given in Figures S40-45. The kinetically most accessible intermediate is **31a** with $\Delta G^{\ddagger}_{span}$ = 14.9 kcal/mol. However, loss of Br from **31a** then entails energy spans in excess of 22 kcal/mol (see Figure S44-S45). Therefore, the lowest energy pathway that leads directly back into the catalytic cycle is via **4a^{Ph}** with a barrier of 18.5 kcal/mol. Details of the lowest energy process are given in Figures S40 and S42; these are the same as Figures 5A and 5B in the main text, but with the transition state geometries also shown.

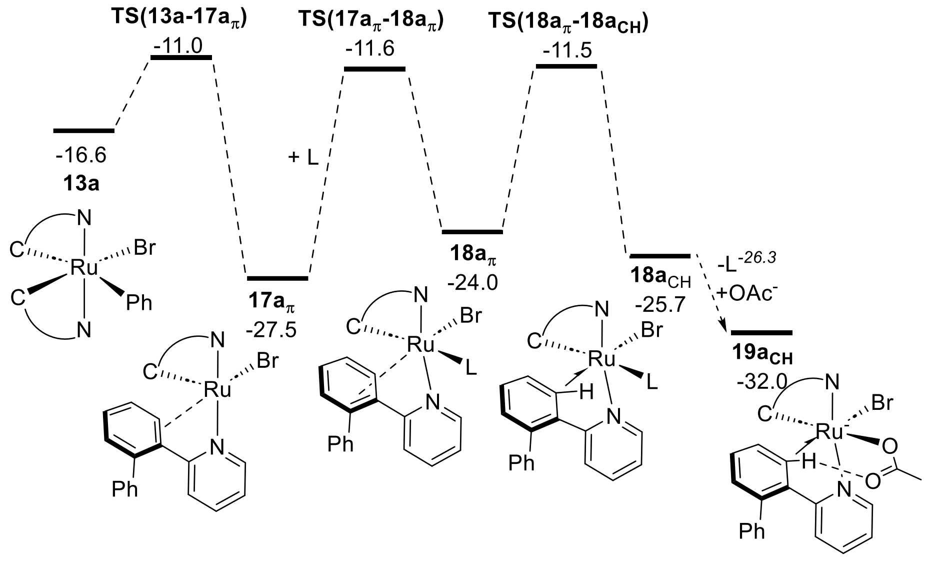

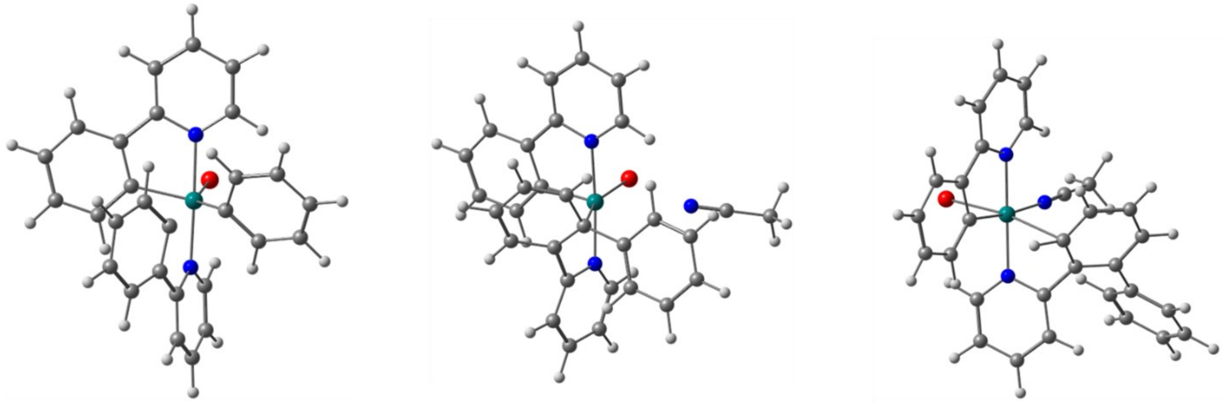

TS(13a-17a$_\pi$)
-11.0

TS(17a$_\pi$-18a$_\pi$)
-11.6

TS(18a$_\pi$-18a$_{CH}$)
-11.5

Figure S40. Computed free energies (kcal/mol) for C–C coupling in **13a** and formation of **19a$_{CH}$** with geometries of transition states also shown. Method: PBE(def2-tzvp,MeOH,D3BJ)//BP86(MeOH,SDD,6-31G**).

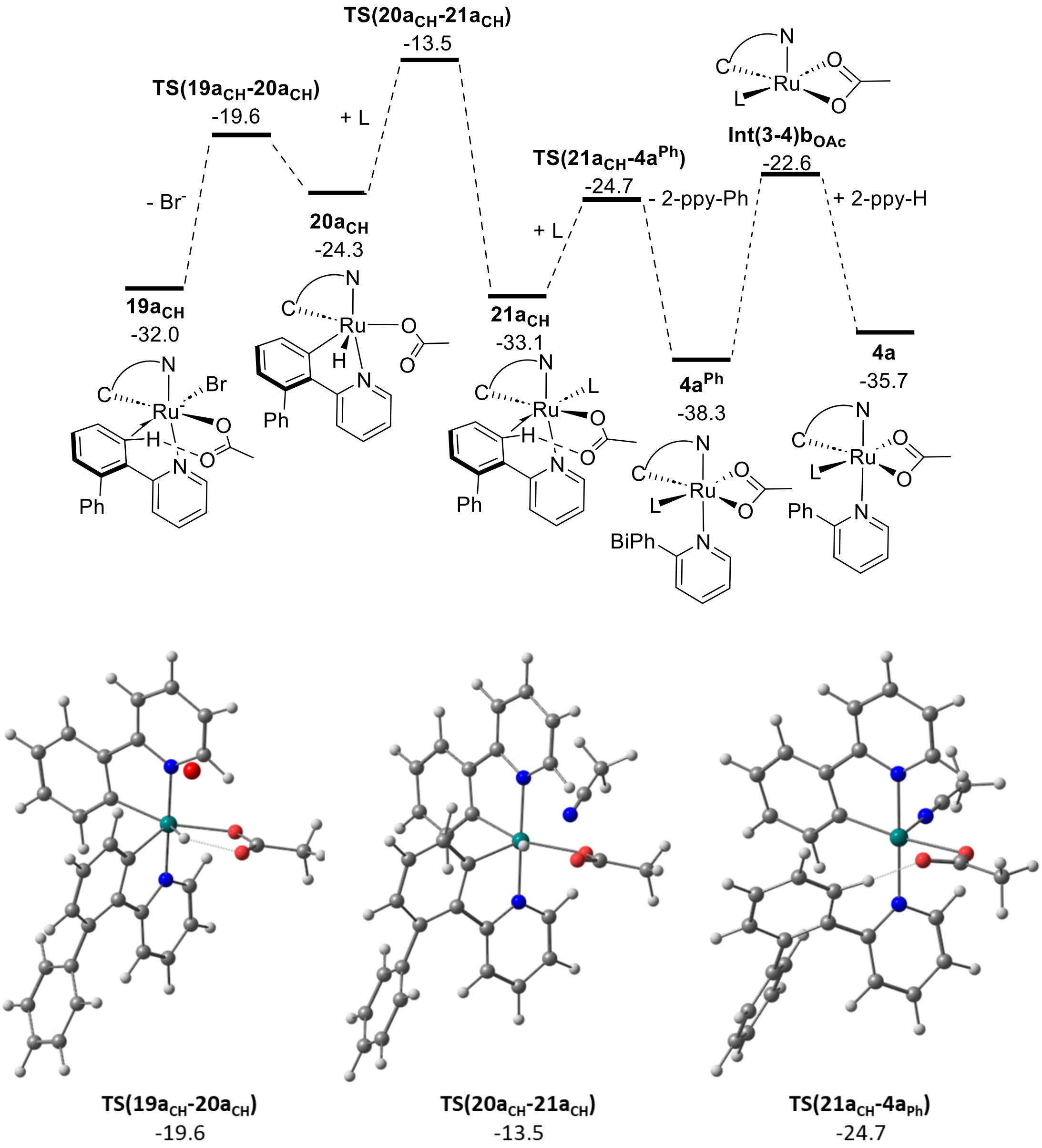

Figure S41. Computed free energies (kcal/mol) for release of the **2-ppy-Ph** product from 19aCH and reformation of **4a** with geometries of transition states also shown. Method: PBE(def2-tzvp,MeOH,D3-BJ)//BP86(MeOH,SDD,6-31G**). Method: PBE(def2-tzvp,MeOH,D3BJ)//BP86(MeOH,SDD,6-31G**)./

13.1. Initial reactions of $17a_\pi$

The reactions of **$17a_\pi$** were assessed and are summarized in Figure S40.

(i) Rearrangement gives C–H agostic isomers **$17a_{CH}$** and **$17a'_{CH}$** in which the C–H agostic bond lies either parallel or anti-parallel to the Ru–C_{aryl} bond. Free energy spans for the formation of these species are 8.4 kcal/mol and 18.4 kcal/mol respectively.

(ii) Bromide loss to form **$17a_\pi$-Br** is relatively high in energy (ΔG = +24.2 kcal/mol).

(iii) Addition of L gives **$18a_\pi$** which can then rearrange first to **$18a'_{CH}$** and then **$18a_{CH}$** with overall energy spans of 15.9 kcal/mol and 16.0 kcal/mol respectively. L loss from **$18a_{CH}$** therefore provides an alternative route to **$17a'_{CH}$**.

(iv) OAc^- addition gives **$26a_\pi$** at -20.4 kcal/mol from which Br^- dissociation gives **$27a_\pi$** with an energy span of 20.8 kcal/mol.

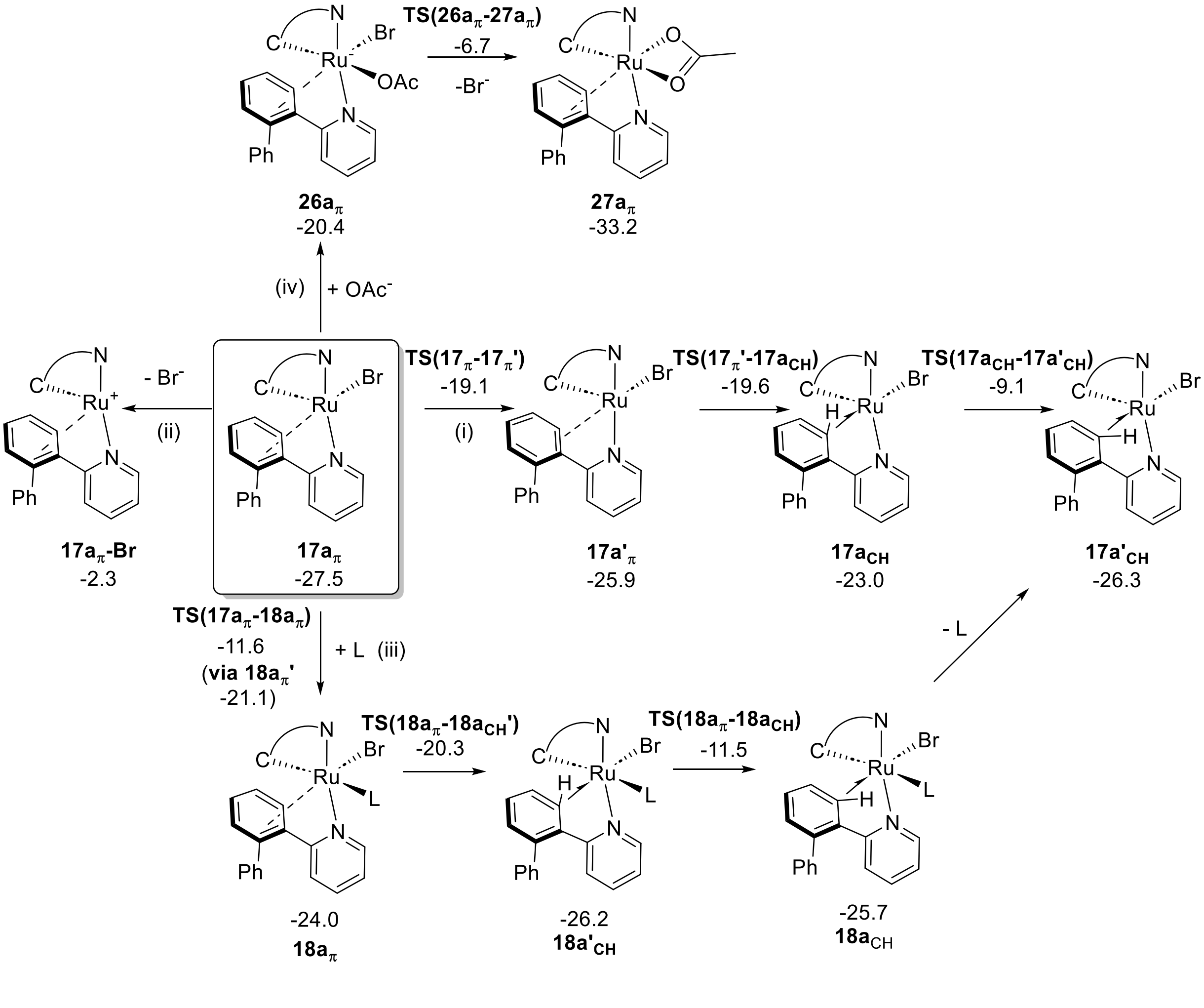

Figure S42. Computed free energies (kcal/mol) for initial reactivity of **$17a_\pi$**. Method: PBE(def2-tzvp,MeOH,D3BJ)//BP86(MeOH,SDD,6-31G**).

13.2. Product release mechanisms

The lowest energy pathways located for the formation of κ-N-**2-ppy-Ph** adducts highlighted in Figure S39 are shown in subsequent subsections.

13.2.1. Product release via $\mathbf{2c_{2\text{-}ppy\text{-}Ph}}$ and 24a from common intermediate $\mathbf{18a'_{CH}}$ (Figure S41)

24a: displacement of the C–H agostic interaction in $\mathbf{18a'_{CH}}$ by MeCN gives **24a** directly via a transition state at -1.2 kcal/mol. Accessing **24a** proceeds with $\Delta G^{\ddagger}_{span}$ = 26.3 kcal/mol relative to $\mathbf{27a_{\pi}}$ at -27.5 kcal/mol. **2-ppy-Ph** dissociation then forms **25a** at -15.9 kcal/mol.

$\mathbf{2c_{2\text{-}ppy\text{-}Ph}}$: Br^- loss gives $\mathbf{22a_{CH}}$ to which addition of two MeCN ligands gives $\mathbf{2c_{2\text{-}ppy\text{-}Ph}}$. The second of these involves displacement of the C–H agostic interaction via $\mathbf{TS(23_{CH}\text{-}2c_{2\text{-}ppy\text{-}Ph})}$ at -3.4 kcal/mol. Accessing $\mathbf{2c_{2\text{-}ppy\text{-}Ph}}$ proceeds with $\Delta G^{\ddagger}_{span}$ = 24.1 kcal/mol relative to $\mathbf{27a_{\pi}}$ at -27.5 kcal/mol. **2-ppy-Ph** dissociation then forms **Int(1-2)b** at -14.5 kcal/mol.

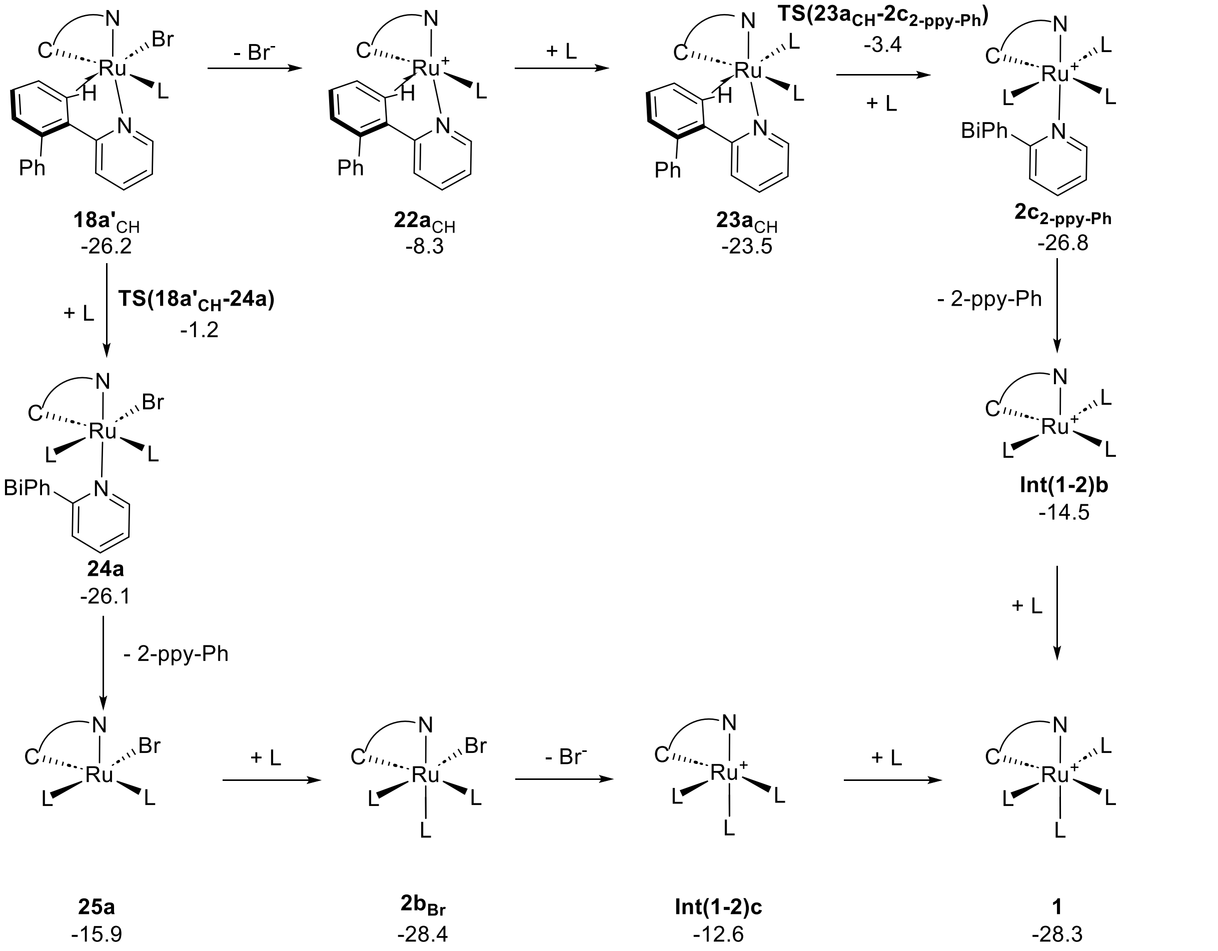

Figure S43. Lowest free energy pathways (kcal/mol) for the formation of $\mathbf{2c_{2\text{-}ppy\text{-}Ph}}$ and **24a** from $\mathbf{17a_{\pi}}$ via common intermediate $\mathbf{18a'_{CH}}$. Method: PBE(def2-tzvp,MeOH,D3BJ)//BP86(MeOH,SDD,6-31G**).

13.2.2. Product release via $5b^{Ph}$ and $4a^{Ph}$ from common intermediate $21a_{CH}$ (Figure S42)

(i) Formation of **$21a_{CH}$**: OAc^- addition to **$17a'_{CH}$** followed by Br^-/L substitution gives **$21a_{CH}$** and proceeds with $\Delta G^{\ddagger}_{span}$ = 18.5 kcal/mol relative to relative to **$19a_{CH}$** at -32.0 kcal/mol.

(ii) Displacement of the C–H agostic interaction in **$21a_{CH}$** by L then gives **$5b^{Ph}$** which can lose **2-ppy-Ph** to give **$3b_{OAc}$** directly. This process has an energy span of 25.2 kcal/mol.

(iii) Alternatively, displacement of the C–H agostic interaction by the OAc^- ligand reverting to a κ^2-binding mode gives **$4a^{Ph}$** with a much lower barrier of only 8.4 kcal/mol. The overall energy span to form **$4a^{Ph}$** via this route is the 18.5 kcal/mol associated with Br-/L substitution in **$17a'_{CH}$**.

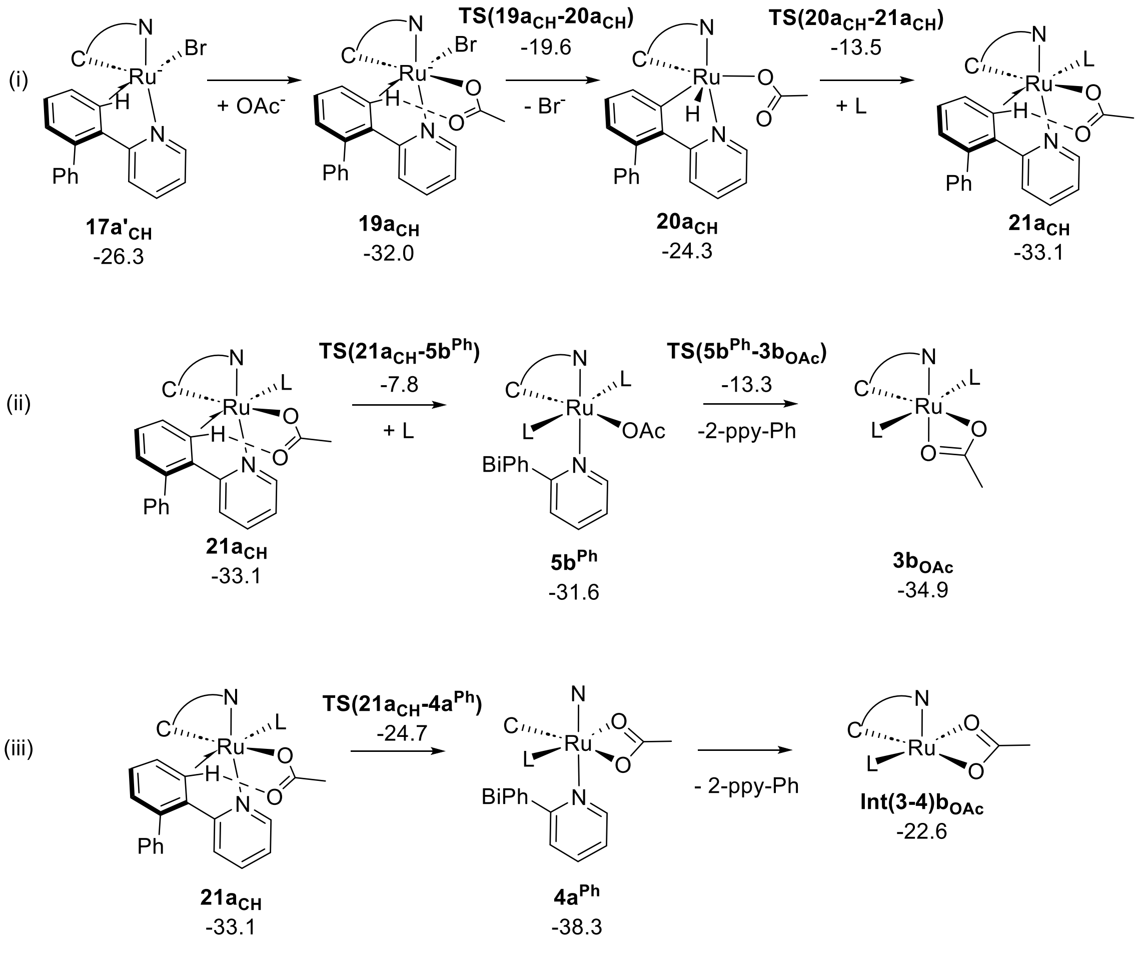

Figure S44. Lowest free energy pathway (kcal/mol) for the formation of **$5b^{Ph}$** and one pathway to form **$4a^{Ph}$** from **$17a_{\pi}$** via common intermediate **$21a_{CH}$**. Method: PBE(def2-tzvp,MeOH,D3BJ)//BP86(MeOH,SDD,6-31G**).

13.2.3. Product release via $\mathbf{5f^{Ph}}$ or 29a from $\mathbf{17a_\pi}$ from common intermediate $\mathbf{26a_\pi}$ (Figure S43)

(i) via $\mathbf{5f^{Ph}}$: OAc^- addition to $\mathbf{17a_\pi}$ forms $\mathbf{26a_\pi}$ at -20.4 kcal/mol, with Br^- displacement leading to κ^2-OAc species $\mathbf{27a_\pi}$ at -33.2 kcal/mol. This has $\Delta G^{\ddagger}_{span}$ = 20.8 kcal/mol. Addition of two ligands L then gives $\mathbf{5f^{Ph}}$, with the second addition involving a transition state at -5.3 kcal/mol. **2-ppy-Ph** loss then forms $\mathbf{3c_{OAc}}$ directly. $\Delta G^{\ddagger}_{span}$ for this process is 27.9 kcal/mol, i.e. from $\mathbf{27a_\pi}$ to $\mathbf{TS(28a_{CH}\text{-}5f^{Ph})}$, assuming facile π- to C–H rearrangement in **28a**.

(ii) alternatively $\mathbf{26a_\pi}$ can give **29a** directly via a transition state at -0.9 kcal/mol. 2-ppy-Ph loss forms **30a** from which Br^- dissociation gives $\mathbf{Int(3\text{-}4)c_{OAc}}$. This process has $\Delta G^{\ddagger}_{span}$ = 26.6 kcal/mol relative to $\mathbf{27a_\pi}$ at -27.5 kcal/mol.

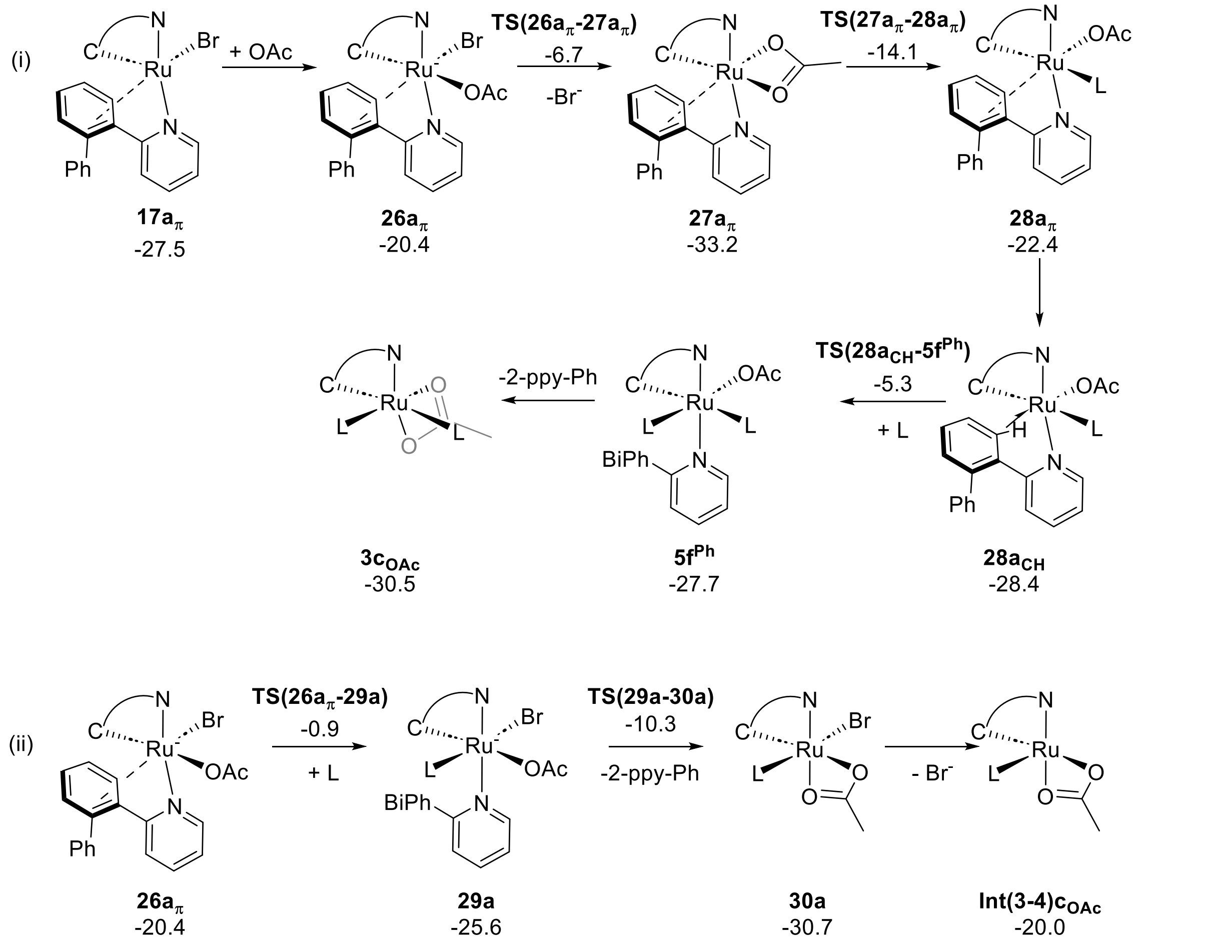

Figure S45. Lowest free energy pathways (kcal/mol) for the formation of $\mathbf{5f^{Ph}}$ and **29a** from $\mathbf{17a_\pi}$ via common intermediate $\mathbf{26a_\pi}$. Method: PBE(def2-tzvp,MeOH,D3BJ)//BP86(MeOH,SDD,6-31G**).

13.2.4. Product release via 31a from 19a$_{CH}$ (Figure S44)

Formation of **31a**: Addition of OAc^{-} to **17a'$_{CH}$** followed by displacement of the C–H agostic by the formation of κ^2-OAc complex **31a** proceeds with $\Delta G^{\ddagger}_{span}$ = 14.9 kcal/mol relative to **19a$_{CH}$**. Br^{-} dissociation gives **27a$_{\pi}$** at which MeCN addition displaces the π-bound arene via **TS(27a$_{\pi}$-4a^{Ph})** at -10.2 kcal/mol. $\Delta G^{\ddagger}_{span}$ for the formation of **4a^{Ph}** via this route is 23.0 kcal/mol.

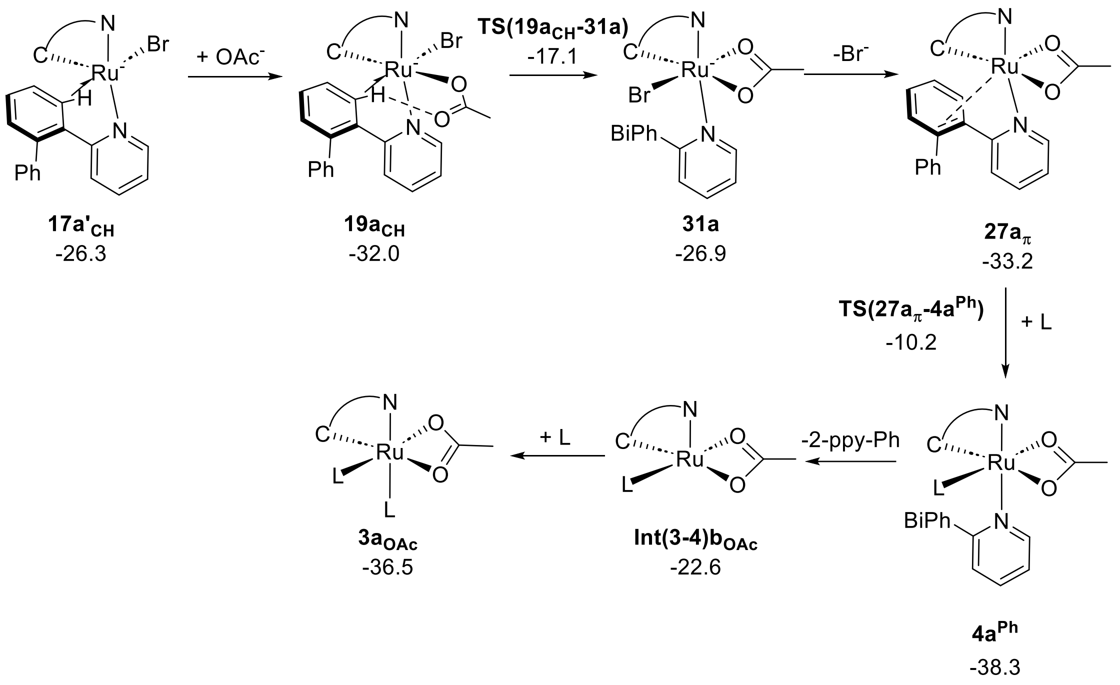

Figure S46. Lowest free energy pathway (kcal/mol) for the formation of **31a** from **17a$_{\pi}$** via **19a$_{CH}$** and onward reactions to **4a^{Ph}**. Method: PBE(def2-tzvp,MeOH,D3BJ)//BP86(MeOH,SDD,6-31G**).

13.2.5. Bromide loss after product release direct from 31a (Figure S45)

(i) Dissociation of 2-ppy-Ph from **31a** gives **32a** at -18.5 kcal/mol from which Br^- must be lost to re-enter the catalytic cycle. **32a** can add two ligands L where the second entails κ^2- κ^1-displacement of OAc^- via **TS(33a-34a)** at -3.0 kcal/mol. Displacement of Br^- by OAc^- reverting to a κ^2-binding mode gives **$3a_{OAc}$**. No transition state could be located for this step, but the $\Delta G^{\ddagger}_{span}$ to form **$3a_{OAc}$** by this route is already 30.2 kcal/mol via **TS(33a-34a)** relative to **$27a_{\pi}$** at -33.2 kcal/mol (see Figure S44).

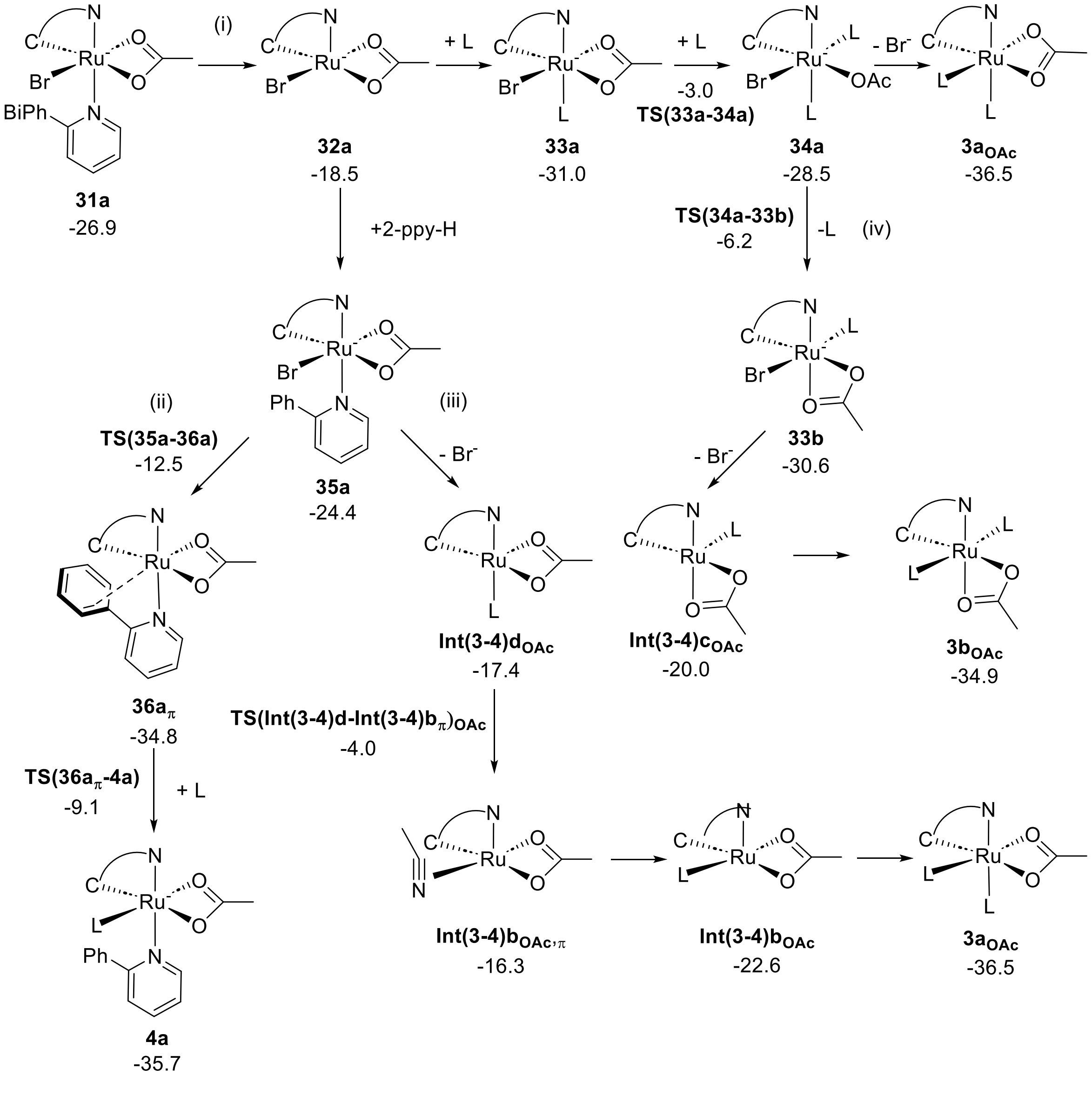

Figure S47. Lowest free energy pathway (kcal/mol) for the formation of **31a** from **$17a_{\pi}$** via **$19a_{CH}$** and onward reactions to **$4a^{Ph}$**. Method: PBE(def2-tzvp,MeOH,D3BJ)//BP86(MeOH,SDD,6-31G**).

Alternatively, 2-ppy-H adds to **32a** to give **35a** at -24.4 kcal/mol from which:

(ii) Stepwise Br^-/L substitution via **36a**$_\pi$ then gives **4a** at -35.7 kcal/mol. This has $\Delta G^{\ddagger}_{span}$ = 25.7 kcal/mol via **TS(36a$_\pi$-4a)** relative to **36a**$_\pi$.

(iii) Br^- dissociation from **35a** gives **Int(3-4)d**$_{\mathbf{OAc}}$ at -17.4 kcal/mol. Isomerisation proceeds via a transition state at -4.0 kcal/mol to an isomer of **Int(3-4)b**$_{\mathbf{OAc}}$ with a π-bound MeCN ligand. Isomerisation gives **Int(3-4)b**$_{\mathbf{OAc}}$ which adds MeCN to give **3a**$_{\mathbf{OAc}}$ at -36.5 kcal/mol. $\Delta G^{\ddagger}_{span}$ = 29.2 kcal/mol via **TS(Int(3-4)d-Int(3-4)b$_\pi$)$_{\mathbf{OAc}}$** at -4.0 kcal/mol relative to **27a**$_\pi$ at -33.2 kcal/mol.

Finally,

(iv) Loss of L from **34a** with OAc reverting to a κ^2-binding mode gives **33b** at -30.6 kcal/mol. Br^-/L substitution via **Int(3-4)c**$_{\mathbf{OAc}}$ gives **3b**$_{\mathbf{OAc}}$ at -34.9 kcal/mol. $\Delta G^{\ddagger}_{span}$ = 27.0 kcal/mol via **TS(34a-33b)** at -6.2 kcal/mol relative to **27a**$_\pi$ at -33.2 kcal/mol.

14. Calculations with Model 2.

14.1 C-H Activation

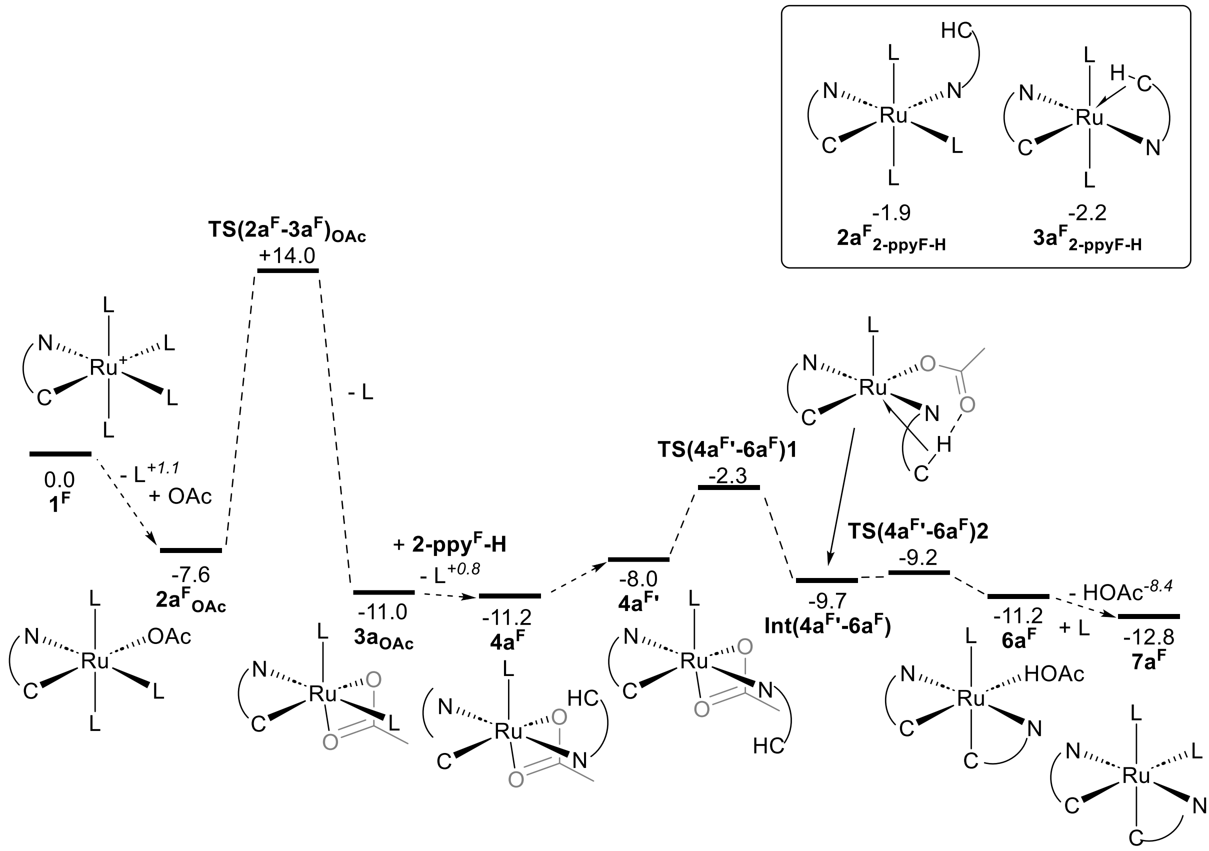

Figure S48. Computed free energy profile (kcal/mol, L = MeCN) for C–H activation of **2-ppy^F-H** at **1^F** to form bis-cyclometalated species **7a^F**. Insert: low energy species computed upon reaction of **1^F** with **2-ppy^F-H** in the absence fo OAc$^-$. Method: PBE(def2-tzvp,MeOH,D3BJ)// BP86(MeOH,SDD,6-31G**).

14.2 C-Br Activation

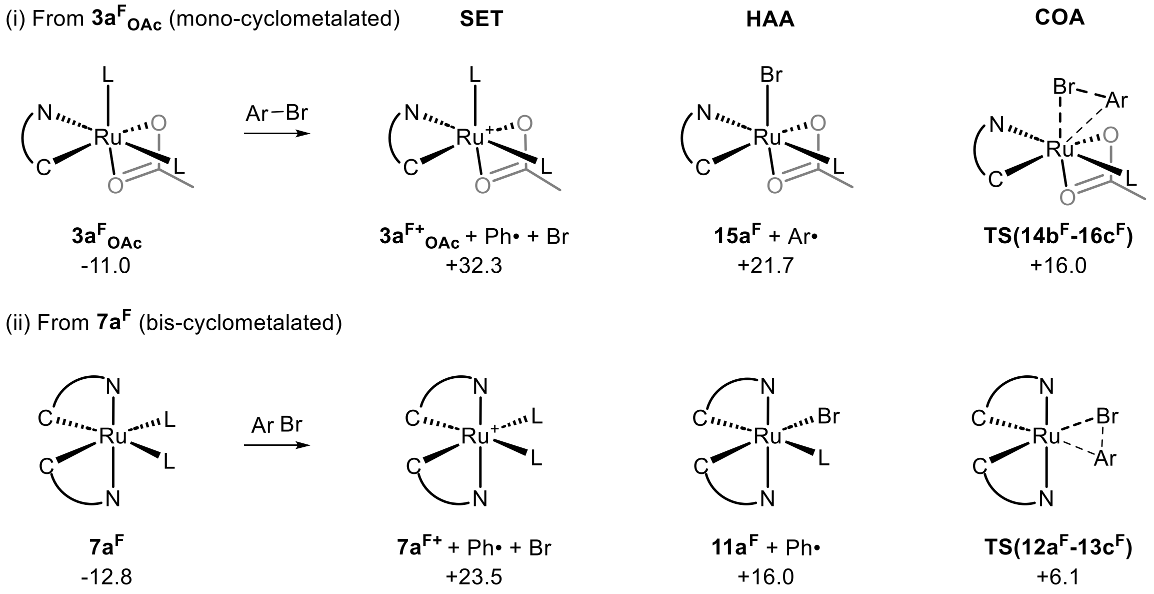

(i) mono-cyclometalated

Mechanism	ΔG/kcal/mol
SET	+43.3
HAA	+32.7
COA	+27.0

(ii) bis-cyclometalated

Mechanism	ΔG/kcal/mol
SET	+36.3
HAA	+28.8
COA	+18.9

Figure S49. Computed free energy changes (kcal/mol, L = MeCN) for alternative Ar–Br activation processes at (i) **3a^{F}$_{OAc}$** (mono-cyclometalated) and (ii) **7a^{F}** (bis-cyclometalated) Overall free energy changes are also indicated. Method: PBE(def2-tzvp,MeOH,D3BJ)// BP86(MeOH,SDD,6-31G**).

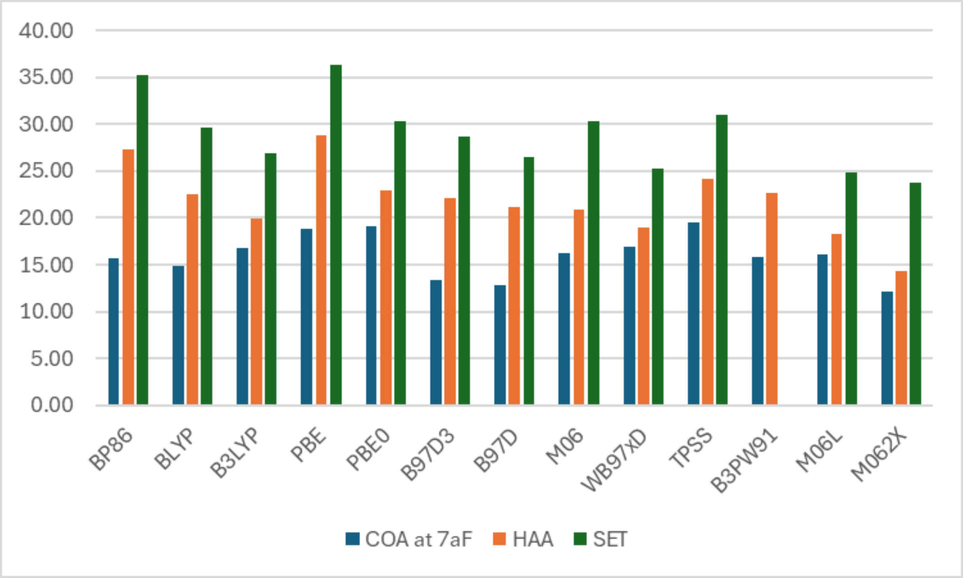

Figure S50. Free energies (kcal/mol) computed with different functionals for Ar–Br activation at **7a**^{F} via concerted oxidative addition (COA), halogen atom abstraction (HAA) and single electron transfer (SET).

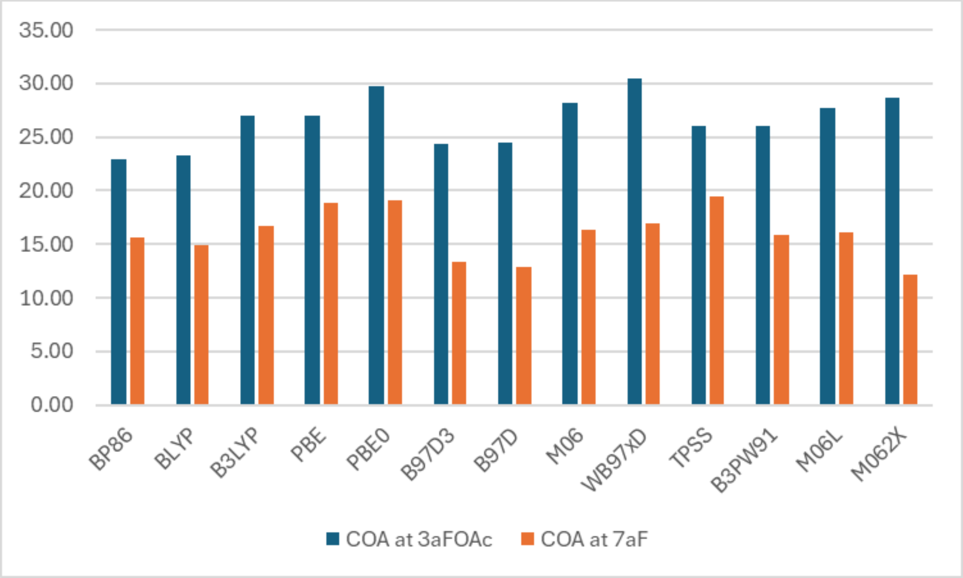

Figure S51. Free energies (kcal/mol) computed with different functionals for the lowest energy pathways for Ar–Br activation at bis-cyclometalated **7a**^{F} (COA via **TS(12a**^{F}**-13c**^{F}**)**) and via mono-cyclometalated **3a**$^{F}_{OAc}$ (COA via **TS(14b**^{F}**-16c**^{F}**)**).

15. Computed energies (au) and Cartesian coordinates of all species.

In the following:

- SCF(BP86) is the electronic energy after optimisation with the BP86 functional in MeOH solvent.
- G(298 K) is the free energy associated with that geometry
- SCF(PBE) is the corrected energy computed with the PBE functional with a the def2-tzvp basis set and including corrections for MeOH solvent and dispersion (BJD3)

15.1 Model 1.

```
6
MeCN
SCF(BP86)= -132.758496813
G(298 K) = -132.738474
SCF(PBE) = -132.645834184
Low Freq. = 386.2682cm-1,
387.3596cm-1
N   1.44973 -0.00005 -0.00001
C   0.27547  0.00012  0.00003
C  -1.18457 -0.00003 -0.00000
H  -1.56457  0.28923  0.99284
H  -1.56451  0.71520 -0.74697
H  -1.56441 -1.00457 -0.24595

8
HOAc
SCF(BP86)= -229.094757012
G(298 K) = -229.061803
SCF(PBE) = -228.938241641
Low Freq. = 55.2702cm-1,
410.8832cm-1
O  -0.77341 -1.05926 -0.00007
H  -1.72734 -0.82217 -0.00023
C  -0.09224  0.12262  0.00026
O  -0.65819  1.21063 -0.00007
C   1.40257 -0.10247 -0.00001
H   1.69723 -0.68633  0.88734
H   1.69692 -0.68649 -0.88736
H   1.92392  0.86311 -0.00020

7
OAc-
SCF(BP86)= -228.602181107
G(298 K) = -228.582026
SCF(PBE) = -228.466411053
Low Freq. = 50.6031cm-1,
413.4339cm-1
O   0.72038  1.16376  0.00157
C   0.20306  0.00038 -0.00737
O   0.80725 -1.11925  0.00163
C  -1.36102 -0.04220 -0.00246
H  -1.74225  0.29702  0.97933
H  -1.77510  0.65299 -0.75500
H  -1.75592 -1.05517 -0.19099

21
2-phenylpyridine
SCF(BP86)= -479.343363591
G(298 K) = -479.212739
SCF(PBE) = -478.928599592
Low Freq. = 46.3306cm-1,
90.8371cm-1
C   3.53778 -0.08611 -0.02194
C   2.71897 -1.20914 -0.22129
N   1.37275 -1.17106 -0.21684
C   0.76553  0.02704 -0.00156
C   1.51223  1.20596  0.22942
C   2.91125  1.14714  0.21290
C  -0.72608  0.02532 -0.00433
C  -1.42664 -1.18644  0.19938
C  -2.82777 -1.21279  0.20935
C  -3.55982 -0.02897  0.00937
C  -2.87609  1.18000 -0.20469
C  -1.47351  1.20789 -0.21225
H  -0.96119  2.15541 -0.40690
H  -3.35149 -2.16002  0.37640
H  -4.65450 -0.04898  0.01601
H  -3.43624  2.10533 -0.37493
H  -0.84754 -2.10174  0.35130
H   1.00752  2.15273  0.44041
H   3.16904 -2.19516 -0.39818
H   3.50280  2.05090  0.39090
H   4.62724 -0.18050 -0.04311

12
PhBr
SCF(BP86)= -245.059957503
G(298 K) = -245.003269
SCF(PBE) = -2805.21168795
Low Freq. = 160.5163cm-1,
238.9968cm-1
Br  1.82448  0.00000  0.00000
C  -0.11136  0.00000 -0.00000
C  -0.79046 -1.22706 -0.00000
C  -0.79046  1.22706 -0.00000
C  -2.19638 -1.21567 -0.00000
H  -0.23869 -2.17045  0.00000
C  -2.19638  1.21567 -0.00000
H  -0.23869  2.17045 -0.00000
C  -2.90003 -0.00000 -0.00001
H  -2.73734 -2.16733 -0.00000
H  -2.73734  2.16733 -0.00001
H  -3.99426 -0.00000 -0.00001

45
1
SCF(BP86)= -1104.78669141
G(298 K) = -1104.500983
SCF(PBE) = -1103.88724119
Low Freq. = 24.5126cm-1,
24.8407cm-1
N   1.00110 -1.35971 -0.00003
```

```
C   2.28124 -0.84466 -0.00006
C   3.38803 -1.71871 -0.00009
C   3.19413 -3.10276 -0.00009
C   1.88322 -3.60853 -0.00005
C   0.81887 -2.70384 -0.00002
C   2.34206  0.62155 -0.00006
C   3.55594  1.34741 -0.00010
C   3.53630  2.74747 -0.00010
C   2.30086  3.42318 -0.00006
C   1.09285  2.70207 -0.00003
C   1.07451  1.28949 -0.00003
H   4.51919  0.82485 -0.00012
Ru -0.54068  0.04590  0.00001
N  -1.90095  1.52680  0.00005
C  -2.70039  2.38621  0.00008
C  -3.68256  3.46207  0.00011
N  -0.52878  0.04195 -2.00124
C  -0.51167  0.03595 -3.17373
C  -0.49369  0.02541 -4.62978
N  -2.14385 -1.38539  0.00006
C  -3.04283 -2.13970  0.00008
C  -4.15640 -3.07907  0.00011
N  -0.52867  0.04195  2.00127
C  -0.51150  0.03597  3.17375
C  -0.49344  0.02545  4.62981
H  -1.52337  0.00617  5.02177
H   0.04204 -0.86589  4.99530
H   0.01507  0.92620  5.01036
H  -4.11140 -3.71870  0.89634
H  -5.11333 -2.53257  0.00012
H  -4.11143 -3.71872 -0.89611
H   4.39961 -1.30557 -0.00012
H   4.05368 -3.77949 -0.00011
H   1.68033 -4.68217 -0.00004
H  -0.22042 -3.03959  0.00001
H   4.47573  3.30946 -0.00012
H   2.27996  4.51942 -0.00006
H   0.14660  3.25493 -0.00001
H  -4.32155  3.39240  0.89551
H  -3.17713  4.44176  0.00016
H  -4.32153  3.39247 -0.89530
H  -1.52362  0.00523 -5.02168
H   0.01403  0.92658 -5.01039
H   0.04253 -0.86549 -4.99528

39
Int(1-2)a
SCF(BP86)= -972.004470367
G(298 K) = -971.760324
SCF(PBE) = -971.218063315
Low Freq. = 4.8583cm-1, 32.8633cm-
1
N  -1.48475  0.00001 -0.84655
C  -2.20224 -0.00003  0.32997
C  -3.61069 -0.00004  0.29191
C  -4.27698 -0.00001 -0.93770
C  -3.52729  0.00004 -2.12715
C  -2.13273  0.00005 -2.03650
C  -1.35802 -0.00007  1.52752
C  -1.85995 -0.00012  2.84896
C  -0.97903 -0.00015  3.93724
C   0.41051 -0.00013  3.70723
C   0.92219 -0.00007  2.39693
C   0.05418 -0.00004  1.28471
H  -2.94079 -0.00015  3.02907
Ru  0.57528  0.00003 -0.63776
N   2.56737  0.00004 -0.39223
C   3.73961  0.00005 -0.31065
C   5.19137  0.00005 -0.20205
N   0.57082 -1.98959 -0.71851
C   0.55714 -3.16113 -0.79583
C   0.54259 -4.61243 -0.90247
N   0.57077  1.98965 -0.71839
C   0.55704  3.16120 -0.79565
C   0.54246  4.61250 -0.90220
H   1.40918  4.95740 -1.48949
H  -0.38180  4.94743 -1.40071
H   0.59013  5.06666  0.10109
H  -4.17689 -0.00008  1.22664
H  -5.37041 -0.00001 -0.96995
H  -4.00724  0.00007 -3.10853
H  -1.49465  0.00008 -2.92563
H  -1.37008 -0.00020  4.95934
H   1.10385 -0.00015  4.55613
H   2.00577 -0.00005  2.24268
H   5.60773  0.89659 -0.68992
H   5.49649 -0.00126  0.85735
H   5.60791 -0.89516 -0.69220
H   1.40935 -4.95727 -1.48975
H   0.59023 -5.06665  0.10079
H  -0.38164 -4.94735 -1.40104

39
Int(1-2)b
SCF(BP86)= -971.985187843
G(298 K) = -971.741530
SCF(PBE) = -971.197435885
Low Freq. = 30.3401cm-1,
33.2483cm-1
N   0.05578  0.00094  1.27962
C   1.42388  0.00114  1.50168
C   1.90903  0.00236  2.82421
C   1.02393  0.00334  3.90571
C  -0.35896  0.00308  3.65501
C  -0.80431  0.00187  2.33205
C   2.22273  0.00006  0.27655
C   3.63543 -0.00017  0.24038
C   4.29573 -0.00129 -0.99521
C   3.54696 -0.00218 -2.18947
C   2.13960 -0.00195 -2.15174
C   1.44591 -0.00083 -0.92316
H   4.22125  0.00049  1.16665
Ru -0.55779 -0.00039 -0.62819
N  -0.56002 -1.99175 -0.69268
C  -0.55703 -3.16425 -0.75379
C  -0.56010 -4.61703 -0.84026
N  -2.68157 -0.00013 -0.35940
C  -3.85476 -0.00020 -0.30586
C  -5.30914 -0.00032 -0.23600
N  -0.55967  1.99084 -0.69518
C  -0.55654  3.16325 -0.75803
C  -0.55944  4.61589 -0.84669
```

```
H  -1.42946  4.95752 -1.43102
H  -0.61541  5.05610  0.16238
H   0.36160  4.96945 -1.33817
H  -5.66214  0.89663  0.29813
H  -5.73666 -0.00118 -1.25193
H  -5.66191 -0.89653  0.29954
H   2.98900  0.00257  2.99241
H   1.40500  0.00431  4.93083
H  -1.09171  0.00381  4.46537
H  -1.86502  0.00165  2.07532
H   5.38975 -0.00148 -1.03105
H   4.06577 -0.00308 -3.15514
H   1.57893 -0.00268 -3.09594
H  -1.43046 -4.95947 -1.42362
H   0.36065 -4.97141 -1.33167
H  -0.61559 -5.05570  0.16951

39
Int(1-2)c
SCF(BP86)= -971.982124836
G(298 K) = -971.738308
SCF(PBE) = -971.194628312
Low Freq. = 23.1494cm-1,
32.1214cm-1
N   0.97617 -1.34505 -0.24063
C   2.25022 -0.81454 -0.28370
C   3.36725 -1.67260 -0.28541
C   3.19088 -3.05905 -0.23419
C   1.88810 -3.58099 -0.17775
C   0.81126 -2.69099 -0.18204
C   2.28819  0.65328 -0.31968
C   3.48748  1.39698 -0.38496
C   3.44490  2.79764 -0.39431
C   2.20246  3.45631 -0.33636
C   1.00648  2.71769 -0.27582
C   1.01044  1.30383 -0.28042
H   4.45791  0.89013 -0.42788
Ru -0.57822  0.04298 -0.31075
N  -1.94729  1.49667 -0.47469
C  -2.75934  2.33903 -0.57866
C  -3.75600  3.39160 -0.71307
N  -2.13560 -1.40320 -0.54231
C  -3.00569 -2.17034 -0.72490
C  -4.08024 -3.12561 -0.95385
N  -0.68516  0.05309  1.58850
C  -0.74061  0.05086  2.76206
C  -0.82532  0.03064  4.21609
H  -1.85253 -0.21231  4.53463
H  -0.14003 -0.73016  4.62484
H  -0.54894  1.01400  4.63065
H  -4.03049 -3.93623 -0.20891
H  -5.05769 -2.62350 -0.87142
H  -3.98724 -3.56204 -1.96167
H   4.37301 -1.24754 -0.32523
H   4.05911 -3.72444 -0.23518
H   1.70035 -4.65626 -0.13132
H  -0.22284 -3.03997 -0.14264
H   4.37489  3.37298 -0.44139
H   2.16599  4.55187 -0.33527
H   0.05345  3.25601 -0.22704
H  -4.76726  2.95736 -0.77247
H  -3.70970  4.06808  0.15612
H  -3.56587  3.97797 -1.62706

39
TS(Int(1-2)a-Int(1-2)b)
SCF(BP86)= -971.975404315
G(298 K) = -971.732407
SCF(PBE) = -971.186712450
Low Freq. = -165.7709cm-1,
24.3528cm-1
N   0.96939 -0.00001 -1.20362
C   2.19226 -0.00000 -0.55942
C   3.38030 -0.00001 -1.31445
C   3.32317 -0.00001 -2.71170
C   2.06876 -0.00002 -3.34694
C   0.91503 -0.00001 -2.55891
C   2.05264  0.00001  0.89186
C   3.11265  0.00001  1.82468
C   2.82611  0.00002  3.19613
C   1.48547  0.00002  3.63256
C   0.42653  0.00001  2.70439
C   0.69064  0.00001  1.32075
H   4.15471  0.00001  1.48493
Ru -0.71993 -0.00000 -0.09647
N  -0.74764  1.98865 -0.11082
C  -0.76027  3.16328 -0.12500
C  -0.79114  4.61815 -0.14463
N  -2.90443 -0.00000 -0.38243
C  -4.05064 -0.00000 -0.13029
C  -5.47422  0.00000  0.16053
N  -0.74764 -1.98865 -0.11080
C  -0.76027 -3.16328 -0.12497
C  -0.79113 -4.61816 -0.14458
H  -1.27385 -5.00044  0.76992
H  -1.35675 -4.97527 -1.02091
H   0.23470 -5.01767 -0.19686
H  -5.94585 -0.89930 -0.26676
H  -5.62552  0.00000  1.25233
H  -5.94585  0.89930 -0.26676
H   4.34280 -0.00000 -0.79616
H   4.24446 -0.00002 -3.30114
H   1.97515 -0.00002 -4.43531
H  -0.08897 -0.00002 -2.99252
H   3.64160  0.00002  3.92602
H   1.26343  0.00003  4.70578
H  -0.61048  0.00002  3.06361
H  -1.27402  5.00044  0.76979
H   0.23471  5.01767 -0.19672
H  -1.35660  4.97527 -1.02105

39
TS(Int(1-2)a-Int(1-2)c)
SCF(BP86)= -971.973144573
G(298 K) = -971.730099
SCF(PBE) = -971.185495162
Low Freq. = -169.6030cm-1,
21.0077cm-1
N  -1.34005  0.93159  0.22490
C  -2.28049  0.08350 -0.32239
C  -3.64277  0.44284 -0.31127
C  -4.04364  1.65075  0.26778
```

```
C  -3.07411  2.49404  0.83821
C  -1.73542  2.09813  0.79522
C  -1.70850 -1.14974 -0.86730
C  -2.45934 -2.13277 -1.55048
C  -1.83310 -3.28760 -2.03533
C  -0.45051 -3.46661 -1.83349
C   0.30834 -2.49489 -1.15739
C  -0.29429 -1.30680 -0.68578
H  -3.53475 -1.99533 -1.70856
Ru  0.62299  0.26971  0.12650
N   2.49198 -0.42490 -0.06691
C   3.59902 -0.80350 -0.17429
C   4.96726 -1.27593 -0.32770
N   1.34096  2.26888 -0.40677
C   1.60978  3.26675 -0.96375
C   1.94287  4.51153 -1.63464
N   0.41969 -0.62724  1.80025
C   0.29059 -1.17721  2.83243
C   0.15480 -1.80042  4.14205
H   0.63971 -1.18173  4.91579
H  -0.91166 -1.91370  4.39752
H   0.62255 -2.79877  4.14168
H   1.68654  5.36328 -0.98394
H   3.01899  4.54236 -1.86808
H   1.36604  4.59240 -2.57035
H  -4.38266 -0.23081 -0.75047
H  -5.10101  1.93067  0.27975
H  -3.34286  3.44307  1.30796
H  -0.93709  2.71543  1.21506
H  -2.41785 -4.04755 -2.56267
H   0.04100 -4.37485 -2.20056
H   1.37935 -2.66125 -1.00511
H   5.67957 -0.48748 -0.03438
H   5.13564 -2.16123  0.30696
H   5.15606 -1.55271 -1.37813



46
2a OAc
SCF(BP86)= -1200.65437674
G(298 K) = -1200.367358
SCF(PBE) = -1199.71563265
Low Freq. = 15.7448cm-1,
21.5053cm-1
C   3.79104  1.02218 -0.35218
C   2.51738  0.42695 -0.19531
C   1.31924  1.21899 -0.13636
C   1.48374  2.62150 -0.24207
C   2.75039  3.21206 -0.39715
C   3.91037  2.41375 -0.45338
C   2.31929 -1.02267 -0.07683
N   1.00148 -1.40021  0.07612
C   0.68161 -2.71111  0.20788
C   1.64724 -3.72148  0.18990
C   2.99642 -3.35990  0.02906
C   3.32874 -2.00809 -0.10289
Ru -0.39486  0.14589  0.08511
O  -1.98922 -1.38265  0.44735
C  -3.18043 -1.45381 -0.07837
C  -3.97205 -2.69896  0.37484
N  -0.55112 -0.01227 -1.89173
C  -0.61507 -0.11159 -3.06003
C  -0.72093 -0.24472 -4.50723
N  -0.24928  0.25605  2.06869
C  -0.16706  0.29942  3.23950
C  -0.06939  0.35314  4.69243
N  -1.59763  1.73154  0.08820
C  -2.27967  2.68973  0.10541
C  -3.14837  3.86041  0.11890
O  -3.70514 -0.64586 -0.87814
H   4.37265 -1.70822 -0.22609
H  -1.77811 -0.34314 -4.80469
H  -0.29381  0.63985 -5.00766
H  -0.17457 -1.13955 -4.84793
H  -2.93025  4.51025 -0.74489
H  -4.20818  3.55809  0.07243
H  -2.98994  4.44327  1.04136
H   4.69444  0.40233 -0.39520
H   4.89638  2.87414 -0.57459
H   2.83707  4.30283 -0.47524
H   0.59993  3.26954 -0.20192
H   3.78015 -4.12340  0.00826
H   1.34273 -4.76557  0.29934
H  -0.38955 -2.90123  0.33368
H  -1.07523  0.33191  5.14328
H   0.50272 -0.51146  5.06723
H   0.44071  1.27773  5.00920
H  -3.45093 -3.61827  0.05556
H  -4.04023 -2.73474  1.47547
H  -4.98649 -2.69623 -0.05165

46
2b OAc
SCF(BP86)= -1200.65229453
G(298 K) = -1200.365274
SCF(PBE) = -1199.71180376
Low Freq. = 22.6587cm-1,
23.1695cm-1
C  -1.14697 -1.30583  0.00202
C  -2.42070 -0.68243 -0.22406
C  -3.59984 -1.44488 -0.39471
C  -3.54770 -2.84324 -0.34109
C  -2.31125 -3.47926 -0.11277
C  -1.13905 -2.72119  0.05605
C  -2.40363  0.78417 -0.24954
N  -1.15468  1.33842 -0.04651
C  -1.02403  2.68901 -0.02952
C  -2.10183  3.55913 -0.21344
C  -3.37794  3.01119 -0.42856
C  -3.52248  1.62085 -0.44478
Ru  0.41273 -0.01746  0.16831
N   0.22844  0.01614  2.13688
C   0.12597  0.03586  3.30975
C  -0.00020  0.06912  4.76239
N   1.77036 -1.46647  0.33460
C   2.55016 -2.33295  0.48575
C   3.52478 -3.40779  0.62693
N   1.93310  1.47162  0.28193
C   2.76979  2.28945  0.39208
C   3.81212  3.30157  0.51517
H  -4.56339 -0.95181 -0.56939
```

```
H   0.85514  0.60058  5.21206
H  -0.92767  0.58941  5.05422
H  -0.03069 -0.95463  5.17112
H   3.46632  4.13328  1.15051
H   4.71662  2.86350  0.96795
H   4.07318  3.70060 -0.47874
H  -4.50620  1.17356 -0.60891
H  -4.24685  3.65866 -0.58032
H  -1.93577  4.63915 -0.18999
H  -0.01030  3.06106  0.13568
H  -4.46040 -3.43344 -0.47374
H  -2.26332 -4.57425 -0.06774
H  -0.19239 -3.24667  0.22866
H   4.24582 -3.17198  1.42709
H   3.02232 -4.35697  0.87728
H   4.08101 -3.54309 -0.31585
O   0.44659  0.02370 -1.94078
C   1.49865 -0.12464 -2.70972
O   2.67868 -0.31125 -2.35077
C   1.13863 -0.08141 -4.20591
H   0.39083  0.69982 -4.41717
H   2.03945  0.09127 -4.81378
H   0.69683 -1.04795 -4.50651

46
2c OAc
SCF(BP86)= -1200.65042640
G(298 K) = -1200.363311
SCF(PBE) = -1199.71031458
Low Freq. = 21.0477cm-1,
30.6644cm-1
C   1.07197 -1.34018 -0.16043
C   2.39562 -0.82139  0.02455
C   3.52946 -1.66773  0.00346
C   3.37222 -3.04428 -0.20212
C   2.07944 -3.57554 -0.38871
C   0.95277 -2.73434 -0.36760
C   2.47024  0.62981  0.23165
N   1.24260  1.26891  0.20874
C   1.19561  2.61353  0.39504
C   2.33599  3.39262  0.60686
C   3.59014  2.75865  0.63007
C   3.64927  1.37441  0.44196
Ru -0.41196  0.04607 -0.09621
N  -0.26052  0.28332 -2.06971
C  -0.16593  0.41726 -3.23331
C  -0.05241  0.58796 -4.67633
N  -0.54514 -0.22652  1.86819
C  -0.56484 -0.37663  3.03287
C  -0.63561 -0.57499  4.47498
N  -1.84838  1.60536 -0.05274
C  -2.60923  2.50217 -0.06857
C  -3.56332  3.60503 -0.08177
H   4.53675 -1.25863  0.14604
H  -1.03912  0.46780 -5.15336
H   0.33371  1.59283 -4.91477
H   0.63583 -0.16310 -5.09778
H  -3.42522  4.21790 -0.98757
H  -4.59654  3.22016 -0.07045
H  -3.41678  4.24724  0.80221
H   4.61320  0.85883  0.45779
H   4.50609  3.33440  0.79333
H   2.23429  4.47156  0.74965
H   0.19671  3.05528  0.36706
H   4.24887 -3.70039 -0.21828
H   1.95457 -4.65339 -0.55103
H  -0.04837 -3.15638 -0.51705
H  -1.63098 -0.95651  4.75777
H   0.12552 -1.30299  4.80068
H  -0.46029  0.37619  5.00435
O  -1.86961 -1.43541 -0.54117
C  -2.99453 -1.63651  0.09746
O  -3.43374 -1.00223  1.07999
C  -3.79011 -2.82605 -0.47395
H  -3.84130 -2.77882 -1.57409
H  -3.28582 -3.77295 -0.21225
H  -4.80839 -2.84507 -0.05766

46
TS(2a-3a)OAc
SCF(BP86)= -1200.61867669
G(298 K) = -1200.333298
SCF(PBE) = -1199.67946220
Low Freq. = -129.0439cm-1,
4.4467cm-1
C  -3.35726 -1.70439  0.13832
C  -2.23623 -0.87045 -0.05493
N  -0.96516 -1.40469  0.02973
C  -0.80288 -2.72861  0.28018
C  -1.88354 -3.59276  0.47465
C  -3.18583 -3.06650  0.40507
C  -2.26020  0.56572 -0.35064
C  -3.45533  1.30756 -0.49432
C  -3.40427  2.67758 -0.78145
C  -2.15100  3.30550 -0.93019
C  -0.96134  2.56981 -0.78720
C  -0.96941  1.18639 -0.48210
Ru  0.59469 -0.07267 -0.23651
N   2.00225  1.31061 -0.42457
C   2.84258  2.12839 -0.53441
C   3.87506  3.15080 -0.65055
O   2.12628 -1.72004  0.01082
C   2.57777 -1.53435  1.20927
O   2.15144 -0.60829  1.95721
N   0.59845 -0.40443 -2.10861
C   0.60536 -0.62222 -3.26696
C   0.63871 -0.91279 -4.69555
C   3.63937 -2.51472  1.70973
N  -0.36603  0.69925  2.35008
C  -0.59545  1.29113  3.33723
C  -0.87664  2.02568  4.56770
H  -4.36250 -1.27913  0.07752
H  -0.18622  1.70866  5.36550
H  -0.75260  3.10730  4.39877
H  -1.91099  1.83465  4.89527
H   3.45118  4.09049 -1.04271
H   4.32139  3.35290  0.33748
H   4.67493  2.81485 -1.33131
H  -4.43098  0.81937 -0.38364
H  -4.33003  3.25152 -0.89311
H  -2.10434  4.37714 -1.16042
H  -0.00166  3.08732 -0.90641
```

H -4.05690 -3.71172 0.55468
H -1.70241 -4.65174 0.67565
H 0.23887 -3.06123 0.31668
H 1.55427 -0.49740 -5.14925
H 0.62782 -2.00222 -4.86860
H -0.23385 -0.46916 -5.20377
H 3.17096 -3.49395 1.91228
H 4.41316 -2.67651 0.94170
H 4.10826 -2.15273 2.63653

46
TS(2a-3b)OAc
SCF(BP86)= -1200.61810399
G(298 K) = -1200.333024
SCF(PBE) = -1199.67855349
Low Freq. = -36.9063cm-1,
13.6225cm-1
C -2.92362 2.60726 0.43071
C -2.10525 1.47959 0.18919
C -0.68385 1.51276 0.39526
C -0.14235 2.73865 0.85408
C -0.95505 3.86099 1.09588
C -2.34834 3.80023 0.88551
C -2.61074 0.19340 -0.28920
N -1.62421 -0.76676 -0.44645
C -1.94185 -2.00746 -0.90329
C -3.25405 -2.36391 -1.22122
C -4.27594 -1.41055 -1.06229
C -3.94672 -0.13396 -0.59685
Ru 0.24739 -0.24919 0.00944
O 1.03248 -2.27179 -0.50742
C 2.10075 -2.69423 0.10291
C 2.49553 -4.14157 -0.23565
N -0.01536 -0.78829 1.89808
C -0.19040 -1.09845 3.01877
C -0.37751 -1.49535 4.40779
N 0.59681 0.26973 -1.87061
C 0.80353 0.55413 -2.99297
C 1.08575 0.91280 -4.37644
N 2.95192 1.47348 0.19376
C 3.97290 2.04847 0.12559
C 5.24213 2.76463 0.04368
O 2.78817 -2.02902 0.91706
H -4.72307 0.62532 -0.46985
H 0.26706 -2.35659 4.65021
H -0.12321 -0.66185 5.08337
H -1.42775 -1.78113 4.58362
H 5.50282 3.18416 1.02842
H 6.04306 2.07825 -0.27414
H 5.16688 3.58698 -0.68547
H -4.00699 2.55856 0.26747
H -2.97644 4.67679 1.07551
H -0.50168 4.79442 1.45200
H 0.93910 2.81357 1.01887
H -5.31426 -1.65998 -1.30011
H -3.46255 -3.37337 -1.58473
H -1.09108 -2.68903 -0.99550
H 2.13672 0.68494 -4.62062
H 0.43550 0.34229 -5.06004
H 0.91052 1.98909 -4.53940
H 1.71614 -4.83714 0.12127
H 2.57114 -4.27574 -1.32781
H 3.45417 -4.41019 0.23243

46
TS(2b-3c)OAc
SCF(BP86)= -1200.637550
G(298 K) = -1200.334609
SCF(PBE) = -1199.67766926
Low Freq. = -91.6975cm-1,
14.3527cm-1
C 0.71360 1.49502 -0.23322
C 2.14331 1.40941 -0.15354
C 2.96871 2.54636 -0.31013
C 2.38922 3.80030 -0.54365
C 0.98519 3.91433 -0.61415
C 0.16550 2.78182 -0.45834
C 2.64229 0.06233 0.12028
N 1.63553 -0.88688 0.25429
C 1.97188 -2.17332 0.55128
C 3.29544 -2.58827 0.70945
C 4.32794 -1.64592 0.55922
C 3.98951 -0.32097 0.26645
Ru -0.24075 -0.27214 -0.04575
N -0.48958 -0.00052 1.88866
C -0.60183 0.14172 3.05276
C -0.76517 0.31964 4.49101
N -2.82282 1.13121 0.48076
C -3.81899 1.72505 0.66150
C -5.05826 2.46625 0.87963
N -1.20413 -2.16331 0.06471
C -1.78025 -3.18829 0.10552
C -2.49494 -4.45808 0.15136
H 4.06032 2.45959 -0.25024
H -1.75519 0.75172 4.71446
H -0.67976 -0.64711 5.01531
H 0.00805 0.99986 4.88546
H -2.05505 -5.16940 -0.56687
H -2.43405 -4.89425 1.16192
H -3.55672 -4.30875 -0.10516
H 4.77040 0.43556 0.15014
H 5.37578 -1.93906 0.67138
H 3.50236 -3.63564 0.94397
H 1.13337 -2.86503 0.65040
H 3.02330 4.68450 -0.66695
H 0.52971 4.89621 -0.79268
H -0.92403 2.89634 -0.51520
H -5.58930 2.06742 1.75856
H -4.83646 3.53151 1.05220
H -5.71281 2.37770 -0.00204
O 0.09514 -0.61569 -2.10254
C -1.01223 -0.40294 -2.75635
O -2.06820 -0.01777 -2.19327
C -0.96668 -0.68963 -4.25517
H -1.17256 -1.76041 -4.43056
H -1.73516 -0.10449 -4.78209
H 0.02664 -0.46677 -4.67485

46
TS(2b-3a)OAc
SCF(BP86)= -1200.620039
G(298 K) = -1200.352165

SCF(PBE) = -1199.69741698
Low Freq. = -64.7417cm-1, 7.0795cm-1

C -1.58167 0.92889 -0.37798
C -2.62583 -0.05046 -0.23806
C -3.98043 0.29459 -0.44928
C -4.32942 1.60126 -0.81174
C -3.31598 2.56877 -0.97029
C -1.96655 2.23743 -0.76082
C -2.17214 -1.39854 0.10948
N -0.80054 -1.49976 0.23372
C -0.24089 -2.70087 0.52589
C -1.00209 -3.85702 0.72044
C -2.40119 -3.76968 0.60820
C -2.98317 -2.53578 0.29962
Ru 0.23943 0.27645 0.02675
N 0.59328 -0.07223 -1.87886
C 0.79946 -0.27780 -3.02056
C 1.07574 -0.54505 -4.42709
N 1.14781 2.04057 -0.09806
C 1.70639 3.07533 -0.16224
C 2.39275 4.35948 -0.22549
N 2.82805 -1.40673 -0.16293
C 3.91387 -1.73596 -0.46432
C 5.26460 -2.14524 -0.83707
H -4.76708 -0.46011 -0.33277
H 2.16372 -0.57670 -4.60579
H 0.64458 -1.51511 -4.72615
H 0.63982 0.24199 -5.06497
H 5.26213 -2.58748 -1.84600
H 5.93803 -1.27337 -0.83170
H 5.64435 -2.89180 -0.12147
H -4.06865 -2.44694 0.20445
H -3.02914 -4.65317 0.75766
H -0.50335 -4.80070 0.95559
H 0.85093 -2.70057 0.59188
H -5.37920 1.86580 -0.97381
H -3.58218 3.59271 -1.25913
H -1.20017 3.00916 -0.88887
H 2.92973 4.46108 -1.18317
H 1.67032 5.18849 -0.13953
H 3.12383 4.44455 0.59592
O -0.14716 0.58632 2.07135
C 0.89268 0.58085 2.86731
O 2.07517 0.38658 2.50462
C 0.54795 0.85618 4.33530
H -0.29205 0.22270 4.66431
H 1.42091 0.67477 4.97911
H 0.22992 1.90643 4.45481

46

TS(2a-κ[1]-3a)$_{OAc}$
SCF(BP86)= -1201.15462875
G(298 K) = -1200.331309
SCF(PBE) = -1199.67619363
Low Freq. = -96.9625cm-1, 9.6307cm-1

C -1.21099 0.55837 1.06340
C -2.44830 0.09391 0.49483
C -3.69801 0.54345 0.97940
C -3.75469 1.45014 2.04572
C -2.55663 1.90685 2.63045
C -1.31234 1.46278 2.14938
C -2.31169 -0.85599 -0.61602
C -3.36314 -1.47904 -1.31866
C -3.08298 -2.37056 -2.35949
C -1.74357 -2.63319 -2.69681
C -0.73431 -1.99161 -1.97425
N -1.00265 -1.12643 -0.96380
Ru 0.44988 -0.14835 0.14692
N 0.50678 -1.58205 1.37844
C 0.53212 -2.47883 2.14254
C 0.61926 -3.60943 3.05869
N 1.72861 0.93287 1.21070
C 2.47290 1.60578 1.82594
C 3.40160 2.44396 2.57268
O 1.90099 -0.62434 -1.40823
C 3.17124 -0.92368 -1.28410
C 3.96840 -0.77071 -2.59029
O 3.73958 -1.30857 -0.24000
N 0.20561 2.73236 -1.53303
C -0.13772 3.67536 -2.14148
C -0.56241 4.84905 -2.89758
H -4.39873 -1.26361 -1.04307
H 1.55923 -4.16505 2.89986
H 0.58843 -3.26356 4.10544
H -0.22584 -4.29878 2.89574
H 3.15441 2.42981 3.64718
H 4.43707 2.08699 2.44348
H 3.34220 3.48551 2.21507
H -4.63141 0.18937 0.52636
H -4.72290 1.79669 2.42139
H -2.59581 2.61173 3.46989
H -0.39731 1.83371 2.62625
H -3.89983 -2.85645 -2.90167
H -1.47962 -3.32164 -3.50353
H 0.33003 -2.12576 -2.18826
H 3.46532 -1.29874 -3.41733
H 4.02080 0.29504 -2.87385
H 4.99123 -1.15891 -2.47430
H -0.48601 5.75090 -2.26965
H 0.07626 4.97724 -3.78595
H -1.60777 4.72922 -3.22387

40

κ[1]-3a$_{OAc}$
SCF(BP86)= -1068.33511853
G(298 K) = -1067.610926
SCF(PBE) = -1067.02746055
Low Freq. = 25.0222cm-1, 27.5454cm-1

C -3.56724 1.35274 -0.76041
C -2.38577 0.62378 -0.49338
C -1.10868 1.27897 -0.39493
C -1.10104 2.68801 -0.54250
C -2.27906 3.40895 -0.80510
C -3.51626 2.74362 -0.91792
C -2.36041 -0.83220 -0.31344
N -1.09396 -1.34878 -0.12131
C -0.92733 -2.68251 0.06702
C -2.00048 -3.57701 0.06918
C -3.29734 -3.07165 -0.13070

```
C  -3.47321 -1.69741 -0.32301
Ru  0.45687  0.02320 -0.14798
O   1.78271 -1.54677 -0.76036
C   3.06913 -1.70036 -0.53129
C   3.80248 -2.49007 -1.62352
N   0.31377  0.29197  1.72172
C   0.22537  0.44719  2.88717
C   0.17565  0.57703  4.33833
N   1.86056  1.41736 -0.31726
C   2.68718  2.24600 -0.44151
C   3.71731  3.26325 -0.60345
O   3.67888 -1.27910  0.47207
H  -4.47444 -1.28733 -0.47800
H   0.72017 -0.25185  4.82240
H   0.63422  1.52913  4.65362
H  -0.86937  0.56300  4.69006
H   3.50682  4.12868  0.04654
H   4.70811  2.85650 -0.34057
H   3.74602  3.61106 -1.64955
H  -4.53088  0.83770 -0.84896
H  -4.43211  3.30696 -1.12368
H  -2.23545  4.49883 -0.91934
H  -0.15363  3.23275 -0.45608
H  -4.16197 -3.74211 -0.13511
H  -1.81773 -4.64338  0.22313
H   0.11040 -3.00321  0.19160
H   3.26532 -3.42477 -1.85404
H   3.83236 -1.89761 -2.55471
H   4.83255 -2.72199 -1.31533

40
3a OAc
SCF(BP86)= -1067.89095854
G(298 K) = -1067.644443
SCF(PBE) = -1067.05585667
Low Freq. = 17.1288cm-1,
19.3910cm-1
C  -3.69707 -0.30310 -0.24969
C  -2.32851  0.03667 -0.25957
N  -1.39195 -0.86919  0.20112
C  -1.79814 -2.08092  0.65966
C  -3.14260 -2.46059  0.69194
C  -4.10955 -1.55162  0.22664
C  -1.74528  1.29778 -0.72326
C  -2.50776  2.35803 -1.26575
C  -1.87860  3.53482 -1.68931
C  -0.47835  3.65184 -1.56774
C   0.28508  2.60164 -1.03096
C  -0.31339  1.39003 -0.60009
Ru  0.57806 -0.24909  0.12215
O   1.37655 -2.45761  0.21841
C   1.26536 -2.46684 -1.05470
C   1.67399 -3.67253 -1.87382
O   0.82193 -1.41044 -1.66322
N   0.46872  0.47142  1.93870
C   0.40669  0.86881  3.04671
C   0.34214  1.32317  4.43060
N   2.44231  0.40602 -0.06774
C   3.55844  0.76393 -0.18441
C   4.93347  1.22186 -0.34009
H  -4.43378  0.41678 -0.61598
H   1.28479  1.09085  4.95422
H   0.17650  2.41251  4.47585
H  -0.48449  0.82154  4.96128
H   5.06047  2.21719  0.11742
H   5.63380  0.52098  0.14415
H   5.19275  1.29326 -1.40963
H  -3.59631  2.26662 -1.36016
H  -2.46989  4.35503 -2.10904
H   0.01983  4.57312 -1.89369
H   1.37122  2.72322 -0.94716
H  -5.17166 -1.81446  0.23492
H  -3.41826 -3.44764  1.07166
H  -0.99539 -2.74549  0.99099
H   1.12830 -3.70265 -2.82853
H   2.75368 -3.61328 -2.09724
H   1.49690 -4.59886 -1.30716

40
3b OAc
SCF(BP86)= -1067.88805277
G(298 K) = -1067.641717
SCF(PBE) = -1067.05305357
Low Freq. = 29.0970cm-1,
30.9249cm-1
C   3.41026  0.02160 -1.41164
C   2.23286  0.01374 -0.63713
N   0.99599  0.00034 -1.25627
C   0.93161 -0.00580 -2.61212
C   2.07013  0.00136 -3.42288
C   3.33496  0.01554 -2.80849
C   2.14306  0.01722  0.82430
C   0.79959  0.00592  1.32794
C   0.64166  0.00627  2.73465
C   1.75233  0.01817  3.59753
C   3.06424  0.02988  3.07977
C   3.25863  0.02926  1.69232
Ru -0.66472 -0.00872 -0.04252
O  -2.54809 -0.02028  1.02889
C  -3.28333 -0.02198 -0.03684
C  -4.79247  0.02037  0.10983
N  -0.69293 -1.98753 -0.05661
C  -0.72324 -3.16377 -0.07722
C  -0.76758 -4.61984 -0.10919
N  -0.72117  1.96962 -0.06721
C  -0.76608  3.14529 -0.09368
C  -0.82856  4.60053 -0.13236
O  -2.73364 -0.02805 -1.18967
H   4.27890  0.03803  1.29018
H  -1.62284 -4.96350 -0.71450
H   0.15938 -5.02039 -0.55190
H  -0.87296 -5.02467  0.91107
H   4.38246  0.03232 -0.91109
H   4.24819  0.02165 -3.41099
H   1.95963 -0.00401 -4.51018
H  -0.07831 -0.01668 -3.03264
H   3.92560  0.03924  3.75561
H   1.59772  0.01835  4.68342
H  -0.36940 -0.00290  3.16186
H  -1.69539  4.93072 -0.72864
H  -0.92614  5.00925  0.88713
H   0.08789  5.01002 -0.58860
```

H -5.10748 -0.41555 1.06953
H -5.13236 1.07055 0.08244
H -5.27523 -0.51194 -0.72343

40
3c OAc
SCF(BP86)= -1067.88302718
G(298 K) = -1067.636477
SCF(PBE) = -1067.04627969
Low Freq. = 4.8263cm-1, 19.8679cm-1
C -2.55335 2.39848 -1.15934
C -1.80587 1.30384 -0.68141
N -0.42683 1.40275 -0.56303
C   0.17951 2.57430 -0.89888
C -0.52257 3.68315 -1.37582
C -1.91907 3.59408 -1.51141
C -2.34247 0.00040 -0.27833
C -1.35496 -0.94261 0.16490
C -1.82696 -2.21489 0.57199
C -3.19489 -2.54053 0.54036
C -4.14461 -1.59794 0.09651
C -3.71744 -0.32788 -0.31282
Ru 0.54959 -0.27410 0.09208
O   0.86971 -1.43065 -1.70790
C   1.34837 -2.41384 -1.01975
C   1.86514 -3.65647 -1.70117
N   0.44416 0.37330 1.92913
C   0.38643 0.74162 3.04797
C   0.33504 1.16648 4.44200
N   2.51418 0.47535 -0.11387
C   3.62146 0.85376 -0.24193
C   4.98980 1.32927 -0.40637
O   1.39953 -2.28133 0.25945
H -4.45926 0.40263 -0.65714
H   1.27437 0.90455 4.95780
H   0.19314 2.25807 4.51072
H -0.50016 0.67251 4.96646
H   5.12777 2.28555 0.12448
H   5.70378 0.59409 0.00003
H   5.21299 1.48434 -1.47486
H -3.63862 2.30344 -1.25220
H -2.50184 4.44217 -1.88276
H   0.02258 4.59549 -1.63153
H   1.26454 2.59125 -0.77437
H -5.20913 -1.85347 0.07184
H -3.52729 -3.53513 0.86235
H -1.10573 -2.96720 0.91453
H   1.30657 -3.84780 -2.62957
H   2.92823 -3.51757 -1.96534
H   1.78997 -4.52391 -1.02896

34
Int(3-4)aOAc
SCF(BP86)= -935.100696823
G(298 K) = -934.895980
SCF(PBE) = -934.376080424
Low Freq. = 21.0957cm-1, 33.6242cm-1
C   1.53930 2.86929 0.60435
C   1.11479 1.57935 0.21432
C -0.21879 1.12257 0.49932
C -1.08075 2.00479 1.19636
C -0.64659 3.28550 1.58038
C   0.66168 3.72307 1.28513
C   1.94985 0.60494 -0.48609
C   3.28546 0.77058 -0.89931
C   3.95427 -0.26652 -1.55918
C   3.27142 -1.47355 -1.79749
C   1.94669 -1.59281 -1.37155
N   1.28511 -0.58575 -0.73954
Ru -0.65875 -0.67283 -0.16623
O -2.83867 -0.57479 -0.09227
C -2.85523 -0.12839 -1.30013
C -4.14459 0.22680 -1.99321
O -1.71516 0.03458 -1.89433
N -0.16774 -1.54819 1.49303
C   0.09944 -2.11770 2.49347
C   0.38649 -2.83689 3.72817
H   2.55728 3.20854 0.37855
H -0.38617 -2.62943 4.48802
H   0.41270 -3.92455 3.54427
H   1.36521 -2.53054 4.13383
H   3.79485 1.71745 -0.69940
H   4.99164 -0.13870 -1.88179
H   3.75219 -2.31412 -2.30436
H   1.36849 -2.50804 -1.53255
H   0.99172 4.72201 1.58767
H -1.33411 3.95185 2.11505
H -2.10264 1.68591 1.43170
H -4.07307 0.01697 -3.07107
H -4.98632 -0.32679 -1.55263
H -4.33830 1.30713 -1.87182

34
Int(3-4)bOAc
SCF(BP86)= -935.088770274
G(298 K) = -934.884597
SCF(PBE) = -934.365477252
Low Freq. = 16.5729cm-1, 33.5807cm-1
C   1.24133 -0.58976 -0.79804
C   1.89702 0.65884 -0.52102
C   3.20952 0.92046 -0.97447
C   3.90125 -0.05425 -1.70524
C   3.28019 -1.29196 -1.98077
C   1.97503 -1.55638 -1.53083
C   1.07632 1.58956 0.24739
C   1.43257 2.87464 0.69822
C   0.52867 3.64618 1.43621
C -0.74446 3.11870 1.71947
C -1.06711 1.84247 1.25433
N -0.18833 1.08753 0.53743
Ru -0.62529 -0.71671 -0.14017
O -2.98306 -0.56728 -0.11432
C -2.87100 -0.10970 -1.30051
O -1.68405 0.03669 -1.81666
N -0.03823 -1.60325 1.49328
C   0.26809 -2.17280 2.48161
C   0.59862 -2.89103 3.70565
C -4.08031 0.23402 -2.14105
H   2.42891 3.26039 0.46503

H   0.05201 -3.84872 3.74347
H   1.67951 -3.10446 3.75343
H   0.31917 -2.29583 4.59151
H   3.69267 1.88159 -0.76111
H   4.91826 0.14311 -2.05903
H   3.82376 -2.05575 -2.54994
H   1.51669 -2.52890 -1.75656
H   0.81029 4.64353 1.78598
H -1.48584 3.68315 2.29055
H -2.04258 1.38156 1.42863
H -3.85913 1.07279 -2.81817
H -4.35474 -0.63745 -2.76117
H -4.93758 0.48108 -1.49810

34
Int(3-4)c OAc
SCF(BP86)= -935.084339107
G(298 K) = -934.880472
SCF(PBE) = -934.360973290
Low Freq. = 23.2254cm-1,
30.3903cm-1
C -3.17779 1.71359 -0.22748
C -2.07247 0.83488 -0.22775
C -0.72332 1.32646 -0.16481
C -0.55293 2.72872 -0.05645
C -1.65618 3.60142 -0.05144
C -2.96988 3.09744 -0.14159
C -2.17406 -0.62593 -0.27946
C -3.35406 -1.39271 -0.30987
C -3.28539 -2.79082 -0.32317
C -2.02436 -3.41228 -0.30232
C -0.88072 -2.60918 -0.28299
N -0.93786 -1.25135 -0.28374
Ru 0.71382 -0.04175 -0.35365
O   2.69051 -1.17440 -0.94040
C   3.24468 -0.02372 -0.98059
C   4.70957 0.13503 -1.32920
O   2.54879 1.03356 -0.69052
N   0.98562 -0.15756 1.49893
C   1.16918 -0.22596 2.66403
C   1.41878 -0.32136 4.09724
H -4.20082 1.32447 -0.29418
H -4.32360 -0.88712 -0.31829
H -4.20155 -3.38826 -0.34531
H -1.92035 -4.50015 -0.30318
H   0.12655 -3.03596 -0.27494
H -3.82491 3.78119 -0.14041
H -1.49396 4.68341 0.02284
H   0.46075 3.14347 0.01246
H   1.94994 -1.25776 4.33952
H   2.03385 0.52753 4.44148
H   0.46710 -0.30771 4.65481
H   4.88159 1.07732 -1.87106
H   5.30233 0.16953 -0.39825
H   5.06008 -0.71733 -1.92893

34
Int(3-4)d OAc
SCF(BP86)= -935.081255503
G(298 K) = -934.876666
SCF(PBE) = -934.357550659
Low Freq. = 29.7429cm-1,
33.7510cm-1
C   0.34807 1.48420 0.09558
C   1.78588 1.36200 0.14580
C   2.60949 2.47199 0.43582
C   2.04013 3.72667 0.69151
C   0.63769 3.87025 0.65858
C -0.18842 2.77139 0.37005
C   2.30179 0.01388 -0.10999
C   3.65050 -0.38912 -0.08682
C   3.99279 -1.72356 -0.33664
C   2.97173 -2.65076 -0.60781
C   1.64712 -2.20456 -0.62152
N   1.30720 -0.90919 -0.38682
Ru -0.61695 -0.17059 -0.42881
N -2.42342 0.62300 -0.49655
C -3.51428 1.06769 -0.57507
C -4.85149 1.63686 -0.67931
O -1.58722 -2.35640 -0.39394
C -1.52556 -2.21038 0.86637
O -1.01577 -1.10580 1.34698
C -2.04072 -3.24197 1.84195
H   4.42904 0.34696 0.13086
H -4.83939 2.52565 -1.33263
H -5.55644 0.90128 -1.10184
H -5.21812 1.94145 0.31549
H   3.70007 2.36157 0.46298
H   2.68059 4.58534 0.91726
H   0.18722 4.84973 0.86021
H -1.27516 2.91325 0.35448
H   5.04092 -2.03666 -0.31675
H   3.19002 -3.70358 -0.80403
H   0.80790 -2.87687 -0.82107
H -1.65890 -3.05612 2.85606
H -3.14342 -3.20287 1.86648
H -1.74951 -4.24990 1.50833

34
Int(3-4)e OAc
SCF(BP86)= -935.072421602
G(298 K) = -934.867819
SCF(PBE) = -934.346393031
Low Freq. = 21.1062cm-1,
28.0338cm-1
C   3.67426 -0.29943 -0.20319
C   2.30256 0.03334 -0.14926
C   1.27649 -0.95149 -0.37083
C   1.71625 -2.27786 -0.62127
C   3.08223 -2.60860 -0.67053
C   4.06607 -1.62023 -0.46413
C   1.79973 1.38029 0.13881
C   2.57809 2.51854 0.41934
C   1.96932 3.74615 0.70501
C   0.56545 3.81846 0.70822
C -0.17007 2.66756 0.41811
N   0.41103 1.46748 0.13291
Ru -0.59824 -0.23642 -0.35003
O -1.52038 -2.24007 -0.37773
C -1.51414 -2.20904 0.90276
C -2.09205 -3.30188 1.75868
O -1.00486 -1.14268 1.45043

```
N  -2.50525  0.61762 -0.50028
C  -3.57597  1.07958 -0.67840
C  -4.89414  1.65773 -0.90692
H   4.44287  0.46610 -0.04220
H  -4.82342  2.50250 -1.61230
H  -5.57817  0.90291 -1.32908
H  -5.31998  2.02749  0.04068
H   3.66821  2.43321  0.41421
H   2.57743  4.62875  0.92374
H   0.03892  4.75092  0.92786
H  -1.26233  2.67256  0.40224
H   5.12913 -1.87968 -0.50393
H   3.38662 -3.64349 -0.87001
H   0.97009 -3.06569 -0.78351
H  -1.67797 -3.25868  2.77645
H  -3.18790 -3.18291  1.82124
H  -1.88383 -4.28392  1.30760

55
4a
SCF(BP86)= -1414.46686576
G(298 K) = -1414.105055
SCF(PBE) = -1413.34189112
Low Freq. = 6.4156cm-1, 26.9355cm-
1
C   4.41825 -1.02582 -0.55138
C   3.15016 -0.41702 -0.45804
N   2.12340 -1.05838  0.20817
C   2.34474 -2.28023  0.75871
C   3.58242 -2.92585  0.68738
C   4.64071 -2.28266  0.02122
C   2.75751  0.87649 -1.02215
C   3.63551  1.70059 -1.76306
C   3.17739  2.90128 -2.31895
C   1.82763  3.26881 -2.14445
C   0.95181  2.45627 -1.40431
C   1.38493  1.25063 -0.79220
Ru  0.34547 -0.02454  0.34375
N  -0.27903 -0.85752 -1.30442
C  -0.56801 -1.37826 -2.32253
C  -0.98726 -2.04037 -3.55214
H  -0.55137 -1.54072 -4.43350
H  -0.66566 -3.09583 -3.55656
H  -2.08668 -2.01020 -3.63852
H   4.67958  1.40189 -1.91482
H   3.85770  3.53860 -2.89302
H   1.45410  4.19603 -2.59619
H  -0.09223  2.77206 -1.30016
H   5.22665 -0.50950 -1.07605
H   5.62472 -2.75553 -0.05137
H   3.70721 -3.90939  1.14762
H   1.48791 -2.71777  1.27818
N  -1.34794  1.23426  0.59207
C  -1.04833  2.45134  1.14524
H  -0.00594  2.57259  1.44368
C  -1.96786  3.48720  1.30725
H  -1.63842  4.43006  1.75204
C  -3.28981  3.29268  0.87940
C  -3.62518  2.04358  0.34986
H  -4.04200  4.08095  0.97561
H  -4.65344  1.82353  0.05064
C  -2.66118  1.01826  0.23161
C  -3.12297 -0.31656 -0.24256
C  -3.95797 -0.40669 -1.37948
C  -4.48439 -1.64397 -1.78427
H  -5.12375 -1.69716 -2.67168
C  -4.19450 -2.80685 -1.05014
H  -4.60990 -3.77123 -1.36055
C  -3.37308 -2.72312  0.08804
H  -3.15265 -3.62381  0.67115
C  -2.83808 -1.49004  0.49137
H  -2.19740 -1.42778  1.37631
O  -0.24588 -1.23528  2.34176
C   0.41636 -0.33650  2.95980
H  -4.18289  0.49622 -1.95749
O   0.96911  0.62572  2.28929
C   0.59411 -0.38607  4.46362
H   1.31825 -1.17845  4.72079
H   0.96807  0.57184  4.85283
H  -0.36053 -0.64153  4.94969

55
4b
SCF(BP86)= -1414.46521132
G(298 K) = -1414.103559
SCF(PBE) = -1413.34058899
Low Freq. = 20.3478cm-1,
32.0030cm-1
C  -4.21134  0.43642 -1.26846
C  -3.03161  0.20122 -0.53190
N  -1.82167  0.70523 -0.98130
C  -1.78602  1.41492 -2.14080
C  -2.92598  1.66360 -2.90825
C  -4.16451  1.16827 -2.45860
C  -2.91134 -0.57351  0.70330
C  -4.00621 -1.19788  1.34618
C  -3.80613 -1.95627  2.50478
C  -2.49957 -2.09849  3.02068
C  -1.40775 -1.47945  2.39231
C  -1.56826 -0.68549  1.22439
Ru -0.21135  0.38970  0.26175
N   1.20436  0.12963  1.61670
C   2.01092  0.03328  2.47076
C   3.02741 -0.10823  3.50535
H   3.18890  0.85364  4.02022
H   2.72218 -0.85813  4.25456
H   3.98271 -0.42703  3.05496
H  -5.01942 -1.09264  0.94031
H  -4.65428 -2.43878  3.00108
H  -2.33557 -2.70082  3.92271
H  -0.40674 -1.60757  2.81928
H  -5.16249  0.04035 -0.90250
H  -5.07928  1.35090 -3.03040
H  -2.84052  2.23775 -3.83439
H  -0.80095  1.79567 -2.42275
N   0.41477 -1.28364 -0.84944
C  -0.55770 -2.15597 -1.27093
H  -1.58229 -1.85907 -1.04930
C  -0.30427 -3.37142 -1.90555
H  -1.14814 -4.00207 -2.19865
C   1.02329 -3.75798 -2.13598
C   2.02913 -2.86725 -1.74816
```

```
H   1.26782 -4.70798 -2.61931
H   3.08051 -3.09150 -1.94731
C   1.72135 -1.63639 -1.13259
C   2.85335 -0.70594 -0.86263
C   2.85887  0.60121 -1.39399
C   3.98659  1.42328 -1.24449
H   3.97979  2.43169 -1.67086
C   5.12171  0.95526 -0.56039
H   5.99969  1.59932 -0.44566
C   5.12738 -0.34733 -0.03192
H   6.00830 -0.72284  0.49914
C   4.00462 -1.17488 -0.18920
H   4.01154 -2.19023  0.22163
O   0.70057  2.53173 -0.44542
C   0.00001  3.01331  0.50196
H   1.98053  0.96965 -1.92929
O  -0.72401  2.22686  1.23719
C   0.01616  4.49594  0.82191
H   0.07320  5.08649 -0.10529
H  -0.87345  4.78765  1.39931
H   0.91224  4.72998  1.42299

55
4c
SCF(BP86)= -1414.46312567
G(298 K) = -1414.101297
SCF(PBE) = -1413.33910484
Low Freq. = 24.3966cm-1,
25.8405cm-1
C   3.64408  0.57789  0.73588
C   2.70007 -0.43460  0.45207
C   2.76242 -1.10240 -0.79178
C   3.75050 -0.75844 -1.72794
C   4.67914  0.25805 -1.44318
C   4.61939  0.92921 -0.21009
C   1.76123 -0.89386  1.51302
C   2.30485 -1.19339  2.78177
C   1.51260 -1.74593  3.79184
C   0.16971 -2.01881  3.49586
C  -0.32222 -1.68137  2.23432
N   0.42609 -1.09475  1.25126
Ru -0.74782 -0.45714 -0.42322
N  -1.89089  0.00410 -1.94681
H   3.37178 -1.01818  2.94250
H   1.93896 -1.98124  4.77139
H  -0.50030 -2.47900  4.22692
H  -1.36471 -1.86196  1.97192
H   3.60357  1.10025  1.69781
H   5.33588  1.72466  0.01937
H   5.44766  0.52315 -2.17671
H   3.79729 -1.29091 -2.68401
C  -2.56856  0.27186 -2.87400
C  -3.40394  0.59313 -4.02502
H  -3.65987  1.66585 -4.02380
H  -2.87482  0.36246 -4.96510
H  -4.34089  0.01170 -3.99870
N   0.19351  1.36581 -0.64502
C   1.10431  1.64749 -1.61293
H   1.38255  0.80741 -2.25318
C   1.68133  2.91013 -1.77315
H   2.41230  3.07027 -2.56963
C   1.31489  3.93952 -0.88874
C   0.35216  3.67247  0.09040
H   1.76084  4.93532 -0.97103
H   0.02149  4.46232  0.77079
C  -0.22527  2.39097  0.18766
C  -1.33688  2.00384  1.05746
C  -1.81160  0.66842  0.83076
C  -2.96835  0.27094  1.54688
H  -3.38455 -0.73199  1.38489
C  -3.59291  1.12943  2.46983
H  -4.48215  0.78843  3.01401
C  -3.08393  2.42311  2.70480
H  -3.56754  3.08507  3.43060
C  -1.95908  2.86142  1.99221
H  -1.57248  3.87337  2.16380
O  -1.69076 -2.39227 -0.30755
C  -0.92841 -3.00059 -1.15999
O   0.02512 -2.37266 -1.72665
H   2.03207 -1.88516 -1.01930
C  -1.21493 -4.45365 -1.49123
H  -1.78798 -4.93885 -0.68742
H  -0.27493 -4.99890 -1.66616
H  -1.80971 -4.50574 -2.42001

55
4d
SCF(BP86)= -1414.45935479
G(298 K) = -1414.097730
SCF(PBE) = -1413.33266234
Low Freq. = 21.0038cm-1,
26.4436cm-1
C   4.55654 -0.57461 -0.69641
C   3.21857 -0.15201 -0.52061
C   2.24087 -0.99148  0.11251
C   2.67817 -2.27388  0.53106
C   4.00771 -2.69519  0.35115
C   4.95304 -1.84535 -0.25953
C   2.70179  1.13957 -0.97877
C   3.43165  2.12647 -1.67031
C   2.80666  3.29784 -2.10990
C   1.43283  3.46085 -1.86160
C   0.75075  2.46214 -1.16270
N   1.35556  1.33439 -0.69634
Ru  0.43690 -0.15127  0.37444
N  -0.19653 -0.87426 -1.31159
C  -0.52075 -1.32051 -2.35476
C  -0.98147 -1.89904 -3.61174
H  -0.43170 -2.82803 -3.84000
H  -2.05734 -2.13422 -3.54571
H  -0.82822 -1.19282 -4.44513
H   4.49386  1.96105 -1.87019
H   3.37530  4.06258 -2.64711
H   0.88789  4.34499 -2.20276
H  -0.31674  2.54585 -0.95077
H   5.29275  0.08264 -1.17469
H   5.98840 -2.17496 -0.39536
H   4.31426 -3.69270  0.68968
H   1.96418 -2.94896  1.01876
N  -1.44291  1.09066  0.85334
C  -1.12267  2.20874  1.56646
H  -0.09198  2.24301  1.92902
```

C -2.00604 3.26053 1.82203
H -1.66584 4.12540 2.39798
C -3.31287 3.17552 1.31855
C -3.67392 2.02108 0.61813
H -4.03867 3.97697 1.48567
H -4.69585 1.88926 0.25241
C -2.73735 0.98261 0.40966
C -3.20226 -0.26472 -0.25813
C -2.89891 -1.53337 0.28468
C -3.42770 -2.69608 -0.29544
H -3.19385 -3.67281 0.14157
C -4.25982 -2.61372 -1.42645
H -4.67236 -3.52368 -1.87446
C -4.56202 -1.35602 -1.97639
H -5.20510 -1.28059 -2.85939
C -4.04216 -0.18980 -1.39271
H -4.27645 0.78708 -1.82941
O -0.13553 -1.58509 1.94128
C 0.43604 -0.85000 2.82860
H -2.24501 -1.60005 1.15920
O 0.98630 0.24994 2.43260
C 0.49695 -1.26958 4.27622
H 1.41002 -1.86684 4.44632
H 0.53953 -0.38852 4.93368
H -0.37166 -1.89327 4.53449

55
4e
SCF(BP86)= -1414.45286826
G(298 K) = -1414.091318
SCF(PBE) = -1413.32829808
Low Freq. = 12.9996cm-1,
29.9988cm-1
C 1.43842 -3.54083 -0.98165
C 0.98373 -2.20406 -0.91890
C 1.67848 -1.20664 -0.15523
C 2.85604 -1.62702 0.51309
C 3.31171 -2.95695 0.44905
C 2.60058 -3.91966 -0.29464
C -0.19085 -1.70342 -1.63839
C -0.94642 -2.42383 -2.58291
C -1.99190 -1.80931 -3.27992
C -2.26613 -0.45616 -3.01373
C -1.50521 0.21513 -2.05586
N -0.48794 -0.37234 -1.36261
Ru 0.81972 0.62363 -0.16230
N -0.06051 2.52205 -0.28634
C -0.44507 3.63105 -0.39553
C -0.93157 4.99888 -0.53538
H -0.15004 5.63929 -0.97685
H -1.81982 5.02871 -1.18818
H -1.20553 5.41118 0.44990
H -0.68979 -3.46817 -2.78234
H -2.57309 -2.36791 -4.01952
H -3.06630 0.07942 -3.53162
H -1.69619 1.25928 -1.80398
H 0.88903 -4.29207 -1.56200
H 2.95243 -4.95579 -0.34025
H 4.22621 -3.24797 0.98074
H 3.43383 -0.89570 1.09221
N 0.01570 0.15445 1.71342

C 0.98997 0.20339 2.67709
H 1.94282 0.62070 2.34310
C 0.82432 -0.26206 3.98027
H 1.65777 -0.18613 4.68376
C -0.40331 -0.83655 4.34530
C -1.42194 -0.85174 3.38880
H -0.56932 -1.23763 5.34917
H -2.41533 -1.23728 3.63414
C -1.21521 -0.32736 2.09404
C -2.40436 -0.23169 1.19957
C -2.89518 1.03335 0.80832
C -4.11211 1.14043 0.11844
H -4.48840 2.12854 -0.16670
C -4.85158 -0.01278 -0.19650
H -5.80223 0.07292 -0.73288
C -4.36953 -1.27535 0.18712
H -4.94029 -2.17848 -0.05264
C -3.15940 -1.38395 0.89088
H -2.79326 -2.36742 1.20448
O 2.67380 1.63254 0.47327
C 3.03014 1.74626 -0.75911
H -2.32569 1.93079 1.06459
O 2.23092 1.29199 -1.66494
C 4.35666 2.35995 -1.13497
H 5.14325 1.58601 -1.09508
H 4.32318 2.76288 -2.15787
H 4.62865 3.15516 -0.42461

61
5a
SCF(BP86)= -1547.22367196
G(298 K) = -1546.820984
SCF(PBE) = -1545.99804503
Low Freq. = 11.5975cm-1,
15.9568cm-1
C -3.46086 -1.07118 0.86705
C -2.55482 -0.02117 1.13706
C -2.85272 1.28120 0.68172
C -4.02916 1.51858 -0.04705
C -4.91617 0.46626 -0.33170
C -4.62814 -0.83083 0.12701
C -1.40763 -0.24769 2.06141
C -1.72676 -0.75705 3.34081
C -0.75492 -0.87278 4.33672
C 0.54387 -0.44944 4.02428
C 0.81130 0.00461 2.73293
N -0.11663 0.09382 1.73093
Ru 0.77802 0.39237 -0.23461
N 1.68694 0.54977 -1.98053
H -2.76834 -1.02041 3.54278
H -1.00858 -1.25837 5.32849
H 1.35485 -0.48172 4.75679
H 1.82294 0.29788 2.45782
H -3.24124 -2.08051 1.23163
H -5.31592 -1.65582 -0.08580
H -5.83278 0.65712 -0.89973
H -4.25929 2.53466 -0.38516
C 2.21520 0.63718 -3.02710
C 2.87732 0.74948 -4.32132
H 2.61892 -0.11290 -4.95775
H 2.56246 1.67223 -4.83632

```
H   3.97207  0.77424 -4.19224
N  -0.56475 -0.86575 -1.20015
C  -1.58300 -0.41957 -1.97612
H  -1.72531  0.66446 -1.95913
C  -2.38756 -1.28206 -2.72532
H  -3.19677 -0.87425 -3.33615
C  -2.13747 -2.66394 -2.66074
C  -1.07631 -3.12652 -1.87585
H  -2.75441 -3.37063 -3.22442
H  -0.84649 -4.19459 -1.83438
C  -0.27345 -2.21348 -1.16085
C   0.93862 -2.53704 -0.39806
C   1.66673 -1.40034  0.09771
C   2.88950 -1.67782  0.75820
H   3.49790 -0.84610  1.13530
C   3.35374 -2.99203  0.94829
H   4.30444 -3.16437  1.46784
C   2.60540 -4.08924  0.47863
H   2.96225 -5.11271  0.63386
C   1.40054 -3.85831 -0.19870
H   0.82286 -4.71245 -0.57193
O  -0.30865  2.21864 -0.95869
C  -0.50105  3.34070 -0.33386
O  -0.35634  3.55462  0.89695
H  -2.17074  2.10048  0.92826
C  -0.97828  4.48674 -1.24919
H  -0.21181  4.71024 -2.01152
H  -1.18396  5.39912 -0.66923
H  -1.89174  4.18761 -1.79115
N   2.20988  1.51983  0.55041
C   3.09268  2.20032  0.93275
C   4.16210  3.06803  1.41117
H   4.76427  3.43296  0.56240
H   3.74260  3.94004  1.94085
H   4.82816  2.52421  2.10179
```

61
5b
SCF(BP86)= -1547.22282483
G(298 K) = -1546.820141
SCF(PBE) = -1545.99440633
Low Freq. = 24.3885cm-1,
29.5952cm-1

```
C   3.93080  0.71893 -1.38464
C   3.18280  0.31037 -0.25615
C   3.19385 -1.05107  0.11433
C   3.93150 -1.98174 -0.63295
C   4.66601 -1.57077 -1.75890
C   4.66043 -0.21638 -2.13532
C   2.53016  1.34236  0.59993
C   3.35341  2.41327  1.01891
C   2.88240  3.39677  1.89048
C   1.56087  3.27705  2.34192
C   0.78292  2.21894  1.87455
N   1.21256  1.25286  1.00189
Ru -0.37849 -0.03716  0.27715
N  -1.46659  0.27085  1.91828
H   4.38846  2.43137  0.66779
H   3.52887  4.21615  2.21761
H   1.11918  3.99553  3.03767
H  -0.25836  2.13640  2.18165
H   3.93040  1.77374 -1.68028
H   5.22893  0.11515 -3.01058
H   5.24226 -2.30009 -2.33773
H   3.93588 -3.03441 -0.33100
C  -2.18456  0.35503  2.84614
C  -3.06401  0.45523  4.00376
H  -3.78177  1.28132  3.87033
H  -3.62748 -0.48303  4.13815
H  -2.47402  0.64469  4.91572
N  -2.06996 -1.02696 -0.44439
C  -2.30075 -2.35910 -0.31789
H  -1.46829 -2.93810  0.10804
C  -3.49816 -2.95151 -0.73588
H  -3.63620 -4.02906 -0.61220
C  -4.49599 -2.14308 -1.30460
C  -4.25013 -0.77543 -1.45820
H  -5.44785 -2.57275 -1.63209
H  -5.00489 -0.12791 -1.91214
C  -3.02437 -0.22189 -1.03370
C  -2.61372  1.17712 -1.19114
C  -1.30550  1.48571 -0.68588
C  -0.83395  2.80057 -0.92887
H   0.17059  3.08661 -0.59793
C  -1.62080  3.76425 -1.58483
H  -1.21972  4.77194 -1.74896
C  -2.91737  3.44637 -2.03461
H  -3.53132  4.19908 -2.53986
C  -3.40786  2.14878 -1.84350
H  -4.40730  1.89347 -2.21482
O   0.52207 -1.65020  1.63120
C   0.61207 -2.94292  1.61046
O   0.28751 -3.71046  0.66421
H   2.60214 -1.36551  0.97825
C   1.16694 -3.55789  2.91060
H   2.05374 -3.00590  3.26336
H   1.42408 -4.61847  2.76737
H   0.40480 -3.48734  3.70708
N   0.51553 -0.48982 -1.43522
C   0.88406 -0.78145 -2.51257
C   1.37194 -1.15156 -3.83444
H   1.50513 -2.24417 -3.90238
H   0.65689 -0.83486 -4.61164
H   2.34403 -0.66806 -4.02520
```

61
5c
SCF(BP86)= -1547.21857774
G(298 K) = -1546.815949
SCF(PBE) = -1545.99121931
Low Freq. = 13.3194cm-1,
27.2501cm-1

```
C   3.67780  1.01254  0.19906
C   2.75342  0.33875  1.02783
C   2.96676 -1.02563  1.31876
C   4.06197 -1.70773  0.76580
C   4.96274 -1.03532 -0.07832
C   4.76856  0.32792 -0.35816
C   1.69640  1.11188  1.74290
C   2.15163  2.14316  2.59396
C   1.26688  2.85293  3.40952
C  -0.08553  2.49005  3.36041
```

```
C  -0.48537  1.49020  2.47394
N   0.35758  0.80473  1.63892
Ru -0.66988 -0.36416  0.12509
N   0.25399 -2.23433  0.43834
H   3.22534  2.34701  2.62275
H   1.62455  3.64550  4.07305
H  -0.83854  2.97980  3.98389
H  -1.53749  1.22460  2.39262
H   3.53098  2.07640 -0.01551
H   5.46972  0.86181 -1.00774
H   5.81844 -1.56838 -0.50539
H   4.22179 -2.76338  1.00882
C   0.62275 -3.34776  0.52407
C   1.07835 -4.72949  0.61905
H   2.08119 -4.83514  0.17351
H   1.12679 -5.04625  1.67386
H   0.38275 -5.39731  0.08417
N   0.58059  0.24310 -1.42876
C   1.59237 -0.50123 -1.94143
H   1.82805 -1.41429 -1.39096
C   2.30822 -0.13127 -3.08310
H   3.11262 -0.77478 -3.44792
C   1.97923  1.07525 -3.72273
C   0.93237  1.84629 -3.20762
H   2.52377  1.40547 -4.61266
H   0.64230  2.77809 -3.70025
C   0.22170  1.41335 -2.06916
C  -0.95412  2.07592 -1.49179
C  -1.58793  1.36325 -0.41784
C  -2.77538  1.94110  0.09829
H  -3.32030  1.42669  0.90002
C  -3.29157  3.15567 -0.38847
H  -4.21350  3.56753  0.04040
C  -2.63208  3.84718 -1.42301
H  -3.02764  4.79682 -1.79824
C  -1.46580  3.30163 -1.97620
H  -0.95917  3.83554 -2.78900
O  -1.68072 -1.27263 -1.50250
C  -2.48716 -2.30532 -1.47822
O  -2.83952 -2.97790 -0.48802
H   2.28359 -1.54129  1.99771
C  -3.04380 -2.63982 -2.87618
H  -3.85964 -1.93851 -3.12612
H  -3.45069 -3.66216 -2.89192
H  -2.27121 -2.53444 -3.65457
N  -2.02661 -0.86080  1.47585
C  -2.82306 -1.15257  2.29327
C  -3.84016 -1.56061  3.25466
H  -4.43264 -2.39762  2.84724
H  -3.37413 -1.89056  4.19819
H  -4.52421 -0.72479  3.47803

61
5d
SCF(BP86)= -1547.22031336
G(298 K) = -1546.818388
SCF(PBE) = -1545.99158619
Low Freq. = 17.1837cm-1,
18.8873cm-1
C  -4.45194 -0.34116  0.43582
C  -3.41279  0.03096 -0.44946
C  -3.08875 -0.83042 -1.51939
C  -3.80373 -2.02347 -1.70979
C  -4.82814 -2.38970 -0.82072
C  -5.14497 -1.54820  0.26019
C  -2.78382  1.37606 -0.34173
C  -3.63879  2.49179 -0.18123
C  -3.12297  3.79023 -0.20984
C  -1.74553  3.94804 -0.41953
C  -0.95586  2.80023 -0.53446
N  -1.42824  1.52535 -0.47366
Ru  0.37366  0.02845  0.26389
N   0.88017  1.55102  1.40926
H  -4.71408  2.32227 -0.07880
H  -3.78283  4.65594 -0.09797
H  -1.28123  4.93576 -0.48435
H   0.12579  2.89532 -0.65766
H  -4.71053  0.31829  1.27143
H  -5.93943 -1.82574  0.96068
H  -5.38096 -3.32255 -0.97234
H  -3.56519 -2.66599 -2.56362
C   1.22635  2.42881  2.11593
C   1.64265  3.51958  2.99008
H   2.68451  3.37277  3.32058
H   1.57588  4.48683  2.46413
H   0.99715  3.56294  3.88332
N   1.75455  0.62447 -1.16224
C   1.47023  1.30668 -2.29965
H   0.41021  1.50230 -2.48000
C   2.45227  1.73011 -3.19970
H   2.16115  2.27674 -4.10038
C   3.79678  1.43955 -2.91231
C   4.09677  0.71116 -1.75649
H   4.59657  1.76586 -3.58401
H   5.13344  0.45331 -1.52364
C   3.06679  0.28729 -0.89332
C   3.22928 -0.56240  0.28891
C   2.00696 -0.90826  0.95993
C   2.11035 -1.79900  2.05592
H   1.20410 -2.09393  2.59733
C   3.35099 -2.31217  2.47580
H   3.39346 -3.00003  3.32932
C   4.53967 -1.94615  1.81379
H   5.50520 -2.34163  2.14590
C   4.47459 -1.07255  0.71978
H   5.39888 -0.79740  0.19787
O  -0.27601 -1.63762 -0.82735
C   0.38894 -2.37905 -1.68059
O   1.51387 -2.15052 -2.17039
H  -2.28892 -0.54978 -2.20809
C  -0.34913 -3.67892 -2.04438
H   0.00389 -4.48786 -1.37943
H  -0.11657 -3.97526 -3.07941
H  -1.43744 -3.58571 -1.91316
N  -0.84206 -0.64372  1.69131
C  -1.55423 -1.04659  2.53714
C  -2.44379 -1.55389  3.57433
H  -3.46079 -1.68788  3.16899
H  -2.08408 -2.52561  3.95223
H  -2.49207 -0.84644  4.41877

61
```

5e
SCF(BP86)= -1547.21879144
G(298 K) = -1546.816609
SCF(PBE) = -1545.99011825
Low Freq. = 7.5412cm-1, 18.2228cm-1

```
C  -3.90786 -1.19148  0.82775
C  -3.01315 -0.95141 -0.24045
C  -2.38778 -2.05489 -0.85767
C  -2.64378 -3.36195 -0.41610
C  -3.53078 -3.58951  0.65055
C  -4.15919 -2.49793  1.27433
C  -2.86777  0.43788 -0.76900
C  -4.06373  1.11827 -1.09503
C  -4.02834  2.40441 -1.64025
C  -2.77246  2.98670 -1.85656
C  -1.63009  2.26660 -1.49627
N  -1.63717  1.01764 -0.95113
Ru  0.49993  0.50762 -0.01813
N   1.40182  1.77144 -1.25714
H  -5.01657  0.60627 -0.93538
H  -4.95296  2.92885 -1.89952
H  -2.66648  3.98531 -2.28942
H  -0.64312  2.71078 -1.63101
H  -4.40126 -0.34447  1.31604
H  -4.85021 -2.66267  2.10746
H  -3.73546 -4.61057  0.98889
H  -2.15826 -4.20636 -0.91652
C   2.01346  2.47899 -1.97262
C   2.76787  3.36013 -2.85518
H   3.83045  3.06586 -2.87248
H   2.37240  3.30928 -3.88332
H   2.69912  4.40280 -2.50312
N   1.16498 -1.01082 -1.25441
C   0.61859 -1.34072 -2.45398
H  -0.24840 -0.74374 -2.74923
C   1.11765 -2.36410 -3.26418
H   0.62917 -2.58643 -4.21639
C   2.23860 -3.08766 -2.82031
C   2.84413 -2.71855 -1.61481
H   2.64604 -3.91024 -3.41558
H   3.74328 -3.23624 -1.26970
C   2.32157 -1.65562 -0.84910
C   2.94052 -1.06495  0.34277
C   2.27406  0.10030  0.84819
C   2.88870  0.77756  1.92694
H   2.40769  1.67788  2.32678
C   4.08643  0.31170  2.49858
H   4.53589  0.85324  3.34012
C   4.71174 -0.85166  2.00472
H   5.63969 -1.21644  2.45743
C   4.14091 -1.53638  0.92402
H   4.63550 -2.43454  0.53512
O   0.15724  2.24522  1.16324
C  -0.61330  2.42654  2.20342
O  -1.34334  1.58234  2.76720
H  -1.71282 -1.89165 -1.69996
C  -0.58646  3.87939  2.71709
H   0.41938  4.32031  2.63096
H  -0.92471  3.92393  3.76364
H  -1.27153  4.49780  2.10999
N  -0.22848 -0.80578  1.28243
C  -0.49709 -1.62432  2.08027
C  -0.85368 -2.59832  3.10327
H  -1.50368 -2.13158  3.86230
H  -1.39455 -3.44648  2.65325
H   0.05283 -2.97781  3.60346
```

61
5f
SCF(BP86)= -1547.21798662
G(298 K) = -1546.815288
SCF(PBE) = -1545.98920490
Low Freq. = 20.3143cm-1, 21.3481cm-1

```
C  -3.87195  0.33597  1.48204
C  -3.19766  0.23110  0.24422
C  -3.36944 -0.93981 -0.52288
C  -4.17112 -1.99077 -0.05082
C  -4.82127 -1.88621  1.19044
C  -4.66939 -0.71683  1.95553
C  -2.51776  1.43138 -0.32774
C  -3.32234  2.58872 -0.44598
C  -2.85645  3.73738 -1.08865
C  -1.56205  3.69589 -1.62457
C  -0.79556  2.54573 -1.44081
N  -1.21732  1.41830 -0.78547
Ru  0.39320  0.00718 -0.37664
N   1.38591  0.57118 -1.98298
H  -4.34188  2.54549 -0.05381
H  -3.48961  4.62405 -1.18568
H  -1.13031  4.54413 -2.16262
H   0.23092  2.51170 -1.80392
H  -3.76034  1.24729  2.07930
H  -5.17699 -0.62048  2.92105
H  -5.45032 -2.70521  1.55427
H  -4.30056 -2.88917 -0.66364
C   2.03373  0.82202 -2.93709
C   2.82440  1.13895 -4.12124
H   3.53343  1.95609 -3.90665
H   3.39903  0.25687 -4.45072
H   2.16808  1.45555 -4.94938
N   2.07936 -1.09383  0.12554
C   2.33550 -2.35211 -0.31434
H   1.54442 -2.81056 -0.90742
C   3.52038 -3.03172 -0.02320
H   3.66961 -4.04393 -0.40827
C   4.49042 -2.39194  0.76785
C   4.23450 -1.09712  1.22654
H   5.42957 -2.89377  1.02000
H   4.97359 -0.57503  1.83977
C   3.02548 -0.44856  0.89636
C   2.63781  0.90441  1.30835
C   1.36820  1.35403  0.80884
C   0.94756  2.63296  1.25456
H  -0.02475  3.02661  0.93831
C   1.73399  3.42777  2.10820
H   1.36595  4.41074  2.42739
C   2.98644  2.96990  2.56034
H   3.60057  3.58873  3.22291
C   3.43254  1.70347  2.16304
H   4.39788  1.33655  2.53125
```

```
O  -0.58608 -0.52528  1.40470
C  -0.63325 -1.73401  1.90446
O  -0.24227 -2.78980  1.36131
H  -2.88993 -1.01321 -1.50068
C  -1.22356 -1.76247  3.32442
H  -2.17031 -1.20019  3.36182
H  -0.52389 -1.27910  4.02894
H  -1.39500 -2.79868  3.65253
N  -0.41393 -1.51597 -1.60188
C  -0.71556 -2.33510 -2.39147
C  -1.10311 -3.36772 -3.34599
H  -0.23858 -3.66540 -3.96213
H  -1.47794 -4.25750 -2.81352
H  -1.89785 -2.99621 -4.01381

61
5g
SCF(BP86)= -1547.21648399
G(298 K) = -1546.813822
SCF(PBE) = -1545.98820007
Low Freq. = 19.3868cm-1,
22.7928cm-1
C  -3.50424  0.82733 -1.16638
C  -2.53893 -0.17134 -1.42227
C  -2.83597 -1.50981 -1.08787
C  -4.06154 -1.83716 -0.48680
C  -5.00762 -0.83381 -0.21566
C  -4.72519  0.49962 -0.55704
C  -1.31804  0.16138 -2.21454
C  -1.53695  0.66151 -3.51679
C  -0.46801  0.89993 -4.38455
C   0.82110  0.61272 -3.91838
C   0.98757  0.15321 -2.60956
N  -0.04793 -0.05934 -1.73794
Ru  0.64924 -0.40511  0.28335
N   1.47285 -0.63045  2.06331
H  -2.56876  0.82657 -3.83833
H  -0.64018  1.28102 -5.39564
H   1.70490  0.75465 -4.54658
H   1.99057 -0.03543 -2.21224
H  -3.29000  1.86536 -1.44210
H  -5.45932  1.28652 -0.35567
H  -5.96465 -1.09164  0.24957
H  -4.28404 -2.88146 -0.24367
C   1.96338 -0.75987  3.12488
C   2.58807 -0.92240  4.43253
H   2.40092 -0.03429  5.05861
H   2.17996 -1.80702  4.94904
H   3.67813 -1.05047  4.32460
N  -0.84974  0.65260  1.21317
C  -1.91646  0.07413  1.82786
H  -2.00471 -1.00489  1.69420
C  -2.85482  0.79813  2.56564
H  -3.69012  0.27222  3.03473
C  -2.70412  2.19118  2.67383
C  -1.60471  2.79468  2.05661
H  -3.42460  2.79298  3.23594
H  -1.44880  3.87337  2.14300
C  -0.66893  2.01798  1.34305
C   0.57062  2.50789  0.73620
C   1.41959  1.48965  0.18375
C   2.65551  1.93056 -0.35319
H   3.33078  1.18307 -0.78804
C   3.01935  3.29041 -0.36077
H   3.98443  3.59162 -0.78808
C   2.15803  4.27051  0.16973
H   2.44050  5.32873  0.15642
C   0.93277  3.87540  0.72038
H   0.26117  4.63546  1.13727
O   2.23155 -1.71342 -0.45504
C   3.38165 -1.65630 -1.05822
O   3.89262 -0.66461 -1.63907
H  -2.11124 -2.29314 -1.32222
C   4.14653 -2.99220 -1.03307
H   4.41148 -3.25530  0.00566
H   5.06699 -2.92767 -1.63221
H   3.51371 -3.80782 -1.42132
N  -0.22748 -2.32482  0.53932
C  -0.59122 -3.41373  0.79579
C  -1.06072 -4.75770  1.11274
H  -0.84694 -4.99732  2.16744
H  -2.14838 -4.83236  0.94866
H  -0.55588 -5.50112  0.47427

60
2a 2ppy-H
SCF(BP86)= -1451.35856945
G(298 K) = -1450.957544
SCF(PBE) = -1450.17083537
Low Freq. = 18.4629cm-1,
23.8745cm-1
C   2.13778 -1.11643 -0.08327
C   3.01120 -0.41662 -0.97584
C   4.25403 -0.96705 -1.36747
C   4.64927 -2.22193 -0.88891
C   3.79816 -2.93018 -0.01909
C   2.56343 -2.38314  0.37503
C   2.51309  0.87441 -1.46205
N   1.27218  1.22770 -0.97088
C   0.68954  2.37259 -1.40760
C   1.30284  3.23733 -2.31762
C   2.57839  2.90642 -2.80375
C   3.17766  1.71920 -2.37521
Ru  0.42989 -0.12636  0.39491
N  -0.43849 -1.03042 -1.15907
C  -0.81449 -1.58748 -2.12044
C  -1.28191 -2.27900 -3.31359
N  -0.19391 -1.52034  1.68174
C  -0.49186 -2.33507  2.47456
C  -0.86087 -3.35189  3.45068
N   1.45891  0.69604  1.89613
C   2.13539  1.10345  2.76578
C   2.96897  1.61412  3.84505
H   4.91651 -0.42043 -2.04798
H  -1.85473 -3.17630 -3.02937
H  -1.93677 -1.61814 -3.90417
H  -0.42475 -2.58400 -3.93607
H   4.16289  1.43431 -2.75214
H   3.09419  3.56076 -3.51268
H   0.78476  4.14675 -2.63140
H  -0.30097  2.58723 -1.00344
H   5.61119 -2.64725 -1.19235
```

```
H   4.09979 -3.91536  0.35588
H   1.92411 -2.95650  1.05503
H  -1.77246 -3.87922  3.12506
H  -0.04825 -4.08850  3.56424
H  -1.05516 -2.88597  4.43071
H   2.35600  1.80670  4.74076
H   3.75066  0.88016  4.10094
H   3.45369  2.55516  3.53757
N  -1.42327  1.31291  0.86403
C  -1.04237  2.45291  1.50668
C  -1.83665  3.59589  1.63239
C  -3.11232  3.59198  1.05213
C  -3.53911  2.42256  0.41742
C  -2.69662  1.28930  0.35391
H  -0.03250  2.44429  1.91973
H  -1.44750  4.46756  2.16505
H  -3.76570  4.46753  1.10944
H  -4.54308  2.35277 -0.00969
C  -3.26330  0.04496 -0.23739
C  -3.90135  0.08877 -1.49744
C  -4.52469 -1.05471 -2.02026
C  -4.53396 -2.25195 -1.28342
C  -3.91630 -2.29842 -0.02157
C  -3.28500 -1.15861  0.49880
H  -3.89594  1.02013 -2.07376
H  -5.00740 -1.00874 -3.00179
H  -5.02892 -3.14140 -1.68640
H  -3.93641 -3.22272  0.56500
H  -2.82010 -1.18863  1.48707

60
2b 2ppy-H
SCF(BP86)= -1451.35601509
G(298 K) = -1450.954685
SCF(PBE) = -1450.16812148
Low Freq. = 15.3167cm-1,
27.3033cm-1
C  -1.85369  0.63057  1.12354
C  -3.05067 -0.03985  0.69570
C  -4.25966  0.07138  1.42230
C  -4.30832  0.84156  2.59002
C  -3.14357  1.49618  3.03662
C  -1.94278  1.39253  2.31224
C  -2.90693 -0.88119 -0.49896
N  -1.62911 -0.90361 -1.02546
C  -1.36215 -1.69746 -2.09403
C  -2.33370 -2.49306 -2.70517
C  -3.64353 -2.46521 -2.19616
C  -3.92435 -1.65909 -1.08984
Ru -0.27411  0.41533 -0.14280
N  -1.08935  1.85802 -1.24879
C  -1.57842  2.69823 -1.90515
C  -2.18306  3.74011 -2.72455
N   0.78429  1.84038  0.77704
C   1.29304  2.78526  1.25530
C   1.92949  3.94617  1.86297
N   1.17034  0.21793 -1.70303
C   1.84415  0.23864 -2.66428
C   2.69453  0.25262 -3.84667
H  -5.16491 -0.44397  1.08206
H  -1.42133  4.47883 -3.02280
H  -2.62414  3.29958 -3.63366
H  -2.97641  4.25651 -2.15994
H   2.27379 -0.39986 -4.62894
H   2.77399  1.27754 -4.24401
H   3.70354 -0.10603 -3.58562
H  -4.93442 -1.63015 -0.67376
H  -4.43346 -3.06791 -2.65389
H  -2.06301 -3.11426 -3.56241
H  -0.33206 -1.67134 -2.45571
H  -5.24349  0.92782  3.15239
H  -3.17428  2.09374  3.95547
H  -1.05722  1.92108  2.68247
H   1.55657  4.87169  1.39412
H   1.70737  3.98437  2.94219
H   3.02244  3.89925  1.72695
N   0.53842 -1.27300  0.96087
C   1.83948 -1.72369  0.98610
C   2.15312 -3.02138  1.44418
C   1.16298 -3.87351  1.94019
C  -0.14775 -3.38395  1.98839
C  -0.40799 -2.10550  1.49399
C   3.00177 -0.86753  0.60044
H   3.20144 -3.33045  1.42950
H   1.41284 -4.87627  2.29837
H  -0.97267 -3.97612  2.39287
H  -1.42317 -1.70955  1.51226
C   3.37111  0.24045  1.39172
C   4.55783  0.94194  1.12269
C   5.39233  0.54389  0.06520
C   5.04388 -0.57352 -0.71339
C   3.86357 -1.28156 -0.43988
H   2.74197  0.53046  2.23739
H   4.84185  1.78918  1.75541
H   6.31881  1.08979 -0.13970
H   5.69911 -0.90370 -1.52610
H   3.60123 -2.16108 -1.03729

60
2c 2ppy-H
SCF(BP86)= -1451.35562273
G(298 K) = -1450.954315
SCF(PBE) = -1450.16729514
Low Freq. = 14.6907cm-1,
20.1382cm-1
C  -1.30568  1.27144 -0.86958
C  -2.58989  0.84023 -1.33973
C  -3.38501  1.65530 -2.17884
C  -2.91819  2.91051 -2.58662
C  -1.64737  3.34326 -2.16379
C  -0.85949  2.53461 -1.32386
C  -2.99161 -0.50957 -0.92802
N  -2.06089 -1.16768 -0.15128
C  -2.30587 -2.43959  0.25084
C  -3.48007 -3.12252 -0.07616
C  -4.44695 -2.46232 -0.85241
C  -4.19606 -1.15516 -1.27800
Ru -0.35845 -0.06894  0.35219
N   0.48464 -0.84274 -1.28171
C   0.80216 -1.35198 -2.29002
C   1.20735 -1.97725 -3.54113
N  -1.40203  0.57629  1.93265
```

C -2.08719 0.85832 2.84425
C -2.92686 1.21594 3.97917
N 0.40623 -1.56851 1.66125
C 0.69827 -2.37809 2.46142
C 1.06997 -3.38461 3.44761
H -4.36676 1.31154 -2.52357
H 1.36367 -3.05859 -3.39505
H 0.42899 -1.83170 -4.30807
H 2.14977 -1.53007 -3.89656
H 1.45201 -4.28650 2.94234
H 1.85443 -2.99243 4.11523
H 0.19361 -3.66313 4.05531
H -4.93204 -0.62611 -1.88826
H -5.38156 -2.96148 -1.12477
H -3.62481 -4.14692 0.27557
H -1.52465 -2.90383 0.85549
H -3.53293 3.54172 -3.23627
H -1.26600 4.31723 -2.49281
H 0.12615 2.90607 -1.02338
H -2.30231 1.41688 4.86504
H -3.51578 2.11852 3.74777
H -3.61963 0.39126 4.21408
N 1.26275 1.32961 0.79141
C 2.57627 1.29874 0.38022
C 3.43431 2.40255 0.58230
C 2.99463 3.54979 1.24629
C 1.67119 3.56420 1.70462
C 0.85825 2.45992 1.44982
C 3.18839 0.10451 -0.27537
H 4.46475 2.32250 0.22660
H 3.66595 4.39785 1.40839
H 1.25329 4.42031 2.24056
H -0.18542 2.47174 1.75951
C 3.75690 0.23969 -1.56180
C 4.44765 -0.83046 -2.15189
C 4.59385 -2.04391 -1.45735
C 4.05271 -2.17677 -0.16743
C 3.35884 -1.10883 0.42261
H 3.64850 1.18623 -2.10194
H 4.87917 -0.71244 -3.15129
H 5.13889 -2.87638 -1.91381
H 4.18416 -3.11044 0.38917
H 2.96257 -1.20493 1.43555

60
TS(2a-3e)2ppy
SCF(BP86)= -1451.32080930
G(298 K) = -1450.921687
SCF(PBE) = -1450.13221374
Low Freq. = -82.6211cm-1,
11.1917cm-1
C -2.15533 0.02252 0.71322
C -2.87776 0.44873 -0.44783
C -4.19960 0.93765 -0.34933
C -4.83268 1.00601 0.89896
C -4.14235 0.58306 2.05041
C -2.82661 0.09262 1.95459
C -2.13939 0.36333 -1.71223
N -0.85337 -0.12292 -1.58457
C -0.07248 -0.23898 -2.68832
C -0.50973 0.12133 -3.96547
C -1.81501 0.61904 -4.11212
C -2.62700 0.73798 -2.98025
Ru -0.27447 -0.63004 0.35369
N -0.43060 2.68632 0.11006
C -0.81196 3.79639 0.10509
C -1.29090 5.17561 0.09778
N 0.14941 -1.02768 2.26391
C 0.38181 -1.29976 3.38435
C 0.66651 -1.61921 4.77627
N -1.01214 -2.36443 0.13993
C -1.52493 -3.41749 0.01991
C -2.13978 -4.72891 -0.13896
H -4.74050 1.26880 -1.24303
H -1.11818 5.64093 1.08129
H -0.75664 5.75805 -0.66965
H -2.36984 5.20045 -0.12320
H -3.64497 1.12427 -3.07294
H -2.19488 0.90957 -5.09606
H 0.16302 0.00848 -4.81898
H 0.93151 -0.63372 -2.52090
H -5.85756 1.38317 0.97412
H -4.63457 0.63015 3.02898
H -2.31647 -0.23600 2.86682
H 1.11712 -0.74749 5.27891
H -0.26256 -1.88969 5.30463
H 1.37046 -2.46529 4.83915
H -1.56059 -5.49114 0.40818
H -3.16994 -4.72020 0.25416
H -2.17171 -5.00902 -1.20499
N 1.89264 -1.28430 -0.26660
C 1.98825 -2.58291 -0.66402
C 3.10998 -3.13243 -1.29037
C 4.21367 -2.30348 -1.53544
C 4.14868 -0.97521 -1.10515
C 2.99158 -0.48680 -0.45811
H 1.10475 -3.19864 -0.48271
H 3.10446 -4.18605 -1.58136
H 5.11066 -2.68586 -2.03131
H 5.00177 -0.30381 -1.23334
C 3.01075 0.91022 0.05868
C 3.47510 1.96145 -0.76530
C 3.60677 3.26115 -0.25434
C 3.29236 3.52697 1.08983
C 2.83539 2.48807 1.91759
C 2.69358 1.18844 1.40735
H 3.72467 1.75768 -1.81202
H 3.96012 4.06651 -0.90623
H 3.40710 4.53917 1.49069
H 2.60063 2.68553 2.96858
H 2.36385 0.37469 2.05920

60
TS(2a-3f)2ppy
SCF(BP86)= -1451.32357949
G(298 K) = -1450.924495
SCF(PBE) = -1450.13570725
Low Freq. = -88.8532cm-1,
7.5578cm-1
C -1.96328 0.83946 -0.84436
C -3.07863 -0.04501 -0.97271
C -4.29476 0.37745 -1.55702

```
C  -4.41419  1.68824 -2.03551
C  -3.31922  2.57067 -1.93385
C  -2.11096  2.15116 -1.34669
C  -2.81631 -1.39909 -0.49064
N  -1.53653 -1.57516  0.01477
C  -1.13267 -2.81483  0.39906
C  -1.97786 -3.92558  0.35415
C  -3.29341 -3.75887 -0.11039
C  -3.70270 -2.49306 -0.53801
Ru -0.35741  0.05254  0.09311
N   0.39217 -0.48034 -1.67071
C   0.73969 -0.82510 -2.73827
C   1.19921 -1.24628 -4.05380
N   0.48074  3.08636  0.80443
C   0.56072  4.16760  1.25441
C   0.65795  5.51236  1.81230
N  -1.16914  0.61467  1.81872
C  -1.68802  0.95606  2.81620
C  -2.31473  1.39656  4.05424
H  -5.14466 -0.30931 -1.64350
H   1.06001 -0.43331 -4.78530
H   2.26932 -1.50703 -4.01231
H   0.62833 -2.12667 -4.39165
H  -4.71283 -2.33963 -0.92631
H  -3.98419 -4.60592 -0.14948
H  -1.60058 -4.89742  0.68112
H  -0.10290 -2.89242  0.75044
H  -5.35243  2.02312 -2.48924
H  -3.41159  3.59524 -2.31338
H  -1.27752  2.85864 -1.26296
H   1.57869  6.00237  1.45779
H  -0.20989  6.11449  1.49939
H   0.68091  5.46390  2.91260
H  -1.61817  2.03267  4.62483
H  -3.22594  1.97679  3.83457
H  -2.58889  0.52751  4.67463
N   1.44942 -0.81901  1.29063
C   1.10476 -1.41204  2.46816
C   1.98712 -2.13755  3.27235
C   3.31787 -2.27297  2.85182
C   3.70017 -1.64693  1.66235
C   2.76153 -0.91316  0.90251
H   0.05744 -1.30292  2.75998
H   1.62700 -2.58780  4.20099
H   4.04450 -2.83671  3.44414
H   4.73669 -1.68933  1.31764
C   3.23943 -0.20837 -0.31905
C   4.06030 -0.89255 -1.24482
C   4.57330 -0.22926 -2.37008
C   4.28588  1.13110 -2.57892
C   3.48361  1.82208 -1.65527
C   2.96230  1.16028 -0.53230
H   4.28031 -1.95428 -1.09041
H   5.19977 -0.77555 -3.08267
H   4.69152  1.65047 -3.45307
H   3.26896  2.88566 -1.80249
H   2.35304  1.71567  0.18776

54
3a 2ppy-H
SCF(BP86)= -1318.59276778
G(298 K) = -1318.232782
SCF(PBE) = -1317.51145446
Low Freq. = 16.4785cm-1,
30.7784cm-1
C  -2.06208  0.02498  0.28470
C  -2.20262 -1.14338  1.10073
C  -3.39908 -1.39259  1.81149
C  -4.47640 -0.50389  1.71390
C  -4.36142  0.63616  0.89563
C  -3.17295  0.89499  0.18914
C  -1.04959 -2.04661  1.10916
N   0.01081 -1.61060  0.34020
C   1.09992 -2.40961  0.20473
C   1.21387 -3.64824  0.84163
C   0.15907 -4.08594  1.65898
C  -0.97647 -3.28112  1.78524
Ru -0.26504  0.26194 -0.56452
N  -0.89319 -0.65530 -2.21896
C  -1.25065 -1.20216 -3.19499
C  -1.67413 -1.87909 -4.41298
N  -0.69064  2.05125 -1.35921
C  -0.92127  3.10586 -1.82458
C  -1.21790  4.41044 -2.39953
H  -3.49309 -2.28370  2.44189
H  -1.05414 -1.55341 -5.26443
H  -1.56960 -2.97015 -4.29593
H  -2.72889 -1.64615 -4.63275
H  -1.81841 -3.60925  2.39965
H   0.21683 -5.04524  2.18156
H   2.11545 -4.24789  0.69548
H   1.89377 -2.02824 -0.44029
H  -5.40100 -0.69927  2.26581
H  -5.20435  1.33113  0.80626
H  -3.11196  1.79031 -0.43755
H  -1.69363  4.28964 -3.38658
H  -1.90328  4.97056 -1.74226
H  -0.29006  4.99272 -2.52320
N   0.57719  1.24355  1.09574
C   1.94237  1.23812  1.26870
C   2.53282  1.90456  2.36009
C   1.73624  2.58672  3.28501
C   0.34698  2.59965  3.09159
C  -0.18789  1.92492  1.99213
C   2.76160  0.52700  0.25965
H   3.62109  1.90356  2.45740
H   2.19219  3.10914  4.13057
H  -0.32320  3.12492  3.77652
H  -1.26336  1.90109  1.80339
C   2.33295  0.45065 -1.08945
C   3.08924 -0.24335 -2.04987
C   4.28497 -0.87780 -1.67504
C   4.71926 -0.81009 -0.33889
C   3.96643 -0.11639  0.62054
H   1.48198  1.07111 -1.44440
H   2.74603 -0.27524 -3.08825
H   4.87668 -1.41941 -2.41912
H   5.64489 -1.31029 -0.03821
H   4.29892 -0.10637  1.66301

54
3b 2ppy-H
```

SCF(BP86)= -1318.58865966
G(298 K) = -1318.228935
SCF(PBE) = -1317.50701339
Low Freq. = 2.3536cm-1, 29.5306cm-1

```
C    3.97461 -1.13261  1.26655
C    2.85891 -0.48425  0.69040
C    1.55244 -1.06777  0.75228
C    1.40912 -2.26326  1.48908
C    2.52269 -2.90010  2.06912
C    3.81061 -2.34768  1.94334
C    2.91886  0.84630  0.07930
N    1.68528  1.33044 -0.29866
C    1.58842  2.58295 -0.80774
C    2.69908  3.41144 -0.98965
C    3.96744  2.92148 -0.63463
C    4.07324  1.63549 -0.09584
Ru   0.10325 -0.02782 -0.17813
N   -0.45402  0.68764  1.59440
C   -0.74987  1.09869  2.65346
C   -1.12977  1.61358  3.96124
C   -2.11324  1.31751 -0.99231
C   -2.98269  0.52030 -0.20487
C   -4.03444  1.15083  0.49487
C   -4.21288  2.53953  0.40821
C   -3.35199  3.32107 -0.38296
C   -2.30318  2.70643 -1.08580
C   -2.78663 -0.94599 -0.15426
N   -1.49398 -1.41132 -0.21376
C   -1.29765 -2.75176 -0.34890
C   -2.34173 -3.67891 -0.34562
C   -3.65942 -3.21605 -0.20416
C   -3.87729 -1.83702 -0.12268
N    0.67517 -0.73807 -1.94936
C    1.02503 -1.15601 -2.99012
C    1.45217 -1.66494 -4.28540
H    5.04916  1.23758  0.19355
H   -2.22494  1.57504  4.07944
H   -0.66543  1.00920  4.75748
H   -0.79793  2.65908  4.07047
H    4.97096 -0.68124  1.20287
H    4.67506 -2.84941  2.38916
H    2.38006 -3.83282  2.62678
H    0.42005 -2.71212  1.62578
H    4.86151  3.53644 -0.77284
H    2.56474  4.41335 -1.40417
H    0.57991  2.90991 -1.07213
H    0.57633 -1.98248 -4.87462
H    1.98976 -0.88072 -4.84328
H    2.12294 -2.52965 -4.15293
H   -0.25678 -3.06685 -0.44354
H   -2.11537 -4.74312 -0.44768
H   -4.50176 -3.91317 -0.18757
H   -4.89191 -1.43337 -0.07636
H   -4.69733  0.55628  1.13118
H   -5.02720  3.01455  0.96406
H   -3.50283  4.40226 -0.45703
H   -1.64819  3.30403 -1.72772
H   -1.36213  0.83506 -1.64885
```

54

3c 2ppy-H
SCF(BP86)= -1318.58592835
G(298 K) = -1318.226511
SCF(PBE) = -1317.50400830
Low Freq. = 22.6751cm-1, 27.5669cm-1

```
C    1.32531 -0.60267 -1.32149
C    2.65862 -0.67725 -0.80025
C    3.70153 -1.29758 -1.52718
C    3.44586 -1.85836 -2.78354
C    2.14308 -1.80199 -3.31443
C    1.10559 -1.18443 -2.59330
C    2.84809 -0.08459  0.52570
N    1.69481  0.43623  1.08337
C    1.74535  0.99549  2.32064
C    2.92316  1.06887  3.06636
C    4.10583  0.54892  2.51384
C    4.06136 -0.02722  1.24198
Ru  -0.00397  0.35145 -0.10158
N    0.52009  2.11312 -0.91009
C    0.83330  3.14028 -1.38295
C    1.20455  4.41632 -1.97856
N   -1.20496  1.39373  1.34561
C   -1.90458  1.95915  2.09821
C   -2.76969  2.66223  3.03527
H    4.71516 -1.34711 -1.11447
H    0.32151  4.89267 -2.43514
H    1.61330  5.09072 -1.20859
H    1.96855  4.26329 -2.75811
H   -2.20791  2.92119  3.94717
H   -3.15051  3.58810  2.57475
H   -3.62390  2.02322  3.31125
H    4.96860 -0.43820  0.79268
H    5.04815  0.59243  3.06745
H    2.90403  1.52804  4.05760
H    0.80189  1.38868  2.70220
H    4.25341 -2.33806 -3.34537
H    1.93409 -2.24178 -4.29639
H    0.10462 -1.16118 -3.04281
N   -0.67743 -1.48378  0.61408
C   -1.98683 -1.83141  0.35191
C   -2.50312 -3.06402  0.79642
C   -1.69335 -3.94948  1.51358
C   -0.36087 -3.58804  1.76816
C    0.10613 -2.35845  1.29991
C   -2.77014 -0.84735 -0.41424
H   -3.53618 -3.32907  0.55945
H   -2.09071 -4.90839  1.85745
H    0.31805 -4.24686  2.31499
H    1.13684 -2.04188  1.46932
C   -2.06497  0.17580 -1.11128
C   -2.76615  1.16275 -1.83113
C   -4.16915  1.15979 -1.83963
C   -4.87259  0.16235 -1.13933
C   -4.18053 -0.83042 -0.42969
H   -0.99120 -0.04607 -1.52337
H   -2.20878  1.92298 -2.38649
H   -4.71417  1.93040 -2.39356
H   -5.96677  0.16108 -1.13964
H   -4.74589 -1.57532  0.13855
```

54
3d 2ppy-H
SCF(BP86)= -1318.58312246
G(298 K) = -1318.223763
SCF(PBE) = -1317.50134024
Low Freq. = 27.0758cm-1,
28.2111cm-1

```
C  -3.51837 -1.55875 -1.77415
C  -2.56840 -0.78667 -1.06638
C  -1.24834 -1.28316 -0.81442
C  -0.93587 -2.56709 -1.31455
C  -1.88209 -3.33248 -2.02061
C  -3.17711 -2.83099 -2.24938
C  -2.84691  0.56325 -0.55970
N  -1.78619  1.15276  0.09704
C  -1.92504  2.40797  0.59476
C  -3.10631  3.14297  0.47118
C  -4.19684  2.55662 -0.19296
C  -4.06134  1.26443 -0.70776
Ru -0.05765 -0.01812  0.28052
N   0.98395  1.36270  1.53013
C   1.57363  2.08651  2.24064
C   2.29940  2.99155  3.12140
C   1.18326  0.95910 -1.38346
C   2.51476  0.47258 -1.23546
C   3.58183  1.26666 -1.70387
C   3.33057  2.50026 -2.32356
C   2.01205  2.96611 -2.48266
C   0.93761  2.19128 -2.02104
C   2.71231 -0.80233 -0.52431
N   1.64700 -1.22597  0.24336
C   1.76498 -2.39415  0.93061
C   2.91704 -3.18212  0.89562
C   4.00410 -2.76312  0.11098
C   3.89255 -1.56659 -0.60322
N  -0.81511 -0.81105  1.92832
C  -1.28087 -1.25582  2.90944
C  -1.84759 -1.81364  4.12914
H  -4.89651  0.79078 -1.22943
H  -4.52638 -1.17091 -1.95820
H  -3.91374 -3.42745 -2.79689
H  -1.60766 -4.32556 -2.39489
H   0.06386 -2.98606 -1.15343
H  -5.13922  3.10010 -0.30790
H  -3.16197  4.15049  0.89044
H  -1.04859  2.81348  1.10460
H   3.18769  3.38833  2.60403
H   1.65266  3.83371  3.41645
H   2.62466  2.45632  4.02805
H  -1.04998 -1.99796  4.86722
H  -2.57923 -1.11303  4.56360
H  -2.35560 -2.76668  3.90904
H   0.89314 -2.68453  1.52016
H   2.94941 -4.11099  1.47040
H   4.91720 -3.36161  0.05078
H   4.71236 -1.22323 -1.23879
H   0.31491  0.18487 -1.46955
H  -0.09283  2.53234 -2.15828
H   1.82417  3.92743 -2.97052
H   4.17046  3.10476 -2.67922
H   4.61600  0.93942 -1.55977
```

54
3e 2ppy-H
SCF(BP86)= -1318.57858087
G(298 K) = -1318.219102
SCF(PBE) = -1317.49656969
Low Freq. = 25.8066cm-1,
27.1532cm-1

```
C  -3.59474 -1.40275 -1.57303
C  -2.53089 -0.55553 -1.19790
C  -1.20001 -1.03269 -1.36318
C  -0.94942 -2.30564 -1.90843
C  -2.02176 -3.13196 -2.27581
C  -3.34150 -2.67484 -2.10903
C  -2.76048  0.76098 -0.56494
N  -1.73559  1.24310  0.21154
C  -1.90626  2.42897  0.84858
C  -3.07337  3.19170  0.74340
C  -4.11678  2.71567 -0.06656
C  -3.95505  1.49186 -0.72445
Ru  0.09932  0.01186  0.33083
N   0.84209  0.85108  1.93585
C   1.28584  1.35501  2.89908
C   1.82980  1.98742  4.09263
C   1.77120 -1.14462  0.16052
C   2.84248 -0.55606 -0.58880
C   4.05720 -1.24745 -0.80848
C   4.23693 -2.53291 -0.28492
C   3.19998 -3.12851  0.45978
C   1.99181 -2.44298  0.67751
C   2.57830  0.78764 -1.11186
N   1.31453  1.27024 -0.83218
C   0.95647  2.50303 -1.27418
C   1.81646  3.32094 -2.00931
C   3.10668  2.84695 -2.30179
C   3.48245  1.57941 -1.85073
N  -0.88235 -1.27990  1.50991
C  -1.45516 -2.01127  2.22681
C  -2.15514 -2.93079  3.11329
H   4.86441 -0.78753 -1.38952
H   4.48171  1.19355 -2.06645
H   3.80986  3.45917 -2.87396
H   1.47482  4.30434 -2.34073
H  -0.05646  2.82414 -1.02262
H   5.17665 -3.06825 -0.45388
H   3.33633 -4.13441  0.87427
H   1.20456 -2.93243  1.26199
H  -3.05637 -3.32333  2.61490
H  -1.50069 -3.77633  3.38166
H  -2.45980 -2.41019  4.03578
H   1.03585  2.12257  4.84526
H   2.62860  1.36350  4.52586
H   2.24886  2.97493  3.83910
H  -1.06529  2.76051  1.46477
H  -3.15249  4.13948  1.28177
H  -5.03932  3.29034 -0.18776
H  -4.74385  1.10583 -1.37471
H  -0.33942 -0.28688 -1.45407
H   0.08413 -2.63620 -2.04435
H  -1.83148 -4.12481 -2.69432
H  -4.18218 -3.31691 -2.38914
```

```
H  -4.62950 -1.08415 -1.41566

54
3f 2ppy-H
SCF(BP86)= -1318.57475793
G(298 K) = -1318.215148
SCF(PBE) = -1317.49259669
Low Freq. = 29.5962cm-1,
31.0964cm-1
C  -1.86735 -1.38635  0.76173
C  -2.96042 -0.67903  0.18896
C  -4.04668 -1.41519 -0.32961
C  -4.05921 -2.81610 -0.26337
C  -2.98614 -3.50687  0.32532
C  -1.89438 -2.79058  0.83922
C  -2.92472  0.79656  0.13016
N  -1.68148  1.37120  0.09819
C  -1.59941  2.72378  0.16391
C  -2.71872  3.56113  0.19947
C  -3.99491  2.97735  0.18273
C  -4.09485  1.58240  0.16138
Ru  0.08813 -0.00478  0.17164
N   0.52787  0.90586  1.89618
C   0.80717  1.45662  2.89420
C   1.14828  2.13397  4.13688
C   1.70572 -1.21116  0.51120
C   2.95307 -0.68020  0.04650
C   4.18160 -1.33678  0.28800
C   4.20084 -2.54157  1.00061
C   2.99043 -3.08176  1.47524
C   1.77027 -2.42315  1.23421
C   2.84060  0.56742 -0.70871
N   1.55059  1.05011 -0.80456
C   1.29448  2.13106 -1.58387
C   2.30074  2.83061 -2.25318
C   3.62864  2.39139 -2.11849
C   3.89148  1.25306 -1.35240
N  -0.28688 -0.85667 -1.59307
C  -0.47340 -1.32881 -2.65067
C  -0.71046 -1.92186 -3.95927
H   5.12399 -0.91044 -0.07405
H  -1.79204 -1.96211 -4.16737
H  -0.22124 -1.31878 -4.74141
H  -0.30290 -2.94551 -3.99375
H   4.91240  0.87464 -1.25967
H   4.44495  2.92024 -2.61887
H   2.03864  3.69812 -2.86322
H   0.24646  2.42414 -1.66554
H   5.14929 -3.05401  1.19003
H   2.99710 -4.02093  2.04063
H   0.85455 -2.86825  1.63872
H   0.23396  2.49912  4.63237
H   1.66885  1.44014  4.81704
H   1.80822  2.99274  3.93201
H  -0.58894  3.14062  0.18993
H  -2.58417  4.64479  0.24280
H  -4.89589  3.59666  0.21074
H  -5.07227  1.09542  0.20295
H  -4.87463 -0.89070 -0.81617
H  -4.90774 -3.36929 -0.67736
H  -3.00006 -4.59914  0.38697
H  -1.06749 -3.32612  1.31201
H  -1.11528 -0.86008  1.43057

48
Int(3-4)a 2ppy-H
SCF(BP86)= -1185.80669069
G(298 K) = -1185.488981
SCF(PBE) = -1184.83350482
Low Freq. = 28.1634cm-1,
32.2443cm-1
C  -1.12429  0.04108  1.35486
C  -2.45067 -0.32445  0.94626
C  -3.44941 -0.60342  1.90690
C  -3.15351 -0.52919  3.27261
C  -1.85335 -0.17723  3.68464
C  -0.85144  0.10452  2.73851
C  -2.66208 -0.39389 -0.49955
N  -1.52428 -0.10781 -1.22414
C  -1.54713 -0.14571 -2.57966
C  -2.70427 -0.47013 -3.29094
C  -3.87789 -0.76174 -2.57235
C  -3.85382 -0.72372 -1.17472
Ru  0.13590  0.41813 -0.14454
N  -0.44591  2.32965 -0.24838
C  -0.79295  3.44790 -0.34004
C  -1.20311  4.83985 -0.45008
H  -4.45931 -0.88164  1.58601
H  -0.32033  5.49905 -0.41655
H  -1.73601  5.00525 -1.40064
H  -1.87484  5.10287  0.38340
H  -4.75569 -0.94777 -0.59967
H  -4.80189 -1.01637 -3.09917
H  -2.67945 -0.49019 -4.38284
H  -0.60502  0.09555 -3.08139
H  -3.92826 -0.74594  4.01420
H  -1.61556 -0.12054  4.75269
H   0.15004  0.37827  3.09016
N   0.92762 -1.49324 -0.09639
C   2.30439 -1.57754 -0.06330
C   2.94835 -2.82715 -0.07344
C   2.19040 -4.00314 -0.11399
C   0.78980 -3.90640 -0.13317
C   0.19850 -2.64105 -0.12093
C   2.99928 -0.28045 -0.00365
H   4.03938 -2.87051 -0.03282
H   2.68414 -4.97869 -0.11941
H   0.15488 -4.79536 -0.15311
H  -0.88631 -2.51694 -0.13178
C   2.23673  0.86160  0.40079
C   2.85003  2.13314  0.46294
C   4.19040  2.28569  0.08052
C   4.93458  1.16787 -0.34106
C   4.34376 -0.10411 -0.38626
H   1.34116  0.71397  1.15816
H   2.27229  2.99197  0.81659
H   4.65851  3.27407  0.11757
H   5.97986  1.28768 -0.64102
H   4.92673 -0.95678 -0.74769

48
Int(3-4)b 2ppy-H
```

SCF(BP86)= -1185.80134000
G(298 K) = -1185.483868
SCF(PBE) = -1184.82848086
Low Freq. = 15.4863cm-1, 33.2642cm-1

```
C    3.14457 -1.18642  2.19507
C    2.32384 -0.57274  1.22163
C    0.95713 -0.97474  1.06623
C    0.45587 -1.98130  1.91864
C    1.28290 -2.58245  2.88499
C    2.62796 -2.19057  3.02274
C    2.77162  0.50663  0.33698
N    1.77976  0.97679 -0.49572
C    2.05082  1.97983 -1.36700
C    3.31273  2.57265 -1.45728
C    4.33588  2.10725 -0.61262
C    4.06152  1.07153  0.28604
Ru  -0.04458 -0.01657 -0.38147
C   -1.21569  1.61289  0.57859
C   -2.59369  1.31540  0.33022
C   -3.50928  2.38105  0.22398
C   -3.08054  3.70670  0.39340
C   -1.72995  3.99321  0.66838
C   -0.80099  2.94898  0.77677
C   -2.96532 -0.09458  0.11650
N   -1.91234 -0.92912 -0.19787
C   -2.16595 -2.24810 -0.41423
C   -3.44900 -2.79308 -0.33064
C   -4.52595 -1.95174 -0.00534
C   -4.27622 -0.59393  0.22162
N    0.61677 -1.29174 -1.73331
C    0.99945 -2.01903 -2.57286
C    1.45951 -2.92451 -3.61421
H    4.84480  0.69389  0.94796
H    4.18888 -0.87634  2.31074
H    3.26698 -2.66298  3.77504
H    0.87191 -3.36319  3.53489
H   -0.58688 -2.30330  1.83551
H    5.33668  2.54649 -0.65681
H    3.48265  3.37821 -2.17551
H    1.21632  2.29776 -1.99924
H    0.60865 -3.25324 -4.23306
H    2.19798 -2.42034 -4.25865
H    1.93107 -3.81093 -3.15905
H   -1.29652 -2.86152 -0.66098
H   -3.59226 -3.86083 -0.51328
H   -5.54149 -2.34842  0.07702
H   -5.09044  0.08224  0.49354
H   -0.57107  0.81212  1.13896
H    0.24529  3.15835  1.01720
H   -1.40548  5.02916  0.80543
H   -3.80519  4.52180  0.30663
H   -4.55726  2.18122 -0.01900
```

48
Int(3-4)c 2ppy-H
SCF(BP86)= -1185.78711837
G(298 K) = -1185.469168
SCF(PBE) = -1184.81795715
Low Freq. = 27.9404cm-1, 29.0685cm-1

```
C    2.14763  0.08274 -0.30662
C    2.38341  1.46095  0.00816
C    3.67944  1.92193  0.32912
C    4.76229  1.03338  0.34149
C    4.55001 -0.32196  0.02733
C    3.26213 -0.79019 -0.28961
C    1.20668  2.33285 -0.05950
N    0.04119  1.67768 -0.41176
C   -1.09691  2.40412 -0.56961
C   -1.14973  3.78594 -0.36809
C    0.02096  4.45633  0.01907
C    1.20179  3.72209  0.16765
Ru   0.25301 -0.37867 -0.75651
N    0.65389 -2.28006 -1.20448
C    0.87607 -3.39804 -1.49851
C    1.16345 -4.77377 -1.87650
H    3.84910  2.97731  0.56895
H    2.12938  4.22456  0.45189
H    0.01626  5.53590  0.19508
H   -2.09520  4.31439 -0.51111
H   -1.98369  1.84459 -0.87114
H    5.76447  1.39396  0.59276
H    5.39315 -1.02216  0.03107
H    3.12732 -1.85157 -0.52161
H    1.41719 -4.82482 -2.94836
H    2.01421 -5.16208 -1.29261
H    0.28402 -5.41206 -1.69108
N   -0.30244 -0.89275  1.10230
C   -1.63741 -0.84193  1.45545
C   -2.04313 -1.20422  2.75393
C   -1.10799 -1.62712  3.70241
C    0.24080 -1.69604  3.32242
C    0.60410 -1.32956  2.02688
C   -2.61249 -0.42720  0.42408
H   -3.10826 -1.17614  2.99565
H   -1.42663 -1.91210  4.70854
H    1.01705 -2.03148  4.01450
H    1.64067 -1.35874  1.69072
C   -2.36457 -0.69878 -0.94440
C   -3.26800 -0.28580 -1.93886
C   -4.43525  0.40708 -1.57892
C   -4.69229  0.68109 -0.22294
C   -3.79080  0.27187  0.77069
H   -1.53839 -1.38008 -1.24164
H   -3.06044 -0.51753 -2.98786
H   -5.14212  0.73040 -2.34882
H   -5.59509  1.22939  0.06266
H   -3.98495  0.53135  1.81573
```

48
Int(3-4)d 2ppy-H
SCF(BP86)= -1185.78199587
G(298 K) = -1185.464815
SCF(PBE) = -1184.81201153
Low Freq. = 12.7074cm-1, 26.9796cm-1

```
C   -4.11904  1.48659  0.25466
C   -2.97359  0.70063  0.00285
C   -1.65759  1.22885  0.22031
C   -1.55304  2.50590  0.81622
C   -2.70060  3.27733  1.07891
```

C -3.98224 2.77959 0.77822
C -3.01836 -0.71163 -0.39245
N -1.76905 -1.29622 -0.45637
C -1.66568 -2.62592 -0.70666
C -2.78041 -3.43498 -0.94265
C -4.05555 -2.84473 -0.92321
C -4.17171 -1.47857 -0.64270
Ru -0.17712 0.03645 -0.37908
N 0.27718 -0.37657 1.41116
C 0.52965 -0.61367 2.53622
C 0.86199 -0.92620 3.91946
C 2.09656 -1.42381 -0.96497
C 2.91272 -0.48214 -0.28832
C 3.92537 -0.95493 0.57396
C 4.11174 -2.33301 0.75990
C 3.29822 -3.26029 0.08454
C 2.29282 -2.80309 -0.78266
C 2.70359 0.96643 -0.51281
N 1.40392 1.40222 -0.63636
C 1.18992 2.69860 -1.00020
C 2.22544 3.61562 -1.19051
C 3.54990 3.19443 -0.99446
C 3.78420 1.85526 -0.66309
H -5.15539 -1.00338 -0.61172
H 1.95116 -0.85340 4.07483
H 0.36119 -0.22031 4.60256
H 0.53762 -1.94986 4.17072
H -5.12028 1.08548 0.06256
H -4.87100 3.38667 0.97597
H -2.59187 4.27286 1.52372
H -0.56937 2.91385 1.07223
H -4.94957 -3.44429 -1.11735
H -2.64347 -4.50066 -1.14035
H -0.64920 -3.02736 -0.71745
H 0.14379 2.98916 -1.11631
H 1.98672 4.64264 -1.47738
H 4.38500 3.88873 -1.12214
H 4.80301 1.47394 -0.55744
H 4.54908 -0.24097 1.12111
H 4.89477 -2.68470 1.43878
H 3.45463 -4.33396 0.22564
H 1.67887 -3.51847 -1.33882
H 1.38748 -1.08331 -1.74197

48
Int(3-4)e 2ppy-H
SCF(BP86)= -1185.78271135
G(298 K) = -1185.465404
SCF(PBE) = -1184.80927299
Low Freq. = 15.3970cm-1,
32.5837cm-1
C 1.36530 -1.31780 -0.71753
C 2.73091 -0.88982 -0.59505
C 3.80359 -1.78522 -0.80457
C 3.54893 -3.12640 -1.11729
C 2.21901 -3.57710 -1.21453
C 1.14923 -2.68647 -1.01470
C 2.91837 0.51452 -0.21835
N 1.73539 1.21192 -0.04557
C 1.77694 2.50811 0.36269
C 2.97586 3.18243 0.60139
C 4.18640 2.49648 0.40739
C 4.15122 1.15977 -0.00083
Ru 0.01026 0.18332 -0.55351
N -1.15085 1.97131 -0.60923
C -1.80153 2.94109 -0.72838
C -2.60550 4.14518 -0.87522
H 4.84047 -1.44140 -0.72298
H -1.95385 5.01423 -1.06066
H -3.30257 4.03219 -1.72122
H -3.18640 4.32376 0.04419
H 5.08104 0.60548 -0.14915
H 5.14462 2.99548 0.57774
H 2.95088 4.22395 0.93025
H 0.80974 2.99673 0.49177
H 4.38119 -3.81876 -1.27761
H 2.01424 -4.62824 -1.44702
H 0.12630 -3.07536 -1.09606
N -0.51510 -0.37229 1.28478
C -1.82002 -0.78807 1.50010
C -2.23515 -1.17634 2.78758
C -1.34107 -1.15517 3.86085
C -0.01763 -0.74938 3.62210
C 0.35968 -0.37364 2.33378
C -2.68702 -0.80018 0.31567
H -3.26481 -1.51280 2.93010
H -1.66410 -1.46164 4.85912
H 0.72942 -0.72824 4.41927
H 1.37711 -0.05750 2.10516
C -2.05662 -0.72466 -0.95870
C -2.82693 -0.69317 -2.13740
C -4.22757 -0.70897 -2.05487
C -4.85748 -0.76834 -0.79711
C -4.09611 -0.80874 0.38018
H -0.98739 -1.18266 -1.09873
H -2.32678 -0.66603 -3.11061
H -4.82898 -0.67950 -2.96863
H -5.94970 -0.77524 -0.73269
H -4.60466 -0.81567 1.34907

48
Int(3-4)f 2ppy-H
SCF(BP86)= -1185.77817197
G(298 K) = -1185.461249
SCF(PBE) = -1184.80394902
Low Freq. = 20.3594cm-1,
27.5720cm-1
C -3.47944 2.41817 0.20255
C -2.58849 1.33502 0.34608
C -1.21060 1.61779 0.60255
C -0.77052 2.94827 0.76955
C -1.67602 4.00848 0.62394
C -3.02783 3.73967 0.34152
C -3.01242 -0.06877 0.15879
N -2.01224 -0.94414 -0.18750
C -2.33335 -2.24394 -0.40806
C -3.63576 -2.73769 -0.28517
C -4.65892 -1.85156 0.08860
C -4.34182 -0.50690 0.31240
Ru -0.00491 -0.03243 -0.40142
N 0.65043 -1.31368 -1.73208
C 1.02990 -2.07449 -2.54310

```
C   1.48141 -3.01943 -3.55305
C   1.73649  0.98910 -0.53548
C   2.77703  0.53660  0.33469
C   4.05714  1.13719  0.33360
C   4.32194  2.19705 -0.54212
C   3.31097  2.65610 -1.41205
C   2.03640  2.06088 -1.40626
C   2.37931 -0.56946  1.20041
N   1.06684 -0.98008  1.02056
C   0.56248 -1.99334  1.77448
C   1.32336 -2.65071  2.74147
C   2.65727 -2.25323  2.94063
C   3.17728 -1.21258  2.16810
H   4.84544  0.78141  1.00673
H   4.20977 -0.88199  2.30641
H   3.28057 -2.74899  3.69011
H   0.86937 -3.45790  3.32079
H  -0.47809 -2.25926  1.58128
H   5.31095  2.66585 -0.55144
H   3.52112  3.48488 -2.09814
H   1.26597  2.43840 -2.09126
H   0.68702 -3.18122 -4.30004
H   2.37967 -2.63538 -4.06328
H   1.72533 -3.98554 -3.08139
H  -1.50332 -2.89534 -0.69733
H  -3.83537 -3.79541 -0.47376
H  -5.68755 -2.20302  0.20839
H  -5.11594  0.20070  0.61909
H  -0.57136  0.81464  1.13476
H   0.27938  3.13843  1.00778
H  -1.33208  5.04105  0.73595
H  -3.73657  4.56493  0.22381
H  -4.52830  2.23278 -0.04744

48
Int(3-4)g 2ppy-H
SCF(BP86)= -1185.77017533
G(298 K) = -1185.454021
SCF(PBE) = -1184.79738712
Low Freq. = 26.4000cm-1,
32.1185cm-1
C   3.22495 -0.97847  2.06767
C   2.30852 -0.33002  1.21493
C   0.94797 -0.78767  1.19264
C   0.55427 -1.87144  2.01353
C   1.48801 -2.49873  2.84958
C   2.82066 -2.04869  2.87911
C   2.69870  0.73703  0.27822
N   1.75113  1.04252 -0.66837
C   2.03729  1.97230 -1.61216
C   3.25731  2.65430 -1.65915
C   4.22227  2.37005 -0.67846
C   3.93943  1.40592  0.29499
Ru -0.13331 -0.05089 -0.51183
C  -1.94687 -0.95476 -0.28288
C  -3.02518 -0.05681  0.02085
C  -4.35926 -0.51172  0.12599
C  -4.65255 -1.86940 -0.05501
C  -3.61060 -2.77304 -0.33891
C  -2.28362 -2.32043 -0.44692
C  -2.63600  1.34182  0.23727
N  -1.27916  1.58285  0.12618
C  -0.79724  2.83378  0.34687
C  -1.62328  3.91155  0.66998
C  -3.00717  3.69005  0.76821
C  -3.50827  2.40363  0.55139
N   0.69964 -1.63891 -1.39774
C   1.18924 -2.54445 -1.96205
C   1.77942 -3.66890 -2.67181
H  -5.17312  0.18575  0.35319
H  -4.58122  2.21253  0.62652
H  -3.68688  4.51061  1.01555
H  -1.18349  4.89694  0.83975
H   0.28448  2.95053  0.25819
H  -5.68501 -2.22281  0.02956
H  -3.83542 -3.83730 -0.47546
H  -1.49395 -3.04796 -0.66715
H   2.60694 -4.09096 -2.07863
H   1.02487 -4.45416 -2.84147
H   2.17645 -3.33552 -3.64458
H   1.24549  2.16442 -2.34332
H   3.43685  3.39445 -2.44272
H   5.18276  2.89296 -0.67250
H   4.67547  1.17599  1.06904
H   0.05007  0.04497  1.16488
H  -0.48862 -2.19905  1.99607
H   1.17528 -3.33669  3.48022
H   3.55116 -2.53798  3.53010
H   4.27412 -0.66833  2.07944

48
Int(3-4)h 2ppy-H
SCF(BP86)= -1185.77261066
G(298 K) = -1185.455576
SCF(PBE) = -1184.79810928
Low Freq. = 21.8458cm-1,
32.0514cm-1
C   1.55018  1.76177  0.20806
C   2.70661  1.19632 -0.41658
C   3.48144  2.00306 -1.27400
C   3.14052  3.34611 -1.49266
C   2.01753  3.90612 -0.85817
C   1.23228  3.12198 -0.00105
C   3.02057 -0.22668 -0.18069
N   1.93793 -1.01660  0.10174
C   2.15390 -2.31487  0.42943
C   3.42637 -2.89530  0.44128
C   4.53374 -2.09911  0.10763
C   4.32802 -0.74831 -0.19663
Ru -0.01575  0.05761  0.13396
N  -0.38169 -0.72703  1.82875
C  -0.62104 -1.21513  2.87120
C  -0.89941 -1.79917  4.17558
C  -1.76696  1.09599  0.27311
C  -2.87757  0.48360 -0.39403
C  -4.16193  1.07268 -0.39692
C  -4.38049  2.27063  0.29511
C  -3.31953  2.86443  1.00497
C  -2.03961  2.27990  0.99655
C  -2.57515 -0.79975 -1.03035
N  -1.27581 -1.23178 -0.83498
C  -0.86316 -2.40169 -1.38852
```

C -1.70966 -3.21075 -2.14846
C -3.03604 -2.79391 -2.35026
C -3.46204 -1.58505 -1.79268
H -4.99554 0.59654 -0.92509
H -4.48530 -1.23577 -1.95012
H -3.72751 -3.40198 -2.94047
H -1.32754 -4.14381 -2.56863
H 0.17848 -2.67435 -1.21255
H -5.37383 2.73005 0.29692
H -3.49127 3.78735 1.57065
H -1.24431 2.75982 1.57985
H 0.01427 -1.79986 4.79260
H -1.67803 -1.21720 4.69580
H -1.25002 -2.83819 4.06231
H 1.26551 -2.89808 0.68975
H 3.53902 -3.94900 0.70808
H 5.54361 -2.51873 0.10478
H 5.17247 -0.09015 -0.41605
H 4.33965 1.56888 -1.79608
H 3.75019 3.95532 -2.16666
H 1.75588 4.95488 -1.02703
H 0.37062 3.56128 0.50584
H 1.10214 1.26860 1.14157

48
Int(3-4)a 2ppy-H (pi)
SCF(BP86)= -1185.79827384
G(298 K) = -1185.480636
SCF(PBE) = -1184.82817603
Low Freq. = 30.3360cm-1,
34.6600cm-1
C -1.95871 0.06377 -2.99181
C -1.57166 -0.05403 -1.63674
C -0.20385 -0.29649 -1.29006
C 0.73121 -0.45957 -2.33555
C 0.33244 -0.34540 -3.67905
C -1.00993 -0.07630 -4.01083
C -2.50147 0.02553 -0.50873
N -1.88731 -0.13196 0.71375
C -2.63496 -0.13369 1.84519
C -4.02129 0.03675 1.82839
C -4.66133 0.22125 0.58970
C -3.89671 0.21349 -0.58052
Ru 0.17746 -0.38665 0.66479
C 0.56808 1.66234 1.65267
C 1.37436 1.75238 0.45787
C 1.05199 2.73532 -0.52385
C -0.03737 3.58072 -0.34252
C -0.83153 3.49702 0.83213
C -0.51863 2.57331 1.82252
C 2.58427 0.89429 0.33985
N 2.24663 -0.41742 0.53565
C 3.20851 -1.37236 0.50868
C 4.54425 -1.04458 0.24713
C 4.89118 0.29446 -0.00063
C 3.89444 1.28464 0.04549
N -0.07904 -2.32535 0.64264
C -0.23877 -3.49005 0.64980
C -0.42035 -4.93352 0.67076
H -4.37549 0.34510 -1.55420
H -3.00518 0.25921 -3.25060
H -1.31272 0.01428 -5.05853
H 1.07689 -0.46937 -4.47373
H 1.78060 -0.67173 -2.10782
H -5.74461 0.36421 0.53869
H -4.57967 0.02763 2.76746
H -2.08302 -0.27336 2.77945
H 0.39630 -5.41296 1.23516
H -1.38153 -5.18926 1.14599
H -0.41913 -5.32744 -0.35890
H 2.87875 -2.40062 0.67739
H 5.29754 -1.83616 0.22964
H 5.92793 0.56639 -0.21745
H 4.12567 2.33932 -0.12551
H 1.67110 2.81156 -1.42262
H -0.27693 4.32683 -1.10643
H -1.67246 4.18372 0.96847
H -1.08949 2.54731 2.75552
H 0.97010 1.15542 2.54362

48
Int(3-4)b 2ppy-H (pi)
SCF(BP86)= -1185.79943598
G(298 K) = -1185.481655
SCF(PBE) = -1184.82712878
Low Freq. = 30.5593cm-1,
34.1878cm-1
C -2.35740 -0.36674 2.81721
C -1.83704 -0.17807 1.51710
C -0.45118 0.12820 1.32013
C 0.36645 0.27310 2.45890
C -0.16326 0.08959 3.75006
C -1.52067 -0.23825 3.93252
C -2.63190 -0.23628 0.29226
N -1.88749 0.01328 -0.84329
C -2.48689 0.03674 -2.06145
C -3.85340 -0.20342 -2.21896
C -4.62711 -0.47909 -1.07699
C -4.01185 -0.49307 0.17878
Ru 0.10616 0.31756 -0.58774
C 2.26840 0.54312 -1.33581
C 2.53337 -0.40334 -0.28021
C 3.43610 -0.04920 0.76030
C 4.02520 1.21327 0.78247
C 3.74267 2.16001 -0.23544
C 2.90123 1.82207 -1.29088
C 1.93369 -1.75718 -0.38374
N 0.60431 -1.68053 -0.70658
C -0.11419 -2.81735 -0.87555
C 0.47266 -4.07777 -0.71498
C 1.82747 -4.16531 -0.35376
C 2.57240 -2.98695 -0.18371
N -0.19737 2.28672 -0.48603
C -0.40607 3.44136 -0.42568
C -0.64768 4.87544 -0.35733
H -4.59546 -0.69921 1.07958
H -3.41680 -0.60811 2.95851
H -1.92293 -0.38472 4.93960
H 0.49222 0.20267 4.62104
H 1.42405 0.52927 2.34198
H -5.69875 -0.67807 -1.16683
H -4.29336 -0.17679 -3.21847

```
H  -1.82984  0.24855 -2.91048
H  -1.25499  5.20187 -1.21756
H   0.30888  5.42312 -0.36908
H  -1.18741  5.12447  0.57099
H  -1.17025 -2.69311 -1.12668
H  -0.13350 -4.97475 -0.86302
H   2.30106 -5.14090 -0.21326
H   3.63203 -3.01403  0.08303
H   3.65628 -0.77754  1.54699
H   4.71370  1.47521  1.59163
H   4.21734  3.14547 -0.20706
H   2.73471  2.52353 -2.11395
H   1.88959  0.17886 -2.30630

55
4a
SCF(BP86)= -1414.46686576
G(298 K) = -1414.105055
SCF(PBE) = -1413.34189112
Low Freq. = 6.4156cm-1, 26.9355cm-
1
C   4.41825 -1.02582 -0.55138
C   3.15016 -0.41702 -0.45804
N   2.12340 -1.05838  0.20817
C   2.34474 -2.28023  0.75871
C   3.58242 -2.92585  0.68738
C   4.64071 -2.28266  0.02122
C   2.75751  0.87649 -1.02215
C   3.63551  1.70059 -1.76306
C   3.17739  2.90128 -2.31895
C   1.82763  3.26881 -2.14445
C   0.95181  2.45627 -1.40431
C   1.38493  1.25063 -0.79220
Ru  0.34547 -0.02454  0.34375
N  -0.27903 -0.85752 -1.30442
C  -0.56801 -1.37826 -2.32253
C  -0.98726 -2.04037 -3.55214
H  -0.55137 -1.54072 -4.43350
H  -0.66566 -3.09583 -3.55656
H  -2.08668 -2.01020 -3.63852
H   4.67958  1.40189 -1.91482
H   3.85770  3.53860 -2.89302
H   1.45410  4.19603 -2.59619
H  -0.09223  2.77206 -1.30016
H   5.22665 -0.50950 -1.07605
H   5.62472 -2.75553 -0.05137
H   3.70721 -3.90939  1.14762
H   1.48791 -2.71777  1.27818
N  -1.34794  1.23426  0.59207
C  -1.04833  2.45134  1.14524
H  -0.00594  2.57259  1.44368
C  -1.96786  3.48720  1.30725
H  -1.63842  4.43006  1.75204
C  -3.28981  3.29268  0.87940
C  -3.62518  2.04358  0.34986
H  -4.04200  4.08095  0.97561
H  -4.65344  1.82353  0.05064
C  -2.66118  1.01826  0.23161
C  -3.12297 -0.31656 -0.24256
C  -3.95797 -0.40669 -1.37948
C  -4.48439 -1.64397 -1.78427
H  -5.12375 -1.69716 -2.67168
C  -4.19450 -2.80685 -1.05014
H  -4.60990 -3.77123 -1.36055
C  -3.37308 -2.72312  0.08804
H  -3.15265 -3.62381  0.67115
C  -2.83808 -1.49004  0.49137
H  -2.19740 -1.42778  1.37631
O  -0.24588 -1.23528  2.34176
C   0.41636 -0.33650  2.95980
H  -4.18289  0.49622 -1.95749
O   0.96911  0.62572  2.28929
C   0.59411 -0.38607  4.46362
H   1.31825 -1.17845  4.72079
H   0.96807  0.57184  4.85283
H  -0.36053 -0.64153  4.94969

55
TS(4a-6f)1
SCF(BP86)= -1414.46284883
G(298 K) = -1414.101165
SCF(PBE) = -1413.33927592
Low Freq. = -26.2754cm-1,
8.2961cm-1
C   4.08346 -0.86283 -0.86897
C   3.01918 -0.43725 -0.04361
C   2.22750 -1.41066  0.61615
C   2.50568 -2.77757  0.44716
C   3.56147 -3.19222 -0.38249
C   4.34956 -2.23039 -1.03910
C   2.73688  1.00242  0.16335
C   3.78261  1.93943  0.28694
C   3.50377  3.27447  0.59733
C   2.16526  3.64341  0.80868
C   1.16707  2.67995  0.65526
N   1.42109  1.38763  0.29885
Ru -0.16736  0.03285  0.08697
N   0.41429 -0.58171 -1.68431
C   0.72604 -0.93393 -2.76550
C   1.13662 -1.38771 -4.08909
H   1.01048 -0.58334 -4.83317
H   2.19741 -1.68921 -4.07698
H   0.53174 -2.25382 -4.40658
H   4.81468  1.59592  0.17751
H   4.31303  4.00303  0.70020
H   1.88870  4.66453  1.08423
H   0.11223  2.91880  0.80046
H   4.68703 -0.12177 -1.40356
H   5.17162 -2.54499 -1.69011
H   3.77436 -4.25831 -0.51177
H   1.90284 -3.52118  0.97944
C  -1.53439  1.17518 -0.79039
C  -1.33554  2.41726 -1.44137
H  -0.32542  2.83253 -1.52737
C  -2.41297  3.14176 -1.98253
H  -2.22487  4.10630 -2.46917
C  -3.72690  2.63869 -1.91048
C  -3.95123  1.38807 -1.32027
H  -4.56298  3.20819 -2.32871
H  -4.96802  0.97896 -1.29420
C  -2.87281  0.65198 -0.78032
C  -2.99839 -0.70564 -0.24535
```

```
N  -1.78584 -1.25685  0.11840
C  -1.74681 -2.53693  0.56666
H  -0.75435 -2.91219  0.82990
C  -2.89236 -3.32685  0.69886
H  -2.80358 -4.35226  1.06640
C  -4.13742 -2.76992  0.35647
H  -5.05686 -3.35444  0.45666
C  -4.18503 -1.45537 -0.11996
H  -5.14128 -1.00378 -0.39775
O  -0.83761  0.75163  1.94842
C  -0.48884  0.10175  3.02765
O   0.26727 -0.90041  3.05691
H   1.46316 -1.11290  1.36538
H  -0.52511  0.32734  5.19109
H  -2.13497  0.23230  4.42514
H  -1.19784  1.73798  4.29304
C  -1.11447  0.64022  4.31659
```

```
55
Int(4a-6f)
SCF(BP86)= -1414.46285206
G(298 K) = -1414.101897
SCF(PBE) = -1413.33938449
Low Freq. = 11.2441cm-1,
19.9547cm-1
C  -4.07563 -0.90922  0.83131
C  -2.99708 -0.45799  0.03908
C  -2.14952 -1.41247 -0.57982
C  -2.39420 -2.78588 -0.40859
C  -3.46591 -3.22546  0.38681
C  -4.30549 -2.28260  1.00609
C  -2.74707  0.98640 -0.16936
C  -3.80724  1.90437 -0.30900
C  -3.54610  3.24314 -0.61908
C  -2.21132  3.63466 -0.81355
C  -1.19862  2.68871 -0.64560
N  -1.43672  1.39390 -0.28964
Ru  0.14753  0.04036 -0.06754
N  -0.41164 -0.53197  1.72576
C  -0.71113 -0.85935  2.81803
C  -1.09817 -1.28097  4.15929
H  -1.34845 -0.40758  4.78487
H  -1.97925 -1.94276  4.11492
H  -0.27322 -1.83029  4.64327
H  -4.83493  1.54439 -0.21220
H  -4.36593  3.95785 -0.73450
H  -1.94907  4.65992 -1.08771
H  -0.14615  2.94512 -0.77777
H  -4.71946 -0.18279  1.33821
H  -5.13971 -2.61658  1.63140
H  -3.65126 -4.29644  0.51781
H  -1.75370 -3.51629 -0.91455
C   1.53869  1.19079  0.76765
C   1.35869  2.44256  1.40485
H   0.35239  2.86412  1.50523
C   2.44988  3.16982  1.91423
H   2.27602  4.14218  2.39047
C   3.75958  2.65923  1.82318
C   3.96620  1.39865  1.24774
H   4.60661  3.23067  2.21602
H   4.98032  0.98409  1.20820
C   2.87345  0.66036  0.74056
C   2.98122 -0.70749  0.22752
N   1.75896 -1.26063 -0.09953
C   1.70684 -2.54918 -0.52109
H   0.70809 -2.92645 -0.75491
C   2.84631 -3.34622 -0.66217
H   2.74578 -4.37832 -1.00724
C   4.10030 -2.78758 -0.35779
H   5.01543 -3.37738 -0.46612
C   4.16230 -1.46416  0.09155
H   5.12577 -1.01085  0.33997
O   0.80451  0.72953 -1.94539
C   0.47482  0.07502 -3.02838
O  -0.26992 -0.93441 -3.07080
H  -1.38188 -1.10497 -1.32739
H   0.55334  0.28435 -5.19308
H   2.14814  0.25246 -4.38942
H   1.15580  1.72435 -4.29206
C   1.11142  0.62408 -4.30820
```

```
55
6f
SCF(BP86)= -1414.41022778
G(298 K) = -1414.049107
SCF(PBE) = -1413.28532610
Low Freq. = 10.2966cm-1,
20.4860cm-1
C   4.05821 -1.50799  0.06843
C   2.91158 -0.68596  0.13008
N   1.64747 -1.24902  0.11671
C   1.54747 -2.59871 -0.00134
C   2.64916 -3.45513 -0.07140
C   3.93702 -2.89688 -0.02455
C   2.93388  0.78302  0.19031
C   1.65129  1.42622  0.09665
C   1.68977  2.83833  0.24819
C   2.88200  3.56768  0.41405
C   4.12074  2.90085  0.45744
C   4.14117  1.50410  0.35768
Ru  0.00232  0.06743  0.07185
O   0.05311 -0.02429 -2.09114
C   0.00270 -0.90836 -2.96162
C   0.07621 -0.58563 -4.43354
C  -1.69009 -1.21049  0.44318
C  -2.94278 -0.49764  0.41811
C  -4.16429 -1.06864  0.85167
C  -4.19846 -2.38323  1.33145
C  -2.99661 -3.11321  1.39110
C  -1.78889 -2.52795  0.96806
C  -2.88089  0.87451 -0.10434
C  -4.00069  1.66771 -0.43785
C  -3.83534  2.91581 -1.04484
C  -2.53236  3.35268 -1.33735
C  -1.45711  2.54257 -0.96388
N  -1.60160  1.34945 -0.32745
N  -0.02282  0.31382  1.98467
C  -0.03183  0.46449  3.15502
C  -0.04256  0.65647  4.60160
O  -0.12246 -2.20038 -2.60399
H  -0.61624  1.56002  4.87053
H  -0.50065 -0.20880  5.11040
```

```
H   0.98607  0.77692  4.98174
H  -5.00581  1.28289 -0.24641
H  -4.70577  3.52564 -1.30578
H  -2.34250  4.30496 -1.83970
H  -0.42721  2.83682 -1.16930
H  -5.09307 -0.48516  0.83513
H  -5.14172 -2.82670  1.66813
H  -3.00005 -4.13959  1.78027
H  -0.88388 -3.13959  1.08107
H   0.75194  3.40994  0.25734
H   2.84613  4.65968  0.51889
H   5.05358  3.45999  0.58730
H   5.10330  0.98233  0.42944
H   0.52994 -2.98262 -0.05801
H   2.49039 -4.53311 -0.16076
H   4.82841 -3.52998 -0.07232
H   5.04903 -1.04667  0.07896
H  -0.15116 -2.76673 -3.40323
H  -0.82767 -0.94964 -4.95105
H   0.94712 -1.08660 -4.89009
H   0.16555  0.49870 -4.57445

55
TS(4a-4a')
SCF(BP86)= -1414.44413141
G(298 K) = -1414.083155
SCF(PBE) = -1413.32079629
Low Freq. = -11.2523cm-1,
10.3980cm-1
C  -4.17083  1.67690  0.67294
C  -2.85953  1.29482  0.32566
N  -2.48907 -0.03368  0.36766
C  -3.40063 -0.97099  0.73753
C  -4.71163 -0.64174  1.09051
C  -5.10374  0.70930  1.05909
C  -1.77089  2.16189 -0.11293
C  -1.91274  3.55217 -0.32329
C  -0.83460  4.30738 -0.79886
C   0.38662  3.65965 -1.07043
C   0.53639  2.27784 -0.85466
C  -0.52555  1.48309 -0.36416
Ru -0.53722 -0.48337 -0.03306
N  -0.11758 -0.20875  1.86009
C   0.10514 -0.07096  3.01092
C   0.37404  0.07866  4.43617
H  -0.05572 -0.76381  5.00460
H   1.46031  0.10960  4.62468
H  -0.07010  1.01524  4.81282
H  -2.87230  4.04391 -0.12417
H  -0.94357  5.38419 -0.96357
H   1.23727  4.23695 -1.45229
H   1.49994  1.81286 -1.07170
H  -4.45056  2.73344  0.64055
H  -6.12243  1.00154  1.33103
H  -5.40670 -1.43369  1.38078
H  -3.03890 -2.00178  0.71482
N   1.41170 -1.51879 -0.14621
C   1.05862 -2.83932 -0.04628
H   0.04084 -3.07552 -0.38877
C   1.89078 -3.83197  0.47587
H   1.53039 -4.86284  0.52713
C   3.16775 -3.47235  0.93216
C   3.56716 -2.14067  0.77913
H   3.84509 -4.21286  1.36766
H   4.57359 -1.82004  1.06228
C   2.69471 -1.18929  0.20960
C   3.24985  0.15916 -0.10423
C   3.46081  0.51799 -1.45466
C   4.10687  1.72218 -1.77624
H   4.27248  1.98531 -2.82591
C   4.54042  2.58484 -0.75439
H   5.04262  3.52456 -1.00559
C   4.33601  2.23273  0.59059
H   4.67451  2.89923  1.39047
C   3.70624  1.02021  0.91539
H   3.56186  0.73912  1.96378
O  -1.81750 -2.84381 -1.26168
C  -1.60058 -2.03094 -2.20221
H   3.12808 -0.15780 -2.24939
O  -0.98538 -0.88993 -2.05039
C  -2.08908 -2.33092 -3.61673
H  -3.13286 -1.98480 -3.71983
H  -1.48305 -1.80909 -4.37268
H  -2.07515 -3.41515 -3.80508

55
4a’
SCF(BP86)= -1414.45981108
G(298 K) = -1414.097870
SCF(PBE) = -1413.33391929
Low Freq. = 23.6521cm-1,
23.9106cm-1
C  -4.05184  1.73631 -0.41603
C  -2.76632  1.25087 -0.09869
N  -2.46477 -0.08435 -0.29458
C  -3.42351 -0.92256 -0.76927
C  -4.71405 -0.48984 -1.08516
C  -5.03120  0.86930 -0.91084
C  -1.65907  2.01507  0.47541
C  -1.76871  3.37349  0.85617
C  -0.70659  4.00932  1.50839
C   0.45951  3.27115  1.80162
C   0.57523  1.92698  1.41173
C  -0.45157  1.25940  0.69516
Ru -0.51571 -0.63817  0.04715
N  -0.86979 -1.15260  1.88174
C  -1.09854 -1.48944  2.99055
C  -1.41300 -1.95210  4.33751
H  -1.63865 -3.03237  4.33545
H  -0.56463 -1.77429  5.01966
H  -2.29176 -1.41444  4.73187
H  -2.69235  3.93131  0.66046
H  -0.78992  5.05979  1.80580
H   1.28465  3.75174  2.34158
H   1.49590  1.38710  1.65463
H  -4.27727  2.79636 -0.27110
H  -6.02870  1.24605 -1.15649
H  -5.44748 -1.20665 -1.46330
H  -3.10829 -1.96084 -0.90340
N   1.53293 -1.30379  0.28401
C   1.54012 -2.59000  0.75654
H   0.55329 -3.03055  0.90707
```

C 2.68850 -3.32798 1.03873
H 2.59049 -4.34789 1.41988
C 3.93914 -2.73768 0.80772
C 3.95842 -1.43690 0.30246
H 4.87050 -3.27893 0.99792
H 4.90836 -0.94840 0.07143
C 2.76223 -0.72480 0.04501
C 2.91140 0.65909 -0.49149
C 2.19192 1.12210 -1.61388
C 2.43708 2.40347 -2.13300
H 1.87817 2.74362 -3.01163
C 3.39302 3.24561 -1.54073
H 3.57710 4.24511 -1.94839
C 4.11517 2.79365 -0.42287
H 4.85999 3.44084 0.05178
C 3.88241 1.50995 0.09105
H 4.43974 1.17065 0.97081
O -0.74067 -2.57499 -1.27159
C -0.51940 -1.83343 -2.28718
H 1.44701 0.47275 -2.08033
O -0.29627 -0.56825 -2.11155
C -0.54522 -2.38776 -3.69605
H -1.53373 -2.19304 -4.14769
H 0.21179 -1.89285 -4.32348
H -0.37674 -3.47433 -3.68747

55
TS(4a'-6a)1
SCF(BP86)= -1414.44471580
G(298 K) = -1414.083256
SCF(PBE) = -1413.32478012
Low Freq. = -98.5348cm-1,
20.4975cm-1
C -1.91781 3.23490 1.11546
C -1.66229 1.92564 0.64442
C -0.40504 1.26988 0.88006
C 0.53421 1.97958 1.66868
C 0.27565 3.27536 2.14822
C -0.94799 3.91424 1.86251
C -2.66237 1.11921 -0.06362
N -2.24053 -0.16414 -0.35006
C -3.10142 -1.04020 -0.92687
C -4.40385 -0.68727 -1.28896
C -4.83742 0.62732 -1.04249
C -3.96356 1.52693 -0.42405
Ru -0.26330 -0.60202 0.10516
O -0.34325 -2.65605 -0.78183
C -0.28364 -2.51605 -2.06520
C -0.25601 -3.78629 -2.91234
H -2.87921 3.72202 0.91410
N 1.77533 -0.97782 0.45531
C 2.82480 -0.20127 0.00364
C 4.16120 -0.56084 0.27878
C 4.45864 -1.70918 1.01699
C 3.38922 -2.50553 1.45662
C 2.08656 -2.11545 1.14820
C 2.52537 1.00924 -0.79435
C 3.34016 2.16030 -0.69795
C 3.04632 3.30769 -1.44895
C 1.93419 3.32556 -2.31009
C 1.12290 2.18443 -2.41962

C 1.41378 1.03395 -1.66705
H 4.18882 2.17130 -0.00626
N -0.77962 -1.29424 1.78950
C -1.12257 -1.72099 2.83492
C -1.54704 -2.27882 4.11350
O -0.22792 -1.38170 -2.63077
H 4.96392 0.06380 -0.12129
H 5.49585 -1.98651 1.22518
H 3.55249 -3.42687 2.02204
H 1.23216 -2.71911 1.45735
H 3.68163 4.19411 -1.35334
H 1.70663 4.22230 -2.89558
H 0.26644 2.18051 -3.10199
H -1.79915 -3.34766 4.00756
H -0.74571 -2.18054 4.86516
H -2.43830 -1.74597 4.48537
H -2.70214 -2.04485 -1.08601
H -5.05745 -1.42997 -1.75341
H -5.84727 0.94340 -1.32107
H -4.28614 2.54968 -0.21219
H 1.49874 1.51425 1.90432
H 1.03155 3.79398 2.75063
H -1.14600 4.92575 2.23195
H 0.83373 0.11280 -1.86666
H 0.78708 -4.00860 -3.19897
H -0.82940 -3.64298 -3.84156
H -0.65193 -4.65018 -2.35775

55
Int(4a'-6a)
SCF(BP86)= -1414.45936386
G(298 K) = -1414.099055
SCF(PBE) = -1413.33816602
Low Freq. = 20.1623cm-1,
32.8415cm-1
C -2.88385 2.50463 1.21214
C -2.18500 1.39030 0.69052
C -0.78999 1.19342 0.95556
C -0.15105 2.15961 1.76620
C -0.84659 3.26624 2.28652
C -2.21652 3.44284 2.00910
C -2.81834 0.35918 -0.14009
N -1.96799 -0.66363 -0.51035
C -2.44432 -1.69066 -1.25937
C -3.76817 -1.75091 -1.70156
C -4.64386 -0.70912 -1.34853
C -4.16409 0.34277 -0.56237
Ru 0.00584 -0.50658 0.14504
O 0.54264 -2.53003 -0.58930
C 1.02240 -2.67544 -1.78943
C 1.46681 -4.09845 -2.14576
H -3.95117 2.64166 1.00255
N 1.98208 -0.20304 0.67820
C 2.70047 0.76300 -0.00630
C 4.06425 0.97982 0.27246
C 4.71441 0.22590 1.25374
C 3.97874 -0.75593 1.94082
C 2.63315 -0.94156 1.62165
C 1.92993 1.50246 -1.01164
C 2.34542 2.73472 -1.55822
C 1.53006 3.42039 -2.46969

```
C   0.28292  2.88185 -2.84431
C  -0.14205  1.65709 -2.31258
C   0.65828  0.95617 -1.37833
H   3.29754  3.17930 -1.25006
N  -0.48167 -1.44279  1.83047
C  -0.79965 -1.98064  2.82573
C  -1.18315 -2.64925  4.06208
O   1.16164 -1.75181 -2.63600
H   4.61197  1.73528 -0.29691
H   5.77325  0.39141  1.47159
H   4.43827 -1.38097  2.71107
H   2.02783 -1.69763  2.12573
H   1.86324  4.37705 -2.88396
H  -0.35266  3.41762 -3.55732
H  -1.09970  1.22697 -2.62333
H  -1.34543 -3.72409  3.87901
H  -0.39164 -2.53485  4.82098
H  -2.11614 -2.21364  4.45597
H  -1.71662 -2.47554 -1.48071
H  -4.09941 -2.59963 -2.30538
H  -5.68722 -0.72032 -1.67784
H  -4.82996  1.15935 -0.27129
H   0.91578  2.04951  1.99623
H  -0.31988  3.99603  2.91338
H  -2.75802  4.30394  2.41421
H   0.59372 -0.23966 -1.53712
H   2.50903 -4.24665 -1.81183
H   1.43425 -4.25470 -3.23452
H   0.84342 -4.85056 -1.63832

55
TS(4a'-6a)2
SCF(BP86)= -1414.45530857
G(298 K) = -1414.098443
SCF(PBE) = -1413.33317885
Low Freq. = -884.5481cm-1,
32.2023cm-1
C  -2.82120  2.53937  1.28281
C  -2.14860  1.42253  0.73289
C  -0.76133  1.17780  1.00376
C  -0.10339  2.10457  1.84683
C  -0.77338  3.21333  2.39433
C  -2.13621  3.43530  2.11272
C  -2.80041  0.43464 -0.13339
N  -1.97092 -0.60005 -0.52369
C  -2.47320 -1.59015 -1.30665
C  -3.79341 -1.60100 -1.76296
C  -4.64441 -0.54602 -1.38884
C  -4.14176  0.46799 -0.56781
Ru -0.00446 -0.50823  0.15758
O   0.53087 -2.53919 -0.65130
C   0.96246 -2.52955 -1.85521
C   1.40225 -3.83333 -2.49766
H  -3.88275  2.71115  1.06893
C   0.65129  0.89596 -1.28968
C   1.95779  1.42139 -0.99552
C   2.43519  2.58393 -1.63878
C   1.64159  3.24818 -2.58339
C   0.35796  2.75130 -2.88837
C  -0.12168  1.59591 -2.25587
H   3.42071  2.99105 -1.38623
C   2.71222  0.70142  0.03338
C   4.07411  0.90455  0.33530
C   4.69793  0.15638  1.33765
C   3.93946 -0.80754  2.02624
C   2.59675 -0.97870  1.68698
N   1.97133 -0.24646  0.72202
N  -0.52865 -1.53400  1.82892
C  -0.86109 -2.09957  2.80504
C  -1.26165 -2.80595  4.01522
O   1.04730 -1.45516 -2.55851
H   4.64187  1.64734 -0.23136
H   5.75477  0.31318  1.57206
H   4.37806 -1.42680  2.81326
H   1.97168 -1.71757  2.19314
H   2.01708  4.15076 -3.07608
H  -0.26662  3.26983 -3.62483
H  -1.11513  1.21443 -2.51829
H  -1.08853 -2.17324  4.90133
H  -2.33203 -3.06622  3.97111
H  -0.67711 -3.73422  4.12520
H  -1.77245 -2.39329 -1.54794
H  -4.14104 -2.42323 -2.39377
H  -5.68429 -0.51844 -1.72795
H  -4.78684  1.29457 -0.25816
H   0.95831  1.95937  2.08049
H  -0.23202  3.91030  3.04575
H  -2.65770  4.29843  2.53930
H   0.67304 -0.45266 -1.67540
H   2.48531 -3.79443 -2.70267
H   0.89244 -3.96757 -3.46534
H   1.18821 -4.68881 -1.84235

55
6a
SCF(BP86)= -1414.46456047
G(298 K) = -1414.103828
SCF(PBE) = -1413.34053554
Low Freq. = 16.7830cm-1,
28.4550cm-1
C  -2.95383  2.74449  0.65033
C  -2.23939  1.55779  0.36282
C  -0.86509  1.39350  0.75579
C  -0.27026  2.48443  1.44114
C  -0.98353  3.66150  1.72549
C  -2.33039  3.79767  1.33073
C  -2.82504  0.41112 -0.33607
N  -1.95095 -0.65343 -0.48231
C  -2.39435 -1.78216 -1.09898
C  -3.68662 -1.91498 -1.61197
C  -4.57992 -0.83637 -1.48041
C  -4.14096  0.32421 -0.83527
Ru -0.04140 -0.37527  0.28002
O   0.62333 -2.54638 -0.13647
C   1.21565 -2.92295 -1.17115
C   1.69272 -4.33548 -1.38098
H  -4.00195  2.84935  0.34508
C   0.67129  0.58062 -1.38939
C   2.00060  1.12648 -1.25559
C   2.59183  1.90493 -2.27595
C   1.88983  2.17085 -3.45925
C   0.58823  1.65547 -3.62195
```

C -0.00149 0.88189 -2.60818
H 3.60067 2.31554 -2.15004
C 2.67073 0.81308 0.01026
C 3.99083 1.16928 0.35192
C 4.52776 0.80860 1.59180
C 3.72210 0.07904 2.48456
C 2.42162 -0.25337 2.10132
N 1.88159 0.09645 0.89925
N -0.62664 -1.20891 2.08972
C -0.99226 -1.66160 3.11407
C -1.43682 -2.21806 4.38659
O 1.47349 -2.09596 -2.18487
H 4.59606 1.73019 -0.36555
H 5.55234 1.08642 1.85613
H 4.09012 -0.23274 3.46587
H 1.76028 -0.81663 2.76392
H 2.35084 2.77452 -4.24790
H 0.03252 1.86170 -4.54481
H -1.01658 0.49588 -2.76403
H -1.37277 -1.45672 5.18179
H -2.48183 -2.56192 4.31280
H -0.80454 -3.07566 4.67029
H -1.66783 -2.59636 -1.16217
H -3.98093 -2.84815 -2.09963
H -5.60023 -0.90217 -1.86993
H -4.81791 1.17439 -0.71380
H 0.77649 2.41088 1.75950
H -0.48827 4.48208 2.25941
H -2.88488 4.71537 1.55294
H 1.11902 -1.18347 -1.90336
H 2.79031 -4.34466 -1.48667
H 1.27098 -4.74116 -2.31499
H 1.39796 -4.96462 -0.53162

47
Int(6a-7a)
SCF(BP86)= -1185.34627881
G(298 K) = -1185.040991
SCF(PBE) = -1184.37686993
Low Freq. = 23.5447cm-1, 27.2162cm-1
N -2.01988 0.77678 -0.20267
C -2.88717 -0.30682 -0.20075
C -4.27212 -0.11185 -0.03525
C -4.79452 1.17523 0.13647
C -3.90760 2.26594 0.14163
C -2.54284 2.02336 -0.02993
C -2.21372 -1.60073 -0.36795
C -0.77951 -1.53248 -0.48119
C -0.10438 -2.77625 -0.60498
C -0.79099 -4.00249 -0.63805
C -2.19671 -4.03513 -0.54363
C -2.90461 -2.83332 -0.40717
Ru -0.01162 0.34387 -0.53183
N 0.53568 2.32166 -0.86530
C 0.84279 3.42238 -1.15948
C 1.21308 4.78343 -1.52642
C 0.57320 0.24127 1.35104
C 1.94938 -0.13926 1.55439
C 2.50094 -0.21566 2.85456
C 1.71450 0.06777 3.97722
C 0.36156 0.42820 3.79885
C -0.19753 0.50937 2.51356
C 2.69718 -0.42742 0.33181
N 1.93578 -0.27109 -0.81463
C 2.51008 -0.51303 -2.02377
C 3.84002 -0.91493 -2.16666
C 4.62508 -1.07627 -1.00918
C 4.04529 -0.82922 0.23982
H 3.55149 -0.49942 2.99073
H -3.99815 -2.86362 -0.33075
H -4.93940 -0.97806 -0.03840
H -5.87050 1.32457 0.26645
H -4.26009 3.29218 0.27559
H -1.81608 2.83942 -0.03512
H -2.73360 -4.98916 -0.57215
H -0.23073 -4.94048 -0.73717
H 0.99012 -2.78534 -0.67962
H 1.26477 5.42168 -0.62863
H 2.19750 4.79561 -2.02310
H 0.46539 5.20972 -2.21601
H 1.85680 -0.37316 -2.89137
H 4.24654 -1.09572 -3.16521
H 5.67085 -1.38945 -1.08191
H 4.63495 -0.94714 1.15337
H -1.25104 0.79032 2.40653
H -0.26236 0.64667 4.67422
H 2.14512 0.00720 4.98192

53
7a
SCF(BP86)= -1318.12843918
G(298 K) = -1317.781488
SCF(PBE) = -1317.04958142
Low Freq. = 3.0125cm-1, 15.5800cm-1
C 2.74272 -2.32457 1.81459
C 2.09848 -1.34021 1.02809
C 0.71912 -0.98979 1.23441
C 0.04654 -1.69122 2.26899
C 0.68554 -2.66966 3.04996
C 2.03983 -2.99124 2.82569
C 2.77013 -0.60683 -0.04894
C 4.11206 -0.77455 -0.44966
C 4.64217 -0.01656 -1.49812
C 3.80971 0.91702 -2.14104
C 2.48880 1.04913 -1.70752
N 1.95884 0.31561 -0.69056
Ru -0.00002 0.46146 -0.00030
N -0.56554 1.95766 -1.36105
N 0.56523 1.95903 1.35909
C 0.90348 2.79149 2.12081
C 1.31584 3.82157 3.06768
H 1.40848 3.39630 4.08077
H 2.29044 4.24606 2.77484
H 0.57284 4.63582 3.09543
H 4.73754 -1.50695 0.06810
H 5.68298 -0.14934 -1.80843
H 4.17141 1.53736 -2.96556
H 1.80498 1.76154 -2.17525
H 3.79619 -2.57556 1.64126
H 2.53886 -3.75332 3.43361

```
H   0.12749 -3.18812  3.83976
H  -1.00846 -1.46638  2.46941
C  -0.90399  2.78954 -2.12332
C  -1.31702  3.81896 -3.07062
H  -1.38518  3.39908 -4.08788
H  -2.30340  4.22537 -2.79193
H  -0.58726  4.64550 -3.08022
N  -1.95886  0.31599  0.69011
C  -2.48898  1.05052  1.70625
H  -1.80530  1.76356  2.17323
C  -3.80989  0.91866  2.13987
H  -4.17173  1.53984  2.96369
C  -4.64215 -0.01579  1.49795
C  -4.11187 -0.77484  0.45034
H  -5.68294 -0.14842  1.80838
H  -4.73721 -1.50791 -0.06663
C  -2.76996 -0.60730  0.04947
C  -2.09816 -1.34169 -1.02678
C  -0.71888 -0.99118 -1.23351
C  -0.04619 -1.69358 -2.26737
H   1.00876 -1.46874 -2.46806
C  -0.68501 -2.67297 -3.04730
H  -0.12687 -3.19214 -3.83656
C  -2.03923 -2.99459 -2.82265
H  -2.53811 -3.75742 -3.42975
C  -2.74221 -2.32702 -1.81221
H  -3.79563 -2.57804 -1.63859
```

```
55
4b
SCF(BP86)= -1414.46521132
G(298 K) = -1414.103559
SCF(PBE) = -1413.34058899
Low Freq. = 20.3478cm-1,
32.0030cm-1
C  -4.21134  0.43642 -1.26846
C  -3.03161  0.20122 -0.53190
N  -1.82167  0.70523 -0.98130
C  -1.78602  1.41492 -2.14080
C  -2.92598  1.66360 -2.90825
C  -4.16451  1.16827 -2.45860
C  -2.91134 -0.57351  0.70330
C  -4.00621 -1.19788  1.34618
C  -3.80613 -1.95627  2.50478
C  -2.49957 -2.09849  3.02068
C  -1.40775 -1.47945  2.39231
C  -1.56826 -0.68549  1.22439
Ru -0.21135  0.38970  0.26175
N   1.20436  0.12963  1.61670
C   2.01092  0.03328  2.47076
C   3.02741 -0.10823  3.50535
H   3.18890  0.85364  4.02022
H   2.72218 -0.85813  4.25456
H   3.98271 -0.42703  3.05496
H  -5.01942 -1.09264  0.94031
H  -4.65428 -2.43878  3.00108
H  -2.33557 -2.70082  3.92271
H  -0.40674 -1.60757  2.81928
H  -5.16249  0.04035 -0.90250
H  -5.07928  1.35090 -3.03040
H  -2.84052  2.23775 -3.83439
H  -0.80095  1.79567 -2.42275
N   0.41477 -1.28364 -0.84944
C  -0.55770 -2.15597 -1.27093
H  -1.58229 -1.85907 -1.04930
C  -0.30427 -3.37142 -1.90555
H  -1.14814 -4.00207 -2.19865
C   1.02329 -3.75798 -2.13598
C   2.02913 -2.86725 -1.74816
H   1.26782 -4.70798 -2.61931
H   3.08051 -3.09150 -1.94731
C   1.72135 -1.63639 -1.13259
C   2.85335 -0.70594 -0.86263
C   2.85887  0.60121 -1.39399
C   3.98659  1.42328 -1.24449
H   3.97979  2.43169 -1.67086
C   5.12171  0.95526 -0.56039
H   5.99969  1.59932 -0.44566
C   5.12738 -0.34733 -0.03192
H   6.00830 -0.72284  0.49914
C   4.00462 -1.17488 -0.18920
H   4.01154 -2.19023  0.22163
O   0.70057  2.53173 -0.44542
C   0.00001  3.01331  0.50196
H   1.98053  0.96965 -1.92929
O  -0.72401  2.22686  1.23719
C   0.01616  4.49594  0.82191
H   0.07320  5.08649 -0.10529
H  -0.87345  4.78765  1.39931
H   0.91224  4.72998  1.42299
```

```
55
4c
SCF(BP86)= -1414.46312567
G(298 K) = -1414.101297
SCF(PBE) = -1413.33910484
Low Freq. = 24.3966cm-1,
25.8405cm-1
C   3.64408  0.57789  0.73588
C   2.70007 -0.43460  0.45207
C   2.76242 -1.10240 -0.79178
C   3.75050 -0.75844 -1.72794
C   4.67914  0.25805 -1.44318
C   4.61939  0.92921 -0.21009
C   1.76123 -0.89386  1.51302
C   2.30485 -1.19339  2.78177
C   1.51260 -1.74593  3.79184
C   0.16971 -2.01881  3.49586
C  -0.32222 -1.68137  2.23432
N   0.42609 -1.09475  1.25126
Ru -0.74782 -0.45714 -0.42322
N  -1.89089  0.00410 -1.94681
H   3.37178 -1.01818  2.94250
H   1.93896 -1.98124  4.77139
H  -0.50030 -2.47900  4.22692
H  -1.36471 -1.86196  1.97192
H   3.60357  1.10025  1.69781
H   5.33588  1.72466  0.01937
H   5.44766  0.52315 -2.17671
H   3.79729 -1.29091 -2.68401
C  -2.56856  0.27186 -2.87400
C  -3.40394  0.59313 -4.02502
```

```
H  -3.65987  1.66585 -4.02380
H  -2.87482  0.36246 -4.96510
H  -4.34089  0.01170 -3.99870
N   0.19351  1.36581 -0.64502
C   1.10431  1.64749 -1.61293
H   1.38255  0.80741 -2.25318
C   1.68133  2.91013 -1.77315
H   2.41230  3.07027 -2.56963
C   1.31489  3.93952 -0.88874
C   0.35216  3.67247  0.09040
H   1.76084  4.93532 -0.97103
H   0.02149  4.46232  0.77079
C  -0.22527  2.39097  0.18766
C  -1.33688  2.00384  1.05746
C  -1.81160  0.66842  0.83076
C  -2.96835  0.27094  1.54688
H  -3.38455 -0.73199  1.38489
C  -3.59291  1.12943  2.46983
H  -4.48215  0.78843  3.01401
C  -3.08393  2.42311  2.70480
H  -3.56754  3.08507  3.43060
C  -1.95908  2.86142  1.99221
H  -1.57248  3.87337  2.16380
O  -1.69076 -2.39227 -0.30755
C  -0.92841 -3.00059 -1.15999
O   0.02512 -2.37266 -1.72665
H   2.03207 -1.88516 -1.01930
C  -1.21493 -4.45365 -1.49123
H  -1.78798 -4.93885 -0.68742
H  -0.27493 -4.99890 -1.66616
H  -1.80971 -4.50574 -2.42001

55
TS(4c-6d)1
SCF(BP86)= -1414.46127299
G(298 K) = -1414.099522
SCF(PBE) = -1413.34009291
Low Freq. = -31.2827cm-1,
19.2610cm-1
C   3.76011  0.72625  0.76983
C   2.76477 -0.24964  0.53768
C   2.70367 -0.88501 -0.72394
C   3.62218 -0.54076 -1.73055
C   4.60242  0.43959 -1.49623
C   4.66704  1.07355 -0.24312
C   1.85804 -0.67146  1.63704
C   2.40484 -0.91920  2.91383
C   1.60705 -1.41355  3.94995
C   0.25785 -1.67770  3.67355
C  -0.23549 -1.40071  2.39766
N   0.52047 -0.87696  1.38763
Ru -0.51653 -0.42641 -0.41588
N  -1.41992 -0.16545 -2.13953
H   3.47438 -0.75100  3.06431
H   2.03340 -1.61000  4.93794
H  -0.41493 -2.08946  4.43049
H  -1.28301 -1.57491  2.14921
H   3.80831  1.23092  1.74065
H   5.42393  1.84135 -0.05318
H   5.31501  0.70555 -2.28378
H   3.57352 -1.04654 -2.70077
C  -1.94363 -0.01570 -3.18517
C  -2.57380  0.15698 -4.48823
H  -2.92761  1.19459 -4.60780
H  -1.85491 -0.06131 -5.29596
H  -3.43724 -0.52094 -4.59492
N   0.08256  1.56890 -0.45808
C   1.12059  2.07706 -1.17318
H   1.71564  1.34463 -1.72392
C   1.43863  3.43833 -1.19750
H   2.28876  3.78084 -1.79297
C   0.66029  4.33032 -0.44063
C  -0.42738  3.82562  0.27953
H   0.88963  5.40000 -0.42191
H  -1.06764  4.49862  0.85648
C  -0.72330  2.44862  0.24738
C  -1.88660  1.80560  0.85938
C  -2.00301  0.40380  0.58017
C  -3.16005 -0.26301  1.05562
H  -3.28665 -1.33034  0.83910
C  -4.13439  0.41772  1.80602
H  -5.01641 -0.12444  2.16806
C  -3.98838  1.78955  2.09871
H  -4.74733  2.31374  2.68841
C  -2.86781  2.48141  1.62021
H  -2.76220  3.55093  1.83764
O  -1.27663 -2.39897 -0.28909
C  -0.58313 -3.21136 -1.03340
O   0.42187 -2.85265 -1.70588
H   1.95381 -1.66556 -0.91336
C  -1.07397 -4.65645 -1.08703
H  -1.71699 -4.90004 -0.22812
H  -0.21780 -5.34799 -1.12174
H  -1.65952 -4.80788 -2.01095

55
Int(4c-6d)
SCF(BP86)= -1414.46467222
G(298 K) = -1414.103332
SCF(PBE) = -1413.34134318
Low Freq. = 15.8933cm-1,
29.5374cm-1
C   3.85480  1.04997  0.56062
C   2.71959  0.20925  0.54249
C   2.26913 -0.31437 -0.70010
C   2.97816 -0.02123 -1.88297
C   4.10473  0.81561 -1.85129
C   4.54139  1.35146 -0.62507
C   1.96068 -0.08859  1.77431
C   2.56567 -0.10794  3.04685
C   1.81250 -0.41927  4.18297
C   0.45140 -0.72389  4.02008
C  -0.09900 -0.69510  2.73750
N   0.62016 -0.37408  1.62557
Ru -0.21956 -0.41143 -0.28673
N  -0.84186 -0.56574 -2.15857
H   3.63593  0.09718  3.12983
H   2.28032 -0.43886  5.17138
H  -0.18302 -0.98560  4.87097
H  -1.15267 -0.92172  2.55982
H   4.18375  1.50182  1.50200
H   5.41350  2.01192 -0.59265
```

```
H   4.64433  1.04724 -2.77528
H   2.64188 -0.45800 -2.82879
C  -1.19999 -0.66156 -3.27603
C  -1.61314 -0.79224 -4.66782
H  -2.51775 -0.19121 -4.85774
H  -0.81290 -0.44456 -5.34255
H  -1.83790 -1.84593 -4.90345
N  -0.29584  1.68798 -0.41720
C   0.67684  2.50948 -0.89515
H   1.58421  2.01513 -1.24533
C   0.54822  3.90030 -0.95001
H   1.37143  4.49984 -1.34715
C  -0.63997  4.49001 -0.48918
C  -1.65629  3.65788 -0.01178
H  -0.77458  5.57561 -0.50902
H  -2.59806  4.08655  0.34068
C  -1.48294  2.25912  0.01165
C  -2.48875  1.28818  0.44591
C  -2.08696 -0.08244  0.34237
C  -3.03397 -1.06743  0.71879
H  -2.75137 -2.12310  0.64607
C  -4.30991 -0.71074  1.18825
H  -5.02170 -1.49494  1.47382
C  -4.68417  0.64450  1.29466
H  -5.67825  0.91854  1.66256
C  -3.77344  1.64060  0.92201
H  -4.06754  2.69352  1.00313
O  -0.48687 -2.48751  0.02103
C   0.23730 -3.39638 -0.58089
O   1.19874 -3.17613 -1.35461
H   1.57005 -1.20179 -0.76212
C  -0.20903 -4.83352 -0.28786
H  -0.55587 -4.94190  0.75160
H   0.61184 -5.53899 -0.48484
H  -1.05409 -5.09553 -0.94886

55
TS(4c-6d)2
SCF(BP86)= -1414.44622024
G(298 K) = -1414.087990
SCF(PBE) = -1413.32226382
Low Freq. = -289.6258cm-1,
30.4605cm-1
C   3.70202  1.49616  0.61814
C   2.55098  0.67520  0.60263
C   1.96167  0.21494 -0.62886
C   2.63545  0.61280 -1.81984
C   3.78281  1.42167 -1.80746
C   4.31909  1.87084 -0.58295
C   1.85268  0.27634  1.83200
C   2.38602  0.38672  3.13435
C   1.65796 -0.05854  4.24032
C   0.38849 -0.62509  4.02954
C  -0.10137 -0.70749  2.72498
N   0.59391 -0.26668  1.64064
Ru -0.10494 -0.41061 -0.32100
N  -0.53563 -0.58707 -2.24235
H   3.38608  0.80725  3.26788
H   2.07549  0.02426  5.24825
H  -0.21919 -0.99709  4.85861
H  -1.08664 -1.12587  2.50466
```

```
H   4.10878  1.86972  1.56530
H   5.20797  2.50997 -0.56731
H   4.26471  1.70783 -2.75028
H   2.24096  0.27441 -2.78743
C  -0.76795 -0.69695 -3.39270
C  -1.03825 -0.83369 -4.81927
H  -0.62111  0.02212 -5.37633
H  -0.57920 -1.75832 -5.20787
H  -2.12401 -0.87832 -5.00760
N  -0.69533  1.60564 -0.34149
C   0.08949  2.66112 -0.69088
H   1.10800  2.41013 -0.99033
C  -0.35344  3.98624 -0.68136
H   0.33404  4.78322 -0.97686
C  -1.67451  4.25620 -0.28968
C  -2.49608  3.18291  0.06141
H  -2.05736  5.28093 -0.26364
H  -3.53193  3.36063  0.36159
C  -2.00818  1.85905  0.02806
C  -2.79624  0.66675  0.35078
C  -2.08892 -0.57210  0.22050
C  -2.82860 -1.74852  0.51159
H  -2.33191 -2.72283  0.42472
C  -4.17603 -1.70364  0.91041
H  -4.71442 -2.63556  1.12481
C  -4.84357 -0.46786  1.03689
H  -5.89231 -0.43181  1.35011
C  -4.15192  0.71664  0.75598
H  -4.67438  1.67562  0.85561
O   0.04868 -2.53667 -0.16842
C   1.14309 -3.11403 -0.46167
O   2.20090 -2.45120 -0.83154
H   1.88800 -1.34316 -0.75500
C   1.25558 -4.61529 -0.38782
H   2.05420 -4.89415  0.31895
H   1.53845 -5.01534 -1.37533
H   0.30393 -5.05923 -0.06711

55
6d
SCF(BP86)= -1414.44625089
G(298 K) = -1414.086561
SCF(PBE) = -1413.32210322
Low Freq. = 18.2971cm-1,
26.8449cm-1
C   3.72335  1.54648  0.51181
C   2.54440  0.76702  0.56480
C   1.94992  0.19916 -0.61936
C   2.64727  0.45667 -1.83584
C   3.82260  1.22312 -1.89156
C   4.36453  1.77576 -0.71276
C   1.82300  0.51545  1.81901
C   2.32863  0.78136  3.11023
C   1.57712  0.47209  4.24629
C   0.31048 -0.11507  4.07891
C  -0.15210 -0.35327  2.78355
N   0.56689 -0.04791  1.66843
Ru -0.09251 -0.42576 -0.27685
N  -0.47052 -0.82372 -2.17458
H   3.32551  1.21750  3.21404
H   1.97394  0.67617  5.24535
```

H -0.31569 -0.38401 4.93367
H -1.13362 -0.79497 2.59438
H 4.13628 1.99909 1.42121
H 5.27561 2.38190 -0.75092
H 4.32202 1.39559 -2.85297
H 2.24945 0.03935 -2.77110
C -0.66533 -1.06786 -3.31153
C -0.88885 -1.37421 -4.71981
H -0.48843 -0.56986 -5.35970
H -0.38339 -2.31666 -4.99024
H -1.96600 -1.48460 -4.92961
N -0.73378 1.55239 -0.52086
C 0.03173 2.58156 -0.97629
H 1.05842 2.32064 -1.23762
C -0.43976 3.88956 -1.11257
H 0.23294 4.66560 -1.48728
C -1.77007 4.16997 -0.76079
C -2.57166 3.12221 -0.30275
H -2.17514 5.18281 -0.84693
H -3.61387 3.30784 -0.03064
C -2.05628 1.81301 -0.19123
C -2.82347 0.64375 0.24627
C -2.08852 -0.58656 0.25456
C -2.81150 -1.74114 0.65518
H -2.29538 -2.70929 0.67720
C -4.16608 -1.68449 1.02747
H -4.69005 -2.60054 1.32883
C -4.85962 -0.45645 1.01622
H -5.91401 -0.41055 1.30865
C -4.18648 0.70713 0.62474
H -4.72954 1.65989 0.61641
O 0.10020 -2.52681 0.11169
C 1.18553 -3.13715 -0.11815
O 2.24913 -2.51865 -0.56988
H 1.95448 -1.46593 -0.61466
C 1.31136 -4.61761 0.12053
H 2.11280 -4.80952 0.85270
H 1.59673 -5.12246 -0.81693
H 0.36319 -5.02891 0.49035

55
4c’
SCF(BP86)= -1414.45960026
G(298 K) = -1414.097874
SCF(PBE) = -1413.33531459
Low Freq. = 25.4519cm-1,
31.7366cm-1
C 2.79442 1.25114 -0.42530
C 2.74898 0.17050 0.48343
C 3.71130 -0.85965 0.37076
C 4.69437 -0.81266 -0.62908
C 4.74293 0.27443 -1.51861
C 3.78918 1.30170 -1.41305
C 1.82755 0.20144 1.65122
C 2.38500 -0.05343 2.92580
C 1.62805 0.09643 4.08988
C 0.30690 0.54792 3.95930
C -0.19966 0.76522 2.67786
N 0.50488 0.55873 1.52341
Ru -0.82423 0.60239 -0.16785
N -2.19474 0.73528 -1.55725

H 3.44050 -0.33108 2.98321
H 2.06843 -0.09932 5.07195
H -0.33111 0.73055 4.82785
H -1.22105 1.12057 2.53306
H 2.04314 2.04320 -0.35461
H 3.82175 2.15160 -2.10329
H 5.51879 0.31955 -2.29002
H 5.42596 -1.62339 -0.70896
C -3.03093 0.83240 -2.38309
C -4.07723 0.96230 -3.39025
H -3.73910 1.60745 -4.21854
H -4.33448 -0.02716 -3.80380
H -4.98750 1.40441 -2.95092
N -1.70598 -1.06592 0.63253
C -2.72505 -1.02668 1.53112
H -3.10782 -0.02886 1.76530
C -3.26068 -2.17402 2.12341
H -4.07953 -2.07904 2.84110
C -2.72026 -3.42559 1.77868
C -1.69070 -3.48022 0.83294
H -3.10285 -4.34418 2.23363
H -1.26531 -4.44258 0.53462
C -1.19830 -2.29660 0.24698
C -0.20486 -2.19095 -0.81917
C 0.07845 -0.83952 -1.21954
C 0.88614 -0.68305 -2.37494
H 1.11011 0.32687 -2.73782
C 1.43549 -1.78693 -3.04716
H 2.06479 -1.62626 -3.93127
C 1.19416 -3.10224 -2.59331
H 1.63520 -3.95836 -3.11451
C 0.36583 -3.30250 -1.48307
H 0.14771 -4.32397 -1.14795
O -0.05670 2.54308 -0.80774
C -0.83181 3.22903 -0.03072
O -1.64383 2.62680 0.75023
H 3.68112 -1.70714 1.06399
C -0.78245 4.74373 -0.09128
H 0.22140 5.09280 -0.37596
H -1.07435 5.17948 0.87575
H -1.49552 5.10111 -0.85492

55
TS(4c'-6b)1
SCF(BP86)= -1414.44506623
G(298 K) = -1414.083477
SCF(PBE) = -1413.32471202
Low Freq. = -70.7670cm-1,
8.8442cm-1
C 3.20306 2.46011 0.97360
C 2.09085 1.79402 0.41980
N 2.03329 0.40985 0.46318
C 3.07169 -0.29453 0.99536
C 4.19299 0.33151 1.54281
C 4.25632 1.73681 1.54134
C 0.95224 2.39897 -0.26714
C 0.82418 3.79085 -0.48393
C -0.25061 4.28363 -1.23288
C -1.18687 3.37641 -1.77021
C -1.06530 1.99460 -1.53965
C -0.00752 1.44968 -0.76691

Ru 0.41961 -0.48247 -0.28953
N 1.32875 -0.73007 -2.01684
C 1.87776 -0.88804 -3.04680
C 2.53903 -1.10279 -4.32786
H 2.61173 -2.18142 -4.54608
H 1.97559 -0.61637 -5.14167
H 3.55779 -0.68121 -4.30748
H 1.56633 4.49001 -0.08009
H -0.35519 5.35963 -1.40695
H -2.02293 3.75401 -2.37193
H -1.82301 1.32465 -1.96215
H 3.23523 3.55269 0.94724
H 5.11747 2.25823 1.96935
H 4.99648 -0.27951 1.96201
H 2.95049 -1.38132 0.96788
C -2.62105 -0.89968 -0.57883
C -3.47079 -0.85494 -1.69733
H -3.30847 -1.55297 -2.52535
C -4.52834 0.06868 -1.74912
H -5.19028 0.10111 -2.62062
C -4.73545 0.94949 -0.67225
C -3.89251 0.90594 0.44789
H -5.55286 1.67692 -0.70671
H -4.04758 1.61129 1.27097
C -2.82238 -0.01551 0.50451
C -1.95995 -0.10135 1.70721
N -0.61513 -0.36215 1.55890
C 0.11945 -0.54391 2.69438
H 1.17202 -0.78497 2.54084
C -0.39994 -0.42942 3.98458
H 0.25844 -0.58424 4.84325
C -1.75938 -0.12211 4.14102
H -2.20874 -0.02533 5.13346
C -2.53561 0.02760 2.98862
H -3.60994 0.21450 3.06230
O 1.07806 -2.50930 0.37212
C 0.39822 -3.40656 -0.26698
H -1.83970 -1.67688 -0.53580
O -0.51101 -3.12873 -1.10225
C 0.70749 -4.86873 0.05880
H 0.01521 -5.21905 0.84498
H 0.55341 -5.50425 -0.82683
H 1.73544 -4.99039 0.43304

55
Int(4c'-6b)
SCF(BP86)= -1414.46173889
G(298 K) = -1414.100803
SCF(PBE) = -1413.33972035
Low Freq. = 16.5600cm-1,
24.7263cm-1
C -4.00326 -1.03665 0.92554
C -2.66464 -0.92919 0.49131
N -2.09577 0.31411 0.32782
C -2.83211 1.43079 0.57093
C -4.16009 1.37207 1.00000
C -4.75592 0.11200 1.18345
C -1.76693 -2.04026 0.16637
C -2.17208 -3.39158 0.27708
C -1.29247 -4.42397 -0.06468
C -0.00300 -4.09495 -0.52220
C 0.40167 -2.75212 -0.62685
C -0.45146 -1.67160 -0.28622
Ru -0.11561 0.37014 -0.31688
N -0.73944 0.47814 -2.20496
C -1.10941 0.55672 -3.31845
C -1.54655 0.65493 -4.70539
H -1.53179 1.70587 -5.03879
H -0.88116 0.06794 -5.36006
H -2.57337 0.26768 -4.81012
H -3.17906 -3.64131 0.63026
H -1.60662 -5.46930 0.02072
H 0.69637 -4.89290 -0.80044
H 1.41405 -2.54977 -0.98820
H -4.44969 -2.02555 1.05602
H -5.79369 0.02732 1.51963
H -4.70929 2.29915 1.18333
H -2.29917 2.37236 0.40950
C 1.98122 -0.11317 -0.76638
C 2.56944 -0.46277 -2.00807
H 2.07117 -0.16118 -2.93517
C 3.76900 -1.18433 -2.05875
H 4.19939 -1.46071 -3.02729
C 4.42584 -1.55159 -0.86631
C 3.87988 -1.19001 0.37296
H 5.36081 -2.11905 -0.90443
H 4.38611 -1.50233 1.29243
C 2.67211 -0.46327 0.43981
C 2.02313 -0.09297 1.70215
N 0.70820 0.31680 1.58574
C 0.03911 0.69505 2.70792
H -0.99429 1.01426 2.55721
C 0.61690 0.67920 3.97850
H 0.02609 0.99397 4.84244
C 1.95229 0.26120 4.11071
H 2.43986 0.24145 5.08955
C 2.65275 -0.11997 2.96327
H 3.69873 -0.42882 3.03299
O -0.24653 2.56193 -0.13679
C 0.72586 3.33905 -0.52426
H 1.47105 0.98515 -0.81768
O 1.81027 2.95230 -1.02855
C 0.46979 4.83388 -0.30082
H 0.45088 5.04957 0.78136
H 1.25669 5.44115 -0.77088
H -0.51344 5.12259 -0.70681

55
TS(4c'-6b)2
SCF(BP86)= -1414.45619535
G(298 K) = -1414.098881
SCF(PBE) = -1413.33351022
Low Freq. = -935.9643cm-1,
24.8905cm-1
C 4.15799 0.76442 0.67913
C 2.79851 0.71049 0.30206
N 2.11331 -0.48163 0.36387
C 2.75931 -1.60222 0.78049
C 4.10297 -1.60065 1.16424

C 4.81562 -0.38988 1.11336
C 1.99290 1.83860 -0.17953
C 2.52886 3.14355 -0.29670
C 1.73962 4.19714 -0.76951
C 0.40390 3.93785 -1.12890
C -0.13227 2.64363 -1.00971
C 0.62757 1.54458 -0.53275
Ru 0.07847 -0.41724 -0.24167
N 0.60499 -0.88971 -2.09803
C 0.91508 -1.18957 -3.19354
C 1.27857 -1.56306 -4.55516
H 1.12807 -2.64462 -4.70961
H 0.65875 -1.01499 -5.28434
H 2.33809 -1.32664 -4.74823
H 3.57006 3.34082 -0.01699
H 2.15844 5.20487 -0.85873
H -0.22717 4.75214 -1.50598
H -1.17520 2.48834 -1.30157
H 4.69670 1.71396 0.62799
H 5.86928 -0.34772 1.40553
H 4.57383 -2.53094 1.49260
H 2.14872 -2.50892 0.79636
C -1.88037 0.15919 -0.72902
C -2.47536 0.32780 -2.01215
H -1.97810 -0.10820 -2.88639
C -3.67414 1.03126 -2.18730
H -4.09524 1.14925 -3.19265
C -4.34356 1.59009 -1.07765
C -3.80214 1.43292 0.20423
H -5.27844 2.14275 -1.21484
H -4.31551 1.88465 1.06068
C -2.59323 0.72574 0.38845
C -1.95349 0.55559 1.69376
N -0.67556 0.02642 1.63965
C -0.00408 -0.18961 2.80269
H 0.99868 -0.60992 2.69688
C -0.54084 0.10517 4.05693
H 0.04892 -0.09077 4.95607
C -1.83569 0.64989 4.12664
H -2.29017 0.89284 5.09165
C -2.53808 0.86944 2.93927
H -3.55176 1.27743 2.96743
O -0.00897 -2.58811 0.27395
C -1.11986 -3.16169 0.00263
H -1.65107 -1.24927 -0.58660
O -2.11480 -2.53346 -0.51614
C -1.28654 -4.63871 0.30426
H -2.10642 -4.77819 1.02814
H -1.56906 -5.17699 -0.61513
H -0.36054 -5.06595 0.71270

55
6b
SCF(BP86)= -1414.46394065
G(298 K) = -1414.102993
SCF(PBE) = -1413.33968777
Low Freq. = 12.0391cm-1,
25.4042cm-1
C -4.29313 -0.41886 0.47743
C -2.92385 -0.44561 0.13281
N -2.09064 0.57891 0.52607
C -2.60531 1.61058 1.24296
C -3.95152 1.68608 1.61240
C -4.81270 0.64604 1.21992
C -2.25588 -1.50143 -0.64184
C -2.94987 -2.64478 -1.10686
C -2.28704 -3.63334 -1.84261
C -0.91449 -3.47613 -2.11715
C -0.21981 -2.34476 -1.65731
C -0.85008 -1.31685 -0.90688
Ru -0.03713 0.37170 -0.13802
N -0.44290 1.38777 -1.78556
C -0.67988 2.00917 -2.75995
C -0.95193 2.78110 -3.96705
H -0.71426 3.84604 -3.80455
H -0.34265 2.41292 -4.80981
H -2.01618 2.70181 -4.24544
H -4.01779 -2.76821 -0.89218
H -2.83109 -4.51467 -2.19811
H -0.38276 -4.24246 -2.69490
H 0.84645 -2.25182 -1.88897
H -4.94931 -1.23361 0.16015
H -5.87374 0.66654 1.48716
H -4.31065 2.54138 2.19114
H -1.88694 2.38887 1.51703
C 1.83106 -0.17514 -0.75097
C 2.46859 0.02193 -2.01411
H 1.95722 0.62255 -2.77695
C 3.72432 -0.52416 -2.32086
H 4.17026 -0.34420 -3.30699
C 4.41733 -1.30673 -1.37304
C 3.83649 -1.53246 -0.11880
H 5.39585 -1.73516 -1.61315
H 4.37034 -2.15085 0.61285
C 2.57321 -0.98115 0.19467
C 1.90034 -1.19941 1.47470
N 0.63009 -0.64903 1.54460
C -0.08394 -0.79208 2.69397
H -1.07944 -0.34170 2.68660
C 0.40212 -1.46550 3.81615
H -0.22096 -1.54523 4.71077
C 1.69012 -2.02883 3.75906
H 2.10595 -2.56506 4.61742
C 2.43334 -1.89062 2.58412
H 3.43834 -2.31597 2.51786
O 0.36884 2.34552 0.94996
C 1.42710 2.99704 0.80058
H 2.12374 1.64100 -0.30576
O 2.42399 2.55783 0.03790
C 1.67356 4.32677 1.46091
H 2.57595 4.26959 2.09133
H 1.85944 5.09534 0.69276
H 0.80838 4.61284 2.07218

55
4d
SCF(BP86)= -1414.45935479
G(298 K) = -1414.097730
SCF(PBE) = -1413.33266234
Low Freq. = 21.0038cm-1,
26.4436cm-1
C 4.55654 -0.57461 -0.69641

```
C   3.21857 -0.15201 -0.52061
C   2.24087 -0.99148  0.11251
C   2.67817 -2.27388  0.53106
C   4.00771 -2.69519  0.35115
C   4.95304 -1.84535 -0.25953
C   2.70179  1.13957 -0.97877
C   3.43165  2.12647 -1.67031
C   2.80666  3.29784 -2.10990
C   1.43283  3.46085 -1.86160
C   0.75075  2.46214 -1.16270
N   1.35556  1.33439 -0.69634
Ru  0.43690 -0.15127  0.37444
N  -0.19653 -0.87426 -1.31159
C  -0.52075 -1.32051 -2.35476
C  -0.98147 -1.89904 -3.61174
H  -0.43170 -2.82803 -3.84000
H  -2.05734 -2.13422 -3.54571
H  -0.82822 -1.19282 -4.44513
H   4.49386  1.96105 -1.87019
H   3.37530  4.06258 -2.64711
H   0.88789  4.34499 -2.20276
H  -0.31674  2.54585 -0.95077
H   5.29275  0.08264 -1.17469
H   5.98840 -2.17496 -0.39536
H   4.31426 -3.69270  0.68968
H   1.96418 -2.94896  1.01876
N  -1.44291  1.09066  0.85334
C  -1.12267  2.20874  1.56646
H  -0.09198  2.24301  1.92902
C  -2.00604  3.26053  1.82203
H  -1.66584  4.12540  2.39798
C  -3.31287  3.17552  1.31855
C  -3.67392  2.02108  0.61813
H  -4.03867  3.97697  1.48567
H  -4.69585  1.88926  0.25241
C  -2.73735  0.98261  0.40966
C  -3.20226 -0.26472 -0.25813
C  -2.89891 -1.53337  0.28468
C  -3.42770 -2.69608 -0.29544
H  -3.19385 -3.67281  0.14157
C  -4.25982 -2.61372 -1.42645
H  -4.67236 -3.52368 -1.87446
C  -4.56202 -1.35602 -1.97639
H  -5.20510 -1.28059 -2.85939
C  -4.04216 -0.18980 -1.39271
H  -4.27645  0.78708 -1.82941
O  -0.13553 -1.58509  1.94128
C   0.43604 -0.85000  2.82860
H  -2.24501 -1.60005  1.15920
O   0.98630  0.24994  2.43260
C   0.49695 -1.26958  4.27622
H   1.41002 -1.86684  4.44632
H   0.53953 -0.38852  4.93368
H  -0.37166 -1.89327  4.53449

55
TS(4d-6e)1
SCF(BP86)= -1414.44528731
G(298 K) = -1414.084167
SCF(PBE) = -1413.32371509
Low Freq. = -66.5650cm-1,
23.7191cm-1
C   4.09730 -1.01711 -0.84087
C   3.07643 -0.49282 -0.01836
C   2.29506 -1.38167  0.75964
C   2.53359 -2.76489  0.70673
C   3.54499 -3.27914 -0.12260
C   4.32608 -2.40122 -0.89505
C   2.82726  0.96735  0.06419
C   3.89693  1.88737  0.05912
C   3.64805  3.25375  0.22816
C   2.32341  3.67350  0.42706
C   1.30799  2.71259  0.41135
N   1.52886  1.38743  0.20269
Ru -0.23934 -0.00773  0.13581
N   0.34597 -0.92083 -1.50002
C   0.65382 -1.43597 -2.51502
C   1.06972 -2.08242 -3.75417
H   0.94133 -1.39955 -4.61086
H   2.13274 -2.36990 -3.69347
H   0.47215 -2.99074 -3.94023
H   4.92024  1.51568 -0.04147
H   4.47275  3.97257  0.23003
H   2.07346  4.72520  0.59040
H   0.26210  2.98961  0.56979
H   4.69591 -0.34297 -1.46256
H   5.11406 -2.79462 -1.54541
H   3.72894 -4.35765 -0.16297
H   1.93351 -3.44061  1.32525
N  -1.56562  1.03167 -0.93767
C  -1.23720  2.10835 -1.70302
H  -0.17391  2.35232 -1.74335
C  -2.19110  2.86431 -2.38809
H  -1.86773  3.72450 -2.97967
C  -3.54473  2.49694 -2.29372
C  -3.88417  1.36390 -1.54739
H  -4.31887  3.07502 -2.80657
H  -4.92554  1.03706 -1.48131
C  -2.89077  0.61594 -0.88577
C  -3.07510 -0.63980 -0.16200
C  -1.85046 -1.19924  0.33260
C  -1.93772 -2.46752  0.95707
H  -1.02659 -2.94643  1.33876
C  -3.17074 -3.12583  1.11582
H  -3.20609 -4.10418  1.61048
C  -4.36467 -2.53883  0.64623
H  -5.32229 -3.05332  0.77717
C  -4.31627 -1.29614  0.00107
H  -5.24322 -0.84579 -0.37410
O  -0.90601  1.07245  1.81208
C  -0.46029  0.59734  2.94079
O   0.32265 -0.38518  3.02573
H   1.56101 -0.98432  1.48687
H  -0.22757  1.24765  4.99879
H  -1.88206  0.76042  4.55537
H  -1.25939  2.33518  3.99751
C  -0.98102  1.28940  4.19768

55
Int(4d-6e)
SCF(BP86)= -1414.45088372
```

G(298 K) = -1414.090166
SCF(PBE) = -1413.32678276
Low Freq. = 7.4186cm-1, 22.9119cm-1

C	3.99925	-1.35059	-0.67690
C	2.89982	-0.66915	-0.11406
C	1.75844	-1.40951	0.32052
C	1.78001	-2.81632	0.21665
C	2.88066	-3.48280	-0.34315
C	3.99243	-2.74833	-0.79253
C	2.87166	0.79997	0.00707
C	4.03492	1.58738	0.13062
C	3.92295	2.96894	0.32070
C	2.64098	3.53523	0.41096
C	1.52823	2.69834	0.27899
N	1.62196	1.36442	0.04952
Ru	-0.11140	-0.01839	0.01348
N	0.16336	-0.67886	-1.82811
C	0.30006	-1.03110	-2.94324
C	0.47446	-1.47080	-4.32271
H	0.96533	-0.68651	-4.92329
H	1.09749	-2.37982	-4.35762
H	-0.50304	-1.70057	-4.77850
H	5.01724	1.10825	0.10965
H	4.81870	3.58889	0.42052
H	2.49849	4.60520	0.58377
H	0.51263	3.09477	0.35857
H	4.85437	-0.78195	-1.05676
H	4.85039	-3.26191	-1.23758
H	2.87272	-4.57453	-0.42534
H	0.92648	-3.39681	0.57678
N	-1.60093	1.15774	-0.76404
C	-1.37385	2.29104	-1.47596
H	-0.32462	2.55505	-1.62444
C	-2.40490	3.08002	-1.99180
H	-2.16263	3.98667	-2.55161
C	-3.73335	2.67897	-1.76844
C	-3.97333	1.48950	-1.07459
H	-4.56854	3.27684	-2.14515
H	-4.99764	1.14184	-0.91732
C	-2.89819	0.71525	-0.59039
C	-2.99388	-0.59122	0.06249
C	-1.72866	-1.20917	0.35518
C	-1.79722	-2.48658	0.96432
H	-0.87266	-3.00555	1.24242
C	-3.02007	-3.11466	1.26245
H	-3.02098	-4.10284	1.73827
C	-4.24223	-2.48243	0.96091
H	-5.19401	-2.97038	1.19480
C	-4.22588	-1.21713	0.36164
H	-5.17552	-0.71852	0.13422
O	-0.55960	0.89468	1.85411
C	-0.02019	0.49888	2.97745
O	0.81682	-0.42771	3.10286
H	1.06553	-0.97702	1.14974
H	0.25051	1.27226	4.99139
H	-1.40385	0.72401	4.62191
H	-0.83482	2.27817	3.96140
C	-0.52520	1.25206	4.21114

55

TS(4d-6e)2
SCF(BP86)= -1414.44012989
G(298 K) = -1414.082984
SCF(PBE) = -1413.31521339
Low Freq. = -969.4680cm-1, 23.3175cm-1

C	-3.94606	-1.18563	1.07891
C	-2.83645	-0.58388	0.44555
C	-1.59869	-1.29779	0.25202
C	-1.57997	-2.63779	0.72591
C	-2.68535	-3.23590	1.35160
C	-3.87749	-2.51026	1.53064
C	-2.87748	0.80753	-0.03069
C	-4.06350	1.53090	-0.28246
C	-3.99787	2.82071	-0.81855
C	-2.73757	3.36309	-1.12172
C	-1.59978	2.59968	-0.84305
N	-1.64542	1.36506	-0.28234
Ru	0.08604	0.02192	-0.00944
N	0.05111	-0.05088	1.95017
C	0.04861	-0.06168	3.12779
C	0.04191	-0.07064	4.58634
H	-0.44334	0.83822	4.98083
H	-0.50669	-0.95045	4.96175
H	1.07316	-0.11244	4.97483
H	-5.03306	1.06639	-0.08433
H	-4.91393	3.38476	-1.01837
H	-2.63142	4.35608	-1.56643
H	-0.60091	2.97924	-1.07536
H	-4.86203	-0.60857	1.24932
H	-4.74002	-2.96810	2.02570
H	-2.61580	-4.27061	1.70699
H	-0.66654	-3.22997	0.61049
N	1.69519	1.38601	0.26738
C	1.55657	2.68629	0.62611
H	0.52941	3.05281	0.69446
C	2.64015	3.52503	0.90298
H	2.46346	4.56736	1.18025
C	3.93673	2.99066	0.81649
C	4.09035	1.64145	0.48441
H	4.81350	3.61256	1.02117
H	5.08880	1.19860	0.44215
C	2.95965	0.83739	0.22398
C	2.98199	-0.59915	-0.07756
C	1.69304	-1.23432	-0.16580
C	1.71711	-2.60804	-0.51974
H	0.77241	-3.14552	-0.65833
C	2.91268	-3.31762	-0.73272
H	2.87346	-4.38007	-1.00301
C	4.15786	-2.67257	-0.60721
H	5.09067	-3.22221	-0.77061
C	4.18722	-1.30979	-0.28745
H	5.15487	-0.79888	-0.21778
O	0.28522	0.26525	-2.11374
C	-0.36473	-0.54637	-2.85818
O	-1.14596	-1.45562	-2.37900
H	-1.10743	-1.26978	-1.12173
H	-1.21277	-0.33321	-4.82428
H	0.21380	-1.38701	-4.74730
H	0.42390	0.39402	-4.63811
C	-0.22054	-0.45060	-4.35956

55
6e
SCF(BP86)= -1414.44305416
G(298 K) = -1414.082222
SCF(PBE) = -1413.31733227
Low Freq. = 13.3398cm-1,
18.4893cm-1
C -4.03683 -1.12874 0.97380
C -2.87134 -0.51493 0.46001
C -1.59815 -1.19643 0.42052
C -1.60911 -2.51404 0.96011
C -2.76723 -3.12741 1.46684
C -3.99364 -2.43734 1.47094
C -2.88627 0.86438 -0.04953
C -4.05314 1.59388 -0.36785
C -3.95478 2.87521 -0.91838
C -2.67823 3.40691 -1.16795
C -1.56108 2.63885 -0.82585
N -1.63878 1.41084 -0.25469
Ru 0.06181 0.03535 0.06259
N 0.12289 0.08130 2.00583
C 0.17392 0.13334 3.18251
C 0.23508 0.21355 4.63788
H -0.20521 1.15946 4.99719
H -0.31923 -0.62360 5.09428
H 1.28163 0.16434 4.98271
H -5.03524 1.14166 -0.20622
H -4.85727 3.44146 -1.16818
H -2.54370 4.39372 -1.61875
H -0.55082 3.00977 -1.01832
H -4.98232 -0.57518 1.01224
H -4.89875 -2.90669 1.87026
H -2.71167 -4.14609 1.86969
H -0.67168 -3.07911 0.99944
N 1.76670 1.38298 0.16568
C 1.71449 2.70289 0.46851
H 0.71240 3.12988 0.56142
C 2.84956 3.49616 0.66484
H 2.73844 4.55774 0.90056
C 4.11183 2.89016 0.55218
C 4.18023 1.52074 0.27817
H 5.02777 3.47212 0.69363
H 5.15226 1.02382 0.22151
C 2.99798 0.76904 0.09981
C 2.94173 -0.68318 -0.12626
C 1.62951 -1.27829 -0.09445
C 1.59475 -2.67255 -0.36181
H 0.62962 -3.18991 -0.39585
C 2.75125 -3.43317 -0.61162
H 2.66236 -4.50814 -0.81203
C 4.01927 -2.82266 -0.61224
H 4.92303 -3.40945 -0.80716
C 4.10760 -1.44548 -0.37563
H 5.09189 -0.96328 -0.40390
O 0.19034 0.15812 -2.08293
C -0.39877 -0.67004 -2.82804
O -1.16566 -1.62711 -2.33917
H -1.15222 -1.47590 -1.30098
H -1.26714 -0.52514 -4.78345
H 0.16465 -1.57108 -4.68731
H 0.36722 0.21562 -4.62700
C -0.27006 -0.62508 -4.32458

55
4d’
SCF(BP86)= -1414.45717079
G(298 K) = -1414.095566
SCF(PBE) = -1413.32952164
Low Freq. = 19.3059cm-1,
22.1041cm-1
C -4.13316 1.69101 -0.33973
C -2.83737 1.19451 -0.06898
C -2.49378 -0.17953 -0.30532
C -3.52712 -1.02029 -0.79193
C -4.81734 -0.52649 -1.05400
C -5.12365 0.83245 -0.83465
C -1.75554 1.99695 0.50554
C -1.84909 3.33972 0.92188
C -0.76533 3.96698 1.54488
C 0.40853 3.22462 1.76088
C 0.46144 1.90219 1.31356
N -0.56986 1.29394 0.66836
Ru -0.56903 -0.64497 -0.00329
N -0.86736 -1.19252 1.82587
C -1.05092 -1.54100 2.93992
C -1.29458 -2.01652 4.29707
H -1.45784 -3.10791 4.30097
H -0.43617 -1.79071 4.95223
H -2.19028 -1.53125 4.72035
H -2.78630 3.88183 0.76864
H -0.83914 5.00894 1.87028
H 1.27700 3.65623 2.26504
H 1.35457 1.29045 1.44840
H -4.37476 2.74646 -0.16525
H -6.12787 1.21510 -1.04483
H -5.59270 -1.20261 -1.43504
H -3.31033 -2.07868 -0.98140
N 1.69683 -1.27206 0.23666
C 1.72185 -2.54424 0.72431
H 0.74302 -3.01347 0.84573
C 2.88486 -3.24205 1.06165
H 2.81147 -4.26039 1.45285
C 4.12149 -2.60911 0.87378
C 4.12334 -1.31230 0.35434
H 5.06149 -3.11708 1.10960
H 5.06637 -0.79595 0.15674
C 2.90882 -0.65658 0.04287
C 2.98412 0.72445 -0.51380
C 2.18736 1.12997 -1.60717
C 2.32542 2.41985 -2.14399
H 1.70860 2.71651 -2.99916
C 3.25047 3.32576 -1.59771
H 3.35219 4.33154 -2.01836
C 4.04664 2.93181 -0.50844
H 4.76621 3.63099 -0.07012
C 3.91940 1.64085 0.02491
H 4.53144 1.34795 0.88462
O -0.70446 -2.51536 -1.11200
C -0.50743 -1.90257 -2.22594
H 1.46669 0.42871 -2.03663
O -0.32527 -0.62513 -2.18182

C -0.51709 -2.63907 -3.54220
H -1.48918 -2.48559 -4.04252
H   0.26616 -2.24497 -4.20810
H -0.37072 -3.71742 -3.38541

55
TS(4d'-6c)1
SCF(BP86)= -1414.44332312
G(298 K) = -1414.081946
SCF(PBE) = -1413.32220982
Low Freq. = -85.1811cm-1,
23.0474cm-1
C -3.99778 1.42300 -0.41834
C -2.69627 1.00663 -0.05421
C -2.22121 -0.31601 -0.34823
C -3.14100 -1.20117 -0.96306
C -4.43778 -0.78972 -1.31689
C -4.86702 0.52748 -1.05468
C -1.74810 1.85189 0.68053
C -2.00855 3.14096 1.18764
C -1.04486 3.80876 1.95017
C   0.17903 3.16831 2.20778
C   0.39766 1.89853 1.66623
N -0.52083 1.25297 0.90255
Ru -0.27857 -0.64557 0.09784
N -0.72284 -1.37994 1.78195
C -0.99803 -1.84058 2.83345
C -1.33375 -2.42423 4.12702
H -1.46689 -3.51549 4.03512
H -0.53533 -2.23064 4.86333
H -2.27325 -1.99190 4.51076
H -2.97766 3.60823 0.99365
H -1.24880 4.80739 2.34793
H   0.95762 3.63848 2.81386
H   1.33550 1.36214 1.82794
H -4.33910 2.44309 -0.20575
H -5.87511 0.84818 -1.33738
H -5.12216 -1.49723 -1.80092
H -2.82848 -2.23033 -1.17610
N   1.92111 -0.89364 0.37516
C   2.33038 -2.00623 1.04450
H   1.52841 -2.66612 1.38506
C   3.67022 -2.30938 1.29859
H   3.92493 -3.21862 1.84979
C   4.66035 -1.43466 0.82301
C   4.25887 -0.29834 0.11371
H   5.72196 -1.64150 0.98696
H   5.00148 0.38650 -0.30427
C   2.88792 -0.03964 -0.09926
C   2.44410 1.16175 -0.84693
C   1.28752 1.10892 -1.66069
C   0.85104 2.25262 -2.35109
H -0.03659 2.19117 -2.98940
C   1.55847 3.46103 -2.23909
H   1.21674 4.35145 -2.77670
C   2.71437 3.51939 -1.43988
H   3.27008 4.45780 -1.34493
C   3.15460 2.37990 -0.75021
H   4.03726 2.44553 -0.10560
O -0.24384 -2.60766 -0.69833
C -0.14996 -2.57132 -1.99352
H   0.79222 0.14048 -1.86821
O -0.06936 -1.49271 -2.64620
C -0.16891 -3.91284 -2.71747
H -1.13036 -4.02850 -3.24754
H   0.62825 -3.94230 -3.47757
H -0.04774 -4.75535 -2.02076

55
Int(4d'-6c)
SCF(BP86)= -1414.45538938
G(298 K) = -1414.094789
SCF(PBE) = -1413.33315073
Low Freq. = 26.3603cm-1,
32.8998cm-1
C -4.19122 0.28967 -0.53816
C -2.83653 0.28680 -0.12998
C -1.92569 -0.74342 -0.54498
C -2.46049 -1.76509 -1.36871
C -3.80621 -1.76200 -1.77511
C -4.67709 -0.73233 -1.36282
C -2.25123 1.31811 0.73195
C -2.93508 2.41745 1.29438
C -2.25957 3.32691 2.11256
C -0.89103 3.12987 2.36655
C -0.25659 2.02944 1.78398
N -0.90540 1.14373 0.98777
Ru -0.01932 -0.53682 0.12792
N -0.46525 -1.51039 1.78631
C -0.72540 -2.07062 2.78687
C -1.03774 -2.76839 4.02743
H -0.51095 -3.73633 4.06044
H -0.72178 -2.16623 4.89534
H -2.12181 -2.95463 4.10194
H -4.00020 2.55029 1.08738
H -2.79182 4.17736 2.54923
H -0.31960 3.81110 3.00201
H   0.80550 1.82806 1.94470
H -4.87091 1.08752 -0.21598
H -5.72493 -0.73142 -1.68058
H -4.18458 -2.56831 -2.41554
H -1.80648 -2.58286 -1.69393
N   2.06821 -0.17547 0.65685
C   2.77161 -0.91589 1.55217
H   2.19819 -1.68105 2.08316
C   4.13392 -0.72543 1.79953
H   4.64435 -1.35136 2.53642
C   4.81877 0.26835 1.07871
C   4.10892 1.03214 0.14602
H   5.88818 0.43772 1.23429
H   4.61983 1.79513 -0.44725
C   2.73156 0.80335 -0.04860
C   1.89173 1.56069 -0.99174
C   0.63560 0.99000 -1.37543
C -0.20940 1.71951 -2.24523
H -1.15436 1.26944 -2.56462
C   0.15314 2.99598 -2.69707
H -0.51807 3.55195 -3.36015
C   1.38341 3.55869 -2.30640
H   1.66930 4.55576 -2.65575
C   2.24301 2.84497 -1.45891
H   3.18085 3.30673 -1.13282

O   0.61013 -2.42741 -0.59006
C   1.07971 -2.58384 -1.79739
H   0.58159 -0.18789 -1.56019
O   1.20499 -1.67390 -2.65635
C   1.48337 -4.01715 -2.14994
H   0.83107 -4.39203 -2.95715
H   2.51557 -4.02700 -2.53685
H   1.40844 -4.69303 -1.28591

55
TS(4d'-6c)2
SCF(BP86)= -1414.44979026
G(298 K) = -1414.092825
SCF(PBE) = -1413.32653632
Low Freq. = -951.8453cm-1,
28.1378cm-1
C -4.17721 0.42162 -0.51101
C -2.82333 0.36635 -0.10406
C -1.93374 -0.66765 -0.56088
C -2.49919 -1.63839 -1.42821
C -3.84331 -1.58322 -1.83462
C -4.68914 -0.54997 -1.37946
C -2.21257 1.34295 0.79998
C -2.86452 2.43814 1.40682
C -2.16545 3.29381 2.26224
C -0.80319 3.04662 2.50782
C -0.19949 1.95310 1.88180
N -0.87196 1.11652 1.05094
Ru -0.03018 -0.53598 0.13155
N -0.49349 -1.60690 1.77704
C -0.75650 -2.19347 2.76284
C -1.07513 -2.92399 3.98340
H -0.55662 -3.89699 3.98963
H -0.75518 -2.35002 4.86887
H -2.16069 -3.10377 4.05276
H -3.92484 2.61006 1.20328
H -2.67355 4.14109 2.73263
H -0.21256 3.68517 3.16963
H   0.85625 1.71561 2.03360
H -4.83627 1.22192 -0.15326
H -5.73616 -0.50866 -1.69735
H -4.24106 -2.35182 -2.50904
H -1.87240 -2.46269 -1.79029
N   2.05531 -0.24118 0.69913
C   2.73085 -0.98503 1.61310
H   2.13441 -1.73436 2.14190
C   4.09197 -0.81831 1.88188
H   4.57906 -1.44699 2.63219
C   4.80480 0.15901 1.16454
C   4.12400 0.92667 0.21432
H   5.87406 0.31294 1.33767
H   4.65972 1.67840 -0.37151
C   2.74545 0.72153 -0.00625
C   1.92639 1.47192 -0.97040
C   0.62623 0.93143 -1.27626
C -0.18133 1.68251 -2.17464
H -1.16926 1.29159 -2.44213
C   0.25256 2.89423 -2.73146
H -0.40156 3.44638 -3.41611
C   1.52800 3.40240 -2.41616
H   1.87024 4.34910 -2.84646
C   2.35677 2.69161 -1.53785
H   3.33485 3.10756 -1.27140
O   0.57000 -2.41009 -0.70088
C   0.96620 -2.41117 -1.91978
H   0.65459 -0.38800 -1.75804
O   1.01755 -1.34064 -2.63054
C   1.38639 -3.72042 -2.55312
H   0.75530 -3.92530 -3.43374
H   2.42720 -3.64487 -2.90834
H   1.30002 -4.55018 -1.83850

55
6c
SCF(BP86)= -1414.45656525
G(298 K) = -1414.095760
SCF(PBE) = -1413.33207990
Low Freq. = 21.2058cm-1,
29.1672cm-1
C -4.17091 0.47301 -0.60427
C -2.82977 0.42936 -0.15632
C -1.92161 -0.61485 -0.55794
C -2.46841 -1.61062 -1.41313
C -3.79961 -1.56771 -1.85980
C -4.65876 -0.52153 -1.46057
C -2.24722 1.42163 0.74644
C -2.91001 2.53617 1.30272
C -2.23873 3.40557 2.16701
C -0.88981 3.14883 2.47179
C -0.27185 2.03646 1.89617
N -0.91692 1.18346 1.05552
Ru -0.04685 -0.45508 0.19247
N -0.52641 -1.60011 1.84812
C -0.79311 -2.20900 2.82082
C -1.11909 -2.95910 4.02832
H -0.57455 -3.91778 4.03958
H -0.83644 -2.38501 4.92649
H -2.20051 -3.16988 4.07145
H -3.95933 2.71295 1.05085
H -2.75597 4.26876 2.59660
H -0.31823 3.79637 3.14186
H   0.77422 1.79305 2.09644
H -4.84002 1.28171 -0.28539
H -5.69587 -0.48939 -1.81055
H -4.17762 -2.35630 -2.52271
H -1.83781 -2.44854 -1.73694
N   2.01272 -0.11260 0.83086
C   2.63240 -0.71428 1.87916
H   1.99949 -1.37152 2.48313
C   3.98191 -0.52311 2.18785
H   4.42210 -1.03611 3.04732
C   4.74239 0.33034 1.36861
C   4.11839 0.95500 0.28410
H   5.80418 0.50192 1.56993
H   4.69436 1.61340 -0.37172
C   2.74865 0.73195 0.02491
C   1.98100 1.33898 -1.07384
C   0.62744 0.86298 -1.24771
C -0.11914 1.49778 -2.28266
H -1.15614 1.18247 -2.44861
C   0.42144 2.50441 -3.10060
H -0.19382 2.95808 -3.88715

C   1.74858 2.93514 -2.91266
H   2.17473 3.72076 -3.54526
C   2.51984 2.35384 -1.89760
H   3.54448 2.71007 -1.73991
O   0.63256 -2.33752 -0.62103
C   0.98417 -2.53499 -1.81091
H   0.71245 -0.71797 -2.20464
O   0.99149 -1.56212 -2.71454
C   1.41795 -3.88471 -2.30927
H   0.77506 -4.19825 -3.14837
H   2.45063 -3.82866 -2.69148
H   1.36098 -4.62405 -1.50032

55
4e'
SCF(BP86)= -1414.45558112
G(298 K) = -1414.093746
SCF(PBE) = -1413.32908596
Low Freq. = 22.5067cm-1,
32.3251cm-1
C   3.92952 -1.14196 -0.32280
C   2.81406 -0.58974 -0.99354
C   2.89187 0.73767 -1.46565
C   4.05319 1.49817 -1.25847
C   5.15072 0.94783 -0.57459
C   5.08519 -0.37594 -0.10595
C   1.65531 -1.45711 -1.34985
C   1.93379 -2.63835 -2.06743
C   0.90707 -3.45294 -2.55622
C -0.41066 -3.03052 -2.33224
C -0.63657 -1.86935 -1.59411
N   0.35572 -1.08873 -1.05271
Ru -0.23234 0.38849 0.31309
N   1.25337 -0.17143 1.68674
C   2.03574 -0.40689 2.53493
C   3.02166 -0.70823 3.56547
H   2.72932 -1.60836 4.13126
H   4.00778 -0.88394 3.10405
H   3.10845 0.13590 4.26936
H   2.98150 -2.88143 -2.26440
H   1.12959 -4.36565 -3.11599
H -1.26894 -3.58975 -2.71508
H -1.65429 -1.52975 -1.40320
H   3.88274 -2.17556 0.03670
H   5.93724 -0.81624 0.42253
H   6.05520 1.54395 -0.41512
H   4.10270 2.52328 -1.64023
N -1.65202 -0.86420 1.04135
C -1.43878 -1.83488 1.97662
H -0.39684 -1.97844 2.27005
C -2.46957 -2.58807 2.54015
H -2.23068 -3.34765 3.28935
C -3.79307 -2.35052 2.12605
C -4.02406 -1.37554 1.15069
H -4.62539 -2.91850 2.55177
H -5.04120 -1.17530 0.80243
C -2.95498 -0.64032 0.60190
C -3.05097 0.37661 -0.44779
C -1.79416 0.97714 -0.80948
C -1.83040 1.93692 -1.85420
H -0.89768 2.42685 -2.16033
C -3.02963 2.28523 -2.49917
H -3.02047 3.02955 -3.30509
C -4.24960 1.68787 -2.11669
H -5.18276 1.96453 -2.61848
C -4.25907 0.73336 -1.09215
H -5.20815 0.26783 -0.80022
O -0.60610 2.08664 1.59876
C   0.21413 2.82724 0.92939
H   2.04366 1.16749 -2.00373
O   0.83060 2.29700 -0.06397
C   0.40680 4.28035 1.29179
H   1.41145 4.62146 1.00113
H -0.33397 4.89499 0.75082
H   0.25308 4.43420 2.37043

55
TS(4e'-6b)1
SCF(BP86)= -1414.44613234
G(298 K) = -1414.084827
SCF(PBE) = -1413.32127318
Low Freq. = -92.2263cm-1,
18.6459cm-1
C -4.17077 1.06652 -0.37585
C -2.83153 0.82773 -0.76178
C -2.47775 -0.45888 -1.22243
C -3.43335 -1.48418 -1.29471
C -4.76247 -1.23439 -0.91465
C -5.12750 0.04372 -0.45442
C -1.81595 1.90307 -0.71289
C -2.18127 3.23229 -1.00704
C -1.22856 4.25526 -1.02052
C   0.10498 3.90775 -0.75733
C   0.41745 2.57834 -0.47470
N -0.50657 1.56812 -0.41887
Ru 0.13774 -0.34261 0.15183
N -1.27475 -0.46095 1.72499
C -2.05079 -0.58316 2.60094
C -3.01833 -0.72680 3.68178
H -2.63515 -0.26224 4.60522
H -3.96855 -0.23953 3.40832
H -3.21355 -1.79377 3.87866
H -3.22414 3.44248 -1.25808
H -1.51414 5.28496 -1.25275
H   0.90749 4.65018 -0.77428
H   1.44815 2.28421 -0.27615
H -4.45849 2.04856 0.01346
H -6.15878 0.24305 -0.14563
H -5.51053 -2.03177 -0.97244
H -3.12496 -2.47372 -1.64230
N   1.63719 0.36283 1.25588
C   1.51290 0.74785 2.55955
H   0.49768 0.72699 2.95881
C   2.60397 1.13213 3.33960
H   2.43823 1.42687 4.37913
C   3.88933 1.12965 2.76808
C   4.02504 0.75389 1.42831
H   4.76543 1.41948 3.35518
H   5.01007 0.74894 0.95357
C   2.90088 0.37728 0.66790
C   2.88645 0.01020 -0.74671
C   1.58365 -0.32935 -1.24777

C 1.49875 -0.63521 -2.63013
H 0.52670 -0.89967 -3.06535
C 2.63241 -0.62340 -3.46071
H 2.53053 -0.86634 -4.52544
C 3.90372 -0.30510 -2.93657
H 4.78493 -0.30255 -3.58651
C 4.02999 0.01540 -1.57989
H 5.01675 0.27179 -1.17607
O 0.78218 -2.27038 0.74474
C 0.22665 -3.13677 -0.05255
H -1.45623 -0.65875 -1.58499
O -0.56609 -2.79878 -0.97186
C 0.61465 -4.59593 0.15847
H -0.23248 -5.25603 -0.08301
H 1.44557 -4.85376 -0.52160
H 0.94975 -4.77869 1.19074

55
Int(4e'-6b)
SCF(BP86)= -1414.45589404
G(298 K) = -1414.095233
SCF(PBE) = -1413.33240406
Low Freq. = 24.6730cm-1,
27.5575cm-1
C -4.17828 0.83767 -0.53929
C -2.76607 0.81304 -0.53838
C -2.09732 -0.40053 -0.87884
C -2.84968 -1.53671 -1.24302
C -4.25179 -1.49107 -1.24202
C -4.91608 -0.30116 -0.89154
C -1.96129 1.96701 -0.11161
C -2.44413 3.29014 -0.08150
C -1.61976 4.33593 0.34392
C -0.30145 4.03334 0.72576
C 0.13481 2.70959 0.66701
N -0.66398 1.67742 0.26752
Ru -0.00247 -0.26646 0.11356
N -1.16268 -0.92566 1.78920
C -1.82779 -1.30528 2.67966
C -2.65041 -1.77456 3.78751
H -2.01371 -2.04548 4.64534
H -3.35162 -0.98428 4.10135
H -3.22920 -2.66107 3.48123
H -3.46600 3.49238 -0.41237
H -1.99130 5.36414 0.36538
H 0.39144 4.81071 1.05847
H 1.15390 2.43465 0.94491
H -4.70837 1.74351 -0.22828
H -6.00989 -0.26136 -0.88387
H -4.82759 -2.37994 -1.52038
H -2.30398 -2.43452 -1.54705
N 1.69894 -0.03522 1.23972
C 1.76695 -0.20692 2.58762
H 0.81777 -0.41451 3.08452
C 2.96365 -0.13002 3.30230
H 2.95268 -0.27563 4.38546
C 4.15559 0.12774 2.60243
C 4.09584 0.31057 1.21845
H 5.11316 0.18648 3.12810
H 5.00717 0.51479 0.65039
C 2.86310 0.23463 0.53794
C 2.65218 0.45479 -0.89258
C 1.29307 0.30961 -1.33317
C 1.04142 0.60644 -2.69769
H 0.01939 0.52889 -3.08981
C 2.06679 0.98677 -3.57882
H 1.83036 1.20201 -4.62780
C 3.39853 1.09010 -3.12600
H 4.19818 1.38051 -3.81513
C 3.68739 0.82946 -1.78234
H 4.71949 0.92727 -1.42605
O 0.65669 -2.27748 -0.01743
C 0.48981 -3.02714 -1.07637
H -1.01806 -0.42938 -1.33924
O -0.10945 -2.70624 -2.12774
C 1.14780 -4.40923 -0.95216
H 0.65206 -5.13158 -1.61813
H 2.20715 -4.33669 -1.25632
H 1.12456 -4.77808 0.08522

53
7a
SCF(BP86)= -1318.12843918
G(298 K) = -1317.781488
SCF(PBE) = -1317.04958142
Low Freq. = 3.0125cm-1, 15.5800cm-
1
C 2.74272 -2.32457 1.81459
C 2.09848 -1.34021 1.02809
C 0.71912 -0.98979 1.23441
C 0.04654 -1.69122 2.26899
C 0.68554 -2.66966 3.04996
C 2.03983 -2.99124 2.82569
C 2.77013 -0.60683 -0.04894
C 4.11206 -0.77455 -0.44966
C 4.64217 -0.01656 -1.49812
C 3.80971 0.91702 -2.14104
C 2.48880 1.04913 -1.70752
N 1.95884 0.31561 -0.69056
Ru -0.00002 0.46146 -0.00030
N -0.56554 1.95766 -1.36105
N 0.56523 1.95903 1.35909
C 0.90348 2.79149 2.12081
C 1.31584 3.82157 3.06768
H 1.40848 3.39630 4.08077
H 2.29044 4.24606 2.77484
H 0.57284 4.63582 3.09543
H 4.73754 -1.50695 0.06810
H 5.68298 -0.14934 -1.80843
H 4.17141 1.53736 -2.96556
H 1.80498 1.76154 -2.17525
H 3.79619 -2.57556 1.64126
H 2.53886 -3.75332 3.43361
H 0.12749 -3.18812 3.83976
H -1.00846 -1.46638 2.46941
C -0.90399 2.78954 -2.12332
C -1.31702 3.81896 -3.07062
H -1.38518 3.39908 -4.08788
H -2.30340 4.22537 -2.79193
H -0.58726 4.64550 -3.08022
N -1.95886 0.31599 0.69011
C -2.48898 1.05052 1.70625

H -1.80530 1.76356 2.17323
C -3.80989 0.91866 2.13987
H -4.17173 1.53984 2.96369
C -4.64215 -0.01579 1.49795
C -4.11187 -0.77484 0.45034
H -5.68294 -0.14842 1.80838
H -4.73721 -1.50791 -0.06663
C -2.76996 -0.60730 0.04947
C -2.09816 -1.34169 -1.02678
C -0.71888 -0.99118 -1.23351
C -0.04619 -1.69358 -2.26737
H   1.00876 -1.46874 -2.46806
C -0.68501 -2.67297 -3.04730
H -0.12687 -3.19214 -3.83656
C -2.03923 -2.99459 -2.82265
H -2.53811 -3.75742 -3.42975
C -2.74221 -2.32702 -1.81221
H -3.79563 -2.57804 -1.63859

53
7b
SCF(BP86)= -1318.12660843
G(298 K) = -1317.779721
SCF(PBE) = -1317.04804746
Low Freq. = 23.9495cm-1,
25.6285cm-1
C -4.13845 -0.85951 0.12522
C -2.78322 -0.56273 -0.15609
C -1.94328 0.13853 0.78281
C -2.57052 0.51765 2.00232
C -3.91454 0.22292 2.28152
C -4.70843 -0.47053 1.34271
C -2.12569 -0.94095 -1.40728
C -2.72309 -1.63322 -2.48324
C -1.98503 -1.93628 -3.63029
C -0.63723 -1.53905 -3.69363
C -0.08981 -0.85773 -2.60412
N -0.79787 -0.55677 -1.48382
Ru -0.03454 0.45782 0.17162
N   0.50656 1.38307 1.84254
N -0.54698 2.28880 -0.70106
C -0.81769 3.31415 -1.21395
C -1.15077 4.58270 -1.85237
H -0.35255 5.32235 -1.67490
H -1.26464 4.44443 -2.94035
H -2.09592 4.98207 -1.44860
H -3.77231 -1.93219 -2.41133
H -2.45057 -2.47371 -4.46211
H -0.01530 -1.75137 -4.56717
H   0.95227 -0.52902 -2.60276
H -4.75468 -1.39624 -0.60650
H -5.75661 -0.70050 1.56100
H -4.35366 0.53451 3.23788
H -1.98493 1.05716 2.75737
C   0.83255 1.93440 2.83215
C   1.21681 2.61557 4.06352
H   1.92552 3.43377 3.85155
H   0.33039 3.04233 4.56267
H   1.70025 1.90867 4.75852
N   2.03346 0.51526 -0.54326
C   2.60817 1.50985 -1.26568

H   1.95038 2.34810 -1.51550
C   3.94448 1.48861 -1.67557
H   4.35045 2.32059 -2.25733
C   4.73398 0.38130 -1.31837
C   4.15494 -0.64843 -0.57023
H   5.78511 0.32303 -1.61732
H   4.75538 -1.51510 -0.28118
C   2.79812 -0.57456 -0.18428
C   2.07799 -1.59085 0.60032
C   0.69851 -1.30853 0.90254
C   0.01650 -2.29447 1.66307
H -1.03551 -2.12451 1.92111
C   0.63799 -3.47762 2.09807
H   0.06923 -4.20995 2.68471
C   1.98860 -3.72850 1.78548
H   2.47758 -4.64915 2.12125
C   2.70249 -2.78418 1.03865
H   3.75330 -2.98309 0.79727

53
7c
SCF(BP86)= -1318.11342746
G(298 K) = -1317.766330
SCF(PBE) = -1317.03575979
Low Freq. = 7.6943cm-1, 10.5876cm-
1
C -4.13299 -0.86979 -0.24077
C -2.77504 -0.61069 0.06758
C -2.00136 0.36760 -0.64665
C -2.69136 1.05877 -1.67744
C -4.03969 0.80876 -1.98840
C -4.76732 -0.16132 -1.26866
C -2.05561 -1.32187 1.13292
C -2.61075 -2.31774 1.96677
C -1.84001 -2.92865 2.95876
C -0.50033 -2.53318 3.11386
C   0.00746 -1.54747 2.26433
N -0.73211 -0.94823 1.29316
Ru 0.00001 0.53230 -0.00104
N   0.46905 1.92077 -1.33162
N -0.46850 1.92672 1.32351
C -0.72149 2.75237 2.12559
C -1.03106 3.77326 3.12052
H -2.06418 4.14026 2.99898
H -0.92427 3.36214 4.13845
H -0.34252 4.62892 3.01903
H -3.65570 -2.60798 1.83066
H -2.27599 -3.69922 3.60199
H   0.14638 -2.97563 3.87609
H   1.04101 -1.20064 2.33754
H -4.70393 -1.62318 0.31570
H -5.81738 -0.36042 -1.50806
H -4.53170 1.36945 -2.79365
H -2.15482 1.82079 -2.26029
C   0.72241 2.74268 -2.13741
C   1.03255 3.75889 -3.13695
H   0.90552 3.34946 -4.15325
H   0.35782 4.62429 -3.02583
H   2.07239 4.11114 -3.02997
N   0.73173 -0.95390 -1.28890
C -0.00803 -1.55712 -2.25745

H -1.04159 -1.21058 -2.33186
C 0.49959 -2.54631 -3.10303
H -0.14727 -2.99188 -3.86330
C 1.83931 -2.94111 -2.94660
C 2.61024 -2.32615 -1.95727
H 2.27518 -3.71427 -3.58680
H 3.65523 -2.61578 -1.82021
C 2.05525 -1.32692 -1.12736
C 2.77487 -0.61144 -0.06502
C 2.00137 0.36991 0.64520
C 2.69153 1.06522 1.67309
H 2.15516 1.82976 2.25278
C 4.03982 0.81626 1.98505
H 4.53196 1.38019 2.78796
C 4.76726 -0.15693 1.26931
H 5.81728 -0.35522 1.50951
C 4.13276 -0.86954 0.24439
H 4.70354 -1.62536 -0.30895

53
7d
SCF(BP86)= -1318.11015721
G(298 K) = -1317.763112
SCF(PBE) = -1317.03102175
Low Freq. = 22.0674cm-1,
23.8153cm-1
C 4.04509 -1.55277 -0.22745
C 2.87746 -0.75394 -0.15900
C 1.56096 -1.31758 -0.30738
C 1.52596 -2.69283 -0.65744
C 2.68472 -3.48420 -0.74870
C 3.95257 -2.92189 -0.50456
C 2.94807 0.71379 -0.07294
C 4.13776 1.46209 0.08256
C 4.11091 2.85637 -0.01152
C 2.88828 3.49429 -0.28142
C 1.74008 2.70139 -0.38432
N 1.74082 1.35493 -0.24814
Ru 0.00000 -0.02132 0.00000
N 0.40220 -0.01397 1.94115
N -0.40216 -0.01409 -1.94115
C -0.65597 0.00145 -3.09093
C -0.95977 0.02885 -4.51693
H -1.33506 1.02251 -4.81427
H -0.05410 -0.19312 -5.10574
H -1.72814 -0.72309 -4.76264
H 5.08383 0.94634 0.26744
H 5.03139 3.43631 0.10842
H 2.81676 4.57848 -0.40130
H 0.76775 3.15634 -0.59085
H 5.03619 -1.10414 -0.09101
H 4.85712 -3.53698 -0.55846
H 2.59994 -4.54702 -1.00787
H 0.55845 -3.16877 -0.85154
C 0.65598 0.00166 3.09093
C 0.95962 0.02947 4.51695
H 0.05392 -0.19243 5.10573
H 1.72803 -0.72235 4.76293
H 1.33480 1.02323 4.81407
N -1.74079 1.35497 0.24804
C -1.74003 2.70143 0.38412

H -0.76769 3.15639 0.59061
C -2.88822 3.49435 0.28115
H -2.81668 4.57854 0.40095
C -4.11086 2.85642 0.01131
C -4.13773 1.46214 -0.08266
H -5.03133 3.43637 -0.10867
H -5.08382 0.94639 -0.26748
C -2.94806 0.71382 0.07290
C -2.87746 -0.75390 0.15906
C -1.56097 -1.31754 0.30747
C -1.52598 -2.69277 0.65762
H -0.55847 -3.16871 0.85175
C -2.68475 -3.48412 0.74894
H -2.59998 -4.54693 1.00818
C -3.95260 -2.92181 0.50476
H -4.85715 -3.53689 0.55871
C -4.04509 -1.55271 0.22756
H -5.03619 -1.10407 0.09110

53
7e
SCF(BP86)= -1318.09821282
G(298 K) = -1317.750665
SCF(PBE) = -1317.01949781
Low Freq. = 20.0769cm-1,
23.2240cm-1
C -4.10779 1.36674 -0.61386
C -2.91641 0.68964 -0.25629
C -1.64599 1.35666 -0.15406
C -1.67998 2.74046 -0.46696
C -2.85908 3.42748 -0.81207
C -4.08491 2.74025 -0.88379
C -2.90674 -0.74920 0.04499
C -4.05785 -1.55314 0.19802
C -3.94701 -2.88756 0.59748
C -2.66857 -3.40544 0.86391
C -1.56041 -2.57650 0.67205
N -1.65111 -1.29214 0.23813
Ru 0.00002 0.00068 0.09071
N -0.00239 0.00081 -1.89969
N 0.00262 -0.00043 2.07126
C 0.00426 -0.00176 3.24929
C 0.00814 -0.00657 4.70752
H -1.02431 0.02009 5.09434
H 0.50492 -0.91155 5.09658
H 0.54416 0.87785 5.09061
H -5.04457 -1.11426 0.02941
H -4.84152 -3.50582 0.72017
H -2.52238 -4.43216 1.20945
H -0.54739 -2.93142 0.86609
H -5.05745 0.82483 -0.69919
H -5.00563 3.26513 -1.16005
H -2.82085 4.50091 -1.03706
H -0.74916 3.32257 -0.46880
C -0.00413 0.00063 -3.07561
C -0.00724 -0.00125 -4.53381
H -0.53337 -0.89211 -4.91573
H -0.51448 0.89808 -4.92079
H 1.02582 -0.01477 -4.91897
N 1.65196 1.29319 0.23561
C 1.56228 2.57772 0.66925

```
H   0.54958  2.93335  0.86362
C   2.67103  3.40590  0.86091
H   2.52558  4.43277  1.20635
C   3.94909  2.88706  0.59453
C   4.05891  1.55258  0.19506
H   4.84406  3.50466  0.71720
H   5.04530  1.11296  0.02634
C   2.90720  0.74946  0.04214
C   2.91587 -0.68921 -0.25992
C   1.64507 -1.35542 -0.15767
C   1.67767 -2.73882 -0.47249
H   0.74633 -3.32010 -0.47442
C   2.85607 -3.42634 -0.81897
H   2.81689 -4.49946 -1.04529
C   4.08245 -2.74003 -0.89027
H   5.00264 -3.26534 -1.16750
C   4.10656 -1.36683 -0.61880
H   5.05659 -0.82557 -0.70402
```

```
54
8a
SCF(BP86)= -1413.98500641
G(298 K) = -1413.636703
SCF(PBE) = -1412.86738794
Low Freq. = 17.3619cm-1,
29.2278cm-1
C   3.07385 -2.62584  0.97172
C   2.31900 -1.50555  0.54972
C   0.90809 -1.38589  0.83356
C   0.33259 -2.47282  1.55156
C   1.08343 -3.58427  1.96680
C   2.46318 -3.66736  1.68083
C   2.89272 -0.39276 -0.20967
N   1.97471  0.59813 -0.52040
C   2.39355  1.67804 -1.23598
C   3.71098  1.83654 -1.67311
C   4.65508  0.83975 -1.36197
C   4.23538 -0.27394 -0.62721
Ru  0.03123  0.30001  0.16315
O  -0.44602  2.31546 -0.77210
C  -1.48173  3.09459 -0.69431
C  -1.33681  4.39701 -1.51455
H   4.14562 -2.68971  0.74518
C  -0.59275 -0.64533 -1.52008
C  -1.93987 -1.15362 -1.47379
C  -2.53621 -1.79095 -2.58735
C  -1.81579 -1.95616 -3.77762
C  -0.48868 -1.48347 -3.84993
C   0.10126 -0.84642 -2.74482
H  -3.56794 -2.16039 -2.53135
C  -2.61771 -0.97028 -0.18825
C  -3.90754 -1.43396  0.13858
C  -4.43973 -1.23075  1.41670
C  -3.64906 -0.55441  2.36522
C  -2.38149 -0.10537  1.99341
N  -1.85095 -0.28471  0.74822
O  -2.53630  2.89950 -0.04039
H  -4.48987 -1.96552 -0.62000
H  -5.44046 -1.59368  1.66990
H  -4.00638 -0.37057  3.38276
H  -1.74121  0.43123  2.69760
```

```
H  -2.27926 -2.44877 -4.63937
H   0.08474 -1.61377 -4.77686
H   1.13503 -0.48858 -2.83556
H   1.60352  2.40995 -1.43491
H   3.98723  2.72746 -2.24466
H   5.69659  0.93007 -1.68622
H   4.94810 -1.06374 -0.37248
H  -0.73752 -2.44419  1.79211
H   0.59394 -4.39697  2.51917
H   3.04920 -4.53376  2.00582
H  -2.25903  4.99687 -1.47443
H  -1.09932  4.16548 -2.56696
H  -0.50044  5.00205 -1.12251
N   0.45184  1.32961  1.92499
C   0.70230  1.92448  2.91314
C   0.99901  2.66120  4.13741
H   0.81347  3.73901  3.99361
H   0.36548  2.30622  4.96778
H   2.05551  2.52517  4.42308
```

```
54
8b
SCF(BP86)= -1413.98320744
G(298 K) = -1413.635323
SCF(PBE) = -1412.86580032
Low Freq. = 10.8011cm-1,
12.4711cm-1
C  -2.80759 -2.89692 -0.88054
C  -2.15338 -1.69439 -0.51626
C  -0.75160 -1.47949 -0.79777
C  -0.08771 -2.55566 -1.45271
C  -0.74046 -3.74601 -1.81128
C  -2.11039 -3.92398 -1.52691
C  -2.85461 -0.60503  0.17958
N  -2.04863  0.46472  0.50565
C  -2.58853  1.52523  1.15657
C  -3.93747  1.59232  1.51996
C  -4.77343  0.50913  1.19100
C  -4.22633 -0.58919  0.51909
Ru  0.02096  0.29016 -0.18756
O   0.35084  2.24625  0.87647
C   0.91613  3.37386  0.56429
C   0.75065  4.46259  1.64947
H  -3.87151 -3.03814 -0.65397
N   0.77244 -0.58090  1.54084
C   2.09829 -0.98991  1.50168
C   2.69160 -1.57100  2.64484
C   1.95621 -1.74093  3.82077
C   0.61198 -1.32153  3.84585
C   0.06838 -0.75258  2.69209
H   3.73803 -1.88669  2.60199
C   2.74429 -0.75618  0.21474
C   4.08403 -1.12531 -0.05976
C   4.64017 -0.88813 -1.32094
C   3.84255 -0.27451 -2.31606
C   2.51757  0.09223 -2.04689
C   1.89752 -0.12435 -0.77644
O   1.54044  3.65684 -0.48762
H   4.69759 -1.60386  0.71442
H   5.67594 -1.17401 -1.53364
H   4.27018 -0.08344 -3.30929
```

```
H   1.93280  0.56832 -2.84463
H   2.41954 -2.19163  4.70406
H  -0.00819 -1.43174  4.73966
H  -0.96912 -0.41002  2.65832
H  -1.87159  2.32458  1.37708
H  -4.32039  2.47311  2.04344
H  -5.83632  0.52211  1.45276
H  -4.86300 -1.43751  0.25240
H   0.97713 -2.44402 -1.68889
H  -0.18163 -4.54369 -2.31771
H  -2.62352 -4.85109 -1.80429
H   1.35231  5.35341  1.41217
H   1.04342  4.07655  2.64044
H  -0.31002  4.76233  1.72209
N  -0.49148  1.13676 -1.89018
C  -0.81255  1.63353 -2.91303
C  -1.16652  2.27124 -4.17697
H  -1.01249  3.36263 -4.11726
H  -0.54550  1.88012 -5.00144
H  -2.22535  2.08696 -4.42626

54
8c
SCF(BP86)= -1413.97565373
G(298 K) = -1413.627661
SCF(PBE) = -1412.85771070
Low Freq. = 20.2616cm-1,
24.1369cm-1
C  -3.00708 -2.57451 -1.21725
C  -2.32364 -1.45894 -0.68909
N  -0.96138 -1.29454 -0.92362
C  -0.31678 -2.24698 -1.65696
C  -0.95291 -3.36757 -2.19485
C  -2.33236 -3.53718 -1.97444
C  -2.91055 -0.40362  0.13490
C  -1.97440  0.60651  0.56446
C  -2.51794  1.63349  1.38892
C  -3.87159  1.66568  1.75763
C  -4.76317  0.66156  1.31612
C  -4.27702 -0.37102  0.50543
Ru -0.07770  0.37700 -0.10704
O   0.44319  2.17623  0.95363
C   1.48289  2.95597  0.86393
C   1.45010  4.10896  1.89181
H  -4.07820 -2.68184 -1.02235
C   0.56567 -0.69547  1.50250
C   1.90252 -1.23049  1.41448
C   2.48105 -1.96586  2.47774
C   1.76200 -2.20204  3.65589
C   0.45032 -1.69776  3.77108
C  -0.12368 -0.96697  2.71726
H   3.50331 -2.35490  2.39254
C   2.61868 -0.96550  0.15814
C   3.90198 -1.45235 -0.17146
C   4.47617 -1.15879 -1.41331
C   3.74335 -0.37028 -2.32077
C   2.48225  0.09209 -1.93817
N   1.91136 -0.18006 -0.73361
O   2.45341  2.86286  0.07634
H   4.44605 -2.07080  0.54844
H   5.46969 -1.53887 -1.67092
```

```
H   4.14100 -0.11148 -3.30662
H   1.88241  0.71773 -2.60612
H   2.21622 -2.76992  4.47532
H  -0.12305 -1.87638  4.69006
H  -1.14439 -0.58239  2.83545
H  -1.84116  2.42182  1.74166
H  -4.24643  2.47912  2.39333
H  -5.82017  0.68894  1.60285
H  -4.96733 -1.15247  0.16248
H   0.75158 -2.07572 -1.80648
H  -0.37061 -4.08925 -2.77486
H  -2.86655 -4.40150 -2.38081
H   2.27462  4.81607  1.71337
H   1.54333  3.70605  2.91555
H   0.48939  4.64895  1.84490
N  -0.46376  1.49034 -1.81816
C  -0.65722  2.09990 -2.81090
C  -0.88577  2.85138 -4.04099
H  -0.13233  3.64996 -4.14812
H  -0.81575  2.18830 -4.91996
H  -1.88607  3.31621 -4.03464

48
9a
SCF(BP86)= -1281.22621509
G(298 K) = -1280.918652
SCF(PBE) = -1280.21343995
Low Freq. = 29.5178cm-1,
31.7248cm-1
C  -2.57765 -2.15631 -2.15737
C  -2.00850 -1.21687 -1.26585
C  -0.58896 -0.93444 -1.26265
C   0.19369 -1.69034 -2.18479
C  -0.37672 -2.62378 -3.06354
C  -1.76986 -2.85981 -3.05947
C  -2.78249 -0.45635 -0.28572
N  -1.99978  0.41843  0.46250
C  -2.61347  1.18335  1.41262
C  -3.98371  1.12424  1.67506
C  -4.78416  0.24179  0.92375
C  -4.17050 -0.54395 -0.05922
Ru -0.00257  0.49176 -0.00358
O   0.25709  2.54220  1.05934
C  -0.03437  3.16888 -0.02319
C  -0.10530  4.68905 -0.01576
H  -3.65919 -2.34257 -2.14844
C   0.61625 -0.89274  1.28528
C   2.03992 -1.15431  1.28196
C   2.63062 -2.06514  2.18895
C   1.84150 -2.75844  3.11519
C   0.44559 -2.54006  3.12910
C  -0.14632 -1.63529  2.23463
H   3.71455 -2.23656  2.17357
C   2.79452 -0.40115  0.28127
C   4.18194 -0.47051  0.04535
C   4.77542  0.30888 -0.95501
C   3.95527  1.16605 -1.71429
C   2.58612  1.20722 -1.44278
N   1.99231  0.44787 -0.47575
O  -0.29900  2.52236 -1.10081
H   4.79459 -1.14268  0.65389
```

H 5.85285 0.25281 -1.13796
H 4.36710 1.80010 -2.50483
H 1.90557 1.86773 -1.98819
H 2.30177 -3.46301 3.81616
H -0.18115 -3.08546 3.84663
H -1.23355 -1.49083 2.26992
H -1.94826 1.86428 1.95194
H -4.41160 1.76405 2.45230
H -5.86193 0.17120 1.09956
H -4.76750 -1.23591 -0.66108
H 1.27916 -1.53194 -2.21258
H 0.26474 -3.17771 -3.76126
H -2.21340 -3.58627 -3.74877
H 0.06570 5.09637 -1.02355
H 0.62800 5.11101 0.68878
H -1.10981 5.00753 0.31513

48
9b
SCF(BP86)= -1281.21535463
G(298 K) = -1280.908303
SCF(PBE) = -1280.20167946
Low Freq. = 24.6953cm-1,
29.4475cm-1
C -2.66375 -2.21176 -2.04149
C -2.10703 -1.22543 -1.20273
N -0.72964 -0.98997 -1.21149
C 0.05039 -1.77069 -2.01982
C -0.46295 -2.75986 -2.85917
C -1.85340 -2.98494 -2.88023
C -2.83107 -0.37147 -0.26486
C -1.98238 0.53585 0.47681
C -2.65877 1.36855 1.41864
C -4.04758 1.31332 1.61360
C -4.84540 0.41529 0.86774
C -4.23168 -0.42355 -0.07140
Ru -0.03900 0.50343 -0.03098
O 0.38466 2.42293 0.93407
C 0.11478 3.11902 -0.11794
C 0.17483 4.63431 -0.04746
H -3.74636 -2.37063 -2.02466
C 0.60190 -0.81969 1.31257
C 2.02237 -1.10974 1.29999
C 2.60285 -2.00016 2.23568
C 1.81516 -2.64404 3.19675
C 0.42447 -2.39872 3.21970
C -0.15917 -1.51560 2.29930
H 3.68197 -2.19713 2.21270
C 2.80768 -0.42503 0.26741
C 4.20102 -0.53551 0.07147
C 4.82975 0.17447 -0.95842
C 4.04711 1.00140 -1.78674
C 2.67233 1.07902 -1.54529
N 2.04589 0.38929 -0.55387
O -0.23472 2.52669 -1.19872
H 4.79193 -1.17732 0.73173
H 5.91024 0.08878 -1.11072
H 4.49080 1.58015 -2.60207
H 2.01756 1.72026 -2.14472
H 2.27261 -3.33176 3.91610
H -0.20423 -2.90397 3.96437

H -1.24117 -1.34280 2.33942
H -2.06928 2.08532 2.00626
H -4.52341 1.97490 2.34974
H -5.92956 0.37567 1.01980
H -4.84999 -1.11940 -0.65359
H 1.12120 -1.55791 -1.97277
H 0.22176 -3.34132 -3.48339
H -2.29123 -3.74891 -3.52977
H 0.46772 5.05636 -1.02094
H 0.87817 4.96571 0.73138
H -0.82490 5.03081 0.20413

59
10a
SCF(BP86)= -1430.41165615
G(298 K) = -1430.025172
SCF(PBE) = -3989.60481186
Low Freq. = 11.2581cm-1,
14.9705cm-1
C 4.18993 1.70787 1.41640
C 2.92124 1.46129 0.84075
C 2.21688 0.23191 1.08437
C 2.86262 -0.71869 1.91689
C 4.12266 -0.46746 2.48530
C 4.79291 0.74857 2.23858
C 2.22810 2.41060 -0.03123
C 2.70985 3.67663 -0.42262
C 1.95124 4.49576 -1.26429
C 0.69951 4.03059 -1.70522
C 0.26559 2.77022 -1.28782
N 0.99490 1.95873 -0.47411
Ru 0.42867 0.07031 0.20594
Br -1.81969 -0.20064 -1.41115
H 3.68532 4.01385 -0.06139
H 2.32584 5.47742 -1.56941
H 0.06276 4.62963 -2.36178
H -0.69981 2.36923 -1.60713
H 5.77492 0.94191 2.68253
H 4.58962 -1.22555 3.12612
H 2.36869 -1.67439 2.12562
N 0.08529 -1.88797 0.80395
C -0.54559 -2.25794 1.95345
H -0.86736 -1.43440 2.59544
C -0.77429 -3.58894 2.30938
H -1.28643 -3.81523 3.24855
C -0.33596 -4.60640 1.44326
C 0.31470 -4.24194 0.25979
H -0.49730 -5.66081 1.68652
H 0.66756 -5.01248 -0.43123
C 0.52565 -2.88421 -0.05494
C 1.20289 -2.37665 -1.25375
C 1.29885 -0.94358 -1.33678
C 1.96149 -0.42698 -2.48140
H 2.06915 0.65924 -2.59200
C 2.48595 -1.26180 -3.48374
H 2.98958 -0.82173 -4.35340
C 2.36984 -2.66272 -3.37865
H 2.77627 -3.31426 -4.15936
C 1.72865 -3.21668 -2.26268
C -3.56040 0.25411 -0.66483
C -4.22083 1.40071 -1.12946

```
C  -4.11130 -0.57798  0.32078
C  -5.47881  1.71602 -0.58483
C  -5.36906 -0.24362  0.85368
C  -6.05226  0.89864  0.40344
H  -3.77004  2.03444 -1.89800
H  -3.57389 -1.46542  0.66569
H  -6.00690  2.60661 -0.94000
H  -5.81166 -0.88495  1.62247
H   1.64088 -4.30690 -2.18351
H  -7.03130  1.15111  0.82183
H   4.71178  2.65279  1.22337
N  -0.59903  0.93601  1.79247
C  -1.18967  1.45251  2.67269
C  -1.92630  2.08698  3.75940
H  -1.46261  1.84619  4.73054
H  -1.92998  3.18254  3.63432
H  -2.97015  1.73184  3.77159

59
10a pi
SCF(BP86)= -1430.40377468
G(298 K) = -1430.016994
SCF(PBE) = -3989.60459486
Low Freq. = 15.1011cm-1,
23.4507cm-1
C   4.55758 -1.32785  0.96287
C   3.45384 -0.64507  0.39902
C   2.14725 -0.72886  0.98643
C   2.01970 -1.52817  2.15101
C   3.11936 -2.20416  2.70630
C   4.39491 -2.10719  2.11423
C   3.54522  0.18694 -0.80176
C   4.71550  0.40470 -1.55722
C   4.68814  1.21583 -2.69539
C   3.46840  1.80859 -3.06352
C   2.33897  1.56393 -2.27903
N   2.34367  0.77482 -1.16920
Ru  0.71340  0.33183  0.07341
Br -5.06112  0.31094 -0.70938
H   5.64937 -0.07056 -1.24514
H   5.59610  1.38291 -3.28231
H   3.38308  2.45362 -3.94203
H   1.38128  2.01644 -2.54482
H   5.25087 -2.63423  2.54820
H   2.98285 -2.81241  3.60883
H   1.04153 -1.62504  2.63452
N  -0.81105 -0.37213  1.28567
C  -1.27021  0.24969  2.40689
H  -0.80180  1.21023  2.63197
C  -2.25840 -0.28878  3.23384
H  -2.57478  0.26154  4.12413
C  -2.81998 -1.53292  2.89576
C  -2.36964 -2.17636  1.73833
H  -3.59245 -1.99046  3.52117
H  -2.79015 -3.14370  1.45008
C  -1.36842 -1.59307  0.93544
C  -0.81267 -2.16703 -0.29343
C   0.21880 -1.38349 -0.91508
C   0.77489 -1.91507 -2.10708
H   1.57041 -1.36096 -2.62127
C   0.33795 -3.13212 -2.65950
H   0.79291 -3.50631 -3.58519
C  -0.67990 -3.87734 -2.03063
H  -1.02100 -4.82561 -2.45925
C  -1.25253 -3.39203 -0.84769
C  -3.30554  1.11989 -0.79324
C  -2.37018  0.62060 -1.69520
C  -3.02802  2.21917  0.04813
C  -1.09275  1.23364 -1.76758
C  -1.77278  2.82412 -0.03914
C  -0.79395  2.34245 -0.94303
H  -2.61177 -0.22169 -2.34713
H  -3.78630  2.59631  0.73883
H  -0.40855  0.90747 -2.55420
H  -1.55082  3.69222  0.58914
H  -2.04426 -3.97327 -0.35971
H   0.12929  2.91066 -1.07744
H   5.55046 -1.25139  0.50425
N   1.18596  1.96846  1.29605
C   1.53003  2.84079  2.00925
C   1.94987  3.91848  2.89799
H   2.23285  3.51457  3.88429
H   2.81798  4.45025  2.47450
H   1.12961  4.64201  3.03710

60
10a OAc
SCF(BP86)= -1526.27010094
G(298 K) = -1525.882296
SCF(PBE) = -4085.42435155
Low Freq. = 13.4734cm-1,
21.2392cm-1
C   0.82383  2.94977 -2.61750
C   0.35118  2.26345 -1.47479
C  -0.03836  0.87253 -1.52763
C   0.04948  0.25849 -2.81112
C   0.51543  0.94452 -3.94458
C   0.91176  2.29615 -3.85349
C   0.21576  2.89134 -0.15852
N  -0.24524  2.02319  0.82107
C  -0.41362  2.50017  2.08641
C  -0.15057  3.82495  2.44384
C   0.31447  4.71588  1.45827
C   0.49811  4.23639  0.15635
Ru -0.61111  0.06971  0.22136
O  -0.76452 -0.42287  2.41416
C  -1.20246 -1.46403  3.06997
C  -0.91263 -1.40254  4.58335
H   1.12495  4.00259 -2.54729
C  -2.51454  0.35518 -0.24739
C  -3.18900 -0.77775 -0.83367
C  -4.56142 -0.72290 -1.16969
C  -5.30127  0.44651 -0.95131
C  -4.65913  1.57407 -0.39540
C  -3.29734  1.52825 -0.05620
H  -5.05895 -1.59921 -1.60377
C  -2.34151 -1.95013 -1.04112
C  -2.73260 -3.16352 -1.63907
C  -1.81550 -4.20843 -1.80212
C  -0.49435 -4.00882 -1.35785
C  -0.15504 -2.79072 -0.76675
N  -1.03872 -1.76515 -0.58730
```

O -1.80968 -2.45177 2.59624
H -3.76504 -3.27993 -1.98239
H -2.11894 -5.15098 -2.26757
H   0.26932 -4.78488 -1.46359
H   0.85950 -2.60011 -0.40557
H -6.36417 0.48422 -1.21255
H -5.23005 2.49591 -0.22608
H -2.82699 2.42022 0.37376
H -0.75218 1.74856 2.80611
H -0.30633 4.14613 3.47788
H   0.53059 5.76099 1.70074
H   0.86065 4.90611 -0.62897
H -0.25106 -0.79112 -2.91918
H   0.57209 0.42632 -4.91058
H   1.27873 2.83029 -4.73641
H -1.30717 -2.29111 5.09896
H -1.36661 -0.49808 5.02380
H   0.17461 -1.33428 4.76108
Br 1.91887 -0.58003 1.37503
C   3.52314 -0.73768 0.29285
C   4.73349 -1.06237 0.92494
C   3.43844 -0.53289 -1.09231
C   5.89234 -1.18396 0.13816
C   4.60996 -0.65982 -1.86062
C   5.83366 -0.98407 -1.25161
H   4.77543 -1.21849 2.00685
H   2.48174 -0.27681 -1.55911
H   6.84207 -1.43700 0.62051
H   4.55784 -0.50201 -2.94286
H   6.73980 -1.08062 -1.85765

60
10a OAc pi
SCF(BP86)= -1526.26037598
G(298 K) = -1525.872867
SCF(PBE) = -4085.42182880
Low Freq. = 15.9325cm-1,
23.0022cm-1
C   4.46396 -1.21395 1.29183
C   3.37244 -0.71358 0.54387
C   2.06890 -0.56980 1.12661
C   1.93608 -0.95927 2.48508
C   3.02272 -1.45440 3.22647
C   4.29406 -1.58498 2.63182
C   3.47437 -0.29882 -0.85720
C   4.64769 -0.35283 -1.63492
C   4.64555 0.07516 -2.96726
C   3.44287 0.56907 -3.49965
C   2.30782 0.60315 -2.68565
N   2.28017 0.17326 -1.39109
Ru 0.65643 0.19657 -0.07898
Br -5.02149 0.11001 -0.93258
H   5.56885 -0.73294 -1.18412
H   5.55701 0.03088 -3.57077
H   3.37424 0.93046 -4.52964
H   1.37018 0.99512 -3.08490
H   5.14145 -1.97087 3.20845
H   2.88043 -1.74114 4.27600
H   0.96059 -0.86991 2.97649
N -0.84814 -0.00771 1.32795
C -1.25081 0.96860 2.19194
H -0.75758 1.94311 2.05107
C -2.21352 0.75150 3.18323
H -2.48619 1.57284 3.85298
C -2.81333 -0.51599 3.29639
C -2.42538 -1.51648 2.39904
H -3.56686 -0.71893 4.06416
H -2.87522 -2.51208 2.45619
C -1.44567 -1.25723 1.41860
C -0.94777 -2.21599 0.43196
C   0.08050 -1.70154 -0.43223
C   0.59020 -2.60732 -1.40271
H   1.37923 -2.26812 -2.08563
C   0.11465 -3.92400 -1.52071
H   0.53523 -4.58907 -2.28593
C -0.89901 -4.40014 -0.66202
H -1.26988 -5.42667 -0.75358
C -1.42579 -3.54245 0.31217
C -3.21192 0.77820 -1.15237
C -2.29729 0.02649 -1.87582
C -2.88622 2.03308 -0.58510
C -0.97335 0.52776 -2.04770
C -1.59781 2.53004 -0.77487
C -0.62530 1.79532 -1.50639
H -2.58259 -0.92813 -2.32394
H -3.63110 2.59831 -0.01941
H -0.32548 0.00443 -2.75422
H -1.31634 3.48734 -0.32818
H -2.21328 -3.91159 0.98112
H   0.31101 2.28945 -1.77460
H   5.45463 -1.31355 0.83196
O   1.59829 2.25891 0.44693
C   1.29586 3.37029 1.03015
C   2.37256 4.47014 0.89063
H   2.41741 4.81883 -0.15649
H   2.14714 5.33172 1.53789
H   3.37068 4.07327 1.14077
O   0.23916 3.64471 1.66567

53
12a
SCF(BP86)= -1297.62643075
G(298 K) = -1297.281771
SCF(PBE) = -3856.93133745
Low Freq. = 4.7500cm-1, 21.7306cm-
1
C -3.00758 -3.07505 1.64678
C -2.00463 -2.34529 0.96949
C -1.94569 -0.90746 1.04811
C -2.97994 -0.26604 1.78033
C -3.98158 -0.99766 2.44044
C -3.99289 -2.40634 2.38566
C -0.98794 -2.96555 0.11292
C -0.86757 -4.34038 -0.16452
C   0.11221 -4.80251 -1.05131
C   0.96256 -3.86430 -1.66212
C   0.81118 -2.51196 -1.34579
N -0.12369 -2.04748 -0.46965
Ru -0.34768 -0.08812 0.19413
Br 1.94135 0.92410 -0.91667
H -1.55097 -5.04666 0.31497
H   0.20642 -5.87095 -1.26666

```
H   1.73731 -4.16803 -2.37118
H   1.45418 -1.74939 -1.79370
H  -4.76883 -2.97554  2.90786
H  -4.75874 -0.46894  3.00561
H  -2.99313  0.82805  1.84306
N  -0.70002  1.80717  0.90978
C  -0.27497  2.27820  2.11386
H   0.26651  1.55825  2.73626
C  -0.50813  3.58673  2.54414
H  -0.14093  3.90766  3.52253
C  -1.21724  4.46060  1.69887
C  -1.66091  3.98742  0.45841
H  -1.41960  5.49189  2.00275
H  -2.21236  4.64731 -0.21740
C  -1.39917  2.65943  0.06864
C  -1.79269  2.02006 -1.18730
C  -1.38457  0.64294 -1.32230
C  -1.76821 -0.02732 -2.51428
H  -1.49408 -1.07944 -2.64996
C  -2.48528  0.63031 -3.52717
H  -2.76019  0.08364 -4.43750
C  -2.85895  1.98356 -3.38459
H  -3.41813  2.49103 -4.17722
C  -2.51269  2.67148 -2.21491
C   3.55981  0.35491  0.00900
C   3.44749 -0.46265  1.14086
C   4.79731  0.78483 -0.48905
C   4.62712 -0.86150  1.79504
C   5.96362  0.37477  0.18113
C   5.88149 -0.44550  1.31904
H   2.46713 -0.78395  1.50613
H   4.85668  1.42273 -1.37535
H   4.55626 -1.50022  2.68121
H   6.93791  0.70231 -0.19523
H  -2.80751  3.72156 -2.10066
H   6.79424 -0.75979  1.83404
H  -3.02811 -4.17014  1.59527

53
12a pi
SCF(BP86)= -1297.62535426
G(298 K) = -1297.279853
SCF(PBE) = -3856.93228643
Low Freq. = 15.4347cm-1,
23.1462cm-1
C   4.39591  1.38739 -1.52169
C   3.36094  0.59556 -0.97362
C   2.02728  1.11172 -0.81698
C   1.82604  2.47196 -1.17471
C   2.86226  3.25828 -1.70519
C   4.14932  2.71520 -1.89190
C   3.56148 -0.77180 -0.48930
C   4.79486 -1.45064 -0.43972
C   4.88094 -2.72799  0.12368
C   3.71354 -3.30687  0.64924
C   2.51315 -2.59809  0.55978
N   2.40207 -1.36559 -0.01235
Ru  0.65065 -0.23420 -0.26740
Br -4.90513 -0.94394 -0.25022
H   5.69037 -0.96386 -0.83509
H   5.83879 -3.25468  0.16461
```

```
H   3.72188 -4.29250  1.12191
H   1.59304 -3.02495  0.96581
H   4.95365  3.32618 -2.31428
H   2.66701  4.30247 -1.97740
H   0.83794  2.92562 -1.04444
N  -0.89364  1.11892 -0.45984
C  -1.52972  1.43863 -1.62011
H  -1.20783  0.87940 -2.50231
C  -2.52332  2.41754 -1.70308
H  -2.98971  2.63175 -2.66827
C  -2.89702  3.10331 -0.53367
C  -2.24788  2.78865  0.66545
H  -3.67283  3.87428 -0.55868
H  -2.50927  3.31593  1.58705
C  -1.24249  1.80198  0.69392
C  -0.45244  1.40670  1.86043
C   0.57310  0.43058  1.60457
C   1.38105  0.03939  2.70201
H   2.18494 -0.68860  2.55013
C   1.17114  0.56234  3.98931
H   1.81271  0.23543  4.81655
C   0.14970  1.50484  4.22597
H  -0.01129  1.90885  5.23059
C  -0.65647  1.92398  3.16059
C  -3.06017 -1.46928 -0.47325
C  -2.27698 -1.70392  0.65862
C  -2.55131 -1.63403 -1.77964
C  -0.93531 -2.12653  0.49797
C  -1.22822 -2.05396 -1.93902
C  -0.38908 -2.30206 -0.81082
H  -2.69936 -1.59595  1.66091
H  -3.18834 -1.45614 -2.64973
H  -0.38535 -2.44161  1.38871
H  -0.84133 -2.23268 -2.94755
H  -1.45023  2.65783  3.34342
H   0.52437 -2.88557 -0.95850
H   5.40082  0.97130 -1.65796

53
TS(12a-13c)
SCF(BP86)= -1297.62064335
G(298 K) = -1297.275051
SCF(PBE) = -3856.92690890
Low Freq. = -50.1580cm-1,
23.0584cm-1
C  -3.16190  0.50747  2.83401
C  -2.40487  0.30714  1.65641
C  -0.97217  0.46249  1.65203
C  -0.37485  0.87235  2.87493
C  -1.13515  1.07375  4.03824
C  -2.53285  0.88632  4.02586
C  -2.99603 -0.05670  0.36863
C  -4.37195 -0.20652  0.10264
C  -4.81599 -0.51882 -1.18646
C  -3.86200 -0.67313 -2.20768
C  -2.50947 -0.52163 -1.89231
N  -2.05909 -0.23179 -0.63881
Ru -0.06611  0.01542 -0.05663
Br  1.02236 -1.31881 -2.37045
H  -5.09289 -0.06888  0.91308
H  -5.88421 -0.63302 -1.39306
```

H -4.15253 -0.90673 -3.23534
H -1.73567 -0.63330 -2.65618
H -3.12251 1.04075 4.93524
H -0.63581 1.38224 4.96480
H   0.70777 1.03015 2.92052
N   1.82899 0.51104 0.62318
C   2.61163 -0.23678 1.44910
H   2.17227 -1.17818 1.78719
C   3.89533 0.14839 1.84404
H   4.47330 -0.50192 2.50597
C   4.41198 1.36818 1.37137
C   3.60463 2.16657 0.55407
H   5.41832 1.69664 1.64774
H   3.96975 3.13271 0.19488
C   2.30768 1.74265 0.20204
C   1.32156 2.51386 -0.55438
C   0.03064 1.88819 -0.69007
C -0.98927 2.65435 -1.31529
H -1.99596 2.23511 -1.41421
C -0.73563 3.93744 -1.82712
H -1.54509 4.49576 -2.31284
C   0.54762 4.51348 -1.72394
H   0.74288 5.51088 -2.13087
C   1.56912 3.80152 -1.08355
C   0.99785 -2.43817 -0.78114
C -0.24303 -2.84996 -0.24930
C   2.22284 -2.92319 -0.28450
C -0.23087 -3.70436 0.87511
C   2.20011 -3.79067 0.81787
C   0.97931 -4.16840 1.41018
H -1.18521 -2.57720 -0.72677
H   3.16776 -2.61976 -0.74112
H -1.18547 -4.02539 1.30340
H   3.14646 -4.17091 1.21504
H   2.56276 4.25530 -0.98911
H   0.97469 -4.84309 2.27124
H -4.24944 0.36900 2.82259

53
13c
SCF(BP86)= -1297.64323390
G(298 K) = -1297.297910
SCF(PBE) = -3856.94331853
Low Freq. = 27.0939cm-1,
32.3725cm-1
C -0.02633 4.88414 -0.57218
C -1.14387 4.07046 -0.80158
C -1.12856 2.70935 -0.42337
C   0.05046 2.13778 0.11490
C   1.16229 2.97135 0.37419
C   1.12189 4.33099 0.02759
Ru -0.05872 0.10508 -0.02898
C   0.64073 -1.58795 0.83949
C   1.96225 -1.95575 0.45371
C   2.47914 -3.21532 0.83137
C   1.69574 -4.10029 1.58350
C   0.38800 -3.73963 1.96248
C -0.13077 -2.48194 1.60963
C   2.67651 -0.99056 -0.38446
N   1.90396 0.08805 -0.76358
C   2.44313 1.00299 -1.61380

C   3.75343 0.91665 -2.09181
C   4.55857 -0.15664 -1.67724
C   4.00908 -1.11563 -0.82067
H   3.48696 -3.51473 0.52510
H -0.05362 5.94630 -0.83608
C -0.83701 -0.58231 -1.76330
C -2.22775 -0.92381 -1.67934
C -2.89330 -1.47752 -2.79181
C -2.20115 -1.68598 -3.99507
C -0.84144 -1.34421 -4.09712
C -0.16821 -0.80133 -2.98832
H -3.95237 -1.74877 -2.73297
C -2.89938 -0.64121 -0.39726
C -4.26153 -0.85518 -0.12724
C -4.79451 -0.49503 1.11808
C -3.94866 0.08452 2.07403
C -2.59734 0.26855 1.75780
N -2.07130 -0.09510 0.56131
Br 0.72888 0.70647 2.71991
H -4.90500 -1.29641 -0.89201
H -5.85424 -0.66033 1.33242
H -4.31754 0.39066 3.05619
H -1.88163 0.69624 2.46641
H -2.72635 -2.11531 -4.85405
H -0.30417 -1.50690 -5.03749
H   0.89190 -0.54907 -3.08461
H   1.78549 1.82236 -1.90838
H   4.12657 1.68312 -2.77561
H   5.59202 -0.24951 -2.02296
H   4.60705 -1.96840 -0.48988
H -1.13570 -2.20107 1.93640
H -0.22568 -4.43482 2.54548
H   2.10169 -5.07492 1.87184
H -2.03586 2.11689 -0.56993
H   2.05740 2.55489 0.84292
H -2.05578 4.48895 -1.24186
H   1.99492 4.96155 0.22978

53
TS(13c-13a)
SCF(BP86)= -1297.62688853
G(298 K) = -1297.281920
SCF(PBE) = -3856.92824107
Low Freq. = -157.2485cm-1,
26.6219cm-1
C   2.73439 -1.71027 2.73367
C   2.13151 -1.13846 1.58922
C   0.77616 -0.66316 1.61451
C   0.07226 -0.80517 2.83271
C   0.67643 -1.37310 3.96943
C   2.00847 -1.82677 3.92519
C   2.82842 -0.97530 0.31420
C   4.16965 -1.31480 0.04761
C   4.71600 -1.07855 -1.21702
C   3.91791 -0.50043 -2.22107
C   2.59292 -0.18767 -1.92078
N   2.05528 -0.41818 -0.69162
Ru 0.10131 0.04999 -0.15038
H   4.78172 -1.75821 0.83622
H   5.75871 -1.34081 -1.41942
H   4.30957 -0.29758 -3.22039

```
H   1.90250  0.24712 -2.65049
H   2.47407 -2.26933  4.81107
H   0.10109 -1.46416  4.89801
H  -0.96611 -0.46660  2.90338
N  -1.95177  0.50029  0.69979
C  -2.27833  1.54519  1.50509
H  -1.46096  2.20694  1.79195
C  -3.57536  1.79348  1.96279
H  -3.75651  2.65628  2.60837
C  -4.61049  0.92672  1.57868
C  -4.28550 -0.18822  0.80410
H  -5.64170  1.10233  1.89837
H  -5.05120 -0.91409  0.51900
C  -2.95144 -0.40242  0.40599
C  -2.45196 -1.59724 -0.25455
C  -1.05573 -1.57808 -0.42431
C  -0.38123 -2.70858 -0.93088
H   0.70646 -2.73166 -1.04449
C  -1.13949 -3.83144 -1.31453
H  -0.61859 -4.70889 -1.71317
C  -2.54232 -3.84189 -1.18149
H  -3.11409 -4.72448 -1.48319
C  -3.20111 -2.73029 -0.64386
C   0.24644  2.02653  0.31283
C  -0.43624  3.04192 -0.39700
C   1.36672  2.35360  1.10927
C   0.07417  4.34823 -0.40043
C   1.88393  3.66747  1.07356
C   1.24082  4.66221  0.32545
H  -1.33444  2.80257 -0.97062
H   1.85376  1.60209  1.73490
H  -0.44177  5.12357 -0.97692
H   2.77493  3.90469  1.66499
Br -0.67802  0.62581 -2.78054
H  -4.28898 -2.74405 -0.51968
H   1.62940  5.68567  0.32247
H   3.76853 -2.07006  2.69772
```

```
53
13a
SCF(BP86)= -1297.65903475
G(298 K) = -1297.311982
SCF(PBE) = -3856.96577940
Low Freq. = 33.9630cm-1,
41.9340cm-1
C   2.89678 -0.76385  2.68705
C   2.22214 -0.50705  1.46823
C   0.83796 -0.17186  1.46048
C   0.16195 -0.13547  2.69788
C   0.83375 -0.39160  3.90323
C   2.20902 -0.69901  3.90201
C   2.85688 -0.61060  0.15740
C   4.22089 -0.88364 -0.07606
C   4.70500 -0.97582 -1.38245
C   3.81094 -0.79314 -2.45184
C   2.47082 -0.52519 -2.16952
N   1.98764 -0.43445 -0.89979
Ru -0.00470  0.10760 -0.43296
H   4.89631 -1.01686  0.77243
H   5.76291 -1.18489 -1.56623
H   4.13926 -0.85589 -3.49222
```

```
H   1.72922 -0.38667 -2.96083
H   2.73342 -0.89781  4.84170
H   0.28146 -0.35469  4.84887
H  -0.91085  0.08367  2.72614
N  -2.00436  0.63091 -0.05601
C  -2.54868  1.82572 -0.40759
H  -1.86221  2.55091 -0.84797
C  -3.90006  2.12351 -0.22087
H  -4.28152  3.10189 -0.52308
C  -4.73496  1.15261  0.35940
C  -4.19179 -0.09010  0.69335
H  -5.79524  1.35792  0.53291
H  -4.82200 -0.87406  1.12047
C  -2.82546 -0.34634  0.45981
C  -2.14505 -1.62262  0.65287
C  -0.76793 -1.63319  0.30052
C  -0.05262 -2.84543  0.35699
H   1.00988 -2.87182  0.09763
C  -0.69943 -4.03009  0.74868
H  -0.13478 -4.96848  0.77557
C  -2.05852 -4.01416  1.12044
H  -2.55245 -4.93654  1.44109
C  -2.77942 -2.81669  1.06995
C   0.41516  1.94465  0.37806
C   1.32797  2.59405 -0.48953
C  -0.20935  2.69889  1.39318
C   1.53337  3.98715 -0.39213
C   0.02282  4.07995  1.50036
C   0.88874  4.73334  0.60433
H   1.87846  2.04046 -1.25693
H  -0.89251  2.21660  2.09715
H   2.22997  4.47067 -1.08595
H  -0.48172  4.64739  2.29031
Br -0.87994 -0.51162 -2.82089
H  -3.84141 -2.81199  1.33748
H   1.06978  5.80914  0.69425
H   3.96016 -1.02588  2.68361
```

```
53
12b
SCF(BP86)= -1297.61599810
G(298 K) = -1297.271252
SCF(PBE) = -3856.92311214
Low Freq. = 14.9963cm-1,
20.8351cm-1
C  -5.68023  1.02673  0.51267
C  -5.03034  1.76394  1.51669
C  -3.65886  2.05938  1.40772
C  -2.97154  1.60363  0.27561
C  -3.59246  0.86738 -0.74144
C  -4.96307  0.57907 -0.60988
Br -1.06149  2.06106  0.08978
Ru  0.56634  0.14923  0.32250
C   0.35813 -0.61350 -1.48288
C  -0.64076 -1.64646 -1.63256
C  -0.89269 -2.23328 -2.89606
C  -0.17083 -1.83452 -4.02638
C   0.81881 -0.83744 -3.89618
C   1.08004 -0.24495 -2.65056
C  -1.36608 -2.02775 -0.41598
N  -0.97719 -1.31740  0.70053
```

C -1.57474 -1.57890 1.88985
C -2.57566 -2.54251 2.04555
C -2.97915 -3.27514 0.91386
C -2.37073 -3.01443 -0.31825
N   2.11448 -1.08009 0.79098
C   3.38036 -0.50917 0.85449
C   4.48166 -1.28002 1.27563
C   4.32492 -2.62972 1.60732
C   3.04564 -3.20414 1.50297
C   1.97800 -2.40406 1.09347
C   3.41403 0.88871 0.42427
C   4.60197 1.65398 0.37927
C   4.57715 2.96652 -0.10850
C   3.35608 3.50626 -0.56163
C   2.17677 2.74461 -0.50780
C   2.13793 1.41374 -0.00086
H   5.55346 1.22781 0.71979
H   5.49642 3.56035 -0.14399
H   3.32637 4.52876 -0.95798
H   1.25399 3.20839 -0.88491
H   5.18340 -3.22468 1.93246
H   2.86775 -4.25762 1.73441
H   0.96707 -2.80528 1.00448
H -1.22009 -0.98167 2.73709
H -3.02305 -2.71179 3.02843
H -3.75815 -4.03957 0.99305
H -2.67263 -3.57443 -1.20753
H   1.85282 0.52650 -2.57186
H   1.39273 -0.52181 -4.77598
H -0.37189 -2.29477 -4.99909
H -3.14508 2.62988 2.18665
H -3.02534 0.52379 -1.61068
H -5.58551 2.11346 2.39299
H -5.46678 0.00442 -1.39364
H -1.65987 -3.00991 -2.99750
H -6.74693 0.80088 0.60493
H   5.46784 -0.81149 1.33345

53
TS(12b-13b)
SCF(BP86)= -1297.60573901
G(298 K) = -1297.260556
SCF(PBE) = -3856.91239842
Low Freq. = -81.7163cm-1,
22.0135cm-1
C -0.73280 4.31151 1.40454
C   0.46826 3.82465 0.87076
C   0.46495 2.91725 -0.21183
C -0.78611 2.46904 -0.69151
C -2.00537 2.98623 -0.20607
C -1.96432 3.90392 0.85255
Br -0.82897 1.30280 -2.25893
Ru 0.08679 0.05830 -0.06716
C -0.14783 -1.78275 -0.78044
C -1.47122 -2.34440 -0.67340
C -1.77534 -3.60123 -1.24999
C -0.79002 -4.34297 -1.91057
C   0.51981 -3.82839 -1.99131
C   0.83217 -2.57821 -1.43354
C -2.44986 -1.56812 0.09574
N -1.95465 -0.37835 0.59015

C -2.74416 0.35339 1.41931
C -4.05119 -0.00865 1.75930
C -4.58231 -1.18994 1.21202
C -3.77197 -1.97203 0.38321
N   0.96416 -0.57549 1.64463
C   2.35447 -0.59256 1.67838
C   3.02659 -0.99629 2.84984
C   2.31318 -1.39291 3.98422
C   0.90784 -1.38445 3.93134
C   0.27908 -0.97767 2.75417
C   2.97955 -0.16450 0.42939
C   4.38039 -0.12103 0.23939
C   4.91170 0.27995 -0.99245
C   4.03104 0.63702 -2.03406
C   2.63944 0.59615 -1.84153
C   2.04720 0.19866 -0.60844
H   5.06256 -0.40267 1.05068
H   5.99578 0.31262 -1.14275
H   4.43619 0.94802 -3.00518
H   1.99393 0.87924 -2.68258
H   2.83995 -1.70644 4.89025
H   0.29801 -1.69118 4.78515
H -0.80806 -0.95973 2.66707
H -2.29374 1.27004 1.81017
H -4.63415 0.62628 2.43178
H -5.60617 -1.50245 1.43895
H -4.15737 -2.90635 -0.03353
H   1.85421 -2.19652 -1.51839
H   1.30298 -4.40620 -2.49697
H -1.03521 -5.31441 -2.35196
H   1.39716 2.61049 -0.68896
H -2.95686 2.66527 -0.63594
H   1.42953 4.17123 1.26338
H -2.90409 4.30447 1.24559
H -2.79009 -4.00895 -1.17670
H -0.71491 5.02928 2.23000
H   4.11989 -0.99831 2.86004

53
13b
SCF(BP86)= -1297.64819183
G(298 K) = -1297.302048
SCF(PBE) = -3856.95170346
Low Freq. = 24.6413cm-1,
32.3867cm-1
C   0.79580 4.46922 1.53542
C   0.06476 3.56863 2.32620
C -0.19861 2.26395 1.86616
C   0.26765 1.84406 0.60835
C   1.00888 2.74923 -0.18964
C   1.26185 4.05522 0.27722
Ru 0.05364 0.06478 -0.39574
C -0.80298 -1.74543 -0.07339
C -2.18498 -1.86218 0.23633
C -2.77734 -3.14224 0.33358
C -2.01201 -4.29465 0.11991
C -0.64418 -4.18015 -0.18882
C -0.03422 -2.91453 -0.26151
C -2.91704 -0.60116 0.35870
N -2.13123 0.49826 0.14228
C -2.72178 1.71583 0.10028

C -4.08933 1.90771 0.32213
C -4.89115 0.78771 0.59945
C -4.29957 -0.47810 0.60985
N 0.99632 -0.55547 1.39531
C 2.35495 -0.75425 1.34839
C 3.05612 -1.13208 2.51303
C 2.36925 -1.33141 3.71244
C 0.97394 -1.16362 3.73227
C 0.32999 -0.77664 2.55555
C 2.92529 -0.58888 0.01375
C 4.29839 -0.75246 -0.27289
C 4.75467 -0.63626 -1.59168
C 3.83765 -0.35937 -2.62350
C 2.47265 -0.18558 -2.33690
C 1.97226 -0.28739 -1.01477
Br -0.83670 0.58957 -2.71521
H 5.01410 -0.97175 0.52686
H 5.81852 -0.76399 -1.81575
H 4.19023 -0.27509 -3.65779
H 1.78023 0.03732 -3.15505
H 2.90978 -1.62551 4.61670
H 0.38689 -1.32741 4.63905
H -0.75326 -0.64216 2.51740
H -2.06170 2.55865 -0.11463
H -4.50890 2.91609 0.28017
H -5.96273 0.89899 0.78978
H -4.90333 -1.36967 0.79645
H 1.03625 -2.84433 -0.47244
H -0.04003 -5.07828 -0.35635
H -2.47902 -5.28139 0.19583
H -0.78922 1.59174 2.49452
H 1.39018 2.45707 -1.17292
H -0.31234 3.87498 3.30828
H 1.83342 4.74188 -0.35716
H -3.84332 -3.23767 0.56462
H 0.99954 5.48330 1.89383
H 4.13783 -1.27864 2.46490

53
12c
SCF(BP86)= -1297.60594280
G(298 K) = -1297.261563
SCF(PBE) = -3856.91076631
Low Freq. = 13.5245cm-1,
17.2899cm-1
N 0.67127 1.89221 0.44766
C 0.10419 3.04510 -0.06179
C 0.72934 4.29521 0.12997
C 1.91698 4.38414 0.86467
C 2.46883 3.21098 1.40855
C 1.81595 1.99772 1.17124
C -1.17838 2.82845 -0.74962
C -1.64602 1.46447 -0.83313
C -2.94717 1.27062 -1.37222
C -3.71688 2.33850 -1.86057
C -3.21779 3.65610 -1.81907
C -1.95628 3.89455 -1.26001
Ru -0.35438 0.07571 -0.22709
C -1.34786 -1.48275 -1.01281
C -2.28494 -2.12762 -0.13016
C -3.03917 -3.25513 -0.52860

C -2.88398 -3.77623 -1.81986
C -1.97083 -3.16748 -2.70951
C -1.22249 -2.04648 -2.31190
C -2.36192 -1.49998 1.18502
N -1.49384 -0.41779 1.33020
C -1.48064 0.26511 2.51175
C -2.28544 -0.08741 3.59590
C -3.15690 -1.18548 3.47145
C -3.18794 -1.88294 2.25997
Br 1.55602 -1.70047 0.58721
C 3.31779 -1.24385 -0.10536
C 3.40059 -0.47505 -1.27344
C 4.67544 -0.15656 -1.77403
C 5.83125 -0.60507 -1.11321
C 5.71772 -1.37689 0.05545
C 4.45254 -1.70563 0.57479
H 0.28151 5.19824 -0.29343
H 2.40195 5.35347 1.01517
H 3.38829 3.22865 1.99972
H 2.21218 1.05670 1.56567
H -3.81324 4.48916 -2.20672
H -4.71097 2.14496 -2.28171
H -3.35175 0.25387 -1.42257
H -0.52398 -1.59483 -3.03119
H -1.84791 -3.57476 -3.72088
H -3.46495 -4.64910 -2.13537
H -3.74480 -3.73109 0.16371
H -0.78754 1.10808 2.55291
H -2.22528 0.49663 4.51796
H -3.80128 -1.48694 4.30236
H 2.49326 -0.13238 -1.77934
H 4.35968 -2.30381 1.48541
H 4.75717 0.44362 -2.68579
H 6.61490 -1.72860 0.57452
H -3.85953 -2.73655 2.13085
H 6.81993 -0.35386 -1.50886
H -1.58379 4.92420 -1.20854

53
TS(12c-13e)
SCF(BP86)= -1297.60064007
G(298 K) = -1297.255590
SCF(PBE) = -3856.90869985
Low Freq. = -29.1696cm-1,
22.9487cm-1
N 2.09142 0.48687 -0.65603
C 3.01108 0.32994 0.36262
C 4.36408 0.68044 0.16716
C 4.79286 1.17934 -1.06743
C 3.85500 1.31865 -2.10538
C 2.52671 0.96314 -1.85289
C 2.44803 -0.23754 1.59546
C 1.04442 -0.57639 1.57192
C 0.52003 -1.18643 2.74508
C 1.30765 -1.40603 3.88619
C 2.66782 -1.03640 3.89862
C 3.22978 -0.45817 2.75472
Ru 0.04406 -0.07440 -0.07143
C -1.75460 -0.71343 0.56818
C -2.12438 -2.03071 0.11541
C -3.36914 -2.61924 0.43850

```
C  -4.28420 -1.92375  1.23841
C  -3.94407 -0.64045  1.72018
C  -2.70956 -0.05522  1.39300
C  -1.08337 -2.69497 -0.66020
N   0.06794 -1.92395 -0.81934
C   1.13492 -2.46410 -1.47880
C   1.11231 -3.74331 -2.03479
C  -0.05215 -4.52295 -1.90844
C  -1.14225 -3.98856 -1.21555
Br -1.13410  1.40739 -2.17930
C  -1.28867  2.56951 -0.63499
C  -2.56194  2.99120 -0.22311
C  -2.65330  3.81348  0.91184
C  -1.49767  4.19299  1.61946
C  -0.23531  3.76810  1.17992
C  -0.11157  2.95897  0.03077
H   5.08103  0.55638  0.98306
H   5.84200  1.45027 -1.21964
H   4.13974  1.69587 -3.09119
H   1.76009  1.06102 -2.62809
H   3.28259 -1.20389  4.78894
H   0.85998 -1.86890  4.77410
H  -0.53500 -1.47815  2.76068
H  -2.48132  0.94376  1.78648
H  -4.65286 -0.09616  2.35698
H  -5.24922 -2.37441  1.49287
H  -3.62636 -3.62100  0.07262
H   2.01269 -1.82045 -1.55388
H   1.99897 -4.11407 -2.55573
H  -0.10405 -5.52858 -2.33568
H  -3.45755  2.68071 -0.76612
H   0.87292  2.67708 -0.35024
H  -3.63904  4.15305  1.24481
H   0.67044  4.07770  1.71035
H  -2.05693 -4.57475 -1.08999
H  -1.58356  4.83000  2.50463
H   4.28979 -0.17951  2.76629

53
13e
SCF(BP86)= -1297.62835665
G(298 K) = -1297.283760
SCF(PBE) = -3856.92829750
Low Freq. = 28.4578cm-1,
31.1989cm-1
N  -1.91981  0.77335 -0.65394
C  -2.40095  1.70187  0.23493
C  -3.58621  2.42086 -0.02255
C  -4.31014  2.16072 -1.18976
C  -3.83263  1.18259 -2.07753
C  -2.63862  0.51884 -1.77146
C  -1.58012  1.82181  1.44048
C  -0.37960  1.04284  1.45101
C   0.48546  1.13155  2.57258
C   0.11024  1.88377  3.69772
C  -1.08858  2.62215  3.69142
C  -1.92525  2.59784  2.56602
Ru -0.00482 -0.25824 -0.01853
C   1.75281 -1.11964  0.48147
C   2.95145 -0.33753  0.30127
C   4.21161 -0.83034  0.70096
```

```
C   4.32431 -2.10278  1.27887
C   3.17148 -2.88977  1.45921
C   1.91428 -2.41186  1.05446
C   2.77169  0.99673 -0.28205
N   1.46421  1.35408 -0.47378
C   1.16634  2.56147 -1.00187
C   2.14861  3.48538 -1.37053
C   3.49823  3.13287 -1.18946
C   3.80950  1.88398 -0.64648
Br  0.40698 -0.64322 -2.53391
C  -1.17597 -1.91196  0.18420
C  -0.91063 -3.17692 -0.41343
C  -1.67786 -4.31236 -0.11079
C  -2.74081 -4.22888  0.80958
C  -3.03890 -2.99041  1.40222
C  -2.29389 -1.84478  1.06878
H  -3.93408  3.17498  0.68800
H  -5.23312  2.70823 -1.40198
H  -4.36916  0.93447 -2.99701
H  -2.21622 -0.23706 -2.43896
H  -1.36580  3.22622  4.56078
H   0.76731  1.91056  4.57346
H   1.43518  0.59087  2.57540
H   1.03643 -3.04710  1.20090
H   3.25402 -3.88261  1.91600
H   5.30506 -2.47686  1.58936
H   5.11236 -0.21976  0.57608
H   0.10153  2.77814 -1.12733
H   1.85906  4.45304 -1.78781
H   4.29724  3.82424 -1.47324
H  -0.08833 -3.26643 -1.13259
H  -2.56718 -0.89027  1.53147
H  -1.44520 -5.26883 -0.59330
H  -3.86881 -2.90610  2.11366
H   4.85306  1.58763 -0.51444
H  -3.33746 -5.11559  1.04851
H  -2.85420  3.17722  2.57356

53
12d
SCF(BP86)= -1297.58475657
G(298 K) = -1297.240419
SCF(PBE) = -3856.88909993
Low Freq. = 12.7727cm-1,
21.3380cm-1
C   0.63171  1.95236 -0.02716
C  -0.20213  3.11258 -0.20043
C   0.31065  4.43029 -0.15446
C   1.67406  4.64220  0.08828
C   2.52039  3.53287  0.28527
C   2.00412  2.22571  0.22244
C  -1.62866  2.82312 -0.38679
N  -1.94176  1.46926 -0.42519
C  -3.25299  1.10224 -0.50787
C  -4.29757  2.02425 -0.59546
C  -3.99306  3.39583 -0.59973
C  -2.65442  3.78419 -0.49085
Ru -0.38832  0.13921 -0.40389
C  -1.56756 -1.52384 -0.93865
C  -2.10387 -2.23377  0.18828
C  -2.93315 -3.37042  0.04083
```

C -3.24633 -3.83896 -1.24211
C -2.73163 -3.16933 -2.37216
C -1.91017 -2.03752 -2.21588
C -1.72745 -1.65949 1.48117
N -0.91871 -0.52829 1.39027
C -0.51878 0.09485 2.53869
C -0.87858 -0.35989 3.80732
C -1.69005 -1.50351 3.91877
C -2.10805 -2.14374 2.74946
Br 1.44197 -1.56577 -0.74746
C 3.32894 -1.13412 -0.34851
C 4.17564 -0.77353 -1.40478
C 5.52491 -0.49931 -1.11516
C 6.00010 -0.58584 0.20430
C 5.12908 -0.94959 1.24540
C 3.77719 -1.23016 0.97578
H -0.34435 5.29674 -0.30635
H 2.07416 5.66100 0.12657
H 3.58759 3.69247 0.48456
H 2.69811 1.38866 0.37387
H -4.78406 4.14769 -0.67651
H -5.32689 1.66256 -0.66294
H -3.43462 0.02607 -0.51757
H -1.52447 -1.54041 -3.11815
H -2.97402 -3.53685 -3.37709
H -3.88597 -4.71931 -1.36439
H -3.33550 -3.89440 0.91654
H 0.10657 0.97617 2.38556
H -0.52444 0.18246 4.68780
H -1.99109 -1.88518 4.89864
H 3.79754 -0.70455 -2.42887
H 3.09256 -1.51373 1.78022
H 6.20123 -0.21655 -1.92814
H 5.49658 -1.01909 2.27421
H -2.74160 -3.03322 2.80460
H 7.05068 -0.37025 0.42168
H -2.39171 4.84512 -0.47342

53
13d
SCF(BP86)= -1297.64178009
G(298 K) = -1297.295973
SCF(PBE) = -3856.94360106
Low Freq. = 28.2268cm-1,
31.3612cm-1
C 5.13295 0.88000 -0.33576
C 4.57804 -0.28626 0.21719
C 3.18402 -0.47132 0.23185
C 2.29673 0.48641 -0.33261
C 2.88861 1.64454 -0.91165
C 4.27893 1.84783 -0.89443
Ru 0.28284 0.17637 -0.36837
N -2.11010 -0.25257 -0.51069
C -2.87001 0.65170 0.17690
C -4.26793 0.50231 0.29409
C -4.90302 -0.56541 -0.34611
C -4.12524 -1.46161 -1.09549
C -2.73880 -1.26918 -1.13934
C -2.10162 1.78739 0.69142
C -0.69677 1.72090 0.51699
C 0.09370 2.84192 0.85677
C -0.51187 4.01092 1.35793
C -1.90039 4.06121 1.56499
C -2.69023 2.95507 1.22895
N 0.47267 -0.45919 1.67709
C 0.22040 -1.78860 1.93928
C 0.19939 -2.28181 3.25773
C 0.49334 -1.42972 4.32520
C 0.82107 -0.09353 4.04500
C 0.79709 0.34747 2.71886
C 0.08870 -2.58767 0.72727
C 0.05711 -3.99811 0.68018
C 0.11074 -4.65633 -0.55592
C 0.20909 -3.91136 -1.74517
C 0.20301 -2.50246 -1.71381
C 0.14089 -1.83240 -0.47235
Br 0.08754 0.96453 -2.76197
H 0.02172 -4.58215 1.60583
H 0.09140 -5.74977 -0.59236
H 0.27086 -4.42430 -2.71100
H 0.25096 -1.93599 -2.64760
H 0.48219 -1.80251 5.35330
H 1.08848 0.61035 4.83695
H 1.02737 1.38384 2.47162
H 1.17986 2.82125 0.73137
H 0.11701 4.87293 1.60519
H -2.36607 4.96163 1.97679
H -3.77605 3.00954 1.35792
H -2.09766 -1.96003 -1.69296
H -4.57359 -2.30226 -1.63116
H -5.98692 -0.69213 -0.26758
H 2.78336 -1.38816 0.67696
H 2.25219 2.40295 -1.38051
H 5.23006 -1.05331 0.65089
H 4.69667 2.76096 -1.33409
H -4.84860 1.22179 0.87678
H 6.21744 1.03251 -0.33486
H -0.03535 -3.33481 3.43292

11
Ph radical
SCF(BP86)= -231.557620399
G(298 K) = -231.500912
SCF(PBE) = -231.349695163
Low Freq. = 382.5057cm-1,
411.0433cm-1
C -0.00000 1.40688 0.00000
C -1.23477 0.77788 -0.00000
C 1.23477 0.77788 0.00000
C -1.22220 -0.63697 0.00000
C 1.22220 -0.63697 -0.00000
C -0.00000 -1.33264 -0.00000
H -2.18021 1.33119 0.00000
H 2.18021 1.33119 0.00000
H -2.17101 -1.18571 0.00000
H 2.17101 -1.18571 -0.00000
H 0.00000 -2.42738 0.00000

1
bromide
SCF(BP86)= -13.6002179499
G(298 K) = -13.616393

SCF(PBE) = -2573.94659342
Br 0.00000 0.00000 0.00000

53
7a radical
SCF(BP86)= -1317.97296039
G(298 K) = -1317.625828
SCF(PBE) = -1316.89162409
Low Freq. = 27.3500cm-1,
29.6826cm-1

C	2.86478	-2.28621	1.71185
C	2.17298	-1.33654	0.92938
C	0.79094	-1.03278	1.18541
C	0.15560	-1.72325	2.24753
C	0.85099	-2.65929	3.02780
C	2.20583	-2.94383	2.75886
C	2.78118	-0.60453	-0.18407
C	4.10848	-0.74261	-0.63821
C	4.56916	0.03290	-1.70534
C	3.69137	0.94982	-2.30959
C	2.38580	1.05001	-1.82458
N	1.92670	0.29242	-0.79490
Ru	0.00000	0.39085	0.00000
N	-0.73302	1.97022	-1.30064
N	0.73302	1.97022	1.30064
C	1.17613	2.81417	1.98264
C	1.72433	3.86102	2.83122
H	1.89618	3.46559	3.84532
H	2.68219	4.21685	2.41881
H	1.02029	4.70639	2.89054
H	4.77365	-1.45998	-0.15140
H	5.59771	-0.07339	-2.06166
H	4.00569	1.57943	-3.14549
H	1.66442	1.74486	-2.26047
H	3.91689	-2.51593	1.51266
H	2.74455	-3.67827	3.36571
H	0.33921	-3.17518	3.84778
H	-0.89699	-1.52093	2.47202
C	-1.17613	2.81417	-1.98264
C	-1.72433	3.86102	-2.83122
H	-1.89620	3.46559	-3.84531
H	-2.68218	4.21687	-2.41879
H	-1.02028	4.70638	-2.89056
N	-1.92670	0.29242	0.79490
C	-2.38580	1.05001	1.82458
H	-1.66442	1.74486	2.26047
C	-3.69136	0.94982	2.30960
H	-4.00569	1.57943	3.14550
C	-4.56916	0.03290	1.70534
C	-4.10848	-0.74261	0.63820
H	-5.59771	-0.07339	2.06166
H	-4.77365	-1.45998	0.15140
C	-2.78118	-0.60453	0.18407
C	-2.17298	-1.33654	-0.92938
C	-0.79094	-1.03278	-1.18541
C	-0.15560	-1.72324	-2.24753
H	0.89699	-1.52093	-2.47202
C	-0.85099	-2.65928	-3.02781
H	-0.33921	-3.17517	-3.84778
C	-2.20583	-2.94382	-2.75886
H	-2.74455	-3.67826	-3.36572
C	-2.86478	-2.28621	-1.71185
H	-3.91689	-2.51592	-1.51266

54
8a radical
SCF(BP86)= -1413.85155219
G(298 K) = -1413.502464
SCF(PBE) = -1412.73052956
Low Freq. = 19.9479cm-1,
24.4766cm-1

C	2.92862	-2.81566	0.61341
C	2.22918	-1.62683	0.30562
C	0.86284	-1.44297	0.70542
C	0.25320	-2.50188	1.42248
C	0.95610	-3.67581	1.74096
C	2.29465	-3.83777	1.33134
C	2.82838	-0.50396	-0.42395
N	1.98134	0.57237	-0.58488
C	2.43193	1.69165	-1.20688
C	3.72789	1.79488	-1.71825
C	4.60032	0.70122	-1.57758
C	4.14535	-0.44821	-0.92490
Ru	0.04503	0.35660	0.19833
O	-0.36689	2.34361	-0.50329
C	-1.50418	2.84901	-0.93999
C	-1.35347	4.27383	-1.49209
H	3.96993	-2.94810	0.29907
C	-0.74387	-0.56999	-1.40355
C	-2.08754	-1.04883	-1.23569
C	-2.76706	-1.70904	-2.28474
C	-2.13269	-1.90209	-3.51734
C	-0.81344	-1.43782	-3.70547
C	-0.13130	-0.79128	-2.66268
H	-3.79355	-2.06712	-2.14648
C	-2.66966	-0.78761	0.07557
C	-3.95397	-1.17729	0.50729
C	-4.39804	-0.84545	1.78901
C	-3.54142	-0.11818	2.63709
C	-2.27786	0.24097	2.16873
N	-1.83269	-0.08092	0.92292
O	-2.61178	2.28112	-0.92819
H	-4.59934	-1.74206	-0.17059
H	-5.39447	-1.14655	2.12550
H	-3.84315	0.16794	3.64791
H	-1.57624	0.80345	2.78896
H	-2.65976	-2.41043	-4.33122
H	-0.31774	-1.58712	-4.67189
H	0.89546	-0.44746	-2.83210
H	1.69488	2.49712	-1.27948
H	4.03974	2.71527	-2.21873
H	5.62083	0.74569	-1.96946
H	4.80781	-1.30836	-0.79898
H	-0.78956	-2.40564	1.74576
H	0.45919	-4.47334	2.30563
H	2.83887	-4.75690	1.57116
H	-2.32847	4.67974	-1.79867
H	-0.67257	4.27117	-2.36056
H	-0.90367	4.93453	-0.73199
N	0.79686	1.31036	2.00365
C	1.25988	1.81313	2.95692
C	1.83052	2.43815	4.14185

H   1.83190 3.53367 4.02320
H   1.23603 2.17652 5.03204
H   2.86695 2.09411 4.28870

48
9a radical
SCF(BP86)= -1281.09320049
G(298 K) = -1280.784593
SCF(PBE) = -1280.07640129
Low Freq. = 30.5663cm-1,
32.1632cm-1
C -2.60253 -2.10880 -2.18104
C -2.02431 -1.19323 -1.27549
C -0.60960 -0.92143 -1.29981
C   0.16952 -1.60499 -2.26880
C -0.41443 -2.50602 -3.17244
C -1.80071 -2.76168 -3.12694
C -2.78487 -0.45508 -0.26461
N -2.00819 0.42214 0.46974
C -2.59856 1.19740 1.41853
C -3.96410 1.12741 1.70252
C -4.76367 0.22832 0.97388
C -4.16714 -0.56048 -0.01494
Ru 0.00045 0.47532 -0.00376
O   0.34689 2.47258 1.03754
C -0.01538 3.11624 -0.01614
C -0.07080 4.62908 -0.00718
H -3.67885 -2.31216 -2.15799
C   0.62181 -0.90294 1.30673
C   2.03903 -1.16181 1.28635
C   2.62482 -2.06293 2.20146
C   1.82818 -2.71381 3.15311
C   0.43952 -2.47077 3.19471
C -0.15188 -1.58422 2.28163
H   3.70300 -2.25654 2.18139
C   2.79375 -0.42698 0.26860
C   4.17728 -0.52147 0.02177
C   4.76734 0.26262 -0.97471
C   3.95995 1.14589 -1.71391
C   2.59339 1.20564 -1.43225
N   2.00955 0.43480 -0.47590
O -0.36054 2.45832 -1.06667
H   4.78520 -1.21178 0.61235
H   5.84076 0.18942 -1.17175
H   4.37722 1.78160 -2.49894
H   1.91656 1.87670 -1.96842
H   2.28636 -3.40952 3.86328
H -0.18005 -2.98237 3.93997
H -1.23344 -1.41522 2.32540
H -1.92774 1.88088 1.94657
H -4.38668 1.76750 2.48113
H -5.83607 0.14669 1.17312
H -4.76894 -1.26303 -0.59732
H   1.24940 -1.42627 -2.31537
H   0.20919 -3.01919 -3.91323
H -2.25300 -3.46872 -3.82965
H   0.07565 5.03045 -1.02076
H   0.68564 5.04166 0.67692
H -1.06418 4.95409 0.34886

42
12a radical
SCF(BP86)= -1066.03528245
G(298 K) = -1065.773024
SCF(PBE) = -3625.53244968
Low Freq. = 18.5179cm-1,
33.7463cm-1
C -2.54502 -2.06964 -2.28594
C -1.99516 -1.18717 -1.33297
C -0.57939 -0.93071 -1.30961
C   0.23514 -1.59134 -2.26297
C -0.32505 -2.46368 -3.20936
C -1.71368 -2.70458 -3.21980
C -2.78310 -0.47228 -0.32510
C -4.16844 -0.59448 -0.10991
C -4.78895 0.15585 0.89513
C -4.00744 1.02792 1.67253
C -2.63681 1.11802 1.41477
N -2.02465 0.38700 0.44592
Ru 0.00008 0.44834 -0.00036
Br 0.00058 3.05953 -0.00042
H -4.75337 -1.28134 -0.72693
H -5.86443 0.06195 1.07041
H -4.44665 1.63476 2.46826
H -1.98583 1.78917 1.98073
H -2.14716 -3.38681 -3.95774
H   0.32102 -2.96434 -3.93879
H   1.31633 -1.42150 -2.26364
N   2.02492 0.38607 -0.44639
C   2.63754 1.11652 -1.41533
H   1.98686 1.78738 -1.98200
C   4.00830 1.02623 -1.67244
H   4.44788 1.63268 -2.46826
C   4.78938 0.15451 -0.89422
C   4.16835 -0.59527 0.11092
H   5.86494 0.06045 -1.06896
H   4.75300 -1.28182 0.72854
C   2.78292 -0.47287 0.32542
C   1.99452 -1.18717 1.33338
C   0.57882 -0.93032 1.30975
C -0.23598 -1.59035 2.26327
H -1.31712 -1.42013 2.26384
C   0.32383 -2.46256 3.21000
H -0.32248 -2.96272 3.93955
C   1.71237 -2.70392 3.22066
H   2.14555 -3.38608 3.95884
C   2.54400 -2.06954 2.28667
H   3.62209 -2.26150 2.31019
H -3.62320 -2.26118 -2.30932

48
11a
SCF(BP86)= -1198.81016565
G(298 K) = -1198.506761
SCF(PBE) = -3758.19846447
Low Freq. = 27.0555cm-1,
31.1145cm-1
C   2.67819 -2.03232 2.32096
C   2.06917 -1.24074 1.32186
C   0.69856 -0.83474 1.43906
C -0.01570 -1.25619 2.58653
C   0.59929 -2.03159 3.58277

```
C   1.94626 -2.42461  3.44907
C   2.76147 -0.77837  0.11619
C   4.09875 -1.06436 -0.22564
C   4.65081 -0.54126 -1.39804
C   3.85195  0.27330 -2.21887
C   2.53205  0.52667 -1.83784
N   1.98356  0.01249 -0.70624
Ru  0.01370  0.34664 -0.05369
Br -0.70194  1.93615 -2.12170
H   4.69972 -1.69789  0.43148
H   5.68741 -0.76335 -1.66788
H   4.23770  0.70746 -3.14479
H   1.86085  1.15054 -2.43579
H   2.42154 -3.03577  4.22285
H   0.02732 -2.33786  4.46599
H  -1.06550 -0.96851  2.70797
N  -1.93094  0.49207  0.64395
C  -2.39893  1.49584  1.43433
H  -1.66863  2.26075  1.70740
C  -3.72035  1.55476  1.87810
H  -4.03812  2.38346  2.51580
C  -4.61276  0.53877  1.48866
C  -4.14636 -0.48976  0.66723
H  -5.65524  0.55324  1.81955
H  -4.82047 -1.28822  0.34702
C  -2.80118 -0.50638  0.24377
C  -2.18531 -1.50664 -0.62563
C  -0.78889 -1.30021 -0.89338
C  -0.14099 -2.24922 -1.72363
H   0.92550 -2.13373 -1.94655
C  -0.83900 -3.33238 -2.27955
H  -0.31527 -4.04542 -2.92656
C  -2.21167 -3.51253 -2.00742
H  -2.75228 -4.36080 -2.43928
C  -2.88270 -2.60307 -1.18134
H  -3.94882 -2.74974 -0.97571
H   3.72473 -2.34200  2.22653
N   0.76895  2.11963  0.94339
C   1.23137  3.05301  1.48218
C   1.80103  4.21680  2.14573
H   1.74365  4.09045  3.23893
H   2.85679  4.33902  1.85495
H   1.24284  5.12332  1.86134

49
11a OAc
SCF(BP86)= -1294.68233152
G(298 K) = -1294.377223
SCF(PBE) = -3854.03006020
Low Freq. = 18.5175cm-1,
33.2964cm-1
C  -2.96560 -2.80778 -0.53409
C  -2.25040 -1.62172 -0.24943
C  -0.86565 -1.48289 -0.59999
C  -0.25100 -2.59095 -1.23626
C  -0.96663 -3.76322 -1.53029
C  -2.32646 -3.87642 -1.17472
C  -2.84705 -0.45176  0.39817
N  -1.97313  0.60884  0.54096
C  -2.42354  1.76694  1.09306
C  -3.73490  1.92611  1.54560
C  -4.63223  0.84955  1.42182
C  -4.18087 -0.33948  0.84252
Ru -0.02321  0.31318 -0.14020
O   0.40980  2.29764  0.58326
C   1.54557  2.80965  1.00572
C   1.41044  4.27135  1.46301
H  -4.02346 -2.90062 -0.26259
C   0.68343 -0.60862  1.49939
C   2.02131 -1.11581  1.40233
C   2.63542 -1.76545  2.49748
C   1.94179 -1.91626  3.70414
C   0.62615 -1.42014  3.82044
C   0.00633 -0.78591  2.73170
H   3.65975 -2.14672  2.41497
C   2.66847 -0.89031  0.11500
C   3.96225 -1.31722 -0.24660
C   4.47648 -1.01643 -1.51014
C   3.67651 -0.28355 -2.40657
C   2.39956  0.11180 -2.00568
N   1.88581 -0.17868 -0.77857
O   2.64731  2.22883  1.05306
H   4.55974 -1.88605  0.47098
H   5.48051 -1.34597 -1.79390
H   4.03111 -0.02057 -3.40688
H   1.72979  0.67420 -2.66338
H   2.42033 -2.41603  4.55271
H   0.08297 -1.53479  4.76600
H  -1.01967 -0.41709  2.84200
H  -1.66899  2.55647  1.15996
H  -4.04119  2.87797  1.98784
H  -5.66610  0.93853  1.76880
H  -4.85918 -1.18935  0.72942
H   0.80721 -2.53235 -1.51639
H  -0.46495 -4.59777 -2.03459
H  -2.88173 -4.79359 -1.39697
H   2.37970  4.66952  1.79750
H   0.68230  4.34466  2.28895
H   1.02554  4.89519  0.63840
Br -0.82040  1.42094 -2.56803

58
13aMeCN
SCF(BP86)= -1416.81624228
G(298 K) = -1416.425353
SCF(PBE) = -1415.65390622
Low Freq. = 30.0815cm-1,
37.9963cm-1
N  -2.00761 -0.68947  0.13927
C  -2.81058  0.16896 -0.57616
C  -4.16167 -0.15008 -0.81878
C  -4.70535 -1.32446 -0.29256
C  -3.88984 -2.16369  0.48555
C  -2.55090 -1.81389  0.67345
C  -2.13693  1.40608 -0.96655
C  -0.77861  1.52128 -0.56308
C  -0.08225  2.71781 -0.81328
C  -0.72860  3.78912 -1.45531
C  -2.06622  3.66707 -1.87739
C  -2.76956  2.48321 -1.63087
Ru -0.03359 -0.04207  0.51593
C   0.49851 -1.95862  0.02135
```

```
C   0.92902 -0.12905 -1.34402
C   2.29616  0.27227 -1.32701
C   3.03786  0.34624 -2.53001
C   2.43298  0.03819 -3.75261
C   1.07294 -0.32608 -3.78131
C   0.33401 -0.40835 -2.59041
C   2.82858  0.66878 -0.02553
N   1.88479  0.68052  0.98009
C   2.25335  1.07160  2.23056
C   3.55506  1.46380  2.54467
C   4.52804  1.44829  1.53022
C   4.15908  1.04855  0.24367
N  -0.89229  0.86930  2.22040
H   4.08588  0.66283 -2.51172
H  -3.81590  2.40064 -1.94265
H  -4.78121  0.53276 -1.40495
H  -5.75433 -1.57604 -0.47310
H  -4.27541 -3.08035  0.93792
H  -1.87515 -2.43958  1.25945
H  -2.55894  4.49756 -2.39195
H  -0.17843  4.71859 -1.63723
H   0.96493  2.82239 -0.51544
H   1.46537  1.06025  2.98592
H   3.79371  1.76996  3.56584
H   5.55997  1.74244  1.74164
H   4.89952  1.02386 -0.55927
H  -0.72363 -0.68671 -2.64122
H   0.58479 -0.54877 -4.73625
H   3.01070  0.09517 -4.68000
C  -0.10938 -2.92465 -0.80778
C   0.13500 -4.28968 -0.59405
C   0.99815 -4.71299  0.43454
C   1.63329 -3.75568  1.23549
C   1.42228 -2.37728  1.00984
H  -0.79057 -2.62218 -1.60664
H  -0.35918 -5.02676 -1.23607
H   1.18762 -5.77921  0.59253
H   2.33326 -4.06170  2.02037
H   1.97698 -1.66093  1.62180
C  -1.43832  1.39586  3.11344
C  -2.10658  2.05001  4.22466
H  -1.36857  2.56568  4.85962
H  -2.83293  2.78421  3.84009
H  -2.64185  1.29891  4.82775

59
13a OAc
SCF(BP86)= -1512.70426563
G(298 K) = -1512.312029
SCF(PBE) = -1511.50001005
Low Freq. = 28.0083cm-1,
29.9202cm-1
N   1.94820  0.48274 -0.31339
C   2.64837 -0.61749 -0.75698
C   3.96673 -0.48594 -1.23585
C   4.59572  0.76163 -1.21640
C   3.89330  1.86654 -0.70470
C   2.57771  1.68671 -0.27171
C   1.90909 -1.86537 -0.60284
C   0.60006 -1.72914 -0.06162
C  -0.14224 -2.88926  0.23785
C   0.40998 -4.16194  0.01123
C   1.69504 -4.29392 -0.55081
C   2.44285 -3.15050 -0.85352
Ru  0.00685  0.15804  0.39940
C  -0.46904  1.82817 -0.68515
C  -1.10199 -0.43086 -1.28476
C  -2.48415 -0.68083 -1.03298
C  -3.34779 -1.10791 -2.07151
C  -2.85310 -1.30423 -3.36403
C  -1.48376 -1.08969 -3.62040
C  -0.62713 -0.66336 -2.59351
C  -2.91425 -0.52379  0.35500
N  -1.89112 -0.20331  1.22236
C  -2.15614 -0.07785  2.55111
C  -3.43448 -0.25440  3.08130
C  -4.49168 -0.56777  2.20755
C  -4.22602 -0.70291  0.84326
O   0.71106  0.37708  2.33276
C   1.90369  0.17681  2.88789
O   2.68512 -0.73365  2.58073
C   2.21575  1.17657  4.00186
H  -4.40700 -1.29757 -1.86631
H   3.45222 -3.25783 -1.26513
H   4.49684 -1.36772 -1.60447
H   5.62132  0.87022 -1.58095
H   4.34812  2.85845 -0.64596
H   1.98784  2.51903  0.11704
H   2.11275 -5.28708 -0.74304
H  -0.17310 -5.05510  0.26163
H  -1.15442 -2.80665  0.64504
H  -1.28909  0.17000  3.16963
H  -3.59301 -0.14436  4.15691
H  -5.50827 -0.70545  2.58756
H  -5.03180 -0.94848  0.14724
H   0.43572 -0.51838 -2.81803
H  -1.08295 -1.25829 -4.62645
H  -3.52233 -1.63391 -4.16484
H   3.16858  0.92547  4.48929
H   1.40661  1.18205  4.75069
H   2.27705  2.19551  3.58296
C   0.03787  2.34538 -1.89624
C  -0.17201  3.68977 -2.24410
C  -0.89876  4.54465 -1.39514
C  -1.42805  4.03487 -0.20093
C  -1.24531  2.67830  0.14315
H   0.60946  1.70217 -2.57084
H   0.23880  4.06973 -3.18631
H  -1.06300  5.59116 -1.67119
H  -2.01594  4.67709  0.46443
H  -1.70673  2.30953  1.06475

51
2a PhBr
I-MeCN_BrPh_trans-N_2
SCF(BP86)= -1217.06857014
G(298 K) = -1216.743353
SCF(PBE) = -3776.44240674
Low Freq. = 11.9746cm-1,
16.5342cm-1
N   1.40092 -1.47789  0.39313
C   2.65569 -1.60260 -0.16652
```

```
C   3.37153 -2.80954 -0.03289
C   2.81436 -3.88447  0.66545
C   1.53506 -3.74203  1.22799
C   0.86608 -2.52578  1.06801
C   3.11675 -0.40357 -0.87250
C   4.36900 -0.30914 -1.52259
C   4.73305  0.87185 -2.18046
C   3.84416  1.96377 -2.18967
C   2.59720  1.87760 -1.54401
C   2.20531  0.69929 -0.87363
H   5.06276 -1.15719 -1.51716
Ru  0.47516  0.37599  0.10387
N  -0.37355 -0.32305 -1.55855
C  -0.87423 -0.74266 -2.53327
C  -1.50870 -1.26560 -3.73441
N  -0.29988  2.19070 -0.24404
C  -0.76481  3.25200 -0.43612
C  -1.32669  4.57305 -0.68236
N   1.29198  1.04076  1.80291
C   1.77742  1.42647  2.79933
C   2.36799  1.90272  4.04178
H   1.65391  2.55068  4.57581
H   2.62773  1.04973  4.68978
H   3.28270  2.48036  3.83065
H  -1.74678  4.98819  0.24836
H  -0.54615  5.25673 -1.05500
H  -2.12971  4.50895 -1.43468
H   4.36472 -2.89909 -0.47958
H   3.36936 -4.82135  0.76976
H   1.05647 -4.55284  1.78237
H  -0.13057 -2.36370  1.48530
H   5.70261  0.94292 -2.68326
H   4.12420  2.89066 -2.70346
H   1.92473  2.74121 -1.56540
H  -2.54786 -1.55954 -3.51292
H  -1.51598 -0.49843 -4.52581
H  -0.95878 -2.14814 -4.09975
Br -1.85544 -0.12953  1.64518
C  -3.36912 -0.40894  0.46115
C  -3.58527 -1.68725 -0.07166
C  -4.20667  0.67867  0.17806
C  -4.68878 -1.87372 -0.92399
C  -5.30423  0.46800 -0.67595
C  -5.54508 -0.80163 -1.22661
H  -2.91734 -2.51790  0.16919
H  -4.01301  1.66362  0.61015
H  -4.87460 -2.86627 -1.34604
H  -5.96985  1.30582 -0.90554
H  -6.40177 -0.95671 -1.88918
```

```
51
2a PhBr pi
I-MeCN_eta2-BrPh_trans-C_2
SCF(BP86)= -1217.06237015
G(298 K) = -1216.737250
SCF(PBE) = -3776.43992813
Low Freq. = 18.4922cm-1,
24.9209cm-1
N   2.17216 -1.32153  0.16045
C   3.41212 -0.75950  0.38392
C   4.53734 -1.58448  0.58413
C   4.40340 -2.97540  0.55841
C   3.13455 -3.53370  0.33087
C   2.05213 -2.67203  0.13789
C   3.40892  0.70567  0.38643
C   4.56360  1.49459  0.59538
C   4.47373  2.89152  0.58207
C   3.22629  3.50524  0.35943
C   2.07185  2.72877  0.15085
C   2.13730  1.31956  0.15962
H   5.53554  1.01982  0.76897
Ru  0.61341  0.04722 -0.11788
N   0.98398  0.04361 -2.08062
C   1.23891  0.05663 -3.22580
C   1.54493  0.06391 -4.64908
N  -0.79839  1.44184 -0.36627
C  -1.63988  2.24788 -0.51125
C  -2.67129  3.26087 -0.68647
N   0.27656  0.03683  1.85057
C   0.11545  0.04968  3.01267
C  -0.09617  0.05667  4.45301
H  -1.17493  0.08785  4.67710
H   0.33472 -0.85206  4.90406
H   0.38570  0.93985  4.90324
H  -3.65311  2.86052 -0.38405
H  -2.44351  4.14666 -0.07085
H  -2.72409  3.56857 -1.74358
H   5.51496 -1.12915  0.75962
H   5.27591 -3.61655  0.71364
H   2.97695 -4.61433  0.30173
H   1.04866 -3.06365 -0.04236
H   5.36890  3.49996  0.74380
H   3.15038  4.59858  0.34795
H   1.11409  3.22973 -0.01993
H   0.85108 -0.59933 -5.19100
H   1.44928  1.08512 -5.05255
H   2.57595 -0.28831 -4.81550
Br -5.29399  0.01964  0.54950
C  -3.67332 -0.76815 -0.14311
C  -2.77539 -1.36779  0.74453
C  -3.43963 -0.73059 -1.53063
C  -1.59864 -1.94924  0.22574
C  -2.26855 -1.31254 -2.03119
C  -1.34120 -1.92250 -1.15988
H  -2.98279 -1.39906  1.81667
H  -4.16409 -0.26532 -2.20369
H  -0.92754 -2.47385  0.91202
H  -2.08273 -1.30235 -3.10926
H  -0.46860 -2.43363 -1.57574
```

```
51
2b PhBr
SCF(BP86)= -1217.05964696
G(298 K) = -1216.734579
SCF(PBE) = -3776.43348406
Low Freq. = 10.8160cm-1,
18.9045cm-1
C  -5.84495  0.24565 -0.08045
C  -5.24488 -0.29580 -1.22967
C  -3.95454 -0.85229 -1.16444
C  -3.29713 -0.85053  0.07246
C  -3.86945 -0.32092  1.23602
```

```
C  -5.15978  0.23253  1.14663
Br -1.51378 -1.64620  0.18003
Ru  0.37344  0.12782  0.00731
N   0.43095  0.24769  1.99928
C   0.48082  0.32031  3.16922
C   0.53849  0.41105  4.62146
C   1.84901 -1.29233  0.05963
C   3.17800 -0.75948 -0.00530
C   4.31680 -1.59707  0.02078
C   4.16401 -2.98565  0.11141
C   2.86982 -3.53498  0.17529
C   1.73849 -2.69867  0.14922
C   3.25807  0.69999 -0.10186
C   4.43930  1.46577 -0.18089
C   4.37501  2.85853 -0.27174
C   3.11637  3.48311 -0.28305
C   1.97205  2.68709 -0.20249
N   2.03173  1.33340 -0.11415
N   0.32216 -0.01311 -1.98343
C   0.30656 -0.09332 -3.15380
C   0.28202 -0.19255 -4.60642
N  -1.06520  1.70071 -0.05720
C  -1.89354  2.53143 -0.09210
C  -2.92982  3.55361 -0.13575
H   5.40740  0.95912 -0.17077
H  -0.47803  0.50747  5.03639
H   1.00914 -0.49323  5.04057
H   1.12950  1.29127  4.92300
H  -3.91282  3.09724  0.06532
H  -2.73267  4.32708  0.62401
H  -2.95228  4.02801 -1.13013
H   5.32470 -1.17023 -0.02973
H   5.04441 -3.63568  0.13180
H   2.74030 -4.62116  0.24586
H   0.74988 -3.17388  0.20022
H   5.29284  3.45035 -0.33311
H   3.01253  4.56852 -0.35263
H   0.96937  3.11791 -0.20635
H  -0.60386 -0.76163 -4.93236
H   0.24323  0.81309 -5.05605
H   1.18691 -0.70857 -4.96655
H  -3.47957 -1.27417 -2.05392
H  -3.32985 -0.33713  2.18631
H  -5.77665 -0.28986 -2.18616
H  -5.62598  0.64927  2.04482
H  -6.84937  0.67535 -0.13998
```

```
51
2c PhBr
SCF(BP86)= -1217.05766252
G(298 K) = -1216.732583
SCF(PBE) = -3776.43211063
Low Freq. = 8.1638cm-1, 26.5810cm-
1
C   0.36319 -1.38806  0.88863
C   1.26400 -2.30132  0.24837
C   1.23460 -3.68623  0.53357
C   0.32111 -4.19149  1.46685
C  -0.56303 -3.30979  2.11805
C  -0.53788 -1.93264  1.83211
C   2.20400 -1.69672 -0.70165
N   2.07213 -0.32903 -0.83685
C   2.90060  0.34444 -1.67555
C   3.88954 -0.29153 -2.42917
C   4.03207 -1.68467 -2.31123
C   3.18699 -2.38291 -1.44444
Ru  0.56754  0.55206  0.31364
Br -0.99863  0.18351 -1.72490
N   1.85752  0.84197  1.75980
C   2.63661  1.00693  2.62183
C   3.60504  1.22030  3.68877
N  -0.92773  1.22832  1.46345
C  -1.79955  1.63055  2.13936
C  -2.89191  2.11436  2.97239
N   0.87697  2.54775 -0.40689
C   1.02408  3.65271 -0.77512
C   1.20989  5.02231 -1.23549
H   1.92482 -4.37398  0.03217
H   1.51805  5.66464 -0.39470
H   1.98758  5.05883 -2.01552
H   0.26752  5.41172 -1.65355
H  -3.85684  1.91153  2.47955
H  -2.87985  1.60545  3.95016
H  -2.79848  3.20039  3.13584
H   3.28426 -3.46588 -1.33603
H   4.79399 -2.21842 -2.88682
H   4.52826  0.29854 -3.09071
H   2.74423  1.42416 -1.72810
H   0.29945 -5.26317  1.68887
H  -1.27640 -3.69929  2.85393
H  -1.23790 -1.27143  2.35491
H   3.42317  2.19087  4.17873
H   3.52379  0.42066  4.44313
H   4.62779  1.21721  3.27765
C  -2.81330 -0.22635 -1.14013
C  -3.73060  0.82706 -1.03334
C  -5.04501  0.52021 -0.63689
C  -5.41271 -0.80722 -0.35965
C  -4.46875 -1.84096 -0.47725
C  -3.14806 -1.55953 -0.87172
H  -3.43565  1.85603 -1.25395
H  -5.77872  1.32750 -0.54989
H  -6.43780 -1.03666 -0.05364
H  -4.75277 -2.87615 -0.26431
H  -2.40584 -2.35564 -0.96291
```

```
5a PhBr
SCF(BP86)= -1312.93583190
G(298 K) = -1312.609810
SCF(PBE) = -3872.26790083
Low Freq. = 8.3542cm-1, 19.3217cm-
1
N   1.90841 -1.36189 -0.20492
C   3.20394 -0.90231 -0.34603
C   4.24814 -1.80295 -0.64322
C   3.98110 -3.16597 -0.79854
C   2.65818 -3.61993 -0.65692
C   1.66060 -2.68653 -0.36471
```

C 3.33885 0.54166 -0.15684
C 4.57528 1.22789 -0.21134
C 4.62691 2.60749 0.01434
C 3.43524 3.30460 0.30560
C 2.20425 2.63188 0.35809
C 2.11328 1.23918 0.11265
H 5.50176 0.68220 -0.42469
Ru 0.47472 0.10862 0.16545
N 0.68817 -0.08184 2.11654
C 0.81531 -0.19889 3.28223
C 0.95932 -0.36654 4.72361
N -0.81617 1.59452 0.44222
C -1.60649 2.44425 0.63475
C -2.58144 3.50861 0.83518
H -2.54152 4.22556 -0.00198
H -3.59936 3.08773 0.89030
H -2.37387 4.05126 1.77236
H 5.26800 -1.42572 -0.75510
H 4.79041 -3.86509 -1.02941
H 2.39569 -4.67457 -0.77118
H 0.61683 -2.99397 -0.25826
H 5.58401 3.13724 -0.02696
H 3.46948 4.38450 0.49387
H 1.29454 3.19859 0.58148
H 1.87017 -0.94451 4.95267
H 1.03375 0.61439 5.22204
H 0.09044 -0.90499 5.13805
Br -1.77186 -1.72116 0.46553
O 0.11769 -0.02017 -1.88380
C -0.04989 0.93130 -2.77536
O 0.04055 2.15977 -2.59041
C -0.38276 0.36960 -4.16790
H -1.30738 -0.23080 -4.12943
H 0.42308 -0.29970 -4.51382
H -0.51137 1.18680 -4.89259
C -3.46757 -0.85288 0.09363
C -3.94865 -0.83903 -1.22321
C -4.16558 -0.25798 1.15447
C -5.17684 -0.20291 -1.47833
C -5.39209 0.37235 0.87640
C -5.89685 0.40192 -0.43465
H -3.38317 -1.31035 -2.03096
H -3.76754 -0.28491 2.17209
H -5.56615 -0.18519 -2.50110
H -5.95061 0.83766 1.69474
H -6.85180 0.89403 -0.64214

45
5a PhBr-OAc
SCF(BP86)= -1084.26051565
G(298 K) = -1083.977050
SCF(PBE) = -3643.74772063
Low Freq. = 7.4534cm-1, 25.2945cm-1
N -2.07261 -1.17488 -0.40223
C -2.99391 -0.44076 -1.11999
C -4.12327 -1.07053 -1.67854
C -4.33499 -2.43788 -1.47870
C -3.41486 -3.16398 -0.70493
C -2.30228 -2.49561 -0.18842
C -2.69278 0.99227 -1.17789
C -3.45599 1.92634 -1.91325
C -3.13399 3.28864 -1.86864
C -2.05075 3.72431 -1.08235
C -1.27884 2.80194 -0.35347
C -1.56188 1.41894 -0.40946
H -4.30253 1.59282 -2.52302
Ru -0.44536 -0.07015 0.32999
N -1.34097 0.00395 2.01858
C -1.89709 0.06253 3.05161
C -2.56764 0.11189 4.34293
N 0.98738 1.13533 1.01718
C 1.81399 1.84394 1.45729
C 2.82773 2.73854 1.99629
H 3.63097 2.88730 1.25643
H 2.38223 3.71737 2.23845
H 3.26525 2.30937 2.91248
H -4.83961 -0.48048 -2.25506
H -5.21289 -2.92841 -1.90865
H -3.54690 -4.22909 -0.50156
H -1.56273 -3.02350 0.41818
H -3.72830 4.00962 -2.43824
H -1.80431 4.79094 -1.03493
H -0.44266 3.16570 0.25065
H -2.33024 -0.78926 4.93226
H -2.23947 1.00119 4.90572
H -3.65938 0.16653 4.20101
Br 4.60770 -0.02272 -1.38916
C 3.17736 -0.88215 -0.42982
C 2.03084 -1.27341 -1.13019
C 3.32896 -1.12146 0.94899
C 0.97806 -1.92175 -0.42175
C 2.29024 -1.76241 1.64110
C 1.11522 -2.16584 0.97516
H 1.95433 -1.12506 -2.21000
H 4.24417 -0.82599 1.46731
H 0.19854 -2.42217 -1.00391
H 2.40837 -1.97006 2.70900
H 0.37022 -2.75753 1.51451

```
52
5b PhBr
SCF(BP86)= -1312.93519107
G(298 K) = -1312.608800
SCF(PBE) = -3872.26730638
Low Freq. = 12.1138cm-1,
16.1871cm-1
N   1.08214  1.67272  0.16051
C   2.32906  1.98151 -0.35455
C   2.75829  3.32221 -0.42218
C   1.92799  4.35362  0.02788
C   0.66245  4.02732  0.54433
C   0.28282  2.68295  0.59089
C   3.09351  0.80977 -0.79128
C   4.39701  0.87204 -1.33670
C   5.05437 -0.30353 -1.72058
C   4.40866 -1.54638 -1.55793
C   3.11272 -1.61524 -1.01672
C   2.42346 -0.44518 -0.62327
H   4.90285  1.83665 -1.46151
Ru  0.59154 -0.34718  0.16641
N  -0.20448 -0.10725 -1.62958
C  -0.65130  0.10932 -2.69474
C  -1.25140  0.33757 -4.00233
N   1.35586 -0.56306  1.99294
C   1.81584 -0.69249  3.06779
C   2.36906 -0.84697  4.40687
H   2.58665  0.13983  4.84805
H   1.65077 -1.37304  5.05732
H   3.30358 -1.43085  4.37083
H   3.74627  3.54958 -0.83149
H   2.26054  5.39459 -0.02447
H  -0.02562  4.79506  0.90742
H  -0.68933  2.37668  0.98550
H   6.06336 -0.25505 -2.14283
H   4.92231 -2.46824 -1.85668
H   2.62135 -2.58624 -0.89098
H  -2.34024  0.47813 -3.89647
H  -0.82456  1.23829 -4.47322
H  -1.07065 -0.52529 -4.66488
Br -1.87641 -0.14691  1.57583
O   0.34973 -2.45452  0.21979
C  -0.55654 -3.18532 -0.37279
O  -1.50757 -2.78680 -1.08435
C  -0.34244 -4.69317 -0.15417
H  -0.07480 -4.91038  0.89260
H   0.49412 -5.04324 -0.78467
H  -1.24580 -5.25678 -0.43108
C  -3.37804  0.33864  0.44093
C  -3.60388 -0.41909 -0.71549
C  -4.18166  1.42509  0.81308
C  -4.69686 -0.06215 -1.52746
C  -5.26759  1.75942 -0.01667
C  -5.52540  1.01991 -1.18318
H  -2.93310 -1.25329 -0.96178
H  -3.97344  1.99678  1.72167
H  -4.89837 -0.64101 -2.43510
H  -5.90937  2.60288  0.25728
H  -6.37232  1.28705 -1.82287
```

```
45
5b PhBr-OAc
SCF(BP86)= -1084.26559131
G(298 K) = -1083.982291
SCF(PBE) = -3643.75271519
Low Freq. = 15.4010cm-1,
26.3743cm-1
N  -1.23826 -0.50353  1.26734
C  -2.38754 -1.25858  1.10034
C  -2.86366 -2.04953  2.16333
C  -2.18518 -2.07523  3.38488
C  -1.02247 -1.30098  3.53400
C  -0.58188 -0.53163  2.45507
C  -2.98348 -1.12433 -0.22546
C  -4.16220 -1.76825 -0.66361
C  -4.63051 -1.54491 -1.96488
C  -3.92657 -0.67991 -2.82694
C  -2.75203 -0.03459 -2.39467
C  -2.26482 -0.25041 -1.09173
H  -4.71386 -2.43888  0.00456
Ru -0.61895  0.58033 -0.32331
N  -1.70815  2.13534  0.28398
C  -2.37283  3.03572  0.64086
C  -3.19405  4.15591  1.07399
N   0.46262 -0.96352 -0.94594
C   1.07030 -1.90261 -1.30326
C   1.83625 -3.05748 -1.74711
H   1.77098 -3.15452 -2.84335
H   2.89452 -2.94014 -1.46201
H   1.43918 -3.97622 -1.28506
H  -3.77011 -2.64273  2.01882
H  -2.55565 -2.69006  4.20991
H  -0.45468 -1.28649  4.46711
H   0.31717  0.08199  2.51467
H  -5.54275 -2.04116 -2.30995
H  -4.29605 -0.50633 -3.84386
H  -2.21808  0.63692 -3.07887
H  -3.30181  4.88350  0.25222
H  -4.19492  3.80020  1.36866
H  -2.72610  4.66111  1.93447
Br  4.92794 -0.58046  0.21693
C   3.41266  0.57234 -0.07973
C   2.63936  0.97187  1.01649
C   3.13291  1.00712 -1.39028
C   1.53818  1.82977  0.80298
C   2.04727  1.86477 -1.59834
C   1.22990  2.27553 -0.51122
H   2.90314  0.65050  2.02733
H   3.76343  0.69339 -2.22589
H   1.00149  2.24102  1.66325
H   1.84139  2.23945 -2.60548
H   0.50736  3.08449 -0.65927
```

```
52
5c PhBr
SCF(BP86)= -1312.92736566
G(298 K) = -1312.600985
SCF(PBE) = -3872.26112924
Low Freq. = 19.2097cm-1,
24.7456cm-1
C   0.97313  1.68150  0.22920
C   2.24180  2.00133 -0.36899
C   2.71776  3.33066 -0.44755
C   1.94762  4.38234  0.06398
C   0.69894  4.09922  0.65280
C   0.22671  2.77657  0.73101
C   2.98962  0.84888 -0.87868
C   4.25591  0.87191 -1.49950
C   4.85511 -0.31713 -1.92613
C   4.18076 -1.53484 -1.72603
C   2.92673 -1.51416 -1.11038
N   2.34361 -0.35901 -0.70147
H   4.76601  1.82810 -1.64261
Ru  0.51746 -0.29822  0.19617
N  -0.34013 -0.03960 -1.57106
C  -0.80974  0.15483 -2.63016
C  -1.43248  0.36029 -3.93076
N   1.39487 -0.57889  1.96275
C   1.93624 -0.76211  2.99018
C   2.59629 -0.98562  4.26976
H   2.61713 -0.05422  4.85942
H   2.05938 -1.75700  4.84616
H   3.63298 -1.32488  4.10957
H   3.68847  3.54981 -0.90811
H   2.31302  5.41272  0.00428
H   0.08936  4.91862  1.05324
H  -0.75049  2.59471  1.19480
H   5.83736 -0.29628 -2.40774
H   4.61262 -2.48837 -2.04048
H   2.33580 -2.41377 -0.91250
H  -2.52502  0.45077 -3.81010
H  -1.05251  1.28179 -4.40146
H  -1.22073 -0.49125 -4.59866
Br -1.71304 -0.16455  1.56666
O   0.35742 -2.53309  0.15025
C  -0.57545 -3.29865 -0.33303
O  -1.63881 -2.93957 -0.89814
C  -0.28088 -4.80368 -0.17160
H  -0.13123 -5.05243  0.89307
H   0.65287 -5.06797 -0.69745
H  -1.10446 -5.41314 -0.57278
C  -3.23336  0.35992  0.46355
C  -3.56968 -0.45234 -0.62596
C  -3.94145  1.51807  0.80952
C  -4.68165 -0.07227 -1.40088
C  -5.05038  1.87247  0.01868
C  -5.41904  1.08183 -1.08255
H  -2.96381 -1.34208 -0.84852
H  -3.64530  2.12873  1.66687
H  -4.97179 -0.69019 -2.25734
H  -5.62149  2.77159  0.27089
H  -6.28259  1.36518 -1.69235
```

```
52
5d PhBr
SCF(BP86)= -1312.93290826
G(298 K) = -1312.606201
SCF(PBE) = -3872.26284256
Low Freq. = 17.6131cm-1,
26.0138cm-1
N  -2.13866  0.63146  1.05482
C  -3.06757 -0.39296  1.00749
C  -4.31236 -0.23983  1.65198
C  -4.61120  0.93386  2.34983
C  -3.64875  1.95695  2.40028
C  -2.43228  1.76702  1.74060
C  -2.61004 -1.57639  0.27668
C  -3.38580 -2.75097  0.13922
C  -2.86249 -3.86138 -0.53409
C  -1.55720 -3.79480 -1.06113
C  -0.78933 -2.62437 -0.92701
C  -1.28563 -1.47011 -0.27046
H  -4.39710 -2.80522  0.55938
Ru -0.41147  0.34129  0.01223
N   0.51738 -0.32193  1.61254
C   1.06225 -0.72244  2.57707
C   1.76341 -1.21945  3.75462
N   0.36012  2.29786  0.29665
C   0.79200  3.38524  0.41966
C   1.32038  4.73520  0.57605
H   1.64771  5.13159 -0.39907
H   0.54693  5.40437  0.98759
H   2.18340  4.72926  1.26176
H  -5.04298 -1.05146  1.60233
H  -5.57796  1.04956  2.84874
H  -3.83023  2.89221  2.93586
H  -1.65313  2.53169  1.73870
H  -3.46051 -4.77207 -0.64321
H  -1.13719 -4.66356 -1.58289
H   0.21943 -2.60778 -1.35614
H   1.84531 -0.43126  4.52176
H   1.22535 -2.07780  4.19048
H   2.77963 -1.54720  3.47688
Br  1.66951 -0.12531 -1.53841
O  -1.51411  1.13531 -1.60700
C  -1.22320  0.97517 -2.86552
O  -0.23350  0.35104 -3.32942
C  -2.18980  1.66377 -3.83427
H  -3.14984  1.90522 -3.35492
H  -2.36110  1.02426 -4.71427
H  -1.73313  2.60274 -4.19364
C   3.20171 -0.45186 -0.36206
C   3.44517 -1.75422  0.10019
C   4.04839  0.61640 -0.02926
C   4.56515 -1.98609  0.91944
C   5.16483  0.36918  0.79022
C   5.42338 -0.92779  1.26511
H   2.77446 -2.57438 -0.17165
H   3.84314  1.62412 -0.40188
H   4.76515 -2.99954  1.28238
H   5.83272  1.19595  1.05297
H   6.29468 -1.11488  1.90039
```

52
5e PhBr
SCF(BP86)= -1312.92417459
G(298 K) = -1312.597903
SCF(PBE) = -3872.25730062
Low Freq. = 15.9895cm-1,
21.2746cm-1

```
N   1.38835  1.24390  0.99706
C   2.49567  1.67330  0.28487
C   3.23524  2.78696  0.73237
C   2.86082  3.46640  1.89576
C   1.73592  3.01800  2.60918
C   1.03492  1.90921  2.12830
C   2.77787  0.88188 -0.91963
C   3.84302  1.16095 -1.80782
C   4.03677  0.36187 -2.94201
C   3.16381 -0.71724 -3.19037
C   2.10407 -0.99433 -2.30848
C   1.87681 -0.20978 -1.15280
H   4.52254  2.00104 -1.62195
Ru  0.40921 -0.42373  0.24456
N   1.60883 -1.54329  1.27862
C   2.38570 -2.16542  1.90536
C   3.31103 -2.99112  2.67165
N  -1.09505 -0.48162  1.71040
C  -1.95147 -0.48710  2.51712
C  -3.02832 -0.50192  3.49950
H  -3.04755 -1.46853  4.02937
H  -2.89231  0.30388  4.23952
H  -3.99795 -0.35853  2.99453
H   4.10726  3.11578  0.16094
H   3.43640  4.33012  2.24162
H   1.40132  3.51220  3.52497
H   0.15542  1.51872  2.64549
H   4.86081  0.57736 -3.63020
H   3.31432 -1.34333 -4.07845
H   1.42945 -1.83395 -2.51417
H   2.93581 -4.02659  2.73423
H   4.30197 -3.00704  2.18837
H   3.42795 -2.59785  3.69523
Br -0.95143  1.13257 -1.32658
O  -0.50619 -2.00127 -0.84046
C  -0.77865 -3.20902 -0.41041
O  -0.56751 -3.67005  0.73112
C  -1.39153 -4.10283 -1.50393
H  -0.59582 -4.44283 -2.19036
H  -2.13030 -3.55276 -2.10888
H  -1.86442 -4.98993 -1.05645
C  -2.85981  1.08427 -0.92389
C  -3.47045 -0.16306 -0.74319
C  -3.56127  2.29412 -0.84845
C  -4.85067 -0.18920 -0.47484
C  -4.94262  2.24209 -0.58428
C  -5.58527  1.00703 -0.39735
H  -2.87346 -1.07779 -0.80134
H  -3.05273  3.25207 -0.98805
H  -5.34912 -1.15294 -0.32937
H  -5.51029  3.17590 -0.52244
H  -6.65946  0.97634 -0.19096
```

52
5f PhBr
SCF(BP86)= -1312.92534728
G(298 K) = -1312.598851
SCF(PBE) = -3872.25737292
Low Freq. = 11.5063cm-1,
21.2705cm-1

```
N   0.80969  1.38365  1.03250
C   1.85040  2.12630  0.50550
C   2.17401  3.38237  1.05945
C   1.44916  3.88727  2.14312
C   0.39460  3.12106  2.66865
C   0.11308  1.88244  2.08672
C   2.52380  1.49194 -0.63337
C   3.60005  2.09093 -1.32827
C   4.18686  1.43559 -2.41827
C   3.69234  0.17700 -2.81485
C   2.62256 -0.41911 -2.12383
C   2.00348  0.20756 -1.01323
H   3.98495  3.07126 -1.02353
Ru  0.47343 -0.46938  0.14944
N   0.31151 -2.19053 -0.82194
C   0.20370 -3.18509 -1.44214
C   0.09629 -4.43618 -2.18326
N  -1.10841 -0.99793  1.43434
C  -2.04190 -1.26599  2.09741
C  -3.20120 -1.60429  2.91386
H  -3.17411 -2.67030  3.19370
H  -3.21310 -0.99803  3.83448
H  -4.12912 -1.40973  2.35103
H   2.99912  3.95998  0.63467
H   1.70203  4.86156  2.57201
H  -0.20236  3.46840  3.51584
H  -0.69222  1.24614  2.46074
H   5.02012  1.89998 -2.95579
H   4.14636 -0.34132 -3.66838
H   2.26214 -1.40010 -2.45584
H  -0.94318 -4.60438 -2.51075
H   0.74405 -4.41520 -3.07563
H   0.40293 -5.28437 -1.54810
Br -1.05062  0.61017 -1.60941
O   1.94386 -1.14051  1.46657
C   1.94442 -2.31177  2.06664
O   1.09665 -3.21751  1.96351
C   3.19066 -2.50412  2.94800
H   3.51930 -1.55804  3.40528
H   4.02208 -2.88153  2.32673
H   2.98591 -3.24829  3.73251
C  -2.92708  0.71364 -1.06075
C  -3.72320 -0.43618 -1.14937
C  -3.42357  1.94020 -0.59933
C  -5.07127 -0.34509 -0.75787
C  -4.77547  2.01060 -0.21603
C  -5.59625  0.87304 -0.29308
H  -3.30715 -1.37908 -1.51386
H  -2.77724  2.81998 -0.53877
H  -5.70868 -1.23256 -0.82263
H  -5.18112  2.96152  0.14351
H  -6.64651  0.93577  0.00743
```

46
14a
SCF(BP86)= -1180.16320509
G(298 K) = -1179.877365
SCF(PBE) = -3739.60037648
Low Freq. = 12.3535cm-1,
25.6701cm-1
C 2.57050 -3.14031 0.09939
C 2.09047 -1.82130 -0.03141
N 0.98929 -1.41192 0.69830
C 0.38367 -2.28828 1.54194
C 0.82124 -3.60558 1.70202
C 1.93776 -4.03872 0.96475
C 2.64454 -0.77356 -0.89306
C 3.78840 -0.95206 -1.70466
C 4.26154 0.09928 -2.49944
C 3.58559 1.33684 -2.48193
C 2.44774 1.52139 -1.67827
C 1.93992 0.48197 -0.85681
Ru 0.39469 0.54360 0.42428
O -0.70038 0.59968 2.50556
C 0.43124 0.94959 2.98191
C 0.61705 1.26488 4.44850
O 1.44501 1.06413 2.17755
N -0.07555 2.44347 0.13438
C -0.37071 3.57576 -0.00945
C -0.72467 4.97741 -0.19697
H 3.44109 -3.45647 -0.48135
H -0.91275 5.18620 -1.26343
H -1.63398 5.22839 0.37452
H 0.09579 5.62962 0.14653
H 4.31498 -1.91368 -1.71662
H 5.14754 -0.04053 -3.12704
H 3.95027 2.16389 -3.10328
H 1.94192 2.49383 -1.68772
H 2.30985 -5.06272 1.06492
H 0.29628 -4.27046 2.39261
H -0.46662 -1.88527 2.09861
H 1.67934 1.22666 4.72976
H 0.23619 2.28017 4.65521
H 0.03796 0.55901 5.06337
Br -1.02380 -0.11359 -1.54948
C -2.86624 -0.53276 -1.00013
C -3.75871 -0.97899 -1.98266
C -3.22798 -0.36500 0.33971
C -5.07862 -1.26933 -1.59220
C -4.55399 -0.66238 0.70618
C -5.47641 -1.11190 -0.25349
H -3.44359 -1.09989 -3.02339
H -2.49569 -0.01570 1.07631
H -5.79303 -1.62001 -2.34384
H -4.86043 -0.53885 1.75002
H -6.50538 -1.33986 0.04092

46
14a pi
SCF(BP86)= -1180.15886520
G(298 K) = -1179.873264
SCF(PBE) = -3739.59611967
Low Freq. = 13.1857cm-1,
14.7192cm-1
C 4.63199 1.37295 0.30171
C 3.44312 0.63528 0.10055
C 2.15991 1.22162 0.35621
C 2.12759 2.55222 0.83843
C 3.31573 3.27678 1.03983
C 4.56984 2.69230 0.76847
C 3.39870 -0.75794 -0.34341
C 4.50937 -1.56810 -0.64822
C 4.33221 -2.90265 -1.02715
C 3.02583 -3.41813 -1.08380
C 1.95549 -2.57471 -0.77836
N 2.10948 -1.26608 -0.43561
Ru 0.60373 0.07683 -0.05841
O -0.79908 1.73207 -0.15611
C -0.51102 1.91528 -1.39808
C -1.18227 2.99118 -2.21189
O 0.35925 1.12148 -1.93152
N 0.49035 -0.54857 1.78011
C 0.43060 -0.89709 2.90638
C 0.32032 -1.30468 4.30190
H 5.60899 0.91884 0.09893
H 1.30085 -1.23146 4.80152
H -0.39282 -0.65599 4.83854
H -0.03076 -2.34770 4.37685
H 5.51446 -1.14294 -0.58137
H 5.19484 -3.53137 -1.26583
H 2.82877 -4.45770 -1.35785
H 0.92768 -2.94310 -0.80585
H 5.49106 3.26265 0.92495
H 3.26550 4.30801 1.40939
H 1.16196 3.02671 1.04547
H -0.50030 3.36804 -2.98882
H -2.07101 2.57032 -2.71344
H -1.51150 3.81643 -1.56354
H -0.59380 -1.99358 -2.09208
C -1.43967 -1.55476 -1.55649
C -2.39190 -0.79501 -2.26737
C -1.62986 -1.80263 -0.18263
C -3.52090 -0.27862 -1.61803
C -2.76261 -1.28732 0.48528
C -3.68639 -0.53419 -0.24432
H -2.25883 -0.61269 -3.33794
H -0.94354 -2.44800 0.37293
H -4.26445 0.30371 -2.16818
H -2.91617 -1.48637 1.54859
Br -5.24662 0.16797 0.65380

39
14a-OAc
SCF(BP86)= -951.503796506
G(298 K) = -951.259708
SCF(PBE) = -3511.10040240
Low Freq. = 15.6871cm-1,
39.2914cm-1
N -1.33020 1.34699 -0.18178
C -2.61802 0.89781 -0.00155
C -3.66925 1.82422 0.15281
C -3.40302 3.19533 0.13571
C -2.07712 3.63378 -0.02242
C -1.06975 2.67922 -0.17345
C -2.73179 -0.55947 0.04374

C -3.94795 -1.25120 0.24558
C -3.95010 -2.64760 0.34183
C -2.73591 -3.35333 0.25470
C -1.52321 -2.66825 0.05012
C -1.50025 -1.26612 -0.08423
H -4.89112 -0.70180 0.33480
Ru 0.15797 -0.10372 -0.43299
N 0.35055 -0.06735 1.57540
C 0.45984 -0.03894 2.74061
C 0.60598 -0.00993 4.18730
H 1.48192 -0.60747 4.48725
H 0.74410 1.02769 4.53157
H -0.29481 -0.42951 4.66352
H -4.69013 1.46065 0.29160
H -4.21738 3.91593 0.25253
H -1.81825 4.69493 -0.02981
H -0.02271 2.96552 -0.29250
H -4.89121 -3.18429 0.49534
H -2.73003 -4.44506 0.34612
H -0.59281 -3.24428 0.00188
Br 3.87613 0.09754 0.49676
C 2.44839 -0.10953 -0.76201
C 1.96504 1.03048 -1.45121
C 1.90837 -1.42738 -0.97668
C 0.84580 0.84622 -2.33107
C 0.86140 -1.58522 -1.93177
C 0.32602 -0.45259 -2.62882
H 2.38954 2.02008 -1.27431
H 2.29155 -2.28305 -0.41823
H 0.39035 1.72481 -2.79732
H 0.43266 -2.57736 -2.09315
H -0.50626 -0.56976 -3.32494

46
14b
SCF(BP86)= -1180.16394695
G(298 K) = -1179.878103
SCF(PBE) = -3739.60016913
Low Freq. = 14.7501cm-1, 26.8633cm-1
C -4.52902 -0.81248 0.61026
C -3.26762 -0.27627 0.28268
N -2.13038 -1.04310 0.45934
C -2.23517 -2.30319 0.95949
C -3.46464 -2.86824 1.30608
C -4.63442 -2.10928 1.12295
C -2.98850 1.06392 -0.23092
C -3.99479 2.01674 -0.50967
C -3.64939 3.29165 -0.97328
C -2.28873 3.61424 -1.14919
C -1.28580 2.66865 -0.87440
C -1.59135 1.35687 -0.42729
Ru -0.36848 -0.19583 -0.07140
O 0.62283 -2.31868 -0.37121
C 0.17942 -2.26130 -1.57081
C 0.44515 -3.37380 -2.56006
O -0.48689 -1.21576 -1.94858
N -0.04696 0.45355 1.74094
C 0.15230 0.81578 2.84559
C 0.42644 1.23374 4.21514
H -5.42454 -0.20268 0.46438
H -0.51382 1.47849 4.73675
H 0.93576 0.42891 4.77206
H 1.07147 2.12829 4.22381
H -5.05186 1.76460 -0.36464
H -4.42821 4.02946 -1.19087
H -2.00737 4.61379 -1.50187
H -0.23925 2.96794 -1.01252
H -5.61424 -2.52372 1.37778
H -3.49652 -3.88537 1.70460
H -1.29223 -2.84778 1.05258
H -0.20350 -3.28226 -3.44292
H 1.49821 -3.32726 -2.88782
H 0.29156 -4.35292 -2.08006
Br 1.72285 1.00845 -0.95354
C 3.41032 0.36124 -0.17763
C 4.43561 1.29675 0.01165
C 3.54274 -0.99267 0.15098
C 5.65197 0.84432 0.55505
C 4.76778 -1.41910 0.69683
C 5.81838 -0.50792 0.89804
H 4.29698 2.34935 -0.25127
H 2.71284 -1.68812 -0.01319
H 6.46588 1.55935 0.71174
H 4.89499 -2.47379 0.96190
H 6.76678 -0.85105 1.32265

46
14c
SCF(BP86)= -1180.16434459
G(298 K) = -1179.878633
SCF(PBE) = -3739.59947180
Low Freq. = 8.6158cm-1, 11.3988cm-1
C 4.56641 1.13842 0.21151
C 3.31047 0.51067 0.04650
C 2.09063 1.21093 0.32309
C 2.19268 2.54571 0.78471
C 3.44529 3.16230 0.95112
C 4.63522 2.46306 0.66175
C 3.13125 -0.87567 -0.38553
C 4.15957 -1.78098 -0.71303
C 3.85816 -3.09425 -1.08616
C 2.51052 -3.49274 -1.11638
C 1.52536 -2.55937 -0.78769
N 1.80135 -1.27036 -0.44649
Ru 0.41984 0.19768 -0.03510
O -0.82433 1.98492 -0.11135
C -0.57087 2.11433 -1.36774
C -1.17903 3.22686 -2.18263
O 0.20008 1.23281 -1.91543
N 0.29722 -0.43011 1.80110
C 0.22733 -0.79677 2.92097
C 0.11073 -1.22911 4.30858
H 5.49337 0.59542 -0.00712
H 1.11004 -1.33084 4.76401
H -0.46565 -0.49418 4.89584
H -0.40003 -2.20490 4.37050
H 5.19917 -1.44529 -0.66945
H 4.65710 -3.79613 -1.34219
H 2.21685 -4.50991 -1.38783
H 0.46770 -2.83555 -0.79044

H   5.60771 2.94903 0.79060
H   3.49712 4.19828 1.30721
H   1.27925 3.10966 1.00547
H -0.48886 3.54008 -2.98039
H -2.10843 2.86897 -2.65923
H -1.42935 4.08340 -1.53986
Br -1.80473 -1.27533 -0.76806
C -3.48244 -0.50843 -0.12791
C -4.44824 -0.12964 -1.06969
C -3.67799 -0.36466 1.25215
C -5.65758 0.41478 -0.60044
C -4.89339 0.18421 1.69870
C -5.88067 0.57260 0.77753
H -4.26706 -0.25145 -2.14099
H -2.90350 -0.66811 1.96097
H -6.42306 0.71622 -1.32247
H -5.06248 0.30408 2.77351
H -6.82388 0.99787 1.13356

46
14d
SCF(BP86)= -1180.15940933
G(298 K) = -1179.873874
SCF(PBE) = -3739.59610142
Low Freq. = 11.5648cm-1,
12.7969cm-1
C -3.51685 -1.77512 1.53620
C -2.51113 -1.19175 0.73325
C -1.85673 0.02722 1.11648
C -2.27183 0.62384 2.33205
C -3.27359 0.04214 3.13034
C -3.89848 -1.15887 2.73573
C -2.03966 -1.74700 -0.53766
C -2.49152 -2.92527 -1.16426
C -1.94862 -3.32538 -2.39011
C -0.95041 -2.53212 -2.98362
C -0.53297 -1.37336 -2.32310
N -1.04913 -0.98152 -1.12872
Ru -0.43400 0.70271 -0.13867
O   0.42623 2.49497 0.72690
C   1.24846 2.69411 -0.25313
C   2.13248 3.92484 -0.24189
O   1.27906 1.86700 -1.22810
N -1.72681 1.77908 -1.08860
C -2.51028 2.44050 -1.66973
C -3.47163 3.26315 -2.39325
H -4.00435 -2.70858 1.23032
H -4.49165 2.86598 -2.25987
H -3.44909 4.30074 -2.02003
H -3.23827 3.27283 -3.47126
H -3.27114 -3.52329 -0.68428
H -2.29885 -4.24002 -2.87758
H -0.49874 -2.79959 -3.94226
H   0.23835 -0.71694 -2.73681
H -4.67724 -1.60815 3.36050
H -3.57192 0.52670 4.06789
H -1.79956 1.55841 2.66071
H   3.08611 3.71997 -0.75093
H   1.62628 4.74090 -0.78699
H   2.31844 4.26574 0.78733
Br 1.15871 -0.67948 1.32196
C   2.95712 -0.83134 0.56520
C   4.01303 -0.18929 1.22461
C   3.14124 -1.59122 -0.59741
C   5.30748 -0.31872 0.68848
C   4.44268 -1.70344 -1.11808
C   5.52219 -1.07084 -0.47826
H   3.83698 0.39862 2.12941
H   2.29540 -2.08031 -1.08769
H   6.14548 0.17561 1.18981
H   4.60645 -2.29218 -2.02613
H   6.53186 -1.16505 -0.88926

46
14e
SCF(BP86)= -1180.15371016
G(298 K) = -1179.867757
SCF(PBE) = -3739.58901926
Low Freq. = 4.3822cm-1, 15.0477cm-
1
C   3.55320 2.28116 -0.54433
C   2.45696 1.51422 -0.08683
C   2.16868 0.21630 -0.62935
C   3.03815 -0.25683 -1.64450
C   4.12750 0.50724 -2.10048
C   4.38844 1.77936 -1.55141
C   1.53520 1.95250 0.96483
C   1.59843 3.17045 1.66959
C   0.66665 3.46566 2.66997
C -0.33445 2.52042 2.95612
C -0.36431 1.32762 2.23152
N   0.53935 1.02729 1.25545
Ru 0.56801 -0.71161 0.19351
O   1.10220 -2.67241 -0.63974
C   1.83443 -2.89160 0.39155
C   2.56729 -4.19265 0.59186
O   1.89630 -1.95518 1.28629
N -1.04333 -1.63261 1.16709
C -1.91660 -2.19805 1.71947
C -3.00326 -2.88822 2.40367
H   3.76058 3.27044 -0.11907
H -3.96758 -2.40946 2.16647
H -3.04742 -3.94378 2.08830
H -2.85213 -2.85264 3.49527
H   2.38948 3.88525 1.42670
H   0.71929 4.41181 3.21647
H -1.08878 2.69821 3.72722
H -1.12334 0.56428 2.41576
H   5.23640 2.37342 -1.90796
H   4.77940 0.11245 -2.88948
H   2.85712 -1.24529 -2.08477
H   3.48128 -4.03488 1.18357
H   1.92142 -4.89971 1.14143
H   2.81855 -4.64256 -0.37998
Br -0.78811 0.28758 -1.67296
C -2.69246 0.56268 -1.26292
C -3.61153 -0.41864 -1.65750
C -3.07786 1.73304 -0.59520
C -4.97276 -0.21047 -1.36928
C -4.44367 1.92014 -0.31458
C -5.38764 0.95316 -0.69983
H -3.27993 -1.32227 -2.17636

H -2.33720 2.48151 -0.30181
H -5.70588 -0.96478 -1.67212
H -4.76423 2.82914 0.20420
H -6.44829 1.10738 -0.47936

51
22a
SCF(BP86)= -1217.02920753
G(298 K) = -1216.703177
SCF(PBE) = -3776.40806341
Low Freq. = 28.9653cm-1,
32.0018cm-1
C 1.85993 -0.50634 0.46059
C 2.55768 0.72174 0.50332
C 3.91403 0.72929 0.91263
C 4.54416 -0.46392 1.27830
C 3.82983 -1.67853 1.24279
C 2.48316 -1.70121 0.84352
C 1.82178 1.90648 0.06368
C 2.36620 3.20077 -0.05951
C 1.58378 4.24709 -0.55187
C 0.25429 3.98562 -0.92170
C -0.24478 2.69202 -0.76508
N 0.50802 1.67136 -0.27637
H 3.40757 3.37436 0.22023
Ru -0.18516 -0.29561 -0.10513
N -1.43378 -0.00267 -1.74886
C -2.15143 0.13858 -2.66015
C -3.02944 0.30565 -3.80490
N -0.01371 -0.45878 1.91043
C 0.04276 -0.61085 3.06758
C 0.10827 -0.78464 4.50882
H 1.15815 -0.78558 4.84310
H -0.43062 0.03708 5.00781
H -0.35996 -1.74186 4.78973
H 4.47470 1.66881 0.94519
H 5.59117 -0.45162 1.59540
H 4.32162 -2.61359 1.53135
H 1.93435 -2.64693 0.82263
H 2.00558 5.25093 -0.65330
H -0.39637 4.76675 -1.32134
H -1.27583 2.44700 -1.02101
H -2.60413 -0.21414 -4.67870
H -4.02080 -0.11897 -3.57869
H -3.14076 1.37647 -4.03911
Br 1.25084 -0.98681 -2.28589
C -2.01144 0.41111 0.79595
C -2.06407 1.60482 1.54004
C -3.18446 -0.35059 0.63416
C -3.28408 2.05000 2.08365
C -4.40315 0.10087 1.17708
C -4.45862 1.30297 1.90013
H -1.15894 2.19092 1.72383
H -3.16960 -1.29079 0.07611
H -3.30530 2.98199 2.65915
H -5.30817 -0.49927 1.03136
H -5.40627 1.64957 2.32473
N -0.74610 -2.24642 -0.03920
C -1.04887 -3.37611 -0.01747
C -1.41960 -4.78235 0.00284
H -1.33073 -5.17820 1.02720
H -2.46032 -4.90143 -0.33896
H -0.75441 -5.35511 -0.66322

51
22b
SCF(BP86)= -1217.02645967
G(298 K) = -1216.700745
SCF(PBE) = -3776.40512828
Low Freq. = 26.8980cm-1,
28.2836cm-1
C -1.81729 0.45568 0.81135
C -2.54929 -0.75141 0.75708
C -3.84795 -0.79212 1.31862
C -4.39296 0.35323 1.91008
C -3.65434 1.55101 1.94634
C -2.35894 1.60345 1.39975
C -1.90827 -1.87610 0.06965
C -2.49803 -3.13813 -0.13773
C -1.81739 -4.11746 -0.86543
C -0.54951 -3.81715 -1.38720
C -0.00094 -2.55614 -1.14334
N -0.65013 -1.60655 -0.42100
H -3.49280 -3.34083 0.26493
Ru 0.09374 0.33065 -0.08737
N -1.24575 1.04547 -1.55183
C -1.98940 1.45528 -2.35443
C -2.89510 1.95960 -3.37277
N 0.27884 0.17883 1.92905
C 0.38050 0.16116 3.09324
C 0.52112 0.12294 4.53915
H -0.39298 -0.28962 4.99574
H 1.38129 -0.50806 4.81577
H 0.68488 1.14259 4.92373
H -4.43451 -1.71568 1.29027
H -5.39727 0.31496 2.34235
H -4.08344 2.44770 2.40557
H -1.78449 2.53340 1.44109
H -2.27305 -5.09783 -1.03051
H 0.01743 -4.54303 -1.97466
H 0.97857 -2.26916 -1.52898
H -3.86697 1.44605 -3.29819
H -3.04754 3.04190 -3.23397
H -2.46726 1.78105 -4.37261
Br 1.42860 0.28592 -2.39743
C 2.04711 -0.47707 0.45976
C 2.19185 -1.79323 0.93353
C 3.18003 0.35657 0.39427
C 3.46559 -2.29229 1.27278
C 4.44885 -0.14420 0.73964
C 4.59947 -1.47266 1.17241
H 1.32275 -2.44242 1.06912
H 3.09503 1.38790 0.04434
H 3.55688 -3.32395 1.63036
H 5.32092 0.51499 0.66365
H 5.58822 -1.85918 1.43993
N 0.68183 2.25604 0.15996
C 0.98013 3.38065 0.28251
C 1.35668 4.77744 0.43332
H 1.94215 4.90914 1.35744
H 1.96733 5.09588 -0.42676
H 0.45438 5.40758 0.48699

```
51
22c
I-MeCN_LPh_ax_BrLL_2
SCF(BP86)= -1217.02192472
G(298 K) = -1216.696468
SCF(PBE) = -3776.39984001
Low Freq. = 29.7937cm-1,
32.4102cm-1
C   1.84951 -0.47298 -0.53579
C   2.54733  0.75885 -0.54844
C   3.91037  0.78085 -0.93400
C   4.56645 -0.40847 -1.26533
C   3.87333 -1.63449 -1.20600
C   2.51761 -1.66857 -0.84135
C   1.80281  1.93779 -0.10491
C   2.32152  3.24710 -0.03784
C   1.54499  4.28442  0.48203
C   0.24865  3.99863  0.94097
C  -0.22777  2.69128  0.83510
N   0.51409  1.68111  0.30959
H   3.33813  3.44076 -0.38686
Ru -0.12741 -0.30866  0.19251
N  -1.33051 -0.04466  1.91072
C  -1.94173  0.08285  2.89906
C  -2.71953  0.24901  4.11516
N   1.12184 -0.80367  1.84509
C   1.75763 -1.07872  2.78636
C   2.55956 -1.42419  3.94852
H   3.23121 -0.58830  4.20141
H   1.90200 -1.63288  4.80748
H   3.16327 -2.31974  3.73038
H   4.46129  1.72637 -0.96095
H   5.62010 -0.38593 -1.55927
H   4.38925 -2.56896 -1.45142
H   1.98280 -2.62215 -0.81976
H   1.94667  5.30013  0.53695
H  -0.39365  4.77086  1.37041
H  -1.23356  2.42564  1.16199
H  -3.79276  0.27567  3.86671
H  -2.52822 -0.59218  4.80035
H  -2.44048  1.19191  4.61213
Br -0.10098 -0.45531 -2.43902
C  -2.05958  0.28858 -0.57110
C  -3.15302 -0.57529 -0.36241
C  -2.29537  1.54249 -1.17083
C  -4.45521 -0.18884 -0.73660
C  -3.59726  1.92711 -1.54025
C  -4.68510  1.06458 -1.32388
H  -3.00966 -1.55490  0.10081
H  -1.46563  2.21600 -1.40024
H  -5.28818 -0.87893 -0.56066
H  -3.75107  2.90345 -2.01348
H  -5.69697  1.36409 -1.61563
N  -0.60486 -2.27611  0.09769
C  -0.83656 -3.42266  0.06110
C  -1.12721 -4.84702  0.00457
H  -0.72801 -5.34834  0.90091
H  -2.21648 -5.00726 -0.04098
H  -0.66201 -5.28903 -0.89122
```

```
51
22d
SCF(BP86)= -1217.02153999
G(298 K) = -1216.695701
SCF(PBE) = -3776.39957068
Low Freq. = 15.4701cm-1,
17.5282cm-1
C  -1.87610  0.38867  0.56498
C  -2.19738  1.71561  0.21863
C  -3.45693  2.24936  0.59014
C  -4.37202  1.46307  1.29191
C  -4.04074  0.13585  1.62929
C  -2.79418 -0.40293  1.27158
C  -1.17695  2.42863 -0.52777
C  -1.28205  3.74479 -1.02093
C  -0.22965  4.29947 -1.74911
C   0.92041  3.52364 -1.97029
C   0.97711  2.23112 -1.44820
N  -0.04674  1.68216 -0.74129
H  -2.19299  4.31718 -0.83209
Ru  0.03474 -0.36361 -0.02581
N   0.70469 -0.63094 -1.95494
C   1.11498 -0.84922 -3.02694
C   1.62740 -1.12063 -4.35808
N  -1.60233 -1.50306 -1.09350
C  -2.34995 -2.12277 -1.74518
C  -3.28577 -2.90555 -2.53848
H  -4.30748 -2.77697 -2.14686
H  -3.25879 -2.57152 -3.58792
H  -3.01364 -3.97226 -2.49361
H  -3.71092  3.28064  0.32601
H  -5.34286  1.87794  1.57816
H  -4.75448 -0.48844  2.17665
H  -2.54998 -1.43375  1.54082
H  -0.30200  5.31840 -2.13935
H   1.77387  3.90791 -2.53361
H   1.86253  1.61014 -1.58019
H   2.62233 -1.58842 -4.28216
H   0.94605 -1.80620 -4.88686
H   1.71204 -0.18055 -4.92624
Br  0.74828 -2.81401  0.43136
C   1.92803  0.33482  0.56622
C   3.07900 -0.31298  0.06635
C   2.10037  1.45129  1.41208
C   4.36580  0.15408  0.40368
C   3.38584  1.91227  1.74850
C   4.52651  1.26539  1.24480
H   2.99048 -1.19115 -0.57867
H   1.22829  1.97977  1.81108
H   5.24262 -0.36748  0.00352
H   3.48924  2.78237  2.40651
H   5.52783  1.62315  1.50565
N  -0.06595 -0.38633  1.99057
C  -0.04299 -0.44502  3.15672
C  -0.01727 -0.52604  4.60776
H  -1.00105 -0.24628  5.01779
H   0.22412 -1.55479  4.92058
H   0.74802  0.15896  5.00711
```

```
51
22e
SCF(BP86)= -1217.01741107
G(298 K) = -1216.691268
SCF(PBE) = -3776.39545032
Low Freq. = 23.4749cm-1,
31.1183cm-1
C  -1.21548  1.53646 -0.31853
C  -2.60526  1.27792 -0.28276
C  -3.51408  2.35442 -0.44280
C  -3.05285  3.65947 -0.62201
C  -1.66875  3.90891 -0.63671
C  -0.75584  2.85267 -0.48307
C  -3.01879 -0.10152 -0.06518
C  -4.35032 -0.56668 -0.06177
C  -4.60620 -1.92546  0.13138
C  -3.52275 -2.79916  0.31756
C  -2.22783 -2.27350  0.31663
N  -1.97163 -0.95562  0.13951
H  -5.17303  0.13463 -0.21823
Ru  0.17149 -0.09196  0.00605
N   0.23354 -1.64268 -1.31245
C   0.31156 -2.52842 -2.07038
C   0.40023 -3.63659 -3.00544
N   0.33381  1.16126  1.58965
C   0.42917  1.80335  2.56042
C   0.54951  2.60167  3.76896
H  -0.18140  3.42594  3.74898
H   0.35757  1.97158  4.65250
H   1.56586  3.02121  3.84141
H  -4.59055  2.15920 -0.41640
H  -3.76515  4.48076 -0.74317
H  -1.28978  4.92758 -0.76843
H   0.31499  3.07164 -0.49632
H  -5.63404 -2.29949  0.13200
H  -3.66834 -3.87199  0.46487
H  -1.35760 -2.91113  0.48224
H   0.64378 -3.25726 -4.01075
H   1.18890 -4.33488 -2.68188
H  -0.56242 -4.17114 -3.04295
Br  0.54516 -1.78347  1.92438
C   2.30304  0.09271  0.04131
C   2.93944  1.33868  0.20835
C   3.10573 -1.05026 -0.15655
C   4.34382  1.44012  0.20267
C   4.51036 -0.94696 -0.16302
C   5.13654  0.29648  0.01890
H   2.35031  2.25158  0.34217
H   2.65156 -2.03637 -0.28598
H   4.81160  2.42167  0.33920
H   5.11124 -1.85190 -0.30833
H   6.22874  0.37327  0.01460
N   0.66402  0.83381 -1.75114
C   0.98115  1.21689 -2.80848
C   1.37949  1.72112 -4.11247
H   2.45565  1.95783 -4.11152
H   1.17849  0.96085 -4.88426
H   0.80795  2.63281 -4.35002
```

```
51
22f
SCF(BP86)= -1217.01125141
G(298 K) = -1216.685601
SCF(PBE) = -3776.38901855
Low Freq. = 20.4921cm-1,
29.1826cm-1
C   1.32246 -1.42201 -0.08990
C   2.68591 -1.04620 -0.01555
C   3.68945 -2.04981 -0.05720
C   3.34783 -3.39876 -0.13856
C   1.98862 -3.76645 -0.16967
C   0.98593 -2.78613 -0.14586
C   2.98099  0.36916  0.15016
C   4.26666  0.94974  0.18424
C   4.39858  2.32566  0.37988
C   3.24063  3.10622  0.53578
C   1.99659  2.47343  0.48170
N   1.86170  1.13882  0.29783
H   5.15126  0.32233  0.05360
Ru -0.18225  0.12477  0.06336
N  -0.29708  1.65607 -1.26501
C  -0.42365  2.56394 -1.98947
C  -0.58002  3.67091 -2.91665
N  -0.45957 -1.14721  1.62387
C  -0.66738 -1.75517  2.59947
C  -0.92373 -2.53519  3.79913
H  -0.40415 -3.50458  3.73115
H  -0.55655 -1.99294  4.68528
H  -2.00578 -2.71234  3.90790
H   4.74321 -1.75891 -0.00419
H   4.12983 -4.16326 -0.16500
H   1.70080 -4.82161 -0.22180
H  -0.06058 -3.09544 -0.19441
H   5.39025  2.78606  0.40852
H   3.29176  4.18614  0.69288
H   1.07241  3.04449  0.58724
H  -0.54351  3.29413 -3.95169
H  -1.55087  4.16393 -2.74678
H   0.22841  4.40439 -2.76839
Br -0.41383 -1.03491 -2.31047
C  -2.27574 -0.14615 -0.07609
C  -2.89077 -1.41651 -0.05362
C  -3.10796  0.99267 -0.14644
C  -4.28998 -1.54680 -0.10640
C  -4.51046  0.86158 -0.20020
C  -5.10872 -0.40694 -0.18090
H  -2.27984 -2.32375 -0.01236
H  -2.68151  2.00059 -0.15926
H  -4.73663 -2.54760 -0.09402
H  -5.12980  1.76409 -0.25602
H  -6.19813 -0.50797 -0.22392
N  -0.59089  1.44171  1.59156
C  -0.90654  2.12913  2.48281
C  -1.29612  2.99057  3.58478
H  -1.01862  2.51654  4.54000
H  -0.78688  3.96379  3.50071
H  -2.38679  3.14655  3.56307
```

51
TS(20a-22b)
SCF(BP86)= -1217.01627998
G(298 K) = -1216.691273
SCF(PBE) = -3776.39444313
Low Freq. = -178.9851cm-1, 25.5500cm-1

```
C   1.81199 -0.61783  0.90233
C   2.69348  0.49598  0.81988
C   3.95169  0.45195  1.46219
C   4.33361 -0.68809  2.18101
C   3.46456 -1.79107  2.26111
C   2.20735 -1.75280  1.62910
C   2.23190  1.63258  0.01274
C   2.96618  2.81206 -0.22115
C   2.45981  3.79859 -1.07167
C   1.22221  3.58435 -1.69774
C   0.52606  2.40492 -1.42141
N   0.99445  1.45956 -0.56893
H   3.93867  2.94543  0.25801
Ru  0.03391 -0.35423 -0.09764
N   1.02842 -1.19483 -1.65362
C   1.65850 -1.68979 -2.50542
C   2.43547 -2.30468 -3.56909
N  -0.38764  0.32399  1.77399
C  -0.54654  0.68272  2.87536
C  -0.75374  1.13536  4.24204
H   0.21429  1.37473  4.71080
H  -1.38905  2.03550  4.24628
H  -1.24974  0.34451  4.82758
H   4.63792  1.30279  1.40153
H   5.30837 -0.71731  2.67745
H   3.76273 -2.68405  2.82106
H   1.53962 -2.61516  1.71169
H   3.02894  4.71442 -1.25476
H   0.79338  4.31149 -2.39114
H  -0.42946  2.17916 -1.90062
H   3.46260 -1.90575 -3.56084
H   2.47104 -3.39654 -3.42572
H   1.97250 -2.08446 -4.54450
Br -1.71059 -0.17828 -2.23293
C  -2.26128  0.48030  0.05700
C  -2.42044  1.86591  0.19880
C  -3.22443 -0.41833  0.53731
C  -3.54914  2.35300  0.88614
C  -4.34474  0.08795  1.22308
C  -4.51331  1.47131  1.40150
H  -1.68566  2.57395 -0.18854
H  -3.12456 -1.49340  0.37970
H  -3.66318  3.43568  1.00916
H  -5.09213 -0.61683  1.60404
H  -5.39219  1.85844  1.92608
N  -0.67020 -2.18631  0.36118
C  -1.01849 -3.28270  0.58445
C  -1.45789 -4.64088  0.86781
H  -2.43581 -4.62247  1.37565
H  -1.55501 -5.20756 -0.07231
H  -0.72675 -5.14848  1.51771
```

45
23a
SCF(BP86)= -1084.27564888
G(298 K) = -1083.991868
SCF(PBE) = -3643.76026886
Low Freq. = 2.9808cm-1, 34.6214cm-1

```
C  -1.74503  0.77225  0.27293
C  -2.60801 -0.34038  0.40903
C  -3.98652 -0.11653  0.61836
C  -4.47708  1.19355  0.70060
C  -3.60474  2.28987  0.57589
C  -2.22346  2.08493  0.39217
C  -1.98067 -1.64645  0.22307
C  -2.63226 -2.89076  0.29941
C  -1.93298 -4.06002 -0.01278
C  -0.59188 -3.96077 -0.41490
C   0.01297 -2.70161 -0.45125
N  -0.64939 -1.56675 -0.11031
H  -3.68496 -2.93189  0.58788
Ru  0.19274  0.36494 -0.23076
N   0.94625  2.22808 -0.51556
C   1.40034  3.28890 -0.71300
C   1.95232  4.60976 -0.95878
N   0.22880  0.88746  1.77561
C   0.25649  1.25334  2.88539
C   0.28980  1.69922  4.26858
H  -0.71558  1.61817  4.71200
H   0.99099  1.07462  4.84489
H   0.62092  2.74911  4.31489
H  -4.67659 -0.96178  0.70300
H  -5.54544  1.36185  0.86479
H  -3.99116  3.31204  0.64026
H  -1.54640  2.94088  0.33438
H  -2.43093 -5.03212  0.03970
H  -0.01046 -4.84125 -0.69763
H   1.05198 -2.57971 -0.75804
H   1.14746  5.36197 -0.92092
H   2.70588  4.84994 -0.19160
H   2.42546  4.63828 -1.95341
Br  0.09074  0.04485 -2.66346
C   1.88263 -0.54992  0.55342
C   3.00991 -0.21064 -0.21899
C   1.97446 -1.46420  1.61163
C   4.23074 -0.87117  0.04009
C   3.20582 -2.09543  1.86979
C   4.33192 -1.80546  1.08156
H   2.96622  0.53280 -1.01986
H   1.10153 -1.69912  2.22741
H   5.10276 -0.62328 -0.57430
H   3.27515 -2.81443  2.69277
H   5.28863 -2.29549  1.28693
```

45
23a (triplet)
SCF(BP86)= -1084.24606016
G(298 K) = -1083.964937
SCF(PBE) = -3643.72682550
Low Freq. = 25.1253cm-1, 29.4049cm-1

```
C  -1.83469  0.81691  0.04970
```

C -2.71663 -0.28555 0.19088
C -4.09465 -0.06770 0.40057
C -4.58558 1.24418 0.46809
C -3.71280 2.34028 0.31950
C -2.33730 2.13237 0.11583
C -2.09264 -1.59908 0.02280
C -2.78283 -2.82578 0.00756
C -2.09984 -4.01123 -0.27533
C -0.72813 -3.94622 -0.56051
C -0.08436 -2.70718 -0.50761
N -0.73150 -1.55587 -0.19296
H -3.85837 -2.83821 0.19693
Ru 0.19774 0.35943 -0.09891
N   0.97216 2.22038 -0.08775
C   1.42083 3.30203 -0.09108
C   1.97397 4.64431 -0.09700
N   0.06617 0.43256 1.95141
C -0.03251 0.53769 3.11171
C -0.15153 0.65892 4.55479
H   0.64568 1.31279 4.94310
H -1.13159 1.09228 4.81147
H -0.06427 -0.33548 5.02146
H -4.78682 -0.90887 0.50660
H -5.65400 1.41481 0.63063
H -4.10422 3.36212 0.36407
H -1.67067 2.99352 0.01170
H -2.63255 -4.96600 -0.29387
H -0.15111 -4.83589 -0.82270
H   0.98118 -2.61646 -0.71840
H   2.60021 4.79668 0.79672
H   2.58626 4.79310 -1.00094
H   1.15471 5.38203 -0.09269
Br 0.25675 0.35764 -2.57436
C   2.02744 -0.64775 0.23497
C   3.12986 -0.24535 -0.57005
C   2.26067 -1.54751 1.30661
C   4.43026 -0.66959 -0.25350
C   3.55995 -1.97316 1.61428
C   4.64922 -1.53375 0.83543
H   2.97430 0.40473 -1.43623
H   1.42554 -1.90085 1.92016
H   5.27121 -0.33723 -0.87155
H   3.72551 -2.64814 2.46045
H   5.66230 -1.87774 1.06689

45
23b
I-2MeCN_Br_trans-
N_Ph_trans_C_2_ts_cbr_2_ircr_opt_2
SCF(BP86)= -1084.27451449
G(298 K) = -1083.990695
SCF(PBE) = -3643.75864029
Low Freq. = 30.9824cm-1,
34.9177cm-1
N -0.52184 1.67347 -0.18832
C -1.83984 1.84153 -0.53055
C -2.36163 3.12019 -0.80442
C -1.54447 4.24482 -0.67258
C -0.21825 4.06867 -0.24789
C   0.25357 2.77383 -0.01786
C -2.59999 0.60090 -0.49738
C -3.99070 0.47901 -0.71164
C -4.60897 -0.76911 -0.57285
C -3.84691 -1.89939 -0.22685
C -2.45452 -1.80100 -0.03775
C -1.84621 -0.53672 -0.13916
H -4.58639 1.35799 -0.97654
Ru 0.14793 -0.37444 0.27962
N   0.17450 -1.31076 -1.51695
C   0.18218 -1.90885 -2.52043
C   0.20094 -2.64639 -3.77290
N   0.11505 0.38385 2.13735
C   0.07676 0.78375 3.23675
C   0.03364 1.28152 4.59944
H   0.00119 0.43651 5.30578
H   0.93252 1.88682 4.80128
H -0.86112 1.90913 4.74021
H -3.40832 3.22128 -1.10048
H -1.93915 5.24338 -0.87942
H   0.45553 4.91558 -0.09936
H   1.28068 2.60054 0.30409
H -5.68575 -0.86444 -0.74013
H -4.32746 -2.87750 -0.12167
H -1.87160 -2.69423 0.19536
H   0.26827 -3.72730 -3.57025
H -0.72046 -2.44265 -4.34183
H   1.07164 -2.33792 -4.37376
Br 0.90219 -2.55714 1.20802
C   1.84878 0.45382 -0.54736
C   2.05489 0.88845 -1.86340
C   2.89531 0.45456 0.39529
C   3.34351 1.29378 -2.25871
C   4.18537 0.84461 -0.02600
C   4.41022 1.26516 -1.34504
H   1.22883 0.92339 -2.57888
H   2.74296 0.15527 1.43605
H   3.50381 1.63250 -3.28759
H   5.00267 0.82941 0.70269
H   5.41018 1.58280 -1.65602

45
23b (triplet)
SCF(BP86)= -1084.24690213
G(298 K) = -1083.965111
SCF(PBE) = -3643.72740664
Low Freq. = -21.2600cm-1,
13.5383cm-1
N -0.48918 1.69568 0.11473
C -1.84085 1.91059 -0.02353
C -2.35189 3.22260 -0.05200
C -1.49353 4.31414 0.10109
C -0.12483 4.07616 0.29496
C   0.33696 2.75785 0.29434
C -2.65052 0.69047 -0.09500
C -4.06063 0.69025 -0.15928
C -4.76079 -0.52370 -0.18742
C -4.06025 -1.74366 -0.14615
C -2.65613 -1.75483 -0.08651
C -1.94045 -0.54083 -0.02306
H -4.61807 1.63174 -0.18238
Ru 0.15819 -0.34990 0.13218
N -0.01690 -0.54083 -1.88348

```
C  -0.14913 -0.70142 -3.03285
C  -0.30954 -0.89891 -4.46382
N   0.26626 -0.22691  2.12206
C   0.29741 -0.15954  3.29063
C   0.33674 -0.08202  4.73929
H   1.20877 -0.63831  5.11945
H   0.40726  0.96988  5.05918
H  -0.58149 -0.52606  5.15777
H  -3.42467  3.37904 -0.18288
H  -1.88848  5.33361  0.08308
H   0.58717  4.89104  0.44408
H   1.39309  2.52834  0.43471
H  -5.85326 -0.51858 -0.24649
H  -4.60735 -2.69211 -0.16830
H  -2.12064 -2.70846 -0.07855
H   0.51295 -1.52270 -4.84899
H  -1.26874 -1.40233 -4.66512
H  -0.29744  0.07420 -4.98052
Br  0.67935 -2.78394  0.27188
C   2.10861  0.28094 -0.33606
C   2.47316  1.07875 -1.45112
C   3.12515 -0.09797  0.58791
C   3.79334  1.51697 -1.62154
C   4.44447  0.34806  0.41723
C   4.78350  1.15315 -0.68702
H   1.71641  1.37718 -2.18352
H   2.88907 -0.74371  1.43895
H   4.05357  2.14077 -2.48293
H   5.21160  0.05440  1.14163
H   5.81666  1.48755 -0.82462

45
23c
SCF(BP86)= -1084.26819709
G(298 K) = -1083.985232
SCF(PBE) = -3643.74595360
Low Freq. = 28.3062cm-1,
34.0646cm-1
N   1.18028  1.21560 -0.28939
C   2.46398  0.77183 -0.05665
C   3.55747  1.64724 -0.19826
C   3.34042  2.97509 -0.58097
C   2.02650  3.40894 -0.81932
C   0.97377  2.50038 -0.66390
C   2.52613 -0.63481  0.33436
C   3.70409 -1.30884  0.70013
C   3.64452 -2.66309  1.06777
C   2.41400 -3.35487  1.07512
C   1.23547 -2.69626  0.70672
C   1.25465 -1.31988  0.34760
H   4.66962 -0.79381  0.70458
Ru -0.30795 -0.21789 -0.13567
N  -2.01564  1.08502 -0.86364
C  -2.90570  1.73103 -1.26292
C  -4.00869  2.53577 -1.76609
N  -1.65395 -1.73964 -0.11049
C  -2.42144 -2.62207 -0.17946
C  -3.37060 -3.72060 -0.25586
H  -4.05750 -3.68376  0.60524
H  -3.95718 -3.64568 -1.18572
H  -2.83374 -4.68295 -0.24576
H   4.57044  1.28371 -0.01095
H   4.18405  3.66122 -0.69415
H   1.80872  4.43502 -1.12458
H  -0.06509  2.78670 -0.83880
H   4.56421 -3.18564  1.34774
H   2.38492 -4.40968  1.36570
H   0.28931 -3.24378  0.70727
H  -4.85232  1.88326 -2.04224
H  -4.34023  3.24448 -0.99058
H  -3.68459  3.09984 -2.65526
Br -0.04711 -0.41970 -2.67695
C  -0.90503  0.64170  1.60104
C   0.00942  1.31496  2.45872
C  -2.21754  0.37032  2.08588
C  -0.33047  1.57005  3.79433
C  -2.55532  0.64137  3.41723
C  -1.61234  1.24065  4.27465
H   1.00447  1.59652  2.10749
H  -2.96298 -0.07685  1.42397
H   0.40187  2.04072  4.45799
H  -3.55696  0.39642  3.78459
H  -1.88295  1.47035  5.31013

45
23c (triplet)
SCF(BP86)= -1084.26636880
G(298 K) = -1083.985488
SCF(PBE) = -3643.74479713
Low Freq. = 26.7284cm-1,
29.6403cm-1
N   1.14741  1.26275  0.52066
C   2.42902  0.75976  0.45378
C   3.51250  1.53980  0.89448
C   3.28451  2.82095  1.41019
C   1.97039  3.31067  1.48162
C   0.92729  2.50147  1.02525
C   2.50516 -0.60105 -0.08928
C   3.70606 -1.31848 -0.23567
C   3.68063 -2.62624 -0.74926
C   2.45740 -3.22324 -1.11692
C   1.25647 -2.51378 -0.97896
C   1.24697 -1.19019 -0.46997
H   4.66511 -0.87345  0.04678
Ru -0.32433  0.01672 -0.25658
N  -1.96938  1.50039 -0.01380
C  -2.83486  2.28581  0.04713
C  -3.90920  3.26266  0.12326
N  -1.67658 -1.30604 -1.05111
C  -2.46748 -2.03858 -1.50451
C  -3.44346 -2.95493 -2.06847
H  -3.96835 -3.48233 -1.25550
H  -4.17951 -2.39618 -2.66846
H  -2.93787 -3.69412 -2.71037
H   4.52804  1.14280  0.83388
H   4.12441  3.43034  1.75500
H   1.74790  4.30209  1.88193
H  -0.11317  2.82941  1.05154
H   4.61663 -3.18160 -0.86030
H   2.44665 -4.24439 -1.51119
H   0.31581 -2.98970 -1.26935
H  -4.66238  3.05032 -0.65210
```

H -4.38786 3.21465 1.11441
H -3.50548 4.27564 -0.03361
Br -0.10171 1.05592 -2.60287
C -0.82354 -0.71727 1.59168
C 0.16538 -1.15675 2.51990
C -2.18623 -0.77378 2.00330
C -0.19113 -1.59429 3.80189
C -2.53989 -1.22385 3.28166
C -1.54424 -1.63169 4.18983
H 1.22275 -1.15016 2.24063
H -2.97662 -0.46696 1.31297
H 0.58958 -1.91683 4.49884
H -3.59542 -1.25988 3.57073
H -1.82072 -1.98401 5.18857

45
23d
SCF(BP86)= -1084.26611883
G(298 K) = -1083.983579
SCF(PBE) = -3643.74378600
Low Freq. = 22.5138cm-1,
30.0072cm-1
N -1.15117 -0.99086 -0.84462
C -2.31008 -1.07588 -0.10289
C -3.40535 -1.83089 -0.56022
C -3.31141 -2.52723 -1.77026
C -2.11552 -2.45766 -2.50032
C -1.05998 -1.68183 -2.00649
C -2.24451 -0.34588 1.16305
C -3.22419 -0.39885 2.17194
C -3.04456 0.33328 3.35626
C -1.89270 1.12507 3.54815
C -0.91653 1.20215 2.54570
C -1.05143 0.42259 1.37176
H -4.12437 -1.00802 2.04613
Ru 0.34369 0.23344 -0.04231
N 1.69809 1.50043 0.76045
C 2.46453 2.27734 1.18640
C 3.41438 3.23863 1.72336
N -0.83098 2.09597 -0.67160
C -1.44493 3.04327 -0.97793
C -2.20289 4.22262 -1.36853
H -1.51903 5.07248 -1.52226
H -2.74552 4.02361 -2.30644
H -2.92790 4.48085 -0.58038
H -4.32095 -1.87300 0.03420
H -4.15595 -3.11893 -2.13361
H -1.98985 -2.99117 -3.44542
H -0.11441 -1.58567 -2.54397
H -3.81346 0.29532 4.13372
H -1.76614 1.68975 4.47708
H -0.03812 1.83665 2.68718
H 3.17363 3.45911 2.77598
H 4.43607 2.82952 1.66707
H 3.37053 4.17352 1.14166
Br 1.30593 0.72051 -2.36631
C 1.41403 -1.37106 0.45491
C 0.80599 -2.62097 0.79981
C 2.84277 -1.29399 0.52915
C 1.57304 -3.69888 1.25365
C 3.60975 -2.38946 0.93630
C 2.97722 -3.59257 1.31078
H -0.28038 -2.73308 0.75518
H 3.34874 -0.36933 0.23879
H 1.08167 -4.63108 1.55023
H 4.70102 -2.31041 0.97073
H 3.57719 -4.44701 1.63981

45
23d (triplet)
SCF(BP86)= -1084.26157055
G(298 K) = -1083.981037
SCF(PBE) = -3643.74101714
Low Freq. = 26.7963cm-1,
30.0164cm-1
N -1.25550 -0.82229 -0.90825
C -2.37374 -0.94096 -0.11085
C -3.52380 -1.59617 -0.58957
C -3.52803 -2.15589 -1.86969
C -2.37031 -2.05616 -2.65682
C -1.25870 -1.38287 -2.14560
C -2.21205 -0.36701 1.22464
C -3.19604 -0.41666 2.23269
C -2.93233 0.13227 3.49474
C -1.68357 0.72834 3.75800
C -0.69713 0.78712 2.76166
C -0.94158 0.23666 1.48250
H -4.16556 -0.88668 2.04189
Ru 0.32729 0.31080 -0.10312
N 1.68996 1.47581 0.86068
C 2.44920 2.19280 1.38986
C 3.39306 3.08083 2.04093
N -0.79409 2.16758 -0.65607
C -1.39790 3.13138 -0.92857
C -2.14693 4.32995 -1.27237
H -1.45208 5.16053 -1.47409
H -2.75688 4.14318 -2.17058
H -2.81003 4.60792 -0.43772
H -4.40669 -1.66878 0.04911
H -4.41765 -2.66901 -2.24482
H -2.31645 -2.48922 -3.65817
H -0.33999 -1.27223 -2.72398
H -3.69737 0.09205 4.27552
H -1.47548 1.14821 4.74754
H 0.26324 1.25637 2.98797
H 2.93322 3.50466 2.94892
H 4.30258 2.52526 2.32014
H 3.66048 3.90310 1.35746
Br 1.48907 0.61879 -2.35587
C 1.34288 -1.36963 0.41041
C 0.69367 -2.59435 0.73974
C 2.76882 -1.36279 0.42934
C 1.42516 -3.74371 1.06767
C 3.50087 -2.51627 0.74115
C 2.83300 -3.71270 1.06552
H -0.39868 -2.64782 0.76335
H 3.31230 -0.45030 0.16697
H 0.89709 -4.66715 1.32817
H 4.59554 -2.48347 0.73192
H 3.40422 -4.61232 1.31608

45
23e
SCF(BP86)= -1084.26019592
G(298 K) = -1083.976833
SCF(PBE) = -3643.74255541
Low Freq. = 24.0790cm-1,
26.3669cm-1
N -1.77023 -0.69697 0.06966
C -2.64471 0.29282 0.42699
C -3.99930 -0.01867 0.66756
C -4.44542 -1.33492 0.55181
C -3.52671 -2.33765 0.19875
C -2.19544 -1.98780 -0.02532
C -2.03586 1.61271 0.50505
C -2.78172 2.78865 0.75494
C -2.15622 4.03763 0.72544
C -0.78508 4.10789 0.42684
C -0.04329 2.93643 0.18762
C -0.63676 1.65981 0.25188
H -3.85538 2.72625 0.95838
Ru 0.27900 -0.23447 -0.17952
N 0.39086 -0.99312 1.68194
C 0.40359 -1.50735 2.73171
C 0.43023 -2.14525 4.03766
N 0.01197 0.36153 -2.08596
C -0.23702 0.65783 -3.19021
C -0.54018 1.02374 -4.56343
H 0.24437 0.64082 -5.23560
H -1.50920 0.58919 -4.85818
H -0.59420 2.12021 -4.65686
H -4.68875 0.77875 0.95230
H -5.49438 -1.58015 0.73978
H -3.82411 -3.38348 0.09392
H -1.44143 -2.72612 -0.29820
H -2.73205 4.94717 0.91992
H -0.28092 5.07903 0.37995
H 1.01895 3.04429 -0.05287
H 1.10565 -3.01552 4.01555
H 0.78645 -1.43089 4.79721
H -0.58311 -2.48254 4.30855
Br 1.05208 -2.53226 -1.06414
C 1.82621 0.88604 0.39124
C 2.33134 1.13822 1.68661
C 2.66121 0.96155 -0.75203
C 3.71368 1.24919 1.85364
C 4.06165 1.00558 -0.55034
C 4.58122 1.15666 0.73853
H 1.65854 1.20869 2.54453
H 2.26750 0.97128 -1.76914
H 4.12449 1.40325 2.85627
H 4.72075 1.00086 -1.42389
H 5.66124 1.25609 0.88282

45
23e (triplet)
SCF(BP86)= -1084.26276553
G(298 K) = -1083.981477
SCF(PBE) = -3643.74166744
Low Freq. = 28.4892cm-1,
31.6649cm-1
N -1.99893 -0.59859 -0.00798
C -2.73666 0.55501 -0.02313
C -4.14629 0.50858 0.00485
C -4.79763 -0.72786 0.04514
C -4.02914 -1.90151 0.05559
C -2.63325 -1.79068 0.02955
C -1.93900 1.78716 -0.08007
C -2.52253 3.06913 -0.12644
C -1.71798 4.21320 -0.21102
C -0.31369 4.08829 -0.25573
C 0.28259 2.82402 -0.20661
C -0.50486 1.64515 -0.10107
H -3.60964 3.18546 -0.10325
Ru 0.23914 -0.24007 0.00759
N 0.14010 -0.19274 2.02597
C 0.00353 -0.20703 3.18733
C -0.15482 -0.22448 4.63041
N 0.20946 -0.30286 -2.01531
C 0.10167 -0.33261 -3.17919
C -0.01904 -0.37548 -4.62562
H 0.87422 -0.85022 -5.06257
H -0.91353 -0.95260 -4.91009
H -0.11123 0.64973 -5.01927
H -4.72798 1.43266 -0.00573
H -5.89007 -0.77377 0.06685
H -4.49240 -2.89067 0.08407
H -1.98640 -2.67257 0.04039
H -2.18374 5.20233 -0.24955
H 0.31364 4.98221 -0.33109
H 1.37134 2.74315 -0.24239
H 0.72214 -0.70072 5.09762
H -0.24664 0.80803 5.00480
H -1.06240 -0.78768 4.90099
Br 0.67376 -2.76754 0.08723
C 2.19646 0.31228 0.02662
C 2.77887 1.00970 1.13115
C 3.07118 -0.03072 -1.05055
C 4.14161 1.32995 1.16262
C 4.43714 0.27340 -1.01145
C 4.97998 0.96002 0.09296
H 2.15009 1.31918 1.97092
H 2.68090 -0.57387 -1.91530
H 4.55457 1.86845 2.02220
H 5.08401 -0.02243 -1.84424
H 6.04623 1.20675 0.11796

45
23f
SCF(BP86)= -1084.25192642
G(298 K) = -1083.969457
SCF(PBE) = -3643.73578817
Low Freq. = 28.6636cm-1,
29.6080cm-1
N -1.74605 0.70211 0.20247
C -2.59097 -0.37115 0.36185
C -3.95478 -0.12833 0.63477
C -4.42766 1.17961 0.73525
C -3.53573 2.25646 0.56764
C -2.19332 1.98018 0.31990
C -1.94550 -1.65985 0.18096
C -2.63927 -2.89346 0.21335
C -1.96400 -4.07587 -0.09692

C -0.60363 -4.02099 -0.46059
C   0.09041 -2.79911 -0.47341
C -0.55152 -1.59044 -0.12497
H -3.70531 -2.92450 0.46099
Ru 0.23642 0.30305 -0.18704
N   0.95457 2.37026 -0.46951
C   1.32989 3.46270 -0.66380
C   1.79441 4.81887 -0.90594
N   0.23491 0.81545 1.82274
C   0.22201 1.15223 2.94211
C   0.21276 1.57088 4.33399
H   1.23045 1.49802 4.74994
H -0.13497 2.61360 4.40953
H -0.46197 0.92189 4.91493
H -4.63118 -0.97587 0.76551
H -5.48442 1.36544 0.94644
H -3.86598 3.29533 0.63194
H -1.44779 2.76706 0.19778
H -2.49390 -5.03289 -0.07827
H -0.07705 -4.94064 -0.73855
H   1.14410 -2.79625 -0.76569
H   2.65074 5.04295 -0.24993
H   2.10623 4.92464 -1.95725
H   0.98320 5.53440 -0.69622
Br 0.03427 0.10307 -2.62037
C   1.88692 -0.58042 0.62061
C   2.05130 -1.64646 1.51611
C   2.98014 -0.01713 -0.07042
C   3.31121 -2.25744 1.61548
C   4.22115 -0.69388 -0.01412
C   4.38872 -1.79730 0.83261
H   1.20691 -2.01395 2.10532
H   2.90112 0.90109 -0.65858
H   3.44681 -3.09983 2.30127
H   5.06242 -0.29583 -0.59053
H   5.36942 -2.27540 0.91714

45
23f (triplet)
SCF(BP86)= -1084.25379662
G(298 K) = -1083.974021
SCF(PBE) = -3643.73262028
Low Freq. = 14.9927cm-1,
26.7080cm-1
N -1.84845 0.90340 0.00637
C -2.76326 -0.11878 0.05001
C -4.13824 0.17092 0.17215
C -4.56353 1.50070 0.25112
C -3.61132 2.53216 0.20653
C -2.26112 2.18683 0.08653
C -2.18684 -1.46928 -0.04749
C -2.98196 -2.63370 -0.08707
C -2.38469 -3.89336 -0.22815
C -0.98290 -4.00384 -0.34422
C -0.18053 -2.85676 -0.31417
C -0.75218 -1.57019 -0.14547
H -4.07192 -2.56684 -0.02031
Ru 0.23979 0.16877 -0.05502
N   1.06958 2.21857 0.01021
C   1.49320 3.30830 0.06433
C   2.02457 4.66003 0.12519
N   0.02934 0.24201 1.98767
C -0.13653 0.33302 3.14196
C -0.33773 0.43804 4.57547
H   0.46453 1.04686 5.02230
H -1.31182 0.91017 4.78119
H -0.32220 -0.56820 5.02484
H -4.86922 -0.63974 0.20644
H -5.62860 1.73072 0.34518
H -3.90136 3.58409 0.25904
H -1.47678 2.94673 0.04503
H -3.01061 -4.79020 -0.25804
H -0.52062 -4.98938 -0.46179
H   0.90439 -2.95664 -0.40624
H   2.94738 4.66953 0.72703
H   2.25467 5.01496 -0.89219
H   1.28541 5.33473 0.58562
Br 0.27234 0.30755 -2.52190
C   2.14072 -0.50398 0.23608
C   2.45195 -1.48939 1.22099
C   3.22086 -0.00249 -0.55283
C   3.75042 -1.99467 1.36211
C   4.51728 -0.51220 -0.41518
C   4.78945 -1.50934 0.54354
H   1.66135 -1.87657 1.87174
H   3.02782 0.77243 -1.30009
H   3.95764 -2.76350 2.11386
H   5.32197 -0.13138 -1.05314
H   5.80644 -1.89776 0.65864

45
23g
SCF(BP86)= -1084.25581715
G(298 K) = -1083.973660
SCF(PBE) = -3643.73373942
Low Freq. = 22.5265cm-1,
36.0421cm-1
N   1.05711 0.37564 1.33123
C   2.37946 0.18813 0.99337
C   3.39132 0.43235 1.94634
C   3.04946 0.86023 3.23031
C   1.69261 1.04522 3.55362
C   0.72632 0.79630 2.57704
C   2.57138 -0.27042 -0.37631
C   3.82171 -0.63746 -0.91257
C   3.90275 -1.10439 -2.23080
C   2.73675 -1.20756 -3.02016
C   1.49118 -0.83348 -2.50193
C   1.36364 -0.37757 -1.16170
H   4.73130 -0.57006 -0.30758
Ru -0.31806 0.13259 -0.21768
N -2.12478 0.94507 0.82252
C -3.05293 1.45414 1.32120
C -4.21169 2.07964 1.94023
N -0.00761 2.17702 -0.37525
C   0.18145 3.32893 -0.43528
C   0.41311 4.75981 -0.52446
H -0.37020 5.22867 -1.14105
H   0.39204 5.20030 0.48548
H   1.39846 4.94985 -0.97930
H   4.43850 0.28798 1.67096
H   3.82877 1.04955 3.97349

```
H   1.38049  1.37670  4.54663
H  -0.34058  0.92413  2.77147
H   4.87391 -1.38757 -2.64784
H   2.80824 -1.57711 -4.04844
H   0.60036 -0.89963 -3.13225
H  -4.82447  2.57610  1.17103
H  -4.82134  1.31517  2.44785
H  -3.88443  2.82808  2.67945
Br -1.74246  0.18815 -2.32102
C  -0.85264 -1.56730  0.73710
C   0.07200 -2.42481  1.39570
C  -2.17653 -2.04233  0.49847
C  -0.26643 -3.75622  1.66353
C  -2.50720 -3.37675  0.76921
C  -1.55639 -4.23342  1.35452
H   1.07062 -2.07057  1.66230
H  -2.92270 -1.38131  0.05283
H   0.47005 -4.42084  2.12586
H  -3.51198 -3.74356  0.53690
H  -1.82563 -5.26762  1.59152

45
23g (triplet)
SCF(BP86)= -1084.26031907
G(298 K) = -1083.980347
SCF(PBE) = -3643.73859862
Low Freq. = 26.3516cm-1,
32.2305cm-1
N   0.88314 -0.22631  1.50158
C   2.20130 -0.49668  1.21320
C   3.10370 -0.78221  2.25624
C   2.65187 -0.81633  3.57820
C   1.29596 -0.56396  3.84825
C   0.44311 -0.26593  2.78414
C   2.50581 -0.48088 -0.21708
C   3.78432 -0.74896 -0.74583
C   3.98670 -0.73084 -2.13258
C   2.91222 -0.43980 -2.99673
C   1.64039 -0.15158 -2.48188
C   1.39764 -0.18122 -1.08462
H   4.62675 -0.96872 -0.08295
Ru -0.33354  0.24955 -0.14766
N  -1.92675  1.05628  1.11404
C  -2.74848  1.60004  1.74617
C  -3.77382  2.26824  2.52875
N   0.43764  2.34152  0.06789
C   0.83930  3.43256  0.19355
C   1.33347  4.79126  0.34527
H   0.55223  5.50778  0.04555
H   1.60665  4.97242  1.39709
H   2.22211  4.93773 -0.28915
H   4.15295 -0.97993  2.02665
H   3.34852 -1.03913  4.39102
H   0.89845 -0.58738  4.86525
H  -0.61523 -0.04955  2.93747
H   4.97961 -0.94219 -2.54026
H   3.06915 -0.43716 -4.08045
H   0.81607  0.06315 -3.16724
H  -4.28604  3.02084  1.90830
H  -4.50957  1.53045  2.88687
H  -3.31226  2.76717  3.39610
Br -1.57860  0.94869 -2.18073
C  -1.08584 -1.62097 -0.11960
C  -0.25928 -2.78282 -0.10349
C  -2.49887 -1.81654 -0.07643
C  -0.81506 -4.06745 -0.05514
C  -3.05137 -3.10236 -0.02864
C  -2.21283 -4.23444 -0.01643
H   0.82886 -2.67678 -0.13563
H  -3.16857 -0.95171 -0.09723
H  -0.15732 -4.94297 -0.04999
H  -4.13928 -3.22477 -0.00570
H  -2.64590 -5.23903  0.02051

45
22a(5)
SCF(BP86)= -1084.27665757
G(298 K) = -1083.993190
SCF(PBE) = -3643.76112184
Low Freq. = 6.8735cm-1, 12.0802cm-
1
N  -1.80063  0.07031  1.41545
C  -3.03801 -0.11571  0.83771
C  -4.18807 -0.14597  1.65064
C  -4.07253  0.01219  3.03535
C  -2.79991  0.20139  3.60271
C  -1.68774  0.22478  2.75788
C  -2.97913 -0.26495 -0.61655
C  -4.11221 -0.46176 -1.43750
C  -3.96240 -0.59478 -2.82309
C  -2.67588 -0.53014 -3.39153
C  -1.54207 -0.33398 -2.58156
C  -1.66335 -0.19717 -1.18237
H  -5.11306 -0.51154 -0.99445
Ru -0.20286  0.08714  0.14771
N  -0.34569  2.06050 -0.02374
C  -0.43121  3.22907 -0.11105
C  -0.52524  4.67765 -0.21360
N   0.03146 -1.86838  0.40336
C   0.17160 -3.02171  0.57839
C   0.35508 -4.44784  0.80303
H   0.58581 -4.95564 -0.14787
H   1.18661 -4.61713  1.50700
H  -0.56351 -4.88609  1.22620
H  -5.16852 -0.29396  1.19117
H  -4.96463 -0.01117  3.66768
H  -2.66359  0.32980  4.67895
H  -0.67188  0.36978  3.13694
H  -4.84030 -0.74766 -3.45818
H  -2.55158 -0.63297 -4.47540
H  -0.55587 -0.28999 -3.05882
H  -0.04168  5.02563 -1.14092
H  -1.58273  4.98813 -0.22620
H  -0.02685  5.15243  0.64762
Br  1.82224  0.10825 -1.45911
C   3.38508  0.07898 -0.27834
C   3.62588  1.18673  0.54461
C   4.21176 -1.05096 -0.29886
C   4.75084  1.14952  1.38804
C   5.33317 -1.06243  0.55076
C   5.60098  0.03079  1.39138
H   2.96156  2.05452  0.53030
```

H   3.99361 -1.89867 -0.95349
H   4.95920 2.00474 2.03835
H   5.99428 -1.93439 0.54963
H   6.47500 0.01188 2.04909

45
22b(5)
SCF(BP86)= -1084.27512520
G(298 K) = -1083.991714
SCF(PBE) = -3643.76061003
Low Freq. = 6.1192cm-1, 12.5186cm-1
C   1.87007 0.01786 -0.82202
C   2.02793 1.43682 -0.95721
C   2.98074 1.97140 -1.85364
C   3.78838 1.11803 -2.61556
C   3.64706 -0.27692 -2.48220
C   2.70213 -0.82178 -1.59376
C   1.14581 2.25034 -0.11588
C   1.10617 3.65690 -0.04633
C   0.20930 4.28926 0.82111
C -0.64525 3.50736 1.61821
C -0.57016 2.11612 1.51067
N   0.29460 1.50057 0.66727
H   1.77998 4.24897 -0.67062
Ru 0.45784 -0.55198 0.46427
N   0.61380 -2.53322 0.23454
C   0.66701 -3.70382 0.13674
C   0.74114 -5.15179 0.00499
N   1.72742 -0.59537 1.94866
C   2.47956 -0.61207 2.85156
C   3.40099 -0.63630 3.97810
H   4.35398 -0.15813 3.69791
H   2.96996 -0.09124 4.83402
H   3.60206 -1.67635 4.28293
H   3.09328 3.05629 -1.95751
H   4.52539 1.53520 -3.30861
H   4.27933 -0.94824 -3.07445
H   2.61084 -1.90904 -1.50551
H   0.17709 5.38117 0.87779
H -1.35805 3.96016 2.31131
H -1.20848 1.45374 2.10337
H   1.67462 -5.44119 -0.50548
H   0.71958 -5.62612 0.99995
H -0.11402 -5.52351 -0.58293
Br -1.25679 -0.50369 -1.45791
C -2.98940 -0.21055 -0.60343
C -3.53297 -1.23795 0.17867
C -3.63578 1.01374 -0.81358
C -4.78475 -1.01601 0.78016
C -4.88936 1.20910 -0.20541
C -5.46041 0.20106 0.58902
H -3.00278 -2.18384 0.31579
H -3.18222 1.79392 -1.43001
H -5.22865 -1.80468 1.39536
H -5.41388 2.15733 -0.35770
H -6.43506 0.36328 1.05869

45
TS(22b-23a)
SCF(BP86)= -1084.23357627
G(298 K) = -1083.950704
SCF(PBE) = -3643.71854177
Low Freq. = -175.9224cm-1, 25.4396cm-1
C -1.85905 1.14855 0.00752
C -2.94253 0.22202 0.09497
C -4.26948 0.69177 0.21435
C -4.52808 2.06648 0.28786
C -3.46138 2.98224 0.24425
C -2.13707 2.52609 0.11975
C -2.59301 -1.20027 0.01564
C -3.51079 -2.26494 0.10656
C -3.07281 -3.58568 -0.02128
C -1.71016 -3.82654 -0.25401
C -0.84128 -2.73582 -0.32898
N -1.25097 -1.45018 -0.18240
H -4.56864 -2.05142 0.27438
Ru -0.04930 0.28398 -0.29442
N   0.82682 2.07025 -0.55623
C   1.28808 3.13139 -0.74962
C   1.85631 4.45000 -0.98537
N   0.05203 0.38364 1.64607
C   0.14330 0.41279 2.81342
C   0.26366 0.44104 4.26294
H -0.73640 0.39616 4.72380
H   0.85548 -0.42245 4.60761
H   0.76441 1.36903 4.58327
H -5.10660 -0.01280 0.24882
H -5.55789 2.42255 0.38763
H -3.65812 4.05739 0.31567
H -1.32341 3.25586 0.10792
H -3.78439 -4.41303 0.05045
H -1.31561 -4.83729 -0.38027
H   0.21831 -2.88283 -0.54180
H   1.25579 5.21761 -0.47058
H   2.88998 4.49051 -0.60484
H   1.86476 4.66870 -2.06557
Br 1.31208 -0.75955 -2.10905
C   2.28600 -0.55705 -0.00981
C   3.34386 0.36333 -0.07805
C   2.34733 -1.69764 0.80496
C   4.44429 0.18748 0.77859
C   3.45786 -1.85333 1.65688
C   4.50417 -0.91722 1.64647
H   3.32038 1.20263 -0.77570
H   1.54759 -2.43865 0.83135
H   5.26310 0.91365 0.74611
H   3.49466 -2.72247 2.32179
H   5.37270 -1.05813 2.29674

45
TS(22a-23b)
SCF(BP86)= -1084.24235061
G(298 K) = -1083.959881
SCF(PBE) = -3643.72651128
Low Freq. = -160.9571cm-1, 25.4715cm-1
N -0.58259 1.48706 -0.06986
C -1.92736 1.81307 -0.07762
C -2.33831 3.15776 -0.12822
C -1.38981 4.18165 -0.17294

C   -0.02960 3.83813 -0.16813
C   0.33300 2.48956 -0.11650
C   -2.80520 0.65221 -0.03389
C   -4.21802 0.66623 -0.03264
C   -4.91889 -0.54568 0.00532
C   -4.21808 -1.76736 0.03873
C   -2.80876 -1.78997 0.03558
C   -2.10563 -0.57464 0.00554
H   -4.76646 1.61377 -0.06069
Ru -0.08323 -0.56322 0.03400
N   -0.13594 -0.70515 -1.96219
C   -0.24470 -0.79867 -3.12534
C   -0.37606 -0.91159 -4.56964
N   -0.14458 -0.46801 2.03191
C   -0.24271 -0.43261 3.19922
C   -0.35774 -0.39051 4.64860
H   -0.40786 -1.41519 5.05121
H   0.51603 0.12197 5.08283
H   -1.27189 0.15279 4.93751
H   -3.40700 3.38556 -0.13279
H   -1.70448 5.22823 -0.21157
H   0.75628 4.59609 -0.20301
H   1.37891 2.19245 -0.11038
H   -6.01298 -0.54297 0.00693
H   -4.76868 -2.71392 0.06580
H   -2.27618 -2.74774 0.05985
H   -0.74629 -1.91469 -4.83681
H   -1.08822 -0.15706 -4.94120
H   0.60124 -0.75027 -5.05270
Br   1.65408 -2.27886 0.14742
C   2.30130 0.03133 -0.02006
C   2.85068 0.33916 -1.27310
C   2.86495 0.49224 1.17899
C   3.92053 1.25301 -1.32519
C   3.93494 1.40455 1.10505
C   4.45884 1.78857 -0.14182
H   2.44478 -0.08770 -2.19269
H   2.46969 0.18269 2.14899
H   4.33379 1.53326 -2.29972
H   4.36017 1.80221 2.03241
H   5.30016 2.48640 -0.19043
C   2.01085 3.61925 0.03208
C   3.28002 3.22529 0.48761
C   3.51912 1.86763 0.70780
Ru -0.25499 -0.20882 -0.27897
N   -0.75575 -0.96913 1.53103
C   -1.07048 -1.44706 2.55070
C   -1.46882 -2.05787 3.80948
Br   0.25511 0.37100 -2.67635
H   4.73807 -0.70034 1.04696
H   4.50258 1.51788 1.03127
H   4.07175 3.96221 0.64987
H   1.77791 4.66451 -0.18495
H   0.02585 2.91938 -0.49587
H   4.72252 -3.18942 0.95856
H   2.60697 -4.41669 0.42335
H   0.46477 -3.15044 0.05899
H   -1.99525 -3.00618 3.61376
H   -0.57996 -2.26296 4.42764
H   -2.14092 -1.38152 4.36170
O   -1.70620 -1.39059 -1.04791
C   -2.20852 -2.57412 -0.70532
O   -1.63748 -3.42770 -0.01017
C   -3.59061 -2.80721 -1.30971
H   -4.30991 -2.08698 -0.88433
H   -3.56700 -2.64038 -2.39873
H   -3.93049 -3.83064 -1.09802
C   -1.43473 1.28206 0.51959
C   -1.33885 1.88860 1.78240
C   -2.46286 1.65360 -0.37284
C   -2.27119 2.87363 2.15823
C   -3.39271 2.63921 0.01990
C   -3.30086 3.25082 1.27999
H   -0.53713 1.60828 2.47227
H   -2.54978 1.19553 -1.36213
H   -2.18605 3.34278 3.14455
H   -4.18717 2.92379 -0.67883
H   -4.02477 4.01739 1.57399

46
25a
SCF(BP86)= -1180.16443080
G(298 K) = -1179.878753
SCF(PBE) = -3739.60536988
Low Freq. = 31.9792cm-1,
38.0116cm-1
C   1.43453 -1.21717 0.24853
C   2.62055 -0.51506 0.55872
C   3.80956 -1.23299 0.81678
C   3.80160 -2.63249 0.76026
C   2.61187 -3.32175 0.45737
C   1.41101 -2.62040 0.22970
C   2.48869 0.93204 0.49015
N   1.23017 1.33088 0.10980
C   1.02140 2.64910 -0.14381

46
25a (triplet)
SCF(BP86)= -1180.13370771
G(298 K) = -1179.851041
SCF(PBE) = -3739.57199794
Low Freq. = 31.1530cm-1,
33.5538cm-1
C   1.84861 -0.79975 -0.05316
C   2.75327 0.26786 0.19352
C   4.13722 0.01308 0.32151
C   4.61645 -1.30010 0.22353
C   3.72124 -2.36302 -0.00287
C   2.34171 -2.11708 -0.12639
C   2.15749 1.60726 0.23744
N   0.79365 1.63333 0.04399

```
C   0.16325  2.83367 -0.03653
C   0.83303  4.05008  0.11717
C   2.21425  4.03628  0.36447
C   2.87466  2.80642  0.41803
Ru -0.17725 -0.24634 -0.21518
N  -0.10853 -0.58166  1.78960
C  -0.02690 -0.72203  2.94728
C   0.06252 -0.93336  4.38352
Br -0.18014  0.21728 -2.67134
H   4.84410  0.83168  0.49245
H   3.95323  2.76626  0.58595
H   2.76986  4.96866  0.49917
H   0.27054  4.98360  0.04095
H  -0.90976  2.79641 -0.22545
H   5.68832 -1.49631  0.32543
H   4.09842 -3.38891 -0.07892
H   1.65167 -2.95338 -0.26761
H   0.00706 -2.01138  4.60646
H   1.01583 -0.53537  4.76679
H  -0.76949 -0.41945  4.89137
O  -0.93751 -2.05626 -0.69208
C  -1.11332 -3.14443  0.06618
O  -0.52387 -3.38720  1.12556
C  -2.12409 -4.10490 -0.55528
H  -3.11516 -3.62328 -0.60674
H  -1.83041 -4.35598 -1.58748
H  -2.19449 -5.02305  0.04459
C  -2.06842  0.61090  0.27638
C  -2.34718  1.40314  1.41652
C  -3.14385  0.31106 -0.60323
C  -3.63853  1.89729  1.66432
C  -4.43742  0.80039 -0.35364
C  -4.68951  1.59587  0.77906
H  -1.54559  1.64776  2.12268
H  -2.96765 -0.30655 -1.48948
H  -3.82570  2.51301  2.55109
H  -5.25020  0.55909 -1.04818
H  -5.69833  1.97565  0.97182
```

46
25b
SCF(BP86)= -1180.16346054
G(298 K) = -1179.877907
SCF(PBE) = -3739.60379359
Low Freq. = 24.4368cm-1,
28.2830cm-1

```
C   1.89772 -0.46535  0.18735
C   2.65801  0.72774  0.24605
C   4.06345  0.66090  0.37641
C   4.69996 -0.58392  0.44366
C   3.94082 -1.76717  0.38073
C   2.53827 -1.71498  0.27401
C   1.88472  1.95175  0.08107
N   0.54420  1.71941 -0.10241
C  -0.26575  2.76277 -0.41251
C   0.20070  4.07718 -0.49446
C   1.55869  4.33429 -0.24502
C   2.40519  3.25983  0.03709
Ru -0.11087 -0.32273 -0.12739
N   0.03312 -1.00499  1.79130
C   0.11575 -1.46939  2.86162
C   0.21805 -2.05116  4.19171
Br -0.87602 -2.70345 -0.58414
H   4.65808  1.57932  0.41411
H   3.47265  3.42053  0.20613
H   1.95269  5.35370 -0.28572
H  -0.49885  4.87830 -0.74461
H  -1.31547  2.52284 -0.58680
H   5.78832 -0.63423  0.54421
H   4.43760 -2.74194  0.43003
H   1.95518 -2.63889  0.25155
H  -0.20810 -3.06747  4.19211
H   1.27503 -2.10609  4.49828
H  -0.33365 -1.43203  4.91736
O  -0.00161  0.05585 -2.11200
C  -0.87221 -0.20157 -3.08524
O  -2.08669 -0.40509 -2.93650
C  -0.21207 -0.19208 -4.46138
H   0.39913  0.71481 -4.59425
H   0.46466 -1.05927 -4.54975
H  -0.97526 -0.24796 -5.25015
C  -1.70214  0.56904  0.83911
C  -2.84169  0.43085  0.02608
C  -1.76364  1.23866  2.06908
C  -4.06817  0.96179  0.48088
C  -2.99396  1.77040  2.50268
C  -4.14816  1.62739  1.71516
H  -2.77929 -0.04120 -0.96642
H  -0.86921  1.35976  2.68737
H  -4.95744  0.85410 -0.15024
H  -3.04068  2.29762  3.46190
H  -5.10303  2.04003  2.05631
```

46
25b (triplet)
SCF(BP86)= -1180.13115485
G(298 K) = -1179.848231
SCF(PBE) = -3739.56895491
Low Freq. = 19.0912cm-1,
31.9355cm-1

```
C   1.99767 -0.63202  0.10525
C   2.81160  0.47122 -0.26963
C   4.20581  0.31986 -0.45644
C   4.80295 -0.92835 -0.25087
C   4.01284 -2.02399  0.14805
C   2.62610 -1.87817  0.32840
C   2.10745  1.74328 -0.40724
N   0.74610  1.67464 -0.19495
C   0.03102  2.83013 -0.18044
C   0.60197  4.08086 -0.42226
C   1.97751  4.15728 -0.69243
C   2.72793  2.98145 -0.67585
Ru -0.08933 -0.29019  0.12513
N   0.01396  0.10078  2.11323
C   0.11971  0.33477  3.25560
C   0.24968  0.62097  4.67495
Br -0.77641 -2.60977  0.63907
H   4.82640  1.16959 -0.75927
H   3.80513  3.01254 -0.85354
H   2.45796  5.11839 -0.89696
H  -0.03154  4.97071 -0.39925
H  -1.03504  2.72739  0.02114
```

H   5.88157 -1.04810 -0.39246
H   4.47994 -3.00015 0.32162
H   2.03201 -2.74421 0.63552
H -0.13414 -0.22643 5.26554
H   1.30754 0.79199 4.93160
H -0.33113 1.52335 4.92629
O -0.07471 -0.48842 -1.87917
C -0.90331 -1.19958 -2.65497
O -1.92879 -1.78040 -2.28458
C -0.42279 -1.22105 -4.10494
H -0.24443 -0.19503 -4.46587
H   0.53577 -1.76261 -4.17421
H -1.16690 -1.71721 -4.74348
C -2.04523 0.61160 0.11785
C -2.57795 1.19849 -1.05614
C -2.89870 0.48822 1.23937
C -3.92388 1.59975 -1.12390
C -4.24881 0.88218 1.17101
C -4.76575 1.44196 -0.00936
H -1.94013 1.33518 -1.93629
H -2.52683 0.05358 2.17254
H -4.31230 2.03774 -2.05063
H -4.89372 0.75257 2.04797
H -5.81245 1.76052 -0.05776

46
25c
SCF(BP86)= -1180.15952848
G(298 K) = -1179.874176
SCF(PBE) = -3739.60095308
Low Freq. = 32.3775cm-1,
32.8809cm-1
C   1.63532 -0.93952 -0.24884
C   2.62800 0.04733 -0.47683
C   3.95230 -0.35476 -0.75897
C   4.27235 -1.71763 -0.82065
C   3.27847 -2.68984 -0.60219
C   1.95026 -2.30485 -0.33952
C   2.18743 1.43446 -0.32343
N   0.87636 1.54963 0.07781
C   0.38402 2.77974 0.37119
C   1.14292 3.94568 0.23430
C   2.46513 3.84421 -0.22667
C   2.98973 2.57706 -0.49972
Ru -0.18210 -0.24266 0.32159
N -1.08814 -1.99682 0.68897
C -1.60364 -2.99667 1.01735
C -2.24846 -4.25149 1.37053
Br 0.12204 0.06498 2.77433
H   4.73545 0.39330 -0.91935
H   4.02280 2.46548 -0.83758
H   3.08241 4.73753 -0.35739
H   0.69492 4.91019 0.48489
H -0.64825 2.80965 0.72296
H   5.29960 -2.02427 -1.03997
H   3.53133 -3.75432 -0.64991
H   1.17768 -3.06566 -0.20256
H -1.50630 -4.95448 1.78298
H -3.02906 -4.07698 2.12867
H -2.71062 -4.70196 0.47686
O -0.11797 -0.56075 -1.83180
C -0.90400 -1.37192 -2.51623
O -1.85913 -2.03835 -2.08848
C -0.48471 -1.42910 -3.99350
H -0.31649 -0.41737 -4.39519
H   0.46455 -1.98377 -4.08724
H -1.25794 -1.94156 -4.58386
C -1.78270 0.75995 -0.45849
C -2.88424 0.60407 0.41079
C -1.84463 1.65921 -1.53482
C -4.01818 1.42597 0.23623
C -2.99428 2.45044 -1.71266
C -4.07838 2.34380 -0.82382
H -2.88015 -0.13309 1.21947
H -1.00343 1.74092 -2.22784
H -4.86438 1.31216 0.92268
H -3.03596 3.15400 -2.55147
H -4.97201 2.95958 -0.96701

46
25c (triplet)
SCF(BP86)= -1180.12922202
G(298 K) = -1179.846485
SCF(PBE) = -3739.56895180
Low Freq. = 24.5902cm-1,
25.8644cm-1
C   1.82426 -0.70722 0.15377
C   2.69482 0.39239 -0.09377
C   4.09858 0.21424 -0.09043
C   4.63822 -1.05627 0.14648
C   3.78400 -2.15101 0.38027
C   2.38696 -1.97901 0.36645
C   2.04754 1.69179 -0.30878
N   0.67325 1.68730 -0.18858
C -0.02329 2.83893 -0.35963
C   0.59871 4.05233 -0.65732
C   1.99748 4.07694 -0.78863
C   2.71860 2.89351 -0.61464
Ru -0.20528 -0.17494 0.22384
N -0.94866 -2.01007 0.59372
C -1.39675 -3.07280 0.79214
C -1.94040 -4.39860 1.03183
Br -0.21230 0.32374 2.64066
H   4.77275 1.05984 -0.26328
H   3.80600 2.89259 -0.71686
H   2.51915 5.00888 -1.02517
H -0.00729 4.95207 -0.78588
H -1.10693 2.75657 -0.25301
H   5.72396 -1.19477 0.14852
H   4.20858 -3.14368 0.56859
H   1.73627 -2.84319 0.52855
H -1.35870 -5.14996 0.47350
H -1.88573 -4.63230 2.10765
H -2.99354 -4.44188 0.71047
O -0.09357 -0.53480 -1.78397
C   0.34989 -1.60533 -2.44513
O   0.44232 -2.74564 -1.97618
C   0.70104 -1.27370 -3.89246
H -0.10898 -0.69928 -4.36907
H   1.60756 -0.64483 -3.91365
H   0.88875 -2.19740 -4.45781
C -2.21307 0.44224 -0.34344

C -2.89193 -0.04434 -1.48020
C -2.93013 1.30900 0.52249
C -4.23007 0.30377 -1.74630
C -4.27032 1.65883 0.26069
C -4.92654 1.15915 -0.87563
H -2.36568 -0.70135 -2.18064
H -2.45159 1.71217 1.42244
H -4.72709 -0.09553 -2.63857
H -4.79608 2.32754 0.95297
H -5.96635 1.43497 -1.08198

46
25d
SCF(BP86)= -1180.15817188
G(298 K) = -1179.872259
SCF(PBE) = -3739.60101322
Low Freq. = 31.4541cm-1,
31.8478cm-1
C -1.41187 2.12794 3.73876
C -0.62033 2.73750 2.75350
C -0.30356 2.05249 1.56318
C -0.77428 0.74153 1.34315
C -1.56868 0.13571 2.33575
C -1.88594 0.82316 3.52056
Ru -0.31367 -0.17645 -0.52917
O -1.28685 1.44364 -1.21708
C -2.59774 1.74968 -1.14232
C -2.96345 2.76728 -2.22288
C 0.95281 -0.93385 0.92164
C 2.25366 -0.35973 0.92724
C 3.24486 -0.85846 1.80641
C 2.95256 -1.92685 2.65974
C 1.66982 -2.51110 2.63567
C 0.67287 -2.01024 1.78268
C 2.48471 0.69701 -0.05461
N 1.39761 0.95154 -0.86159
C 1.49758 1.87619 -1.85108
C 2.66718 2.60515 -2.07499
C 3.77843 2.37482 -1.24501
C 3.68356 1.41579 -0.23370
N -1.95514 -1.31455 -0.30188
C -2.90544 -1.99931 -0.27265
C -4.09017 -2.84334 -0.22197
Br 0.61578 -2.08362 -1.91221
O -3.40235 1.30815 -0.32564
H 4.24825 -0.41964 1.81245
H 4.53555 1.21543 0.42027
H 4.70743 2.93409 -1.38880
H 2.69775 3.34012 -2.88296
H 0.59348 2.02744 -2.44396
H 3.72075 -2.31256 3.33689
H 1.44401 -3.36415 3.28502
H -0.31655 -2.47622 1.78006
H -4.20799 -3.38964 -1.17164
H -3.99897 -3.57411 0.59843
H -4.98656 -2.22593 -0.04885
H -2.45233 3.72357 -2.02319
H -2.63555 2.42055 -3.21559
H -4.05077 2.92428 -2.22241
H 0.30758 2.55885 0.81120
H -1.94986 -0.87917 2.19857
H -0.24347 3.75659 2.89908
H -2.50954 0.32895 4.27457
H -1.65697 2.66066 4.66352

46
25d (triplet)
SCF(BP86)= -1180.15386327
G(298 K) = -1179.871063
SCF(PBE) = -3739.59093123
Low Freq. = 18.1035cm-1,
26.0998cm-1
C -1.31073 1.26842 -0.13463
C -2.55029 0.62803 0.19985
C -3.74395 1.37190 0.31480
C -3.72597 2.75700 0.10094
C -2.51819 3.40335 -0.23028
C -1.32723 2.66770 -0.33795
C -2.48055 -0.82381 0.38711
N -1.22510 -1.35757 0.19062
C -1.02379 -2.69477 0.29442
C -2.05954 -3.57348 0.61555
C -3.34624 -3.05003 0.83430
C -3.55499 -1.67273 0.71825
Ru 0.27171 -0.00294 -0.30295
N 1.61806 1.40892 -0.84582
C 2.39853 2.21646 -1.17539
C 3.37701 3.20836 -1.58866
Br -0.21890 -0.38133 -2.86143
H -4.68935 0.87959 0.56531
H -4.54935 -1.24995 0.87863
H -4.17932 -3.71130 1.08947
H -1.85577 -4.64396 0.69288
H 0.00432 -3.02053 0.11195
H -4.65212 3.33331 0.18859
H -2.50975 4.48440 -0.40720
H -0.40097 3.18991 -0.60049
H 3.92337 2.85272 -2.47705
H 2.87606 4.16016 -1.82847
H 4.09632 3.37986 -0.77127
O 1.65420 -1.61670 -0.28741
C 2.97168 -1.60747 -0.15800
O 3.65145 -0.64569 0.23874
C 3.61797 -2.94044 -0.54123
H 3.18253 -3.75728 0.05805
H 3.40962 -3.16779 -1.60032
H 4.70515 -2.90496 -0.38245
C 0.72390 0.37920 1.65620
C 1.00627 -0.71624 2.52304
C 0.77309 1.68778 2.21177
C 1.28639 -0.51242 3.88122
C 1.06956 1.89178 3.56524
C 1.32208 0.79187 4.40937
H 1.01068 -1.73420 2.12305
H 0.56976 2.55384 1.57516
H 1.48751 -1.37280 4.52878
H 1.10133 2.91017 3.96749
H 1.55059 0.95155 5.46812

46
25e
SCF(BP86)= -1180.14961588

G(298 K) = -1179.865045
SCF(PBE) = -3739.58848825
Low Freq. = 25.0093cm-1,
27.1536cm-1
C -1.34181 -0.83249 -1.07747
C -2.58513 -0.36945 -0.52964
C -3.81451 -0.64743 -1.16995
C -3.83085 -1.40191 -2.34788
C -2.61990 -1.88641 -2.88848
C -1.39648 -1.61198 -2.25947
C -2.46577 0.37092 0.72284
N -1.17142 0.51926 1.16301
C -0.92083 1.14283 2.33909
C -1.93883 1.66897 3.13511
C -3.27007 1.54628 2.69488
C -3.53073 0.89286 1.48987
Ru 0.31901 -0.31019 -0.08739
N 2.16058 0.11726 1.04094
C 3.17200 0.22810 1.62106
C 4.43025 0.36304 2.34088
Br 0.05743 -2.17674 1.62831
H -4.75823 -0.28257 -0.75041
H -4.55757 0.77568 1.13542
H -4.09268 1.95081 3.29159
H -1.68867 2.15901 4.07895
H 0.12900 1.19920 2.63480
H -4.78165 -1.62038 -2.84400
H -2.63680 -2.48157 -3.80860
H -0.46536 -1.98952 -2.69464
H 4.29849 0.05480 3.39044
H 5.19812 -0.27501 1.87470
H 4.77024 1.41076 2.31475
O 1.44243 -1.10639 -1.49963
C 2.77475 -1.30726 -1.59389
O 3.36609 -2.14436 -0.91162
C 3.42951 -0.46191 -2.67090
H 2.92320 -0.61845 -3.63747
H 3.32668 0.60682 -2.41778
H 4.49364 -0.72132 -2.76142
C 0.36119 1.67955 -0.60435
C 1.01129 2.70544 0.12021
C -0.21630 2.00158 -1.86210
C 1.14508 3.99351 -0.42115
C -0.04724 3.28715 -2.41212
C 0.62465 4.28834 -1.69502
H 1.42426 2.50495 1.11212
H -0.76280 1.24764 -2.43296
H 1.65999 4.76891 0.15655
H -0.46915 3.50232 -3.40020
H 0.72885 5.29408 -2.11468

46
25e (triplet)
SCF(BP86)= -1180.15456046
G(298 K) = -1179.871928
SCF(PBE) = -3739.59046202
Low Freq. = 24.3548cm-1,
32.4203cm-1
C -1.19100 1.30349 -0.51260
C -2.49889 0.78521 -0.22536
C -3.64160 1.59879 -0.37091
C -3.50240 2.92573 -0.80405
C -2.22450 3.44696 -1.09501
C -1.08302 2.64534 -0.94661
C -2.54688 -0.62096 0.18893
N -1.31545 -1.23530 0.25528
C -1.23234 -2.55090 0.56937
C -2.35976 -3.32379 0.85486
C -3.62303 -2.70924 0.81459
C -3.71437 -1.35573 0.47788
Ru 0.31419 0.00394 -0.24850
N 1.85228 -1.50409 0.20731
C 2.63593 -2.32141 0.50418
C 3.62795 -3.32558 0.85881
Br 0.14546 -1.06298 -2.61787
H -4.64119 1.20933 -0.15241
H -4.68947 -0.86570 0.42983
H -4.52770 -3.28220 1.03723
H -2.24285 -4.38121 1.10272
H -0.22537 -2.97372 0.58360
H -4.39064 3.55508 -0.91677
H -2.12563 4.48341 -1.43574
H -0.08947 3.04943 -1.16510
H 3.35005 -4.29959 0.42521
H 4.61667 -3.02943 0.47280
H 3.68553 -3.42559 1.95459
O 1.62275 1.46320 -0.78938
C 2.91139 1.37632 -1.12822
O 3.51044 0.33912 -1.43703
C 3.58784 2.74626 -1.13338
H 3.07168 3.42296 -1.83447
H 3.52135 3.20323 -0.13174
H 4.64309 2.65103 -1.42550
C 0.45330 0.53328 1.74213
C 0.29256 -0.39972 2.79818
C 0.77635 1.87421 2.08588
C 0.45182 -0.01406 4.13906
C 0.93371 2.25870 3.42534
C 0.77069 1.31728 4.45939
H 0.04730 -1.44355 2.57983
H 0.91434 2.61206 1.29004
H 0.32546 -0.75643 4.93472
H 1.18410 3.29842 3.66403
H 0.89178 1.61940 5.50480

46
25f
SCF(BP86)= -1180.14733108
G(298 K) = -1179.862772
SCF(PBE) = -3739.58280440
Low Freq. = 20.0726cm-1,
33.1712cm-1
C -1.52828 -0.21847 -1.12883
C -2.67984 -0.29809 -0.26563
C -3.98326 -0.41734 -0.79089
C -4.17604 -0.45156 -2.17796
C -3.06710 -0.36417 -3.04427
C -1.76733 -0.25675 -2.52883
C -2.38571 -0.21267 1.16078
N -1.05136 -0.01491 1.43297

```
C  -0.63713  0.11910  2.71625
C  -1.52271  0.06214  3.79427
C  -2.89011 -0.14743  3.53794
C  -3.31954 -0.28487  2.21659
Ru  0.24079 -0.11697 -0.19557
N   2.14940 -0.14913  0.99005
C   3.12515 -0.21756  1.63210
C   4.34808 -0.31597  2.41673
Br  1.63029 -0.39437 -2.28558
H  -4.85091 -0.47753 -0.12557
H  -4.37659 -0.44809  1.99308
H  -3.60923 -0.20169  4.36018
H  -1.14155  0.17730  4.81178
H   0.43483  0.27229  2.85622
H  -5.18788 -0.54137 -2.58549
H  -3.22171 -0.38231 -4.12874
H  -0.91905 -0.19557 -3.21672
H   4.89645 -1.23067  2.13988
H   4.99003  0.55859  2.22462
H   4.10762 -0.35328  3.49121
O   0.20370 -2.05634  0.50406
C   1.13053 -3.00410  0.33998
O   2.27254 -2.88068 -0.10792
C   0.57763 -4.35994  0.80495
H   0.32811 -4.31428  1.87761
H  -0.34843 -4.60489  0.26055
H   1.32821 -5.14356  0.63173
C   0.48471  1.89694  0.10209
C   1.72810  2.51486 -0.20492
C  -0.61983  2.73863  0.40010
C   1.83711  3.91074 -0.29032
C  -0.51035  4.13557  0.30450
C   0.71919  4.72653 -0.03451
H   2.60332  1.89677 -0.41805
H  -1.58557  2.30814  0.67730
H   2.79865  4.36259 -0.55722
H  -1.38620  4.76154  0.50655
H   0.80914  5.81640 -0.08948

46
25f (triplet)
SCF(BP86)= -1180.14870031
G(298 K) = -1179.866611
SCF(PBE) = -3739.58446285
Low Freq. = 22.5621cm-1,
26.2072cm-1
C  -1.44518 -0.23417 -1.13419
C  -2.59248 -0.46688 -0.29868
C  -3.87064 -0.66038 -0.86033
C  -4.03885 -0.60900 -2.25248
C  -2.93142 -0.36305 -3.08615
C  -1.65328 -0.17987 -2.53319
C  -2.34325 -0.46774  1.14894
N  -1.03530 -0.21767  1.49503
C  -0.67174 -0.15171  2.79715
C  -1.58980 -0.33979  3.83346
C  -2.92801 -0.61075  3.50322
C  -3.30428 -0.67423  2.15739
Ru  0.29305 -0.10417 -0.12474
N   2.11158  0.02462  1.11903
C   3.09029  0.05215  1.75993
C   4.31197  0.08162  2.54989
Br  1.76897 -0.09873 -2.17519
H  -4.74115 -0.84647 -0.22293
H  -4.34162 -0.88221  1.88568
H  -3.67296 -0.76872  4.28831
H  -1.25542 -0.27585  4.87134
H   0.38325  0.05521  2.98747
H  -5.03321 -0.75571 -2.68535
H  -3.06675 -0.31576 -4.17214
H  -0.79939  0.00326 -3.19250
H   4.90509 -0.82536  2.35073
H   4.91010  0.96696  2.28069
H   4.06906  0.12458  3.62353
O   0.43832 -2.16619  0.12766
C   1.35514 -3.12216  0.06836
O   2.57503 -2.92617 -0.03629
C   0.75978 -4.52569  0.14280
H   0.14480 -4.62856  1.05201
H   0.09695 -4.69732 -0.72196
H   1.55720 -5.28245  0.14674
C   0.20857  1.96072 -0.02035
C   1.39295  2.73646 -0.15805
C  -0.99937  2.67001  0.22391
C   1.36947  4.13704 -0.06697
C  -1.02507  4.07133  0.31509
C   0.15987  4.81376  0.17218
H   2.34492  2.23491 -0.35593
H  -1.94204  2.12478  0.33338
H   2.30068  4.70194 -0.18828
H  -1.97701  4.58348  0.49514
H   0.14086  5.90631  0.24321

46
25g
SCF(BP86)= -1180.14086929
G(298 K) = -1179.856563
SCF(PBE) = -3739.58243485
Low Freq. = 24.8867cm-1,
29.5088cm-1
C   0.49103 -1.53127  0.38258
C   1.90782 -1.61948  0.21518
C   2.61067 -2.83408  0.39989
C   1.91742 -3.98512  0.78044
C   0.52474 -3.91255  0.99246
C  -0.17344 -2.70980  0.79707
C   2.54322 -0.35981 -0.11184
N   1.65951  0.69365 -0.18711
C   2.08809  1.94716 -0.49727
C   3.43670  2.22245 -0.71156
C   4.36590  1.16687 -0.62811
C   3.91691 -0.11918 -0.32980
Ru -0.29246  0.34831  0.22220
N  -0.99349  2.45152  0.41913
C  -1.36120  3.54791  0.60755
C  -1.81445  4.91074  0.84024
Br -0.19567  0.31327  2.72884
H   3.69614 -2.87375  0.25731
H   4.61955 -0.95329 -0.26536
H   5.43045  1.35178 -0.79808
H   3.74490  3.24464 -0.94204
H   1.31610  2.71453 -0.55957
```

```
H   2.45239 -4.92845  0.92763
H  -0.02264 -4.80516  1.31666
H  -1.25160 -2.69311  0.98172
H  -2.68685  5.12885  0.20368
H  -2.09852  5.03667  1.89720
H  -1.00717  5.62019  0.59717
O  -0.36183  1.02067 -1.81564
C   0.20708  0.39331 -2.83160
O   0.79430 -0.69971 -2.79385
C   0.10510  1.20434 -4.12920
H  -0.81202  1.81132 -4.16252
H   0.96858  1.88922 -4.19311
H   0.14079  0.52513 -4.99383
C  -1.80724 -0.64157 -0.66165
C  -1.91566 -1.75542 -1.50690
C  -2.94780 -0.06604 -0.06122
C  -3.16919 -2.36922 -1.66596
C  -4.18693 -0.73851 -0.17526
C  -4.29915 -1.87928 -0.98208
H  -1.02742 -2.12743 -2.02252
H  -2.90820  0.87870  0.49260
H  -3.25963 -3.24193 -2.32175
H  -5.06615 -0.31554  0.32201
H  -5.27180 -2.36494 -1.10929

46
25g (triplet)
SCF(BP86)= -1180.14818705
G(298 K) = -1179.866142
SCF(PBE) = -3739.58470406
Low Freq. = 28.5948cm-1,
35.1956cm-1
C   0.91484 -1.58489  0.02070
C   2.32649 -1.42605 -0.20036
C   3.16146 -2.55882 -0.31607
C   2.62950 -3.84968 -0.19377
C   1.25474 -4.02014  0.05577
C   0.41525 -2.90095  0.16795
C   2.85836 -0.05117 -0.26657
N   1.92876  0.93322 -0.06412
C   2.30232  2.23000 -0.08359
C   3.62513  2.62936 -0.30486
C   4.59202  1.63352 -0.51785
C   4.20798  0.28827 -0.49832
Ru -0.15318  0.13524  0.10092
N  -1.04206  2.12338  0.35296
C  -1.48047  3.19740  0.50904
C  -2.03554  4.52855  0.70178
Br  0.07304  0.09335  2.60401
H   4.23584 -2.44247 -0.49042
H   4.95273 -0.49364 -0.66321
H   5.63744  1.90217 -0.69639
H   3.88337  3.69108 -0.30877
H   1.50462  2.95855  0.08660
H   3.28744 -4.71992 -0.28152
H   0.83850 -5.02749  0.16537
H  -0.65041 -3.05152  0.36233
H  -3.05614  4.57168  0.28880
H  -2.07388  4.76444  1.77743
H  -1.40860  5.27761  0.19228
O  -0.11954  0.35037 -1.89061
C  -1.01423  0.85658 -2.75330
O  -2.09972  1.35660 -2.44855
C  -0.52066  0.74802 -4.19191
H   0.44752  1.26369 -4.30101
H  -0.35917 -0.31042 -4.45539
H  -1.25554  1.19056 -4.87862
C  -2.02718 -0.67662  0.00704
C  -2.37302 -1.65123 -0.97132
C  -3.05752 -0.27373  0.90365
C  -3.65643 -2.21549 -1.03088
C  -4.33701 -0.84850  0.85984
C  -4.64497 -1.82168 -0.10977
H  -1.62295 -1.96542 -1.70564
H  -2.84183  0.48303  1.66487
H  -3.88828 -2.96246 -1.79868
H  -5.09983 -0.53304  1.58098
H  -5.64647 -2.26292 -0.15139

46
25h
SCF(BP86)= -1180.14160911
G(298 K) = -1179.856702
SCF(PBE) = -3739.58335703
Low Freq. = 27.9855cm-1,
38.6617cm-1
C  -1.33866  0.25006  1.03862
C  -2.40499 -0.62177  0.68252
C  -3.58562 -0.65842  1.46270
C  -3.70054  0.14348  2.60309
C  -2.62765  0.97287  2.98360
C  -1.45738  1.02299  2.20755
C  -2.16138 -1.50113 -0.46120
N  -0.88896 -1.41195 -0.98182
C  -0.52281 -2.21270 -2.01697
C  -1.40336 -3.12704 -2.59691
C  -2.71158 -3.22150 -2.09086
C  -3.08644 -2.40723 -1.01973
Ru  0.40722 -0.00309 -0.13543
N   0.76429 -1.10403  1.46084
C   0.98960 -1.74377  2.41190
C   1.31684 -2.54386  3.57977
Br  2.17134  1.62471  0.87610
H  -4.40675 -1.33012  1.19145
H  -4.09744 -2.46596 -0.60984
H  -3.42912 -3.92258 -2.52664
H  -1.06390 -3.74719 -3.42992
H   0.50936 -2.08892 -2.35383
H  -4.61414  0.11111  3.20447
H  -2.70323  1.58508  3.88896
H  -0.62963  1.66935  2.51762
H   1.44714 -1.89029  4.45751
H   0.51230 -3.26789  3.78534
H   2.25690 -3.08980  3.39748
O   1.93251 -0.90897 -1.16561
C   3.13507 -1.38596 -0.84918
O   3.44644 -1.88207  0.23975
C   4.11237 -1.29078 -2.02118
H   3.65525 -1.67743 -2.94614
H   4.36900 -0.23240 -2.19933
H   5.03017 -1.85168 -1.79416
C  -0.64129  1.66769 -0.65542
```

C -1.09229 1.45073 -1.98584
C -0.60293 2.98963 -0.15054
C -1.32525 2.55579 -2.83733
C -0.89065 4.07073 -0.98916
C -1.23401 3.86017 -2.34302
H -1.25483 0.44805 -2.38580
H -0.32490 3.16959 0.88996
H -1.63204 2.37108 -3.87222
H -0.84127 5.08891 -0.58862
H -1.45880 4.71290 -2.99137

46
25h (triplet)
SCF(BP86)= -1180.15167667
G(298 K) = -1179.869555
SCF(PBE) = -3739.58869035
Low Freq. = 13.3000cm-1,
24.3031cm-1
C   1.48553 -0.37062 -1.01183
C   2.58528 -0.52707 -0.10512
C   3.90048 -0.69816 -0.58539
C   4.14405 -0.73406 -1.96453
C   3.07320 -0.60188 -2.87020
C   1.76279 -0.42780 -2.40071
C   2.23940 -0.49734 1.31716
N   0.89657 -0.32285 1.55709
C   0.43517 -0.30652 2.83201
C   1.28463 -0.45428 3.93117
C   2.66087 -0.62310 3.70755
C   3.13605 -0.64617 2.39333
Ru -0.34097 -0.11023 -0.14126
N -0.57824 -2.32616 -0.22659
C -0.66873 -3.49169 -0.27371
C -0.78904 -4.94103 -0.33392
Br -1.66503 0.11987 -2.23300
H   4.73843 -0.80539 0.11065
H   4.20156 -0.78115 2.19411
H   3.35358 -0.73723 4.54609
H   0.86508 -0.43455 4.93980
H -0.64485 -0.17554 2.93414
H   5.16515 -0.86907 -2.33407
H   3.26263 -0.63426 -3.94856
H   0.94095 -0.32760 -3.11651
H -1.76216 -5.21890 -0.76919
H   0.01649 -5.35687 -0.96001
H -0.71215 -5.36682 0.67917
O -1.94749 -0.10822 1.15639
C -3.20957 -0.53453 1.14677
O -3.70894 -1.24175 0.26283
C -4.00415 -0.06005 2.35956
H -3.52481 -0.41873 3.28569
H -4.00915 1.04181 2.39827
H -5.03725 -0.43180 2.31193
C -0.10943 1.92515 -0.04618
C -0.43868 2.61273 1.14989
C   0.36109 2.70499 -1.13428
C -0.31862 4.01062 1.24945
C   0.49808 4.10025 -1.02968
C   0.15557 4.76296 0.16173
H -0.82823 2.05454 2.00736
H   0.64069 2.21371 -2.07176
H -0.59741 4.51010 2.18466
H   0.87340 4.67022 -1.88761
H   0.25629 5.85057 0.24091

46
25i
SCF(BP86)= -1180.11710371
G(298 K) = -1179.832719
SCF(PBE) = -3739.58030913
Low Freq. = 22.5904cm-1,
23.1479cm-1
C -0.82890 1.60476 -0.36480
C -2.24462 1.60009 -0.51514
C -2.89954 2.75454 -0.99404
C -2.16763 3.90626 -1.31031
C -0.77178 3.92001 -1.14143
C -0.10762 2.78695 -0.64154
C -2.92474 0.34436 -0.18649
N -2.07460 -0.64219 0.22898
C -2.57459 -1.86966 0.48318
C -3.93882 -2.16985 0.37667
C -4.82397 -1.15390 -0.01395
C -4.31085 0.11456 -0.30068
Ru 0.16298 -0.10785 0.09292
N   0.49774 1.00949 1.74553
C   0.73367 1.64051 2.70306
C   1.03212 2.39878 3.91123
Br 0.44614 -1.90949 1.81205
H -3.98679 2.75499 -1.12714
H -4.97535 0.92146 -0.62111
H -5.89767 -1.34859 -0.10186
H -4.28973 -3.18110 0.59962
H -1.84043 -2.61882 0.79388
H -2.68623 4.79581 -1.68214
H -0.19667 4.81973 -1.38656
H   0.97596 2.80873 -0.49813
H   2.05191 2.81604 3.86203
H   0.31618 3.22879 4.03184
H   0.96234 1.74177 4.79434
O -0.05025 -0.99595 -1.66651
C   0.57251 -2.04655 -2.23414
O   1.10281 -2.95913 -1.60556
C   0.50203 -2.00069 -3.75633
H -0.50377 -1.70625 -4.09551
H   1.21421 -1.24492 -4.13057
H   0.77407 -2.98175 -4.17087
C   2.14395 0.19081 -0.30462
C   2.51149 0.56242 -1.62773
C   3.18630 0.06672 0.64998
C   3.84716 0.84228 -1.96162
C   4.52305 0.32020 0.30844
C   4.86069 0.71792 -0.99749
H   1.73849 0.66337 -2.39697
H   2.94731 -0.27111 1.66355
H   4.09593 1.14897 -2.98445
H   5.30807 0.19903 1.06417
H   5.90444 0.91775 -1.26299

46
25i (triplet)
SCF(BP86)= -1180.14961360

G(298 K) = -1179.867020
SCF(PBE) = -3739.58585038
Low Freq. = 20.5550cm-1,
25.3747cm-1
C -0.67868 1.66996 -0.23587
C -2.09827 1.71310 -0.42387
C -2.74281 2.93929 -0.70060
C -2.01149 4.13128 -0.76626
C -0.62345 4.10798 -0.54244
C 0.02908 2.89532 -0.26873
C -2.83488 0.44760 -0.29621
N -2.05889 -0.62704 0.04353
C -2.63746 -1.84069 0.19453
C -4.00955 -2.05127 0.02125
C -4.81693 -0.95742 -0.32851
C -4.22513 0.29872 -0.48705
Ru 0.16284 -0.17554 0.09573
N 0.03460 0.30821 2.07620
C -0.09753 0.59949 3.20194
C -0.25509 0.95087 4.60394
Br 0.64230 -2.59505 0.94235
H -3.82571 2.97066 -0.85561
H -4.83713 1.16066 -0.76174
H -5.89376 -1.08113 -0.47662
H -4.42646 -3.05221 0.15783
H -1.95834 -2.65515 0.46356
H -2.52363 5.07499 -0.97752
H -0.04476 5.03735 -0.57686
H 1.10792 2.90216 -0.09462
H 0.69343 0.79610 5.14297
H -0.55194 2.00845 4.69214
H -1.03613 0.32187 5.06096
O 0.07127 -0.66247 -1.85593
C 0.95338 -1.35078 -2.59089
O 2.00575 -1.85000 -2.17840
C 0.49312 -1.47717 -4.04141
H -0.46015 -2.02979 -4.08867
H 0.31243 -0.47903 -4.47353
H 1.25157 -2.00618 -4.63523
C 2.06708 0.51124 0.02470
C 2.59768 1.10262 -1.16262
C 2.95331 0.38380 1.13512
C 3.93236 1.52009 -1.24139
C 4.29369 0.78474 1.05220
C 4.78893 1.35979 -0.13484
H 1.94651 1.23843 -2.03201
H 2.59102 -0.06402 2.06529
H 4.31028 1.96934 -2.16638
H 4.95603 0.65444 1.91518
H 5.83307 1.68414 -0.19519

46
25j
SCF(BP86)= -1180.14401620
G(298 K) = -1179.859034
SCF(PBE) = -3739.58211046
Low Freq. = 24.8442cm-1,
33.3998cm-1
C -0.73276 1.61708 0.22270
C -2.15620 1.65470 0.21943
C -2.82750 2.89056 0.33249
C -2.10009 4.08227 0.44988
C -0.69478 4.04617 0.46751
C -0.01139 2.81867 0.39590
C -2.82230 0.36471 0.04428
N -1.94386 -0.66634 -0.12673
C -2.42286 -1.89670 -0.40668
C -3.79209 -2.17484 -0.48420
C -4.70511 -1.12939 -0.26578
C -4.21598 0.15350 -0.00444
Ru 0.28513 -0.10217 -0.11146
N 0.67471 0.13009 1.86088
C 0.91664 0.28641 2.99439
C 1.20481 0.45487 4.41053
Br -0.03679 -0.15766 -2.61267
H -3.92152 2.92403 0.32203
H -4.90394 0.98823 0.15088
H -5.78301 -1.31078 -0.30934
H -4.12804 -3.19074 -0.70671
H -1.67146 -2.67171 -0.57256
H -2.62761 5.03690 0.53708
H -0.11930 4.97342 0.55844
H 1.07969 2.81525 0.43334
H 2.29009 0.56271 4.56802
H 0.69637 1.35392 4.79443
H 0.84656 -0.42436 4.97030
O 0.40700 -2.05887 0.30838
C 0.10958 -2.78821 1.38859
O -0.61045 -2.43475 2.32484
C 0.77572 -4.16175 1.30818
H 0.75272 -4.56344 0.28384
H 1.83316 -4.06747 1.60885
H 0.26859 -4.85151 1.99778
C 2.30445 0.22880 -0.33961
C 3.00802 -0.56272 -1.29347
C 3.08097 1.13495 0.43239
C 4.39560 -0.44072 -1.47237
C 4.46245 1.28532 0.23030
C 5.12954 0.49424 -0.72138
H 2.46363 -1.29404 -1.89871
H 2.60510 1.74801 1.20451
H 4.90279 -1.07395 -2.20904
H 5.02071 2.01381 0.82901
H 6.20905 0.60062 -0.87109

46
25j (triplet)
SCF(BP86)= -1180.14617827
G(298 K) = -1179.863642
SCF(PBE) = -3739.58394110
Low Freq. = 26.3518cm-1,
30.4524cm-1
C -0.61840 1.67947 0.23141
C -2.04967 1.70837 0.33797
C -2.72233 2.91954 0.60701
C -2.00340 4.11057 0.77003
C -0.60059 4.10002 0.65724
C 0.08038 2.90088 0.39810
C -2.76160 0.44372 0.10056
N -1.94347 -0.60454 -0.21686

```
C  -2.47773 -1.80894 -0.51441
C  -3.85747 -2.04157 -0.49463
C  -4.71104 -0.98246 -0.14589
C  -4.16033  0.26823  0.15078
Ru  0.23927 -0.14311 -0.14017
N   0.22182 -0.45424  1.86215
C   0.17038 -0.70133  3.00436
C   0.10649 -1.01933  4.42037
Br  0.12981  0.22447 -2.62950
H  -3.81403  2.94152  0.68142
H  -4.81055  1.10519  0.41506
H  -5.79478 -1.12747 -0.11234
H  -4.24543 -3.03148 -0.74709
H  -1.76480 -2.59427 -0.77175
H  -2.53484  5.04417  0.97855
H  -0.03380  5.03021  0.77234
H   1.17013  2.91415  0.31978
H   1.04289 -0.72384  4.92024
H  -0.73833 -0.48871  4.88812
H  -0.04045 -2.10478  4.54276
O   0.69188 -2.15116 -0.38317
C   0.58544 -3.25055  0.36174
O  -0.20333 -3.38664  1.30733
C   1.54164 -4.35358 -0.08073
H   1.42591 -4.55066 -1.15895
H   2.58380 -4.03060  0.08207
H   1.35165 -5.27396  0.48938
C   2.19675  0.47974 -0.00218
C   3.11913  0.20947 -1.05402
C   2.73026  1.08249  1.17379
C   4.48696  0.50167 -0.92836
C   4.09252  1.39835  1.29250
C   4.98100  1.10537  0.24176
H   2.76080 -0.25700 -1.97631
H   2.06566  1.32479  2.00950
H   5.16911  0.26208 -1.75214
H   4.46316  1.86876  2.21027
H   6.04551  1.34547  0.33412

46
25k
SCF(BP86)= -1180.14068308
G(298 K) = -1179.855124
SCF(PBE) = -3739.58289840
Low Freq. = 22.7800cm-1,
35.7643cm-1
C  -0.56292  1.16649  1.01414
C  -1.98012  1.18119  1.09937
C  -2.62715  2.07518  1.98636
C  -1.87487  2.92908  2.79890
C  -0.46852  2.87766  2.74691
C   0.18399  2.00143  1.86308
C  -2.68283  0.19520  0.28572
N  -1.84771 -0.68445 -0.36863
C  -2.39732 -1.63973 -1.16787
C  -3.77557 -1.77462 -1.34378
C  -4.63624 -0.89125 -0.67110
C  -4.08195  0.09734  0.14428
Ru  0.24751 -0.37249 -0.20281
N   0.54805 -1.03428  1.61669
C   0.77661 -1.43505  2.69054
C   1.07927 -1.93981  4.01951
Br  0.68249 -2.89656 -0.84061
H  -3.71965  2.08405  2.05783
H  -4.72612  0.80778  0.66760
H  -5.72110 -0.96826 -0.78707
H  -4.15687 -2.56095 -1.99962
H  -1.68509 -2.30718 -1.65767
H  -2.37946  3.61672  3.48455
H   0.12687  3.52320  3.40224
H   1.27710  1.92966  1.84090
H   2.03672 -1.51981  4.36878
H   0.28184 -1.65468  4.72434
H   1.15894 -3.03869  3.99291
O   2.32927 -0.29102 -0.39482
C   3.25556  0.22199  0.38243
O   3.07610  0.86647  1.43567
C   4.66921 -0.00383 -0.16543
H   4.74499 -0.94404 -0.73193
H   4.92283  0.82517 -0.84956
H   5.39743  0.00058  0.65927
C   0.36962  1.44488 -1.08184
C  -0.50528  1.37729 -2.19549
C   1.41096  2.39830 -1.05601
C  -0.23079  2.16811 -3.33775
C   1.63493  3.20559 -2.17621
C   0.82528  3.08330 -3.32746
H  -1.39313  0.74210 -2.20510
H   2.05485  2.47988 -0.17667
H  -0.89639  2.09285 -4.20405
H   2.45472  3.93175 -2.15871
H   1.00730  3.72385 -4.19598

46
25k (triplet)
SCF(BP86)= -1180.13234520
G(298 K) = -1179.849739
SCF(PBE) = -3739.58989397
Low Freq. = 19.0285cm-1,
28.5057cm-1
C   0.66703  1.14817  1.16653
C   1.96273  1.58139  0.75300
C   2.69795  2.50032  1.53471
C   2.15561  2.99080  2.72664
C   0.87799  2.56539  3.14417
C   0.13673  1.65958  2.37198
C   2.44865  0.97628 -0.48227
N   1.56992  0.07069 -1.04400
C   1.94503 -0.60710 -2.16146
C   3.17933 -0.40640 -2.78141
C   4.07166  0.53370 -2.23917
C   3.69936  1.22311 -1.08439
Ru -0.30754 -0.10941 -0.09917
N  -1.01707  1.73868 -1.12306
C  -1.64178  2.70362 -1.33302
C  -2.48045  3.87647 -1.52656
Br -1.33660 -1.32023 -2.14251
H   3.69398  2.82951  1.21822
H   4.37385  1.95397 -0.63108
H   5.04359  0.71967 -2.70664
H   3.42604 -0.98337 -3.67635
H   1.20622 -1.31307 -2.54876
```

H 2.72523 3.70123 3.33431
H 0.45727 2.94670 4.08139
H -0.85735 1.34484 2.69668
H -3.21157 3.89293 -0.70061
H -1.87695 4.79796 -1.50175
H -3.01309 3.81733 -2.48941
O -1.99650 -0.13674 0.98858
C -3.08017 0.65180 1.02058
O -3.06425 1.88569 1.04048
C -4.36534 -0.16600 1.09160
H -4.46154 -0.77511 0.17665
H -4.32598 -0.86247 1.94500
H -5.23386 0.50051 1.19104
C 0.24935 -1.78810 0.88682
C 1.58453 -2.04436 1.30221
C -0.72520 -2.78743 1.16162
C 1.93181 -3.23960 1.94792
C -0.37772 -3.98559 1.79850
C 0.95263 -4.21818 2.19530
H 2.36196 -1.29270 1.13198
H -1.76208 -2.61535 0.86003
H 2.96800 -3.40766 2.26301
H -1.14623 -4.74357 1.98801
H 1.22303 -5.15378 2.69691

46
24b
SCF(BP86)= -1180.14459162
G(298 K) = -1179.860016
SCF(PBE) = -3739.58749214
Low Freq. = 16.0484cm-1,
18.7280cm-1
C -1.79956 0.09790 -0.89792
C -2.35534 -1.22512 -0.90299
C -3.35229 -1.59128 -1.83579
C -3.82336 -0.65231 -2.76210
C -3.30657 0.66035 -2.74733
C -2.31417 1.03541 -1.82403
C -1.81278 -2.10982 0.12732
N -0.85069 -1.49056 0.90522
C -0.28064 -2.18240 1.92598
C -0.61201 -3.50835 2.21803
C -1.57674 -4.15477 1.42398
C -2.17766 -3.44703 0.37717
Ru -0.33201 0.43473 0.40334
N -1.53231 1.15155 1.73257
C -2.25498 1.56877 2.56554
C -3.13692 2.10820 3.59212
Br 1.29357 -0.50420 -1.37878
H -3.76184 -2.60828 -1.83846
H -2.93362 -3.92533 -0.25190
H -1.85628 -5.19397 1.62072
H -0.11917 -4.01757 3.05007
H 0.46592 -1.63200 2.50715
H -4.59387 -0.93510 -3.48665
H -3.68589 1.40005 -3.46280
H -1.93703 2.06465 -1.79215
H -3.46735 3.12602 3.32428
H -4.02782 1.46841 3.70615
H -2.61416 2.15268 4.56248
O 0.58920 2.27115 0.05506
C 0.10349 3.47309 -0.17063
O -1.08612 3.75090 -0.42067
C 1.17630 4.56905 -0.11328
H 1.62981 4.60574 0.89199
H 1.98858 4.34896 -0.82632
H 0.74039 5.55067 -0.34901
C 3.06833 -0.51892 -0.56059
C 3.86432 -1.66086 -0.71128
C 3.48418 0.61880 0.14278
C 5.14300 -1.65370 -0.12382
C 4.76463 0.59740 0.72514
C 5.59129 -0.53150 0.59278
H 3.50522 -2.53303 -1.26429
H 2.81940 1.48490 0.23170
H 5.78330 -2.53519 -0.22854
H 5.11263 1.47348 1.28177
H 6.58627 -0.53694 1.04809

46
TS(24b-25a')
SCF(BP86)= -1180.13297687
G(298 K) = -1179.848096
SCF(PBE) = -3739.57740826
Low Freq. = -96.8390cm-1,
25.3316cm-1
C 1.91439 0.07952 -0.23552
C 2.20792 1.47735 -0.35629
C 3.47708 1.91830 -0.79719
C 4.47799 0.98680 -1.09716
C 4.21604 -0.38954 -0.94125
C 2.95388 -0.83813 -0.51411
C 1.13138 2.37090 0.07168
N -0.02004 1.69979 0.43746
C -1.02651 2.39527 1.02734
C -0.98303 3.78023 1.21136
C 0.14813 4.48545 0.76584
C 1.21149 3.77170 0.20383
Ru 0.00876 -0.35900 0.16509
N 0.22962 -0.50270 2.07266
C 0.32252 -0.53277 3.24556
C 0.44378 -0.64475 4.69280
Br -0.71780 -0.27662 -2.39141
H 3.68530 2.98908 -0.90328
H 2.11989 4.29105 -0.11265
H 0.20866 5.57230 0.87522
H -1.82547 4.28773 1.68799
H -1.88964 1.80342 1.33973
H 5.46001 1.32782 -1.44029
H 5.00417 -1.12065 -1.15698
H 2.77564 -1.90827 -0.37764
H 0.87782 -1.62170 4.96497
H 1.09672 0.15183 5.08616
H -0.54570 -0.55300 5.17134
O 0.10340 -2.43552 -0.22451
C 0.75313 -3.37711 0.42136
O 1.51298 -3.22431 1.39845
C 0.49086 -4.77449 -0.16180
H -0.57977 -5.02785 -0.07739
H 0.74525 -4.79840 -1.23483
H 1.08403 -5.53260 0.37002
C -2.15487 -0.10693 -1.02858

```
C  -2.93992  1.06934 -1.07119
C  -2.56197 -1.25302 -0.30326
C  -4.08850  1.13085 -0.27423
C  -3.71269 -1.14166  0.50946
C  -4.46929  0.03694  0.53242
H  -2.64295  1.91473 -1.69609
H  -2.01464 -2.19475 -0.38843
H  -4.69426  2.04261 -0.28619
H  -4.02582 -2.01195  1.09537
H  -5.37036  0.10008  1.14945
```

```
46
25a'
SCF(BP86)= -1180.15658853
G(298 K) = -1179.871922
SCF(PBE) = -3739.59751778
Low Freq. = 24.0516cm-1,
34.2595cm-1
C   1.85684 -0.19032 -0.26053
C   2.47039  1.07968 -0.17820
C   3.87118  1.19752 -0.32225
C   4.64651  0.05070 -0.52841
C   4.03135 -1.21496 -0.58655
C   2.63359 -1.34225 -0.48473
C   1.55750  2.15306  0.18363
N   0.28235  1.70087  0.41802
C  -0.62053  2.56144  0.95463
C  -0.32759  3.90438  1.20603
C   0.95046  4.39295  0.88896
C   1.90170  3.50422  0.38334
Ru -0.12352 -0.43120  0.10422
N  -0.34446 -0.34936  2.09453
C  -0.50585 -0.28810  3.25388
C  -0.68970 -0.23711  4.69460
Br  0.07068 -0.74868 -2.48541
H   4.35200  2.17859 -0.25155
H   2.91740  3.83936  0.15934
H   1.20541  5.44391  1.05243
H  -1.09818  4.54877  1.63583
H  -1.60565  2.14957  1.17587
H   5.73133  0.13852 -0.64086
H   4.63698 -2.11332 -0.74659
H   2.16708 -2.32539 -0.57046
H  -0.61871 -1.25107  5.12045
H   0.08698  0.39992  5.14808
H  -1.67960  0.18518  4.93240
O  -0.69336 -2.38163  0.00581
C  -0.19019 -3.25444  0.87137
O   0.79901 -3.03385  1.59116
C  -0.94095 -4.58138  0.88378
H  -1.94716 -4.43512  1.31236
H  -1.07517 -4.95976 -0.14220
H  -0.39605 -5.32125  1.48639
C  -1.89258  0.40628 -0.52477
C  -2.12644  1.44182 -1.44464
C  -2.97554 -0.22160  0.12527
C  -3.44465  1.84788 -1.72101
C  -4.29336  0.19314 -0.16478
C  -4.53217  1.22503 -1.08436
H  -1.28807  1.92804 -1.95045
H  -2.82005 -1.02896  0.84706
H  -3.61435  2.65408 -2.44315
H  -5.12686 -0.30244  0.34515
H  -5.55663  1.54367 -1.30158
```

```
46
24a
SCF(BP86)= -1180.14850054
G(298 K) = -1179.864041
SCF(PBE) = -3739.59171609
Low Freq. = 16.2188cm-1,
23.2376cm-1
C  -1.57063 -0.97557  0.81888
C  -2.90709 -0.70772  0.35877
C  -4.02445 -1.33523  0.95209
C  -3.84996 -2.23771  2.00898
C  -2.54776 -2.51568  2.47158
C  -1.42934 -1.90057  1.88256
C  -2.99252  0.26922 -0.72181
N  -1.75854  0.76767 -1.09563
C  -1.67603  1.69159 -2.08761
C  -2.80445  2.17573 -2.75402
C  -4.06952  1.68235 -2.38668
C  -4.15637  0.72531 -1.36970
Ru -0.15134  0.01842 -0.13527
N   0.00145 -1.42038 -1.45679
C   0.10359 -2.27002 -2.26978
C   0.25718 -3.30816 -3.28164
Br  1.89114 -0.95898  1.12679
H  -5.03628 -1.11373  0.59273
H  -5.12863  0.32562 -1.06904
H  -4.97397  2.03787 -2.88888
H  -2.68553  2.92126 -3.54413
H  -0.66641  2.03445 -2.33348
H  -4.71688 -2.72127  2.47012
H  -2.40147 -3.22092  3.29824
H  -0.43204 -2.14728  2.26819
H   0.37439 -4.29747 -2.80814
H  -0.62855 -3.33858 -3.93796
H   1.14611 -3.11091 -3.90462
O   0.12401  1.69846  1.02596
C  -0.75232  2.52634  1.56550
O  -1.97176  2.32038  1.69541
C  -0.10503  3.82938  2.05223
H   0.29178  4.39690  1.19275
H  -0.84035  4.45083  2.58339
H   0.74606  3.61444  2.71936
C   3.44742 -0.13468  0.26717
C   3.40733  1.24173  0.01066
C   4.53732 -0.94419 -0.07412
C   4.51907  1.82593 -0.62329
C   5.63908 -0.33534 -0.70339
C   5.63025  1.04232 -0.97916
H   2.53093  1.83163  0.29879
H   4.53332 -2.01726  0.13615
H   4.51112  2.90007 -0.83439
H   6.50211 -0.94905 -0.98024
H   6.49044  1.50555 -1.47190
```

```
46
TS(24a-25b')
SCF(BP86)= -1180.11759655
```

G(298 K) = -1179.833662
SCF(PBE) = -3739.56026164
Low Freq. = -169.2482cm-1,
18.2099cm-1

```
C   2.06405  0.63923 -0.10838
C   2.77876 -0.58210 -0.19623
C   4.19004 -0.58909 -0.24161
C   4.88990  0.62403 -0.19496
C   4.18350  1.84063 -0.10278
C   2.77584  1.85432 -0.05974
C   1.90610 -1.74623 -0.20883
N   0.56342 -1.42131 -0.11496
C  -0.34656 -2.42975 -0.09699
C   0.01612 -3.77759 -0.16720
C   1.37383 -4.11878 -0.26723
C   2.31753 -3.08899 -0.28869
Ru  0.06580  0.59863 -0.09366
N   0.06218  0.53851 -2.07691
C   0.10519  0.50758 -3.25189
C   0.15407  0.47935 -4.70802
Br -1.78454  2.25355 -0.16292
H   4.74101 -1.53389 -0.30924
H   3.38514 -3.31170 -0.36378
H   1.68888 -5.16450 -0.32634
H  -0.76821 -4.53816 -0.14344
H  -1.39095 -2.13668 -0.01748
H   5.98381  0.62612 -0.22811
H   4.73206  2.78853 -0.06265
H   2.24127  2.80962  0.00906
H   0.25982  1.50196 -5.10650
H   1.01435 -0.12094 -5.04678
H  -0.76859  0.03459 -5.11616
O   0.05181  0.96597  1.94517
C   0.55052  0.22156  2.91394
O   0.97884 -0.94136  2.80369
C   0.55586  0.94873  4.26447
H  -0.42387  1.41205  4.46493
H   0.80925  0.25021  5.07510
H   1.30314  1.76099  4.24728
C  -2.37009  0.03145  0.02338
C  -2.80002 -0.33189  1.30962
C  -2.98951 -0.45991 -1.13731
C  -3.80530 -1.30951  1.42688
C  -3.99540 -1.43421 -0.99878
C  -4.40079 -1.86288  0.27843
H  -2.33202  0.10866  2.19305
H  -2.67838 -0.11900 -2.12820
H  -4.12344 -1.63153  2.42431
H  -4.46450 -1.84991 -1.89696
H  -5.19095 -2.61327  0.37877
```

46
25b'
SCF(BP86)= -1180.15691414
G(298 K) = -1179.871561
SCF(PBE) = -3739.59849907
Low Freq. = 14.6755cm-1,
20.9968cm-1

```
C   1.78268 -0.53381 -0.01297
C   2.57166  0.63543 -0.10230
C   3.97746  0.53340 -0.16349
C   4.58373 -0.72973 -0.14941
C   3.79256 -1.89121 -0.08083
C   2.38712 -1.80490 -0.04088
C   1.80357  1.86881 -0.07432
N   0.45540  1.65460  0.07814
C  -0.35517  2.72937  0.25152
C   0.11541  4.04469  0.21141
C   1.48330  4.27344 -0.00699
C   2.33172  3.17212 -0.13954
Ru -0.20491 -0.40520  0.31641
N  -0.33799  0.21183  2.22067
C  -0.39876  0.54014  3.34483
C  -0.47221  0.94657  4.73835
Br -0.96221 -2.69509  1.06485
H   4.59529  1.43557 -0.21891
H   3.40655  3.30837 -0.28202
H   1.88191  5.29065 -0.05676
H  -0.59045  4.86763  0.34733
H  -1.41116  2.51593  0.41869
H   5.67381 -0.81084 -0.19874
H   4.26582 -2.87893 -0.07776
H   1.78019 -2.71186 -0.01891
H  -0.30779  0.07672  5.39459
H   0.29483  1.70923  4.94900
H  -1.46658  1.37227  4.95093
O  -0.13255 -1.36196 -1.56359
C   0.41865 -0.88932 -2.66299
O   0.92684  0.23873 -2.81265
C   0.37765 -1.90571 -3.80905
H  -0.65085 -2.26883 -3.96851
H   0.75608 -1.44978 -4.73510
H   0.99865 -2.78163 -3.55576
C  -1.85242  0.40727 -0.59094
C  -1.98719  0.99564 -1.85581
C  -2.97429  0.24600  0.25092
C  -3.26042  1.40421 -2.29749
C  -4.24471  0.64987 -0.21277
C  -4.39119  1.22925 -1.48253
H  -1.10418  1.13589 -2.48576
H  -2.88909 -0.18826  1.25177
H  -3.35957  1.86271 -3.28768
H  -5.11262  0.51350  0.44167
H  -5.37856  1.55009 -1.82966
```

40
27a
SCF(BP86)= -1047.40589872
G(298 K) = -1047.160809
SCF(PBE) = -3606.95134807
Low Freq. = 33.7510cm-1,
39.6606cm-1

```
C  -3.76178  0.25435 -1.25516
C  -2.43127  0.39450 -0.80377
C  -1.69692 -0.74809 -0.39252
C  -2.30866 -2.01698 -0.40267
C  -3.64430 -2.13883 -0.82827
C  -4.36159 -1.01095 -1.27045
C  -1.72043  1.65310 -0.62761
C  -2.21108  2.94050 -0.91868
```

```
C  -1.43882  4.06273 -0.60980
C  -0.18504  3.87598 -0.00292
C   0.26002  2.57603  0.24711
N  -0.47627  1.47956 -0.07024
Ru  0.16208 -0.49826  0.32548
O   0.84199 -2.46626  0.69792
C   0.90151 -2.77585 -0.55994
C   1.41054 -4.11020 -1.01771
O   0.51718 -1.86841 -1.38483
H  -4.33188  1.13226 -1.57612
H  -3.19801  3.04996 -1.37486
H  -1.81052  5.06781 -0.82815
H   0.44858  4.72131  0.27593
H   1.22882  2.38491  0.71116
H  -5.39351 -1.11973 -1.61754
H  -4.11986 -3.12525 -0.82583
H  -1.76599 -2.90810 -0.07533
H   1.15152 -4.28201 -2.07166
H   2.50870 -4.13515 -0.91068
H   0.99402 -4.91095 -0.38715
Br -0.05990  0.15422  2.69426
C   1.83346  0.21585 -0.57882
C   2.96891  0.05564  0.24243
C   1.94759  0.79325 -1.85339
C   4.22356  0.50217 -0.22369
C   3.20734  1.22444 -2.30860
C   4.34553  1.08433 -1.49530
H   2.89852 -0.40430  1.23476
H   1.06541  0.90575 -2.49134
H   5.10250  0.38179  0.41900
H   3.29300  1.67226 -3.30476
H   5.32299  1.42384 -1.85223

40
27a (triplet)
SCF(BP86)= -1047.36980391
G(298 K) = -1047.127438
SCF(PBE) = -3606.91254912
Low Freq. = 31.4554cm-1,
35.7913cm-1
C  -3.96398  0.37675 -0.89114
C  -2.58886  0.45613 -0.57193
C  -1.88703 -0.68851 -0.10881
C  -2.58229 -1.90704  0.03294
C  -3.95398 -1.97682 -0.27063
C  -4.64064 -0.83957 -0.74089
C  -1.79839  1.68455 -0.60870
C  -2.28506  2.95946 -0.95845
C  -1.45768  4.07995 -0.86293
C  -0.14242  3.91066 -0.39997
C   0.30641  2.62589 -0.09139
N  -0.48330  1.52253 -0.21437
Ru  0.16452 -0.42593  0.20977
O   0.56393 -2.49243  0.24234
C   0.53672 -2.58374 -1.05454
C   0.81133 -3.88059 -1.75140
O   0.27726 -1.49765 -1.69276
H  -4.50788  1.25686 -1.24986
H  -3.32132  3.06243 -1.28866
H  -1.83555  5.07134 -1.12817
H   0.53718  4.75697 -0.27531
H   1.32053  2.44811  0.26611
H  -5.70521 -0.90400 -0.98613
H  -4.48918 -2.92470 -0.14516
H  -2.06109 -2.80450  0.38308
H   0.57141 -3.80244 -2.82058
H   1.87854 -4.13638 -1.63590
H   0.22318 -4.68834 -1.28816
Br  0.11342  0.03602  2.61526
C   2.11238  0.17093 -0.28317
C   3.17331 -0.14333  0.60809
C   2.43674  0.72294 -1.54804
C   4.51148  0.05275  0.22403
C   3.77371  0.91498 -1.93077
C   4.81513  0.58084 -1.04423
H   2.95588 -0.54558  1.60367
H   1.63683  0.98807 -2.24916
H   5.31784 -0.20163  0.92123
H   4.00454  1.32858 -2.91874
H   5.85810  0.74100 -1.33678

40
27a'
SCF(BP86)= -1047.38527419
G(298 K) = -1047.141560
SCF(PBE) = -3606.93083563
Low Freq. = 22.9005cm-1,
39.3490cm-1
C   1.64311  3.43122 -0.38657
C   1.36637  2.06404 -0.19290
N   0.12418  1.54608 -0.50536
C  -0.79470  2.36038 -1.09718
C  -0.55333  3.71305 -1.33939
C   0.67892  4.26790 -0.95237
C   2.31522  1.04733  0.23826
C   3.68030  1.27474  0.52969
C   4.52073  0.19316  0.81492
C   4.01105 -1.12112  0.80504
C   2.64993 -1.35811  0.54483
C   1.79715 -0.27513  0.25296
Ru -0.12713 -0.49143 -0.30396
O   0.20921 -0.83358 -2.33646
C   0.19989 -2.13432 -2.18156
C   0.43417 -3.03379 -3.35460
O   0.00647 -2.57970 -0.99265
H   2.62592  3.82152 -0.11116
H   4.08713  2.29126  0.51754
H   5.57707  0.36939  1.03989
H   4.67333 -1.96675  1.01958
H   2.25658 -2.37822  0.57073
H   0.88976  5.32925 -1.11011
H  -1.32942  4.31541 -1.81758
H  -1.74163  1.89482 -1.37218
H   0.11581 -4.05929 -3.12172
H  -0.09993 -2.65251 -4.23838
H   1.51247 -3.03940 -3.59143
Br -0.22422 -1.12489  2.21737
C  -1.96225  0.02479  0.40524
C  -2.47921  1.13323  1.09580
C  -2.81615 -0.89196 -0.24111
C  -3.86261  1.37751  1.05316
C  -4.19812 -0.60729 -0.31202
```

C -4.72070 0.52131 0.33659
H -1.81491 1.80203 1.65061
H -2.43847 -1.82737 -0.67370
H -4.26902 2.24538 1.58366
H -4.85950 -1.30775 -0.83287
H -5.79688 0.71842 0.31101

40
27b
SCF(BP86)= -1047.40046719
G(298 K) = -1047.156467
SCF(PBE) = -3606.94314966
Low Freq. = 26.8139cm-1,
38.6257cm-1
C -3.65152 1.28224 0.52760
C -2.49338 0.51022 0.30725
N -1.24790 1.06059 0.49575
C -1.13119 2.35270 0.88840
C -2.24792 3.16080 1.11637
C -3.53062 2.61378 0.93419
C -2.43575 -0.87887 -0.13425
C -3.56107 -1.66228 -0.45550
C -3.38876 -2.98337 -0.89308
C -2.09462 -3.52811 -1.01096
C -0.96557 -2.76094 -0.68481
C -1.10597 -1.41767 -0.24709
Ru 0.38429 -0.20926 0.26392
O   1.98132 1.20955 1.52222
C   1.39137 0.72723 2.52570
C   1.81230 0.95920 3.95408
O   0.35176 -0.04615 2.31281
H -4.63704 0.83399 0.37952
H -4.57110 -1.24833 -0.37066
H -4.26425 -3.59039 -1.14328
H -1.96698 -4.55929 -1.35705
H   0.03285 -3.19709 -0.77455
H -4.42397 3.22040 1.10678
H -2.10729 4.19769 1.43061
H -0.10986 2.72006 1.01600
H   0.94041 0.96822 4.62478
H   2.47776 0.13735 4.27005
H   2.36548 1.90582 4.03283
Br 2.28626 -1.86654 0.15612
C   0.82110 0.95876 -1.30474
C -0.14745 1.22783 -2.30655
C   2.16605 1.36546 -1.51422
C   0.24170 1.80447 -3.52740
C   2.53472 1.97393 -2.72257
C   1.57768 2.18814 -3.73344
H -1.19609 0.95948 -2.15331
H   2.91516 1.19007 -0.73936
H -0.50777 1.97091 -4.30835
H   3.57626 2.27395 -2.87945
H   1.87284 2.66100 -4.67567

40
27b (triplet)
SCF(BP86)= -1047.39704826
G(298 K) = -1047.154765
SCF(PBE) = -3606.93945321
Low Freq. = 25.8301cm-1,
34.3932cm-1
C -3.65532 1.11482 0.51279
C -2.45774 0.41486 0.26745
N -1.25373 0.96237 0.64453
C -1.21119 2.18704 1.22745
C -2.37035 2.92538 1.47343
C -3.61365 2.37448 1.11643
C -2.32288 -0.88065 -0.39357
C -3.41141 -1.63253 -0.88263
C -3.18483 -2.85393 -1.52950
C -1.86878 -3.33155 -1.68612
C -0.77794 -2.60244 -1.18810
C -0.97484 -1.35318 -0.54522
Ru 0.45275 -0.19569 0.27019
O   1.41890 0.89030 1.95328
C   0.95438 0.06125 2.83037
C   1.32516 0.20589 4.28461
O   0.19575 -0.89008 2.42437
H -4.61080 0.66995 0.22549
H -4.43666 -1.26882 -0.76010
H -4.03072 -3.43517 -1.90864
H -1.69101 -4.28472 -2.19575
H   0.23598 -2.98966 -1.31932
H -4.54018 2.92344 1.30666
H -2.29241 3.90874 1.94268
H -0.21790 2.54743 1.50296
H   0.60547 -0.32484 4.92364
H   2.32604 -0.23101 4.44508
H   1.37405 1.26889 4.56467
Br 2.38439 -1.71205 -0.07337
C   0.87264 1.05323 -1.24787
C   0.12341 1.14378 -2.45268
C   1.98137 1.92668 -1.07323
C   0.48858 2.05724 -3.45232
C   2.33641 2.84483 -2.07340
C   1.59365 2.91074 -3.26686
H -0.74076 0.49060 -2.60702
H   2.56927 1.88251 -0.14959
H -0.09353 2.10807 -4.37894
H   3.19552 3.50742 -1.92304
H   1.87203 3.62532 -4.04817

40
27c
SCF(BP86)= -1047.39570308
G(298 K) = -1047.151606
SCF(PBE) = -3606.93952178
Low Freq. = 29.6361cm-1,
36.5005cm-1
C -3.45958 -0.52878 1.69598
C -2.35065 -0.25223 0.87269
C -1.06916 -0.81507 1.17538
C -0.94084 -1.68346 2.28578
C -2.05476 -1.95257 3.09772
C -3.30783 -1.37645 2.80435
C -2.36028 0.59683 -0.31476
C -3.47731 1.25127 -0.87161
C -3.32372 2.02379 -2.02582
C -2.05012 2.13000 -2.61233
C -0.97715 1.45863 -2.01922

```
N  -1.12213  0.71215 -0.89913
Ru  0.45775 -0.36024  0.01870
O   1.88077 -1.50664  0.95832
C   2.93844 -1.34408  0.18663
C   4.20545 -2.06052  0.59159
O   2.84695 -0.61966 -0.83721
H  -4.43966 -0.09088  1.47936
H  -4.45647  1.14678 -0.39753
H  -4.18410  2.53595 -2.46570
H  -1.88312  2.72071 -3.51615
H   0.03188  1.50527 -2.43614
H  -4.17068 -1.59330  3.44149
H  -1.94924 -2.61516  3.96330
H   0.03084 -2.13379  2.51391
H   5.03275 -1.79264 -0.07945
H   4.46876 -1.80326  1.63059
H   4.04180 -3.15076  0.55570
Br  0.05251 -1.89168 -1.90041
C   1.05388  1.52124  0.38554
C   2.16475  2.13672 -0.23838
C   0.40921  2.17532  1.46962
C   2.68147  3.33467  0.27879
C   0.96601  3.35255  2.00388
C   2.09476  3.93732  1.40864
H   2.63450  1.66830 -1.10668
H  -0.49696  1.75954  1.91746
H   3.54730  3.79959 -0.20479
H   0.48839  3.82483  2.86906
H   2.50259  4.87249  1.80541
```

```
40
27c (triplet)
SCF(BP86)= -1047.39459355
G(298 K) = -1047.152417
SCF(PBE) = -3606.93638924
Low Freq. = 26.5590cm-1,
32.4022cm-1
C  -3.21886  0.57959  1.93113
C  -2.20019  0.34908  0.98752
C  -0.91449 -0.12511  1.41193
C  -0.69340 -0.37127  2.78789
C  -1.71524 -0.13712  3.72094
C  -2.97204  0.33936  3.29319
C  -2.34171  0.52729 -0.46056
C  -3.49917  0.94693 -1.14220
C  -3.48711  1.03729 -2.53833
C  -2.31728  0.70128 -3.24051
C  -1.19398  0.29562 -2.51422
N  -1.20090  0.21580 -1.16414
Ru  0.44209 -0.39665  0.00367
O   2.07683 -0.96933  1.14384
C   2.97167 -1.07393  0.18482
C   4.37096 -1.48600  0.56970
O   2.62624 -0.82556 -1.00616
H  -4.20391  0.94098  1.61808
H  -4.40174  1.19425 -0.57840
H  -4.38306  1.36147 -3.07526
H  -2.26879  0.74812 -4.33067
H  -0.25913  0.01498 -3.00641
H  -3.76486  0.51983  4.02546
H  -1.53789 -0.32474  4.78506
H   0.28340 -0.73959  3.12006
H   5.01582 -1.54318 -0.31756
H   4.78880 -0.75954  1.28635
H   4.34578 -2.46611  1.07410
Br -0.13328 -2.81062 -0.67495
C   1.06445  1.54329 -0.03747
C   1.53702  2.09228 -1.26017
C   1.11328  2.35918  1.12669
C   2.04432  3.39847 -1.31738
C   1.62400  3.66436  1.06644
C   2.08907  4.18971 -0.15400
H   1.52199  1.48704 -2.17217
H   0.74923  1.96962  2.08230
H   2.40649  3.80014 -2.26982
H   1.65384  4.27672  1.97426
H   2.48107  5.21098 -0.19884
```

```
40
27d
SCF(BP86)= -1047.39354779
G(298 K) = -1047.148667
SCF(PBE) = -3606.93659929
Low Freq. = 19.2935cm-1,
35.0018cm-1
C   3.38752  1.62144 -0.83827
C   2.22406  0.82638 -0.69175
C   1.08374  1.32752  0.00329
C   1.14955  2.62310  0.55734
C   2.30838  3.40165  0.41721
C   3.42717  2.90368 -0.28567
C   2.08461 -0.53151 -1.19588
C   3.08314 -1.29144 -1.83824
C   2.82289 -2.60824 -2.21961
C   1.55542 -3.15831 -1.95186
C   0.59077 -2.36438 -1.33415
N   0.83397 -1.07465 -0.96565
Ru -0.54958  0.11565 -0.01240
O  -1.99963  1.49132  0.85672
C  -2.03173  2.17929 -0.22443
C  -2.94648  3.34958 -0.42780
O  -1.22632  1.78469 -1.16098
H   4.25587  1.23882 -1.38491
H   4.06255 -0.84354 -2.02232
H   3.59619 -3.20341 -2.71354
H   1.30872 -4.18802 -2.22126
H  -0.41764 -2.72954 -1.12841
H   4.32526  3.51913 -0.39587
H   2.34565  4.40329  0.85949
H   0.29549  3.02521  1.11254
H  -2.51547  4.05582 -1.15217
H  -3.91078  2.98945 -0.82614
H  -3.13562  3.85318  0.53161
Br -2.54981 -1.06590 -1.24631
C   0.05491 -0.30263  1.82815
C   1.32568 -0.35730  2.45511
C  -1.03511 -1.02965  2.38528
C   1.52556 -1.21960  3.53667
C  -0.81017 -1.92906  3.44964
C   0.46315 -2.01613  4.02555
H   2.15222  0.25210  2.07867
H  -2.04790 -0.90884  1.98459
```

H 2.51296 -1.28235 4.00571
H -1.64417 -2.51399 3.85012
H 0.63284 -2.68108 4.87840

40
27d (triplet)
SCF(BP86)= -1047.39426246
G(298 K) = -1047.151881
SCF(PBE) = -3606.93661567
Low Freq. = 20.4841cm-1,
36.1042cm-1
C 3.61551 1.41993 0.36634
C 2.42956 0.72918 0.03242
C 1.15679 1.25230 0.41179
C 1.10917 2.46121 1.14131
C 2.29467 3.13226 1.47995
C 3.54661 2.61808 1.08654
C 2.38996 -0.55347 -0.66428
C 3.50740 -1.27673 -1.12503
C 3.33467 -2.52279 -1.73074
C 2.03372 -3.03659 -1.86444
C 0.95478 -2.27870 -1.40819
N 1.11417 -1.05674 -0.82873
Ru -0.46362 0.20184 -0.22094
O -1.68962 1.80577 0.21884
C -1.54360 2.48132 -0.90475
C -2.34851 3.74113 -1.07975
O -0.74151 2.03709 -1.77491
H 4.59210 1.02332 0.07085
H 4.50750 -0.85571 -0.99981
H 4.19958 -3.08849 -2.08805
H 1.84551 -4.01263 -2.31778
H -0.07422 -2.63062 -1.49917
H 4.46615 3.15119 1.34611
H 2.24293 4.06433 2.05297
H 0.14284 2.87344 1.44680
H -2.09397 4.23279 -2.02818
H -3.42398 3.49683 -1.06683
H -2.15770 4.42644 -0.23786
Br -2.20384 -1.03630 -1.63800
C -0.68854 -0.73030 1.53610
C 0.29645 -1.53491 2.17267
C -1.94997 -0.57904 2.17904
C 0.02251 -2.17535 3.38909
C -2.22041 -1.22354 3.39511
C -1.23614 -2.02506 4.00352
H 1.28273 -1.65935 1.71643
H -2.72145 0.04726 1.71867
H 0.79375 -2.79174 3.86351
H -3.19952 -1.09980 3.87009
H -1.44630 -2.52721 4.95338

40
27e
SCF(BP86)= -1047.38684824
G(298 K) = -1047.142348
SCF(PBE) = -3606.92851021
Low Freq. = 32.7796cm-1,
36.5145cm-1
C -4.15837 -0.01189 -0.20057
C -2.76787 0.21754 -0.16572
N -1.88266 -0.82159 -0.09143
C -2.34926 -2.08713 -0.02392
C -3.71667 -2.38478 -0.04468
C -4.63544 -1.32497 -0.14035
C -2.10190 1.51981 -0.18200
C -2.76749 2.76127 -0.19199
C -2.03292 3.95654 -0.18808
C -0.62682 3.92226 -0.18269
C 0.05568 2.69314 -0.19992
C -0.67140 1.48034 -0.16684
Ru 0.29047 -0.25235 -0.01208
O 0.34363 -1.90388 -1.25856
C 0.55170 -1.22339 -2.35112
C 0.77081 -1.91175 -3.66144
O 0.56805 0.04206 -2.19264
H -4.85367 0.82829 -0.27270
H -3.86148 2.79975 -0.19654
H -2.55835 4.91632 -0.19474
H -0.05362 4.85518 -0.17758
H 1.14758 2.68325 -0.20515
H -5.71122 -1.52161 -0.16487
H -4.04816 -3.42472 0.00964
H -1.59161 -2.87275 0.04017
H 0.64852 -1.19835 -4.48823
H 1.79537 -2.32086 -3.68967
H 0.06840 -2.75163 -3.77432
Br 0.06857 -0.67212 2.41870
C 2.31145 0.05972 0.07023
C 3.12304 -0.72306 0.94316
C 2.99709 0.95513 -0.80060
C 4.52341 -0.60512 0.95672
C 4.39387 1.09149 -0.77351
C 5.16641 0.30980 0.10539
H 2.65131 -1.43649 1.62703
H 2.42712 1.55688 -1.51618
H 5.11277 -1.22584 1.64143
H 4.88268 1.80500 -1.44687
H 6.25677 0.41115 0.12293

40
27e (triplet)
SCF(BP86)= -1047.38799327
G(298 K) = -1047.145731
SCF(PBE) = -3606.92951650
Low Freq. = 20.5992cm-1,
33.3841cm-1
C -4.03501 0.66782 -0.35446
C -2.62638 0.62975 -0.30150
N -1.97877 -0.52712 0.03424
C -2.68873 -1.64080 0.32453
C -4.08728 -1.66247 0.29635
C -4.76835 -0.48448 -0.05338
C -1.72419 1.75785 -0.57077
C -2.19020 3.03282 -0.95340
C -1.28896 4.07861 -1.19299
C 0.09362 3.85628 -1.05549
C 0.57517 2.59112 -0.68652
C -0.31305 1.51750 -0.41979
Ru 0.22833 -0.35561 0.08008
O 0.27840 -2.57601 -0.27838
C 0.16641 -2.35146 -1.53689

```
C   0.11501 -3.45814 -2.55118
O   0.12254 -1.10905 -1.91062
H  -4.55146  1.59143 -0.62578
H  -3.26303  3.21566 -1.06896
H  -1.66361  5.06283 -1.49012
H   0.80186  4.67060 -1.24185
H   1.65224  2.43671 -0.58642
H  -5.86139 -0.46396 -0.09019
H  -4.62270 -2.58283  0.54161
H  -2.10152 -2.52723  0.57953
H  -0.32788 -3.10456 -3.49297
H   1.14202 -3.80785 -2.75543
H  -0.45822 -4.30915 -2.15416
Br  0.10772 -0.29413  2.53593
C   2.23458 -0.02173 -0.08451
C   2.97275  0.70930  0.89809
C   2.97957 -0.52485 -1.19314
C   4.35853  0.88978  0.79774
C   4.36647 -0.34192 -1.29815
C   5.06496  0.36337 -0.30144
H   2.44812  1.12164  1.76603
H   2.45923 -1.04749 -2.00134
H   4.89284  1.44152  1.57920
H   4.90391 -0.74371 -2.16434
H   6.14738  0.50785 -0.38241

40
27f
SCF(BP86)= -1047.38577262
G(298 K) = -1047.140547
SCF(PBE) = -3606.93159565
Low Freq. = 32.9657cm-1,
44.9763cm-1
C  -0.96074  3.65650 -0.15078
C  -0.92001  2.24711 -0.18956
N   0.12510  1.56764  0.39661
C   1.08038  2.26226  1.07453
C   1.06019  3.65233  1.16959
C   0.03195  4.36587  0.52492
C  -1.93550  1.36475 -0.73452
C  -3.13058  1.79070 -1.36184
C  -4.08983  0.84915 -1.74271
C  -3.86890 -0.51992 -1.48517
C  -2.67841 -0.95727 -0.88175
C  -1.68891 -0.01962 -0.51284
Ru  0.01504 -0.49751  0.42818
O  -0.95612 -2.18156  1.48016
C  -1.08182 -1.42938  2.51374
C  -1.70094 -1.90560  3.79079
O  -0.67064 -0.19685  2.39574
H  -1.78841  4.17970 -0.63573
H  -3.31108  2.85634 -1.53757
H  -5.01417  1.17530 -2.22911
H  -4.62812 -1.25785 -1.76668
H  -2.51768 -2.02291 -0.70350
H   0.00225  5.45838  0.56301
H   1.84878  4.16111  1.72886
H   1.87328  1.66685  1.53080
H  -1.26729 -1.37424  4.65076
H  -2.78365 -1.69076  3.76475
H  -1.56654 -2.99170  3.89700
Br  0.32745 -2.09603 -1.57687
C   1.93422 -0.16540 -0.27772
C   2.40483  0.66499 -1.30726
C   2.84202 -0.83211  0.56832
C   3.78951  0.84806 -1.47534
C   4.22749 -0.62202  0.40767
C   4.70347  0.21333 -0.61553
H   1.70108  1.16936 -1.97696
H   2.50039 -1.52523  1.34878
H   4.15070  1.49483 -2.28256
H   4.92600 -1.13717  1.07592
H   5.77994  0.36084 -0.74796

40
26a
SCF(BP86)= -1047.37026712
G(298 K) = -1047.126292
SCF(PBE) = -3606.91740452
Low Freq. = 11.1317cm-1,
17.2782cm-1
C  -0.36448  3.29819 -1.16065
C   0.23802  2.13059 -0.65410
N  -0.11925  0.88675 -1.15773
C  -1.05123  0.81727 -2.14775
C  -1.66734  1.94904 -2.68555
C  -1.31972  3.21576 -2.17938
C   1.26524  2.04362  0.37900
C   1.80786  3.14973  1.07200
C   2.80151  2.95626  2.03961
C   3.25707  1.64890  2.31688
C   2.72559  0.54099  1.63594
C   1.71599  0.70353  0.65145
Ru  0.86977 -0.71266 -0.39979
O   2.52102 -0.90571 -1.70060
C   2.96592 -1.94728 -1.06432
C   4.17548 -2.69265 -1.55959
O   2.30662 -2.32044 -0.02602
H  -0.07321  4.27052 -0.75335
H   1.45280  4.16475  0.85683
H   3.22014  3.81309  2.57709
H   4.03533  1.49517  3.07425
H   3.09442 -0.46507  1.86675
H  -1.78615  4.12262 -2.57523
H  -2.40761  1.83283 -3.48124
H  -1.29083 -0.19119 -2.49854
H   4.66282 -3.22443 -0.72948
H   3.86571 -3.43784 -2.31345
H   4.88563 -2.00147 -2.03747
Br -0.81378 -1.20090  1.35230
C  -2.69560 -0.84442  0.82061
C  -3.33377  0.28810  1.34034
C  -3.32961 -1.74070 -0.04865
C  -4.67077  0.52484  0.96974
C  -4.66605 -1.48520 -0.40686
C  -5.33386 -0.35751  0.10028
H  -2.80895  0.97078  2.01448
H  -2.80196 -2.61654 -0.43707
H  -5.18890  1.40419  1.36540
H  -5.18126 -2.17475 -1.08317
H  -6.37354 -0.16627 -0.18239
```

40
TS(26a-27a)
SCF(BP86)= -1047.33563234
G(298 K) = -1047.092245
SCF(PBE) = -3606.88097472
Low Freq. = -183.4042cm-1, 16.3532cm-1

```
C  -4.27736 -0.67856 -0.14539
C  -2.94014 -0.22328 -0.16321
C  -1.86783 -1.10118  0.19812
C  -2.18218 -2.43884  0.53344
C  -3.51611 -2.88187  0.54492
C  -4.56488 -2.00424  0.20542
C  -2.54123  1.14531 -0.49486
C  -3.40297  2.17131 -0.92319
C  -2.90335  3.44728 -1.20499
C  -1.52888  3.68037 -1.03809
C  -0.71354  2.63074 -0.60875
N  -1.18027  1.37803 -0.35026
Ru -0.06965 -0.24744  0.24043
O   0.87583 -2.22958  0.36559
C   0.83480 -2.29646 -0.91322
C   1.41750 -3.44349 -1.68879
O   0.28656 -1.29075 -1.53312
H  -5.09900  0.00089 -0.39809
H  -4.46918  1.95966 -1.03744
H  -3.57227  4.24353 -1.54344
H  -1.08336  4.65988 -1.22902
H   0.35288  2.78470 -0.44140
H  -5.60212 -2.35362  0.21648
H  -3.74038 -3.91909  0.81923
H  -1.37855 -3.13453  0.79571
H   0.92430 -3.54314 -2.66657
H   2.49243 -3.25749 -1.85715
H   1.31829 -4.37656 -1.11452
Br  1.15198  1.06624  1.91498
C   2.43128  0.40094  0.16507
C   3.32813 -0.61891  0.52600
C   2.73520  1.33540 -0.83614
C   4.51208 -0.76580 -0.21699
C   3.92167  1.16443 -1.57676
C   4.80756  0.11885 -1.27064
H   3.10306 -1.29796  1.35108
H   2.06995  2.16694 -1.07578
H   5.20879 -1.56998  0.04229
H   4.14808  1.86512 -2.38735
H   5.73687  0.00508 -1.83722
```

40
TS(26a-27e’)
SCF(BP86)= -1047.36003738
G(298 K) = -1047.115953
SCF(PBE) = -3606.90769877
Low Freq. = -101.1373cm-1, 25.6533cm-1

```
C   2.27890  3.01097 -0.85040
C   1.81184  1.72824 -0.50590
N   0.48564  1.37719 -0.73523
C  -0.32980  2.28088 -1.34884
C   0.09641  3.55669 -1.72366
C   1.42420  3.93763 -1.45687
C   2.60019  0.63363  0.05427
C   3.96616  0.72083  0.40543
C   4.63811 -0.40330  0.90290
C   3.94294 -1.62243  1.05051
C   2.58304 -1.72061  0.70953
C   1.87431 -0.59775  0.21055
Ru -0.03610 -0.51877 -0.23702
O   0.15696 -1.46155 -2.08722
C  -0.10578 -2.63930 -1.59799
C  -0.18982 -3.83949 -2.50078
O  -0.32456 -2.70694 -0.33545
H   3.32253  3.26993 -0.65076
H   4.50785  1.66738  0.29180
H   5.69596 -0.33412  1.17596
H   4.46843 -2.50316  1.43891
H   2.05919 -2.67488  0.83557
H   1.78749  4.93308 -1.72818
H  -0.60809  4.23544 -2.21148
H  -1.35475  1.94444 -1.52441
H   0.08859 -4.75009 -1.95006
H  -1.22774 -3.95912 -2.85869
H   0.46223 -3.71142 -3.37732
Br -0.73356  0.06427  2.21341
C  -2.25151  0.25936  0.98921
C  -2.78552  1.54432  0.76857
C  -2.87337 -0.91152  0.50519
C  -3.93326  1.65529 -0.03087
C  -4.01797 -0.76265 -0.30496
C  -4.54528  0.50935 -0.57549
H  -2.30818  2.42927  1.19559
H  -2.49079 -1.90174  0.76185
H  -4.35269  2.64806 -0.22199
H  -4.50563 -1.65983 -0.69908
H  -5.44293  0.61016 -1.19248
```

40
26a'
SCF(BP86)= -1047.36931968
G(298 K) = -1047.125492
SCF(PBE) = -3606.91536921
Low Freq. = 9.9534cm-1, 16.0389cm-1

```
C   1.98983 -1.57868  2.68875
C   1.76290 -0.90716  1.46588
C   0.66741  0.01689  1.31844
C  -0.16025  0.23473  2.45182
C   0.07789 -0.43824  3.66150
C   1.15074 -1.34809  3.78591
C   2.58352 -1.05838  0.26865
C   3.73697 -1.85364  0.12250
C   4.43406 -1.87332 -1.08982
C   3.96358 -1.08452 -2.15702
C   2.81369 -0.31533 -1.96983
N   2.12621 -0.29038 -0.79423
Ru  0.48667  0.85914 -0.43362
O  -0.81881  2.52421  0.13365
C   0.22633  3.25196  0.30726
C   0.13812  4.71090  0.66470
O   1.38048  2.68207  0.12681
H   2.82458 -2.28344  2.78492
H   4.08307 -2.45331  0.96916
```

```
H   5.32994 -2.49062 -1.20361
H   4.47298 -1.06478 -3.12389
H   2.40196  0.31030 -2.76811
H   1.32692 -1.86902  4.73242
H  -0.57869 -0.25351  4.52044
H  -0.99781  0.93776  2.37876
H   0.09492  5.31377 -0.25957
H  -0.77628  4.90693  1.24372
H   1.02466  5.02363  1.23575
Br -0.93856 -0.90290 -1.46597
C  -2.75911 -0.92172 -0.69725
C  -3.08978 -1.93255  0.21343
C  -3.66187  0.07400 -1.08971
C  -4.38986 -1.93797  0.75209
C  -4.95592  0.04919 -0.53849
C  -5.31847 -0.95198  0.37851
H  -2.36047 -2.69538  0.49901
H  -3.36983  0.84911 -1.80355
H  -4.66995 -2.71899  1.46600
H  -5.67831  0.81691 -0.83316
H  -6.32719 -0.96412  0.80232

40
TS(26a'-27d)
SCF(BP86)= -1047.34883362
G(298 K) = -1047.105225
SCF(PBE) = -3606.89876786
Low Freq. = -170.5521cm-1,
10.7554cm-1
C  -1.64038  3.11200 -0.91166
C  -1.46112  1.77768 -0.47893
C  -0.22274  1.35352  0.11090
C   0.80620  2.31588  0.25254
C   0.61423  3.64018 -0.17439
C  -0.60633  4.04283 -0.75878
C  -2.46525  0.72585 -0.57351
C  -3.76648  0.83123 -1.10145
C  -4.61179 -0.28274 -1.12308
C  -4.13898 -1.50515 -0.60930
C  -2.84283 -1.56500 -0.09483
N  -2.01226 -0.48389 -0.06480
Ru -0.16754 -0.49962  0.76213
O   1.45664 -0.42430  2.25191
C   0.61449  0.05593  3.09229
C   0.97008  0.34968  4.52283
O  -0.59289  0.26298  2.65108
H  -2.58994  3.42398 -1.36229
H  -4.10796  1.79344 -1.49326
H  -5.62333 -0.20253 -1.53147
H  -4.76066 -2.40405 -0.60450
H  -2.43010 -2.48841  0.32202
H  -0.74526  5.07721 -1.08910
H   1.42318  4.37015 -0.05102
H   1.76270  2.02013  0.69661
H   1.15539 -0.59558  5.06117
H   1.89835  0.94174  4.56314
H   0.15754  0.89546  5.02262
Br  0.51858 -2.26303 -1.03322
C   1.84984 -0.71088 -1.06188
C   1.73904  0.17529 -2.14871
C   3.05210 -0.87221 -0.35014
C   2.84782  0.97377 -2.47532
C   4.14177 -0.05453 -0.69153
C   4.04838  0.86585 -1.75225
H   0.80841  0.25024 -2.71651
H   3.12891 -1.59596  0.46390
H   2.76626  1.67714 -3.31073
H   5.07531 -0.15501 -0.12762
H   4.91029  1.48402 -2.02081

40
26b
SCF(BP86)= -1047.36036739
G(298 K) = -1047.117041
SCF(PBE) = -3606.90934583
Low Freq. = 17.0010cm-1,
27.5966cm-1
C   2.30026 -0.11576  2.97985
C   1.94280 -0.16806  1.61982
N   0.86118  0.58068  1.16258
C   0.16819  1.36353  2.03939
C   0.49614  1.43605  3.39412
C   1.58227  0.68299  3.87671
C   2.58628 -0.94244  0.56241
C   3.72211 -1.76374  0.73987
C   4.25880 -2.45876 -0.35179
C   3.65759 -2.33193 -1.62255
C   2.52653 -1.51652 -1.80372
C   1.95907 -0.79480 -0.72276
Ru  0.43222  0.48638 -0.75451
O  -0.67307  2.56728 -0.90553
C   0.48215  3.04021 -1.16692
C   0.69493  4.49271 -1.52070
O   1.50674  2.23378 -1.16414
H   3.15072 -0.70979  3.32616
H   4.19092 -1.86072  1.72653
H   5.13882 -3.09613 -0.21860
H   4.07590 -2.87750 -2.47700
H   2.07645 -1.43991 -2.80282
H   1.86431  0.72115  4.93270
H  -0.09400  2.07919  4.05203
H  -0.65320  1.93587  1.60170
H   1.74020  4.79101 -1.35528
H   0.45313  4.64670 -2.58702
H   0.02020  5.12919 -0.92853
Br -1.06921 -1.49369 -0.62390
C  -2.91408 -0.99604 -0.11650
C  -3.79201 -2.01957  0.25801
C  -3.28119  0.35173 -0.15668
C  -5.10686 -1.66236  0.60971
C  -4.60091  0.68461  0.20114
C  -5.51040 -0.31654  0.58223
H  -3.47023 -3.06500  0.28120
H  -2.55662  1.11899 -0.45210
H  -5.81154 -2.44504  0.90826
H  -4.91317  1.73372  0.17849
H  -6.53460 -0.04823  0.85865

40
TS(26b-27b)
SCF(BP86)= -1047.34058742
G(298 K) = -1047.097613
```

SCF(PBE) = -3606.89398040
Low Freq. = -168.3862cm-1,
7.5750cm-1
C -1.64676 2.84973 -1.52943
C -1.48966 1.62350 -0.85623
N -0.27076 1.31045 -0.26521
C 0.75107 2.21034 -0.31756
C 0.62631 3.44154 -0.96407
C -0.59204 3.76632 -1.58788
C -2.48674 0.58016 -0.66393
C -3.80824 0.60512 -1.16211
C -4.66957 -0.46904 -0.90307
C -4.20992 -1.56893 -0.14670
C -2.89532 -1.60007 0.35026
C -2.00092 -0.52533 0.11263
Ru -0.14956 -0.36855 0.77545
O 1.52802 0.00931 2.52776
C 0.59606 0.68727 3.05542
C 0.73517 1.35646 4.40334
O -0.54710 0.80245 2.42587
H -2.60995 3.07952 -1.99341
H -4.16613 1.45884 -1.74984
H -5.69459 -0.45510 -1.28699
H -4.88453 -2.41018 0.05175
H -2.56085 -2.46714 0.93477
H -0.71802 4.72271 -2.10341
H 1.47649 4.12809 -0.97257
H 1.67297 1.90449 0.18251
H -0.11699 2.01882 4.61179
H 0.79243 0.58564 5.19078
H 1.67331 1.93299 4.43713
Br 0.45349 -2.37809 -0.79371
C 1.87605 -0.93514 -0.88787
C 1.90881 -0.17131 -2.07123
C 3.00277 -1.05797 -0.05166
C 3.07207 0.55951 -2.36395
C 4.14935 -0.30959 -0.36447
C 4.19152 0.49956 -1.51514
H 1.04167 -0.13361 -2.73529
H 2.97519 -1.69525 0.83460
H 3.09876 1.17075 -3.27205
H 5.02025 -0.37673 0.29603
H 5.09628 1.06519 -1.75656

46
16a
SCF(BP86)= -1180.17542502
G(298 K) = -1179.888636
SCF(PBE) = -3739.61512706
Low Freq. = 34.3916cm-1,
37.4847cm-1
C 0.57259 0.84087 1.32024
C 1.98353 0.91470 1.14071
C 2.77754 1.62338 2.07340
C 2.18464 2.23475 3.18283
C 0.79197 2.13390 3.37599
C -0.00824 1.44882 2.44819
C 2.52804 0.17922 -0.00049
N 1.58702 -0.55656 -0.68916
C 1.97248 -1.31643 -1.74704
C 3.29752 -1.36850 -2.18493
C 4.26634 -0.61058 -1.50498
C 3.87684 0.16290 -0.40814
Ru -0.33885 -0.52294 0.06338
N -2.17149 -0.62787 0.89241
C -3.22125 -0.82250 1.37675
C -4.52818 -1.04567 1.97784
Br -1.39218 -0.71939 -2.28126
H 3.86335 1.67864 1.94239
H 4.61329 0.75680 0.13839
H 5.31215 -0.62520 -1.82515
H 3.55594 -1.99147 -3.04459
H 1.17319 -1.87402 -2.23973
H 2.80312 2.77967 3.90248
H 0.32587 2.59719 4.25250
H -1.09028 1.39709 2.60041
H -4.41203 -1.38356 3.02036
H -5.11282 -0.11140 1.96895
H -5.07788 -1.81542 1.41230
O 0.31054 -1.70883 1.76633
C 0.09529 -2.82440 1.14704
O -0.34244 -2.73243 -0.05546
C 0.32223 -4.14325 1.82714
H 1.13175 -4.06069 2.56736
H -0.59924 -4.44308 2.35569
H 0.56223 -4.91785 1.08400
C -0.68784 1.54108 -0.48321
C -1.83443 2.24147 -0.05019
C 0.16903 2.19012 -1.40134
C -2.12232 3.53393 -0.52646
C -0.11532 3.48328 -1.87423
C -1.26460 4.16619 -1.44084
H -2.52131 1.78720 0.66852
H 1.06431 1.68448 -1.77393
H -3.02572 4.04495 -0.17340
H 0.56818 3.95310 -2.59115
H -1.48672 5.17307 -1.80947

46
16b
SCF(BP86)= -1180.16890970
G(298 K) = -1179.882193
SCF(PBE) = -3739.60864352
Low Freq. = 29.6492cm-1,
30.9781cm-1
C -1.80542 -0.53193 -0.43426
C -2.58082 0.64117 -0.56904
C -3.93518 0.54413 -0.96850
C -4.49966 -0.71034 -1.21971
C -3.72185 -1.87589 -1.07002
C -2.37061 -1.79070 -0.69087
C -1.89832 1.86922 -0.18723
N -0.61301 1.65041 0.24711
C 0.10547 2.69402 0.73759
C -0.40274 3.99360 0.78575
C -1.69937 4.23764 0.30180
C -2.45090 3.16486 -0.18268
Ru 0.14227 -0.32609 0.24335
N 0.22873 -0.93874 -1.68376

```
C   0.34158 -1.31994 -2.78363
C   0.48384 -1.80381 -4.14871
Br  1.25525 -2.62494  0.62207
H  -4.54524  1.44783 -1.06897
H  -3.46969  3.31627 -0.54751
H  -2.11975  5.24727  0.31295
H   0.21791  4.79587  1.19194
H   1.10829  2.45690  1.09330
H  -5.54687 -0.78515 -1.52802
H  -4.16532 -2.85926 -1.25981
H  -1.76627 -2.69714 -0.59363
H   1.07858 -2.73161 -4.15833
H  -0.50803 -2.01029 -4.58223
H   0.99434 -1.04709 -4.76615
O  -1.15451 -1.02383  2.12909
C  -0.28231 -0.43284  2.84195
O   0.66856  0.20370  2.23163
C  -0.30072 -0.47904  4.34862
H  -1.31329 -0.69911  4.71524
H   0.37837 -1.27865  4.69187
H   0.05772  0.47259  4.76928
C   1.82679  0.63522 -0.50769
C   2.99952  0.47377  0.26340
C   1.86720  1.47124 -1.64132
C   4.18175  1.15282 -0.09495
C   3.05073  2.14569 -1.99421
C   4.21408  1.98949 -1.22212
H   3.00076 -0.17134  1.14665
H   0.96950  1.61087 -2.25307
H   5.08122  1.01413  0.51598
H   3.05714  2.79554 -2.87674
H   5.13603  2.51233 -1.49697
```

```
46
16c
SCF(BP86)= -1180.16837732
G(298 K) = -1179.881969
SCF(PBE) = -3739.60663511
Low Freq. = 16.3055cm-1,
29.4896cm-1
C  -1.73672 -0.75238 -0.32554
C  -2.60679  0.36509 -0.39437
C  -3.98163  0.16769 -0.66849
C  -4.48306 -1.12638 -0.84405
C  -3.62022 -2.23572 -0.73706
C  -2.24973 -2.05019 -0.48804
C  -2.00805  1.66296 -0.08315
N  -0.68749  1.58537  0.30112
C  -0.05832  2.70699  0.74013
C  -0.68450  3.95496  0.77210
C  -2.01385  4.05778  0.32973
C  -2.67704  2.90268 -0.09325
Ru  0.18354 -0.30442  0.30671
N   0.94383 -2.15925  0.43194
C   1.37789 -3.24343  0.53526
C   1.91016 -4.59313  0.65282
Br  0.20124 -0.78362 -2.31539
H  -4.66197  1.02396 -0.72424
H  -3.71790  2.95090 -0.42185
H  -2.52922  5.02258  0.32668
H  -0.13089  4.82476  1.13382
H   0.97416  2.57295  1.06435
H  -5.54697 -1.27528 -1.05280
H  -4.01396 -3.25048 -0.86132
H  -1.58266 -2.91556 -0.43902
H   1.34632 -5.28033  0.00123
H   2.97080 -4.60834  0.35362
H   1.82880 -4.94217  1.69493
O  -0.91911 -0.79786  2.23590
C   0.04010 -0.28870  2.91638
O   1.02215  0.21646  2.25320
C   0.04708 -0.30816  4.42148
H  -0.98073 -0.34008  4.81039
H   0.57829 -1.21119  4.76867
H   0.57639  0.57203  4.81503
C   1.94767  0.55327 -0.42768
C   3.14161  0.04462  0.13004
C   2.03359  1.67564 -1.28130
C   4.38038  0.66614 -0.13013
C   3.27049  2.28960 -1.54466
C   4.45165  1.78907 -0.96810
H   3.12174 -0.83056  0.78604
H   1.13201  2.06726 -1.76210
H   5.29050  0.25498  0.32194
H   3.30760  3.15902 -2.21115
H   5.41557  2.26454 -1.17792
```

```
46
36d
SCF(BP86)= -1180.16639534
G(298 K) = -1179.879625
SCF(PBE) = -3739.60658343
Low Freq. = 31.0166cm-1,
36.7489cm-1
N  -1.47622 -0.53922 -1.00944
C  -2.51964  0.16768 -0.45204
C  -3.78026  0.17831 -1.08238
C  -3.97554 -0.53364 -2.26856
C  -2.90249 -1.25947 -2.81558
C  -1.67223 -1.24010 -2.15653
C  -2.17026  0.84188  0.79576
C  -0.80057  0.77595  1.18186
C  -0.41058  1.31311  2.42292
C  -1.35831  1.92492  3.25770
C  -2.70758  2.02433  2.86047
C  -3.11224  1.47958  1.63810
Ru  0.31135 -0.53822  0.02201
N   1.35906 -0.44005 -1.68970
C   1.98902 -0.49432 -2.67483
C   2.77437 -0.55766 -3.89656
Br  2.51676 -0.83227  1.39048
H  -4.59922  0.74912 -0.63815
H  -4.16685  1.52636  1.34674
H  -3.43932  2.51340  3.51062
H  -1.04113  2.33213  4.22403
H   0.63653  1.25514  2.73303
H  -4.95156 -0.52519 -2.76193
H  -3.00934 -1.83409 -3.73866
H  -0.80715 -1.79030 -2.53188
H   3.58074 -1.30021 -3.78304
H   2.13550 -0.84531 -4.74702
H   3.22350  0.42810 -4.09943
```

```
O   0.38447 -2.70150 -0.23415
C  -0.22731 -2.87815  0.88355
O  -0.56356 -1.80886  1.52796
C  -0.51221 -4.24334  1.43534
H  -0.64624 -4.96546  0.61634
H   0.34502 -4.57113  2.04873
H  -1.40644 -4.22139  2.07539
C   0.68986  1.58733 -0.32662
C   1.72728  2.28935  0.32195
C   0.00545  2.23796 -1.37782
C   2.06149  3.60094 -0.06171
C   0.33866  3.55071 -1.75822
C   1.36922  4.24289 -1.10165
H   2.28173  1.81081  1.13364
H  -0.79631  1.72663 -1.91858
H   2.87322  4.11936  0.46203
H  -0.21267  4.02713 -2.57714
H   1.62990  5.26450 -1.39732

46
16e
SCF(BP86)= -1180.16370479
G(298 K) = -1179.876872
SCF(PBE) = -3739.60415562
Low Freq. = 27.9404cm-1,
31.9401cm-1
C   1.06890 -0.72987  0.92885
C   2.36132 -0.28239  0.54006
C   3.51513 -0.81254  1.16802
C   3.38801 -1.75023  2.19660
C   2.10400 -2.15464  2.61684
C   0.95582 -1.64658  1.99048
C   2.39982  0.76098 -0.48200
N   1.15841  1.22917 -0.86342
C   1.07683  2.23056 -1.77777
C   2.20358  2.79758 -2.37495
C   3.47409  2.31828 -2.01353
C   3.56644  1.30130 -1.06096
Ru -0.50489  0.36105  0.03349
N  -0.14189  1.26557  1.78956
C   0.00091  1.87869  2.77554
C   0.17588  2.63400  4.00526
Br -2.66168 -0.53003  1.22726
H   4.51211 -0.47496  0.86608
H   4.54301  0.91486 -0.76004
H   4.37835  2.73488 -2.46611
H   2.07725  3.59819 -3.10753
H   0.07363  2.58371 -2.01756
H   4.28119 -2.15460  2.68248
H   1.99816 -2.87010  3.43986
H  -0.03552 -1.96942  2.32320
H  -0.71567  2.52995  4.64426
H   1.05661  2.25705  4.54994
H   0.33000  3.69972  3.77115
O  -1.74706  2.10431 -0.40109
C  -2.15119  1.56655 -1.49935
O  -1.61036  0.43404 -1.81177
C  -3.20467  2.19607 -2.36053
H  -4.19583  1.82442 -2.04736
H  -3.19253  3.28965 -2.24191
H  -3.05319  1.92310 -3.41546
C  -0.13453 -1.56184 -0.82182
C   0.56914 -1.58111 -2.04737
C  -0.75338 -2.75305 -0.38830
C   0.62611 -2.75185 -2.82674
C  -0.69371 -3.92005 -1.17031
C  -0.00370 -3.92914 -2.39414
H   1.06206 -0.68200 -2.42485
H  -1.29231 -2.77344  0.56144
H   1.16990 -2.73240 -3.77819
H  -1.19269 -4.82763 -0.81142
H   0.04451 -4.84049 -2.99914

46
16f
SCF(BP86)= -1180.16241886
G(298 K) = -1179.875570
SCF(PBE) = -3739.60219292
Low Freq. = 25.6510cm-1,
31.5328cm-1
C  -1.34058  0.30220  1.04195
C  -1.49117  1.71620  1.06342
C  -2.55611  2.31203  1.78515
C  -3.45807  1.51691  2.49358
C  -3.29947  0.11424  2.49219
C  -2.25959 -0.48406  1.76949
C  -0.47202  2.47050  0.34859
N   0.53562  1.68068 -0.16488
C   1.56579  2.27665 -0.82784
C   1.63935  3.65685 -1.01632
C   0.61237  4.47150 -0.50755
C  -0.44490  3.86981  0.17631
Ru  0.43665 -0.36114  0.19254
N   2.52845 -0.30126  0.58166
C   3.67109 -0.27043  0.83064
C   5.09571 -0.23587  1.12056
Br  1.36812 -0.78783 -2.21262
H  -2.66316  3.40164  1.80395
H  -1.25748  4.47566  0.58434
H   0.63838  5.55643 -0.64333
H   2.48864  4.07686 -1.56078
H   2.32695  1.60276 -1.22263
H  -4.27839  1.97956  3.05068
H  -3.99846 -0.51637  3.05246
H  -2.17549 -1.57486  1.75633
H   5.29165 -0.70253  2.09906
H   5.64566 -0.78833  0.34158
H   5.45051  0.80696  1.14008
O   0.60456 -0.77099  2.31248
C   0.61780 -2.04941  2.10131
O   0.47861 -2.42358  0.88149
C   0.76714 -3.02247  3.23457
H   1.07523 -2.50484  4.15365
H  -0.19910 -3.52482  3.41234
H   1.50443 -3.79668  2.97105
C  -1.31277 -0.80229 -0.95833
C  -1.83707 -2.11192 -0.94102
C  -1.86859  0.12759 -1.86512
C  -2.87216 -2.48428 -1.81985
C  -2.90335 -0.24521 -2.73988
C  -3.41308 -1.55571 -2.72395
H  -1.43104 -2.85464 -0.24824
```

H -1.48756 1.15315 -1.90354
H -3.25308 -3.51206 -1.79225
H -3.31030 0.49660 -3.43696
H -4.22010 -1.84654 -3.40462

46
16g
SCF(BP86)= -1180.16289192
G(298 K) = -1179.875899
SCF(PBE) = -3739.60263438
Low Freq. = 23.4928cm-1,
29.7957cm-1
C 1.95518 -0.12803 0.25132
C 2.44743 1.19795 0.24492
C 3.81493 1.45770 0.50292
C 4.68524 0.40177 0.78134
C 4.19205 -0.91745 0.80722
C 2.83496 -1.18280 0.55884
C 1.45299 2.20506 -0.07266
N 0.21116 1.66633 -0.29625
C -0.79483 2.49904 -0.68055
C -0.62092 3.87652 -0.82246
C 0.64003 4.43869 -0.56081
C 1.68301 3.59122 -0.18750
Ru -0.06765 -0.46946 -0.14791
N 0.18339 -1.05471 1.77256
C 0.26136 -1.46688 2.86461
C 0.35791 -1.98146 4.22243
Br -0.80991 -0.12994 -2.57350
H 4.19013 2.48607 0.48445
H 2.68259 3.98663 0.00781
H 0.80570 5.51563 -0.65582
H -1.47029 4.49018 -1.13223
H -1.75731 2.02956 -0.87656
H 5.74232 0.60030 0.98252
H 4.86600 -1.75141 1.03121
H 2.47526 -2.21322 0.58539
H 0.06306 -3.04313 4.24369
H 1.39341 -1.89001 4.58770
H -0.30947 -1.41202 4.88926
O 1.03328 -2.28004 -1.28427
C 0.01732 -2.98350 -0.98812
O -0.90439 -2.44237 -0.25392
C -0.16496 -4.39515 -1.48737
H 0.80866 -4.85403 -1.71103
H -0.71038 -5.00087 -0.74815
H -0.76169 -4.37269 -2.41577
C -1.80221 0.23232 0.76429
C -3.01151 -0.28234 0.24682
C -1.85024 1.09215 1.88044
C -4.24187 0.07235 0.83718
C -3.08087 1.44050 2.46603
C -4.28361 0.93350 1.94507
H -3.00646 -0.95947 -0.61211
H -0.92346 1.49961 2.29858
H -5.16992 -0.33205 0.41686
H -3.09381 2.10978 3.33384
H -5.24227 1.20581 2.39864

46
16h
SCF(BP86)= -1180.15768493
G(298 K) = -1179.871021
SCF(PBE) = -3739.59687955
Low Freq. = 25.4795cm-1,
28.9891cm-1
C 1.94370 -0.00427 -0.04284
C 2.33920 -1.36393 -0.03680
C 3.69882 -1.72473 -0.20292
C 4.66742 -0.73351 -0.36510
C 4.27930 0.62159 -0.36017
C 2.93083 0.98642 -0.21820
C 1.26364 -2.29747 0.22929
N 0.06035 -1.66764 0.42323
C -1.00290 -2.40960 0.83669
C -0.93188 -3.79099 1.01910
C 0.27895 -4.45362 0.75343
C 1.38278 -3.69652 0.36160
Ru -0.07381 0.47725 0.20284
N -0.70005 0.27736 2.10325
C -1.07769 0.21277 3.21052
C -1.53602 0.13477 4.58822
Br 0.43812 1.11140 -2.28166
H 3.99005 -2.78014 -0.19261
H 2.34852 -4.17167 0.17368
H 0.36119 -5.53842 0.86581
H -1.82083 -4.33047 1.35445
H -1.93081 -1.86568 1.01144
H 5.71925 -1.00723 -0.49218
H 5.03134 1.40820 -0.48606
H 2.65509 2.04140 -0.24105
H -0.83133 0.66597 5.24841
H -2.53128 0.59945 4.67874
H -1.60169 -0.91866 4.90441
O 1.00818 2.27071 1.34764
C 0.04229 3.00847 0.96426
O -0.85741 2.47435 0.20286
C -0.05622 4.46584 1.33805
H 0.49092 4.66023 2.27166
H 0.39824 5.07400 0.53682
H -1.10791 4.77158 1.44124
C -1.82491 -0.16232 -0.71741
C -2.99679 0.44365 -0.21326
C -1.94420 -1.09983 -1.76392
C -4.25720 0.12443 -0.76062
C -3.20283 -1.41525 -2.30534
C -4.36690 -0.80355 -1.80706
H -2.94476 1.17770 0.59681
H -1.04896 -1.58316 -2.16709
H -5.15260 0.60862 -0.35398
H -3.26867 -2.14181 -3.12333
H -5.34697 -1.05069 -2.22818

46
16i
SCF(BP86)= -1180.15909121
G(298 K) = -1179.872426
SCF(PBE) = -3739.59629930
Low Freq. = 22.1688cm-1,
24.4093cm-1
C 1.62168 0.23228 -0.98783
C 1.95705 1.60320 -0.86959

```
C   3.08846  2.12894 -1.53992
C   3.89335  1.29030 -2.31301
C   3.57491 -0.07909 -2.41572
C   2.45637 -0.60735 -1.75213
C   1.04368  2.37603 -0.04921
N   0.00781  1.61926  0.44269
C  -0.97254  2.24630  1.15070
C  -0.93610  3.61022  1.44139
C   0.14466  4.38197  0.97988
C   1.13558  3.75649  0.22384
Ru -0.02662 -0.47867  0.06596
N   1.78122 -1.32489  1.06107
C   2.67603 -1.83311  1.61813
C   3.78411 -2.46741  2.31863
Br -0.76327 -0.63214  2.53995
H   3.33220  3.19284 -1.45313
H   1.98111  4.32574 -0.16965
H   0.20382  5.45226  1.19729
H  -1.74960  4.05186  2.02218
H  -1.79098  1.61461  1.49286
H   4.76811  1.69408 -2.83182
H   4.20598 -0.74544 -3.01385
H   2.23970 -1.67416 -1.84180
H   3.64862 -3.56081  2.32238
H   4.73532 -2.22565  1.81809
H   3.82492 -2.10906  3.35977
O  -0.19657 -1.37658 -1.93324
C  -0.47643 -2.52302 -1.41756
O  -0.44609 -2.57347 -0.13050
C  -0.84451 -3.70613 -2.26119
H  -0.44502 -3.59415 -3.27933
H  -0.46496 -4.63175 -1.80260
H  -1.94419 -3.77932 -2.32120
C  -1.92214  0.01185 -0.60211
C  -3.04820 -0.69252 -0.11719
C  -2.13329  0.98928 -1.60232
C  -4.34131 -0.41370 -0.60516
C  -3.42253  1.26352 -2.09234
C  -4.53577  0.56497 -1.59247
H  -2.92629 -1.46016  0.65372
H  -1.28064  1.54161 -2.01347
H  -5.19720 -0.97042 -0.20559
H  -3.55412  2.02595 -2.86915
H  -5.54097  0.78004 -1.97042
```

```
46
16j
SCF(BP86)= -1180.15641373
G(298 K) = -1179.870343
SCF(PBE) = -3739.59391028
Low Freq. = 26.1507cm-1,
28.4057cm-1
N   0.82798  0.65128  1.35250
C   2.18070  0.44765  1.20972
C   3.06744  1.05540  2.12661
C   2.57096  1.83069  3.17425
C   1.17896  1.99732  3.31096
C   0.34016  1.39621  2.37458
C   2.53470 -0.43221  0.10487
C   1.41564 -0.96493 -0.61429
C   1.69707 -1.87867 -1.65740
C   3.01877 -2.21837 -1.99037
C   4.10742 -1.66270 -1.28579
C   3.86564 -0.77322 -0.23514
Ru -0.40513 -0.40129  0.04585
N  -2.40312 -0.00932  0.85189
C  -3.49154  0.00681  1.28667
C  -4.84490  0.03975  1.82326
Br -1.43340 -0.53020 -2.30705
H   4.14303  0.90183  2.01132
H   4.70886 -0.35064  0.32232
H   5.13429 -1.93010 -1.55419
H   3.20582 -2.92301 -2.80911
H   0.86963 -2.31855 -2.22255
H   3.25783  2.29915  3.88503
H   0.74651  2.58511  4.12375
H  -0.74592  1.49838  2.41410
H  -4.81585 -0.01674  2.92324
H  -5.34436  0.97577  1.52576
H  -5.42462 -0.81349  1.43596
O  -0.28420 -1.61475  1.80648
C  -0.74100 -2.67218  1.21265
O  -1.00129 -2.53661 -0.03450
C  -0.97318 -3.94652  1.96565
H  -0.22260 -4.06355  2.76180
H  -1.97158 -3.91692  2.43545
H  -0.93594 -4.80465  1.27870
C  -0.30815  1.62524 -0.65754
C  -1.23822  2.59408 -0.22834
C   0.71905  2.03251 -1.53819
C  -1.15497  3.92695 -0.67802
C   0.80120  3.36312 -1.98639
C  -0.13584  4.31989 -1.55907
H  -2.04624  2.32341  0.45630
H   1.45223  1.30552 -1.89820
H  -1.89763  4.65551 -0.33247
H   1.60348  3.64733 -2.67738
H  -0.07195  5.35558 -1.90907
```

```
46
16k
SCF(BP86)= -1180.15824076
G(298 K) = -1179.871266
SCF(PBE) = -3739.59539862
Low Freq. = 21.8326cm-1,
26.8116cm-1
N  -1.91346 -0.97095 -0.01373
C  -2.94209 -0.07255  0.07356
C  -4.28637 -0.49559  0.01557
C  -4.57386 -1.85882 -0.08775
C  -3.51136 -2.77809 -0.12464
C  -2.20099 -2.29114 -0.09138
C  -2.48117  1.29697  0.27810
C  -1.07665  1.47520  0.35853
C  -0.55621  2.75461  0.61837
C  -1.41905  3.84702  0.80563
C  -2.81421  3.67691  0.71654
C  -3.34053  2.40861  0.45761
Ru  0.19815 -0.15330  0.07419
N   0.40752  1.00889 -1.56554
```

```
C   0.53533  1.62928 -2.54908
C   0.69796  2.39691 -3.77486
Br  0.62659 -1.94685 -1.73143
H  -5.09341  0.23966  0.06430
H  -4.42555  2.27247  0.40100
H  -3.48464  4.53075  0.85392
H  -0.99687  4.83530  1.01758
H   0.52498  2.90341  0.68905
H  -5.61177 -2.20246 -0.12839
H  -3.68774 -3.85503 -0.18370
H  -1.33957 -2.96048 -0.14069
H   1.73215  2.76952 -3.85360
H   0.48016  1.76182 -4.64886
H   0.00697  3.25531 -3.77683
O   0.50541  0.52600  2.15490
C   0.48219 -0.69529  2.55749
O   0.26557 -1.57612  1.63886
C   0.72038 -1.07372  3.98756
H   1.75939 -1.42829  4.10030
H   0.56798 -0.20742  4.64668
H   0.04776 -1.89533  4.27816
C   2.27294  0.13141  0.19827
C   2.91045  1.37654 -0.00095
C   3.08792 -0.96698  0.56514
C   4.30001  1.52609  0.16940
C   4.47917 -0.82224  0.72851
C   5.09333  0.42580  0.53343
H   2.32556  2.25543 -0.29291
H   2.63971 -1.95349  0.72163
H   4.75945  2.50926  0.01317
H   5.08152 -1.69467  1.00818
H   6.17520  0.53897  0.66123

46
16l
SCF(BP86)= -1180.15398225
G(298 K) = -1179.867014
SCF(PBE) = -3739.59423456
Low Freq. = 27.7344cm-1,
33.6974cm-1
N  -0.09807  1.22103  1.21396
C   1.03503  1.97599  0.98267
C   1.38441  3.01614  1.86960
C   0.59349  3.28345  2.98830
C  -0.54791  2.49390  3.21850
C  -0.85668  1.47826  2.31381
C   1.78200  1.57316 -0.20346
C   1.28428  0.42872 -0.88981
C   1.91793  0.02901 -2.08411
C   3.02585  0.73191 -2.57538
C   3.53374  1.85041 -1.87915
C   2.91046  2.26958 -0.70122
Ru -0.54742 -0.27402 -0.12073
N  -2.59328  0.06528  0.18312
C  -3.73434  0.23161  0.38124
C  -5.15302  0.45464  0.60962
Br -1.18538  1.37997 -2.03382
H   2.28426  3.60510  1.67622
H   3.28873  3.15166 -0.17361
H   4.40384  2.39171 -2.26295
H   3.49955  0.40749 -3.50859
H   1.54027 -0.83569 -2.63844
H   0.86453  4.08830  3.67742
H  -1.19214  2.65395  4.08637
H  -1.72551  0.83206  2.45283
H  -5.70803 -0.48702  0.47069
H  -5.31879  0.82306  1.63468
H  -5.53010  1.20217 -0.10708
O  -1.23469 -2.01696  1.00496
C  -1.44167 -2.66537 -0.09989
O  -1.15528 -2.06467 -1.19815
C  -1.95591 -4.07676 -0.07376
H  -2.54628 -4.25948  0.83576
H  -2.56038 -4.27986 -0.96999
H  -1.09503 -4.76797 -0.07355
C   1.20488 -1.37831  0.42585
C   1.63966 -2.49419 -0.32591
C   1.75712 -1.18921  1.71240
C   2.58478 -3.39355  0.19554
C   2.70401 -2.09268  2.23165
C   3.12575 -3.19911  1.47839
H   1.24040 -2.67038 -1.32820
H   1.45177 -0.34411  2.33452
H   2.90069 -4.24925 -0.41212
H   3.10797 -1.92227  3.23619
H   3.86520 -3.89826  1.88221

46
16m
SCF(BP86)= -1180.15330978
G(298 K) = -1179.866941
SCF(PBE) = -3739.59150994
Low Freq. = 15.2523cm-1,
31.3054cm-1
N  -1.01587  0.66046  1.07624
C  -2.32483  0.34268  0.81136
C  -3.34677  0.93420  1.58671
C  -3.02112  1.80966  2.62298
C  -1.66746  2.08841  2.89229
C  -0.69149  1.49389  2.09397
C  -2.49844 -0.63013 -0.26145
C  -1.28439 -1.13864 -0.82471
C  -1.39432 -2.12096 -1.83333
C  -2.65114 -2.55686 -2.28712
C  -3.83700 -2.03148 -1.73686
C  -3.76061 -1.07347 -0.72121
Ru  0.43392 -0.37943 -0.07269
N   0.27567 -1.44570  1.64528
C   0.22806 -2.13920  2.58541
C   0.19211 -2.99831  3.75644
Br  2.88167  0.35704  1.10436
H  -4.39031  0.68998  1.37470
H  -4.68210 -0.67052 -0.28737
H  -4.81217 -2.37344 -2.09677
H  -2.70613 -3.31462 -3.07686
H  -0.49741 -2.56137 -2.27914
H  -3.81195  2.26640  3.22492
H  -1.36789  2.75487  3.70426
H   0.37802  1.66394  2.24306
H   1.15502 -2.95191  4.28997
H  -0.61439 -2.67077  4.43211
H  -0.00226 -4.03791  3.44677
```

```
O   1.57483 -2.07602 -0.68046
C   1.85466 -1.56505 -1.82918
O   1.31695 -0.41439 -2.05278
C   2.75834 -2.23655 -2.81367
H   3.79963 -1.92539 -2.62033
H   2.69819 -3.32950 -2.70361
H   2.49378 -1.93994 -3.83934
C   0.21612  1.58381 -0.87684
C  -0.63967  1.74350 -1.98699
C   0.92414  2.69605 -0.38729
C  -0.76020  2.99924 -2.61257
C   0.79461  3.94952 -1.01677
C  -0.04667  4.10777 -2.12966
H  -1.19861  0.89392 -2.38625
H   1.59565  2.58817  0.46856
H  -1.42112  3.10149 -3.48077
H   1.36249  4.80209 -0.62723
H  -0.14580  5.08446 -2.61471

46
16n
SCF(BP86)= -1180.15400035
G(298 K) = -1179.867505
SCF(PBE) = -3739.59232622
Low Freq. = 30.5221cm-1,
36.8864cm-1
N  -1.01978  0.53385 -1.23196
C  -2.33628  0.26248 -0.93854
C  -3.34541  0.77599 -1.78185
C  -3.00243  1.53021 -2.90478
C  -1.64453  1.76766 -3.19227
C  -0.67871  1.25575 -2.32725
C  -2.52412 -0.57773  0.23803
C  -1.31370 -1.02153  0.85690
C  -1.41857 -1.87413  1.97681
C  -2.67636 -2.25241  2.47783
C  -3.86207 -1.79480  1.86753
C  -3.78720 -0.96153  0.74673
Ru  0.39369 -0.40525 -0.02174
N   1.36187 -0.32999  1.74580
C   1.96288 -0.37785  2.74821
C   2.70730 -0.42017  3.99535
Br  2.77923  0.12204 -1.37476
H  -4.39312  0.56881 -1.55152
H  -4.70898 -0.61242  0.26886
H  -4.83725 -2.09183  2.26540
H  -2.73418 -2.91024  3.35251
H  -0.51256 -2.24607  2.46643
H  -3.78451  1.92678 -3.55863
H  -1.33409  2.33952 -4.06962
H   0.39418  1.39846 -2.48216
H   3.78821 -0.47414  3.78833
H   2.49920  0.49218  4.57817
H   2.40648 -1.30018  4.58630
O   0.09622 -1.75282 -1.64896
C   0.74471 -2.70148 -1.05490
O   1.17717 -2.41554  0.12344
C   0.99825 -4.01951 -1.71189
H   0.17203 -4.27175 -2.39338
H   1.92848 -3.95645 -2.30293
H   1.12025 -4.80597 -0.95247
C   0.22676  1.62841  0.67620
C   0.97392  2.66654  0.08650
C  -0.64293  1.93768  1.74357
C   0.84094  3.99104  0.54604
C  -0.77052  3.26258  2.20157
C  -0.03166  4.29625  1.60312
H   1.66814  2.44393 -0.72910
H  -1.21966  1.14812  2.23307
H   1.43070  4.78350  0.07108
H  -1.45095  3.47979  3.03283
H  -0.13133  5.32685  1.95969

46
16o
SCF(BP86)= -1180.15302055
G(298 K) = -1179.866918
Low Freq. = 24.8061cm-1,
28.7871cm-1
N  -1.76115 -0.83413 -0.23691
C  -2.69727  0.16940 -0.31328
C  -4.03810 -0.16404 -0.60894
C  -4.40483 -1.49686 -0.79668
C  -3.42841 -2.50542 -0.68721
C  -2.11342 -2.13069 -0.41629
C  -2.17825  1.50270 -0.02486
C  -0.78839  1.54990  0.31969
C  -0.28182  2.79884  0.75100
C  -1.09021  3.94816  0.78125
C  -2.44136  3.88896  0.38469
C  -2.98767  2.66383 -0.00961
Ru  0.16745 -0.23337  0.30518
N   1.02314 -2.22480  0.44834
C   1.43335 -3.31483  0.58614
C   1.94662 -4.66769  0.75159
Br  0.21392 -0.60827 -2.32244
H  -4.78505  0.63003 -0.67936
H  -4.04474  2.60997 -0.29238
H  -3.06338  4.78946  0.39861
H  -0.66319  4.90095  1.11551
H   0.76515  2.87682  1.05962
H  -5.44386 -1.75344 -1.02333
H  -3.67334 -3.56173 -0.81907
H  -1.30334 -2.85832 -0.34759
H   1.12026 -5.39475  0.69933
H   2.67301 -4.89400 -0.04544
H   2.44635 -4.76391  1.72892
O  -0.66923 -0.62518  2.24385
C   0.34658 -0.13015  2.86612
O   1.27018  0.34686  2.11259
C   0.45067 -0.15223  4.36110
H  -0.55030 -0.10859  4.81488
H   0.93488 -1.09177  4.67914
H   1.06500  0.68961  4.71226
C   1.95037  0.64483 -0.43570
C   3.15883  0.00643 -0.08407
C   2.01432  1.86306 -1.14409
C   4.40019  0.59530 -0.39683
C   3.25432  2.44395 -1.46297
C   4.45503  1.81556 -1.08771
H   3.14741 -0.94587  0.45212
H   1.09519  2.35627 -1.46972
```

H 5.32400 0.08461 -0.10105
H 3.27620 3.38970 -2.01673
H 5.42001 2.26778 -1.34003

46
16p
SCF(BP86)= -1180.15006404
G(298 K) = -1179.863761
SCF(PBE) = -3739.58682459
Low Freq. = 20.1618cm-1, 29.2471cm-1
N 1.84760 1.05266 0.34662
C 2.92041 0.23084 0.13613
C 4.23623 0.73952 0.15676
C 4.44502 2.09407 0.42785
C 3.33620 2.92132 0.68081
C 2.05752 2.35978 0.62472
C 2.52744 -1.16050 -0.04663
C 1.13069 -1.41883 -0.05454
C 0.68233 -2.75176 -0.10340
C 1.60100 -3.81008 -0.17003
C 2.98691 -3.55410 -0.20348
C 3.44502 -2.23707 -0.13604
Ru -0.21316 0.15598 0.17313
N -0.45906 1.83057 -0.92182
C -0.67134 2.83329 -1.48699
C -0.93622 4.06680 -2.20842
Br -0.46718 -0.72033 -2.35534
H 5.08250 0.07477 -0.03377
H 4.52118 -2.03471 -0.13087
H 3.70036 -4.38124 -0.26844
H 1.22992 -4.84005 -0.20650
H -0.38835 -2.97090 -0.09730
H 5.46000 2.50165 0.44916
H 3.45418 3.98219 0.91475
H 1.16331 2.95941 0.81134
H -1.99392 4.34996 -2.08298
H -0.29719 4.87851 -1.82553
H -0.73148 3.92029 -3.28149
O -0.45245 -1.06749 1.97489
C -0.53694 -0.00408 2.68992
O -0.39957 1.11187 2.04781
C -0.80664 -0.03931 4.16321
H -1.88020 0.14609 4.34000
H -0.54522 -1.02313 4.57786
H -0.23660 0.75258 4.67249
C -2.27491 -0.14337 0.18683
C -2.90666 -1.38138 -0.07109
C -3.10257 0.94608 0.55188
C -4.30165 -1.52867 0.03112
C -4.50198 0.80466 0.64537
C -5.10905 -0.43399 0.38724
H -2.30748 -2.24784 -0.36916
H -2.66263 1.92495 0.77358
H -4.75702 -2.50474 -0.17437
H -5.11321 1.67060 0.92599
H -6.19593 -0.54680 0.46194

46
16q
SCF(BP86)= -1180.14613904
G(298 K) = -1179.859508
SCF(PBE) = -3739.58303880
Low Freq. = 12.2797cm-1, 20.4564cm-1
C 1.63676 -1.09820 0.47950
C 2.75665 -0.24279 0.27412
C 4.06197 -0.72708 0.52050
C 4.25031 -2.02952 1.00174
C 3.13868 -2.85502 1.24850
C 1.83487 -2.39002 0.98834
C 2.46954 1.14298 -0.13408
N 1.13427 1.43853 -0.31020
C 0.74672 2.71095 -0.56659
C 1.66740 3.75265 -0.71176
C 3.03550 3.46531 -0.59035
C 3.43305 2.15725 -0.29536
Ru -0.11614 -0.23388 -0.15091
N -0.02161 0.29404 1.79933
C 0.15359 0.59588 2.91696
C 0.34323 0.96544 4.31197
Br -1.71228 0.98074 -2.06927
H 4.93439 -0.08904 0.34576
H 4.49199 1.91888 -0.17378
H 3.78472 4.25268 -0.71289
H 1.30723 4.76206 -0.92366
H -0.32793 2.86177 -0.68538
H 5.26309 -2.39719 1.19303
H 3.28487 -3.86801 1.63916
H 0.97677 -3.04467 1.16825
H 1.11656 0.32925 4.77219
H 0.65797 2.01906 4.38518
H -0.60143 0.83666 4.86501
O 0.48227 -1.08760 -2.01550
C -0.08288 -2.21690 -1.74326
O -0.69422 -2.28555 -0.61290
C -0.05668 -3.36611 -2.70712
H 0.84647 -3.32136 -3.33318
H -0.09887 -4.32185 -2.16424
H -0.93879 -3.30568 -3.36810
C -2.12929 -0.17642 0.66634
C -2.85895 -1.35765 0.86324
C -2.67168 1.05427 1.08625
C -4.14626 -1.29752 1.44248
C -3.95305 1.10581 1.66005
C -4.69989 -0.07283 1.83864
H -2.45643 -2.32598 0.56202
H -2.10442 1.98107 0.96044
H -4.70659 -2.22925 1.58149
H -4.36261 2.07464 1.96771
H -5.69754 -0.03367 2.28817

46
16r
SCF(BP86)= -1180.14239246
G(298 K) = -1179.855926
SCF(PBE) = -3739.58084544
Low Freq. = 29.1893cm-1, 31.9828cm-1
C 0.87133 -1.45233 -0.17562
C 2.28475 -1.32068 -0.14201
C 3.09325 -2.47737 -0.27538

C   2.51117 -3.73910 -0.42617
C   1.10782 -3.86302 -0.44711
C   0.29461 -2.72592 -0.31776
C   2.83635 0.03104 0.00201
N   1.89219 1.02221 0.09715
C   2.30081 2.30766 0.20287
C   3.64726 2.68186 0.23102
C   4.62202 1.67397 0.14881
C   4.21237 0.34330 0.03238
Ru -0.22912 0.33044 0.09230
N -0.13962 -0.59411 1.86124
C -0.06749 -0.98561 2.96214
C   0.01648 -1.48976 4.32320
Br -0.24423 -0.10943 -2.43901
H   4.18435 -2.38854 -0.26276
H   4.95540 -0.45455 -0.03734
H   5.68702 1.92301 0.17156
H   3.91706 3.73738 0.31581
H   1.51389 3.06299 0.25911
H   3.14575 -4.62496 -0.52716
H   0.64451 -4.84856 -0.56673
H -0.79363 -2.83026 -0.34598
H -0.98177 -1.79240 4.67885
H   0.69165 -2.36021 4.35788
H   0.41234 -0.70528 4.98849
O -0.66726 1.90605 1.47281
C -0.95154 2.74214 0.52664
O -0.80499 2.28967 -0.67022
C -1.44177 4.12532 0.81617
H -2.54528 4.11808 0.84543
H -1.07059 4.46897 1.79308
H -1.12405 4.81559 0.02026
C -2.25108 -0.23341 0.05448
C -3.19938 0.53633 -0.67055
C -2.76408 -1.36313 0.74226
C -4.56648 0.20131 -0.70429
C -4.12932 -1.70762 0.70881
C -5.04355 -0.92499 -0.01377
H -2.86641 1.41176 -1.23596
H -2.09298 -2.00664 1.32045
H -5.25870 0.82588 -1.28225
H -4.47389 -2.59468 1.25426
H -6.10637 -1.18905 -0.04106

46
TS(14a-16b)
SCF(BP86)= -1180.12466577
G(298 K) = -1179.838353
SCF(PBE) = -3739.56384439
Low Freq. = -187.2637cm-1,
24.1984cm-1
C   2.17059 -0.36711 -0.15634
C   2.73353 0.93730 -0.21727
C   4.13106 1.13959 -0.13768
C   4.99063 0.04500 0.00136
C   4.44884 -1.25303 0.06089
C   3.05926 -1.45858 -0.01097
C   1.76232 2.00372 -0.42362
N   0.45006 1.56958 -0.49827
C -0.48708 2.47634 -0.88351
C -0.20505 3.82493 -1.12109
C   1.10777 4.28785 -0.95562
C   2.09355 3.35974 -0.61706
Ru 0.14524 -0.52276 -0.04862
N   0.38332 -0.13153 1.86839
C   0.56501 0.09745 3.00677
C   0.77489 0.36362 4.42412
Br -2.05365 -1.86770 0.85408
H   4.54932 2.15080 -0.18464
H   3.13674 3.67076 -0.52345
H   1.36223 5.34019 -1.11214
H -1.01697 4.48900 -1.42744
H -1.49709 2.10337 -1.02130
H   6.07257 0.19913 0.06175
H   5.11385 -2.11817 0.16614
H   2.66652 -2.47720 0.04498
H   0.47569 -0.51170 5.02404
H   1.83943 0.57485 4.61740
H   0.17866 1.23337 4.74590
O   0.49325 -2.65127 -0.44869
C   0.23158 -2.45516 -1.69561
O -0.06070 -1.24628 -2.04507
C   0.21951 -3.58562 -2.68692
H   0.96510 -4.34595 -2.41110
H -0.77514 -4.06483 -2.68125
H   0.41649 -3.20994 -3.70162
C -2.22429 0.20920 0.04922
C -2.84823 0.21280 -1.21675
C -2.60259 1.14304 1.03734
C -3.79793 1.20743 -1.51343
C -3.55753 2.12508 0.72568
C -4.15905 2.16504 -0.54704
H -2.57834 -0.53922 -1.96364
H -2.15286 1.10738 2.03335
H -4.26057 1.22355 -2.50649
H -3.83412 2.86080 1.48907
H -4.90930 2.92706 -0.77886

46
TS(14a-16i)
SCF(BP86)= -1180.12716066
G(298 K) = -1179.841139
SCF(PBE) = -3739.56596078
Low Freq. = -193.3799cm-1,
23.0861cm-1
C -3.78928 -0.85024 -1.57951
C -2.57336 -0.33648 -1.07198
C -1.84808 -1.04345 -0.06761
C -2.40597 -2.23197 0.44970
C -3.62550 -2.72769 -0.04462
C -4.30974 -2.04641 -1.07053
C -2.01702 0.96808 -1.43752
C -2.60686 1.89278 -2.32233
C -2.06305 3.17163 -2.47097
C -0.93951 3.51914 -1.70274
C -0.37363 2.55653 -0.86298
N -0.85753 1.29003 -0.75649
Ru -0.07511 -0.22616 0.44229
O   0.59206 -2.04287 1.37413

```
C   0.75430 -2.63236  0.23613
C   1.16078 -4.07923  0.15865
O   0.52745 -1.93573 -0.82233
N  -0.94151  0.67479  1.96195
C  -1.47604  1.17401  2.88107
C  -2.13482  1.76083  4.03892
H  -4.33803 -0.31391 -2.36145
H  -2.19929  2.85559  3.92908
H  -3.15537  1.35488  4.13474
H  -1.57175  1.52618  4.95717
H  -3.50616  1.60790 -2.87417
H  -2.52015  3.89228 -3.15517
H  -0.49737  4.51751 -1.74891
H   0.49837  2.78016 -0.24269
H  -5.25201 -2.44247 -1.46221
H  -4.04216 -3.65418  0.36672
H  -1.87863 -2.78349  1.23548
H   1.59303 -4.30739 -0.82606
H   1.88415 -4.31668  0.95367
H   0.27230 -4.71751  0.30772
Br  1.99366  1.36429  1.49626
C   2.22275  0.13956 -0.41239
C   2.26469  0.82541 -1.63927
C   3.16173 -0.87924 -0.13708
C   3.17495  0.40821 -2.63034
C   4.06889 -1.27374 -1.13170
C   4.07944 -0.63744 -2.38688
H   1.59553  1.66050 -1.85257
H   3.17943 -1.36367  0.84244
H   3.17300  0.92538 -3.59646
H   4.78441 -2.07340 -0.90987
H   4.79704 -0.93949 -3.15594
```

```
46
TS(14a-16k)
SCF(BP86)= -1180.12872600
G(298 K) = -1179.842729
SCF(PBE) = -3739.56672897
Low Freq. = -182.8760cm-1,
13.2243cm-1
N   1.68261 -1.11573 -0.53099
C   2.90197 -0.48643 -0.35507
C   4.10285 -1.17303 -0.62413
C   4.07765 -2.48483 -1.10312
C   2.83284 -3.09472 -1.32269
C   1.66950 -2.38063 -1.02701
C   2.80479  0.90807  0.06875
C   1.47898  1.42830  0.17440
C   1.32964  2.76829  0.59386
C   2.45104  3.56845  0.87555
C   3.75415  3.04984  0.75105
C   3.92806  1.72116  0.35007
Ru -0.00286  0.08468 -0.13542
N   0.18855 -0.28647  1.79466
C   0.39304 -0.50858  2.93070
C   0.62370 -0.78495  4.34282
Br -1.49863 -2.30506 -0.13785
H   5.05582 -0.66156 -0.46938
H   4.94128  1.31460  0.26091
H   4.62464  3.67634  0.96929
H   2.30481  4.60649  1.19589
H   0.32737  3.19398  0.69284
H   5.01019 -3.01521 -1.31648
H   2.74972 -4.11034 -1.71755
H   0.68302 -2.82250 -1.17543
H  -0.32100 -1.06996  4.83459
H   1.34450 -1.61101  4.45894
H   1.02697  0.10931  4.84587
O  -1.12462  1.95240 -0.58158
C  -0.91353  1.75396 -1.83300
O  -0.26879  0.67887 -2.15530
C  -1.42524  2.70028 -2.88395
H  -2.45628  2.41501 -3.15667
H  -1.44863  3.72904 -2.49500
H  -0.80038  2.64945 -3.78772
C  -2.42695 -0.28773  0.16236
C  -2.86616 -0.00426  1.46996
C  -3.25013  0.01229 -0.94607
C  -4.08449  0.67617  1.65257
C  -4.46672  0.68128 -0.74256
C  -4.89108  1.02210  0.55525
H  -2.27150 -0.29470  2.33802
H  -2.94274 -0.27296 -1.95603
H  -4.40423  0.91937  2.67222
H  -5.09284  0.91918 -1.60980
H  -5.84701  1.53191  0.70963
```

```
46
TS(14a-16q)
SCF(BP86)= -1180.13263099
G(298 K) = -1179.846493
SCF(PBE) = -3739.57112292
Low Freq. = -186.1661cm-1,
23.2307cm-1
C   4.21495  0.46749  0.69739
C   2.90275  0.09047  0.33053
C   1.79727  0.95050  0.61055
C   2.04089  2.15300  1.30405
C   3.34832  2.51446  1.67858
C   4.43698  1.67793  1.36691
C   2.56942 -1.19050 -0.30452
C   3.48658 -2.21558 -0.60784
C   3.04031 -3.42819 -1.14184
C   1.66412 -3.60669 -1.35556
C   0.79353 -2.55653 -1.05307
N   1.22230 -1.36384 -0.56447
Ru  0.04049  0.28801 -0.13143
O  -0.67262  2.35129 -0.07737
C  -0.12976  2.57973 -1.22338
C  -0.22139  3.92943 -1.88272
O   0.47353  1.58986 -1.79163
N  -0.16057 -0.51621  1.66720
C  -0.19157 -0.95616  2.75522
C  -0.26059 -1.48477  4.11096
H   5.06605 -0.18328  0.46872
H   0.46211 -2.30708  4.23929
H  -0.02991 -0.69377  4.84368
H  -1.27364 -1.86968  4.31277
H   4.54990 -2.05903 -0.41059
H   3.75249 -4.22435 -1.37701
H   1.25972 -4.54080 -1.75327
H  -0.28189 -2.63869 -1.22111
```

H   5.45389 1.96565 1.65191
H   3.51947 3.45643 2.21215
H   1.20510 2.81951 1.54074
H   0.50532 4.00976 -2.70353
H -1.23649 4.07027 -2.29262
H -0.04593 4.72666 -1.14377
Br -1.78581 -0.49424 -2.01460
C -2.39523 -0.17340 0.15093
C -3.16599 0.97894 0.37557
C -2.74301 -1.40268 0.74619
C -4.23630 0.91588 1.28825
C -3.81869 -1.44764 1.64849
C -4.56901 -0.29044 1.92779
H -2.92030 1.91772 -0.12487
H -2.17706 -2.31156 0.52380
H -4.81756 1.82416 1.48374
H -4.07533 -2.40219 2.12145
H -5.41496 -0.33414 2.62082

46
TS(14b-16a)
SCF(BP86)= -1180.13256731
G(298 K) = -1179.847854
SCF(PBE) = -3739.57563272
Low Freq. = -177.3503cm-1,
19.7109cm-1
C   0.46023 -0.63225 1.33316
C -0.00985 -1.99030 1.31279
C   0.49098 -2.94886 2.22444
C   1.46462 -2.58697 3.16225
C   1.94113 -1.25935 3.19172
C   1.44934 -0.30044 2.29095
C -1.03299 -2.27959 0.30827
N -1.40070 -1.17197 -0.43286
C -2.34520 -1.29913 -1.40158
C -2.96326 -2.51727 -1.69434
C -2.59753 -3.65460 -0.95136
C -1.63152 -3.53020 0.05177
Ru -0.49781 0.60074 0.10697
N   0.34834 2.28315 0.73649
C   0.78161 3.30926 1.11885
C   1.32965 4.57097 1.60028
Br 0.33113 1.01380 -2.39353
H   0.11639 -3.97892 2.20603
H -1.33386 -4.40113 0.64169
H -3.06241 -4.62453 -1.15172
H -3.71604 -2.56586 -2.48509
H -2.59611 -0.37883 -1.93519
H   1.84987 -3.32904 3.86889
H   2.70254 -0.97185 3.92679
H   1.83492 0.72334 2.33407
H   1.13953 4.68189 2.68082
H   2.41873 4.60569 1.43090
H   0.86480 5.42025 1.07252
O -1.98110 0.84861 1.54132
C -2.81792 1.57534 0.84701
O -2.54054 1.88689 -0.34974
C -4.08525 2.03574 1.53335
H -4.27275 1.46236 2.45272
H -3.99035 3.10351 1.79588
H -4.94068 1.93622 0.84728
C   1.75061 0.05008 -1.23221
C   2.83724 0.83383 -0.79639
C   1.88859 -1.33425 -1.46134
C   4.05625 0.19824 -0.51419
C   3.12001 -1.94560 -1.17717
C   4.20714 -1.18809 -0.70554
H   2.73062 1.91400 -0.67313
H   1.05209 -1.92141 -1.84784
H   4.89831 0.80100 -0.15708
H   3.22715 -3.02325 -1.34124
H   5.16769 -1.67073 -0.50173

46
TS(14b-16c)
SCF(BP86)= -1180.14169034
G(298 K) = -1179.856643
SCF(PBE) = -3739.58320491
Low Freq. = -159.7519cm-1,
13.6232cm-1
C -1.81567 -0.91127 -0.02925
C -2.76625 0.15059 -0.20695
C -4.12155 -0.12629 -0.49582
C -4.55948 -1.45086 -0.62138
C -3.63863 -2.50509 -0.46113
C -2.28785 -2.23812 -0.17610
C -2.22933 1.50268 -0.03803
N -0.87563 1.52635 0.23640
C -0.27663 2.71456 0.51324
C -0.96364 3.93081 0.48206
C -2.33119 3.92626 0.15567
C -2.96284 2.70489 -0.09792
Ru 0.04335 -0.32358 0.37902
N   0.76759 -2.15008 0.67176
C   1.17349 -3.22916 0.91189
C   1.67090 -4.56814 1.20085
Br 0.32334 -0.67666 -2.25412
H -4.83908 0.69204 -0.62489
H -4.03070 2.67383 -0.32928
H -2.89886 4.86055 0.11315
H -0.43099 4.85771 0.70921
H   0.78480 2.65181 0.76354
H -5.60966 -1.66241 -0.84638
H -3.97571 -3.54326 -0.56442
H -1.59135 -3.07544 -0.06329
H   0.90368 -5.32418 0.96411
H   2.57176 -4.78120 0.60170
H   1.93215 -4.65525 2.26857
O -0.27443 -0.16251 2.42700
C   0.85523 0.35167 2.83655
O   1.77004 0.61920 2.00875
C   1.01426 0.58049 4.32706
H   0.05298 0.49329 4.85369
H   1.71325 -0.16815 4.73778
H   1.45080 1.57519 4.50835
C   1.85675 0.29304 -1.30203
C   2.99105 -0.47776 -0.96570
C   1.96493 1.67751 -1.55280
C   4.21386 0.17851 -0.75500
C   3.19864 2.31028 -1.33221
C   4.32510 1.56990 -0.92934
H   2.91216 -1.56067 -0.85159

H   1.10142 2.24717 -1.90369
H   5.08914 -0.41362 -0.46696
H   3.27786 3.38969 -1.50009
H   5.28664 2.06925 -0.77715

46
TS(14c-16d)
SCF(BP86)= -1180.12370163
G(298 K) = -1179.839084
SCF(PBE) = -3739.56660678
Low Freq. = -162.4831cm-1,
7.8156cm-1
C -0.34134 1.53881 -0.37697
C -1.70552 1.95212 -0.57327
C -2.03518 3.24471 -1.03982
C -1.02768 4.17471 -1.31124
C   0.31187 3.80205 -1.10078
C   0.64797 2.51503 -0.64135
C -2.70967 0.95705 -0.22173
N -2.15163 -0.22291 0.20622
C -2.94417 -1.23522 0.64521
C -4.33602 -1.12969 0.65457
C -4.93052 0.05825 0.19093
C -4.11176 1.10324 -0.24425
Ru -0.12765 -0.40386 0.20284
N -0.10212 0.11488 2.10932
C -0.13662 0.40154 3.25047
C -0.16856 0.75204 4.66467
Br 1.78338 -2.04251 0.66360
H -3.08490 3.52274 -1.18617
H -4.54942 2.04140 -0.59423
H -6.01878 0.16769 0.17727
H -4.93475 -1.97000 1.01393
H -2.42499 -2.14126 0.96183
H -1.28021 5.17603 -1.67373
H   1.11572 4.52137 -1.29668
H   1.70388 2.29225 -0.49568
H -0.44012 -0.12646 5.27342
H   0.81944 1.11617 4.99210
H -0.91178 1.54665 4.84273
O -0.17034 -1.00222 -1.80918
C -0.65590 -2.20906 -1.97224
O -1.05522 -2.93800 -1.03305
C -0.68262 -2.68706 -3.42326
H -1.15736 -1.93243 -4.07134
H   0.34931 -2.82819 -3.78828
H -1.22391 -3.64019 -3.50688
C   2.33757 -0.06175 -0.02894
C   2.73290 -0.01150 -1.38065
C   3.00166 0.69355 0.96047
C   3.73619 0.89603 -1.75643
C   4.00543 1.59125 0.56470
C   4.37377 1.69819 -0.79094
H   2.22627 -0.63713 -2.11976
H   2.72476 0.59630 2.01330
H   4.02331 0.97011 -2.81083
H   4.50522 2.20417 1.32225
H   5.16642 2.39081 -1.09006

46
TS(14e-16j)
SCF(BP86)= -1180.12324113
G(298 K) = -1179.838148
SCF(PBE) = -3739.56485616
Low Freq. = -178.4728cm-1,
18.3416cm-1
N -0.34575 0.86855 1.25138
C   0.42437 2.02160 1.27441
C   0.06997 3.08616 2.12731
C -1.05943 2.99330 2.94535
C -1.83540 1.82097 2.90076
C -1.44901 0.78921 2.04372
C   1.57689 1.97740 0.37455
C   1.72503 0.73743 -0.33163
C   2.82219 0.63566 -1.22223
C   3.71853 1.70324 -1.40966
C   3.54941 2.91357 -0.70575
C   2.47851 3.05026 0.18684
Ru 0.36722 -0.67164 0.13824
N -1.00036 -2.16859 0.76824
C -1.66020 -3.06148 1.15668
C -2.48530 -4.16244 1.63804
Br -0.33582 -0.88018 -2.42273
H   0.69194 3.98517 2.14445
H   2.34879 3.99240 0.73250
H   4.24962 3.74218 -0.85409
H   4.55752 1.59435 -2.10769
H   2.97689 -0.29723 -1.77763
H -1.33157 3.81939 3.60893
H -2.72904 1.70046 3.51851
H -2.01502 -0.14082 1.96659
H -2.47558 -4.19246 2.73994
H -3.52582 -4.03486 1.29733
H -2.10295 -5.12232 1.25398
O   1.62924 -1.31230 1.67654
C   2.18342 -2.23593 0.94627
O   1.77936 -2.34533 -0.26401
C   3.22765 -3.14264 1.53813
H   3.67573 -2.68691 2.43303
H   2.76258 -4.10105 1.82751
H   4.00820 -3.35794 0.79259
C -1.60540 0.31825 -1.27678
C -2.85947 -0.23895 -0.95192
C -1.45799 1.70801 -1.47644
C -3.95248 0.61955 -0.74955
C -2.56567 2.54499 -1.26921
C -3.81616 2.01081 -0.90780
H -2.97879 -1.32053 -0.85855
H -0.49261 2.12494 -1.77298
H -4.92242 0.18648 -0.48134
H -2.44604 3.62493 -1.40897
H -4.67793 2.66976 -0.76524

46
TS(14e-16o)
SCF(BP86)= -1180.12951889
G(298 K) = -1179.844325
SCF(PBE) = -3739.56900812
Low Freq. = -168.3333cm-1,
13.0689cm-1
N   1.81682 0.94542 -0.04852
C   2.80847 -0.01724 -0.16374

C   4.13136 0.37161 -0.45797
C   4.45060 1.71932 -0.64418
C   3.42952 2.68089 -0.54061
C   2.13255 2.25470 -0.25018
C   2.33546 -1.38189 0.06114
C   0.93125 -1.48514 0.34230
C   0.44054 -2.78240 0.63705
C   1.27809 -3.91119 0.62046
C   2.64831 -3.78512 0.31075
C   3.17741 -2.51884 0.03566
Ru -0.04822 0.27793 0.41231
N -0.92847 2.19294 0.64255
C -1.38864 3.25222 0.86736
C -1.96010 4.56539 1.13882
Br -0.26895 0.51584 -2.24791
H   4.90638 -0.39474 -0.54104
H   4.24570 -2.41895 -0.18956
H   3.29626 -4.66754 0.29359
H   0.86313 -4.90008 0.85084
H -0.62064 -2.90717 0.88019
H   5.47830 2.01754 -0.87146
H   3.62576 3.74614 -0.68576
H   1.30182 2.95716 -0.16157
H -1.19548 5.34971 1.01433
H -2.79264 4.76934 0.44566
H -2.34321 4.60585 2.17160
O   0.22849 0.23473 2.48284
C -0.93177 -0.29182 2.74123
O -1.68163 -0.55666 1.74375
C -1.35229 -0.53064 4.16750
H -0.47974 -0.53513 4.83669
H -2.03863 0.27192 4.48873
H -1.89271 -1.48633 4.24724
C -1.83036 -0.43765 -1.24588
C -2.99066 0.34079 -1.04926
C -1.91111 -1.83230 -1.42843
C -4.22661 -0.31107 -0.91326
C -3.15893 -2.46108 -1.28697
C -4.31983 -1.71041 -1.02670
H -2.92392 1.42853 -0.98195
H -1.01483 -2.41438 -1.65188
H -5.12489 0.29005 -0.73437
H -3.21866 -3.54907 -1.40109
H -5.29019 -2.20808 -0.93638

46
2a OAc radical
SCF(BP86)= -1200.48550273
G(298 K) = -1200.198114
SCF(PBE) = -1199.54529213
Low Freq. = 27.1752cm-1,
33.5960cm-1
C   3.63213 1.46178 -0.30114
C   2.45345 0.69421 -0.20402
C   1.17580 1.32856 -0.05125
C   1.12692 2.74290 -0.05698
C   2.30520 3.50091 -0.13886
C   3.55599 2.86145 -0.26263
C   2.42159 -0.77043 -0.25961
N   1.15994 -1.30516 -0.12610
C   0.98419 -2.64802 -0.13680
C   2.05567 -3.53187 -0.29484
C   3.35035 -3.00793 -0.44389
C   3.53131 -1.62148 -0.42596
Ru -0.39042 0.06096 0.15429
O -1.73472 -1.58208 0.11724
C -2.95248 -1.60249 -0.41396
C -3.74958 -2.84222 -0.00358
N -0.63862 0.28437 -1.83590
C -0.76438 0.40528 -2.99100
C -0.94778 0.55041 -4.42563
N -0.12915 -0.17428 2.13466
C   0.01939 -0.31442 3.28692
C   0.20265 -0.48813 4.71747
N -1.82095 1.44827 0.47347
C -2.65564 2.24296 0.68153
C -3.69561 3.22853 0.93112
O -3.41884 -0.74051 -1.17396
H   4.52964 -1.19252 -0.53903
H -2.02222 0.52428 -4.66921
H -0.52526 1.51062 -4.76283
H -0.43783 -0.27063 -4.95467
H -3.68844 3.99245 0.13688
H -4.68262 2.73840 0.94790
H -3.52342 3.71981 1.90242
H   4.60881 0.97916 -0.41013
H   4.47074 3.45711 -0.33966
H   2.25348 4.59469 -0.11248
H   0.16316 3.25443 0.03070
H   4.20936 -3.67262 -0.57222
H   1.86854 -4.60822 -0.30407
H -0.05153 -2.97859 -0.02057
H -0.76753 -0.40523 5.23335
H   0.63927 -1.47880 4.92238
H   0.88190 0.29019 5.10175
H -3.23881 -3.75310 -0.35852
H -3.81044 -2.91194 1.09522
H -4.76341 -2.80328 -0.42669

46
2b OAc radical
SCF(BP86)= -1200.48734262
G(298 K) = -1200.200330
SCF(PBE) = -1199.54528927
Low Freq. = 20.4244cm-1,
22.6797cm-1
C -1.18586 -1.30720 -0.01858
C -2.46145 -0.67642 -0.21734
C -3.63457 -1.44881 -0.34165
C -3.56165 -2.84627 -0.25772
C -2.31957 -3.48031 -0.04791
C -1.14792 -2.71873 0.07596
C -2.43515 0.78638 -0.28203
N -1.17610 1.32795 -0.13385
C -1.01335 2.67292 -0.17725
C -2.08577 3.54874 -0.36360
C -3.37670 3.01477 -0.51296
C -3.54848 1.62838 -0.47142
Ru 0.36552 -0.04124 0.09031
N   0.13588 0.01523 2.11260

```
C   0.01707  0.06880  3.27599
C  -0.13080  0.13065  4.72019
N   1.77834 -1.47967  0.28410
C   2.59496 -2.30472  0.43546
C   3.61844 -3.32566  0.60005
N   1.95403  1.45082  0.37551
C   2.81650  2.21538  0.58014
C   3.89131  3.16604  0.81892
H  -4.60633 -0.97068 -0.50181
H   0.74120  0.63773  5.16388
H  -1.04595  0.68439  4.98420
H  -0.19599 -0.89055  5.12958
H   3.51465  4.02368  1.39885
H   4.70541  2.67986  1.37984
H   4.28594  3.52873 -0.14385
H  -4.54300  1.19159 -0.58770
H  -4.23804  3.67207 -0.66142
H  -1.90327  4.62546 -0.39136
H   0.01032  3.03344 -0.05819
H  -4.47337 -3.44407 -0.35227
H  -2.27046 -4.57243  0.01819
H  -0.19273 -3.22657  0.23974
H   4.16174 -3.16599  1.54545
H   3.15798 -4.32662  0.61749
H   4.33517 -3.27523 -0.23578
O   0.50376  0.08885 -1.91339
C   1.61947 -0.08275 -2.63714
O   2.76328 -0.18266 -2.18257
C   1.31300 -0.11804 -4.13181
H   0.80251  0.80991 -4.43787
H   2.24232 -0.23489 -4.70631
H   0.63091 -0.95431 -4.35871

40
3a OAc radical
SCF(BP86)= -1067.72270762
G(298 K) = -1067.476200
SCF(PBE) = -1066.88716000
Low Freq. = 31.9156cm-1,
33.5582cm-1
C  -3.70282 -0.11483 -0.24634
C  -2.32218  0.15622 -0.24287
N  -1.42748 -0.80897  0.16442
C  -1.87688 -2.02114  0.57058
C  -3.23898 -2.33610  0.59060
C  -4.16566 -1.36635  0.17365
C  -1.67828  1.40979 -0.64032
C  -2.36656  2.54977 -1.09664
C  -1.64686  3.69441 -1.47279
C  -0.23742  3.70854 -1.40445
C   0.46117  2.57691 -0.96154
C  -0.24198  1.41931 -0.54290
Ru  0.56339 -0.25811  0.13001
O   1.15342 -2.42744 -0.18393
C   1.08769 -2.22183 -1.44787
C   1.42717 -3.28719 -2.45274
O   0.73923 -1.03588 -1.83930
N   0.44990  0.16811  2.07440
C   0.39774  0.37797  3.22692
C   0.33729  0.64350  4.65279
N   2.50445  0.28914  0.04618
C   3.64595  0.55193  0.00441
C   5.05973  0.88753 -0.05337
H  -4.40576  0.65367 -0.57622
H   1.22726  0.22556  5.15068
H   0.31137  1.73257  4.82296
H  -0.56956  0.19059  5.08468
H   5.25221  1.80490  0.52624
H   5.66050  0.06514  0.36757
H   5.36320  1.05432 -1.09957
H  -3.45896  2.55352 -1.16625
H  -2.18465  4.57782 -1.82991
H   0.31293  4.60697 -1.70232
H   1.55385  2.59604 -0.91746
H  -5.23731 -1.58392  0.17514
H  -3.55729 -3.32637  0.92438
H  -1.10744 -2.73755  0.86754
H   0.94717 -3.07762 -3.41922
H   2.52059 -3.30620 -2.60253
H   1.11875 -4.27433 -2.07822

40
3c OAc radical
SCF(BP86)= -1067.71386259
G(298 K) = -1067.467459
SCF(PBE) = -1066.87698223
Low Freq. = 25.4330cm-1,
28.3532cm-1
C  -2.63720  2.25914 -1.26934
C  -1.85969  1.20698 -0.74802
N  -0.48869  1.34831 -0.63528
C   0.09926  2.51612 -1.00202
C  -0.63300  3.58550 -1.52091
C  -2.02578  3.45303 -1.65978
C  -2.35388 -0.08566 -0.27879
C  -1.33247 -0.99128  0.17243
C  -1.72052 -2.27964  0.61683
C  -3.07378 -2.64423  0.64255
C  -4.06589 -1.74264  0.19769
C  -3.71048 -0.46835 -0.26592
Ru  0.52443 -0.28535  0.09959
O   0.87122 -1.47015 -1.59221
C   1.43863 -2.40052 -0.88045
C   2.03318 -3.61729 -1.52119
N   0.39204  0.57093  1.90532
C   0.33183  1.04717  2.97445
C   0.26525  1.62929  4.30355
N   2.55534  0.50314 -0.13370
C   3.66067  0.86772 -0.26561
C   5.03060  1.32450 -0.43121
O   1.47679 -2.19455  0.39016
H  -4.49128  0.21637 -0.61219
H   1.07605  1.22979  4.93382
H   0.36322  2.72510  4.23723
H  -0.70520  1.38608  4.76614
H   5.21524  2.19659  0.21628
H   5.73102  0.51789 -0.16201
H   5.19993  1.61387 -1.48093
H  -3.71812  2.13290 -1.36537
H  -2.62528  4.27252 -2.06551
H  -0.11285  4.50185 -1.80920
H   1.18243  2.56564 -0.87476
```

H -5.11886 -2.03994 0.21140
H -3.36571 -3.63496 1.00666
H -0.95842 -2.98656 0.96054
H 1.39436 -3.96265 -2.34799
H 3.02103 -3.36100 -1.94171
H 2.16250 -4.41641 -0.77799

45
1a radical
SCF(BP86)= -1104.59378
G(298 K) = -1104.308660
SCF(PBE) = -1103.69435243
Low Freq. = 24.2597cm-1,
34.7592cm-1
N 1.05486 -1.32346 -0.00003
C 2.32944 -0.79513 -0.00006
C 3.44973 -1.64823 -0.00009
C 3.26856 -3.03422 -0.00008
C 1.96362 -3.55298 -0.00004
C 0.88223 -2.66612 -0.00002
C 2.36131 0.66630 -0.00007
C 3.54106 1.43175 -0.00011
C 3.45802 2.83225 -0.00011
C 2.19922 3.48155 -0.00007
C 1.02032 2.73318 -0.00003
C 1.06432 1.31179 -0.00003
H 4.52472 0.95296 -0.00014
Ru -0.47926 0.07029 0.00001
N -1.91885 1.51256 0.00005
C -2.78136 2.30294 0.00007
C -3.85276 3.28564 0.00010
N -0.50364 0.01587 -2.01605
C -0.53357 -0.04207 -3.18334
C -0.57034 -0.11023 -4.63245
N -2.12105 -1.38864 0.00006
C -3.03631 -2.11832 0.00009
C -4.16879 -3.02897 0.00012
N -0.50353 0.01588 2.01607
C -0.53339 -0.04206 3.18336
C -0.57009 -0.11021 4.63248
H -1.61039 -0.23068 4.97486
H 0.03149 -0.96425 4.98264
H -0.15678 0.82002 5.05534
H -4.13207 -3.66750 0.89727
H -5.11049 -2.45742 0.00013
H -4.13210 -3.66753 -0.89701
H 4.45512 -1.22191 -0.00011
H 4.13386 -3.70234 -0.00010
H 1.77415 -4.62866 -0.00003
H -0.15124 -3.01727 0.00001
H 4.37588 3.42742 -0.00014
H 2.15514 4.57512 -0.00007
H 0.05389 3.24435 -0.00001
H -4.47887 3.15465 0.89726
H -3.43137 4.30367 0.00011
H -4.47889 3.15468 -0.89706
H -1.61065 -0.23075 -4.97476
H -0.15711 0.82002 -5.05533
H 0.03126 -0.96423 -4.98264

55
4a radical
SCF(BP86)= -1414.30469350
G(298 K) = -1413.942085
SCF(PBE) = -1413.17792639
Low Freq. = 24.6524cm-1,
29.7047cm-1
C 4.30349 -1.29923 -0.23546
C 3.08381 -0.59733 -0.28134
N 1.97740 -1.08957 0.37142
C 2.04870 -2.27366 1.02758
C 3.23138 -3.01544 1.08937
C 4.38005 -2.51298 0.45443
C 2.81443 0.64234 -1.01008
C 3.76579 1.32159 -1.79830
C 3.39297 2.47039 -2.50941
C 2.06804 2.94316 -2.44094
C 1.11839 2.29144 -1.63673
C 1.47023 1.13241 -0.90638
Ru 0.26644 0.08474 0.29513
N -0.32706 -1.05758 -1.22888
C -0.56334 -1.71967 -2.16750
C -0.87456 -2.53079 -3.33203
H -0.23522 -2.22978 -4.17811
H -0.69015 -3.59427 -3.10878
H -1.93237 -2.39993 -3.61081
H 4.79467 0.95408 -1.86817
H 4.13213 2.99479 -3.12240
H 1.77302 3.82753 -3.01560
H 0.09569 2.67721 -1.60716
H 5.18200 -0.89475 -0.74359
H 5.32357 -3.06456 0.49373
H 3.24486 -3.96324 1.63240
H 1.13001 -2.60033 1.51939
N -1.44482 1.37256 0.32514
C -1.19580 2.68771 0.59979
H -0.16308 2.92300 0.86093
C -2.17109 3.68340 0.54832
H -1.89485 4.71608 0.77440
C -3.48091 3.32750 0.19569
C -3.75835 1.97980 -0.04667
H -4.27335 4.07868 0.13396
H -4.77517 1.64915 -0.27226
C -2.74062 1.00693 0.04170
C -3.09999 -0.42465 -0.12626
C -3.90486 -0.82573 -1.21685
C -4.28860 -2.16745 -1.35748
H -4.90096 -2.46893 -2.21320
C -3.88992 -3.12115 -0.40300
H -4.19644 -4.16632 -0.51154
C -3.10951 -2.72505 0.69549
H -2.81387 -3.45826 1.45273
C -2.71449 -1.38522 0.83682
H -2.12663 -1.07667 1.70649
O -0.06810 -0.60729 2.46427
C 0.55117 0.44559 2.85567
H -4.20874 -0.08814 -1.96691
O 0.98452 1.24862 1.93970
C 0.80053 0.73036 4.31087
H 1.68354 0.15810 4.64409
H 0.99621 1.79997 4.47142
H -0.05992 0.40411 4.91383

61
5a radical
SCF(BP86)= -1547.05969172
G(298 K) = -1546.656479
SCF(PBE) = -1545.83140778
Low Freq. = 15.4917cm-1,
29.9969cm-1
C -3.57415 -0.52597 0.84313
C -2.59452 0.48236 0.98358
C -2.76273 1.71114 0.31000
C -3.88216 1.91209 -0.51328
C -4.84088 0.89691 -0.67033
C -4.68409 -0.32333 0.00956
C -1.51444 0.32088 1.99976
C -1.93942 0.15730 3.33715
C -1.02242 0.10543 4.38819
C   0.33622 0.23768 4.07569
C   0.70618 0.36572 2.73820
N -0.17597 0.38855 1.69444
Ru 0.79261 0.23908 -0.25367
N   1.81045 -0.02680 -1.98086
H -3.01396 0.10421 3.52824
H -1.36047 -0.01577 5.42121
H   1.11233 0.22977 4.84471
H   1.75846 0.43515 2.47091
H -3.45849 -1.47154 1.38333
H -5.43077 -1.11594 -0.10168
H -5.71341 1.05957 -1.31134
H -4.01147 2.87212 -1.02382
C   2.39553 -0.19658 -2.97955
C   3.12450 -0.40351 -4.21959
H   2.79582 -1.33973 -4.69837
H   2.93599 0.43751 -4.90630
H   4.20531 -0.46185 -4.01316
N -0.65827 -0.98567 -1.08353
C -1.59146 -0.54911 -1.96233
H -1.60401 0.53049 -2.12926
C -2.48148 -1.42372 -2.59133
H -3.21989 -1.02819 -3.29222
C -2.40870 -2.79468 -2.29412
C -1.43238 -3.24785 -1.40155
H -3.09826 -3.50378 -2.76105
H -1.34130 -4.31297 -1.17596
C -0.53961 -2.33068 -0.81375
C   0.60801 -2.66517 0.03705
C   1.45509 -1.55927 0.39144
C   2.62898 -1.83995 1.13200
H   3.31379 -1.02622 1.39537
C   2.94276 -3.14883 1.52831
H   3.85852 -3.34436 2.09653
C   2.07977 -4.21501 1.20088
H   2.31905 -5.23315 1.52311
C   0.91744 -3.97654 0.45395
H   0.26269 -4.81585 0.19730
O -0.07263 1.96144 -1.13742
C   0.08072 3.23570 -0.81464
O   0.02035 3.67112 0.35005
H -2.02764 2.50667 0.45931
C   0.28904 4.15774 -2.01323
H   1.23335 3.90054 -2.52293
H   0.32373 5.20873 -1.69224
H -0.52262 4.02017 -2.74654
N   2.32185 1.37269 0.42225
C   3.22838 2.05910 0.70944
C   4.34729 2.91383 1.07569
H   4.96633 3.12171 0.18782
H   3.97449 3.86817 1.48182
H   4.97099 2.41884 1.83760

60
2a 2-ppy-H radical
SCF(BP86)= -1451.16715967
G(298 K) = -1450.766473
SCF(PBE) = -1449.97930831
Low Freq. = 13.4135cm-1,
20.0759cm-1
C   2.15890 -1.04574 -0.05217
C   3.00132 -0.34517 -0.99119
C   4.24381 -0.88429 -1.37484
C   4.66662 -2.10681 -0.83424
C   3.85757 -2.79891 0.09781
C   2.62793 -2.26745 0.49710
C   2.46568 0.91895 -1.49177
N   1.21881 1.23586 -0.99174
C   0.58666 2.34745 -1.43902
C   1.16879 3.21609 -2.36731
C   2.45104 2.92581 -2.85937
C   3.09804 1.76770 -2.42073
Ru 0.43416 -0.14161 0.36556
N -0.42175 -1.02947 -1.22113
C -0.77210 -1.57921 -2.19253
C -1.18335 -2.26632 -3.40269
N -0.21324 -1.55562 1.66626
C -0.52689 -2.36307 2.45374
C -0.92524 -3.36945 3.42417
N   1.43406 0.67793 1.92876
C   2.08257 1.05412 2.82707
C   2.88155 1.52702 3.94231
H   4.88804 -0.36195 -2.08809
H -1.79469 -3.14591 -3.14743
H -1.77758 -1.58483 -4.03225
H -0.29040 -2.58846 -3.96326
H   4.08962 1.51160 -2.79974
H   2.93555 3.58884 -3.58104
H   0.61710 4.10211 -2.68918
H -0.40854 2.53310 -1.03456
H   5.63296 -2.52429 -1.13197
H   4.20024 -3.75350 0.50940
H   2.01626 -2.80920 1.22273
H -1.93658 -3.73850 3.18912
H -0.21910 -4.21521 3.40009
H -0.92834 -2.93234 4.43564
H   2.24873 1.63335 4.83803
H   3.68466 0.80155 4.15159
H   3.33196 2.50157 3.69443
N -1.39949 1.30752 0.90226
C -1.03390 2.44086 1.56489
C -1.86133 3.55248 1.73901
C -3.14863 3.52239 1.18752
C -3.55207 2.36081 0.52380
C -2.67852 1.25724 0.40785

H -0.01647 2.45955 1.95527
H -1.48685 4.42119 2.28593
H -3.82744 4.37444 1.28462
H -4.55975 2.27254 0.10991
C -3.21089 0.02131 -0.23107
C -3.76971 0.08516 -1.52696
C -4.35239 -1.05366 -2.10391
C -4.40137 -2.26238 -1.38645
C -3.86884 -2.32566 -0.08760
C -3.27805 -1.19043 0.48847
H -3.73527 1.02650 -2.08537
H -4.77433 -0.99512 -3.11218
H -4.86533 -3.14723 -1.83346
H -3.92627 -3.25686 0.48478
H -2.89308 -1.22859 1.51025

40
2b PhBr-Ph
SCF(BP86)= -985.435670105
G(298 K) = -985.194626
SCF(PBE) = -3545.00633527
Low Freq. = 21.0054cm-1,
24.2772cm-1
N -0.88827 1.50537 -0.25062
C -2.22659 1.18423 -0.22978
C -3.19422 2.19587 -0.39101
C -2.79787 3.52480 -0.55646
C -1.42739 3.83385 -0.55757
C -0.50446 2.79844 -0.39925
C -2.49206 -0.23855 -0.02190
C -3.78897 -0.79820 0.01246
C -3.95096 -2.17803 0.17639
C -2.81583 -3.00236 0.29639
C -1.52203 -2.45568 0.25289
C -1.33320 -1.06236 0.11580
H -4.67166 -0.15939 -0.09408
Ru 0.45753 -0.09229 0.03920
N 0.50767 -0.53946 -1.91978
C 0.53922 -0.79917 -3.05983
C 0.58512 -1.12503 -4.47647
N 2.20179 1.19170 -0.25571
C 3.18384 1.80378 -0.43403
C 4.40544 2.55993 -0.65237
N 0.41082 0.33754 1.99987
C 0.38079 0.58140 3.14366
C 0.34804 0.88237 4.56594
H 1.37085 0.86871 4.97560
H -0.09215 1.87911 4.72958
H -0.26013 0.13005 5.09369
H 4.51729 3.32881 0.12854
H 5.27194 1.88008 -0.61775
H 4.37060 3.04717 -1.63999
H -4.25406 1.93188 -0.38215
H -3.54707 4.31167 -0.68053
H -1.06813 4.85780 -0.68240
H 0.56991 2.98592 -0.39728
H -4.95492 -2.61182 0.20653
H -2.93712 -4.08309 0.42913
H -0.65414 -3.11386 0.35366
H 1.62848 -1.29187 -4.78909
H 0.00180 -2.03977 -4.66935
H 0.16067 -0.29742 -5.06724
Br 1.95598 -2.03603 0.50436

40
2a PhBr-Ph
SCF(BP86)= -985.440742838
G(298 K) = -985.199333
SCF(PBE) = -3545.01071516
Low Freq. = 4.8217cm-1, 21.7888cm-
1
N -1.28872 -1.13172 -0.03090
C -2.46813 -0.41797 -0.02376
C -3.70465 -1.09094 -0.04582
C -3.73742 -2.48852 -0.07328
C -2.52655 -3.19843 -0.07831
C -1.32353 -2.48517 -0.05626
C -2.28513 1.03530 0.00845
C -3.34500 1.96278 0.02400
C -3.06714 3.33754 0.05056
C -1.73187 3.79783 0.06044
C -0.67113 2.88393 0.04358
C -0.91837 1.48798 0.02200
H -4.38658 1.62673 0.01523
Ru 0.45000 0.02534 0.00874
N 0.42481 -0.04457 2.02387
C 0.40266 -0.08698 3.19238
C 0.37984 -0.14630 4.64328
N 2.06781 1.23667 0.05305
C 3.02247 1.91411 0.07766
C 4.20395 2.76279 0.10766
N 0.46422 0.07377 -2.00913
C 0.46543 0.10077 -3.17815
C 0.47225 0.12883 -4.63025
H 1.50830 0.18926 -5.00011
H -0.00096 -0.78507 -5.02413
H -0.09011 1.00685 -4.98721
H 4.81763 2.58279 -0.78971
H 3.90805 3.82403 0.13316
H 4.80603 2.53637 1.00240
H -4.63388 -0.51685 -0.04142
H -4.69488 -3.01655 -0.09053
H -2.50222 -4.29045 -0.09956
H -0.34787 -2.97945 -0.05947
H -3.89232 4.05601 0.06234
H -1.52798 4.87337 0.08087
H 0.35896 3.25213 0.05106
H 1.40857 -0.21058 5.03252
H -0.10068 0.76105 5.04404
H -0.18962 -1.03011 4.97304
Br 2.14504 -2.02625 -0.05173

40
2c PhBr-Ph
SCF(BP86)= -985.438985827
G(298 K) = -985.197933
SCF(PBE) = -3545.00887746
Low Freq. = 25.6412cm-1,
26.8335cm-1
N 1.06736 1.32949 0.01354
C 2.34876 0.82110 0.02334

C 3.45586 1.69182 0.00920
C 3.25576 3.07470 -0.01949
C 1.94289 3.57470 -0.03421
C 0.87718 2.67106 -0.01751
C 2.40200 -0.64126 0.04528
C 3.59710 -1.38805 0.05631
C 3.54265 -2.78884 0.08093
C 2.29699 -3.45290 0.09716
C 1.10357 -2.71843 0.08735
C 1.12412 -1.30229 0.04580
H 4.57108 -0.88848 0.04770
Ru -0.45295 -0.07728 0.02095
N -0.43961 -0.07644 2.05338
C -0.43859 -0.05441 3.22377
C -0.43532 -0.03119 4.67629
N -1.86946 -1.53035 -0.00825
C -2.71259 -2.34241 -0.03674
C -3.75647 -3.35508 -0.07839
N -2.11525 1.36751 0.18490
C -3.03220 2.08683 0.29642
C -4.16929 2.98453 0.42548
H -4.69445 2.78825 1.37401
H -3.82629 4.03136 0.41216
H -4.86749 2.82279 -0.41129
H -4.37863 -3.21487 -0.97702
H -3.30723 -4.36091 -0.10688
H -4.39597 -3.27336 0.81525
H 4.46750 1.27973 0.01765
H 4.11168 3.75517 -0.03240
H 1.73756 4.64736 -0.05932
H -0.16188 3.00577 -0.02882
H 4.47127 -3.36754 0.09091
H 2.26497 -4.54730 0.11805
H 0.14586 -3.24708 0.10180
H -1.46964 -0.01241 5.05543
H 0.07084 -0.93290 5.05786
H 0.10129 0.86099 5.03707
Br -0.56647 0.09838 -2.46928

41
5a PhBr-Ph
SCF(BP86)= -1081.32668841
G(298 K) = -1081.083450
SCF(PBE) = -3640.85330305
Low Freq. = 28.7056cm-1,
31.6172cm-1
N 1.40892 -1.10447 -0.27193
C 2.55568 -0.34215 -0.22090
C 3.81136 -0.93124 -0.46328
C 3.89664 -2.29490 -0.76263
C 2.71776 -3.05563 -0.81634
C 1.49528 -2.42394 -0.56643
C 2.31558 1.06879 0.10114
C 3.33701 2.03394 0.22349
C 3.01258 3.35626 0.55921
C 1.66915 3.72278 0.77865
C 0.64691 2.76910 0.65514
C 0.94311 1.43365 0.29813
H 4.38578 1.76272 0.06324
Ru -0.37102 -0.06441 0.04509
N -0.25432 -0.34852 2.05616
C -0.18062 -0.49431 3.21579
C -0.09577 -0.68595 4.65511
N -2.03498 1.02544 0.31874
C -3.02522 1.61985 0.51734
C -4.25005 2.37616 0.73636
H -4.48779 2.97407 -0.15877
H -5.08997 1.69256 0.94067
H -4.12875 3.05633 1.59514
H 4.71469 -0.31806 -0.41969
H 4.86947 -2.75735 -0.95301
H 2.73456 -4.12347 -1.04766
H 0.54107 -2.95910 -0.58813
H 3.80763 4.10217 0.65630
H 1.42309 4.75629 1.04568
H -0.39242 3.06491 0.82597
H 0.84876 -1.18952 4.91675
H -0.13652 0.28973 5.16614
H -0.94044 -1.30408 5.00029
Br -1.97473 -2.28193 0.07566
O -0.43633 0.04533 -1.96396
C -0.95874 0.99557 -2.73990
O -1.23832 2.14678 -2.38206
C -1.14978 0.51311 -4.17694
H -1.89398 -0.30099 -4.20217
H -0.20680 0.10446 -4.57508
H -1.49331 1.34019 -4.81404

41
5b PhBr-Ph
SCF(BP86)= -1081.32613902
G(298 K) = -1081.082655
SCF(PBE) = -3640.85385552
Low Freq. = 21.7607cm-1,
26.8012cm-1
N -1.47878 -1.02960 -0.16039
C -2.57863 -0.20358 -0.24354
C -3.86293 -0.74828 -0.44391
C -4.02954 -2.13032 -0.55994
C -2.89906 -2.95860 -0.47392
C -1.64684 -2.37185 -0.27609
C -2.26973 1.22058 -0.10190
C -3.23694 2.25122 -0.14449
C -2.84699 3.58566 0.01658
C -1.48921 3.89344 0.22671
C -0.52131 2.87421 0.27217
C -0.89464 1.52514 0.10157
H -4.29447 2.01343 -0.30131
Ru 0.40274 -0.04955 0.12559
N 0.56838 0.05542 -1.86094
C 0.58777 0.09379 -3.03065
C 0.65711 0.14435 -4.48360
N 0.19791 -0.16829 2.11907
C 0.07458 -0.22805 3.28190
C -0.07483 -0.30769 4.72765
H -0.98883 -0.86840 4.98182
H 0.79390 -0.82230 5.16907
H -0.14627 0.70495 5.15641
H -4.72537 -0.08056 -0.50682
H -5.02539 -2.55556 -0.71486
H -2.97425 -4.04550 -0.55706
H -0.73247 -2.96610 -0.20136

H -3.59588 4.38287 -0.01797
H -1.17937 4.93659 0.35706
H   0.53031 3.12487 0.44165
H   0.40336 -0.83969 -4.91005
H -0.05129 0.89363 -4.87234
H   1.67643 0.41823 -4.80152
Br 1.75208 -2.35338 0.14953
O   1.99549 1.12714 0.53797
C   3.04063 1.42930 -0.24152
O   3.33168 0.87867 -1.30886
C   3.88617 2.54905 0.36281
H   4.20219 2.28280 1.38472
H   3.28921 3.47376 0.43826
H   4.77131 2.73629 -0.26104

41
5c PhBr-Ph
SCF(BP86)= -1081.32301580
G(298 K) = -1081.079660
SCF(PBE) = -3640.85225011
Low Freq. = 14.8620cm-1,
27.8127cm-1
N   0.87775 1.49986 0.20865
C   2.20715 1.29820 -0.08244
C   3.10128 2.38795 -0.07402
C   2.64368 3.66789 0.24851
C   1.28770 3.84627 0.56918
C   0.43645 2.73948 0.53868
C   2.54765 -0.09813 -0.35534
C   3.85440 -0.54006 -0.66520
C   4.09804 -1.89827 -0.89579
C   3.03252 -2.81431 -0.81635
C   1.72948 -2.37964 -0.51352
C   1.45594 -1.01368 -0.27231
H   4.68209 0.17483 -0.72510
Ru -0.38129 -0.19380 0.14947
N -0.66666 -0.01317 -1.81937
C -0.81295 0.05045 -2.97878
C -1.01495 0.15482 -4.41643
N -0.10404 -0.41652 2.11807
C   0.05521 -0.54282 3.27152
C   0.25072 -0.70397 4.70499
H   0.88516 0.10902 5.09352
H -0.72130 -0.67688 5.22362
H   0.73987 -1.66922 4.91362
H   4.15374 2.22129 -0.31507
H   3.33584 4.51490 0.25658
H   0.88443 4.82557 0.83780
H -0.62539 2.80966 0.77278
H   5.10907 -2.24223 -1.13507
H   3.21491 -3.88066 -0.99292
H   0.91751 -3.11125 -0.46051
H -0.04295 0.18457 -4.93489
H -1.58587 -0.71394 -4.78216
H -1.57438 1.07466 -4.65266
Br -1.73741 -2.33232 0.09606
O -2.04701 1.03024 0.65124
C -2.79883 1.76382 -0.15731
O -2.38598 2.38964 -1.14736
C -4.26877 1.79924 0.26846
H -4.35732 2.06461 1.33447
H -4.71518 0.79761 0.14466
H -4.82788 2.52202 -0.34324

41
5d PhBr-Ph
SCF(BP86)= -1081.31600009
G(298 K) = -1081.073299
SCF(PBE) = -3640.84233812
Low Freq. = 19.8363cm-1,
21.2929cm-1
N   1.01738 1.48480 0.17303
C   2.34668 1.12913 0.28061
C   3.32471 2.12690 0.47877
C   2.95015 3.46868 0.56714
C   1.58963 3.81054 0.45444
C   0.65727 2.79175 0.25777
C   2.58679 -0.30576 0.15997
C   3.87248 -0.88998 0.23154
C   4.01681 -2.27470 0.09627
C   2.87687 -3.07861 -0.11325
C   1.59846 -2.50251 -0.18442
C   1.41127 -1.10682 -0.04253
H   4.76042 -0.26855 0.39099
Ru -0.32453 -0.06718 -0.07660
N -0.16820 0.17709 -2.07962
C -0.07745 0.34363 -3.23584
C   0.03219 0.54049 -4.67236
N -2.11386 1.15829 -0.22978
C -3.07102 1.80201 -0.43516
C -4.27348 2.58986 -0.66780
H -5.02168 2.36953 0.11071
H -4.03621 3.66552 -0.64219
H -4.70112 2.34187 -1.65257
H   4.37579 1.84067 0.56358
H   3.70720 4.24280 0.72232
H   1.25088 4.84723 0.51913
H -0.41151 2.99559 0.16568
H   5.01103 -2.72947 0.15040
H   2.99058 -4.16345 -0.22151
H   0.72545 -3.14268 -0.34917
H   0.69223 1.39664 -4.88644
H   0.45979 -0.36250 -5.13798
H -0.96171 0.73164 -5.10804
Br -1.85131 -2.11411 -0.46351
O -0.34216 -0.16028 1.94790
C -1.39216 -0.19911 2.77124
O -2.57694 -0.02528 2.46250
C -0.95693 -0.48017 4.21109
H -0.20380 0.25661 4.53540
H -0.48596 -1.47567 4.27433
H -1.82430 -0.44449 4.88530

41
5e PhBr-Ph
SCF(BP86)= -1081.31742950
G(298 K) = -1081.074606
SCF(PBE) = -3640.84522319
Low Freq. = 28.9626cm-1,
32.8380cm-1
N   1.19741 1.30352 0.16986
C   2.46111 0.75318 0.23638

```
C   3.58552  1.59340  0.38191
C   3.42014  2.97706  0.46466
C   2.12229  3.51857  0.40594
C   1.04057  2.64894  0.26492
C   2.47539 -0.70479  0.14887
C   3.65200 -1.48904  0.18775
C   3.56505 -2.88061  0.07540
C   2.30298 -3.49254 -0.08112
C   1.13248 -2.71848 -0.12680
C   1.18637 -1.31079 -0.00735
H   4.63381 -1.01696  0.30364
Ru -0.37989 -0.04396 -0.04772
N  -0.52791  0.02348  1.94838
C  -0.56910  0.07341  3.11824
C  -0.65426  0.13544  4.56903
N  -1.99708  1.41492 -0.18558
C  -2.86321  2.19563 -0.30025
C  -3.94719  3.15715 -0.44331
H  -4.91432  2.66175 -0.25997
H  -3.82344  3.97916  0.27991
H  -3.94741  3.57415 -1.46320
H   4.58411  1.15230  0.42907
H   4.29062  3.62997  0.57663
H   1.94605  4.59507  0.46725
H   0.01146  3.01121  0.21270
H   4.47350 -3.49036  0.10733
H   2.23656 -4.58340 -0.16692
H   0.15754 -3.20193 -0.24854
H  -1.67182 -0.13006  4.89912
H   0.06564 -0.56502  5.02231
H  -0.42201  1.15609  4.91448
Br -0.16309  0.13391 -2.58398
O  -1.67308 -1.61954 -0.22075
C  -2.82981 -1.75609  0.42434
O  -3.27226 -0.99445  1.29637
C  -3.58722 -3.00717 -0.02466
H  -2.97851 -3.90758  0.16250
H  -3.78012 -2.96642 -1.10973
H  -4.54115 -3.09138  0.51494

41
5f PhBr-Ph
SCF(BP86)= -1081.32494830
G(298 K) = -1081.081808
SCF(PBE) = -3640.85156509
Low Freq. = 22.8465cm-1,
29.1052cm-1
N   1.18280  1.32311  0.11706
C   2.44843  0.79758  0.27370
C   3.55785  1.65426  0.42201
C   3.37961  3.04003  0.40944
C   2.08347  3.55932  0.24619
C   1.01631  2.66959  0.10455
C   2.48402 -0.66514  0.25600
C   3.66509 -1.42581  0.39656
C   3.60319 -2.82480  0.35879
C   2.36213 -3.46989  0.18064
C   1.18477 -2.71789  0.04129
C   1.21134 -1.30312  0.07480
H   4.63414 -0.93442  0.53548
Ru -0.35160 -0.05056 -0.05952
N  -1.74888 -1.48863 -0.24757
C  -2.55412 -2.32610 -0.40234
C  -3.56018 -3.36469 -0.57202
N  -1.97970  1.42648 -0.19241
C  -2.86775  2.18425 -0.28526
C  -3.97395  3.12405 -0.39709
H  -4.75546  2.87247  0.33772
H  -3.61997  4.15018 -0.20830
H  -4.40612  3.07499 -1.40943
H   4.55611  1.22733  0.54541
H   4.23820  3.70797  0.52383
H   1.89431  4.63534  0.22849
H  -0.01015  3.01905 -0.02463
H   4.51915 -3.41421  0.46709
H   2.31726 -4.56436  0.15163
H   0.23164 -3.23870 -0.09604
H  -4.16367 -3.16535 -1.47246
H  -3.07760 -4.34977 -0.67931
H  -4.22851 -3.38925  0.30432
Br -0.15301  0.08859 -2.60425
O  -0.42132  0.07250  1.98128
C  -1.50188 -0.11665  2.74050
O  -2.65192 -0.33587  2.34064
C  -1.15750 -0.02112  4.22889
H  -0.70459  0.95850  4.45435
H  -0.41538 -0.79158  4.49685
H  -2.06256 -0.15681  4.83760

35
15c
SCF(BP86)= -948.555553749
G(298 K) = -948.352378
SCF(PBE) = -3508.18783738
Low Freq. = 28.2390cm-1,
32.2229cm-1
C   3.71820  0.91259 -0.26121
C   2.41366  0.37349 -0.26413
C   1.30423  1.14799  0.19785
C   1.54235  2.46628  0.65176
C   2.84707  2.98625  0.67259
C   3.93245  2.21462  0.21008
C   2.07280 -0.97416 -0.71879
C   2.97516 -1.93195 -1.22128
C   2.52076 -3.20504 -1.57438
C   1.15830 -3.50860 -1.41442
C   0.29882 -2.52459 -0.92055
N   0.73149 -1.27875 -0.59571
Ru -0.49334  0.25301  0.12468
O  -1.59554  2.02982  0.48882
C  -1.56119  2.33169 -0.77035
C  -2.26437  3.53883 -1.31558
O  -0.91295  1.51357 -1.53156
N  -0.32299 -0.63583  1.89069
C  -0.22859 -1.13531  2.94807
C  -0.12732 -1.74885  4.26274
H   4.56783  0.32282 -0.62103
H   0.93117 -1.83666  4.55706
H  -0.65833 -1.13640  5.00951
H  -0.57495 -2.75599  4.24209
H   4.03097 -1.67152 -1.32779
H   3.21812 -3.95150 -1.96513
```

H   0.75553 -4.49137 -1.67118
H -0.77227 -2.69717 -0.78687
H   4.94428 2.63147 0.21623
H   3.02078 4.00151 1.04598
H   0.70783 3.07870 1.00872
H -1.80407 3.85682 -2.26202
H -3.32177 3.28723 -1.50874
H -2.23817 4.35891 -0.58267
Br -2.78587 -0.99240 -0.41307

35
15a
SCF(BP86)= -948.564469719
G(298 K) = -948.361743
SCF(PBE) = -3508.19737203
Low Freq. = 33.0118cm-1,
34.5822cm-1
C -3.75191 -0.19511 -0.19520
C -2.38304 0.13285 -0.21005
N -1.43511 -0.82955 0.06667
C -1.82705 -2.09530 0.35757
C -3.17355 -2.46911 0.38825
C -4.15253 -1.50110 0.10567
C -1.80265 1.44584 -0.49836
C -2.55469 2.59144 -0.82895
C -1.90171 3.80354 -1.09562
C -0.49461 3.87982 -1.03748
C   0.26396 2.74526 -0.71184
C -0.36684 1.50869 -0.42490
Ru 0.52381 -0.20941 0.00511
O   1.27899 -2.35286 -0.48108
C   1.10075 -2.06720 -1.71283
C   1.45024 -3.02749 -2.82126
O   0.62766 -0.89361 -2.00913
N   2.42599 0.42237 -0.09398
C   3.55386 0.74328 -0.14661
C   4.94992 1.15052 -0.21178
H -4.49548 0.57439 -0.41707
H   5.09223 2.10188 0.32648
H   5.59187 0.38184 0.24855
H   5.25557 1.28725 -1.26193
H -3.64759 2.54561 -0.88262
H -2.48836 4.69092 -1.35285
H   0.00804 4.83014 -1.24771
H   1.35519 2.81885 -0.67114
H -5.21462 -1.76177 0.12064
H -3.44104 -3.50081 0.62894
H -1.01998 -2.80430 0.55782
H   0.86424 -2.81143 -3.72627
H   2.52098 -2.92100 -3.06786
H   1.27842 -4.06372 -2.49462
Br 0.56122 -0.09165 2.49681

35
15b
SCF(BP86)= -948.554345157
G(298 K) = -948.351920
SCF(PBE) = -3508.18782840
Low Freq. = 26.6905cm-1,
27.4750cm-1
C   3.70934 0.91408 -0.11344
C   2.41573 0.35953 -0.19418
N   1.33208 1.08242 0.26030
C   1.50703 2.33026 0.76999
C   2.77000 2.91418 0.87332
C   3.88989 2.19154 0.42167
C   2.05551 -0.94923 -0.72998
C   2.98577 -1.86199 -1.27613
C   2.55147 -3.10971 -1.73578
C   1.18681 -3.45398 -1.64037
C   0.25680 -2.55830 -1.08994
C   0.65931 -1.27679 -0.64215
Ru -0.51522 0.17899 0.09998
O -1.64638 2.17199 0.38306
C -1.49574 2.34630 -0.87479
C -2.07447 3.53361 -1.59953
O -0.83798 1.44362 -1.54394
N -0.35928 -0.73425 1.86361
C -0.27465 -1.21269 2.93138
C -0.18798 -1.80797 4.25544
H   4.56587 0.33717 -0.47031
H   0.78756 -1.57416 4.71176
H -0.99075 -1.41665 4.90129
H -0.29135 -2.90288 4.17995
H   4.04721 -1.60013 -1.34254
H   3.26973 -3.81586 -2.16357
H   0.84705 -4.43224 -1.99889
H -0.79834 -2.84161 -1.02652
H   4.89251 2.62372 0.48621
H   2.86726 3.91558 1.29901
H   0.59645 2.84064 1.09359
H -1.51229 3.74178 -2.52143
H -3.12075 3.31339 -1.87423
H -2.07455 4.41703 -0.94409
Br -2.77370 -0.91418 -0.29837

35
15d
SCF(BP86)= -948.556963335
G(298 K) = -948.354484
SCF(PBE) = -3508.19077356
Low Freq. = 23.7289cm-1,
28.9485cm-1
C -3.21762 -1.09378 1.48510
C -2.15287 -0.53005 0.75042
C -0.79045 -0.82532 1.08744
C -0.53947 -1.69053 2.17858
C -1.60343 -2.24114 2.90966
C -2.94003 -1.94522 2.56285
C -2.29890 0.36488 -0.39284
C -3.50447 0.82200 -0.96218
C -3.47249 1.68585 -2.05961
C -2.22854 2.08968 -2.57942
C -1.06178 1.61160 -1.97960
N -1.08736 0.76815 -0.91732
Ru 0.61852 0.04172 -0.01051
O   2.44234 -0.60559 0.85872
C   3.21198 0.11760 0.09960
C   4.70587 0.08470 0.32441
O   2.67586 0.85123 -0.79548
N   0.63450 1.69815 1.13020
C   0.64548 2.67001 1.78622

C 0.66489 3.87756 2.59497
H -4.25805 -0.87317 1.22267
H -0.28775 3.98577 3.13813
H 1.49311 3.83251 3.32066
H 0.80790 4.75604 1.94452
H -4.45801 0.49420 -0.54028
H -4.40427 2.04260 -2.50775
H -2.15736 2.76304 -3.43686
H -0.06766 1.88913 -2.34092
H -3.76316 -2.38352 3.13573
H -1.39746 -2.90866 3.75370
H 0.49433 -1.92993 2.45271
H 5.23698 0.50221 -0.54241
H 4.95521 0.68586 1.21592
H 5.04288 -0.94621 0.51354
Br 0.72182 -1.79891 -1.70561

35
15e
SCF(BP86)= -948.553420082
G(298 K) = -948.350812
SCF(PBE) = -3508.18547981
Low Freq. = 26.4073cm-1,
27.5197cm-1
C -3.75868 -0.40071 -0.34247
C -2.39691 -0.02331 -0.31494
C -1.37570 -0.96418 0.04316
C -1.77371 -2.28518 0.35978
C -3.12830 -2.65218 0.33900
C -4.12093 -1.71203 -0.01436
C -1.89930 1.30842 -0.63652
C -2.66931 2.41935 -1.03620
C -2.05205 3.64226 -1.30521
C -0.65464 3.74625 -1.16859
C 0.07042 2.62331 -0.77276
N -0.52251 1.42339 -0.51874
Ru 0.49309 -0.25788 0.00996
O 1.46441 -2.20995 0.06269
C 1.35883 -2.29268 -1.21406
C 1.90873 -3.45009 -1.99736
O 0.76292 -1.30586 -1.81618
N 2.51944 0.52576 -0.21460
C 3.62637 0.88436 -0.35790
C 4.99894 1.33499 -0.53181
H -4.53482 0.32236 -0.61610
H 5.19263 2.20621 0.11437
H 5.69849 0.52656 -0.26534
H 5.16704 1.62380 -1.58187
H -3.75277 2.31261 -1.13193
H -2.64760 4.50591 -1.61427
H -0.12832 4.68419 -1.36147
H 1.15475 2.65058 -0.64845
H -5.17472 -2.00747 -0.03175
H -3.41974 -3.67636 0.59805
H -1.01587 -3.02760 0.63364
H 1.18643 -3.76684 -2.76556
H 2.83198 -3.13713 -2.51469
H 2.14242 -4.28983 -1.32812
Br 0.42870 0.39516 2.42679

59
TS(10a-16^{bis}a)
SCF(BP86)= -1430.37344850
G(298 K) = -1429.987192
SCF(PBE) = -3989.57578363
Low Freq. = -229.4385cm-1,
23.9227cm-1
C 3.60252 1.45746 1.94175
C 2.65091 0.69768 1.22158
C 1.52350 1.32508 0.60520
C 1.41772 2.73017 0.71645
C 2.37178 3.48244 1.42359
C 3.46275 2.84640 2.04767
C 2.77139 -0.74437 0.99448
C 3.81300 -1.56186 1.47920
C 3.88425 -2.90903 1.11357
C 2.91312 -3.41670 0.23435
C 1.89261 -2.56823 -0.20235
N 1.77815 -1.26972 0.18675
Ru 0.27306 0.08144 -0.40099
H 4.57288 -1.13001 2.13568
H 4.69062 -3.54517 1.49025
H 2.93773 -4.45239 -0.11451
H 1.13268 -2.91513 -0.90677
H 4.20140 3.43175 2.60470
H 2.26234 4.57120 1.49251
H 0.57270 3.24974 0.25235
N -1.14510 1.53742 -0.80704
C -1.35671 2.08906 -2.03391
H -0.73493 1.69180 -2.83931
C -2.29383 3.09819 -2.26445
H -2.41359 3.50260 -3.27297
C -3.06103 3.56880 -1.18350
C -2.86405 2.99989 0.07804
H -3.80236 4.36093 -1.32475
H -3.45559 3.33955 0.93238
C -1.90868 1.97882 0.26123
C -1.62978 1.27667 1.51444
C -0.64904 0.23647 1.42371
C -0.36797 -0.48206 2.60888
H 0.37221 -1.29025 2.58559
C -1.01334 -0.18797 3.82199
H -0.77121 -0.76606 4.72220
C -1.96863 0.84696 3.89180
H -2.46688 1.07924 4.83879
C -2.27751 1.57468 2.73697
C -1.69222 -1.68852 -0.29889
C -1.42396 -2.84825 0.45223
C -2.98239 -1.11904 -0.29475
C -2.42844 -3.37662 1.28212
C -3.97350 -1.66380 0.53739
C -3.70586 -2.79245 1.33379
H -0.44697 -3.33417 0.41890
H -3.21218 -0.25975 -0.92912
H -2.20130 -4.26412 1.88392
H -4.96852 -1.20438 0.54532
Br -0.81355 -1.62802 -2.32489
H -3.02264 2.37706 2.79134
H -4.48705 -3.22165 1.96877
H 4.46268 0.96807 2.41242
N 1.43839 0.52552 -2.12906
C 2.13604 0.84081 -3.02047

C   3.00025 1.24415 -4.12249
H   2.47305 1.95179 -4.78296
H   3.90724 1.73230 -3.72998
H   3.29993 0.36279 -4.71220

59
$16^{bis}a$
SCF(BP86)= -1430.42000503
G(298 K) = -1430.031517
SCF(PBE) = -3989.62217203
Low Freq. = 28.2269cm-1,
33.6060cm-1
C -3.28073 2.36758 -1.23156
C -2.43703 1.30919 -0.81271
C -1.10956 1.58176 -0.38233
C -0.67981 2.92206 -0.32320
C -1.52868 3.96653 -0.72134
C -2.82785 3.68924 -1.19278
C -2.86627 -0.08600 -0.72876
C -4.14174 -0.56958 -1.08914
C -4.45990 -1.91608 -0.90057
C -3.49161 -2.76762 -0.34227
C -2.23948 -2.24283 -0.01569
N -1.91347 -0.93662 -0.21054
Ru -0.06315 -0.07731 0.34119
H -4.88002 0.11763 -1.50926
H -5.44807 -2.29507 -1.17758
H -3.69333 -3.82672 -0.16311
H -1.45232 -2.85794 0.42398
H -3.48443 4.50328 -1.51531
H -1.17331 5.00181 -0.67106
H   0.33201 3.15694 0.02188
N   1.71662 0.85744 0.92726
C   2.12404 0.94752 2.22332
H   1.46861 0.46994 2.95384
C   3.30794 1.58193 2.60063
H   3.58254 1.62282 3.65767
C   4.12215 2.14869 1.60318
C   3.71410 2.06138 0.27159
H   5.05911 2.65011 1.86289
H   4.32692 2.49544 -0.52214
C   2.50322 1.41494 -0.05992
C   1.95196 1.27605 -1.40152
C   0.70629 0.58701 -1.49707
C   0.11249 0.47554 -2.77547
H -0.85352 -0.02740 -2.88214
C   0.73282 1.00233 -3.91425
H   0.24516 0.89917 -4.89018
C   1.97484 1.66616 -3.81331
H   2.45936 2.07554 -4.70503
C   2.57537 1.80279 -2.56099
C   0.92225 -1.62412 -0.76138
C   2.25134 -1.95615 -0.40088
C   0.30450 -2.43455 -1.74042
C   2.90993 -3.06720 -0.95761
C   0.95751 -3.55029 -2.29701
C   2.26582 -3.87830 -1.90748
H   2.78286 -1.35892 0.34499
H -0.70035 -2.19693 -2.10025
H   3.93400 -3.29690 -0.64003
H   0.43666 -4.15629 -3.04778
Br 0.25595 -1.97802 2.32496
H   3.53419 2.32551 -2.47741
H   2.77784 -4.74333 -2.34218
H -4.30037 2.15847 -1.57214
N -0.98665 0.78892 2.04640
C -1.51515 1.26958 2.97342
C -2.16642 1.84835 4.13886
H -1.57667 2.69919 4.51624
H -3.17524 2.20186 3.87164
H -2.25074 1.09040 4.93449

59
TS($16^{bis}a$-13a)
SCF(BP86)= -1430.40721633
G(298 K) = -1430.020037
SCF(PBE) = -3989.60624081
Low Freq. = -69.2446cm-1,
14.0245cm-1
C -3.09496 -0.49221 2.69806
C -2.19253 0.02852 1.74054
C -1.06928 -0.73785 1.32385
C -0.88651 -2.02521 1.87148
C -1.79902 -2.54222 2.80413
C -2.89632 -1.76918 3.23255
C -2.38437 1.29915 1.04626
C -3.40734 2.23190 1.30947
C -3.53015 3.37550 0.51615
C -2.62428 3.56292 -0.54096
C -1.61962 2.61471 -0.74762
N -1.47812 1.50708 0.03004
Ru -0.01878 0.00109 -0.27972
H -4.10367 2.05101 2.13194
H -4.32108 4.10484 0.71405
H -2.68281 4.43344 -1.19914
H -0.89478 2.71698 -1.55553
H -3.59632 -2.16710 3.97379
H -1.64377 -3.54742 3.21118
H -0.02730 -2.63250 1.57310
N   1.31157 -1.58172 -0.61248
C   1.23238 -2.41298 -1.68763
H   0.42099 -2.19505 -2.38428
C   2.12986 -3.46145 -1.89660
H   2.01769 -4.09750 -2.77818
C   3.16363 -3.66581 -0.96586
C   3.24375 -2.82758 0.14841
H   3.89012 -4.47203 -1.10378
H   4.02698 -2.97712 0.89545
C   2.30281 -1.79190 0.32435
C   2.22731 -0.89278 1.47140
C   1.13867 0.02754 1.46896
C   0.98496 0.86650 2.59421
H   0.14008 1.56087 2.64272
C   1.88901 0.82282 3.66510
H   1.73975 1.48533 4.52510
C   2.97956 -0.07090 3.64482
H   3.68775 -0.10128 4.47855
C   3.14205 -0.92705 2.55309
C   1.35970 1.56541 -0.39588
C   2.46329 1.32790 -1.24947
C   1.25332 2.83473 0.20999
C   3.39659 2.34696 -1.52212

```
C   2.18979  3.84967 -0.06148
C   3.26544  3.61468 -0.93280
H   2.59667  0.35409 -1.72729
H   0.44343  3.04731  0.91206
H   4.23123  2.13559 -2.20057
H   2.07387  4.82608  0.42284
Br -0.41246  0.32904 -2.87536
H   3.98479 -1.62647  2.53757
H   3.99554  4.40356 -1.14148
H  -3.96686  0.09329  3.00829
N  -2.04626 -2.10014 -1.19836
C  -3.06721 -2.57068 -1.53731
C  -4.33535 -3.15227 -1.96686
H  -4.20427 -4.22368 -2.18732
H  -5.09071 -3.04259 -1.17247
H  -4.69579 -2.64349 -2.87522
```

59
TS(16^{bis}a-13a+L)
SCF(BP86)= -1430.41466065
G(298 K) = -1430.026237
SCF(PBE) = -3989.61589069
Low Freq. = -218.5067cm-1,
32.6629cm-1

```
C  -3.27160  2.64458 -0.52166
C  -2.44578  1.50578 -0.36071
C  -1.09411  1.64655  0.06832
C  -0.62011  2.93965  0.36358
C  -1.44775  4.06598  0.21297
C  -2.77338  3.92109 -0.24050
C  -2.90987  0.13230 -0.56911
C  -4.21052 -0.24311 -0.96612
C  -4.54724 -1.59437 -1.07739
C  -3.57364 -2.56318 -0.77971
C  -2.29696 -2.14050 -0.40237
N  -1.95570 -0.82809 -0.30955
Ru -0.08154 -0.12553  0.35269
H  -4.95397  0.52916 -1.17824
H  -5.55545 -1.88893 -1.38361
H  -3.79148 -3.63259 -0.83836
H  -1.50012 -2.84414 -0.14918
H  -3.41480  4.79938 -0.36437
H  -1.05610  5.06237  0.44778
H   0.40958  3.07795  0.70900
N   1.72175  0.64823  1.06560
C   2.16033  0.46861  2.34130
H   1.50111 -0.11471  2.98602
C   3.37878  0.97151  2.80057
H   3.67508  0.79783  3.83809
C   4.20228  1.67817  1.90531
C   3.76737  1.85989  0.59139
H   5.17058  2.07261  2.22698
H   4.39496  2.39007 -0.12912
C   2.51612  1.34943  0.17949
C   1.92985  1.50844 -1.14480
C   0.74901  0.73138 -1.42886
C   0.04417  1.06751 -2.62395
H  -0.88822  0.54641 -2.85802
C   0.52580  2.01665 -3.52424
H  -0.04628  2.23160 -4.43356
C   1.74007  2.69640 -3.27564
H   2.12509  3.42962 -3.99043
C   2.42019  2.44811 -2.08207
C   0.92338 -1.13508 -1.32504
C   2.25402 -1.58379 -1.09994
C   0.16130 -1.86399 -2.27977
C   2.77755 -2.70035 -1.76645
C   0.68231 -2.98556 -2.93866
C   1.99701 -3.41769 -2.68925
H   2.89675 -1.05870 -0.39029
H  -0.85377 -1.54750 -2.52895
H   3.80682 -3.01143 -1.55510
H   0.05379 -3.51897 -3.66063
Br  0.51832 -2.57022  1.64887
H   3.32070  3.02548 -1.84698
H   2.40752 -4.28769 -3.21151
H  -4.30860  2.53418 -0.85704
N  -0.93374  0.12383  2.19638
C  -1.45400  0.31059  3.22880
C  -2.08767  0.52321  4.52137
H  -1.53495  1.28695  5.09211
H  -3.12684  0.86270  4.38284
H  -2.09232 -0.41756  5.09560
```

59
13a+L
SCF(BP86)= -1430.44503210
G(298 K) = -1430.055291
SCF(PBE) = -3989.64467550
Low Freq. = 28.1028cm-1,
31.0647cm-1

```
C  -1.23220  3.88875  0.84350
C  -1.25670  2.51558  0.49911
C  -0.12485  1.66161  0.73140
C   0.96629  2.24318  1.42175
C   0.98273  3.60214  1.77803
C  -0.10933  4.43826  1.47046
C  -2.44900  1.84973 -0.03061
C  -3.68857  2.46566 -0.30601
C  -4.76988  1.70197 -0.75342
C  -4.60087  0.31697 -0.91977
C  -3.35056 -0.24536 -0.64599
N  -2.29501  0.49275 -0.21983
Ru -0.38735 -0.27915  0.21000
H  -3.79855  3.54341 -0.16171
H  -5.73170  2.17859 -0.96585
H  -5.41776 -0.32456 -1.26027
H  -3.14649 -1.31453 -0.75932
H  -0.09107  5.49947  1.73882
H   1.85356  4.01387  2.30213
H   1.83144  1.62599  1.68100
N   1.52359 -1.07156  0.85372
C   1.43799 -2.13439  1.71004
H   0.42566 -2.43952  1.97152
C   2.53644 -2.83474  2.21191
H   2.36701 -3.67037  2.89614
C   3.82301 -2.46335  1.79971
C   3.93580 -1.38258  0.92303
H   4.71098 -3.00324  2.14088
H   4.91527 -1.07048  0.55265
C   2.79350 -0.68003  0.47756
C   2.98848  0.51791 -0.37455
```

C   2.09233 0.84285 -1.42316
C   2.29816 2.02959 -2.15865
H   1.61925 2.26436 -2.98496
C   3.37114 2.88615 -1.87208
H   3.51003 3.80072 -2.45753
C   4.27074 2.55551 -0.84559
H   5.11314 3.21367 -0.61137
C   4.07545 1.38214 -0.10577
C   1.01290 -0.09449 -1.85259
C   1.39941 -1.38082 -2.36621
C -0.28372 0.39448 -2.18563
C   0.55535 -2.10188 -3.19690
C -1.13096 -0.35611 -3.05476
C -0.71659 -1.58172 -3.55721
H   2.40287 -1.75713 -2.14206
H -0.52073 1.44452 -1.99043
H   0.88304 -3.06522 -3.60095
H -2.09905 0.06314 -3.34553
Br -1.19592 -2.95729 -0.20366
H   4.74898 1.15291 0.72608
H -1.36058 -2.14438 -4.24075
H -2.09967 4.52705 0.64015
N -1.04284 -0.53530 2.02548
C -1.47983 -0.64150 3.11195
C -2.01938 -0.80046 4.45620
H -1.20003 -0.93996 5.18075
H -2.59320 0.09546 4.74514
H -2.68580 -1.67760 4.50308

59
TS(10a-16^{bis}b)
SCF(BP86)= -1430.37418729
G(298 K) = -1429.987757
SCF(PBE) = -3989.57358153
Low Freq. = -198.8894cm-1,
11.8745cm-1
C -3.71474 -0.96341 2.05420
C -2.58486 -0.93976 1.20318
C -1.57473 0.06183 1.35151
C -1.74175 1.00472 2.39013
C -2.86494 0.97538 3.23587
C -3.85810 -0.00758 3.06717
C -2.36135 -1.92559 0.13971
C -3.22101 -3.00325 -0.15602
C -2.90937 -3.89846 -1.18316
C -1.72386 -3.69848 -1.90830
C -0.90725 -2.61430 -1.57653
N -1.19776 -1.73316 -0.58216
Ru -0.03579 -0.06967 0.01439
H -4.13614 -3.13570 0.42646
H -3.57684 -4.73418 -1.41310
H -1.42771 -4.36427 -2.72315
H   0.00936 -2.40883 -2.13358
H -4.73426 -0.03030 3.72314
H -2.96636 1.72428 4.03011
H -0.98612 1.78074 2.54912
N   0.78015 1.70624 0.71702
C   1.69045 1.79922 1.72620
H   2.05116 0.84679 2.11864
C   2.14341 3.01681 2.23651
H   2.87230 3.02125 3.05121
C   1.65072 4.21062 1.67962
C   0.70946 4.13047 0.65006
H   1.98859 5.18367 2.04832
H   0.29684 5.04204 0.20986
C   0.26633 2.87542 0.18246
C -0.76418 2.65837 -0.83445
C -1.09837 1.28624 -1.07578
C -2.11729 1.03224 -2.02563
H -2.40813 -0.00264 -2.24036
C -2.76369 2.07454 -2.71269
H -3.54709 1.84314 -3.44436
C -2.40823 3.41642 -2.46896
H -2.90813 4.22838 -3.00726
C -1.40884 3.70681 -1.53189
C   2.35345 -0.74192 -0.61805
C   3.37692 0.16398 -0.28124
C   2.59082 -2.13155 -0.56122
C   4.60066 -0.32435 0.21224
C   3.81818 -2.60373 -0.06839
C   4.82899 -1.70678 0.32611
H   3.23077 1.23945 -0.40821
H   1.82151 -2.84404 -0.87099
H   5.38396 0.39039 0.48945
H   3.98314 -3.68537 -0.00376
Br 1.25347 -0.09162 -2.46672
H -1.13477 4.75194 -1.34702
H   5.78888 -2.08161 0.69491
H -4.48715 -1.73080 1.93158
N   0.76658 -1.28805 1.59354
C   1.08085 -1.92750 2.52675
C   1.47869 -2.72155 3.68265
H   1.68544 -2.06537 4.54392
H   0.67439 -3.42329 3.95711
H   2.38857 -3.29780 3.44923

59
TS(10a-16^{bis}b)
SCF(BP86)= -1430.40243675
G(298 K) = -1430.013872
SCF(PBE) = -3989.60430968
Low Freq. = 21.7548cm-1,
31.5876cm-1
C   2.36357 3.00957 1.77979
C   1.45111 2.26713 0.98943
C   1.42543 0.84656 1.07151
C   2.29028 0.20893 1.98468
C   3.18539 0.95142 2.76814
C   3.23005 2.35716 2.66096
C   0.48963 2.88263 0.07437
C   0.43947 4.25720 -0.24153
C -0.50602 4.73577 -1.15064
C -1.39679 3.82377 -1.74140
C -1.31152 2.47465 -1.39142
N -0.39849 1.99827 -0.50282
Ru -0.20697 -0.03081 0.02184
H   1.15423 4.94176 0.22135
H -0.54415 5.80012 -1.40070

H -2.15101 4.14416 -2.46465
H -1.97446 1.72580 -1.82571
H 3.93032 2.93638 3.27080
H 3.85495 0.43382 3.46426
H 2.27766 -0.88237 2.07482
N 0.17915 -2.03295 0.51697
C -0.63655 -2.79282 1.29638
H -1.55533 -2.30863 1.63009
C -0.34222 -4.11172 1.64577
H -1.03893 -4.66955 2.27648
C 0.84858 -4.68695 1.17001
C 1.69693 -3.91417 0.37518
H 1.11262 -5.71853 1.42071
H 2.63550 -4.33336 0.00496
C 1.35996 -2.58056 0.05633
C 2.18482 -1.67218 -0.73819
C 1.71861 -0.33064 -0.85562
C 2.47130 0.56979 -1.64093
H 2.13748 1.60731 -1.74712
C 3.64267 0.16042 -2.29134
H 4.20777 0.87960 -2.89520
C 4.09948 -1.16959 -2.16934
H 5.01468 -1.48950 -2.67732
C 3.37097 -2.07928 -1.39859
C -2.36862 -0.28804 -0.02860
C -2.98141 -1.49294 -0.45007
C -3.23458 0.73999 0.41511
C -4.37971 -1.65622 -0.44754
C -4.63562 0.58892 0.41484
C -5.21803 -0.61285 -0.01810
H -2.36281 -2.31907 -0.81618
H -2.81840 1.68652 0.77924
H -4.81212 -2.60454 -0.78929
H -5.26843 1.41508 0.76122
Br -0.62551 -0.62789 -2.53831
H 3.71601 -3.11627 -1.32512
H -6.30650 -0.73667 -0.01864
H 2.38105 4.10310 1.72087
N -0.80740 0.19495 1.94882
C -1.12768 0.29921 3.07023
C -1.53962 0.43347 4.45968
H -1.38771 -0.51914 4.99288
H -0.94761 1.21885 4.95672
H -2.60705 0.70335 4.51018

60
TS(8a-16^{bis}a.OAc)
SCF(BP86)= -1526.23608409
G(298 K) = -1525.848490
SCF(PBE) = -4085.39884163
Low Freq. = -211.9441cm-1,
15.1818cm-1
C 3.43819 0.26109 2.66068
C 2.51496 -0.10741 1.65287
C 1.51502 0.80839 1.19136
C 1.49505 2.09176 1.79093
C 2.41662 2.45673 2.78527
C 3.39255 1.53959 3.22770
C 2.52502 -1.40527 0.97927
C 3.43586 -2.45149 1.22870
C 3.40990 -3.61642 0.45497
C 2.47593 -3.69823 -0.59245
C 1.58614 -2.63907 -0.78764
N 1.55444 -1.52383 -0.00475
Ru 0.28568 0.11009 -0.25059
H 4.17532 -2.33800 2.02632
H 4.11496 -4.43039 0.64838
H 2.43254 -4.56529 -1.25749
H 0.86830 -2.65007 -1.61114
H 4.10881 1.82294 4.00607
H 2.37451 3.46144 3.22333
H 0.73989 2.82003 1.47580
N -0.91472 1.79390 -0.37008
C -0.89024 2.68087 -1.40346
H -0.15407 2.46266 -2.17841
C -1.71103 3.80998 -1.45596
H -1.64141 4.48734 -2.31159
C -2.60467 4.05122 -0.39607
C -2.64281 3.14512 0.66840
H -3.25945 4.92808 -0.40221
H -3.33322 3.30325 1.50172
C -1.80031 2.01416 0.67308
C -1.76165 0.96944 1.69539
C -0.85834 -0.11037 1.41616
C -0.80423 -1.15223 2.37562
H -0.12519 -1.99679 2.20795
C -1.59225 -1.13328 3.53801
H -1.52356 -1.95954 4.25688
C -2.46935 -0.05724 3.79279
H -3.07828 -0.04046 4.70298
C -2.55190 0.99097 2.86916
C -1.89145 -1.42603 -0.87153
C -3.12026 -0.74817 -0.72354
C -1.79085 -2.78769 -0.52441
C -4.21554 -1.41555 -0.15280
C -2.89565 -3.43804 0.05262
C -4.11396 -2.76228 0.24330
H -3.21879 0.29243 -1.04297
H -0.86375 -3.34359 -0.68025
H -5.16076 -0.87396 -0.03000
H -2.79649 -4.49057 0.34302
Br -0.81491 -0.80146 -2.64851
H -3.23205 1.82864 3.06608
H -4.97551 -3.28088 0.67549
H 4.20055 -0.44972 3.00081
O 1.76028 0.65273 -1.86360
C 2.42653 1.70771 -2.22802
O 2.35057 2.86433 -1.74598
C 3.41018 1.44055 -3.38859
H 2.88752 0.97509 -4.24143
H 3.89070 2.37357 -3.72085
H 4.19210 0.73136 -3.06526

60
16^{bis}a.OAc
SCF(BP86)= -1526.28868150
G(298 K) = -1525.898354
SCF(PBE) = -4085.45051142
Low Freq. = 32.1890cm-1,
41.0692cm-1
C -3.46271 -1.75514 1.68199
C -2.54447 -0.87779 1.05242

C -1.22433 -1.31186 0.74473
C -0.87434 -2.64489 1.04360
C -1.79320 -3.51340 1.65205
C -3.08887 -3.06682 1.98550
C -2.88087 0.47947 0.63230
C -4.13626 1.10203 0.80151
C -4.35615 2.38648 0.29976
C -3.30824 3.03369 -0.37815
C -2.08129 2.37915 -0.50585
N -1.84931 1.13540 -0.00456
Ru -0.05884 0.06489 -0.28085
H -4.93678 0.56775 1.31943
H -5.32833 2.87241 0.42658
H -3.43039 4.03382 -0.80238
H -1.23672 2.83169 -1.02894
H -3.79993 -3.74257 2.47180
H -1.49692 -4.54517 1.87465
H   0.12785 -3.00596 0.80190
N   1.66483 -1.04626 -0.63828
C   2.08305 -1.38555 -1.88723
H   1.44189 -1.04138 -2.69863
C   3.25880 -2.09895 -2.12252
H   3.54610 -2.33810 -3.14985
C   4.04299 -2.50114 -1.02520
C   3.62040 -2.16366 0.26134
H   4.96875 -3.06532 -1.17431
H   4.21091 -2.45839 1.13268
C   2.42832 -1.42876 0.44494
C   1.87817 -1.00054 1.72406
C   0.67908 -0.22852 1.65051
C   0.09149 0.17747 2.87349
H -0.83869 0.75444 2.85640
C   0.66781 -0.14672 4.10680
H   0.18410 0.18425 5.03325
C   1.86186 -0.89961 4.16549
H   2.31135 -1.15383 5.13066
C   2.45778 -1.32451 2.97688
C   0.99233 1.76823 0.48534
C   2.34945 1.95167 0.11636
C   0.40385 2.80177 1.25184
C   3.06020 3.11739 0.45367
C   1.10744 3.97332 1.58962
C   2.44261 4.14393 1.18952
H   2.86430 1.18107 -0.46448
H -0.62313 2.69872 1.61448
H   4.10472 3.22022 0.13476
H   0.60497 4.75032 2.17863
Br 0.47729 1.55214 -2.66342
H   3.37691 -1.91972 3.01729
H   2.99441 5.05261 1.45370
H -4.47548 -1.41354 1.92309
O -1.19395 -0.94656 -1.82821
C -1.12260 -2.16282 -2.30842
O -0.32624 -3.06488 -1.97800
C -2.19127 -2.42473 -3.38626
H -2.18587 -1.62614 -4.14665
H -2.01885 -3.39673 -3.87205
H -3.19469 -2.42842 -2.92605

60
TS(8a-16^{bis}b.OAc)
SCF(BP86)= -1526.23529058
G(298 K) = -1525.847491
SCF(PBE) = -4085.39584524
Low Freq. = -201.5895cm-1, 5.2004cm-1
C   3.71496 0.86141 2.15460
C   2.65972 0.80630 1.21481
C   1.52270 -0.04163 1.42485
C   1.50807 -0.80949 2.61512
C   2.55700 -0.74752 3.54919
C   3.66762 0.08803 3.32156
C   2.63889 1.60046 -0.01787
C   3.62597 2.53105 -0.39928
C   3.50020 3.25554 -1.58945
C   2.36488 3.03345 -2.38628
C   1.41671 2.09747 -1.96378
N   1.52875 1.37584 -0.81518
Ru 0.14817 0.00062 -0.05054
H   4.49246 2.68902 0.24863
H   4.26638 3.97847 -1.88530
H   2.20634 3.57207 -3.32475
H   0.52989 1.88489 -2.56520
H   4.48695 0.13479 4.04658
H   2.51011 -1.35690 4.46003
H   0.65866 -1.46897 2.82176
N -0.96011 -1.51080 0.85504
C -1.91308 -1.31585 1.81230
H -2.05785 -0.26601 2.11010
C -2.60518 -2.37222 2.41314
H -3.35400 -2.15304 3.18015
C -2.32939 -3.69264 2.01563
C -1.33708 -3.90408 1.05367
H -2.86533 -4.53930 2.45631
H -1.08044 -4.91976 0.73929
C -0.64392 -2.81100 0.49351
C   0.46731 -2.90082 -0.45396
C   1.03278 -1.63567 -0.82839
C   2.14444 -1.67704 -1.71079
H   2.61423 -0.73700 -2.02389
C   2.65786 -2.88887 -2.20472
H   3.51684 -2.88022 -2.88726
C   2.07391 -4.11685 -1.83136
H   2.47124 -5.06106 -2.21881
C   0.97974 -4.11969 -0.95624
C -2.17447 0.73531 -1.00605
C -2.25715 2.14033 -1.11243
C -3.29377 -0.01164 -0.58496
C -3.42705 2.79470 -0.69627
C -4.45421 0.65897 -0.16318
C -4.52909 2.06290 -0.21388
H -1.41268 2.71845 -1.49504
H -3.26060 -1.10399 -0.58519
H -3.47736 3.88773 -0.76184
H -5.31039 0.07212 0.18888
Br -1.07880 -0.22106 -2.60381
H   0.52883 -5.07608 -0.66484
H -5.44411 2.57948 0.09327
H   4.58058 1.51102 1.97976
O -0.44831 1.96033 1.04988
C -1.12492 2.30322 2.09316
O -1.79481 1.55458 2.85865

C -1.11518 3.81984 2.38550
H -1.78519 4.32995 1.67053
H -1.47318 4.02873 3.40583
H -0.10779 4.24721 2.25185

60
16^{bis}b.OAc
SCF(BP86)= -1526.26853084
G(298 K) = -1525.878579
SCF(PBE) = -4085.42958401
Low Freq. = 31.6004cm-1,
34.6478cm-1
C   2.65132 2.84260 1.59919
C   1.65274 2.16850 0.85256
C   1.52569 0.74966 0.92463
C   2.41137 0.05024 1.77325
C   3.39393 0.72327 2.51379
C   3.52107 2.12571 2.42565
C   0.69633 2.85993 -0.01111
C   0.71581 4.24177 -0.30042
C -0.23430 4.79649 -1.16066
C -1.20313 3.95061 -1.72831
C -1.18400 2.59190 -1.40617
N -0.26378 2.03906 -0.56922
Ru -0.19950 0.01143 -0.05967
H   1.48776 4.87329 0.14670
H -0.21823 5.86648 -1.38921
H -1.96806 4.33046 -2.41095
H -1.91074 1.89319 -1.82198
H   4.29081 2.65047 3.00103
H   4.07137 0.15337 3.16082
H   2.33740 -1.03782 1.84559
N -0.00532 -2.00606 0.43578
C -0.88868 -2.69016 1.21036
H -1.73712 -2.11211 1.57210
C -0.74411 -4.04355 1.52102
H -1.49509 -4.53462 2.14572
C   0.37561 -4.73737 1.03013
C   1.29954 -4.04385 0.24621
H   0.52568 -5.79714 1.25815
H   2.18460 -4.55375 -0.14274
C   1.10044 -2.67717 -0.04933
C   1.99560 -1.85600 -0.86236
C   1.64014 -0.48049 -0.98750
C   2.45364 0.33870 -1.80551
H   2.20521 1.39983 -1.91878
C   3.57459 -0.17529 -2.46889
H   4.18765 0.48477 -3.09396
C   3.92238 -1.53777 -2.33139
H   4.79994 -1.94058 -2.84754
C   3.13252 -2.37095 -1.53482
C -2.35894 -0.12810 -0.05595
C -3.08106 -1.26215 -0.50817
C -3.14045 0.93410 0.46331
C -4.48608 -1.33634 -0.44424
C -4.54736 0.87831 0.52180
C -5.23260 -0.26130 0.06858
H -2.53831 -2.11134 -0.93800
H -2.64017 1.82655 0.85431
H -4.99666 -2.23907 -0.80329
H -5.10687 1.73085 0.92778
O -0.78570 0.62486 1.91994
H   3.39232 -3.43207 -1.44786
H -6.32645 -0.31161 0.11411
H   2.73777 3.93377 1.54704
Br -0.74258 -0.52443 -2.64964
C -0.57905 0.07317 3.09215
O -0.01016 -1.00606 3.34254
C -1.12967 0.95223 4.23059
H -2.16926 1.25624 4.02590
H -1.08530 0.40733 5.18522
H -0.52917 1.87404 4.31696

59
10b
SCF(BP86)= -1430.40888871
G(298 K) = -1430.022404
SCF(PBE) = -3989.60281342
Low Freq. = 11.5574cm-1,
20.3921cm-1
C -4.42406 -0.81711 1.51721
C -3.14509 -0.87336 0.91142
C -2.17388 0.16459 1.13315
C -2.56463 1.22741 1.98870
C -3.83417 1.27348 2.58838
C -4.77319 0.24983 2.35243
C -2.73341 -1.98381 0.04122
C -3.52664 -3.10954 -0.27165
C -3.02652 -4.11581 -1.10340
C -1.72334 -3.98640 -1.61412
C -0.98462 -2.85034 -1.27032
N -1.45919 -1.86060 -0.47193
Ru -0.41092 -0.03705 0.19019
Br 1.74430 -0.49756 -1.47566
H -4.53489 -3.19546 0.14199
H -3.64065 -4.98825 -1.34676
H -1.28233 -4.74645 -2.26465
H   0.03528 -2.70921 -1.64160
H -5.76466 0.28495 2.81575
H -4.09543 2.11364 3.24348
H -1.85233 2.03566 2.18536
C   0.35743 1.74550 0.76796
C   1.11391 2.06351 1.93175
H   1.32101 1.27325 2.66423
C   1.60728 3.35452 2.18148
H   2.18695 3.54973 3.09263
C   1.36572 4.40484 1.27010
C   0.61988 4.14472 0.11425
H   1.75240 5.41080 1.46374
H   0.42660 4.96132 -0.59213
C   0.11956 2.84474 -0.13557
C -0.69011 2.51204 -1.30861
N -1.08714 1.18558 -1.35812
C -1.86647 0.77042 -2.39289
H -2.15196 -0.28415 -2.37035
C -2.27854 1.61611 -3.42518
H -2.90253 1.22169 -4.23134
C -1.87330 2.96282 -3.39382
H -2.17364 3.65663 -4.18487
C -1.08013 3.40367 -2.33071
C   3.49093 -0.75004 -0.64277
C   4.27202 -1.84113 -1.04889

C   3.91815 0.16122 0.33287
C   5.53444 -2.01572 -0.45358
C   5.18266 -0.03601 0.91693
C   5.99001 -1.11740 0.52587
H   3.91052 -2.53913 -1.80909
H   3.27758 0.99362 0.63879
H   6.15782 -2.86130 -0.76130
H   5.53241 0.66536 1.68131
H -0.75776 4.44730 -2.28327
H   6.97297 -1.26092 0.98480
H -5.15635 -1.61244 1.33654
N   0.35721 -1.03685 1.71302
C   0.81208 -1.64059 2.61877
C   1.39072 -2.37576 3.73736
H   0.85129 -2.14232 4.67077
H   1.33063 -3.46338 3.56341
H   2.45076 -2.10151 3.87127

59
TS(10b-16^{bis}c)
SCF(BP86)= -1430.37101188
G(298 K) = -1429.984383
SCF(PBE) = -3989.57261274
Low Freq. = -200.4670cm-1, -13.9834cm-1
C   2.63857 1.45473 -2.54706
C   1.95058 1.15907 -1.34959
N   0.85568 0.32137 -1.37651
C   0.44852 -0.21018 -2.55477
C   1.09435 0.05203 -3.76502
C   2.21327 0.90485 -3.75815
C   2.27558 1.66737 -0.01897
C   1.36790 1.25531 1.01859
C   1.67167 1.71140 2.32953
C   2.78585 2.52370 2.59727
C   3.65422 2.91810 1.55729
C   3.39743 2.48596 0.25113
Ru -0.19296 0.10725 0.43670
N -2.02941 -0.95568 -0.27151
C -2.87156 -0.16966 -1.02277
C -4.07960 -0.69224 -1.53530
C -4.45967 -2.00339 -1.23546
C -3.63422 -2.77169 -0.39770
C -2.43894 -2.20588 0.05700
C -2.43853 1.22998 -1.15674
C -1.24016 1.60689 -0.47050
C -0.87966 2.97395 -0.49066
C -1.64899 3.92894 -1.17796
C -2.80641 3.53848 -1.87700
C -3.19901 2.19555 -1.86029
Br 0.50292 -1.97447 2.18680
C   1.23837 -1.96348 0.06245
C   0.69865 -2.94709 -0.78773
C   1.53789 -3.60026 -1.71058
C   2.91160 -3.31473 -1.76563
C   3.44718 -2.36342 -0.87671
C   2.62646 -1.70377 0.05098
N -1.25540 0.51079 2.09543
C -1.89586 0.80217 3.03602
C -2.67657 1.18374 4.20533
H -4.72757 -0.06394 -2.15147
H -5.39525 -2.41166 -1.62957
H -3.90323 -3.78852 -0.09990
H -1.77200 -2.75830 0.72638
H -3.40190 4.27706 -2.42331
H -1.34072 4.98100 -1.17294
H   0.02581 3.29640 0.03223
H   1.01880 1.42223 3.16149
H   2.98394 2.85441 3.62434
H   4.52220 3.55132 1.76821
H   4.07706 2.78369 -0.55622
H -0.42159 -0.86850 -2.49935
H   0.72467 -0.40389 -4.68696
H   2.74349 1.13721 -4.68677
H -0.35977 -3.21029 -0.75466
H   3.05638 -0.98288 0.74967
H   1.10019 -4.34745 -2.38250
H   4.51998 -2.13928 -0.88730
H   3.50316 2.12305 -2.51879
H   3.55973 -3.83841 -2.47519
H -4.10994 1.90126 -2.39275
H -2.01669 1.32668 5.07681
H -3.21004 2.12816 4.00755
H -3.41806 0.40404 4.44424

59
16^{bis}c
SCF(BP86)= -1430.39850881
G(298 K) = -1430.009463
SCF(PBE) = -3989.59846791
Low Freq. = -35.0608cm-1, 26.1288cm-1
C -2.94309 -1.36828 -2.55492
C -2.22160 -1.03777 -1.38395
N -0.95838 -0.50449 -1.48809
C -0.40311 -0.32676 -2.71589
C -1.07196 -0.64567 -3.89428
C -2.37621 -1.17201 -3.81203
C -2.66890 -1.22814 -0.01471
C -1.72203 -0.82026 0.97891
C -2.09774 -1.02784 2.33115
C -3.33718 -1.58981 2.67395
C -4.25788 -1.97155 1.67479
C -3.92186 -1.79165 0.33132
Ru 0.02732 0.00992 0.31648
N   2.03896 0.88286 -0.21611
C   2.94380 0.00802 -0.76171
C   4.26538 0.41240 -1.04981
C   4.66755 1.72020 -0.76847
C   3.74036 2.60557 -0.19361
C   2.44570 2.14579 0.06425
C   2.41599 -1.34375 -0.97011
C   1.08565 -1.58823 -0.53746
C   0.55988 -2.89004 -0.64264
C   1.32376 -3.92983 -1.19709
C   2.63245 -3.68138 -1.65505
C   3.17408 -2.39821 -1.53861
Br 0.02607 1.73571 2.42951
C -0.83154 1.77640 -0.54048
C -0.20344 2.54199 -1.54542
C -0.80086 3.70984 -2.06182
C -2.05503 4.13277 -1.59669

```
C  -2.70628  3.36742 -0.61387
C  -2.10670  2.20330 -0.09877
N   0.95191 -1.04646  1.89827
C   1.47074 -1.62224  2.77471
C   2.11151 -2.31675  3.88155
H   4.97226 -0.29912 -1.48340
H   5.68979  2.04302 -0.98771
H   4.00853  3.63640  0.05216
H   1.68872  2.78966  0.51783
H   3.22667 -4.48802 -2.09560
H   0.89532 -4.93520 -1.27516
H  -0.45796 -3.09470 -0.29576
H  -1.41309 -0.72694  3.12973
H  -3.59310 -1.73092  3.73079
H  -5.22433 -2.40719  1.94785
H  -4.63023 -2.09299 -0.44849
H   0.60997  0.07756 -2.72752
H  -0.57533 -0.48407 -4.85398
H  -2.93335 -1.42863 -4.71767
H   0.76766  2.24133 -1.94934
H  -2.63469  1.63592  0.67219
H  -0.27622  4.28234 -2.83583
H  -3.69009  3.67369 -0.23873
H  -3.94916 -1.78387 -2.45907
H  -2.52118  5.03949 -1.99683
H   4.19562 -2.21326 -1.88683
H   1.34834 -2.73700  4.55623
H   2.74343 -3.13502  3.50048
H   2.74196 -1.61307  4.44913
```

59
10c
ru_cis_nc2_phbr_tN_mecn_tC_2
SCF(BP86)= -1430.39896358
G(298 K) = -1430.012562
SCF(PBE) = -3989.59343770
Low Freq. = 8.5071cm-1, 15.5962cm-1

```
C  -4.16723 -1.31263  1.69888
C  -2.94326 -1.23046  1.00065
N  -2.11003 -0.14030  1.19627
C  -2.49402  0.83898  2.05962
C  -3.69440  0.79605  2.77114
C  -4.55102 -0.30433  2.58641
C  -2.42107 -2.20773  0.04944
C  -3.11015 -3.39551 -0.29352
C  -2.55380 -4.29198 -1.21240
C  -1.29847 -3.99355 -1.78368
C  -0.61619 -2.81478 -1.44115
C  -1.14016 -1.86469 -0.51913
Ru -0.36150 -0.10341  0.14275
Br  1.61217 -0.10445 -1.53783
H  -4.08355 -3.62512  0.15706
H  -3.08458 -5.21159 -1.48023
H  -0.85026 -4.69119 -2.50264
H   0.35714 -2.62659 -1.91114
H  -5.50128 -0.37183  3.12456
H  -3.94602  1.61383  3.45144
H  -1.79590  1.67209  2.16350
N   0.26687  1.89335  0.75834
C   1.05742  2.19693  1.82056
H   1.36596  1.34432  2.43321
C   1.46302  3.49791  2.13192
H   2.10020  3.67675  3.00231
C   1.03390  4.54880  1.30164
C   0.21754  4.25148  0.20572
H   1.33087  5.58202  1.50597
H  -0.12565  5.05494 -0.45151
C  -0.16629  2.91841 -0.05846
C  -1.03259  2.48493 -1.16672
C  -1.27515  1.06891 -1.27072
C  -2.12638  0.65981 -2.33190
H  -2.34314 -0.40839 -2.44995
C  -2.69557  1.57200 -3.23705
H  -3.34466  1.20892 -4.04363
C  -2.43607  2.95145 -3.11452
H  -2.87673  3.66628 -3.81749
C  -1.60693  3.40190 -2.08012
C   3.35889 -0.52870 -0.76562
C   3.92005 -1.78659 -1.02621
C   4.01048  0.43592  0.01563
C   5.18358 -2.08105 -0.48204
C   5.27145  0.12048  0.55304
C   5.85745 -1.13245  0.30515
H   3.38868 -2.52153 -1.63697
H   3.54768  1.40813  0.20446
H   5.63617 -3.05829 -0.67781
H   5.79387  0.86333  1.16421
H  -1.41188  4.47667 -1.98627
H   6.83992 -1.36906  0.72487
H  -4.81605 -2.17759  1.53672
N   0.66176 -1.05851  1.67386
C   1.22744 -1.57835  2.56797
C   1.93082 -2.22056  3.67240
H   1.46320 -1.95008  4.63382
H   1.90113 -3.31754  3.56427
H   2.98511 -1.89846  3.69344
```

59
TS(10c-16^{bis}d)
SCF(BP86)= -1430.35836178
G(298 K) = -1429.972413
SCF(PBE) = -3989.56057745
Low Freq. = -231.7668cm-1, 23.9579cm-1

```
C   3.55076 -3.14134 -0.98743
C   2.24825 -3.65012 -0.84098
C   1.28126 -2.96148 -0.08810
C   1.61949 -1.72416  0.48845
C   2.93179 -1.21818  0.38909
C   3.88248 -1.92310 -0.36659
Br  0.76568 -1.30413  2.45671
Ru -0.29083  0.10862  0.40206
N  -1.39956  0.84653  2.05553
C  -2.02996  1.33070  2.92094
C  -2.80814  1.94010  3.99214
N  -1.56565  1.24519 -0.74333
C  -2.66807  0.60620 -1.27804
C  -3.56628  1.33110 -2.09247
C  -3.36093  2.68912 -2.34058
```

C -2.24872 3.32722 -1.76106
C -1.37730 2.57028 -0.97733
C -2.79998 -0.78842 -0.87257
C -3.88746 -1.60454 -1.26659
C -4.01563 -2.89906 -0.75299
C -3.06673 -3.35873 0.18344
C -1.98384 -2.54880 0.56466
C -1.77018 -1.25007 0.02426
N 1.25863 1.62573 0.62496
C 1.52274 2.33473 1.75186
C 2.50990 3.32194 1.82086
C 3.26735 3.59191 0.66702
C 3.01203 2.86081 -0.49732
C 2.00463 1.87153 -0.50809
C 1.65795 1.01483 -1.65079
C 0.66391 0.00769 -1.41606
C 0.33992 -0.83561 -2.50437
C 0.95606 -0.69834 -3.76010
C 1.92561 0.30173 -3.97309
C 2.27490 1.15215 -2.91852
H -4.64234 -1.22737 -1.96673
H -4.85334 -3.53516 -1.05686
H -3.17755 -4.35760 0.62349
H -1.29004 -2.93685 1.31970
H -4.05923 3.24730 -2.97132
H -2.04961 4.39061 -1.91501
H -0.49217 3.01129 -0.51573
H 0.90372 2.08556 2.61865
H 2.67676 3.86188 2.75671
H 4.04872 4.35796 0.67755
H 3.59859 3.04904 -1.40039
H -0.41175 -1.61953 -2.36228
H 0.67898 -1.37233 -4.57998
H 2.40305 0.41430 -4.95211
H 0.27648 -3.37446 0.01963
H 3.20857 -0.28824 0.89095
H 1.97109 -4.60184 -1.30892
H 4.89669 -1.51655 -0.45165
H 3.02870 1.92987 -3.08685
H 4.30138 -3.69387 -1.56105
H -4.43173 0.81826 -2.51980
H -2.13813 2.35912 4.76044
H -3.43357 2.75179 3.58602
H -3.46417 1.18958 4.46211

59
16[bis]d
SCF(BP86)= -1430.39977495
G(298 K) = -1430.011672
SCF(PBE) = -3989.60142457
Low Freq. = 29.5166cm-1,
33.9250cm-1
C -2.01374 4.16491 -1.56418
C -0.68854 3.85309 -1.90698
C -0.09442 2.65741 -1.46180
C -0.79351 1.74377 -0.64197
C -2.13785 2.06411 -0.32691
C -2.73621 3.25447 -0.77286
Br -0.39525 1.67253 2.40792
Ru 0.11327 0.03586 0.35646
N 0.95571 -0.98757 1.98718
C 1.44346 -1.58044 2.87034
C 2.03853 -2.29661 3.98844
N 1.18720 -1.53728 -0.55095
C 2.49084 -1.28188 -0.90275
C 3.28029 -2.32411 -1.44211
C 2.75073 -3.60537 -1.58828
C 1.42334 -3.84894 -1.18611
C 0.67619 -2.78782 -0.67806
C 2.92796 0.07910 -0.62282
C 4.24221 0.53851 -0.89022
C 4.61647 1.83242 -0.52181
C 3.67646 2.66304 0.12402
C 2.37206 2.21028 0.37638
C 1.94128 0.91279 -0.00160
N -1.80280 -0.96100 0.85436
C -2.22943 -1.22956 2.11388
C -3.46308 -1.82529 2.38832
C -4.30867 -2.14700 1.31168
C -3.88096 -1.87453 0.01040
C -2.61377 -1.28836 -0.20525
C -2.02596 -0.99431 -1.51214
C -0.78150 -0.29460 -1.52567
C -0.13922 -0.11513 -2.77277
C -0.71841 -0.56541 -3.96462
C -1.97266 -1.21204 -3.95030
C -2.61267 -1.42788 -2.72933
H 4.97280 -0.11672 -1.37781
H 5.63013 2.19262 -0.72499
H 3.96629 3.67574 0.42915
H 1.66271 2.87859 0.87406
H 3.36324 -4.41121 -2.00326
H 0.96805 -4.83851 -1.26960
H -0.36264 -2.91297 -0.36703
H -1.54742 -0.93301 2.91425
H -3.75299 -2.02200 3.42372
H -5.28833 -2.60182 1.48662
H -4.52527 -2.10928 -0.84043
H 0.82605 0.39867 -2.80952
H -0.19190 -0.40897 -4.91282
H -2.43192 -1.55591 -4.88215
H 0.93278 2.44425 -1.76710
H -2.72915 1.38479 0.29308
H -0.10488 4.53861 -2.53294
H -3.77522 3.46772 -0.49480
H -3.56769 -1.96354 -2.71086
H -2.47860 5.09295 -1.91363
H 4.31359 -2.11705 -1.73062
H 1.53648 -2.01010 4.92669
H 1.92763 -3.38275 3.84002
H 3.11045 -2.05315 4.06471

53
13a
SCF(BP86)= -1297.65903475
G(298 K) = -1297.311982
SCF(PBE) = -3856.96577940
Low Freq. = 33.9630cm-1,
41.9340cm-1
C 2.89678 -0.76385 2.68705
C 2.22214 -0.50705 1.46823
C 0.83796 -0.17186 1.46048

```
C    0.16195 -0.13547  2.69788
C    0.83375 -0.39160  3.90323
C    2.20902 -0.69901  3.90201
C    2.85688 -0.61060  0.15740
C    4.22089 -0.88364 -0.07606
C    4.70500 -0.97582 -1.38245
C    3.81094 -0.79314 -2.45184
C    2.47082 -0.52519 -2.16952
N    1.98764 -0.43445 -0.89979
Ru  -0.00470  0.10760 -0.43296
H    4.89631 -1.01686  0.77243
H    5.76291 -1.18489 -1.56623
H    4.13926 -0.85589 -3.49222
H    1.72922 -0.38667 -2.96083
H    2.73342 -0.89781  4.84170
H    0.28146 -0.35469  4.84887
H   -0.91085  0.08367  2.72614
N   -2.00436  0.63091 -0.05601
C   -2.54868  1.82572 -0.40759
H   -1.86221  2.55091 -0.84797
C   -3.90006  2.12351 -0.22087
H   -4.28152  3.10189 -0.52308
C   -4.73496  1.15261  0.35940
C   -4.19179 -0.09010  0.69335
H   -5.79524  1.35792  0.53291
H   -4.82200 -0.87406  1.12047
C   -2.82546 -0.34634  0.45981
C   -2.14505 -1.62262  0.65287
C   -0.76793 -1.63319  0.30052
C   -0.05262 -2.84543  0.35699
H    1.00988 -2.87182  0.09763
C   -0.69943 -4.03009  0.74868
H   -0.13478 -4.96848  0.77557
C   -2.05852 -4.01416  1.12044
H   -2.55245 -4.93654  1.44109
C   -2.77942 -2.81669  1.06995
C    0.41516  1.94465  0.37806
C    1.32797  2.59405 -0.48953
C   -0.20935  2.69889  1.39318
C    1.53337  3.98715 -0.39213
C    0.02282  4.07995  1.50036
C    0.88874  4.73334  0.60433
H    1.87846  2.04046 -1.25693
H   -0.89251  2.21660  2.09715
H    2.22997  4.47067 -1.08595
H   -0.48172  4.64739  2.29031
Br  -0.87994 -0.51162 -2.82089
H   -3.84141 -2.81199  1.33748
H    1.06978  5.80914  0.69425
H    3.96016 -1.02588  2.68361

53
TS(13a-17a pi)
SCF(BP86)= -1297.64985888
G(298 K) = -1297.303060
SCF(PBE) = -3856.95654993
Low Freq. = -193.0829cm-1,
32.9329cm-1
C    2.89144 -0.59339  2.62450
C    2.24849 -0.20519  1.43075
C    0.88333  0.25805  1.44588
```

```
C    0.18750  0.13410  2.68588
C    0.82683 -0.27909  3.85633
C    2.19845 -0.60666  3.84138
C    2.86165 -0.40654  0.11778
C    4.23748 -0.61309 -0.10834
C    4.70674 -0.86454 -1.40089
C    3.78056 -0.90329 -2.45575
C    2.42936 -0.67501 -2.18063
N    1.95930 -0.42607 -0.92883
Ru  -0.04433  0.11207 -0.48274
Br  -0.91027 -0.57576 -2.81399
H    4.93375 -0.56406  0.73246
H    5.77381 -1.02118 -1.58266
H    4.09223 -1.09593 -3.48550
H    1.67026 -0.68522 -2.96752
H    2.70712 -0.91301  4.76019
H    0.25526 -0.35046  4.78772
H   -0.87603  0.38521  2.72943
N   -2.03357  0.56222 -0.08984
C   -2.64199  1.72214 -0.45130
H   -1.99375  2.47606 -0.90378
C   -4.00578  1.95074 -0.25641
H   -4.44281  2.90341 -0.56573
C   -4.78219  0.94117  0.34029
C   -4.16990 -0.26552  0.68936
H   -5.85153  1.08957  0.51683
H   -4.75623 -1.07472  1.13207
C   -2.79243 -0.45313  0.45336
C   -2.03266 -1.67969  0.69111
C   -0.65797 -1.63218  0.30430
C    0.12300 -2.79649  0.46348
H    1.17976 -2.78826  0.17860
C   -0.44231 -3.97695  0.97715
H    0.18004 -4.87281  1.08361
C   -1.79803 -4.01447  1.35902
H   -2.23242 -4.93394  1.76385
C   -2.59021 -2.87019  1.21435
C    0.52635  1.82499  0.62802
C    1.51980  2.46649 -0.17778
C   -0.36093  2.68633  1.34827
C    1.55389  3.86743 -0.31938
C   -0.30526  4.07440  1.21072
C    0.64566  4.68326  0.36603
H    2.28399  1.88665 -0.69608
H   -1.11065  2.26219  2.01862
H    2.32178  4.31200 -0.96184
H   -1.01550  4.69082  1.77254
H    0.68509  5.77210  0.26539
H   -3.64646 -2.90515  1.50327
H    3.92691 -0.94819  2.59438

53
17a pi
SCF(BP86)= -1297.67700889
G(298 K) = -1297.329584
SCF(PBE) = -3856.98353582
Low Freq. = 25.7725cm-1,
30.5176cm-1
C    0.05048  1.28113  2.69646
C   -0.79320  0.75263  1.66203
C   -0.95722 -0.70331  1.53698
```

```
C  -0.17786 -1.51391  2.44878
C   0.67266 -0.96494  3.39010
C   0.77764  0.45015  3.52928
C  -1.62346  1.67988  0.84448
C  -2.38160  2.78271  1.25306
C  -3.06941  3.51796  0.27029
C  -2.97306  3.12761 -1.07639
C  -2.16209  2.03755 -1.42162
N  -1.48474  1.34807 -0.47447
Ru  0.03202 -0.06436 -0.38585
Br  0.26226 -0.28159 -2.89243
H  -2.44557  3.04346  2.31306
H  -3.68087  4.37928  0.55423
H  -3.50647  3.67051 -1.86103
H  -2.00842  1.71430 -2.45504
H   1.42320  0.87698  4.30306
H   1.23579 -1.62128  4.06127
H  -0.31780 -2.59902  2.41588
N   1.66105 -1.33272 -0.14246
C   1.64006 -2.68163 -0.30309
H   0.68054 -3.10773 -0.60571
C   2.76221 -3.49019 -0.11229
H   2.67924 -4.57074 -0.25332
C   3.97615 -2.88340  0.25820
C   4.02116 -1.49291  0.38535
H   4.87405 -3.48475  0.42797
H   4.95805 -0.99259  0.64419
C   2.86095 -0.72206  0.16374
C   2.78036  0.73599  0.16264
C   1.49120  1.27259 -0.15826
C   1.38004  2.67803 -0.29226
H   0.42023  3.13060 -0.56078
C   2.48712  3.51561 -0.07788
H   2.37038  4.60039 -0.18512
C   3.74168  2.97660  0.27388
H   4.59880  3.63519  0.44602
C   3.88645  1.59024  0.38567
C  -2.17685 -1.34480  0.92742
C  -3.45992 -0.74552  0.99052
C  -2.07883 -2.62847  0.33472
C  -4.58191 -1.38582  0.44619
C  -3.20154 -3.26679 -0.21481
C  -4.45874 -2.64418 -0.16942
H  -3.58473  0.22045  1.48659
H  -1.11192 -3.13862  0.31319
H  -5.56205 -0.90254  0.51486
H  -3.09040 -4.25524 -0.67215
H   4.86644  1.16837  0.63582
H  -5.33713 -3.13969 -0.59481
H   0.12167  2.36831  2.80021

53
TS(17a pi-17a pi')
SCF(BP86)= -1297.66543467
G(298 K) = -1297.318243
SCF(PBE) = -3856.96980997
Low Freq. = -103.5951cm-1,
23.8959cm-1
C  -0.12072  0.47558 -2.55218
C   1.00557  0.32530 -1.69131
C   1.57539 -0.99364 -1.52305
```

```
C   1.02294 -2.06345 -2.26808
C  -0.05432 -1.87493 -3.14735
C  -0.64002 -0.60708 -3.28209
C   1.59278  1.55610 -1.05050
C   2.38392  2.54908 -1.62990
C   2.78921  3.63314 -0.82579
C   2.38124  3.67655  0.51707
C   1.55446  2.66424  1.02326
N   1.15313  1.63215  0.23875
Ru -0.21426  0.08047  0.33523
Br -0.46120  0.18531  2.81958
H   2.68476  2.46763 -2.67821
H   3.41688  4.42583 -1.24240
H   2.68415  4.49820  1.17170
H   1.17392  2.66416  2.04836
H  -1.49188 -0.45224 -3.95044
H  -0.43561 -2.72314 -3.72463
H   1.47572 -3.05477 -2.16902
N  -1.63233 -1.41251  0.19744
C  -1.40065 -2.73557  0.40352
H  -0.37026 -3.00020  0.65551
C  -2.40136 -3.70603  0.31439
H  -2.15396 -4.75565  0.49225
C  -3.71016 -3.29776 -0.00155
C  -3.96307 -1.93791 -0.20522
H  -4.51996 -4.02904 -0.08116
H  -4.97363 -1.59477 -0.44292
C  -2.91832 -0.99719 -0.09813
C  -3.02961  0.45034 -0.25801
C  -1.80200  1.17973 -0.07667
C  -1.86443  2.59289 -0.19479
H  -0.95962  3.19317 -0.05404
C  -3.07120  3.24636 -0.49284
H  -3.08401  4.33970 -0.57698
C  -4.26295  2.51552 -0.68220
H  -5.19933  3.03160 -0.91639
C  -4.23692  1.12180 -0.56129
C   2.76013 -1.26581 -0.65273
C   3.92812 -0.46831 -0.69657
C   2.74930 -2.38881  0.21030
C   5.03498 -0.77316  0.11031
C   3.85371 -2.68844  1.02165
C   5.00058 -1.87809  0.97789
H   3.98208  0.37974 -1.38368
H   1.86048 -3.02652  0.24913
H   5.93196 -0.14802  0.05274
H   3.81635 -3.55594  1.68837
H  -5.16233  0.55102 -0.70154
H   5.86407 -2.11014  1.60935
H  -0.55573  1.47249 -2.66348

53
17a pi'
SCF(BP86)= -1297.67757517
G(298 K) = -1297.330022
SCF(PBE) = -3856.98109466
Low Freq. = 23.1942cm-1,
32.8395cm-1
C   0.00833 -0.07651 -2.05843
C   1.23279  0.02086 -1.28847
C   2.06189 -1.15559 -1.10239
```

```
C   1.62373 -2.36422 -1.65355
C   0.41796 -2.46481 -2.39877
C  -0.36634 -1.34124 -2.61406
C   1.66723  1.42132 -0.96892
C   2.78365  2.14995 -1.38322
C   2.89744  3.48605 -0.95361
C   1.89813  4.03475 -0.13239
C   0.80822  3.24277  0.25464
N   0.70382  1.95597 -0.15712
Ru -0.45605  0.27119  0.19573
Br -1.03072  0.96467  2.55519
H   3.53939  1.68512 -2.02151
H   3.75525  4.09145 -1.26002
H   1.95898  5.06991  0.21384
H   0.01236  3.60945  0.90846
H  -1.26526 -1.40019 -3.23608
H   0.12742 -3.43208 -2.82030
H   2.22673 -3.26384 -1.49174
N  -1.49133 -1.49365  0.43879
C  -0.97304 -2.64252  0.94086
H   0.09104 -2.61070  1.19169
C  -1.73946 -3.79601  1.13391
H  -1.27117 -4.69368  1.54547
C  -3.10258 -3.76465  0.79286
C  -3.64343 -2.58498  0.26867
H  -3.73408 -4.64684  0.93334
H  -4.70076 -2.53784 -0.00530
C  -2.82765 -1.45074  0.09137
C  -3.24196 -0.15822 -0.46439
C  -2.20843  0.83223 -0.55699
C  -2.53666  2.08506 -1.12207
H  -1.76661  2.85881 -1.21528
C  -3.84568  2.35974 -1.55743
H  -4.07812  3.34303 -1.98293
C  -4.85795  1.38597 -1.44797
H  -5.87543  1.60524 -1.78659
C  -4.55368  0.13093 -0.90517
C   3.34243 -1.10343 -0.34507
C   4.47982 -1.79575 -0.82534
C   3.45422 -0.40020  0.87893
C   5.68414 -1.78906 -0.10592
C   4.65828 -0.39698  1.59892
C   5.77864 -1.09029  1.10959
H   4.42106 -2.32410 -1.78278
H   2.58528  0.12973  1.28345
H   6.55332 -2.32527 -0.50087
H   4.71849  0.14435  2.54883
H  -5.34144 -0.62712 -0.82649
H   6.71845 -1.08459  1.67095
H  -0.42151  0.83014 -2.49528
```

53
TS(17a p'-17aCH)
SCF(BP86)= -1297.66774
G(298 K) = -1297.320889
SCF(PBE) = -3856.97035672
Low Freq. = -123.8214cm-1,
23.5880cm-1

```
C   4.25903  2.24196  0.10500
C   3.27723  1.24264 -0.04722
N   2.02855  1.40159  0.52319
```

```
C   1.76462  2.52477  1.23936
C   2.70338  3.54424  1.42013
C   3.97655  3.39889  0.83947
C   3.41006 -0.01501 -0.78287
C   2.23898 -0.85634 -0.76784
C   2.31405 -2.07994 -1.48413
C   3.48101 -2.45335 -2.17220
C   4.61869 -1.62041 -2.17113
C   4.57724 -0.40438 -1.47758
Ru  0.70265 -0.15600  0.24586
N  -0.69353 -1.62313 -0.14512
C  -1.79182 -1.12005 -0.80470
C  -2.88887 -1.92680 -1.13826
C  -2.87100 -3.28672 -0.79488
C  -1.75070 -3.79686 -0.11560
C  -0.68751 -2.94473  0.19216
C  -1.64888  0.32183 -1.15990
C  -2.66558  1.29821 -0.93810
C  -2.42192  2.62028 -1.37348
C  -1.21317  3.00521 -1.97932
C  -0.20328  2.05508 -2.17002
C  -0.42253  0.72146 -1.76950
C  -3.94988  0.99864 -0.23999
C  -5.17673  1.40700 -0.81159
C  -6.39060  1.16949 -0.14789
C  -6.39816  0.52690  1.10134
C  -5.18365  0.12430  1.68377
C  -3.96998  0.35568  1.01963
Br  1.39791 -1.11173  2.45076
H  -3.73860 -1.48569 -1.66512
H  -3.71217 -3.93533 -1.05439
H  -1.69469 -4.84936  0.17517
H   0.20094 -3.28658  0.72881
H   0.74621  2.32911 -2.63908
H  -1.06704  4.04401 -2.28977
H  -3.19691  3.37384 -1.19777
H   0.76033  2.58453  1.67054
H   2.43708  4.42790  2.00564
H   4.73733  4.17572  0.96063
H   5.24334  2.10544 -0.35124
H   1.44900 -2.75145 -1.50544
H   3.50409 -3.40569 -2.71551
H   5.52527 -1.91846 -2.70715
H  -5.17635  1.89730 -1.79110
H  -3.02756  0.05408  1.48858
H  -7.33160  1.48441 -0.61061
H  -5.17978 -0.36640  2.66255
H   5.45931  0.24682 -1.47876
H  -7.34454  0.34275  1.61986
H   0.29820 -0.05342 -2.07493
```

53
17aCH
SCF(BP86)= -1297.673508
G(298 K) = -1297.326894
SCF(PBE) = -3856.97548020
Low Freq. = 24.4610cm-1,
27.7850cm-1

```
C   5.25706  1.24255 -1.09834
C   4.13708  0.97135 -0.28082
C   4.35449  0.47611  1.02568
```

```
C   5.65622  0.24794  1.49533
C   6.76345  0.50883  0.66868
C   6.55990  1.00700 -0.62873
C   2.75570  1.28609 -0.76242
C   1.69473  0.33758 -0.83381
C   0.36832  0.79369 -1.13066
C   0.13717  2.15398 -1.44438
C   1.19718  3.06684 -1.43522
C   2.48751  2.63454 -1.09171
C   1.82156 -1.11087 -0.58797
N   0.63687 -1.74013 -0.24110
C   0.66100 -3.08319 -0.00602
C   1.81437 -3.85957 -0.12866
C   3.00774 -3.23953 -0.53180
C   3.00316 -1.85979 -0.76010
Ru -1.13919 -0.64971 -0.44187
Br -2.63287 -2.33996  0.74624
N  -2.82641  0.46177 -0.86475
C  -3.16533  1.43939  0.04870
C  -4.31104  2.23427 -0.15291
C  -5.11178  2.03532 -1.28204
C  -4.75484  1.03443 -2.20400
C  -3.61105  0.27175 -1.95661
C  -2.22529  1.52399  1.16648
C  -1.12314  0.61328  1.10252
C  -0.18030  0.63065  2.15202
C  -0.33005  1.51132  3.23854
C  -1.41920  2.40378  3.29411
C  -2.36387  2.40850  2.26099
H   3.91185 -1.35097 -1.08428
H   3.92485 -3.81900 -0.67114
H   1.76383 -4.93268  0.07358
H  -0.29936 -3.51707  0.28644
H  -0.87509  2.48010 -1.70169
H   1.02015  4.11938 -1.67915
H   3.30878  3.35619 -1.03866
H  -3.29034 -0.52338 -2.63673
H  -5.35036  0.84044 -3.09953
H  -6.00259  2.64951 -1.44280
H  -4.56810  3.00529  0.57834
H   0.67347 -0.05467  2.12580
H   0.40938  1.50234  4.04764
H  -1.52946  3.08841  4.14098
H   3.49566  0.28189  1.67648
H   5.10070  1.62842 -2.11142
H   5.80687 -0.12858  2.51247
H   7.41608  1.21446 -1.27903
H  -3.21480  3.09750  2.30887
H   7.77883  0.32833  1.03605
H  -0.36319  0.08733 -1.79885
```

53
TS(17aCH-17a'CH)
SCF(BP86)= -1297.653172
G(298 K) = -1297.307903
SCF(PBE) = -3856.95200648
Low Freq. = -526.2977cm-1,
14.7919cm-1

```
C   4.64249  0.31237 -1.29189
C   4.45336  0.94644 -0.04087
C   5.57635  1.14211  0.79539
```

```
C   6.84914  0.69959  0.40002
C   7.02298  0.06573 -0.84136
C   5.91521 -0.12255 -1.68718
C   3.11186  1.48789  0.33629
C   1.89558  0.72904  0.26925
C   0.66180  1.45445  0.33159
C   0.65240  2.82609  0.66886
C   1.85041  3.51674  0.89384
C   3.06452  2.85744  0.67633
C   1.87446 -0.75212  0.29520
N   0.66678 -1.35339 -0.00278
C   0.57248 -2.71326  0.08798
C   1.62758 -3.52849  0.49237
C   2.84336 -2.92575  0.85186
C   2.95542 -1.53659  0.75369
Ru -0.94968 -0.15864 -0.44513
Br -2.15983 -1.92576 -1.77585
N  -2.63465  1.03526 -0.80701
C  -3.56947  1.05798  0.20502
C  -4.76963  1.78036  0.03914
C  -5.00845  2.47715 -1.14878
C  -4.04025  2.44761 -2.16906
C  -2.87102  1.71247 -1.95710
C  -3.16205  0.29713  1.38594
C  -1.88081 -0.35063  1.28974
C  -1.42446 -1.06130  2.42836
C  -2.20467 -1.14640  3.59341
C  -3.46579 -0.52020  3.66657
C  -3.93760  0.20156  2.56472
H   3.88200 -1.04618  1.05063
H   3.68555 -3.52191  1.21426
H   1.48210 -4.61069  0.54315
H  -0.40320 -3.12553 -0.17758
H  -0.30415  3.35559  0.73044
H   1.83328  4.57427  1.17439
H   4.00598  3.41306  0.72898
H  -2.08616  1.64578 -2.71661
H  -4.18470  2.97746 -3.11368
H  -5.93895  3.03728 -1.28072
H  -5.51061  1.78997  0.84266
H  -0.44815 -1.55561  2.40729
H  -1.82355 -1.70577  4.45599
H  -4.06869 -0.59201  4.57716
H   5.44405  1.62797  1.76799
H   3.78493  0.17477 -1.95902
H   7.70554  0.85053  1.06530
H   6.04434 -0.60348 -2.66235
H  -4.91350  0.69713  2.62250
H   8.01582 -0.27623 -1.15074
H  -0.45796  1.37309 -0.04707
```

53
17a'CH
SCF(BP86)= -1297.680783
G(298 K) = -1297.334239
SCF(PBE) = -3856.98067824
Low Freq. = 20.2920cm-1,
30.2231cm-1

```
C   4.59081  0.41673 -1.02929
C   4.36703  0.91666  0.27481
C   5.47593  1.09594  1.13194
```

C   6.77265 0.76915 0.70225
C   6.98222 0.26895 -0.59347
C   5.88705 0.09717 -1.45865
C   2.99758 1.33144 0.70853
C   1.85612 0.47978 0.66244
C   0.55109 1.05246 0.88056
C   0.42180 2.39929 1.30204
C   1.56004 3.20249 1.41862
C   2.82576 2.67631 1.10781
C   1.87520 -0.97318 0.39018
N   0.66853 -1.48387 -0.05148
C   0.56741 -2.81760 -0.30442
C   1.62576 -3.70533 -0.10592
C   2.84317 -3.20643 0.38877
C   2.96382 -1.83519 0.63540
Ru -0.86297 -0.09383 -0.25094
Br -1.74998 -1.08079 -2.39775
N -2.32591 1.36633 -0.38009
C -3.50304 1.10998 0.29253
C -4.58178 2.01322 0.20799
C -4.46394 3.17370 -0.56301
C -3.25896 3.42064 -1.24590
C -2.21955 2.49470 -1.12745
C -3.46385 -0.13510 1.06009
C -2.23383 -0.87036 0.97444
C -2.11775 -2.05375 1.74082
C -3.18459 -2.50941 2.53430
C -4.39496 -1.79021 2.59373
C -4.52982 -0.60508 1.86138
H   3.89518 -1.42207 1.02538
H   3.68548 -3.87653 0.58296
H   1.48760 -4.76731 -0.32441
H -0.40481 -3.14686 -0.67906
H -0.57171 2.79943 1.52406
H   1.46629 4.24813 1.72910
H   3.70675 3.32497 1.14092
H -1.26212 2.63542 -1.63764
H -3.12120 4.31264 -1.86201
H -5.29936 3.87639 -0.63394
H -5.50984 1.79839 0.74439
H -1.18694 -2.62964 1.72054
H -3.06952 -3.43303 3.11331
H -5.22259 -2.15044 3.21273
H   5.31500 1.48202 2.14421
H   3.74248 0.29240 -1.71070
H   7.61958 0.90658 1.38246
H   6.04341 -0.28071 -2.47439
H -5.46747 -0.04050 1.91610
H   7.99332 0.01764 -0.92968
H -0.28651 0.33007 1.35643

52
17a pi-Br
SCF(BP86)= -1284.03626657
G(298 K) = -1283.686250
SCF(PBE) = -1282.98319368
Low Freq. = 28.7714cm-1,
30.8926cm-1
C   0.16382 1.04813 2.32410
C -0.69588 0.61036 1.24504
C -0.90918 -0.84284 1.03696
C -0.15289 -1.73619 1.90051
C   0.70547 -1.26696 2.86973
C   0.85328 0.14066 3.09646
C -1.56870 1.59647 0.54164
C -2.32099 2.66573 1.03272
C -3.01805 3.46033 0.10234
C -2.93384 3.16243 -1.26790
C -2.12732 2.09702 -1.69534
N -1.44941 1.34795 -0.79694
Ru 0.07296 -0.05647 -0.73547
H -2.36999 2.86286 2.10686
H -3.62689 4.30095 0.44658
H -3.47621 3.75563 -2.00822
H -1.99557 1.84449 -2.75092
H   1.50278 0.49194 3.90403
H   1.24383 -1.97466 3.50775
H -0.33129 -2.81160 1.80185
N   1.71182 -1.35151 -0.57994
C   1.66478 -2.69467 -0.76982
H   0.67904 -3.11448 -0.97966
C   2.79660 -3.51146 -0.71447
H   2.69417 -4.58735 -0.87399
C   4.04094 -2.91785 -0.44521
C   4.10583 -1.53170 -0.27250
H   4.94804 -3.52550 -0.38147
H   5.06535 -1.04429 -0.08320
C   2.93482 -0.75467 -0.35722
C   2.85834 0.70425 -0.28325
C   1.54643 1.25685 -0.42631
C   1.41477 2.66421 -0.43957
H   0.43047 3.13031 -0.54263
C   2.54465 3.49437 -0.32307
H   2.41744 4.58234 -0.34369
C   3.82827 2.93922 -0.17127
H   4.70321 3.58877 -0.07174
C   3.98416 1.54746 -0.15646
C -2.17660 -1.39121 0.43450
C -3.43843 -0.80463 0.70223
C -2.14221 -2.56060 -0.36424
C -4.61163 -1.35163 0.16574
C -3.31734 -3.10029 -0.91087
C -4.55660 -2.49433 -0.65251
H -3.50369 0.06948 1.35535
H -1.19003 -3.06455 -0.54856
H -5.57603 -0.88843 0.39761
H -3.26101 -4.00130 -1.52967
H   4.98721 1.11847 -0.05984
H -5.47525 -2.91646 -1.07163
H   0.25216 2.12216 2.51220

59
TS(17a pi-18a pi)
SCF(BP86)= -1430.42502363
G(298 K) = -1430.037650
SCF(PBE) = -3989.62388417
Low Freq. = -82.9785cm-1,
12.9549cm-1
C -3.38416 -2.66331 -0.81380
C -2.60168 -1.55962 -0.41553
N -1.23983 -1.69806 -0.24493
C -0.66909 -2.91842 -0.42004

```
C  -1.40062 -4.04381 -0.80492
C  -2.78599 -3.91010 -1.01650
C  -3.07989 -0.21560 -0.10067
C  -2.06514  0.70438  0.32069
C  -2.48061  1.99200  0.73586
C  -3.83493  2.36306  0.70549
C  -4.82001  1.45751  0.25936
C  -4.44082  0.17093 -0.13718
Ru -0.20568 -0.00873  0.32129
N   2.30782 -1.64396  1.27041
C   3.17509 -2.07502  1.93395
C   4.25018 -2.61058  2.76401
N   0.78360  1.75932  0.77247
C   0.73052  2.42842 -0.41719
C   1.11612  3.76912 -0.52943
C   1.64191  4.40913  0.60799
C   1.75427  3.69109  1.81089
C   1.30633  2.36438  1.86609
C   0.10992  1.53760 -1.44034
C   0.69996  0.24228 -1.80637
C  -0.01696 -0.49355 -2.83064
C  -1.13892 -0.00369 -3.47146
C  -1.61438  1.30742 -3.18144
C  -0.99391  2.05765 -2.20102
C   2.17252 -0.07100 -1.70486
C   3.15375  0.94649 -1.63788
C   4.52352  0.63721 -1.65033
C   4.95204 -0.69587 -1.75007
C   3.99110 -1.71802 -1.83476
C   2.62541 -1.40929 -1.80778
Br -0.54959 -0.63937  2.77117
H   1.02455  4.28816 -1.48767
H   1.96642  5.45223  0.55305
H   2.16870  4.15639  2.70915
H   1.31881  1.76989  2.78381
H  -2.46179  1.72045 -3.73752
H  -1.62544 -0.60282 -4.24779
H   0.39966 -1.45074 -3.15733
H   0.40734 -2.96172 -0.22972
H  -0.89096 -5.00211 -0.93376
H  -3.38923 -4.76935 -1.32420
H  -4.46128 -2.53809 -0.95387
H  -1.73919  2.71859  1.08498
H  -4.12666  3.36824  1.03210
H  -5.87354  1.75360  0.23373
H   2.85018  1.99531 -1.60230
H   1.89832 -2.22531 -1.85588
H   5.25688  1.44928 -1.60253
H   4.30562 -2.76390 -1.91557
H  -5.20687 -0.54169 -0.46409
H   6.02011 -0.93543 -1.77056
H  -1.35610  3.06186 -1.96141
H   4.59889 -1.84152  3.47174
H   3.88930 -3.48048  3.33571
H   5.09763 -2.92442  2.13398

59
18a' pi
SCF(BP86)= -1430.43903595
G(298 K) = -1430.050455
SCF(PBE) = -3989.64026723
Low Freq. = 22.4122cm-1,
36.9674cm-1
C  -3.49397 -2.51671 -0.73397
C  -2.64387 -1.43367 -0.42339
N  -1.35477 -1.66605  0.01385
C  -0.92921 -2.94917  0.15518
C  -1.73267 -4.05326 -0.13496
C  -3.04382 -3.83168 -0.59414
C  -2.99804 -0.01477 -0.49070
C  -1.95243  0.89228 -0.12454
C  -2.28501  2.26855 -0.09770
C  -3.56763  2.72830 -0.43939
C  -4.57444  1.81841 -0.82283
C  -4.28871  0.44909 -0.84318
Ru -0.19642  0.00662  0.41759
N   1.36929 -1.04117  1.39535
C   2.16204 -1.56367  2.08640
C   3.15640 -2.21785  2.92623
N   0.86261  1.77730  0.60240
C   1.06896  2.14539 -0.69550
C   1.58854  3.40028 -1.03283
C   1.97281  4.26689  0.00709
C   1.81633  3.85099  1.33960
C   1.24768  2.59790  1.60813
C   0.54361  1.07221 -1.59375
C   1.10338 -0.28149 -1.60076
C   0.46430 -1.20938 -2.51008
C  -0.52516 -0.83505 -3.40120
C  -0.92605  0.52773 -3.49121
C  -0.39749  1.45402 -2.61230
C   2.54103 -0.59742 -1.28229
C   3.55336  0.38592 -1.36805
C   4.90496  0.06060 -1.16231
C   5.28531 -1.26123 -0.88582
C   4.29403 -2.25711 -0.81933
C   2.94564 -1.92878 -1.01321
Br -1.16942  0.11562  2.81287
H   1.70419  3.68098 -2.08358
H   2.39425  5.25000 -0.22149
H   2.11427  4.49515  2.17116
H   1.05227  2.23961  2.62256
H  -1.64961  0.83962 -4.25087
H  -0.94933 -1.57747 -4.08475
H   0.85204 -2.23151 -2.55200
H   0.09452 -3.06665  0.51811
H  -1.33325 -5.06200 -0.00164
H  -3.70280 -4.67140 -0.83426
H  -4.51087 -2.31828 -1.08265
H  -1.52592  3.00420  0.19461
H  -3.78745  3.80239 -0.40836
H  -5.57360  2.17668 -1.09157
H   3.29156  1.41563 -1.62196
H   2.19174 -2.71865 -0.93785
H   5.66317  0.84716 -1.24000
H   4.57143 -3.29683 -0.61496
H  -5.07789 -0.25928 -1.12103
H   6.33905 -1.51667 -0.73489
H  -0.71550  2.49977 -2.65291
H   3.50370 -1.53112  3.71535
H   2.72669 -3.11489  3.40135
H   4.02053 -2.51891  2.31098
```

59
18a pi
SCF(BP86)= -1430.44883701
G(298 K) = -1430.059781
SCF(PBE) = -3989.64528652
Low Freq. = 19.3631cm-1,
26.4845cm-1

```
C  -4.06520 -2.32747 -0.50810
C  -3.12561 -1.28090 -0.40630
N  -1.86499 -1.52488  0.10596
C  -1.54594 -2.78984  0.48332
C  -2.43915 -3.86097  0.39632
C  -3.72978 -3.62347 -0.10515
C  -3.34438  0.10667 -0.83201
C  -2.22797  0.98874 -0.65464
C  -2.39656  2.32227 -1.09808
C  -3.60439  2.77187 -1.66243
C  -4.69280  1.89103 -1.81141
C  -4.55895  0.55921 -1.39869
Ru -0.60963  0.13442  0.25337
N   0.77697 -0.91207  1.44903
C   1.43859 -1.51975  2.20812
C   2.27095 -2.26343  3.14518
N   0.57171  1.83755  0.15904
C   1.52861  1.55925 -0.77697
C   2.50299  2.49193 -1.14484
C   2.49296  3.75263 -0.52142
C   1.51069  4.03050  0.44361
C   0.55936  3.05254  0.76183
C   1.28948  0.18169 -1.31106
C   2.29267 -0.85842 -1.33974
C   1.95737 -2.07214 -1.95438
C   0.70113 -2.28626 -2.57618
C  -0.22660 -1.25596 -2.64272
C   0.04644 -0.00014 -2.01900
C   3.66658 -0.67997 -0.78755
C   4.77226 -1.19608 -1.50779
C   6.07924 -1.07972 -1.01225
C   6.31565 -0.43889  0.21547
C   5.23153  0.08716  0.93834
C   3.92347 -0.02736  0.44269
Br -1.73947  0.79554  2.47059
H   3.25126  2.23168 -1.89779
H   3.24055  4.50568 -0.78646
H   1.47395  4.99886  0.94990
H  -0.22651  3.20631  1.50653
H  -1.16284 -1.38021 -3.19604
H   0.49011 -3.25402 -3.04196
H   2.69107 -2.88476 -1.94727
H  -0.53484 -2.92466  0.87191
H  -2.12167 -4.85569  0.71986
H  -4.45993 -4.43449 -0.18366
H  -5.06055 -2.11842 -0.90858
H  -1.57075  3.03662 -0.99377
H  -3.69886  3.81527 -1.98701
H  -5.63433  2.23948 -2.24831
H   4.60320 -1.67307 -2.47889
H   3.09200  0.37489  1.02607
H   6.91550 -1.48269 -1.59306
H   5.40217  0.58854  1.89685
H  -5.40560 -0.12591 -1.52271
H   7.33526 -0.34541  0.60295
H  -0.50472  0.88074 -2.35788
H   2.04228 -1.96235  4.18063
H   2.09086 -3.34632  3.04368
H   3.33566 -2.06078  2.94268
```

59
TS(18a pi-18a'CH)
SCF(BP86)= -1430.44634193
G(298 K) = -1430.057255
SCF(PBE) = -3989.63951969
Low Freq. = -73.5671cm-1,
25.4777cm-1

```
C  -4.47058 -1.95870 -0.74162
C  -3.42129 -1.03323 -0.56597
N  -2.24664 -1.41968  0.05453
C  -2.13308 -2.70059  0.49669
C  -3.14348 -3.65316  0.34542
C  -4.33950 -3.27469 -0.28862
C  -3.43105  0.36707 -1.00135
C  -2.23619  1.10858 -0.70122
C  -2.21587  2.45986 -1.13405
C  -3.30218  3.04747 -1.80721
C  -4.46333  2.29848 -2.07988
C  -4.52354  0.95827 -1.67685
Ru -0.79447  0.05359  0.27351
N   0.53422 -1.19487  1.31019
C   1.22972 -1.90714  1.93992
C   2.09726 -2.79091  2.70959
N   0.59644  1.60427  0.25811
C   1.66073  1.40692 -0.58990
C   2.65385  2.38122 -0.77180
C   2.57316  3.58994 -0.06639
C   1.49412  3.78223  0.81253
C   0.53088  2.77848  0.94640
C   1.61024  0.10536 -1.31215
C   2.70573 -0.80275 -1.39141
C   2.50923 -2.01521 -2.09136
C   1.27928 -2.35734 -2.67903
C   0.20578 -1.46247 -2.60337
C   0.37585 -0.23054 -1.93815
C   4.03474 -0.55851 -0.75515
C   5.21661 -0.76935 -1.50269
C   6.47959 -0.59154 -0.91653
C   6.58531 -0.20547  0.42994
C   5.41841  0.00311  1.18541
C   4.15519 -0.16856  0.59939
Br -1.85871  0.70104  2.48965
H   3.47530  2.18302 -1.46484
H   3.33380  4.36402 -0.20073
H   1.38888  4.70464  1.39020
H  -0.32723  2.87545  1.61688
H  -0.75372 -1.69388 -3.07541
H   1.17133 -3.31615 -3.19497
H   3.34186 -2.72477 -2.14213
H  -1.18841 -2.94686  0.98584
H  -2.98856 -4.66757  0.72226
H  -5.15464 -3.99170 -0.42439
H  -5.39192 -1.63739 -1.23450
H  -1.33092  3.07771 -0.93893
```

H -3.24410 4.09654 -2.12236
H -5.31022 2.75498 -2.60259
H 5.14113 -1.05768 -2.55671
H 3.25301 -0.01297 1.19840
H 7.38188 -0.75154 -1.51575
H 5.49032 0.29684 2.23777
H -5.42614 0.37506 -1.89412
H 7.56992 -0.06806 0.88829
H -0.39863 0.54225 -2.04020
H 1.92481 -2.65191 3.78955
H 1.89732 -3.84459 2.45345
H 3.15483 -2.56900 2.48944

59
18a'CH
SCF(BP86)= -1430.45606199
G(298 K) = -1430.067701
SCF(PBE) = -3989.64810337
Low Freq. = 19.1248cm-1,
20.8263cm-1
C -4.63855 -1.55007 -1.27413
C -3.51977 -0.75720 -0.94445
N -2.47804 -1.29443 -0.21318
C -2.55050 -2.59407 0.17722
C -3.63545 -3.41979 -0.12596
C -4.70375 -2.88542 -0.86675
C -3.32114 0.64562 -1.32472
C -2.09120 1.23571 -0.88054
C -1.85322 2.57654 -1.26708
C -2.78495 3.30580 -2.02722
C -3.99370 2.71082 -2.43712
C -4.25776 1.38081 -2.08790
Ru -0.91763 0.00311 0.26131
N 0.05178 -1.35149 1.57916
C 0.59226 -2.08009 2.32458
C 1.25053 -2.98918 3.25442
N 0.63528 1.37304 0.51414
C 1.82768 1.10635 -0.13357
C 2.86018 2.06769 -0.14604
C 2.71203 3.27350 0.54529
C 1.51868 3.50603 1.24907
C 0.50924 2.54354 1.19806
C 1.86605 -0.18099 -0.85978
C 3.04520 -0.88874 -1.23491
C 2.91220 -2.06486 -2.00857
C 1.65667 -2.57396 -2.37689
C 0.49187 -1.92280 -1.95406
C 0.58932 -0.74854 -1.17713
C 4.42690 -0.52018 -0.79580
C 5.45251 -0.32973 -1.74889
C 6.76638 -0.04709 -1.34021
C 7.07767 0.03819 0.02675
C 6.06707 -0.16123 0.98409
C 4.75343 -0.43720 0.57776
Br -2.27626 0.73067 2.35191
H 3.77156 1.86213 -0.70903
H 3.51156 4.01974 0.53099
H 1.35588 4.42695 1.81524
H -0.44982 2.67751 1.70573
H -0.49540 -2.31295 -2.21887
H 1.59258 -3.48777 -2.97639
H 3.82164 -2.60294 -2.29447
H -1.69810 -2.96005 0.75346
H -3.63581 -4.45768 0.21676
H -5.57220 -3.50005 -1.12156
H -5.45695 -1.11166 -1.85091
H -0.92241 3.07115 -0.96643
H -2.56728 4.34435 -2.30382
H -4.72014 3.27875 -3.02736
H 5.21175 -0.39165 -2.81562
H 3.97164 -0.60028 1.32689
H 7.54674 0.10637 -2.09281
H 6.30390 -0.10777 2.05184
H -5.19647 0.91830 -2.41364
H 8.10218 0.25561 0.34556
H -0.31467 0.01338 -1.37344
H 1.57588 -2.44281 4.15461
H 0.55709 -3.79077 3.55707
H 2.13318 -3.44519 2.77758

59
TS(18a pi-18aCH)
SCF(BP86)= -1430.434091
G(298 K) = -1430.046820
SCF(PBE) = -3989.62361187
Low Freq. = -446.3036cm-1,
15.6210cm-1
C -4.68723 -0.23956 1.92534
C -3.49381 -0.40100 1.18987
N -2.70494 0.69529 0.90869
C -3.08927 1.92142 1.34450
C -4.25898 2.13200 2.07876
C -5.07492 1.02599 2.37356
C -2.96570 -1.66713 0.67160
C -1.73429 -1.56507 -0.06138
C -1.19507 -2.77967 -0.55515
C -1.83061 -4.01634 -0.34628
C -3.04048 -4.08761 0.37215
C -3.60459 -2.91174 0.88096
Ru -0.98692 0.31593 -0.24193
N -0.34081 2.32839 -0.35389
C 0.03143 3.44113 -0.42970
C 0.48867 4.82221 -0.52040
N 0.67082 -0.29843 -1.31649
C 1.85078 -0.56595 -0.65016
C 2.89709 -1.23655 -1.32347
C 2.79886 -1.54882 -2.68099
C 1.63096 -1.18253 -3.36688
C 0.59793 -0.58212 -2.64949
C 1.90357 -0.26009 0.80134
C 3.13341 -0.25488 1.54490
C 3.09942 -0.28340 2.95625
C 1.89421 -0.25043 3.66469
C 0.69645 -0.09405 2.95457
C 0.70117 -0.07735 1.54642
C 4.48833 -0.10243 0.92906
C 5.54679 -0.97071 1.28135
C 6.83722 -0.77372 0.76323
C 7.09392 0.30039 -0.10462
C 6.05147 1.17864 -0.45111
C 4.76107 0.98014 0.05949
Br -2.51788 0.74947 -2.25712

```
H   3.78849 -1.52463 -0.76748
H   3.61493 -2.07225 -3.18712
H   1.49940 -1.38206 -4.43370
H  -0.35404 -0.32275 -3.11979
H  -0.24978  0.03552  3.48950
H   1.88935 -0.28328  4.75839
H   4.05076 -0.28764  3.49735
H  -2.42132  2.74532  1.08259
H  -4.51886  3.14220  2.40489
H  -6.00074  1.15026  2.94324
H  -5.30902 -1.11233  2.14044
H  -0.25550 -2.76529 -1.11976
H  -1.38021 -4.93318 -0.74567
H  -3.53531 -5.05075  0.53385
H   5.34929 -1.81433  1.95128
H   3.95498  1.67283 -0.20419
H   7.64228 -1.46272  1.03888
H   6.24601  2.02627 -1.11644
H  -4.54355 -2.96834  1.44374
H   8.10044  0.45551 -0.50618
H  -0.38310  0.28835  1.33370
H   1.12877  4.95470 -1.40794
H  -0.37382  5.50382 -0.60157
H   1.06875  5.09100  0.37755

59
18aCH
SCF(BP86)= -1430.454016
G(298 K) = -1430.065729
SCF(PBE) = -3989.64722985
Low Freq. = 18.5575cm-1,
20.3634cm-1
C  -3.73398 -2.92244  0.49377
C  -2.71520 -2.01448  0.13759
N  -2.66552 -0.76260  0.72182
C  -3.61171 -0.42569  1.63758
C  -4.63680 -1.29123  2.02551
C  -4.69962 -2.56760  1.43976
C  -1.64259 -2.26534 -0.83053
C  -0.70989 -1.19328 -1.00148
C   0.32807 -1.38752 -1.94044
C   0.44415 -2.58054 -2.67639
C  -0.48141 -3.62619 -2.48936
C  -1.52419 -3.46687 -1.56859
Ru -1.10464  0.47242  0.11069
N  -1.79846  2.20520  1.12759
C  -2.19111  3.19457  1.62505
C  -2.69212  4.41518  2.24362
N   0.59033  1.57934 -0.40864
C   1.79996  1.20985  0.15056
C   2.92566  2.05182  0.02938
C   2.84573  3.23991 -0.70271
C   1.62509  3.57694 -1.31042
C   0.52838  2.73309 -1.12914
C   1.76508 -0.04797  0.92209
C   2.89193 -0.87066  1.21401
C   2.70905 -2.01586  2.02180
C   1.44271 -2.38815  2.49952
C   0.32067 -1.62352  2.16275
C   0.46731 -0.46696  1.36332
C   4.26181 -0.65479  0.65153
C   4.48124 -0.67531 -0.74519
C   5.77633 -0.54083 -1.26642
C   6.87461 -0.38267 -0.40251
C   6.66950 -0.36852  0.98696
C   5.37364 -0.50950  1.51092
Br -2.55468  1.24252 -1.90158
H   3.85616  1.76935  0.52340
H   3.71834  3.89341 -0.79142
H   1.50999  4.48963 -1.90118
H  -0.45255  2.95428 -1.55878
H  -0.67434 -1.90730  2.51803
H   1.33386 -3.28415  3.11917
H   3.58051 -2.64003  2.24351
H  -3.52256  0.57863  2.05799
H  -5.36786 -0.96301  2.76879
H  -5.48904 -3.27276  1.71629
H  -3.76180 -3.90903  0.02403
H   1.05841 -0.58718 -2.11004
H   1.25875 -2.69752 -3.40115
H  -0.39134 -4.55521 -3.06173
H   3.62923 -0.81064 -1.41950
H   5.21561 -0.49518  2.59469
H   5.92950 -0.56606 -2.35038
H   7.51907 -0.24812  1.66721
H  -2.24741 -4.27919 -1.43220
H   7.88468 -0.27556 -0.81097
H  -0.33634  0.36556  1.67489
H  -2.12021  5.28677  1.88596
H  -3.75455  4.55900  1.98770
H  -2.59630  4.35333  3.33990

60
26a pi
SCF(BP86)= -1526.29947525
G(298 K) = -1525.909369
SCF(PBE) = -4085.46103662
Low Freq. = 20.9422cm-1,
35.6335cm-1
C  -3.54852 -2.32559 -1.12639
C  -2.69364 -1.31245 -0.64291
N  -1.40611 -1.61081 -0.24069
C  -0.98704 -2.90519 -0.29134
C  -1.80122 -3.94192 -0.75898
C  -3.10581 -3.64889 -1.19384
C  -3.04785  0.09745 -0.48685
C  -1.99359  0.92927  0.00719
C  -2.32090  2.28684  0.25561
C  -3.60419  2.79863  0.00402
C  -4.62047  1.96452 -0.50757
C  -4.34050  0.61467 -0.74520
Ru -0.22405  0.00006  0.37162
O   1.57129 -0.93746  1.38579
N   0.79724  1.74818  0.74759
C   0.88717  2.28661 -0.50585
C   1.29527  3.60812 -0.71381
C   1.69263  4.36895  0.40345
C   1.66460  3.78104  1.67919
C   1.20453  2.46317  1.82242
C   0.37611  1.27336 -1.47304
C   1.02862 -0.04464 -1.57166
C   0.46290 -0.93666 -2.57217
```

C -0.53011 -0.55371 -3.44997
C -1.01559 0.79162 -3.44326
C -0.56305 1.68025 -2.49100
C   2.51277 -0.23349 -1.36864
C   3.41761 0.82977 -1.59289
C   4.80811 0.63131 -1.52994
C   5.33099 -0.64291 -1.26294
C   4.44378 -1.71559 -1.05870
C   3.05763 -1.51383 -1.11067
Br -1.15370 -0.23539 2.80684
H   1.31658 4.02380 -1.72553
H   2.02661 5.40298 0.27650
H   1.97849 4.34043 2.56490
H   1.10724 1.96911 2.79264
H -1.74216 1.11392 -4.19646
H -0.90087 -1.26306 -4.19741
H   0.91494 -1.92910 -2.67199
H   0.02721 -3.08033 0.10043
H -1.41044 -4.96362 -0.77525
H -3.76631 -4.43690 -1.56941
H -4.56161 -2.06584 -1.44582
H -1.55703 2.96452 0.65480
H -3.81828 3.85504 0.20836
H -5.62095 2.36395 -0.70458
H   3.03560 1.82322 -1.84194
H   2.38788 -2.35289 -0.89982
H   5.48079 1.47716 -1.71015
H   4.83412 -2.71762 -0.84867
H -5.13607 -0.04076 -1.11937
H   6.41370 -0.80183 -1.22045
H -0.93630 2.70887 -2.46920
C   1.98747 -2.11239 1.71991
O   1.58228 -3.22569 1.28082
C   3.14597 -2.10622 2.73717
H   2.94794 -1.39961 3.55994
H   3.31864 -3.11337 3.14718
H   4.06921 -1.77096 2.23160

60
TS(26a pi-27a pi)
SCF(BP86)= -1526.28663743
G(298 K) = -1525.895079
SCF(PBE) = -4085.44072749
Low Freq. = -43.1395cm-1,
31.3742cm-1
C -3.34938 -1.69944 -2.13366
C -2.48868 -0.88471 -1.36976
N -1.30749 -1.39051 -0.86565
C -0.99691 -2.69424 -1.09990
C -1.82109 -3.54130 -1.84698
C -3.01796 -3.03420 -2.38139
C -2.73194 0.50993 -1.00629
C -1.68474 1.13604 -0.26154
C -1.90857 2.46356 0.17810
C -3.10723 3.13846 -0.10560
C -4.12455 2.50851 -0.85094
C -3.93645 1.19470 -1.29390
Ru -0.09152 -0.04292 0.17858
O   0.87031 -1.37790 1.58032
N   1.01009 1.48152 1.02906
C   1.29919 2.21695 -0.08737
C   1.83816 3.50352 -0.00132
C   2.13938 4.01261 1.27756
C   1.89503 3.22224 2.41292
C   1.31962 1.95116 2.25814
C   0.84109 1.41728 -1.25874
C   1.39441 0.04651 -1.41913
C   1.00939 -0.63016 -2.66100
C   0.28248 -0.01417 -3.65158
C -0.10818 1.36380 -3.52550
C   0.17644 2.06003 -2.37503
C   2.80562 -0.28594 -0.97904
C   3.82510 0.69016 -1.07448
C   5.16174 0.37153 -0.78161
C   5.51039 -0.93490 -0.40337
C   4.50806 -1.91810 -0.32555
C   3.17193 -1.60021 -0.61275
Br -2.39063 -0.42332 2.93182
H   2.02659 4.08580 -0.90771
H   2.56647 5.01410 1.38367
H   2.13105 3.58731 3.41598
H   1.06868 1.30710 3.10495
H -0.62305 1.85836 -4.35553
H   0.04513 -0.55893 -4.57162
H   1.39414 -1.64404 -2.81524
H -0.06928 -3.04344 -0.62853
H -1.52168 -4.58167 -2.00120
H -3.68309 -3.66899 -2.97496
H -4.27756 -1.27879 -2.52964
H -1.14025 2.98675 0.75767
H -3.24951 4.16460 0.25386
H -5.05751 3.03651 -1.07342
H   3.57582 1.70474 -1.39914
H   2.39695 -2.36714 -0.50445
H   5.93166 1.14658 -0.86261
H   4.76661 -2.94193 -0.03292
H -4.73812 0.69478 -1.84952
H   6.55197 -1.18589 -0.17649
H -0.11073 3.11124 -2.27327
C   1.08314 -2.65136 1.77373
O   1.15378 -3.53757 0.88621
C   1.28430 -3.03329 3.24586
H   0.36491 -2.81599 3.81663
H   1.52879 -4.10122 3.34487
H   2.09133 -2.42821 3.69206

59
27a pi
SCF(BP86)= -1512.72844718
G(298 K) = -1512.334616
SCF(PBE) = -1511.52245311
Low Freq. = 16.3049cm-1,
27.7709cm-1
C -3.92845 -1.84800 -0.40846
C -2.87383 -0.95248 -0.13370
N -1.60507 -1.43143 0.12603
C -1.39934 -2.77463 0.16045
C -2.41129 -3.70155 -0.09693
C -3.70147 -3.22704 -0.39790
C -2.96958 0.50422 -0.04787
C -1.73856 1.18008 0.24901

```
C  -1.80470  2.58331  0.43669
C  -3.01516  3.28494  0.31237
C  -4.20944  2.60610 -0.00627
C  -4.18436  1.21827 -0.17994
Ru -0.14651 -0.01578  0.49916
O   1.09309 -1.55299  1.84862
N   1.21201  1.51432  0.75198
C   1.36489  1.96011 -0.53230
C   2.03454  3.15285 -0.82330
C   2.62168  3.86583  0.23968
C   2.51420  3.36423  1.54753
C   1.79294  2.18344  1.77578
C   0.62004  1.03713 -1.43603
C   0.93646 -0.40094 -1.46509
C   0.17686 -1.18141 -2.42681
C  -0.74022 -0.62316 -3.29538
C  -0.94305  0.78913 -3.31950
C  -0.26754  1.59360 -2.42318
C   2.29581 -0.95911 -1.11962
C   3.47299 -0.18360 -1.23700
C   4.73801 -0.74300 -0.99119
C   4.86169 -2.09701 -0.64196
C   3.70318 -2.88773 -0.54487
C   2.44146 -2.32545 -0.77963
H   2.10738  3.50379 -1.85657
H   3.16208  4.79702  0.04673
H   2.96853  3.88825  2.39255
H   1.63582  1.77554  2.77795
H  -1.62529  1.23247 -4.05177
H  -1.27193 -1.26232 -4.00791
H   0.40225 -2.24991 -2.50338
H  -0.38294 -3.08323  0.41864
H  -2.18741 -4.77080 -0.06003
H  -4.51775 -3.92359 -0.61109
H  -4.92599 -1.45532 -0.62329
H  -0.89507  3.14448  0.67853
H  -3.02981  4.37114  0.46242
H  -5.15044  3.15659 -0.10619
H   3.40582  0.86317 -1.54409
H   1.55118 -2.95413 -0.68591
H   5.63095 -0.11676 -1.09244
H   3.78115 -3.94790 -0.28108
H  -5.11631  0.68675 -0.40496
H   5.84821 -2.53436 -0.45707
H  -0.41644  2.67782 -2.43028
C   0.37234 -1.13548  2.81074
O  -0.52963 -0.22942  2.57392
C   0.55200 -1.63817  4.22620
H   1.46973 -1.20080  4.65602
H  -0.30210 -1.35885  4.85967
H   0.67593 -2.73229  4.22501

58
22aCH
SCF(BP86)= -1416.82519680
G(298 K) = -1416.436479
SCF(PBE) = -1415.65713088
Low Freq. = 16.9732cm-1,
21.9817cm-1
C  -2.49656 -3.11106 -1.76895
C  -2.34098 -1.93582 -1.00161
C  -1.17092 -1.12366 -1.14191
C  -0.16845 -1.55173 -2.03783
C  -0.32852 -2.72431 -2.79973
C  -1.49175 -3.50341 -2.66435
C  -3.34415 -1.45250 -0.04256
N  -3.04256 -0.22228  0.50268
C  -3.88149  0.32032  1.42217
C  -5.05237 -0.31617  1.84228
C  -5.37604 -1.56544  1.28804
C  -4.51498 -2.13244  0.34229
Ru -1.27920  0.67252 -0.20473
N  -1.88051  2.63688  0.29249
C  -2.25210  3.73621  0.46786
C  -2.71817  5.09487  0.69525
C   0.10424 -0.31276  1.01704
C   1.47660 -0.02187  0.68994
C   2.50985 -0.80847  1.27916
C   2.16402 -1.78936  2.23391
C   0.82738 -2.04626  2.57348
C  -0.20214 -1.33046  1.95249
C   3.95521 -0.73105  0.89434
C   4.93169 -0.35498  1.84252
C   6.29386 -0.34580  1.49822
C   6.69871 -0.72462  0.20768
C   5.73471 -1.11514 -0.73857
C   4.37364 -1.11874 -0.39947
C   1.66610  1.07576 -0.27384
N   0.49640  1.47291 -0.89150
C   0.52933  2.44632 -1.83797
C   1.70554  3.10472 -2.19957
C   2.89244  2.76774 -1.52849
C   2.86874  1.75353 -0.56591
H   3.77923  1.48278 -0.03209
H   3.82744  3.28962 -1.75079
H   1.67761  3.88013 -2.96886
H  -0.43312  2.69989 -2.29205
H   2.96596 -2.38221  2.68433
H   0.58773 -2.82485  3.30468
H  -1.24718 -1.53811  2.19808
H  -3.58528  1.29460  1.81775
H  -5.69033  0.16408  2.58802
H  -6.28454 -2.09305  1.59224
H  -4.74273 -3.10753 -0.09511
H   0.74856 -0.96347 -2.15057
H   0.45812 -3.03286 -3.49712
H  -1.61597 -4.41589 -3.25587
H  -3.40310 -3.71923 -1.67954
H  -0.69475  0.70247  1.32557
H   4.61749 -0.05920  2.84908
H   7.03863 -0.04360  2.24169
H   7.76040 -0.72007 -0.05921
H   6.04377 -1.42318 -1.74280
H   3.62519 -1.42912 -1.13613
H  -2.00584  5.81814  0.26699
H  -3.70302  5.23569  0.22118
H  -2.81358  5.28109  1.77732

64
23aCH
SCF(BP86)= -1549.62918580
G(298 K) = -1549.195842
```

SCF(PBE) = -1548.35180330
Low Freq. = 22.4471cm-1,
24.7601cm-1

C	6.95802	0.07385	0.16338
C	6.69000	-0.11180	-1.20284
C	5.38868	-0.42089	-1.63122
C	4.33351	-0.53702	-0.69875
C	4.61570	-0.35364	0.67462
C	5.91742	-0.05198	1.10062
C	2.96448	-0.92814	-1.15572
C	1.78101	-0.18750	-0.87287
C	0.52045	-0.76066	-1.21625
C	0.42566	-1.98326	-1.90514
C	1.59777	-2.67649	-2.23484
C	2.84207	-2.15240	-1.85464
C	1.74064	1.13617	-0.20857
C	2.76738	2.09829	-0.29179
C	2.63190	3.33197	0.35170
C	1.45892	3.58908	1.07954
C	0.45405	2.62093	1.09836
N	0.56815	1.42422	0.46044
Ru	-0.98693	0.03954	0.33037
C	-2.18293	1.19287	-0.87987
C	-3.40727	0.56336	-1.27790
C	-4.35214	1.24054	-2.08298
C	-4.10068	2.54887	-2.51376
C	-2.89933	3.18118	-2.14313
C	-1.95881	2.51005	-1.34027
C	-3.59337	-0.81671	-0.81254
N	-2.55199	-1.30247	-0.04913
C	-2.60818	-2.57446	0.42175
C	-3.68420	-3.42759	0.16662
C	-4.75449	-2.94740	-0.60676
C	-4.70363	-1.63903	-1.09513
N	-1.99246	0.70887	1.89331
H	3.66388	1.87015	-0.87001
H	3.42605	4.08050	0.28310
H	1.30678	4.53253	1.60979
H	-0.49028	2.78759	1.62046
H	-0.55682	-2.37441	-2.18424
H	1.54641	-3.62803	-2.77266
H	3.75515	-2.71582	-2.07183
H	-1.75267	-2.89512	1.01982
H	-3.67611	-4.44294	0.57033
H	-5.61603	-3.58486	-0.82600
H	-5.52468	-1.24671	-1.70010
H	-1.03361	3.03210	-1.07137
H	-2.69327	4.20302	-2.48227
H	-4.83338	3.07152	-3.13678
H	5.18136	-0.56051	-2.69757
H	3.81081	-0.46159	1.40898
H	7.49440	-0.01634	-1.93938
H	6.12115	0.07926	2.16835
H	-5.28608	0.74986	-2.37838
H	7.97296	0.31174	0.49756
H	-0.38122	-0.04689	-1.38101
C	-2.60396	1.07950	2.82418
C	-3.35460	1.54597	3.98143
H	-3.69734	0.68822	4.58293
H	-2.72060	2.19117	4.61133
H	-4.23415	2.12366	3.65369
N	0.05103	-1.25448	1.67034
C	0.60877	-1.93566	2.44610
C	1.29245	-2.78919	3.40834
H	0.56686	-3.20088	4.12845
H	1.78959	-3.62303	2.88696
H	2.05104	-2.20972	3.95881

70
TS(23aCH-2c2-ppy-Ph)
SCF(BP86)= -1682.36804787
G(298 K) = -1681.894435
SCF(PBE) = -1680.98647538
Low Freq. = -59.4216cm-1,
9.7888cm-1

C	3.58780	2.94959	0.29620
C	2.65084	1.97532	-0.10173
N	2.63998	0.73998	0.51127
C	3.54182	0.47225	1.48853
C	4.49000	1.40478	1.91744
C	4.51158	2.66961	1.30774
C	1.63626	2.13592	-1.15007
C	0.78347	1.00202	-1.36122
C	-0.15546	1.10496	-2.41499
C	-0.28046	2.27508	-3.18819
C	0.54485	3.38628	-2.93751
C	1.50630	3.31276	-1.92094
Ru	1.17314	-0.58371	-0.14379
N	2.40699	-1.23630	-1.43089
C	3.22665	-1.60226	-2.19269
C	4.22955	-2.07914	-3.13619
N	-0.50046	-1.72558	-0.89058
C	-1.75582	-1.76633	-0.32510
C	-2.83655	-2.37497	-0.99819
C	-2.65876	-2.98512	-2.24138
C	-1.37006	-2.98033	-2.79459
C	-0.34173	-2.34586	-2.09915
C	-1.99449	-1.27633	1.07029
C	-3.03837	-0.35955	1.39850
C	-3.31054	-0.11445	2.76431
C	-2.59033	-0.74506	3.78794
C	-1.57076	-1.65207	3.46188
C	-1.28592	-1.91393	2.11440
C	-3.85766	0.35819	0.37897
C	-3.25790	1.04090	-0.70279
C	-4.04103	1.74904	-1.62647
C	-5.43926	1.78672	-1.48963
C	-6.04850	1.11335	-0.41707
C	-5.26554	0.40887	0.51049
N	1.78645	-2.16258	1.15246
C	2.23400	-3.05464	1.77374
N	0.10337	1.79841	2.01114
C	-0.10229	2.86463	2.45691
C	-0.35603	4.19312	3.00654
H	-3.81370	-2.37577	-0.50910
H	-3.49717	-3.46381	-2.75514
H	-1.15282	-3.45229	-3.75623
H	0.66339	-2.30730	-2.51926
H	-4.09603	0.60479	3.01820
H	-2.82801	-0.52927	4.83450
H	-1.01145	-2.16861	4.24814
H	4.06592	-1.62982	-4.12963

H   5.24061 -1.80819 -2.78938
H   4.17287 -3.17624 -3.23162
H   3.48187 -0.52463 1.92919
H   5.19214 1.13528 2.71014
H   5.23898 3.42653 1.61555
H   3.58709 3.92796 -0.19058
H -0.81014 0.25778 -2.64665
H -1.02159 2.31635 -3.99501
H   0.44847 4.29673 -3.53753
H   2.15984 4.17348 -1.74006
H -0.52916 -2.65673 1.85401
H   0.37634 4.42199 3.79698
H -0.27191 4.95234 2.21266
H -1.36938 4.23795 3.43612
H -5.74758 -0.12476 1.33672
H -7.13738 1.13272 -0.30287
H -6.04967 2.33831 -2.21201
H -3.55255 2.27966 -2.45065
H -2.16862 1.03656 -0.80931
C   2.77983 -4.15524 2.55627
H   2.51083 -5.11960 2.09545
H   3.87820 -4.07841 2.60571
H   2.37539 -4.12595 3.58119

70
2c2-ppy-Ph
SCF(BP86)= -1682.40563452
G(298 K) = -1681.930055
SCF(PBE) = -1681.02516996
Low Freq. = 8.4436cm-1, 13.1935cm-1
C   4.18010 -1.60557 -2.08918
C   3.07971 -1.31141 -1.25689
N   2.74667 0.00121 -0.99723
C   3.47639 0.99806 -1.55723
C   4.57273 0.75692 -2.38897
C   4.93244 -0.57419 -2.65714
C   2.20305 -2.29685 -0.61456
C   1.13747 -1.75403 0.17697
C   0.26809 -2.69030 0.78480
C   0.44198 -4.07862 0.63254
C   1.50075 -4.58538 -0.14365
C   2.37907 -3.69210 -0.76800
Ru 1.10528 0.29337 0.26171
N   2.40265 0.27061 1.78387
C   3.25047 0.28616 2.59668
C   4.29425 0.30824 3.61201
N -0.59074 0.33307 1.64269
C -1.88188 0.75724 1.42765
C -2.88672 0.57564 2.40339
C -2.60136 -0.01606 3.63461
C -1.28123 -0.42597 3.86536
C -0.33388 -0.24273 2.85921
C -2.28750 1.51319 0.19939
C -3.28463 1.01776 -0.69119
C -3.75148 1.87280 -1.71767
C -3.26573 3.17924 -1.86687
C -2.29838 3.66665 -0.97435
C -1.82531 2.83719 0.05228
C -3.85373 -0.35818 -0.59495
C -3.02734 -1.49289 -0.42291
C -3.57961 -2.78178 -0.37321
C -4.96798 -2.96438 -0.49132
C -5.80096 -1.84555 -0.66262
C -5.24955 -0.55625 -0.71642
N   1.33449 2.41158 0.29955
C   1.60618 3.55195 0.38032
N   0.01051 0.30622 -1.40566
C -0.46362 0.26637 -2.47860
C -1.07071 0.22441 -3.80139
H -3.89486 0.92900 2.17256
H -3.38106 -0.14538 4.39059
H -0.97621 -0.89389 4.80496
H   0.68947 -0.58394 3.00729
H -4.50235 1.49020 -2.41713
H -3.64663 3.81380 -2.67346
H -1.92603 4.69202 -1.06342
H   4.37101 -0.67653 4.10122
H   5.26599 0.55204 3.15215
H   4.06392 1.06783 4.37683
H   3.15478 2.01293 -1.31711
H   5.12522 1.59946 -2.81195
H   5.78555 -0.80410 -3.30224
H   4.43949 -2.64804 -2.28898
H -0.57110 -2.34081 1.39526
H -0.25428 -4.76926 1.12290
H   1.63733 -5.66503 -0.26186
H   3.20120 -4.08676 -1.37549
H -1.10429 3.22319 0.77620
H -0.60582 0.97792 -4.45825
H -0.93308 -0.77204 -4.25210
H -2.14940 0.43797 -3.72622
H -5.90697 0.31202 -0.83314
H -6.88484 -1.97503 -0.74944
H -5.39776 -3.97049 -0.45035
H -2.91787 -3.64548 -0.24835
H -1.94286 -1.36727 -0.35038
C   1.93162 4.96918 0.47561
H   1.57388 5.37770 1.43476
H   3.02233 5.11489 0.41245
H   1.45171 5.52336 -0.34726

65
TS(18a'CH-24a)
SCF(BP86)= -1563.18723396
G(298 K) = -1562.758217
SCF(PBE) = -4122.27471474
Low Freq. = -45.1130cm-1, 8.5978cm-1
C -5.22572 0.50468 0.78222
C -3.83006 0.36465 0.59512
C -3.15218 1.34881 -0.15965
C -3.84698 2.43672 -0.70888
C -5.23379 2.56327 -0.51833
C -5.92061 1.59149 0.22926
C -3.10212 -0.77419 1.22599
C -2.13065 -1.56128 0.53878
C -1.50873 -2.63217 1.21834
C -1.80687 -2.92449 2.55653
C -2.75431 -2.14557 3.23921
C -3.39011 -1.08850 2.57490
C -1.90359 -1.45186 -0.93836

```
N  -0.65019 -1.19209 -1.44878
C  -0.51542 -1.26696 -2.80991
C  -1.55990 -1.55427 -3.68842
C  -2.84498 -1.77354 -3.17054
C  -3.00036 -1.72249 -1.78295
Ru  1.01636 -0.43866 -0.31613
N  -0.05905  0.85069  2.48657
C  -0.03906  1.42967  3.50747
C  -0.01496  2.14990  4.77740
N   2.49532  0.53597  0.74850
C   2.60694  1.89822  0.54018
C   3.57865  2.64618  1.23475
C   4.44612  2.02126  2.13624
C   4.32838  0.63500  2.33157
C   3.34831 -0.06273  1.62080
C   1.64722  2.43014 -0.43152
C   0.75816  1.45528 -1.00490
C  -0.15305  1.95045 -1.97553
C  -0.20516  3.30935 -2.33640
C   0.66584  4.24172 -1.74045
C   1.59304  3.79654 -0.78935
Br  2.75791 -0.83915 -2.09416
N   1.52534 -2.32184  0.44453
C   1.94115 -3.36867  0.79206
C   2.45006 -4.66418  1.22654
H  -3.97270 -1.91107 -1.32031
H  -3.69523 -1.99117 -3.82339
H  -1.35734 -1.59277 -4.76235
H   0.49369 -1.07092 -3.18026
H  -4.11789 -0.47108  3.11167
H  -3.00059 -2.36034  4.28416
H  -1.31542 -3.76653  3.05418
H   3.22069 -1.14006  1.74022
H   4.98263  0.09306  3.01959
H   5.20124  2.60287  2.67345
H   3.65080  3.72309  1.06116
H  -0.84935  1.26170 -2.46689
H  -0.92832  3.64375 -3.09049
H   0.62633  5.29974 -2.01958
H   2.27705  4.52036 -0.33069
H  -0.80758 -3.26195  0.66699
H   0.32506  1.48135  5.58434
H   0.67256  3.00815  4.71308
H  -1.02351  2.51900  5.02266
H  -5.77064 -0.25722  1.35013
H  -7.00213  1.67580  0.37934
H  -5.77522  3.41230 -0.94831
H  -3.29570  3.19018 -1.28178
H  -2.06939  1.27414 -0.30196
H   2.12784 -5.45599  0.53008
H   3.55226 -4.65210  1.25784
H   2.07267 -4.90700  2.23377

65
24a
SCF(BP86)= -1563.22792224
G(298 K) = -1562.797025
SCF(PBE) = -4122.31637302
Low Freq. = 16.8262cm-1,
22.5851cm-1
C  -5.31759 -0.57421 -0.79877
C  -3.92535 -0.36405 -0.65683
C  -3.09023 -1.49393 -0.49247
C  -3.63080 -2.78856 -0.47130
C  -5.01566 -2.98305 -0.61091
C  -5.85720 -1.86945 -0.77400
C  -3.36862  1.01885 -0.72303
C  -2.37396  1.50231  0.17705
C  -1.92272  2.83275  0.05677
C  -2.40554  3.68114 -0.94987
C  -3.37212  3.20633 -1.85027
C  -3.84583  1.89266 -1.72921
C  -1.96233  0.72312  1.38944
N  -0.66870  0.29810  1.59249
C  -0.41116 -0.30443  2.79637
C  -1.36024 -0.51478  3.79675
C  -2.68169 -0.10310  3.57722
C  -2.96720  0.51873  2.36022
Ru  1.02017  0.27951  0.20522
N  -0.07204  0.28618 -1.41903
C  -0.56144  0.24520 -2.48950
C  -1.19568  0.20913 -3.80141
N   2.65218  0.00556 -1.05353
C   3.01462 -1.30238 -1.30379
C   4.11977 -1.58238 -2.13491
C   4.84849 -0.54276 -2.71892
C   4.45679  0.78280 -2.46676
C   3.35823  1.00909 -1.63339
C   2.15768 -2.29701 -0.65220
C   1.08125 -1.75910  0.13238
C   0.21950 -2.71130  0.73359
C   0.41808 -4.09723  0.59605
C   1.49390 -4.59438 -0.16444
C   2.35994 -3.69030 -0.79068
Br  2.82204  0.29358  2.15604
N   1.24054  2.37977  0.25271
C   1.49531  3.52680  0.31310
C   1.81224  4.94792  0.39141
H  -3.97595  0.87622  2.13750
H  -3.46173 -0.25239  4.32950
H  -1.05376 -1.00335  4.72574
H   0.62442 -0.62055  2.93484
H  -4.59488  1.51943 -2.43577
H  -3.76082  3.85571 -2.64132
H  -2.04145  4.71123 -1.01753
H   3.01728  2.01993 -1.40286
H   4.98755  1.63301 -2.90270
H   5.70555 -0.76147 -3.36290
H   4.40135 -2.62190 -2.32121
H  -0.63302 -2.37210  1.33180
H  -0.27252 -4.79552  1.08452
H   1.65077 -5.67276 -0.27041
H   3.19408 -4.07541 -1.38863
H  -1.19962  3.20706  0.78497
H  -0.64200  0.84076 -4.51633
H  -1.21850 -0.82232 -4.19089
H  -2.23063  0.58376 -3.73091
H  -5.98258  0.28930 -0.90819
H  -6.93870 -2.00765 -0.87660
H  -5.43600 -3.99387 -0.59220
H  -2.96201 -3.64760 -0.35127
H  -2.00830 -1.35876 -0.40211
```

H   1.67330 5.31331 1.42216
H   2.85919 5.12407 0.09387
H   1.15352 5.52406 -0.27883

34
25a
SCF(BP86)= -852.812569274
G(298 K) = -852.612766
SCF(PBE) = -3412.49278404
Low Freq. = -19.3928cm-1,
17.1605cm-1
Ru 0.51109 -0.08082 -0.56014
Br 0.65422 -2.58903 -0.00263
N -0.11966 0.48107 1.24324
C -1.49140 0.48541 1.46457
C -1.99055 0.84729 2.73102
C -1.12113 1.20492 3.76542
C   0.26310 1.19818 3.51903
C   0.72344 0.83406 2.25274
C -2.27484 0.09267 0.29525
C -1.47749 -0.23510 -0.84829
C -2.16546 -0.60979 -2.02628
C -3.57055 -0.67029 -2.06513
C -4.33418 -0.35172 -0.92272
C -3.68656 0.03144 0.25895
N   0.47588 1.76486 -1.23061
C   0.45328 2.86433 -1.65356
C   0.43614 4.21806 -2.19257
N   2.62003 0.05227 -0.27584
C   3.79228 0.10734 -0.20164
C   5.24484 0.17294 -0.10582
H -0.58128 4.48948 -2.51977
H   0.76460 4.94163 -1.42784
H   1.11506 4.29185 -3.05859
H   1.78622 0.81104 2.00592
H   0.98664 1.47006 4.29157
H -1.51357 1.48433 4.74734
H -3.07168 0.84277 2.89358
H -1.59591 -0.86596 -2.93005
H -4.07788 -0.96839 -2.99052
H -5.42729 -0.40169 -0.95807
H -4.28237 0.28141 1.14479
H   5.65889 0.71540 -0.97149
H   5.54338 0.69788 0.81641
H   5.66997 -0.84378 -0.08842

40
2bBr
SCF(BP86)= -985.611162
G(298 K) = -985.369830
SCF(PBE) = -3545.17999268
Low Freq. = 19.7126cm-1,
28.6805cm-1
N -1.02774 1.35555 0.07617
C -2.31051 0.84755 0.01281
C -3.41403 1.72538 -0.02868
C -3.21837 3.10898 -0.00198
C -1.90664 3.60907 0.06760
C -0.84624 2.70021 0.10453
C -2.37490 -0.61719 -0.00369
C -3.58975 -1.33943 -0.06171
C -3.57588 -2.73954 -0.06844
C -2.34204 -3.41807 -0.01476
C -1.13417 -2.70051 0.04210
C -1.10503 -1.28481 0.04294
H -4.55055 -0.81305 -0.10179
Ru 0.50521 -0.05199 0.10823
N   1.84622 -1.53126 0.08960
C   2.63915 -2.39925 0.07858
C   3.61100 -3.48556 0.05577
Br 0.56326 0.02262 -2.50940
N   2.10164 1.36807 0.12786
C   2.99927 2.12612 0.13032
C   4.11018 3.07014 0.12576
N   0.45919 -0.07144 2.07817
C   0.42271 -0.07685 3.25407
C   0.38143 -0.07599 4.71139
H   1.40413 -0.06270 5.12334
H -0.15593 0.81427 5.07826
H -0.13557 -0.97625 5.08288
H   4.06714 3.71395 1.01931
H   5.07009 2.52855 0.12529
H   4.06276 3.70719 -0.77246
H -4.42543 1.31452 -0.08252
H -4.07517 3.78864 -0.03488
H -1.70040 4.68205 0.09179
H   0.19268 3.03383 0.15580
H -4.51602 -3.29890 -0.11367
H -2.32412 -4.51478 -0.01722
H -0.19094 -3.25793 0.08203
H   4.24611 -3.45040 0.95629
H   3.09762 -4.46098 0.02490
H   4.25809 -3.40012 -0.83277

60
19aCH
SCF(BP86)= -1526.32630442
G(298 K) = -1525.937219
SCF(PBE) = -4085.47857243
Low Freq. = 15.7358cm-1,
29.4053cm-1
C -3.91707 -2.62954 0.89994
C -2.84332 -1.87848 0.37839
N -2.67816 -0.55465 0.74433
C -3.57267 0.00873 1.59910
C -4.64894 -0.69496 2.14460
C -4.82404 -2.04462 1.78917
C -1.82065 -2.36536 -0.55300
C -0.81945 -1.40444 -0.91457
C   0.19091 -1.84170 -1.80394
C   0.20546 -3.14681 -2.32733
C -0.79501 -4.07158 -1.96506
C -1.80483 -3.67970 -1.07747
Ru -1.06413 0.44319 -0.09068
Br -2.57828 0.99221 -2.19220
C   0.43812 -0.35158 1.18849
C   1.77155 -0.03852 0.74268
C   2.87538 -0.80858 1.21514
C   2.64934 -1.79662 2.19812
C   1.35564 -2.06624 2.67781
C   0.26031 -1.36261 2.17018
C   4.26635 -0.71230 0.67081

C 4.52895 -0.97138 -0.69478
C 5.84199 -0.95520 -1.18742
C 6.91888 -0.67975 -0.32565
C 6.67219 -0.42738 1.03387
C 5.35740 -0.44855 1.52903
C 1.84440 1.07931 -0.21136
N 0.62737 1.39627 -0.79886
C 0.58163 2.43752 -1.67880
C 1.69522 3.21313 -2.00246
C 2.92260 2.93297 -1.37674
C 2.98677 1.86392 -0.47788
O -1.73383 2.42300 0.66779
C -1.28459 2.86856 1.80277
C -1.71979 4.29713 2.15598
O -0.52800 2.23841 2.59184
H 3.92034 1.63295 0.03680
H 3.80999 3.54046 -1.57782
H 1.58931 4.03405 -2.71727
H -0.40304 2.62271 -2.11620
H 3.50065 -2.38518 2.55513
H 1.20697 -2.84428 3.43488
H -0.75041 -1.58034 2.52989
H -3.39448 1.06458 1.81844
H -5.33488 -0.18838 2.82888
H -5.65606 -2.62798 2.19508
H -4.03539 -3.67538 0.60343
H 0.98169 -1.14150 -2.10118
H 0.99901 -3.44839 -3.02221
H -0.78538 -5.08771 -2.37335
H -2.58267 -4.40074 -0.79883
H -0.30929 0.62662 1.45724
H -1.10099 5.01717 1.59192
H -1.58570 4.49462 3.23002
H -2.76931 4.47256 1.87132
H 5.16746 -0.24899 2.58922
H 7.50405 -0.21331 1.71327
H 7.94337 -0.66580 -0.71171
H 6.02630 -1.16471 -2.24648
H 3.69347 -1.19531 -1.36644

60
TS(19aCH-20aCH)
SCF(BP86)= -1526.313916
G(298 K) = -1525.924211
SCF(PBE) = -4085.45942698
Low Freq. = -73.1545cm-1, 16.0968cm-1
C 4.00428 -2.72479 -0.63207
C 2.89244 -1.94151 -0.26296
N 2.55061 -0.82173 -0.99394
C 3.28760 -0.50330 -2.09175
C 4.39374 -1.25069 -2.50253
C 4.76490 -2.38040 -1.75313
C 1.99831 -2.19642 0.86860
C 0.90158 -1.29064 0.99242
C 0.01047 -1.48222 2.07019
C 0.20665 -2.51335 3.00618
C 1.29644 -3.39553 2.87208
C 2.18987 -3.23852 1.80487
Ru 0.91778 0.31203 -0.29486
Br 2.95290 1.63230 2.44742
C -0.67507 -0.78444 -0.92393
C -1.97498 -0.29590 -0.55561
C -3.14516 -1.04419 -0.90934
C -3.00325 -2.19320 -1.70926
C -1.73418 -2.64131 -2.11652
C -0.58326 -1.95857 -1.70735
C -4.52845 -0.73971 -0.42241
C -4.84702 -0.84794 0.95173
C -6.15933 -0.63943 1.40139
C -7.17859 -0.31988 0.48652
C -6.87657 -0.21759 -0.88143
C -5.56320 -0.43143 -1.33316
C -1.94715 0.99547 0.14153
N -0.65953 1.42456 0.41192
C -0.45627 2.60325 1.06082
C -1.50679 3.43232 1.45403
C -2.82067 3.04403 1.13816
C -3.03618 1.82864 0.48214
O 1.60735 2.17486 -1.11630
C 1.46681 2.43566 -2.38644
C 1.93455 3.82909 -2.80659
O 0.97665 1.64620 -3.23656
H -4.04848 1.51935 0.22469
H -3.67028 3.68233 1.39908
H -1.29222 4.36756 1.97760
H 0.59169 2.84670 1.25964
H -3.90003 -2.76029 -1.97904
H -1.64664 -3.54458 -2.73100
H 0.40386 -2.33664 -1.99265
H 2.93822 0.36515 -2.65437
H 4.94592 -0.94910 -3.39625
H 5.62742 -2.98724 -2.04414
H 4.26318 -3.60596 -0.03938
H -0.85291 -0.81656 2.18324
H -0.49535 -2.63563 3.83913
H 1.44707 -4.20178 3.59726
H 3.04175 -3.92148 1.71185
H 0.64726 0.26269 -1.83610
H 1.21835 4.58260 -2.43614
H 1.99473 3.90757 -3.90135
H 2.91409 4.06162 -2.35959
H -5.32955 -0.35046 -2.40018
H -7.66431 0.02868 -1.60127
H -8.20222 -0.15527 0.83850
H -6.38814 -0.73189 2.46839
H -4.05587 -1.10046 1.66592

59
20aCH
SCF(BP86)= -1512.71630993
G(298 K) = -1512.324930
SCF(PBE) = -1511.50578817
Low Freq. = 7.7637cm-1, 21.2871cm-1
C -2.95568 0.76242 1.54181
C -1.78883 0.53331 0.78017
N -0.59680 1.10747 1.18499
C -0.56979 1.93661 2.26351
C -1.70440 2.20748 3.02809
C -2.91627 1.59394 2.66534
C -1.62699 -0.33170 -0.39396

```
C  -0.25871 -0.57105 -0.75591
C   0.02365 -1.47342 -1.80530
C  -1.01041 -2.12395 -2.48940
C  -2.35118 -1.85146 -2.16739
C  -2.68296 -0.94150 -1.14618
Ru  1.14006  0.53144  0.25160
O   1.53703  3.19897 -1.71289
C   1.87125  3.39910 -0.52456
O   1.77448  2.51968  0.44709
C  -4.13552 -0.61920 -0.98125
C  -5.06168 -1.62968 -0.63917
C  -6.43483 -1.34453 -0.55300
C  -6.90483 -0.04779 -0.81848
C  -5.99349  0.96329 -1.17220
C  -4.62178  0.68111 -1.25299
N   2.95411 -0.08574 -0.62459
C   3.45409 -1.30996 -0.23634
C   4.67839 -1.78092 -0.74764
C   5.39636 -1.00676 -1.66454
C   4.87110  0.23442 -2.06205
C   3.65427  0.65924 -1.52178
C   2.59160 -2.00775  0.72005
C   1.34308 -1.36999  0.99017
C   0.48106 -1.96542  1.93583
C   0.84926 -3.14564  2.60792
C   2.08019 -3.76729  2.32764
C   2.95084 -3.19975  1.38762
C   2.44720  4.74432 -0.07949
H  -3.88642  0.27371  1.25491
H  -3.82359  1.76058  3.25342
H  -1.63225  2.87748  3.88841
H   0.40408  2.38098  2.48559
H  -3.15982 -2.31623 -2.74054
H  -0.77599 -2.83220 -3.29205
H   1.06489 -1.67291 -2.07843
H   3.18912  1.60728 -1.80679
H   5.38921  0.86975 -2.78481
H   6.34622 -1.36852 -2.06892
H   5.05741 -2.75543 -0.42935
H  -0.49210 -1.51127  2.15091
H   0.16788 -3.58768  3.34343
H   2.36240 -4.68961  2.84513
H   3.91742 -3.67671  1.19144
H   0.95586  0.93092 -1.23669
H   1.85701  5.15670  0.75516
H   2.45175  5.45804 -0.91510
H   3.47841  4.60785  0.28803
H  -3.91515  1.46939 -1.53435
H  -6.35287  1.97416 -1.39188
H  -7.97502  0.17403 -0.75395
H  -7.13728 -2.13854 -0.27846
H  -4.69694 -2.64129 -0.43106

65
TS(20aCH-21aCH)
SCF(BP86)= -1645.47558345
G(298 K) = -1645.044420
SCF(PBE) = -1644.15412533
Low Freq. = -95.0446cm-1,
0.6507cm-1
C  -4.84091 -0.97668  0.95207
C  -4.50252 -0.67787 -0.38837
C  -5.51700 -0.21515 -1.25517
C  -6.83075 -0.03596 -0.78976
C  -7.15293 -0.32765  0.54596
C  -6.15399 -0.80222  1.41465
C  -3.11918 -0.94028 -0.90147
C  -1.94642 -0.22897 -0.48549
C  -0.65146 -0.66978 -0.92496
C  -0.56513 -1.76107 -1.81926
C  -1.71945 -2.40766 -2.27533
C  -2.98400 -2.00509 -1.81108
C  -1.91159  0.98084  0.34539
N  -0.62092  1.40759  0.60512
C  -0.40692  2.50730  1.37674
C  -1.45062  3.25895  1.91561
C  -2.77052  2.87743  1.61805
C  -2.99653  1.74295  0.83311
Ru  0.94581  0.39169 -0.25139
O   1.64858  2.32316 -0.82058
C   1.54846  2.75698 -2.04936
O   1.05772  2.10612 -3.00620
C   0.99196 -1.31513  0.88992
C   2.12171 -2.16480  0.68839
C   2.37156 -3.25576  1.55154
C   1.50646 -3.51495  2.62261
C   0.38969 -2.68495  2.83653
C   0.13333 -1.60495  1.97199
C   2.98724 -1.79642 -0.43492
N   2.59725 -0.63610 -1.07060
C   3.30501 -0.20651 -2.14949
C   4.42798 -0.88264 -2.63267
C   4.84698 -2.05472 -1.98042
C   4.11664 -2.51145 -0.87919
N   2.83838  1.40612  2.34571
C   3.73360  1.46644  3.10266
C   4.84410  1.54274  4.04641
C   2.07493  4.17618 -2.26190
H  -4.01487  1.44118  0.59224
H  -3.61786  3.45900  1.99339
H  -1.22785  4.13214  2.53400
H   0.64225  2.76417  1.54085
H  -3.88338 -2.54240 -2.12857
H  -1.63758 -3.24707 -2.97505
H   0.41905 -2.10320 -2.15526
H   5.20882  0.53039  4.28247
H   5.67027  2.12712  3.61119
H   4.51502  2.02945  4.97829
H   2.91766  0.69158 -2.63622
H   4.95495 -0.49400 -3.50774
H   5.72269 -2.60864 -2.33119
H   4.41258 -3.42775 -0.36212
H  -0.75278 -0.98342  2.14336
H  -0.28875 -2.88694  3.67328
H   1.70187 -4.36040  3.29004
H   3.24655 -3.89755  1.39969
H   0.67295  0.48395 -1.78448
H   1.41694  4.89538 -1.74512
H   2.10471  4.42446 -3.33206
H   3.08119  4.28367 -1.82569
H  -5.26759  0.01251 -2.29707
H  -7.60308  0.33120 -1.47385
```

```
H  -8.17694 -0.18996  0.90814
H  -6.39915 -1.04228  2.45464
H  -4.06522 -1.34933  1.62971

65
21aCH
SCF(BP86)= -1645.50541764
G(298 K) = -1645.071084
SCF(PBE) = -1644.18857007
Low Freq. = 21.6762cm-1,
28.5467cm-1
C  -4.36177 -0.88960  0.75567
C  -4.14261 -0.67359 -0.62455
C  -5.25951 -0.45074 -1.46067
C  -6.55944 -0.42617 -0.92840
C  -6.76397 -0.63506  0.44545
C  -5.66053 -0.87074  1.28481
C  -2.76648 -0.76445 -1.20502
C  -1.66582  0.04106 -0.79263
C  -0.35346 -0.25640 -1.28614
C  -0.17241 -1.27692 -2.24714
C  -1.26647 -2.02670 -2.69246
C  -2.54504 -1.77334 -2.16977
C  -1.73672  1.17294  0.15103
N  -0.53428  1.48435  0.76546
C  -0.49990  2.53427  1.63491
C  -1.61434  3.32111  1.92471
C  -2.82803  3.04353  1.27284
C  -2.87907  1.96664  0.38257
Ru  1.16732  0.48744  0.15217
O   1.94651  2.41336 -0.63556
C   1.53244  2.86176 -1.78389
O   0.74554  2.25774 -2.56171
C   0.80169 -1.34126  0.98333
C   1.75205 -2.35876  0.64147
C   1.64829 -3.66912  1.16532
C   0.60730 -3.99504  2.04347
C  -0.33106 -3.00741  2.40251
C  -0.23036 -1.70572  1.87834
C   2.82644 -1.93570 -0.26517
N   2.76814 -0.60259 -0.62038
C   3.72221 -0.08507 -1.43561
C   4.76375 -0.85450 -1.96013
C   4.82988 -2.21762 -1.62198
C   3.85979 -2.75291 -0.76906
N   2.23355  0.91661  1.76739
C   2.87320  1.14899  2.72579
C   3.65854  1.44574  3.91664
C   2.05367  4.25253 -2.16379
H  -3.80167  1.73491 -0.15151
H  -3.71490  3.65913  1.44798
H  -1.51880  4.14855  2.63287
H   0.47246  2.73243  2.09027
H  -3.39447 -2.38860 -2.48362
H  -1.12853 -2.81864 -3.43605
H   0.82937 -1.46643 -2.64501
H   4.25557  0.56592  4.20784
H   4.34337  2.28723  3.72138
H   2.99593  1.71622  4.75531
H   3.62067  0.98386 -1.64123
H   5.50488 -0.38892 -2.61481
H   5.62993 -2.85251 -2.01445
H   3.89718 -3.80889 -0.48891
H  -0.97355 -0.95524  2.17540
H  -1.14377 -3.25539  3.09596
H   0.52887 -5.00851  2.45037
H   2.38190 -4.43770  0.89529
H   0.38055  0.67288 -1.47974
H   1.46423  5.02025 -1.63227
H   1.95131  4.42675 -3.24508
H   3.10536  4.37674 -1.86150
H  -5.10222 -0.28413 -2.53178
H  -7.41272 -0.24354 -1.58998
H  -7.77710 -0.61853  0.86011
H  -5.81251 -1.04718  2.35486
H  -3.50526 -1.08582  1.40901

71
TS(21aCH-5bPh)
SCF(BP86)= -1778.23626378
G(298 K) = -1777.761478
SCF(PBE) = -1776.81446883
Low Freq. = -73.8094cm-1,
11.8423cm-1
C  -1.47862 -3.98672  0.33326
C  -1.61519 -2.58219  0.25454
C  -0.69047 -1.77369 -0.48952
C   0.34377 -2.46859 -1.16753
C   0.47861 -3.86715 -1.09031
C  -0.43005 -4.63262 -0.33483
C  -2.68900 -1.84021  0.92279
N  -2.63083 -0.47364  0.73558
C  -3.55282  0.32155  1.33709
C  -4.58236 -0.19385  2.13280
C  -4.66826 -1.58311  2.31764
C  -3.71096 -2.40378  1.71306
Ru -1.10379  0.20718 -0.51370
N  -0.08782 -0.17052  2.53233
C  -0.02212 -0.43294  3.67434
C   0.06037 -0.75693  5.09573
N   0.58352  0.68150 -1.73452
C   1.82583  1.06880 -1.27466
C   2.92816  1.16879 -2.15089
C   2.79212  0.91214 -3.51663
C   1.51780  0.56356 -3.98987
C   0.46639  0.46063 -3.08120
C   2.01299  1.51963  0.13925
C   3.10716  1.08948  0.94991
C   3.32815  1.74696  2.18172
C   2.50419  2.79314  2.62004
C   1.42051  3.20032  1.82781
C   1.18399  2.56795  0.59913
N  -2.27726 -0.07383 -1.96060
C  -3.07463 -0.25077 -2.81226
C  -4.05343 -0.44184 -3.87621
O  -1.67704  2.38852 -0.76718
C  -2.18858  3.29624  0.00842
C  -2.54773  4.61053 -0.70902
O  -2.41781  3.18581  1.24159
H   3.89018  1.47776 -1.73492
H   3.64780  1.00007 -4.19208
H   1.32920  0.36646 -5.04864
```

H -0.52709 0.16949 -3.42244
C 4.02118 -0.03105 0.58042
H 4.15572 1.40725 2.81345
H 2.70611 3.28037 3.57962
H 0.76430 4.01262 2.15506
H -4.12803 -1.50848 -4.14662
H -5.04892 -0.09307 -3.55318
H -3.76035 0.12595 -4.77541
H -3.39886 1.39881 1.19999
H -5.29784 0.49108 2.59570
H -5.46420 -2.02050 2.92830
H -3.74913 -3.48743 1.85140
H 1.06322 -1.91016 -1.77661
H 1.29478 -4.36450 -1.62830
H -0.32621 -5.72088 -0.27503
H -2.19138 -4.58398 0.91390
H 0.35759 2.89102 -0.04002
H -1.74550 4.91697 -1.39995
H -2.74042 5.41520 0.01662
H -3.45915 4.46230 -1.31530
H -0.77307 -0.28456 5.63969
H 0.00609 -1.84776 5.23947
H 1.01122 -0.38947 5.51323
C 5.42195 0.12176 0.70890
C 6.29200 -0.93789 0.40806
C 5.77892 -2.17384 -0.02058
C 4.38875 -2.34029 -0.14550
C 3.51860 -1.28041 0.15061
H 5.82909 1.08672 1.02989
H 7.37343 -0.79540 0.50551
H 6.45694 -3.00141 -0.25342
H 3.97604 -3.30248 -0.46707
H 2.43729 -1.42859 0.06643

71
5bPh
SCF(BP86)= -1778.27483633
G(298 K) = -1777.797747
SCF(PBE) = -1776.85470803
Low Freq. = 15.6094cm-1,
19.2699cm-1
C 3.15600 1.50285 -0.24735
C 3.93070 0.35839 -0.55114
C 5.33509 0.50560 -0.64321
C 5.94496 1.75240 -0.43480
C 5.16308 2.88056 -0.13359
C 3.76662 2.74930 -0.04226
C 3.29643 -0.96685 -0.81183
C 2.24835 -1.49945 -0.00554
C 1.70115 -2.76165 -0.31630
C 2.15949 -3.49627 -1.41956
C 3.18418 -2.97589 -2.22636
C 3.74256 -1.72699 -1.91939
C 1.85441 -0.87216 1.29544
N 0.56902 -0.44202 1.54106
C 0.31270 -0.01644 2.82100
C 1.24849 0.01347 3.85254
C 2.56435 -0.39592 3.58962
C 2.84995 -0.83706 2.29671
Ru -1.06883 -0.17568 0.16952
O -1.50289 -2.30926 -0.36745
C -1.52716 -3.36315 0.39328
O -1.03386 -3.47608 1.54326
C -1.05534 1.85441 0.36902
C -2.09706 2.53028 -0.35769
C -2.23584 3.93807 -0.32813
C -1.34472 4.72059 0.41554
C -0.30706 4.08776 1.12713
C -0.17000 2.68817 1.09878
C -2.98624 1.66340 -1.13964
N -2.67969 0.32211 -1.05836
C -3.41498 -0.58281 -1.75084
C -4.49705 -0.21066 -2.55411
C -4.83237 1.15138 -2.64475
C -4.07217 2.08621 -1.93563
N 0.05192 -0.00465 -1.46002
C 0.54751 0.14608 -2.51537
C 1.19227 0.31999 -3.81024
N -2.39051 -0.37497 1.63882
C -3.25724 -0.49802 2.42443
C -4.32212 -0.67602 3.40324
C -2.23962 -4.57177 -0.24667
H 3.85165 -1.18734 2.03380
H 3.33588 -0.38142 4.36478
H 0.93954 0.36724 4.84002
H -0.70454 0.32992 2.99806
H 4.53275 -1.31454 -2.55611
H 3.55059 -3.53863 -3.09132
H 1.72636 -4.47768 -1.63933
H -3.89884 -0.75284 4.41847
H -5.01594 0.18032 3.37597
H -4.88984 -1.59679 3.18865
H -3.08545 -1.61905 -1.61965
H -5.06033 -0.97684 -3.09339
H -5.67423 1.48040 -3.26172
H -4.31527 3.15019 -1.99570
H 0.65435 2.23985 1.66416
H 0.40215 4.69100 1.70724
H -1.45281 5.80991 0.43800
H -3.03908 4.42976 -0.88941
H 0.92648 -3.16642 0.34240
H -3.30789 -4.34064 -0.40310
H -2.15723 -5.46330 0.39306
H -1.81065 -4.79373 -1.23848
H 0.62924 -0.21268 -4.59462
H 1.24012 1.38893 -4.07622
H 2.21725 -0.08506 -3.77568
H 5.95361 -0.37219 -0.85966
H 7.03451 1.84046 -0.50213
H 5.63817 3.85358 0.02884
H 3.14427 3.62185 0.18372
H 2.06639 1.41848 -0.19037

71
TS(5bPh-3bOAc)
SCF(BP86)= -1778.25095248
G(298 K) = -1777.775478
SCF(PBE) = -1776.82396394
Low Freq. = -22.3643cm-1,
14.7090cm-1
C 4.26936 1.53270 -0.52701
C 4.79955 0.23478 -0.71539

```
C   6.20238  0.09141 -0.82367
C   7.04909  1.20775 -0.73555
C   6.50894  2.49063 -0.54504
C   5.11556  2.64842 -0.44406
C   3.90659 -0.95324 -0.86303
C   2.83160 -1.24307  0.02834
C   1.98002 -2.33875 -0.24226
C   2.19726 -3.16488 -1.35472
C   3.27318 -2.90369 -2.21983
C   4.10828 -1.80436 -1.97439
C   2.60657 -0.49764  1.30571
N   1.33071 -0.13628  1.60329
C   1.09885  0.45687  2.79512
C   2.09706  0.72184  3.74285
C   3.41502  0.34915  3.43561
C   3.67256 -0.26626  2.20567
Ru -1.68117 -0.23057  0.07525
O  -2.20887 -2.41017  0.10253
C  -1.65945 -3.29781  0.87676
O  -0.70593 -3.10235  1.67206
C  -1.45620  1.77691 -0.12314
C  -2.60298  2.43986 -0.67964
C  -2.63056  3.83758 -0.89219
C  -1.51375  4.61491 -0.56175
C  -0.37068  3.98918 -0.02325
C  -0.34566  2.59875  0.18938
C  -3.71019  1.54562 -1.01534
N  -3.45264  0.21396 -0.73619
C  -4.38436 -0.73715 -1.01203
C  -5.62220 -0.41663 -1.57342
C  -5.91182  0.92924 -1.86082
C  -4.95070  1.90485 -1.58010
N  -0.88326 -0.52307 -1.71498
C  -0.40921 -0.72050 -2.77279
C   0.21397 -0.98184 -4.06261
N  -2.42164 -0.01630  1.89866
C  -2.88506  0.10212  2.97369
C  -3.44879  0.22775  4.31095
C  -2.27765 -4.70275  0.75800
H   4.68457 -0.58260  1.93963
H   4.22839  0.52643  4.14641
H   1.84432  1.20279  4.69215
H   0.05611  0.73200  2.99024
H   4.92166 -1.57335 -2.67045
H   3.45818 -3.54609 -3.08719
H   1.53530 -4.01798 -1.53690
H  -3.32265  1.25562  4.68908
H  -4.52488 -0.01253  4.29466
H  -2.94469 -0.46784  5.00231
H  -4.07809 -1.75676 -0.75904
H  -6.34068 -1.21453 -1.77795
H  -6.87327  1.21234 -2.29941
H  -5.15114  2.95722 -1.79818
H   0.55589  2.13829  0.60929
H   0.50818  4.59405  0.23192
H  -1.52906  5.69764 -0.72401
H  -3.51844  4.32161 -1.31640
H   1.15416 -2.55026  0.44972
H  -3.35386 -4.66744  0.99896
H  -1.77869 -5.41197  1.43496
H  -2.19226 -5.07134 -0.27860
H  -0.45869 -1.57724 -4.70190
H   0.44734 -0.03574 -4.57813
H   1.15040 -1.54546 -3.91171
H   6.62951 -0.90792 -0.96088
H   8.13318  1.07336 -0.81264
H   7.16830  3.36211 -0.47793
H   4.68462  3.64580 -0.30707
H   3.18442  1.66629 -0.46422

65
TS(21aCH-4aPh)
SCF(BP86)= -1645.49084443
G(298 K) = -1645.055631
SCF(PBE) = -1644.17602982
Low Freq. = -92.7573cm-1,
18.0961cm-1
C  -4.22169 -0.40684  0.98617
C  -4.21769 -0.59181 -0.41572
C  -5.46133 -0.62553 -1.08942
C  -6.66718 -0.47110 -0.38722
C  -6.65465 -0.28734  1.00524
C  -5.42655 -0.25825  1.68869
C  -2.95552 -0.81729 -1.18688
C  -1.79886  0.01693 -1.11784
C  -0.65522 -0.33070 -1.87371
C  -0.63038 -1.47150 -2.69039
C  -1.76508 -2.29214 -2.76340
C  -2.90739 -1.95915 -2.02127
C  -1.73354  1.27004 -0.32144
N  -0.55630  1.56242  0.34015
C  -0.50573  2.73116  1.05012
C  -1.56187  3.63698  1.13173
C  -2.74462  3.36470  0.42565
C  -2.81674  2.17417 -0.30103
Ru  1.26426  0.50499  0.16500
O   2.11090  2.46677 -0.50965
C   2.14877  2.44507 -1.80147
O   1.78385  1.44005 -2.48405
C   0.71910 -1.35933  0.75137
C   1.71209 -2.37581  0.53332
C   1.47792 -3.72916  0.87170
C   0.26972 -4.10469  1.47158
C  -0.69865 -3.11861  1.74837
C  -0.46903 -1.77703  1.39809
C   2.98860 -1.89175 -0.00217
N   3.03547 -0.52181 -0.16962
C   4.19081  0.06545 -0.57194
C   5.34274 -0.66685 -0.86969
C   5.30078 -2.06683 -0.74586
C   4.12106 -2.67540 -0.30616
N   1.77429  0.87488  1.94962
C   2.10819  1.09461  3.06009
C   2.53232  1.39276  4.42294
C   2.63494  3.70619 -2.51267
H  -3.71235  1.92687 -0.87460
H  -3.58430  4.06543  0.43337
H  -1.44083  4.54903  1.72235
H   0.44303  2.93031  1.55014
H  -3.78585 -2.61164 -2.05904
H  -1.76617 -3.18747 -3.39349
H   0.26599 -1.70300 -3.27463
```

```
H   3.11558  0.55152  4.83364
H   3.16272  2.29787  4.44586
H   1.65761  1.55837  5.07427
H   4.15420  1.15512 -0.64533
H   6.24773 -0.14578 -1.19214
H   6.17996 -2.67445 -0.98060
H   4.07191 -3.76125 -0.19020
H  -1.23902 -1.03154  1.62711
H  -1.63745 -3.40054  2.24041
H   0.08856 -5.15126  1.73740
H   2.24334 -4.49115  0.68290
H   0.19256  0.37773 -1.94160
H   1.76143  4.29179 -2.84986
H   3.22050  3.44301 -3.40739
H   3.23668  4.34059 -1.84471
H  -5.47684 -0.75888 -2.17649
H  -7.61726 -0.49235 -0.93126
H  -7.59413 -0.16903  1.55477
H  -5.40577 -0.12558  2.77548
H  -3.27335 -0.39179  1.53150

65
4a'Ph
SCF(BP86)= -1645.50547421
G(298 K) = -1645.069026
SCF(PBE) = -1644.18700339
Low Freq. = 11.1947cm-1,
19.3145cm-1
C   2.67519  1.35027 -1.13068
C   3.71176  0.41907 -0.88267
C   5.04635  0.81308 -1.13825
C   5.33703  2.09828 -1.62160
C   4.29900  3.01480 -1.86148
C   2.96837  2.63409 -1.61502
C   3.40955 -0.95786 -0.39813
C   2.45229 -1.21432  0.62678
C   2.19682 -2.54554  1.01952
C   2.88150 -3.62079  0.43422
C   3.83816 -3.37316 -0.56323
C   4.08545 -2.05656 -0.97810
C   1.92166 -0.12434  1.50666
N   0.57625  0.13577  1.64263
C   0.23045  0.98962  2.66330
C   1.13519  1.62616  3.50936
C   2.50932  1.41988  3.31348
C   2.88572  0.53228  2.30498
Ru -1.08891 -0.38045  0.35723
O  -0.58139 -2.61408 -0.12003
C  -0.02977 -2.17540 -1.18755
O   0.07464 -0.89431 -1.36230
C  -1.45777  1.54037 -0.10655
C  -2.61677  1.74252 -0.94133
C  -2.97385  3.02245 -1.42566
C  -2.19571  4.14195 -1.10982
C  -1.04402  3.97145 -0.31592
C  -0.68640  2.70196  0.16987
C  -3.35745  0.52830 -1.28764
N  -2.77296 -0.63026 -0.81382
C  -3.36682 -1.82707 -1.06349
C  -4.55504 -1.94214 -1.79065
C  -5.15880 -0.77350 -2.28628
C  -4.55408  0.46124 -2.03076
N  -2.27278 -0.38065  1.90310
C  -3.03821 -0.47205  2.79896
C  -3.97181 -0.63149  3.90768
C   0.49611 -3.10513 -2.25758
H   3.93927  0.29777  2.13120
H   3.25877  1.91544  3.93727
H   0.75591  2.28841  4.29237
H  -0.83768  1.17707  2.76500
H   4.80564 -1.86696 -1.78099
H   4.38324 -4.20216 -1.02620
H   2.67630 -4.64356  0.76593
H  -4.82505  0.05855  3.79661
H  -4.36072 -1.66367  3.94476
H  -3.47246 -0.41262  4.86660
H  -2.83975 -2.69782 -0.66584
H  -4.99015 -2.93019 -1.96163
H  -6.08892 -0.82429 -2.86033
H  -5.01066  1.38334 -2.39996
H   0.22314  2.62068  0.77302
H  -0.41624  4.83810 -0.07475
H  -2.47262  5.13203 -1.48611
H  -3.85649  3.14568 -2.06426
H   1.47488 -2.72846  1.82057
H   0.20550 -4.14405 -2.04738
H   1.59628 -3.04048 -2.29115
H   0.11195 -2.80299 -3.24554
H   5.86277  0.11162 -0.93469
H   6.37784  2.38594 -1.80438
H   4.52554  4.01778 -2.23793
H   2.14997  3.33699 -1.80433
H   1.63559  1.05008 -0.96521

65
4aPh
SCF(BP86)= -1645.51651070
G(298 K) = -1645.080683
SCF(PBE) = -1644.19837960
Low Freq. = 18.6454cm-1,
24.6177cm-1
C   3.26823  1.45453 -0.42088
C   3.95267  0.24129 -0.67037
C   5.36367  0.27386 -0.76590
C   6.06870  1.47731 -0.60918
C   5.37657  2.67443 -0.35991
C   3.97387  2.65763 -0.26874
C   3.21373 -1.03767 -0.88429
C   2.14352 -1.46720 -0.04683
C   1.46073 -2.66521 -0.35078
C   1.82378 -3.44528 -1.45765
C   2.88760 -3.03694 -2.27895
C   3.56723 -1.84525 -1.99184
C   1.82731 -0.78275  1.24444
N   0.55100 -0.34619  1.51769
C   0.32021  0.14960  2.77529
C   1.29553  0.25721  3.76520
C   2.60436 -0.16050  3.47708
C   2.85517 -0.68389  2.20653
Ru -1.15772 -0.31950  0.25730
N  -0.19788 -0.03819 -1.41579
C   0.27888  0.15185 -2.47820
```

C 0.90531 0.33324 -3.78234
C -1.46366 1.65400 0.34606
C -2.70500 2.10194 -0.23308
C -3.08672 3.46306 -0.20469
C -2.24637 4.42099 0.37556
C -1.01071 4.01333 0.91829
C -0.62790 2.66134 0.89633
C -3.51351 1.05106 -0.85563
N -2.94218 -0.20418 -0.76948
C -3.58107 -1.26857 -1.32061
C -4.80958 -1.15258 -1.97734
C -5.40767 0.11679 -2.06871
C -4.75364 1.21751 -1.50518
O -2.28095 -0.94750 1.97301
H 3.85113 -1.04450 1.93667
H 3.40151 -0.09233 4.22297
H 1.02364 0.66870 4.74104
H -0.70560 0.47264 2.95802
H 4.37772 -1.51028 -2.64799
H 3.18343 -3.63835 -3.14475
H 1.28438 -4.37416 -1.67026
H -3.07247 -2.22898 -1.20023
H -5.28165 -2.04147 -2.40367
H -6.36999 0.24585 -2.57317
H -5.20036 2.21353 -1.56677
H 0.34428 2.38563 1.31993
H -0.33729 4.75970 1.35734
H -2.54259 5.47473 0.39652
H -4.04122 3.77980 -0.64172
H 0.63673 -2.97988 0.29698
H 0.31806 -0.16925 -4.56984
H 0.98008 1.40428 -4.03471
H 1.92082 -0.09792 -3.77537
H 5.91065 -0.65808 -0.94533
H 7.16166 1.47792 -0.67759
H 5.92568 3.61385 -0.23875
H 3.42459 3.58722 -0.08569
H 2.17478 1.45419 -0.36713
C -2.17756 -2.22281 1.76199
O -1.53530 -2.64140 0.74174
C -2.79732 -3.18647 2.75303
H -3.56222 -2.68768 3.36588
H -3.23905 -4.04563 2.22515
H -2.01163 -3.57586 3.42385

60
TS(26a pi-27a pi)
SCF(BP86)= -1526.28663743
G(298 K) = -1525.895079
SCF(PBE) = -4085.44072749
Low Freq. = -43.1395cm-1,
31.3742cm-1
C -3.34938 -1.69944 -2.13366
C -2.48868 -0.88471 -1.36976
N -1.30749 -1.39051 -0.86565
C -0.99691 -2.69424 -1.09990
C -1.82109 -3.54130 -1.84698
C -3.01796 -3.03420 -2.38139
C -2.73194 0.50993 -1.00629
C -1.68474 1.13604 -0.26154
C -1.90857 2.46356 0.17810
C -3.10723 3.13846 -0.10560
C -4.12455 2.50851 -0.85094
C -3.93645 1.19470 -1.29390
Ru -0.09152 -0.04292 0.17858
O 0.87031 -1.37790 1.58032
N 1.01009 1.48152 1.02906
C 1.29919 2.21695 -0.08737
C 1.83816 3.50352 -0.00132
C 2.13938 4.01261 1.27756
C 1.89503 3.22224 2.41292
C 1.31962 1.95116 2.25814
C 0.84109 1.41728 -1.25874
C 1.39441 0.04651 -1.41913
C 1.00939 -0.63016 -2.66100
C 0.28248 -0.01417 -3.65158
C -0.10818 1.36380 -3.52550
C 0.17644 2.06003 -2.37503
C 2.80562 -0.28594 -0.97904
C 3.82510 0.69016 -1.07448
C 5.16174 0.37153 -0.78161
C 5.51039 -0.93490 -0.40337
C 4.50806 -1.91810 -0.32555
C 3.17193 -1.60021 -0.61275
Br -2.39063 -0.42332 2.93182
H 2.02659 4.08580 -0.90771
H 2.56647 5.01410 1.38367
H 2.13105 3.58731 3.41598
H 1.06868 1.30710 3.10495
H -0.62305 1.85836 -4.35553
H 0.04513 -0.55893 -4.57162
H 1.39414 -1.64404 -2.81524
H -0.06928 -3.04344 -0.62853
H -1.52168 -4.58167 -2.00120
H -3.68309 -3.66899 -2.97496
H -4.27756 -1.27879 -2.52964
H -1.14025 2.98675 0.75767
H -3.24951 4.16460 0.25386
H -5.05751 3.03651 -1.07342
H 3.57582 1.70474 -1.39914
H 2.39695 -2.36714 -0.50445
H 5.93166 1.14658 -0.86261
H 4.76661 -2.94193 -0.03292
H -4.73812 0.69478 -1.84952
H 6.55197 -1.18589 -0.17649
H -0.11073 3.11124 -2.27327
C 1.08314 -2.65136 1.77373
O 1.15378 -3.53757 0.88621
C 1.28430 -3.03329 3.24586
H 0.36491 -2.81599 3.81663
H 1.52879 -4.10122 3.34487
H 2.09133 -2.42821 3.69206

59
27a pi
SCF(BP86)= -1512.72844718
G(298 K) = -1512.334616
SCF(PBE) = -1511.52245311
Low Freq. = 16.3049cm-1,
27.7709cm-1
C -3.92845 -1.84800 -0.40846
C -2.87383 -0.95248 -0.13370

N -1.60507 -1.43143 0.12603
C -1.39934 -2.77463 0.16045
C -2.41129 -3.70155 -0.09693
C -3.70147 -3.22704 -0.39790
C -2.96958 0.50422 -0.04787
C -1.73856 1.18008 0.24901
C -1.80470 2.58331 0.43669
C -3.01516 3.28494 0.31237
C -4.20944 2.60610 -0.00627
C -4.18436 1.21827 -0.17994
Ru -0.14651 -0.01578 0.49916
O 1.09309 -1.55299 1.84862
N 1.21201 1.51432 0.75198
C 1.36489 1.96011 -0.53230
C 2.03454 3.15285 -0.82330
C 2.62168 3.86583 0.23968
C 2.51420 3.36423 1.54753
C 1.79294 2.18344 1.77578
C 0.62004 1.03713 -1.43603
C 0.93646 -0.40094 -1.46509
C 0.17686 -1.18141 -2.42681
C -0.74022 -0.62316 -3.29538
C -0.94305 0.78913 -3.31950
C -0.26754 1.59360 -2.42318
C 2.29581 -0.95911 -1.11962
C 3.47299 -0.18360 -1.23700
C 4.73801 -0.74300 -0.99119
C 4.86169 -2.09701 -0.64196
C 3.70318 -2.88773 -0.54487
C 2.44146 -2.32545 -0.77963
H 2.10738 3.50379 -1.85657
H 3.16208 4.79702 0.04673
H 2.96853 3.88825 2.39255
H 1.63582 1.77554 2.77795
H -1.62529 1.23247 -4.05177
H -1.27193 -1.26232 -4.00791
H 0.40225 -2.24991 -2.50338
H -0.38294 -3.08323 0.41864
H -2.18741 -4.77080 -0.06003
H -4.51775 -3.92359 -0.61109
H -4.92599 -1.45532 -0.62329
H -0.89507 3.14448 0.67853
H -3.02981 4.37114 0.46242
H -5.15044 3.15659 -0.10619
H 3.40582 0.86317 -1.54409
H 1.55118 -2.95413 -0.68591
H 5.63095 -0.11676 -1.09244
H 3.78115 -3.94790 -0.28108
H -5.11631 0.68675 -0.40496
H 5.84821 -2.53436 -0.45707
H -0.41644 2.67782 -2.43028
C 0.37234 -1.13548 2.81074
O -0.52963 -0.22942 2.57392
C 0.55200 -1.63817 4.22620
H 1.46973 -1.20080 4.65602
H -0.30210 -1.35885 4.85967
H 0.67593 -2.73229 4.22501

65
TS(27a pi-28a pi)
SCF(BP86)= -1645.46950824
G(298 K) = -1645.035566
SCF(PBE) = -1644.15785894
Low Freq. = -116.6104cm-1,
21.8407cm-1
C -3.78354 -2.07259 -1.03953
C -2.82881 -1.16594 -0.53482
N -1.49495 -1.51214 -0.48939
C -1.11848 -2.75189 -0.89607
C -2.02547 -3.68994 -1.39486
C -3.38539 -3.33910 -1.47727
C -3.09564 0.16591 0.00667
C -1.94043 0.88280 0.45735
C -2.14871 2.13784 1.07602
C -3.44063 2.67399 1.21462
C -4.56580 1.97137 0.73878
C -4.39096 0.71792 0.14199
Ru -0.20560 -0.08093 0.25355
O -0.69244 -0.97462 2.08215
N 0.97650 1.55465 0.82673
C 0.88200 2.33004 -0.29567
C 1.29604 3.66728 -0.31453
C 1.89592 4.19649 0.84226
C 2.05640 3.37089 1.96718
C 1.57815 2.05255 1.93597
C 0.19063 1.55567 -1.36087
C 0.72186 0.26878 -1.83319
C -0.01039 -0.33690 -2.93165
C -1.10128 0.25839 -3.53379
C -1.52994 1.55529 -3.12324
C -0.88747 2.18736 -2.07750
C 2.18389 -0.11007 -1.76391
C 3.20755 0.86084 -1.65009
C 4.56352 0.49842 -1.69929
C 4.93619 -0.84215 -1.88522
C 3.93300 -1.81708 -2.02077
C 2.58097 -1.45570 -1.95748
N 1.74181 -2.28223 0.82206
C 2.47773 -2.45560 1.72059
C 3.37527 -2.65257 2.85319
H 1.16736 4.26717 -1.21994
H 2.24050 5.23471 0.86003
H 2.53164 3.74595 2.87784
H 1.61701 1.35966 2.78269
H -2.35637 2.05063 -3.64267
H -1.60142 -0.24318 -4.36865
H 0.37161 -1.27638 -3.34208
H -0.05280 -2.97086 -0.78967
H -1.66715 -4.67336 -1.71025
H -4.12285 -4.04613 -1.86879
H -4.83649 -1.78074 -1.07914
H -1.29642 2.70993 1.45705
H -3.57125 3.64867 1.69947
H -5.56958 2.39489 0.84553
H 2.94779 1.91695 -1.54717
H 1.82007 -2.23582 -2.04815
H 5.32968 1.27626 -1.61198
H 4.20324 -2.86767 -2.17147
H -5.26723 0.15969 -0.20717
H 5.99326 -1.12314 -1.93331
H -1.20591 3.18288 -1.75306
H 4.39894 -2.34096 2.59129

H   3.00496 -2.03977 3.69078
H   3.39014 -3.71317 3.15084
C -0.04443 -1.01625 3.22073
C -0.75108 -1.89669 4.26072
O   1.04243 -0.46360 3.49958
H -0.34654 -1.70269 5.26488
H -1.83898 -1.72523 4.25454
H -0.58425 -2.96007 4.01484

65
28a pi
SCF(BP86)= -1645.48235054
G(298 K) = -1645.047390
SCF(PBE) = -1644.17225313
Low Freq. = 25.5318cm-1,
30.4627cm-1
C -3.61985 -2.41256 -0.75135
C -2.73020 -1.36128 -0.44378
N -1.43104 -1.63889 -0.06925
C -1.03220 -2.93482 0.01213
C -1.87468 -4.01002 -0.27776
C -3.19808 -3.74234 -0.67122
C -3.04852 0.06889 -0.46671
C -1.95969 0.94334 -0.14922
C -2.25154 2.32729 -0.09494
C -3.53769 2.82589 -0.36634
C -4.58766 1.94802 -0.70172
C -4.34194 0.57086 -0.74639
Ru -0.22759 -0.00748 0.36023
O -1.19631 -0.10890 2.23117
N   0.85370 1.77555 0.50234
C   1.00487 2.10422 -0.81445
C   1.46794 3.36263 -1.21684
C   1.85686 4.28069 -0.22455
C   1.76483 3.90631 1.12575
C   1.24904 2.64595 1.46355
C   0.49285 0.98611 -1.65935
C   1.09766 -0.34786 -1.62120
C   0.50741 -1.32034 -2.51960
C -0.48468 -1.00637 -3.42937
C -0.94321 0.33701 -3.55315
C -0.45664 1.30380 -2.69623
C   2.54114 -0.60879 -1.27327
C   3.52613 0.40197 -1.36323
C   4.88304 0.12289 -1.12658
C   5.29910 -1.18011 -0.81463
C   4.33736 -2.20391 -0.74503
C   2.98330 -1.92103 -0.96967
N   1.26425 -1.13183 1.35633
C   1.98366 -1.71840 2.07474
C   2.89455 -2.43979 2.95299
H   1.53780 3.60515 -2.28114
H   2.23268 5.26945 -0.50395
H   2.07076 4.59042 1.92218
H   1.09704 2.29166 2.48878
H -1.67411 0.60047 -4.32421
H -0.86995 -1.78046 -4.10102
H   0.93504 -2.32726 -2.53437
H   0.00289 -3.08769 0.32617
H -1.49526 -5.03184 -0.19476
H -3.88791 -4.55757 -0.90918
H -4.64456 -2.17888 -1.05220
H -1.45918 3.04034 0.16236
H -3.72535 3.90535 -0.31585
H -5.58928 2.33572 -0.91485
H   3.23884 1.41782 -1.64355
H   2.25419 -2.73356 -0.89335
H   5.61794 0.93103 -1.20810
H   4.64210 -3.23050 -0.51447
H -5.16493 -0.11256 -0.98603
H   6.35718 -1.39954 -0.63910
H -0.80952 2.33683 -2.76872
H   3.81966 -2.68627 2.40601
H   3.15260 -1.82042 3.82756
H   2.43008 -3.37515 3.30581
C -0.74633 0.24072 3.41249
C -1.74476 -0.10432 4.52961
O   0.33987 0.79374 3.68389
H -1.32724 0.16182 5.51150
H -2.68964 0.44418 4.37681
H -1.98726 -1.17992 4.51318

65
28aCH
SCF(BP86)= -1645.49775217
G(298 K) = -1645.063407
SCF(PBE) = -1644.18117556
Low Freq. = 7.3874cm-1, 19.8495cm-
1
C   5.45580 0.42279 1.40582
C   4.33856 0.57789 0.55527
C   4.54164 0.54815 -0.84378
C   5.82518 0.35538 -1.37528
C   6.92849 0.18825 -0.51943
C   6.74003 0.22348 0.87200
C   2.98346 0.85775 1.12619
C   1.82805 0.05940 0.88030
C   0.54781 0.53536 1.31981
C   0.45179 1.72655 2.07542
C   1.60039 2.47100 2.36458
C   2.84860 2.04016 1.88823
C   1.81840 -1.22044 0.14390
N   0.59297 -1.56451 -0.39431
C   0.48388 -2.71709 -1.10962
C   1.55205 -3.59549 -1.29583
C   2.78701 -3.29377 -0.69647
C   2.91412 -2.10125 0.02267
Ru -1.06467 -0.42486 0.12330
O -2.05158 -1.18168 -1.56182
C -3.33705 -1.45131 -1.64426
O -4.19838 -1.27601 -0.76212
C -0.68411 1.17718 -1.07946
C -1.61345 2.25873 -0.95637
C -1.52478 3.40098 -1.78658
C -0.51402 3.49136 -2.75197
C   0.41018 2.43720 -2.89024
C   0.32185 1.30183 -2.06401
C -2.63862 2.08504 0.07896
N -2.57832 0.86567 0.72570
C -3.45898 0.61115 1.72832
C -4.42920 1.52688 2.13874
C -4.51292 2.76572 1.47818

```
C  -3.61142  3.03807  0.44536
N  -1.76376 -2.10898  1.21117
C  -2.14597 -3.07581  1.75793
C  -2.64042 -4.26699  2.43661
C  -3.71583 -2.03511 -3.01540
H   3.85912 -1.84486  0.50334
H   3.63649 -3.97719 -0.78489
H   1.40601 -4.50760 -1.88076
H  -0.51030 -2.89733 -1.52715
H   3.74212  2.64497  2.07284
H   1.52699  3.39521  2.94715
H  -0.52883  2.05444  2.43351
H  -3.36563 -0.37153  2.19495
H  -5.10746  1.26509  2.95516
H  -5.26509  3.50588  1.76719
H  -3.64795  3.99684 -0.07884
H   1.04956  0.49180 -2.19637
H   1.20071  2.50176 -3.64764
H  -0.44749  4.37458 -3.39586
H  -2.24734  4.21957 -1.68820
H  -0.28415 -0.25171  1.67539
H  -3.09129 -2.91307 -3.25038
H  -3.53699 -1.28825 -3.80767
H  -4.77631 -2.32600 -3.02892
H  -2.77569 -4.06463  3.51166
H  -3.61007 -4.56973  2.00855
H  -1.92635 -5.09835  2.31981
H   3.68595  0.68955 -1.51225
H   5.96573  0.34222 -2.46120
H   7.92952  0.03587 -0.93578
H   7.59362  0.09667  1.54608
H   5.31074  0.44771  2.49123
```

```
71
TS(28aCH-5fPh)
SCF(BP86)= -1778.23413435
G(298 K) = -1777.759149
SCF(PBE) = -1776.81076115
Low Freq. = -64.3722cm-1,
4.2469cm-1
C   3.76507  0.08807  0.99566
C   4.10776  0.56659 -0.29057
C   5.47759  0.74813 -0.59532
C   6.47170  0.45638  0.35136
C   6.11684 -0.01959  1.62481
C   4.75953 -0.20085  1.94239
C   3.06447  0.91922 -1.29763
C   1.93747  0.08992 -1.58075
C   0.99850  0.51059 -2.54933
C   1.15217  1.72295 -3.23732
C   2.25954  2.53946 -2.96439
C   3.19669  2.13778 -2.00276
C   1.82803 -1.30198 -1.03873
N   0.71738 -1.69324 -0.32646
C   0.66501 -3.00155  0.07352
C   1.65999 -3.94035 -0.19770
C   2.79342 -3.54334 -0.92398
C   2.86266 -2.21153 -1.34123
Ru -0.88131 -0.40258  0.25482
O  -1.83634 -2.03927  1.02997
C  -2.49738 -2.06255  2.17436
O  -2.71998 -1.10798  2.93672
C  -1.99692 -0.74309 -1.40410
C  -3.21490  0.01645 -1.48746
C  -4.11782 -0.13541 -2.56461
C  -3.85148 -1.05928 -3.58418
C  -2.67984 -1.83917 -3.51717
C  -1.78060 -1.68308 -2.44734
C  -3.44250  0.93629 -0.36739
N  -2.46089  0.87941  0.60665
C  -2.56974  1.69487  1.68865
C  -3.62448  2.59273  1.86316
C  -4.63043  2.65819  0.88253
C  -4.53043  1.82295 -0.23447
N   0.09990 -0.00678  2.05788
C   0.61064  0.19023  3.10136
C   1.21807  0.44067  4.40325
N   0.07061  2.67892  0.09536
C   0.00706  3.82689  0.33282
C  -0.07839  5.25410  0.63012
C  -2.97221 -3.48364  2.51437
H   3.71730 -1.84852 -1.91816
H   3.59434 -4.24886 -1.16341
H   1.53716 -4.96685  0.15924
H  -0.23799 -3.26209  0.63097
H   4.04808  2.78675 -1.77196
H   2.39486  3.48813 -3.49420
H   0.41559  2.01799 -3.99176
H  -0.08403  5.83690 -0.30474
H  -1.00397  5.46798  1.18811
H   0.78464  5.56775  1.23877
H  -1.77191  1.59997  2.42511
H  -3.65381  3.22183  2.75705
H  -5.47395  3.34742  0.98690
H  -5.29468  1.85436 -1.01587
H  -0.88217 -2.31328 -2.42408
H  -2.46964 -2.57471 -4.30360
H  -4.55241 -1.17829 -4.41709
H  -5.03839  0.45908 -2.60852
H   0.15551 -0.14506 -2.78444
H  -2.13844 -4.05129  2.96397
H  -3.29994 -4.02876  1.61567
H  -3.79316 -3.43836  3.24513
H   1.88586  1.31675  4.35489
H   0.43805  0.63518  5.15834
H   1.80879 -0.43211  4.72735
H   5.76312  1.10202 -1.59183
H   7.52607  0.59611  0.09046
H   6.89155 -0.24740  2.36419
H   4.47254 -0.56295  2.93529
H   2.70991 -0.03863  1.25692
```

```
71
5fPh
SCF(BP86)= -1778.26874714
G(298 K) = -1777.792187
SCF(PBE) = -1776.84800433
Low Freq. = 0.4136cm-1, 17.9668cm-
1
C  -3.04167 -1.44913 -0.65883
C  -3.90181 -0.32637 -0.61657
C  -5.29867 -0.54010 -0.68266
```

```
C  -5.82252 -1.83906 -0.77740
C  -4.95953 -2.94796 -0.81241
C  -3.56932 -2.74608 -0.75624
C  -3.35448  1.05937 -0.54050
C  -2.32618  1.43297  0.37359
C  -1.87530  2.76872  0.39304
C  -2.39511  3.72884 -0.48703
C  -3.39855  3.36343 -1.39797
C  -3.86873  2.04285 -1.41612
C  -1.90754  0.53508  1.49842
N  -0.60487  0.13701  1.69898
C  -0.34983 -0.53347  2.86827
C  -1.30691 -0.85011  3.83032
C  -2.64153 -0.48491  3.60196
C  -2.92408  0.21153  2.42658
Ru  1.10639  0.21482  0.35270
O  -0.21494  0.13678 -1.27813
C  -0.09957  0.83767 -2.37848
O   0.70739  1.76609 -2.59872
C   1.15609 -1.81645  0.14736
C   2.22596 -2.31061 -0.67387
C   2.39330 -3.69052 -0.93685
C   1.49866 -4.62409 -0.39981
C   0.42826 -4.16866  0.39402
C   0.26520 -2.79587  0.65523
C   3.10882 -1.28148 -1.23229
N   2.74709  0.00941 -0.90221
C   3.50236  1.04122 -1.35781
C   4.63679  0.85963 -2.15205
C   5.01228 -0.44795 -2.50592
C   4.24056 -1.51636 -2.04182
N   1.28094  2.31733  0.47117
C   1.54303  3.45396  0.62691
C   1.85257  4.87025  0.78824
N   2.37491  0.17191  1.85945
C   3.20177  0.18301  2.70151
C   4.21608  0.19657  3.74981
C  -1.06343  0.37700 -3.48427
H  -3.94128  0.54670  2.20805
H  -3.43183 -0.72077  4.32057
H  -0.99959 -1.38640  4.73223
H   0.68623 -0.83987  3.00758
H  -4.64122  1.75098 -2.13560
H  -3.81601  4.10179 -2.09036
H  -2.02971  4.76011 -0.44738
H   4.66338 -0.80463  3.86823
H   5.02005  0.91024  3.50255
H   3.77058  0.49491  4.71384
H   3.15740  2.03440 -1.07112
H   5.20557  1.73113 -2.48685
H   5.89212 -0.63004 -3.13054
H   4.51284 -2.54340 -2.29864
H  -0.58961 -2.48778  1.26666
H  -0.28651 -4.88959  0.80987
H   1.62771 -5.69242 -0.60263
H   3.21843 -4.04190 -1.56759
H  -1.12460  3.05671  1.13231
H  -2.01740  0.02090 -3.06669
H  -0.60371 -0.46305 -4.03594
H  -1.24632  1.19261 -4.20055
H   2.93682  5.01325  0.92848
H   1.53716  5.43346 -0.10567
H   1.32881  5.28195  1.66673
H  -5.97699  0.31922 -0.63846
H  -6.90729 -1.98494 -0.81781
H  -5.36814 -3.96138 -0.88536
H  -2.88718 -3.60255 -0.79482
H  -1.95884 -1.28213 -0.65489

66
TS(26a pi-29a)
SCF(BP86)= -1659.04856984
G(298 K) = -1658.617975
SCF(PBE) = -4218.09636180
Low Freq. = -70.9373cm-1,
7.7704cm-1
C   3.38795  2.98103  1.16208
C   2.45621  2.15672  0.49751
N   2.43669  0.79537  0.73907
C   3.33956  0.26590  1.60653
C   4.28188  1.04243  2.28704
C   4.30624  2.43067  2.06265
C   1.45219  2.59125 -0.47474
C   0.63214  1.53093 -1.01642
C  -0.32368  1.94539 -1.99015
C  -0.47966  3.28748 -2.37654
C   0.32423  4.29875 -1.81039
C   1.29018  3.94157 -0.86122
Ru  1.02433 -0.30148 -0.29576
Br  2.81248 -0.64467 -2.03609
N  -0.55552 -1.16787 -1.40438
C  -1.82392 -1.42987 -0.91877
C  -2.90008 -1.72842 -1.78055
C  -2.71834 -1.82299 -3.16281
C  -1.41679 -1.62409 -3.65340
C  -0.39477 -1.30302 -2.76322
C  -2.06943 -1.53784  0.55287
C  -3.12155 -0.83939  1.21783
C  -3.41499 -1.18499  2.55815
C  -2.70469 -2.18624  3.23417
C  -1.67086 -2.86971  2.57359
C  -1.36060 -2.54543  1.24510
C  -3.91992  0.24570  0.57656
C  -3.30127  1.27147 -0.17494
C  -4.05976  2.31042 -0.73405
C  -5.45395  2.34810 -0.55725
C  -6.08285  1.33487  0.18605
C  -5.32391  0.29602  0.74796
O   1.75345 -2.06261  0.87393
C   1.84269 -3.33467  0.62919
C   2.72376 -4.10503  1.63340
N  -0.18079  0.90943  2.42320
C  -0.24613  1.62585  3.35082
C  -0.33004  2.51667  4.50504
O   1.29214 -3.96250 -0.31194
H  -3.88038 -1.91122 -1.33178
H  -3.55314 -2.06336 -3.82753
H  -1.18542 -1.70390 -4.71982
H   0.62106 -1.11780 -3.11927
H  -4.20568 -0.63525  3.07995
H  -2.95856 -2.42929  4.27143
H  -1.11722 -3.66468  3.08438
```

```
H   3.24993 -0.81761  1.73385
H   4.98158  0.56104  2.97628
H   5.03028  3.07086  2.57641
H   3.39030  4.05670  0.96442
H  -0.97104  1.19686 -2.46079
H  -1.23309  3.55030 -3.13028
H   0.20243  5.34468 -2.11116
H   1.92329  4.72284 -0.42247
H  -0.58840 -3.09963  0.70169
H   3.77744 -3.79366  1.52165
H   2.65746 -5.19157  1.47019
H   2.42778 -3.87066  2.66996
H   0.03951  2.00303  5.40689
H   0.28035  3.41756  4.33388
H  -1.37532  2.82139  4.67303
H  -5.82481 -0.49904  1.31109
H  -7.16920  1.34848  0.32504
H  -6.04512  3.15915 -0.99526
H  -3.55239  3.09523 -1.30578
H  -2.21539  1.26359 -0.31329

66
29a
SCF(BP86)= -1659.08933023
G(298 K) = -1658.656897
SCF(PBE) = -4218.13746714
Low Freq. = 22.2739cm-1,
24.7150cm-1
C  -3.89504  2.13026 -2.06196
C  -2.83733  1.69382 -1.23582
N  -2.58587  0.34500 -1.09615
C  -3.34953 -0.55317 -1.76676
C  -4.40598 -0.16876 -2.59835
C  -4.68550  1.20144 -2.74601
C  -1.92695  2.54733 -0.46755
C  -0.92612  1.84439  0.29508
C  -0.02677  2.66597  1.02765
C  -0.11547  4.06906  1.02660
C  -1.11343  4.72597  0.27844
C  -2.01520  3.95932 -0.46913
Ru -1.00592 -0.17013  0.14451
Br -2.91655 -0.24919  2.03603
N   0.62870 -0.45268  1.51160
C   1.92528 -0.85169  1.25816
C   2.92824 -0.78309  2.25037
C   2.64295 -0.34696  3.54553
C   1.31473  0.01292  3.82091
C   0.36977 -0.04722  2.79867
C   2.32577 -1.49166 -0.03496
C   3.38487 -0.97640 -0.83854
C   3.84517 -1.75756 -1.92574
C   3.28562 -3.00883 -2.22009
C   2.24332 -3.50757 -1.42168
C   1.77345 -2.75429 -0.33586
C   4.01087  0.35691 -0.60174
C   3.22409  1.50172 -0.33232
C   3.82281  2.75770 -0.15241
C   5.21901  2.89879 -0.23588
C   6.01263  1.77028 -0.50326
C   5.41468  0.51351 -0.68591
O  -1.52076 -2.32733 -0.30928
C  -1.46985 -3.42990  0.36995
C  -2.30169 -4.57890 -0.23892
N   0.08976 -0.09825 -1.45048
C   0.57949  0.00829 -2.51919
C   1.22689  0.12101 -3.82118
O  -0.82816 -3.64105  1.43331
H   3.93732 -1.10562  1.97945
H   3.42212 -0.30481  4.31227
H   1.00157  0.35036  4.81321
H  -0.66731  0.24335  2.98023
H   4.64521 -1.35809 -2.55862
H   3.66216 -3.58821 -3.06977
H   1.80578 -4.48907 -1.63393
H  -3.06476 -1.59616 -1.59117
H  -4.99335 -0.93228 -3.11583
H  -5.50593  1.54025 -3.38637
H  -4.09258  3.20080 -2.16411
H   0.76806  2.20041  1.62069
H   0.60141  4.65831  1.61236
H  -1.18254  5.81884  0.27775
H  -2.79062  4.46633 -1.05617
H   0.98198 -3.14253  0.31550
H  -3.37797 -4.34742 -0.14932
H  -2.10157 -5.53047  0.27708
H  -2.08525 -4.69123 -1.31471
H   0.70780 -0.50007 -4.57119
H   1.21749  1.16696 -4.17213
H   2.27459 -0.21847 -3.75432
H   6.04219 -0.36409 -0.87615
H   7.10200  1.86599 -0.56408
H   5.68476  3.87950 -0.09336
H   3.19087  3.62965  0.04842
H   2.13475  1.40798 -0.28060

66
TS(29a-30a)
SCF(BP86)= -1659.06957755
G(298 K) = -1658.638563
SCF(PBE) = -4218.11170858
Low Freq. = -57.0731cm-1,
10.9453cm-1
C  -4.66483  2.05338 -1.73701
C  -3.47071  1.64815 -1.10775
N  -3.25883  0.30187 -0.84290
C  -4.19656 -0.61520 -1.21044
C  -5.38822 -0.24811 -1.83925
C  -5.63094  1.11190 -2.10574
C  -2.36337  2.50212 -0.68567
C  -1.27058  1.78610 -0.07853
C  -0.15624  2.57498  0.31388
C  -0.13181  3.96995  0.13951
C  -1.22484  4.64360 -0.44594
C  -2.34026  3.90501 -0.86033
Ru -1.56613 -0.19623  0.07024
Br -2.63318  0.08699  2.43118
N   1.33265 -0.34434  1.58834
C   2.61706 -0.63874  1.25193
C   3.68940 -0.41337  2.14643
C   3.43286  0.12530  3.41172
C   2.10701  0.42578  3.75940
C   1.10069  0.17239  2.81671
```

C 2.86255 -1.31460 -0.06035
C 3.92668 -0.94476 -0.93573
C 4.17403 -1.74739 -2.07364
C 3.39205 -2.87521 -2.36145
C 2.32068 -3.21026 -1.51625
C 2.05875 -2.43347 -0.37816
C 4.76107 0.27890 -0.74331
C 4.16801 1.54139 -0.50660
C 4.95861 2.69328 -0.38069
C 6.35798 2.60835 -0.48645
C 6.96021 1.36170 -0.72525
C 6.16919 0.20927 -0.85615
O -2.21156 -2.37102 0.07603
C -1.68145 -3.39338 0.67127
C -2.46214 -4.70935 0.47443
N -0.71186 -0.50521 -1.64404
C -0.23547 -0.69319 -2.70814
C 0.39437 -0.93967 -3.99958
O -0.63636 -3.39943 1.37279
H 4.70721 -0.67701 1.84704
H 4.25241 0.29626 4.11718
H 1.85200 0.84233 4.73828
H 0.04756 0.38317 3.04249
H 4.97983 -1.45574 -2.75569
H 3.61219 -3.47987 -3.24760
H 1.69538 -4.08230 -1.73512
H -3.93383 -1.64810 -0.96200
H -6.11021 -1.02300 -2.11102
H -6.55647 1.43090 -2.59441
H -4.82513 3.11678 -1.93596
H 0.70678 2.08177 0.77663
H 0.74669 4.54276 0.46250
H -1.20214 5.73052 -0.57768
H -3.19044 4.42434 -1.31986
H 1.23091 -2.70098 0.29241
H -3.44886 -4.63811 0.96476
H -1.91533 -5.56364 0.90150
H -2.64841 -4.89278 -0.59742
H -0.27186 -1.52587 -4.65531
H 0.63361 0.01080 -4.50603
H 1.33086 -1.50625 -3.85604
H 6.64496 -0.76197 -1.03029
H 8.04954 1.28367 -0.80657
H 6.97380 3.50799 -0.38553
H 4.47935 3.66234 -0.20611
H 3.07780 1.61987 -0.43971

35
30a
SCF(BP86)= -948.704979030
G(298 K) = -948.503367
SCF(PBE) = -3508.33851729
Low Freq. = 19.6271cm-1,
28.9989cm-1
C -3.45875 -0.50630 -1.30066
C -2.28116 -0.20125 -0.58959
N -1.03951 -0.38808 -1.17884
C -0.98306 -0.87569 -2.44698
C -2.12259 -1.19417 -3.19114
C -3.38813 -1.00370 -2.60679
C -2.18737 0.31460 0.77545
C -0.83591 0.52033 1.22393
C -0.67627 1.01632 2.54434
C -1.78089 1.28277 3.37153
C -3.09693 1.06831 2.90773
C -3.29718 0.58455 1.60803
Ru 0.60853 0.08979 -0.07297
Br 0.76414 -2.38936 0.75585
O 2.71338 -0.24438 -1.16889
C 3.24027 0.14797 -0.07546
C 4.74973 0.16883 0.09786
N 0.60553 1.89661 -0.76016
C 0.61569 2.99960 -1.18649
C 0.63855 4.35417 -1.72776
O 2.49370 0.50275 0.91997
H 0.02496 -1.00539 -2.85248
H -2.01212 -1.58219 -4.20722
H -4.30197 -1.24018 -3.16019
H -4.42944 -0.35119 -0.82058
H 0.33631 1.19445 2.92953
H -1.62001 1.66244 4.38839
H -3.95407 1.27812 3.55620
H -4.31935 0.41672 1.24624
H 5.09406 -0.83241 0.41158
H 5.05034 0.89129 0.87143
H 5.24494 0.41014 -0.85511
H 0.85469 4.34262 -2.81016
H -0.33622 4.84784 -1.57506
H 1.41282 4.95999 -1.22651

60
TS(19aCH-31a)
SCF(BP86)= -1526.30262580
G(298 K) = -1525.911831
SCF(PBE) = -4085.45653746
Low Freq. = -89.3859cm-1,
26.4956cm-1
C 4.10379 2.66962 0.39410
C 2.95684 1.90866 0.08658
N 2.97148 0.53219 0.24834
C 4.12003 -0.06996 0.65962
C 5.28207 0.64154 0.96872
C 5.27150 2.04281 0.84214
C 1.68437 2.41547 -0.42728
C 0.67409 1.40375 -0.63244
C -0.53241 1.86456 -1.22947
C -0.75122 3.21309 -1.55059
C 0.24161 4.18407 -1.29509
C 1.46061 3.77720 -0.73963
Ru 1.20658 -0.45726 -0.11580
Br 1.93537 -0.85859 -2.49705
C -0.76621 0.37073 1.94335
C -1.88937 0.00998 1.16271
C -3.06350 0.82099 1.22163
C -3.05643 1.95216 2.07294
C -1.93478 2.29710 2.84066
C -0.78234 1.50014 2.77680
C -4.30349 0.58496 0.41803
C -4.26986 0.39718 -0.98353
C -5.45448 0.24257 -1.71839
C -6.70136 0.26775 -1.06932
C -6.75204 0.45315 0.32201

```
C  -5.56607  0.61327  1.05629
C  -1.79273 -1.23826  0.36044
N  -0.60848 -1.50621 -0.30871
C  -0.55317 -2.68121 -1.01609
C  -1.59299 -3.60654 -1.08205
C  -2.77641 -3.35628 -0.36703
C  -2.85946 -2.16204  0.35313
O   2.05884 -2.45656  0.49892
C   2.03042 -2.48458  1.78707
C   2.48366 -3.77029  2.48034
O   1.62504 -1.50827  2.49309
H  -3.75430 -1.92524  0.93316
H  -3.60332 -4.07234 -0.36291
H  -1.45921 -4.51830 -1.67146
H   0.39116 -2.85435 -1.53507
H  -3.94779  2.58765  2.10101
H  -1.96524  3.18344  3.48311
H   0.09775  1.74315  3.38148
H   4.06738 -1.15943  0.72755
H   6.17440  0.10198  1.29777
H   6.16112  2.63405  1.08018
H   4.07346  3.75658  0.27625
H  -1.32428  1.13817 -1.44546
H  -1.70214  3.51612 -2.00733
H   0.06887  5.23692 -1.54217
H   2.24562  4.52309 -0.56260
H   0.09748 -0.31515  1.99674
H   1.59877 -4.38744  2.71750
H   2.99094 -3.54195  3.43090
H   3.14961 -4.36262  1.83438
H  -5.61213  0.74743  2.14248
H  -7.71663  0.47077  0.84033
H  -7.62501  0.14467 -1.64421
H  -5.40318  0.10787 -2.80403
H  -3.30685  0.38237 -1.50278

60
31a
SCF(BP86)= -1523.318848
G(298 K) = -1525.928864
SCF(PBE) = -4085.47192453
Low Freq. = 19.3256cm-1,
27.6039cm-1
C   4.74180  1.39134 -0.94108
C   3.55707  0.64542 -0.77695
N   2.46338  1.19155 -0.12960
C   2.55675  2.46227  0.34263
C   3.70912  3.24154  0.21516
C   4.82733  2.69625 -0.44341
C   3.30851 -0.71542 -1.24898
C   2.01648 -1.24512 -0.88411
C   1.67361 -2.50273 -1.45434
C   2.57481 -3.22463 -2.25513
C   3.85703 -2.71260 -2.54359
C   4.21175 -1.45063 -2.04861
Ru  0.89299 -0.08884  0.24435
Br  2.11928 -0.68161  2.48269
C  -1.04126  1.38223  1.68294
C  -2.03964  0.60345  1.04573
C  -3.18409  1.25818  0.50146
C  -3.30061  2.65763  0.66630
C  -2.31973  3.41242  1.32607
C  -1.17498  2.77205  1.82549
C  -4.23072  0.54477 -0.28699
C  -3.87223 -0.34765 -1.32556
C  -4.85933 -0.97696 -2.09860
C  -6.22131 -0.73198 -1.84781
C  -6.58906  0.15345 -0.82082
C  -5.60284  0.78889 -0.04918
C  -1.88288 -0.87657  1.08828
N  -0.62867 -1.38395  0.82611
C  -0.41897 -2.71167  1.08019
C  -1.41985 -3.57837  1.51782
C  -2.71476 -3.07373  1.73351
C  -2.93318 -1.70808  1.52611
O   0.33997  2.47812 -1.89112
C  -0.27467  1.42316 -2.15591
C  -1.12832  1.32227 -3.43220
O  -0.28434  0.31642 -1.45220
H  -3.91041 -1.26055  1.72358
H  -3.52430 -3.72227  2.08053
H  -1.17998 -4.63154  1.68898
H   0.60241 -3.05656  0.91095
H  -4.17124  3.16270  0.23444
H  -2.44370  4.49453  1.43440
H  -0.39679  3.34454  2.34080
H   1.65764  2.84610  0.83043
H   3.72179  4.25694  0.62076
H   5.74693  3.27730 -0.56307
H   5.59615  0.93772 -1.45197
H   0.67972 -2.92783 -1.27654
H   2.27173 -4.19566 -2.66654
H   4.55765 -3.28123 -3.16380
H   5.19233 -1.02858 -2.30135
H  -0.18110  0.87218  2.15069
H  -1.15443  2.29059 -3.95360
H  -2.15787  1.01005 -3.19113
H  -0.70849  0.56025 -4.11136
H  -5.89615  1.46807  0.75882
H  -7.64697  0.34825 -0.61535
H  -6.99029 -1.22658 -2.45022
H  -4.56387 -1.65668 -2.90503
H  -2.80993 -0.52122 -1.52914

65
TS(27a pi-4aPh)
SCF(BP86)= -1645.47065545
G(298 K) = -1645.036840
SCF(PBE) = -1644.15166000
Low Freq. = -63.4510cm-1,
4.0282cm-1
C   3.42521  0.72616  0.80067
C   3.81476  0.53197 -0.54654
C   5.18502  0.65542 -0.87557
C   6.13819  0.95653  0.10955
C   5.73949  1.14536  1.44358
C   4.38015  1.03062  1.78304
C   2.80804  0.25359 -1.61111
C   1.75557 -0.69316 -1.44643
C   0.80945 -0.87043 -2.48154
C   0.89692 -0.14162 -3.67668
C   1.94346  0.77630 -3.85233
```

C   2.87981 0.97053 -2.82698
C   1.72242 -1.64795 -0.29552
N   0.62928 -1.65704 0.54110
C   0.59274 -2.63051 1.50731
C   1.60021 -3.57673 1.69192
C   2.72612 -3.55168 0.85319
C   2.77148 -2.57479 -0.14732
Ru -0.90280 -0.23784 0.48739
O   0.13446 0.87525 2.37823
C -0.63984 0.12614 3.04922
O -1.42413 -0.70805 2.41427
C -2.20368 -1.34175 -0.52015
C -3.46364 -0.69919 -0.81396
C -4.46979 -1.35217 -1.56088
C -4.27292 -2.65892 -2.02595
C -3.06052 -3.31840 -1.73395
C -2.05355 -2.67556 -0.99589
C -3.61268 0.64566 -0.25352
N -2.49469 1.07200 0.44406
C -2.52781 2.27931 1.06800
C -3.63654 3.12932 1.01642
C -4.76734 2.72151 0.28715
C -4.74920 1.47301 -0.34561
N -0.09088 2.28424 -1.18355
C   0.06916 3.44525 -1.10491
C   0.26914 4.88831 -1.00512
C -0.71645 0.16635 4.55822
H   3.61419 -2.51929 -0.84181
H   3.53658 -4.27723 0.96661
H   1.49355 -4.32085 2.48617
H -0.29011 -2.60813 2.14907
H   3.67669 1.71102 -2.95396
H   2.02863 1.34798 -4.78229
H   0.16001 -0.30360 -4.46994
H -0.53363 5.34011 -0.40084
H   1.23835 5.10417 -0.52779
H   0.25818 5.34395 -2.00818
H -1.61985 2.54538 1.61554
H -3.60636 4.08946 1.53828
H -5.65118 3.36293 0.21908
H -5.62168 1.12828 -0.90756
H -1.12525 -3.22024 -0.78674
H -2.90084 -4.34418 -2.08854
H -5.05443 -3.16238 -2.60413
H -5.41500 -0.84111 -1.78028
H   0.01815 -1.61591 -2.35176
H -1.22368 1.09402 4.87448
H   0.29894 0.18165 4.98447
H -1.27460 -0.69471 4.95279
H   5.50501 0.49347 -1.91063
H   7.19501 1.03848 -0.16568
H   6.48207 1.38139 2.21283
H   4.05868 1.18658 2.81843
H   2.36671 0.66079 1.07611

29
32a
SCF(BP86)= -815.906697992
G(298 K) = -815.747080
SCF(PBE) = -3375.65128272
Low Freq. = 35.1455cm-1,
39.4163cm-1
C   1.60919 2.92634 0.52177
C   1.20452 1.63397 0.14083
N -0.11796 1.22836 0.35459
C -0.99908 2.11229 0.91582
C -0.62577 3.39985 1.30378
C   0.70404 3.82015 1.10729
C   2.00880 0.59490 -0.48727
C   1.27163 -0.62548 -0.73106
C   1.99248 -1.68195 -1.36107
C   3.34031 -1.53816 -1.72597
C   4.03594 -0.33233 -1.47561
C   3.36592 0.73090 -0.85616
Ru -0.60336 -0.58908 -0.17063
Br -0.15664 -1.75960 2.06206
O -3.01516 -0.25537 -0.28094
C -2.78150 0.05434 -1.49239
C -3.89854 0.38558 -2.46149
O -1.55621 0.05461 -1.93965
H -2.01801 1.73436 1.03318
H -1.37480 4.05889 1.75153
H   1.02523 4.82323 1.40258
H   2.64867 3.22358 0.35240
H   1.48316 -2.63405 -1.56666
H   3.86341 -2.37318 -2.20914
H   5.08760 -0.23006 -1.76299
H   3.90216 1.66881 -0.66256
H -4.70564 0.92385 -1.94155
H -4.32467 -0.55045 -2.86406
H -3.52771 0.98563 -3.30557

35
33a
SCF(BP86)= -948.706689160
G(298 K) = -948.504711
SCF(PBE) = -3508.33939736
Low Freq. = 8.6492cm-1, 35.8869cm-
1
C -2.54966 2.48809 -1.06751
C -1.78950 1.37365 -0.64136
C -0.34378 1.42038 -0.57431
C   0.25608 2.65854 -0.94998
C -0.50656 3.75817 -1.36932
C -1.91656 3.68135 -1.43321
C -2.37657 0.09394 -0.25395
C -3.74929 -0.22965 -0.21500
C -4.16441 -1.50839 0.17041
C -3.19129 -2.46526 0.51596
C -1.84365 -2.09982 0.46171
N -1.43005 -0.85769 0.09325
Ru 0.53501 -0.25360 -0.00742
O   0.73215 -1.29365 -1.86030
C   1.18028 -2.40579 -1.36028
C   1.54586 -3.53058 -2.30936
Br 0.52189 0.38605 2.51186
O   1.33108 -2.52250 -0.10058
H -1.04005 -2.80109 0.70438
H -3.46612 -3.47848 0.82120
H -5.22953 -1.75797 0.20058
H -4.48771 0.52916 -0.48940

H   1.34759 2.75467 -0.91095
H -0.00203 4.69165 -1.65002
H -2.50667 4.54275 -1.76300
H -3.64394 2.42365 -1.11533
H   1.27295 -4.50249 -1.87010
H   2.63787 -3.53266 -2.47263
H   1.05005 -3.40583 -3.28328
N   2.37500 0.41043 -0.17399
C   3.49316 0.78110 -0.27180
C   4.86428 1.26221 -0.40172
H   5.02420 2.15252 0.23020
H   5.58391 0.48524 -0.09181
H   5.08224 1.53782 -1.44787

41
TS(33a-34a)
SCF(BP86)= -1081.430656
G(298 K) = -1081.190100
SCF(PBE) = -3640.95917384
Low Freq. = -71.6071cm-1,
13.2712cm-1
C   3.62324 0.58271 -0.93523
C   2.33466 0.09911 -0.61152
C   1.16775 0.95137 -0.64855
C   1.40008 2.29706 -1.05066
C   2.68107 2.77308 -1.37351
C   3.80321 1.91841 -1.31644
C   2.07514 -1.28228 -0.20105
C   3.02330 -2.32102 -0.09331
C   2.62832 -3.59975 0.31418
C   1.27279 -3.83253 0.61172
C   0.36906 -2.77336 0.49207
N   0.74698 -1.52698 0.10383
Ru -0.54229 0.07413 -0.10063
N   0.76087 1.07974 2.75543
C   1.54647 1.49293 3.52351
C   2.52366 2.00714 4.47838
Br -1.01599 -0.60798 -2.46573
N -1.64664 1.67985 -0.30724
C -2.30541 2.65167 -0.44584
C -3.10735 3.86218 -0.58322
O -2.23972 -1.21257 0.60736
C -2.97595 -0.94396 1.64313
O -2.81677 0.01000 2.44690
C -4.14614 -1.92403 1.85750
H -0.70047 -2.87355 0.70344
H   0.91653 -4.81624 0.92939
H   3.36505 -4.40506 0.39526
H   4.07104 -2.12036 -0.33420
H   0.55049 2.98871 -1.11010
H   2.81181 3.81954 -1.67793
H   4.80113 2.29127 -1.57024
H   4.49350 -0.08396 -0.89088
H -4.85566 -1.85652 1.01434
H -4.68232 -1.70307 2.79277
H -3.77861 -2.96398 1.88473
H   3.01526 1.17197 5.00228
H   3.29029 2.59883 3.95324
H   2.02472 2.64922 5.22158
H -3.01116 4.48965 0.31964
H -2.77654 4.45634 -1.45227
H -4.17429 3.61460 -0.71808

41
34a
SCF(BP86)= -1081.472229
G(298 K) = -1081.229837
SCF(PBE) = -3641.00166815
Low Freq. = 15.0503cm-1,
26.2920cm-1
C   3.81964 0.95981 0.39955
C   2.53776 0.37926 0.25399
C   1.34226 1.18222 0.21806
C   1.52812 2.58346 0.34937
C   2.80210 3.15837 0.49410
C   3.95660 2.34833 0.51908
C   2.32079 -1.06467 0.12394
C   3.31872 -2.06248 0.11202
C   2.96785 -3.40934 -0.02282
C   1.60916 -3.75179 -0.14736
C   0.65654 -2.72902 -0.13073
N   0.99237 -1.42111 0.00071
Ru -0.37532 0.14308 0.01125
N -0.54230 -0.00496 1.94673
C -0.61417 -0.10054 3.11948
C -0.74082 -0.24161 4.56583
Br -0.16286 0.27176 -2.61936
N -1.53606 1.73999 -0.02574
C -2.20294 2.71145 -0.05836
C -3.05226 3.89666 -0.09824
O -2.01168 -1.36601 -0.33392
C -3.20207 -1.44596 0.18334
O -3.74212 -0.64591 0.98492
C -3.99120 -2.68965 -0.28528
H -0.41955 -2.90579 -0.23223
H   1.28808 -4.79113 -0.25731
H   3.74254 -4.18242 -0.03283
H   4.36938 -1.77481 0.20668
H   0.65086 3.24176 0.33235
H   2.90091 4.24722 0.58889
H   4.94906 2.79728 0.63180
H   4.71685 0.32915 0.42052
H -4.11002 -2.67665 -1.38257
H -4.98661 -2.72618 0.18322
H -3.43990 -3.61238 -0.03407
H   0.02641 -0.93266 4.95364
H -0.61298 0.73335 5.06564
H -1.73467 -0.64148 4.83031
H -2.87702 4.52833 0.78897
H -2.83483 4.49680 -0.99776
H -4.11862 3.61335 -0.11849

50
35a
SCF(BP86)= -1295.273885
G(298 K) = -1294.956956
SCF(PBE) = -3854.61605586
Low Freq. = 27.4935cm-1,
29.8047cm-1
C   4.45714 -1.00604 -0.42571
C   3.19552 -0.38480 -0.32920
N   2.10034 -1.09602 0.13800

```
C   2.26682 -2.40044  0.49155
C   3.49755 -3.05831  0.41114
C   4.61817 -2.34622 -0.05548
C   2.86616  0.99261 -0.68618
C   3.80572  1.90816 -1.21360
C   3.40926  3.19719 -1.58965
C   2.05099  3.55922 -1.45402
C   1.11320  2.65877 -0.92582
C   1.47679  1.35002 -0.48688
Ru  0.35202 -0.05734  0.31395
Br -0.27467 -0.83441 -2.09392
H   4.85356  1.60905 -1.34241
H   4.13769  3.90505 -1.99870
H   1.72084  4.55588 -1.77426
H   0.06804  2.98013 -0.85522
H   5.31160 -0.43024 -0.79318
H   5.59790 -2.82797 -0.13007
H   3.56883 -4.10783  0.70984
H   1.36589 -2.89678  0.86211
N  -1.34140  1.18564  0.66568
C  -1.01492  2.31612  1.37453
H   0.03023  2.36973  1.68112
C  -1.90063  3.35001  1.67308
H  -1.54014  4.21673  2.23437
C  -3.22996  3.25372  1.23227
C  -3.60096  2.08807  0.55728
H  -3.95895  4.04441  1.43229
H  -4.63845  1.93306  0.24878
C  -2.66958  1.05552  0.30279
C  -3.19922 -0.20027 -0.29885
C  -4.11072 -0.13316 -1.37781
C  -4.71457 -1.29601 -1.88038
H  -5.40702 -1.22570 -2.72601
C  -4.43189 -2.54454 -1.29967
H  -4.90828 -3.45214 -1.68533
C  -3.53829 -2.61763 -0.21621
H  -3.32049 -3.58580  0.24829
C  -2.92212 -1.45857  0.27980
H  -2.21986 -1.52110  1.11720
O  -0.36702 -1.63278  2.14083
C   0.24627 -0.80160  2.88144
H  -4.33006  0.83551 -1.84006
O   0.81612  0.24544  2.36639
C   0.31737 -0.99128  4.38652
H   0.64217 -2.01779  4.62148
H   1.00523 -0.27031  4.85162
H  -0.68788 -0.85833  4.82220

50
TS(35a-36a)
SCF(BP86)= SCF
G(298 K) = -1294.940591
SCF(PBE) = -3854.59782488
Low Freq. = -62.3873cm-1,
23.6713cm-1
C   2.59608  0.97814  1.36584
C   3.05536  0.55042  0.09762
C   3.85330  1.42894 -0.67115
C   4.17540  2.70749 -0.19096
C   3.71750  3.12406  1.07108
C   2.93192  2.25431  1.84682
C   2.80037 -0.83427 -0.38294
N   1.54344 -1.39251 -0.25832
C   1.41130 -2.70958 -0.61699
C   2.44407 -3.48407 -1.14420
C   3.70777 -2.90020 -1.32581
C   3.87372 -1.56985 -0.92869
Ru -0.22785 -0.44500  0.36901
O  -0.74127 -2.18585  1.32510
C  -0.23447 -1.99068  2.51430
C  -0.39292 -3.09890  3.53450
N  -2.03994  0.32980  0.94068
C  -3.06453  0.23001  0.01506
C  -4.35925  0.67872  0.34070
C  -4.62092  1.22867  1.60139
C  -3.57121  1.32212  2.53257
C  -2.30393  0.86052  2.16445
C  -2.63218 -0.35343 -1.25493
C  -3.48431 -0.51392 -2.36955
C  -2.99114 -1.04043 -3.57087
C  -1.62940 -1.39761 -3.65448
C  -0.77577 -1.24598 -2.54911
C  -1.23829 -0.73023 -1.30569
O   0.37317 -0.91058  2.77394
Br -0.18280  2.90170 -1.16929
H  -4.53846 -0.21780 -2.30535
H  -3.65345 -1.16319 -4.43398
H  -1.22900 -1.79583 -4.59519
H   0.27625 -1.53034 -2.66090
H  -5.16019  0.59308 -0.39906
H  -5.62681  1.57698  1.85473
H  -3.72462  1.74264  3.52996
H  -1.45247  0.89350  2.84982
H   0.41006 -3.12106 -0.47880
H   2.24767 -4.52555 -1.41335
H   4.54359 -3.46874 -1.74344
H   4.85081 -1.08490 -1.00266
H   4.78462  3.37922 -0.80455
H   3.97478  4.11921  1.44845
H   2.58158  2.56739  2.83620
H   2.00007  0.29609  1.98087
H   4.20536  1.11470 -1.65946
H  -0.81061 -2.68807  4.46779
H  -1.04404 -3.90168  3.16005
H   0.59778 -3.52059  3.77486

49
36a
SCF(BP86)= -1281.687037
G(298 K) = -1281.367044
SCF(PBE) = -1280.67292806
Low Freq. = 33.7581cm-1,
36.5498cm-1
C   3.91261 -0.64428 -0.49742
C   2.50733 -0.52459 -0.52810
N   1.88156  0.49249  0.16648
C   2.63763  1.38744  0.85665
C   4.03089  1.31461  0.91196
C   4.68134  0.27303  0.22428
C   1.57500 -1.38083 -1.26041
C   1.98166 -2.44782 -2.09675
C   1.03116 -3.19161 -2.80380
```

```
C  -0.33545 -2.86445 -2.67986
C  -0.74643 -1.81096 -1.84724
C   0.18713 -1.04017 -1.10806
Ru -0.17664  0.57372  0.02437
H   3.04499 -2.69185 -2.20432
H   1.34744 -4.01495 -3.45235
H  -1.08500 -3.43873 -3.23765
H  -1.81687 -1.58795 -1.77093
H   4.39585 -1.45760 -1.04554
H   5.77141  0.18252  0.24858
H   4.58915  2.06037  1.48380
H   2.07736  2.17759  1.36354
N  -2.23619  0.46552 -0.08563
C  -3.23779  1.01086 -0.81645
H  -2.95840  1.81159 -1.50665
C  -4.55089  0.53077 -0.71104
H  -5.33884  0.98447 -1.31801
C  -4.82975 -0.53514  0.16087
C  -3.78966 -1.08322  0.93586
H  -5.84626 -0.93037  0.24395
H  -3.96821 -1.90396  1.63613
C  -2.50820 -0.54091  0.80355
C  -1.23850 -0.91195  1.48260
C  -0.84742 -2.28141  1.67186
C   0.24662 -2.60286  2.45610
H   0.53877 -3.65053  2.58158
C   0.98287 -1.57877  3.12895
H   1.82731 -1.85405  3.76945
C   0.59789 -0.25416  3.02003
H   1.11143  0.51795  3.60263
C  -0.50123  0.13915  2.17688
H  -1.00644  1.08696  2.40642
O  -0.24496  2.89451  0.46970
C  -0.07574  3.04409 -0.78487
H  -1.43033 -3.06773  1.18165
O   0.02466  1.98723 -1.53791
C   0.04093  4.41011 -1.42198
H   1.06713  4.79184 -1.28137
H  -0.16697  4.36277 -2.50080
H  -0.64838  5.11644 -0.93462
```

55
TS(36a-4a)
SCF(BP86)= SCF
G(298 K) = -1414.063300
SCF(PBE) = -1413.29731671
Low Freq. = -96.4119cm-1,
15.2835cm-1

```
C   4.29972  0.03646 -0.27697
C   2.94785 -0.17671  0.05548
N   1.97557 -0.14155 -0.92900
C   2.34568  0.08965 -2.21672
C   3.67363  0.30862 -2.59515
C   4.67104  0.28204 -1.60436
C   2.40513 -0.44269  1.38874
C   3.19720 -0.49779  2.55673
C   2.60564 -0.73266  3.80509
C   1.20843 -0.90593  3.88114
C   0.41296 -0.85589  2.72400
C   0.97378 -0.62904  1.43655
Ru  0.05065 -0.51892 -0.31163
N   0.71544  2.54718  0.44129
C   1.36626  3.47294  0.75298
C   2.17719  4.62360  1.14022
H   3.09317  4.28603  1.65067
H   2.45993  5.20432  0.24779
H   1.60867  5.27484  1.82301
H   4.28241 -0.35128  2.49561
H   3.22193 -0.77590  4.70891
H   0.73471 -1.08031  4.85518
H  -0.66902 -0.99300  2.82819
H   5.05868  0.00634  0.50981
H   5.72139  0.44677 -1.86291
H   3.91315  0.49253 -3.64580
H   1.52811  0.08533 -2.94313
N  -1.84825 -0.99826  0.41168
C  -2.03583 -2.25149  0.93183
H  -1.17739 -2.91935  0.83639
C  -3.21113 -2.65998  1.56258
H  -3.27899 -3.67592  1.96116
C  -4.27400 -1.74992  1.68029
C  -4.11305 -0.47800  1.11956
H  -5.20813 -2.02888  2.17597
H  -4.92786  0.25079  1.14306
C  -2.91167 -0.12239  0.47456
C  -2.79189  1.20602 -0.18179
C  -3.26834  2.36736  0.46827
C  -3.23238  3.61259 -0.17808
H  -3.59631  4.50428  0.34274
C  -2.73179  3.71455 -1.48786
H  -2.71123  4.68520 -1.99410
C  -2.26463  2.56421 -2.14615
H  -1.88831  2.63369 -3.17231
C  -2.28688  1.31694 -1.49994
H  -1.94930  0.41804 -2.02613
O  -0.60643 -1.28850 -2.58397
C  -0.18764 -2.39572 -2.13195
H  -3.65353  2.29421  1.49096
O   0.27774 -2.46049 -0.91065
C  -0.20752 -3.67002 -2.94718
H   0.33223 -3.51153 -3.89505
H   0.25007 -4.50569 -2.39891
H  -1.24929 -3.92815 -3.20105
```

34
TS(3-4d)-Int(3-4)b,pi OAc
SCF(BP86)= -935.059293
G(298 K) = -934.855735
SCF(PBE) = -934.335206654
Low Freq. = -65.6502cm-1,
34.9533cm-1

```
C   1.33565 -0.47000 -0.61466
C   1.85836  0.85367 -0.39029
C   3.17502  1.20135 -0.76707
C   4.00306  0.24942 -1.37478
C   3.51449 -1.05549 -1.60184
C   2.20880 -1.40997 -1.22423
C   0.91290  1.76149  0.24920
C   1.13746  3.09610  0.63775
C   0.12741  3.83253  1.26496
C  -1.11745  3.21965  1.50120
C  -1.30579  1.89578  1.10206
```

N -0.32035 1.17268 0.49912
Ru -0.53714 -0.73985 -0.03923
N -0.19157 -2.58734 0.73370
C 0.25539 -2.63191 1.84079
C 0.79327 -2.86440 3.17206
O -2.92990 -0.71811 -0.00905
C -2.80640 -0.24153 -1.17926
O -1.60013 -0.01443 -1.64050
C -3.98056 0.03265 -2.08673
H 2.11450 3.54802 0.44605
H 1.79623 -2.41647 3.26611
H 0.13346 -2.42516 3.93854
H 0.87342 -3.95053 3.35355
H 3.55447 2.21493 -0.59100
H 5.02225 0.51702 -1.67135
H 4.16341 -1.80201 -2.07540
H 1.85699 -2.43298 -1.40581
H 0.30483 4.86922 1.56517
H -1.93940 3.75651 1.98137
H -2.25102 1.36715 1.24815
H -3.71021 0.74123 -2.88281
H -4.30672 -0.91241 -2.55513
H -4.82423 0.42579 -1.49958

34
Int(3-4)bOAc,p1
SCF(BP86)= SCF
G(298 K) = -934.874012
SCF(PBE) = -934.355966401
Low Freq. = 36.9886cm-1,
41.6534cm-1
C 1.36268 -0.92048 -0.15631
C 2.03646 0.31450 -0.43663
C 3.40257 0.33763 -0.80033
C 4.11604 -0.86295 -0.88874
C 3.47039 -2.08819 -0.60910
C 2.11679 -2.11721 -0.23574
C 1.16963 1.47531 -0.33196
C 1.50974 2.82745 -0.53911
C 0.54033 3.82561 -0.42206
C -0.78109 3.46333 -0.09505
C -1.08072 2.11733 0.11272
N -0.13523 1.14351 0.00315
Ru -0.56184 -0.79708 0.26105
N -0.14391 -1.67447 2.16824
C -0.44119 -0.48809 2.31120
C -0.57912 0.63660 3.26893
O -2.94790 -0.73115 0.05594
C -2.72160 -0.73611 -1.19339
O -1.48218 -0.67303 -1.61525
C -3.82219 -0.84601 -2.22064
H 2.54214 3.08090 -0.79460
H 0.21100 1.38243 3.08214
H -1.55024 1.13954 3.13534
H -0.49490 0.27137 4.30524
H 3.90542 1.28678 -1.01899
H 5.17362 -0.85191 -1.17065
H 4.03648 -3.02494 -0.67130
H 1.63985 -3.07671 -0.00062
H 0.80541 4.87407 -0.58529
H -1.57434 4.20899 -0.00266
H -2.08603 1.77138 0.36479
H -3.56792 -0.27741 -3.12767
H -3.94354 -1.90493 -2.50872
H -4.77388 -0.49008 -1.80094

15.2 Model 2.

33
2-ppy^{F}-H
SCF(BP86)= -835.080793770
G(298 K) = -834.868226
SCF(PBE) = -834.435584020
Low Freq. = 23.7066cm-1,
48.4071cm-1
C -1.22296 0.98480 -0.12259
C -0.46429 -0.20194 -0.01777
C -1.20208 -1.39995 0.11041
C -2.59239 -1.36815 0.14323
C -3.34571 -0.18451 0.04835
C -2.62823 1.02994 -0.09301
C 1.02610 -0.27581 -0.04798
N 1.54090 -1.50538 -0.31299
C 2.87819 -1.63597 -0.32854
C 3.80126 -0.59940 -0.09148
C 3.26570 0.68136 0.19610
C 1.86820 0.82779 0.21340
F -0.57083 2.18031 -0.28255
F -3.25591 -2.55612 0.27891
C -4.85381 -0.21255 0.09340
C -3.35759 2.34818 -0.21538
H -0.66310 -2.34716 0.18053
H 1.43706 1.80337 0.44288
H 3.25653 -2.64489 -0.54918
C 4.16712 1.85875 0.48049
C 5.28633 -0.85976 -0.14031
H 3.58475 2.77008 0.68651
H 4.81953 1.66205 1.35023
H 4.83713 2.06377 -0.37384
H 5.78287 -0.23616 -0.90578
H 5.77220 -0.62206 0.82339
H 5.49637 -1.91562 -0.37466
H -5.24083 0.39237 0.93260
H -5.28962 0.20780 -0.83048
H -5.22206 -1.24103 0.21091
H -2.65377 3.18342 -0.32862
H -4.03642 2.34710 -1.08641
H -3.98127 2.54020 0.67568

16
para-OMe-C6H4-Br
SCF(BP86)= -359.586193363
G(298 K) = -359.501186
SCF(PBE) = -2919.65889906
Low Freq. = 73.2011cm-1,
132.0599cm-1
C -2.06295 0.28296 0.00000
C -1.24880 1.43841 0.00000
C 0.14530 1.32549 -0.00000
C 0.72730 0.04636 -0.00000
C -0.06131 -1.10983 0.00000

C -1.46296 -0.99346 0.00000
Br 2.65500 -0.11692 -0.00001
H 0.76853 2.22340 -0.00000
H 0.40053 -2.10057 -0.00000
H -1.72712 2.42226 0.00000
H -2.06752 -1.90309 0.00000
O -3.41246 0.51010 0.00001
C -4.27780 -0.63655 0.00001
H -5.30124 -0.23696 0.00001
H -4.12563 -1.25697 -0.90179
H -4.12563 -1.25697 0.90180

15
C6H4OMe radical
SCF(BP86)= -346.084775440
G(298 K) = -345.999938
SCF(PBE) = -345.795273601
Low Freq. = 95.5539cm-1,
210.8852cm-1
C -0.38546 -0.27202 0.00006
C 0.59142 -1.29582 0.00014
C 1.96040 -0.96751 0.00004
C 2.28558 0.38313 -0.00003
C 1.37339 1.42316 0.00003
C -0.00262 1.08668 0.00003
H 2.71975 -1.76149 -0.00003
H 1.67710 2.47911 -0.00006
H 0.26139 -2.34336 0.00011
H -0.74547 1.89130 -0.00012
O -1.68915 -0.71139 -0.00021
C -2.72105 0.28389 -0.00001
H -3.67117 -0.26988 -0.00008
H -2.66914 0.92303 0.90153
H -2.66921 0.92341 -0.90129
57
1^F
SCF(BP86)= -1460.52502706
G(298 K) = -1460.153945
SCF(PBE) = -1459.39374246
Low Freq. = -127.4533cm-1, -
87.6303cm-1
C -1.22610 -0.49533 -0.00002
C -1.67576 0.86592 -0.00005
C -3.06168 1.12913 -0.00009
C -4.05091 0.13786 -0.00009
C -3.62371 -1.21407 -0.00005
C -2.23505 -1.46545 -0.00002
C -0.61937 1.88577 -0.00005
N 0.65509 1.36074 -0.00001
C 1.71545 2.20170 0.00000
C 1.61487 3.60083 -0.00003
C 0.31151 4.16066 -0.00007
C -0.78572 3.28344 -0.00008
Ru 0.81730 -0.71656 0.00003
N 0.80318 -0.70959 2.00083
C 0.78920 -0.69913 3.17311
C 0.77279 -0.68808 4.62913
N 0.91294 -2.73015 0.00007
C 1.04729 -3.89462 0.00009
C 1.17520 -5.34568 0.00012
N 0.80327 -0.70966 -2.00077
C 0.78934 -0.69924 -3.17305
C 0.77300 -0.68824 -4.62907
N 2.94394 -0.75035 0.00007
C 4.11576 -0.81022 0.00010
C 5.57101 -0.87431 0.00012
F -3.51304 2.42785 -0.00012
H 1.54443 -1.36991 5.02217
H 0.97462 0.32911 5.00244
H -0.21248 -1.01401 5.00048
H 5.97141 -0.37373 0.89656
H 5.90431 -1.92463 0.00013
H 5.97144 -0.37374 -0.89631
H -1.79700 3.68988 -0.00011
C 0.06442 5.65231 -0.00010
C 2.87442 4.43610 -0.00001
H 2.69603 1.71899 0.00003
C -5.50676 0.55093 -0.00013
C -4.61015 -2.36211 -0.00005
F -1.86257 -2.79330 0.00002
H 1.72513 -5.67901 0.89527
H 0.17817 -5.81615 0.00015
H 1.72508 -5.67905 -0.89505
H 1.54525 -1.36941 -5.02206
H -0.21197 -1.01503 -5.00047
H 0.97396 0.32912 -5.00239
H 0.99584 6.23545 -0.00010
H -0.52370 5.94787 -0.88631
H -0.52373 5.94790 0.88607
H 3.77029 3.79552 0.00002
H 2.92836 5.08989 -0.88800
H 2.92832 5.08993 0.88795
H -6.17653 -0.31998 -0.00013
H -5.74786 1.16468 0.88532
H -5.74782 1.16465 -0.88561
H -4.08375 -3.32585 -0.00002
H -5.26589 -2.33048 0.88783
H -5.26585 -2.33052 -0.88796

58
$2a^F$ OAc
SCF(BP86)= -1556.39554078
G(298 K) = -1556.025685
SCF(PBE) = -1555.22504991
Low Freq. = 13.9895cm-1,
20.6849cm-1
C -1.22064 -0.72412 0.01891
C -1.94799 0.51435 -0.04334
C -3.35889 0.48922 -0.12363
C -4.11941 -0.68549 -0.14838
C -3.41799 -1.91893 -0.08962
C -2.01357 -1.88102 -0.01043
C -1.12590 1.73226 -0.01577
C -1.56898 3.07143 -0.05171
C -0.66675 4.14504 -0.00851
C 0.72157 3.85642 0.07600
C 1.10180 2.50876 0.10552
N 0.22482 1.47783 0.05911
Ru 0.81705 -0.51369 0.10912
N 0.76025 -0.46502 2.10005
C 0.73484 -0.42375 3.27350
C 0.69785 -0.37793 4.72952

C 1.76734 4.94339 0.13336
C -1.16440 5.56932 -0.04865
F -4.05218 1.67850 -0.18382
C -5.62793 -0.64008 -0.23556
C -4.16641 -3.23192 -0.11324
F -1.37936 -3.10710 0.04165
N 1.35890 -2.43144 0.18663
C 1.72696 -3.54432 0.26086
C 2.16710 -4.93158 0.34115
O 2.92407 0.13500 0.32431
C 3.94275 -0.21588 -0.41390
C 5.25361 0.48878 -0.00707
N 0.86989 -0.53208 -1.87912
C 0.85225 -0.52255 -3.05266
C 0.87422 -0.51644 -4.50949
O 3.94063 -1.01346 -1.37747
H -2.63849 3.27129 -0.11263
H 1.90939 -0.61761 -4.87589
H 0.27557 -1.35267 -4.90674
H 0.45515 0.42810 -4.89353
H 1.31959 -5.61870 0.17975
H 2.93585 -5.13486 -0.42285
H 2.60088 -5.13736 1.33373
H 2.15120 2.20060 0.17657
H 1.58277 -0.88375 5.14952
H 0.69230 0.66790 5.07802
H -0.20828 -0.88030 5.10654
H 5.18687 1.56679 -0.23705
H 5.42711 0.40136 1.07842
H 6.11112 0.06464 -0.55086
H -2.26266 5.60928 -0.11114
H -0.84928 6.12833 0.85086
H -0.74944 6.11308 -0.91646
H 1.61855 5.60181 1.00819
H 2.78059 4.51679 0.19797
H 1.72709 5.59132 -0.76080
H -4.76595 -3.33480 -1.03589
H -3.47271 -4.08151 -0.05695
H -4.87386 -3.30515 0.73254
H -6.09743 -1.13749 0.63229
H -5.98981 0.39683 -0.27028
H -5.99591 -1.16131 -1.13777

58
TS($2a^F$-$3a^F$)OAc
SCF(BP86)= -1556.36066630
G(298 K) = -1555.992244
SCF(PBE) = -1555.18922569
Low Freq. = -123.6259cm-1,
7.5667cm-1
C 1.17658 0.34762 -0.28320
C 0.73825 1.69856 -0.06158
C 1.69212 2.73871 0.00473
C 3.06786 2.54709 -0.16629
C 3.51568 1.22435 -0.43027
C 2.55737 0.19658 -0.48571
C -0.71586 1.87497 0.03630
C -1.42931 3.07824 0.21720
C -2.83203 3.10594 0.22287
C -3.53158 1.88420 0.03115
C -2.76905 0.72058 -0.12512
N -1.41458 0.69547 -0.10547
Ru -0.30943 -1.04721 -0.25283
N -0.43505 -0.99991 -2.14660
C -0.53545 -0.97300 -3.32092
C -0.67096 -0.95268 -4.77264
N 0.76000 -2.72248 -0.31671
C 1.31814 -3.75684 -0.35509
C 2.04905 -5.01755 -0.38962
O -2.10464 -2.37442 -0.18283
C -2.45639 -2.37500 1.06481
C -3.61706 -3.28757 1.45938
O -1.86073 -1.67898 1.93500
N 0.91753 -1.04610 2.33113
C 1.51033 -0.89827 3.33306
C 2.24545 -0.71467 4.58163
H -0.87257 4.00626 0.34645
H 2.78595 -1.63893 4.84121
H 2.97411 0.10485 4.47623
H 1.54898 -0.46941 5.39919
H 1.89030 -5.57734 0.54714
H 1.69987 -5.64063 -1.22963
H 3.13011 -4.83615 -0.51211
F 1.26750 4.02739 0.24548
C 4.03567 3.70548 -0.08498
C 4.98445 0.93862 -0.64526
F 3.04187 -1.06893 -0.75553
C -3.57487 4.40513 0.41664
C -5.03912 1.81709 -0.00184
H -3.24315 -0.25407 -0.27413
H -0.73084 -1.98168 -5.16527
H -1.58770 -0.41483 -5.06790
H 0.19389 -0.45159 -5.23941
H -4.14581 -2.88811 2.33825
H -4.32324 -3.41931 0.62499
H -3.22250 -4.28391 1.72707
H -4.23736 4.35850 1.29974
H -2.88012 5.24813 0.55195
H -4.22349 4.62804 -0.44977
H -5.45490 2.45033 -0.80615
H -5.38830 0.78568 -0.16701
H -5.48018 2.17738 0.94505
H 5.15424 -0.13027 -0.83226
H 5.38220 1.50791 -1.50495
H 5.58352 1.23623 0.23441
H 4.78432 3.55133 0.71308
H 4.59809 3.82918 -1.02816
H 3.50718 4.64647 0.12169

52
$3a^F$ OAc
SCF(BP86)= -1423.63164697
G(298 K) = -1423.302142
SCF(PBE) = -1422.56435221
Low Freq. = 10.9429cm-1,
22.3768cm-1
C 0.87135 0.84821 -0.10121
C -0.26413 1.72790 -0.20526
C -0.05520 3.11160 -0.39996
C 1.20789 3.70763 -0.48166
C 2.34046 2.85866 -0.34984
C 2.12521 1.48266 -0.15884

```
C  -1.57188  1.07565 -0.08466
C  -2.84586  1.68039 -0.10309
C  -4.01908  0.92616  0.04895
C  -3.90002 -0.47947  0.22027
C  -2.60966 -1.02348  0.22278
N  -1.47806 -0.29019  0.07829
Ru  0.41654 -1.10813  0.06271
N   2.23465 -1.90590 -0.04628
C   3.26653 -2.46499 -0.12777
C   4.56515 -3.11828 -0.23459
O  -0.40050 -3.20445 -0.49944
C  -0.32583 -2.83324 -1.72222
O   0.09920 -1.63952 -1.99379
N   0.54336 -0.99388  2.01042
C   0.60002 -0.95777  3.18711
C   0.66114 -0.94072  4.64363
C  -0.68994 -3.76938 -2.85325
H  -2.91419  2.76014 -0.23548
H   1.13845 -1.86195  5.01793
H   1.24563 -0.07540  4.99863
H  -0.35378 -0.87589  5.07049
H   5.32793 -2.55001  0.32357
H   4.52110 -4.14197  0.17286
H   4.87600 -3.17590 -1.29111
F  -1.14667  3.94319 -0.51901
C   1.35941  5.19638 -0.69475
C   3.73905  3.42883 -0.41142
F   3.25575  0.70406 -0.01808
C  -5.37171  1.59514  0.03048
C  -5.10407 -1.37333  0.39098
H  -2.45044 -2.10101  0.32988
H  -0.98757 -3.20467 -3.74907
H   0.18694 -4.38830 -3.11217
H  -1.50112 -4.44578 -2.54487
H  -5.69611 -1.08755  1.27903
H  -5.78353 -1.30583 -0.47768
H  -4.80535 -2.42704  0.50725
H  -5.92055  1.41338  0.97215
H  -5.27901  2.68307 -0.10898
H  -6.00425  1.19571 -0.78277
H   1.92842  5.41671 -1.61601
H   0.37819  5.68428 -0.77615
H   1.90868  5.67038  0.13885
H   4.49257  2.63877 -0.29215
H   3.92076  3.93860 -1.37487
H   3.89940  4.18304  0.38016
```

34
Int(3^{F}-4^{F})bOAc
SCF(BP86)= -935.088770274
G(298 K) = -934.884597
SCF(PBE) = -934.365477252
Low Freq. = 16.5729cm-1,
33.5807cm-1

```
C   1.24133 -0.58976 -0.79804
C   1.89702  0.65884 -0.52102
C   3.20952  0.92046 -0.97447
C   3.90125 -0.05425 -1.70524
C   3.28019 -1.29196 -1.98077
C   1.97503 -1.55638 -1.53083
C   1.07632  1.58956  0.24739
C   1.43257  2.87464  0.69822
C   0.52867  3.64618  1.43621
C  -0.74446  3.11870  1.71947
C  -1.06711  1.84247  1.25433
N  -0.18833  1.08753  0.53743
Ru -0.62529 -0.71671 -0.14017
O  -2.98306 -0.56728 -0.11432
C  -2.87100 -0.10970 -1.30051
O  -1.68405  0.03669 -1.81666
N  -0.03823 -1.60325  1.49328
C   0.26809 -2.17280  2.48161
C   0.59862 -2.89103  3.70565
C  -4.08031  0.23402 -2.14105
H   2.42891  3.26039  0.46503
H   0.05201 -3.84872  3.74347
H   1.67951 -3.10446  3.75343
H   0.31917 -2.29583  4.59151
H   3.69267  1.88159 -0.76111
H   4.91826  0.14311 -2.05903
H   3.82376 -2.05575 -2.54994
H   1.51669 -2.52890 -1.75656
H   0.81029  4.64353  1.78598
H  -1.48584  3.68315  2.29055
H  -2.04258  1.38156  1.42863
H  -3.85913  1.07279 -2.81817
H  -4.35474 -0.63745 -2.76117
H  -4.93758  0.48108 -1.49810
```

79
4a^{F}
SCF(BP86)= -2125.94593495
G(298 K) = -2125.418225
SCF(PBE) = -2124.36003254
Low Freq. = 16.4564cm-1,
19.4825cm-1

```
C   4.44922 -1.35493 -0.25557
C   3.21756 -0.67045 -0.20180
N   2.10690 -1.31654  0.30113
C   2.21577 -2.60779  0.70139
C   3.40618 -3.34496  0.65541
C   4.56750 -2.68805  0.16655
C   2.92052  0.68789 -0.66760
C   3.84340  1.56470 -1.27944
C   3.49991  2.80940 -1.81856
C   2.13063  3.18906 -1.76275
C   1.21981  2.31340 -1.14723
C   1.54249  1.08926 -0.53137
Ru  0.39997 -0.18873  0.50704
N  -0.30034 -0.87070 -1.17553
C  -0.63323 -1.31949 -2.21478
C  -1.07015 -1.88119 -3.48756
H  -0.20594 -2.05020 -4.15203
H  -1.58320 -2.84532 -3.33192
H  -1.76843 -1.19487 -3.99583
F   5.16619  1.18888 -1.36739
F  -0.10536  2.70674 -1.16066
H   5.32505 -0.83289 -0.64148
H   1.29468 -3.04904  1.09457
N  -1.23273  1.11644  0.93589
C  -0.87124  2.25538  1.59817
H   0.19733  2.33944  1.80780
```

```
C  -1.74102  3.27723  1.99039
C  -3.11629  3.13889  1.66958
C  -3.49557  1.96818  1.00065
H  -4.54751  1.80470  0.74946
C  -2.56653  0.96678  0.65581
C  -3.07846 -0.28221  0.02577
C  -3.89183 -0.23753 -1.12289
C  -4.46932 -1.36976 -1.72181
C  -4.21976 -2.63930 -1.13801
C  -3.40449 -2.67670  0.00676
F  -3.14154 -3.89077  0.58272
C  -2.84231 -1.54654  0.59846
H  -2.20898 -1.64186  1.48446
O  -0.27190 -1.48276  2.37846
C   0.46603 -0.70642  3.07585
F  -4.11427  0.98144 -1.70693
O   1.09593  0.26187  2.49092
C   0.64404 -0.91899  4.56426
H   1.38702 -1.71808  4.73263
H   1.00331 -0.00268  5.05490
H  -0.30402 -1.24401  5.01945
C   3.43046 -4.78064  1.12112
H   2.43520 -5.10523  1.46365
H   3.75074 -5.46238  0.31267
H   4.14069 -4.92343  1.95548
C   5.89518 -3.40275  0.09726
H   6.21492 -3.75240  1.09559
H   5.83424 -4.30048 -0.54422
H   6.68255 -2.74679 -0.30479
C   4.54084  3.70289 -2.45335
H   5.53721  3.24196 -2.40438
H   4.30937  3.90370 -3.51519
H   4.59326  4.68466 -1.94876
C   1.67273  4.50061 -2.35923
H   1.88711  4.54502 -3.44252
H   0.59311  4.64367 -2.21696
H   2.20002  5.35504 -1.89781
C  -1.20682  4.48782  2.71719
H  -1.68608  4.60753  3.70532
H  -0.11927  4.41222  2.87336
H  -1.40684  5.41645  2.15301
C  -5.33230 -1.23477 -2.95494
H  -4.92301 -1.82194 -3.79583
H  -6.35319 -1.61287 -2.76832
H  -5.40713 -0.18573 -3.27201
C  -4.80726 -3.90333 -1.71822
H  -4.47999 -4.05557 -2.76211
H  -4.50369 -4.78078 -1.13078
H  -5.91090 -3.86113 -1.73286
C  -4.12677  4.19473  2.04072
H  -3.87058  5.16923  1.58734
H  -5.13860  3.91653  1.70810
H  -4.15507  4.35496  3.13369
```

79
4a'F
SCF(BP86)= -2125.940623
G(298 K) = -2125.412775
SCF(PBE) = -2124.35507777
Low Freq. = 17.0833cm-1,
21.3656cm-1

```
C   1.68809  0.72023 -1.88354
C   2.52061  0.24885 -0.85218
C   3.48917  1.14927 -0.35730
C   3.66536  2.45798 -0.83446
C   2.81951  2.91663 -1.87785
C   1.85187  2.02085 -2.35936
C   2.47346 -1.15900 -0.36212
N   1.30369 -1.76401  0.02812
C   1.39607 -3.06787  0.42913
C   2.56916 -3.82597  0.49329
C   3.77732 -3.20313  0.08979
C   3.69064 -1.87245 -0.33572
Ru -0.74571 -1.08662  0.09575
N  -0.89817 -1.69167  1.92710
C  -1.01637 -2.09433  3.03126
C  -1.18308 -2.61812  4.38221
C  -0.57607  0.78829  0.78777
C  -1.74433  1.61002  0.60064
C  -1.71231  2.96630  0.99606
C  -0.62252  3.56606  1.63591
C   0.50104  2.74054  1.92382
C   0.46303  1.39849  1.51293
C  -2.92547  0.88997  0.11837
N  -2.70097 -0.46511 -0.03190
C  -3.73410 -1.27156 -0.38260
C  -5.03627 -0.81716 -0.62701
C  -5.27867  0.57836 -0.51056
C  -4.21142  1.40924 -0.13784
O  -1.15997 -2.95338 -1.22047
C  -1.04027 -2.20277 -2.24784
O  -0.78839 -0.94296 -2.07910
C  -1.14874 -2.75594 -3.65210
F  -2.81809  3.75668  0.76507
F   4.30128  0.73537  0.66438
H   4.59580 -1.35640 -0.66664
C   5.09282 -3.93953  0.10594
C   2.52328 -5.25744  0.96952
H   0.44656 -3.52608  0.71551
C   4.73327  3.35443 -0.25095
C   2.95188  4.30729 -2.44972
F   1.01526  2.43655 -3.36170
H  -1.41592 -3.69651  4.35698
H  -0.26251 -2.47453  4.97304
H  -2.00839 -2.09738  4.89676
H  -3.47823 -2.33023 -0.48899
C  -6.12301 -1.79239 -1.01028
C  -6.64766  1.15543 -0.77732
H  -4.37384  2.48259 -0.03580
F   1.54565  0.61686  1.87159
C   1.69586  3.29537  2.66539
C  -0.65268  5.02463  2.03236
H   0.91281  0.07709 -2.30685
H  -0.13764 -2.97968 -4.03543
H  -1.60702 -2.01734 -4.32724
H  -1.73327 -3.68739 -3.65745
H   5.33884 -4.29282  1.12352
H   5.05647 -4.83699 -0.53754
H   5.91631 -3.29837 -0.24394
H   1.49683 -5.55923  1.23071
H   2.89614 -5.95085  0.19458
H   3.15980 -5.40489  1.86034
```

H 2.21395 4.47349 -3.24667
H 2.80024 5.07670 -1.67193
H 3.95969 4.47312 -2.87041
H 5.45621 3.66650 -1.02567
H 4.29565 4.27787 0.16756
H 5.28615 2.84287 0.54841
H -6.65443 2.24828 -0.64481
H -6.98386 0.93103 -1.80577
H -7.40455 0.72163 -0.09896
H -6.96049 -1.76834 -0.28986
H -6.55060 -1.55289 -2.00066
H -5.73803 -2.82375 -1.04795
H 2.06540 4.22236 2.19283
H 1.43649 3.55455 3.70878
H 2.51678 2.56594 2.68919
H 0.15253 5.59691 1.53644
H -1.61232 5.48570 1.75968
H -0.50895 5.15209 3.12051

79
TS(4a′^{F}-6a^F)1
SCF(BP86)= -2125.92729520
G(298 K) = -2125.399813
SCF(PBE) = -2124.34560440
Low Freq. = -79.3699cm-1,
21.7897cm-1
C 0.90296 0.71530 -1.78534
C 2.15043 0.79568 -1.13161
C 2.76505 2.06389 -1.08778
C 2.21169 3.22417 -1.65519
C 0.95275 3.12847 -2.30632
C 0.34314 1.86526 -2.34330
C 2.72494 -0.40352 -0.48381
N 1.86125 -1.28548 0.12377
C 2.40629 -2.40195 0.68631
C 3.77191 -2.70528 0.71274
C 4.66626 -1.79610 0.08997
C 4.10982 -0.65596 -0.50512
Ru -0.24716 -1.12890 0.15941
N -0.38046 -1.86922 1.89381
C -0.47983 -2.33550 2.97350
C -0.59556 -2.92653 4.30148
C -0.52978 0.72957 0.94722
C -1.86979 1.23604 0.83003
C -2.16890 2.53019 1.31155
C -1.24036 3.35624 1.95582
C 0.07363 2.84608 2.13925
C 0.36118 1.56389 1.63949
C -2.84516 0.29102 0.27225
N -2.30183 -0.94600 -0.00574
C -3.11144 -1.94630 -0.43253
C -4.48589 -1.80002 -0.65545
C -5.05687 -0.52043 -0.41947
C -4.22058 0.50517 0.04672
O -0.21480 -3.16619 -0.72350
C -0.32723 -3.02594 -2.00322
O -0.45311 -1.89210 -2.55912
C -0.28129 -4.28866 -2.85841
F -3.44622 3.02484 1.16299
F 3.96086 2.18967 -0.43385
H 4.76088 0.06371 -1.00616
C 6.15241 -2.04956 0.05698
C 4.25341 -3.97080 1.37954
H 1.68753 -3.08840 1.13926
C 2.93839 4.54572 -1.56601
C 0.29941 4.33167 -2.94128
F -0.87165 1.73887 -2.96291
H -0.58189 -4.02799 4.24020
H 0.23865 -2.60171 4.94625
H -1.54170 -2.61716 4.77672
H -2.60793 -2.90259 -0.60233
C -5.30827 -2.97158 -1.13455
C -6.52640 -0.26884 -0.65249
H -4.63940 1.49102 0.24866
F 1.64425 1.09654 1.86227
C 1.12315 3.66260 2.85793
C -1.62879 4.72955 2.45417
H 0.41904 -0.25986 -1.98095
H 0.73286 -4.40500 -3.27968
H -0.98182 -4.20660 -3.70410
H -0.51333 -5.18595 -2.26550
H 6.56622 -2.12845 1.07849
H 6.38471 -3.00338 -0.45013
H 6.68548 -1.24228 -0.46821
H 3.41348 -4.54673 1.79864
H 4.79108 -4.62094 0.66634
H 4.95876 -3.74878 2.20029
H -0.65114 4.05501 -3.41772
H 0.09463 5.11690 -2.19197
H 0.95378 4.78350 -3.70755
H 3.17539 4.93516 -2.57223
H 2.31742 5.30954 -1.06611
H 3.87824 4.44486 -1.00688
H -6.79654 0.77284 -0.42090
H -6.80526 -0.47247 -1.70211
H -7.15000 -0.93394 -0.02812
H -6.12447 -3.20850 -0.42853
H -5.78382 -2.75916 -2.10905
H -4.68703 -3.87361 -1.25040
H 1.27529 4.64080 2.36766
H 0.82086 3.87534 3.89961
H 2.08576 3.13431 2.88135
H -1.01310 5.51859 1.98571
H -2.68334 4.94499 2.23296
H -1.48296 4.81918 3.54584

79
Int(4a′^{F}-6a^F)
SCF(BP86)= -2125.941698
G(298 K) = -2125.415435
SCF(PBE) = -2124.35627330
Low Freq. = 20.7567cm-1,
23.0350cm-1
C 0.55006 0.48025 -1.41866
C 1.89655 0.94331 -1.26164
C 2.24139 2.17210 -1.85463
C 1.35589 2.95905 -2.60949
C 0.02867 2.48679 -2.79569
C -0.31543 1.26289 -2.20602
C 2.77340 0.12253 -0.42155
N 2.10318 -0.78602 0.37055
C 2.84192 -1.60976 1.15981

```
C    4.23946 -1.59114  1.23707
C    4.93881 -0.65865  0.42441
C    4.18017  0.18502 -0.39881
Ru   0.03889 -0.95160  0.19260
N   -0.20645 -1.86803  1.92809
C   -0.38025 -2.38922  2.96728
C   -0.58115 -3.03529  4.25769
C   -0.61462  0.77383  1.10081
C   -2.02950  0.99473  1.03318
C   -2.57658  2.15349  1.63023
C   -1.81841  3.11413  2.31017
C   -0.41842  2.89644  2.40712
C    0.11316  1.74142  1.80494
C   -2.79820 -0.05181  0.34611
N   -2.01638 -1.08472 -0.11990
C   -2.60719 -2.13588 -0.73756
C   -3.98476 -2.23852 -0.96650
C   -4.80707 -1.17321 -0.51194
C   -4.19362 -0.09667  0.14521
O    0.40969 -2.96794 -0.62585
C    0.68288 -3.11016 -1.88899
O    0.69962 -2.17828 -2.73889
C    1.03402 -4.53466 -2.32772
F   -3.93451  2.37280  1.55583
F    3.50867  2.66074 -1.66968
H    4.68342  0.90555 -1.04489
C    6.44550 -0.58168  0.43753
C    4.95947 -2.54790  2.15580
H    2.26714 -2.31838  1.76136
C    1.80687  4.26983 -3.20843
C   -0.97288  3.26955 -3.61067
F   -1.57685  0.77776 -2.42362
H   -0.16860 -4.05749  4.24075
H   -0.07566 -2.46374  5.05366
H   -1.65660 -3.09298  4.49305
H   -1.92029 -2.93000 -1.04488
C   -4.55122 -3.44825 -1.66939
C   -6.30169 -1.19845 -0.72017
H   -4.80543  0.72684  0.51307
F    1.47762  1.55928  1.93753
C    0.46668  3.87975  3.13788
C   -2.46929  4.33244  2.92467
H    0.39365 -0.69627 -1.60638
H    2.12391 -4.68399 -2.23035
H    0.76600 -4.69204 -3.38343
H    0.53349 -5.28374 -1.69575
H    6.82564 -0.34549  1.44789
H    6.89936 -1.54707  0.14936
H    6.81275  0.19045 -0.25586
H    4.24989 -3.19190  2.69866
H    5.65189 -3.20065  1.59446
H    5.56890 -2.00697  2.90187
H   -1.93845  2.74783 -3.65540
H   -1.14061  4.27227 -3.17897
H   -0.61356  3.42194 -4.64382
H    1.71185  4.25774 -4.30896
H    1.18780  5.10864 -2.84383
H    2.85482  4.48108 -2.95682
H   -6.77942 -0.29787 -0.30477
H   -6.55492 -1.25881 -1.79407
H   -6.75812 -2.08319 -0.24061
H   -5.28578 -3.97718 -1.03573
H   -5.07946 -3.16510 -2.59765
H   -3.75640 -4.16240 -1.93643
H    0.40927  4.88503  2.68254
H    0.15563  3.99235  4.19226
H    1.51542  3.55382  3.12119
H   -2.05357  5.26603  2.50472
H   -3.55362  4.33219  2.74728
H   -2.30097  4.37226  4.01604
```

79

TS(4a'^{F}-6a^{F})2

SCF(BP86)= -2125.938914

G(298 K) = -2125.415840

SCF(PBE) = -2124.35220132

Low Freq. = -803.8498cm-1, 20.8558cm-1

```
N    2.08920 -0.80498  0.41084
C    2.77804  0.08586 -0.38708
C    4.18557  0.13647 -0.34067
C    4.92399 -0.69948  0.50831
C    4.20503 -1.61364  1.32471
C    2.80890 -1.62092  1.22556
C    1.91145  0.89092 -1.25180
C    0.55092  0.43552 -1.36487
C   -0.28725  1.21580 -2.18739
C    0.08408  2.39100 -2.85716
C    1.42482  2.83824 -2.71365
C    2.29140  2.06973 -1.92095
Ru   0.02566 -0.93939  0.20102
N   -0.25545 -1.92259  1.92423
C   -0.44839 -2.46445  2.95072
C   -0.67214 -3.13599  4.22481
C   -0.60207  0.77231  1.12847
C   -2.01461  1.01774  1.06301
C   -2.54497  2.17605  1.67577
C   -1.77464  3.11630  2.36964
C   -0.37789  2.87622  2.46401
C    0.13792  1.72233  1.84699
C   -2.79760 -0.00633  0.36035
N   -2.02702 -1.03931 -0.12593
C   -2.63612 -2.07170 -0.75843
C   -4.01531 -2.15451 -0.98467
C   -4.82400 -1.08804 -0.50903
C   -4.19435 -0.03139  0.16456
O    0.39332 -2.94858 -0.69859
C    0.64270 -2.95941 -1.95445
O    0.65459 -1.89798 -2.67883
C    0.94400 -4.28456 -2.63300
F   -3.90007  2.41657  1.60257
F    3.57804  2.53089 -1.77949
H    4.70604  0.84204 -0.98933
C    6.43110 -0.63302  0.54500
C    4.90256 -2.56088  2.27071
H    2.21871 -2.31208  1.83300
C    1.90703  4.09702 -3.39508
C   -0.90321  3.15366 -3.70913
F   -1.57384  0.77053 -2.37617
H   -0.17128 -2.58786  5.04009
H   -1.75090 -3.18794  4.44634
H   -0.27033 -4.16219  4.19176
```

H -1.96582 -2.87208 -1.08521
C -4.59791 -3.34560 -1.70626
C -6.31972 -1.09204 -0.71209
H -4.79465 0.79261 0.55002
F 1.49978 1.52092 1.98000
C 0.52061 3.83690 3.20879
C -2.40827 4.33547 3.00033
H 0.45720 -0.85464 -1.77340
H 1.98579 -4.28264 -2.99523
H 0.29345 -4.41349 -3.51305
H 0.80310 -5.12506 -1.93958
H -6.78498 -0.19318 -0.27897
H -6.57826 -1.13001 -1.78583
H -6.78573 -1.97928 -0.24641
H -5.33596 -3.87760 -1.07917
H -5.12656 -3.04088 -2.62748
H -3.81223 -4.06403 -1.98886
H 0.21141 3.93840 4.26491
H 1.56487 3.49718 3.18705
H 0.47680 4.84952 2.76836
H -1.98029 5.26905 2.59260
H -3.49259 4.35232 2.82332
H -2.23953 4.35915 4.09218
H 6.79706 -0.38401 1.55759
H 6.88321 -1.60562 0.27884
H 6.81507 0.12599 -0.15393
H 4.17883 -3.19041 2.81211
H 5.59891 -3.22866 1.73220
H 5.50401 -2.01327 3.01853
H -1.88535 2.66188 -3.70955
H -1.03344 4.18731 -3.34174
H -0.55376 3.22902 -4.75442
H 1.79908 4.02454 -4.49228
H 1.31909 4.97574 -3.07491
H 2.96403 4.29045 -3.16721

79
6a[F]
SCF(BP86)= -2125.94775493
G(298 K) = -2125.420690
SCF(PBE) = -2124.35933155
Low Freq. = 18.4667cm-1,
19.7670cm-1
N -2.03591 -0.65946 -0.62204
C -2.78036 0.09009 0.26842
C -4.18119 0.16344 0.12676
C -4.85594 -0.50629 -0.90398
C -4.08035 -1.27611 -1.81114
C -2.69512 -1.31322 -1.61469
C -1.96837 0.71908 1.31591
C -0.57823 0.32964 1.32636
C 0.18380 0.91824 2.35498
C -0.27626 1.83336 3.31801
C -1.64262 2.21656 3.27056
C -2.44153 1.64145 2.27292
Ru 0.01150 -0.81493 -0.30314
N 0.39550 -1.79838 -2.06305
C 0.65220 -2.33903 -3.07777
C 0.95292 -3.00338 -4.34056
C 0.65693 0.93223 -1.09810
C 2.07178 1.17188 -0.99226
C 2.61621 2.36424 -1.52107
C 1.86306 3.35281 -2.16361
C 0.46566 3.12794 -2.28987
C -0.06651 1.93994 -1.75929
C 2.83615 0.10993 -0.32938
N 2.04507 -0.93514 0.09824
C 2.63865 -1.99592 0.69955
C 4.01277 -2.10150 0.94509
C 4.84022 -1.02610 0.52477
C 4.22957 0.06280 -0.11404
O -0.41169 -2.92322 0.46948
C -0.80913 -3.18182 1.62821
O -0.97911 -2.23592 2.54865
C -1.12610 -4.57782 2.09375
F 3.97203 2.59175 -1.41012
F -3.76302 2.02986 2.24092
H -4.75044 0.75416 0.84466
C -6.35657 -0.41307 -1.03754
C -4.70549 -2.03808 -2.95498
H -2.06079 -1.89252 -2.29110
C -2.21789 3.20205 4.26142
C 0.64754 2.39638 4.37340
F 1.51381 0.55660 2.45440
H 0.64558 -2.37128 -5.19042
H 2.03442 -3.20126 -4.42335
H 0.41519 -3.96390 -4.40511
H 1.96089 -2.80360 0.99105
C 4.57141 -3.32527 1.63072
C 6.33286 -1.05355 0.74971
H 4.84301 0.89610 -0.45670
F -1.42909 1.76443 -1.92031
C -0.41716 4.14385 -2.97870
C 2.51217 4.60675 -2.70387
H -0.75416 -1.34937 2.08771
H -2.19029 -4.64241 2.37516
H -0.53507 -4.81821 2.99247
H -0.90964 -5.30120 1.29751
H -3.94282 -2.57399 -3.54211
H -5.43984 -2.78048 -2.59352
H -5.24966 -1.36348 -3.64051
H -6.64779 0.01032 -2.01605
H -6.82962 -1.41000 -0.97494
H -6.78948 0.22116 -0.24843
H -2.11012 2.83914 5.29960
H -1.69694 4.17515 4.20974
H -3.28569 3.37646 4.07036
H 1.66269 1.99162 4.26448
H 0.70523 3.49801 4.30887
H 0.28679 2.15776 5.39033
H 5.31508 -3.84137 0.99689
H 5.08705 -3.06174 2.57191
H 3.77413 -4.04604 1.87262
H 6.81274 -0.14251 0.35974
H 6.57615 -1.13637 1.82461
H 6.79884 -1.92549 0.25561
H 2.08674 5.51493 -2.23953
H 3.59445 4.60307 -2.51342
H 2.35746 4.70571 -3.79370
H -0.09789 4.30999 -4.02370
H -1.46484 3.81425 -2.98751
H -0.36756 5.12577 -2.47399

71
Int(6a^F-7a^F)
SCF(BP86)= -1896.827752
G(298 K) = -1896.356707
SCF(PBE) = -1895.39358605
Low Freq. = 15.0759cm-1,
16.2652cm-1

```
C  -4.18744  0.07917 -0.20804
C  -2.80694 -0.17212 -0.06843
N  -1.93968  0.21977 -1.06560
C  -2.43693  0.84828 -2.15847
C  -3.79365  1.13265 -2.35501
C  -4.70348  0.72928 -1.33908
C  -2.11180 -0.81455  1.04687
C  -0.68449 -0.93512  0.88341
C   0.00152 -1.50615  1.97378
C  -0.59386 -1.96933  3.15815
C  -2.00573 -1.85870  3.29778
C  -2.71170 -1.28395  2.23619
Ru  0.04133 -0.30320 -0.84953
F  -4.07819 -1.17385  2.37805
C  -2.71514 -2.34002  4.54279
C   0.24117 -2.57484  4.26383
F   1.37242 -1.63759  1.88399
C  -4.25093  1.84082 -3.60751
C  -6.18460  0.99199 -1.47015
N   2.07681 -0.71773 -0.72007
C   2.90895  0.33994 -0.40163
C   4.30676  0.16302 -0.43375
C   4.88877 -1.06979 -0.76712
C   4.02304 -2.15242 -1.07409
C   2.64467 -1.91158 -1.03430
C   2.17727  1.56038 -0.03150
C   0.74050  1.45301 -0.07471
C   0.05321  2.59977  0.36155
C   0.63402  3.80768  0.78795
C   2.05100  3.90074  0.79133
C   2.77313  2.77167  0.38321
C   6.38877 -1.23488 -0.79262
C   4.54624 -3.52186 -1.43521
F  -1.32863  2.56095  0.38203
C  -0.21592  4.97685  1.23042
C   2.75548  5.16520  1.22671
F   4.14775  2.88069  0.40075
N  -0.40802 -2.03960 -1.86080
C  -0.68325 -2.98982 -2.50356
C  -1.00615 -4.16780 -3.29887
H   4.94910  1.00891 -0.18965
H   1.93948 -2.71482 -1.26535
H  -0.83001 -5.08646 -2.71450
H  -2.06418 -4.14603 -3.60836
H  -0.37624 -4.20221 -4.20353
H  -1.69238  1.13747 -2.90993
H  -4.86439 -0.24152  0.58453
H   3.72162 -4.22475 -1.63409
H   5.18675 -3.48617 -2.33501
H   5.16497 -3.94535 -0.62353
H   6.71938 -2.01479 -0.08273
H   6.74163 -1.55147 -1.79102
H   6.89936 -0.29518 -0.53094
H   2.46055  6.02732  0.60140
H   2.50267  5.42923  2.26967
H   3.84637  5.05263  1.15923
H  -1.28481  4.73250  1.16671
H   0.01127  5.26643  2.27248
H  -0.02685  5.86946  0.60675
H  -4.76342  2.79086 -3.37126
H  -3.40056  2.07071 -4.26909
H  -4.97036  1.22768 -4.17996
H  -6.73653  0.61075 -0.59702
H  -6.39513  2.07296 -1.56334
H  -6.60068  0.51305 -2.37513
H  -2.35038 -1.81719  5.44560
H  -3.79846 -2.17098  4.46748
H  -2.54920 -3.41948  4.71263
H  -0.07007 -3.61342  4.47788
H   1.30611 -2.58327  3.99506
H   0.12692 -2.01005  5.20677
```

77
7a^F
SCF(BP86)= -2029.61144726
G(298 K) = -2029.098204
SCF(PBE) = -2028.06870948
Low Freq. = 16.4170cm-1,
17.4127cm-1

```
C   2.59152 -2.12310 -1.87400
C   2.05032 -1.26941 -0.88518
C   0.63756 -1.29947 -0.61754
C  -0.08583 -2.22302 -1.38983
C   0.43844 -3.07795 -2.37540
C   1.83474 -3.02493 -2.63038
C   2.82132 -0.31865 -0.07464
N   2.04161  0.41957  0.79149
C   2.65194  1.32282  1.59911
C   4.02908  1.57245  1.61754
C   4.84484  0.82358  0.72891
C   4.21750 -0.11455 -0.10288
Ru -0.00005 -0.00030  0.84349
N  -0.43570  1.40370  2.31404
C  -0.71504  2.19771  3.13784
C  -1.04578  3.18795  4.15639
C  -0.63733  1.29995 -0.61672
C  -2.05009  1.27028 -0.88448
C  -2.59110  2.12469 -1.87277
C  -1.83415  3.02691 -2.62853
C  -0.43786  3.07956 -2.37342
C   0.08623  2.22391 -1.38838
C  -2.82127  0.31896 -0.07477
C  -4.21750  0.11520 -0.10322
C  -4.84505 -0.82358  0.72767
C  -4.02947 -1.57351  1.61557
C  -2.65228 -1.32413  1.59742
N  -2.04174 -0.42015  0.79076
N   0.43515 -1.40560  2.31300
C   0.71428 -2.20058  3.13593
C   1.04468 -3.19230  4.15315
H  -1.02575  4.20287  3.72573
H  -2.05284  2.99974  4.56392
H  -0.31885  3.14348  4.98432
H  -4.82028  0.70819 -0.79073
```

C -6.34047 -1.02518 0.67771
C -4.60301 -2.60763 2.55457
H -1.98496 -1.88068 2.26120
F -3.94608 2.08865 -2.13235
C -2.47860 3.91006 -3.67280
C 0.44989 4.03090 -3.14329
F 1.45060 2.32373 -1.17418
H 1.21239 -4.17760 3.68741
H 1.95986 -2.89967 4.69400
H 0.22140 -3.28377 4.88100
H 1.98451 1.87858 2.26345
C 4.60236 2.60572 2.55764
C 6.34020 1.02564 0.67908
H 4.82042 -0.70678 -0.79093
F -1.45019 -2.32313 -1.17575
C -0.44912 -4.02890 -3.14596
C 2.47935 -3.90728 -3.67522
F 3.94652 -2.08674 -2.13341
H -6.59745 -2.06717 0.41353
H -6.80682 -0.82776 1.66011
H -6.80915 -0.35929 -0.06348
H -3.81241 -3.07976 3.15942
H -5.33983 -2.16213 3.24737
H -5.13101 -3.40680 2.00343
H -2.32149 4.98111 -3.44993
H -2.05061 3.72913 -4.67554
H -3.56116 3.72965 -3.72781
H 0.40335 3.83334 -4.22972
H 0.13393 5.08038 -3.00024
H 1.49617 3.93988 -2.82177
H 6.80919 0.35965 -0.06183
H 6.59684 2.06762 0.41450
H 6.80650 0.82878 1.66160
H 5.13075 3.40524 2.00740
H 3.81155 3.07748 3.16250
H 5.33878 2.15955 3.25043
H 3.56180 -3.72623 -3.73050
H 2.32291 -4.97852 -3.45279
H 2.05095 -3.72615 -4.67775
H -0.40303 -3.83018 -4.23221
H -0.13262 -5.07838 -3.00412
H -1.49534 -3.93869 -2.82402

84
$2a^F$ 2-ppy^F-H
SCF(BP86)= -2162.83812837
G(298 K) = -2162.270336
SCF(PBE) = -2161.19062894
Low Freq. = 12.8266cm-1,
14.7901cm-1
C -3.33909 -1.37768 0.72854
C -3.19604 -0.20467 -0.03267
C -3.68479 -0.22892 -1.35136
C -4.28557 -1.35151 -1.94490
C -4.40438 -2.53764 -1.17195
C -3.91664 -2.50556 0.14527
C -2.69678 1.05796 0.58606
N -1.41757 1.15629 1.05826
C -1.11067 2.30372 1.72778
C -1.97437 3.38651 1.93754
C -3.28185 3.30243 1.39494
C -3.61485 2.11728 0.72521
Ru 0.46102 -0.16172 0.58435
N 1.36388 0.55481 2.21591
C 1.96226 0.90296 3.16443
C 2.70100 1.33616 4.34209
C 2.26650 -1.01556 0.12567
C 3.18728 -0.13924 -0.53648
C 4.49927 -0.59111 -0.80658
C 4.96312 -1.87340 -0.49028
C 4.05010 -2.76493 0.12937
C 2.74905 -2.30131 0.40430
C 2.63964 1.16000 -0.94727
N 1.31906 1.33946 -0.59620
C 0.66800 2.44425 -1.03565
C 1.26078 3.46065 -1.79490
C 2.63626 3.32030 -2.11649
C 3.30005 2.16155 -1.68903
N -0.27776 -0.97203 -1.08035
C -0.56708 -1.48452 -2.09524
C -0.90730 -2.12810 -3.35635
N -0.26040 -1.64702 1.71237
C -0.67856 -2.51012 2.38824
C -1.22473 -3.60554 3.17806
F 5.39258 0.25857 -1.41615
H 4.34899 2.01900 -1.94618
C 3.36326 4.37699 -2.91004
C 0.44722 4.65148 -2.23829
H -0.38609 2.51173 -0.75903
C 6.38053 -2.29213 -0.80677
C 4.46914 -4.17224 0.48597
F 1.89928 -3.20971 0.99820
H -2.18540 -3.92753 2.74241
H -0.52970 -4.46139 3.18197
H -1.39610 -3.28329 4.21810
H 2.00978 1.50817 5.18325
H 3.43252 0.56556 4.63588
H 3.24024 2.27344 4.12831
H -0.08809 2.36302 2.10681
C -1.50325 4.59644 2.70579
C -4.27506 4.42526 1.54658
H -4.62140 1.98898 0.31575
F -3.54806 0.90778 -2.10069
C -4.80754 -1.28899 -3.36147
C -5.04247 -3.78379 -1.73637
F -4.02493 -3.63308 0.91547
H -3.02321 -1.39979 1.77226
H 4.41816 4.10587 -3.06766
H 2.89308 4.52693 -3.89850
H 3.32999 5.35407 -2.39547
H 0.43453 4.74251 -3.33917
H -0.59517 4.57446 -1.89179
H 0.86798 5.59497 -1.84681
H 3.64683 -4.72031 0.96474
H 4.78371 -4.73421 -0.41191
H 5.33239 -4.16894 1.17551
H 6.92631 -2.58521 0.10797
H 6.39985 -3.16561 -1.48283
H 6.93562 -1.47553 -1.28836
H -5.23330 4.18317 1.06271
H -4.46894 4.64188 2.61236
H -3.88764 5.35924 1.10185

H -1.56578 5.51165 2.09070
H -2.12902 4.77068 3.59915
H -0.46002 4.47887 3.03782
H -5.04625 -4.59359 -0.99403
H -6.08499 -3.59168 -2.04565
H -4.50278 -4.13773 -2.63251
H -4.36788 -2.08388 -3.98843
H -5.90234 -1.43491 -3.38383
H -4.58267 -0.31804 -3.82336
H 0.01017 -2.45672 -3.87203
H -1.54461 -3.00832 -3.17367
H -1.44865 -1.42661 -4.01158

78
$3a^F$ $2\text{-}ppy^F$-H
SCF(BP86)= -2030.06770373
G(298 K) = -2029.542200
SCF(PBE) = -2028.52307105
Low Freq. = 18.1469cm-1,
20.3363cm-1
C 3.63672 -2.22392 0.54593
C 2.65971 -1.22505 0.38813
N 1.33047 -1.50643 0.56848
C 0.99878 -2.74919 1.00640
C 1.91903 -3.78217 1.22092
C 3.28987 -3.51971 0.95526
C 3.00013 0.19188 0.13899
C 2.25862 1.20599 0.79382
C 2.59433 2.54563 0.57991
C 3.62388 2.96488 -0.27694
C 4.36413 1.95648 -0.95034
C 4.03296 0.60967 -0.72018
Ru -0.11356 0.02086 0.27094
N -0.68206 -0.26978 2.15752
C -1.03292 -0.43092 3.26708
C -1.45877 -0.62227 4.64597
C -1.72592 -0.97518 -0.40777
C -2.96639 -0.26436 -0.32262
C -4.15885 -0.91839 -0.70182
C -4.20310 -2.21994 -1.21662
C -2.96813 -2.89723 -1.39447
C -1.78410 -2.24243 -1.00583
C -2.84260 1.15190 0.03623
N -1.53403 1.54787 0.20058
C -1.26392 2.85281 0.44127
C -2.24411 3.84809 0.55199
C -3.60163 3.45259 0.41615
C -3.87678 2.10114 0.15833
N 0.44227 0.33786 -1.61201
C 0.73066 0.52795 -2.73411
C 1.09029 0.76547 -4.12488
H -4.90972 1.77309 0.04183
H 2.15270 0.52302 -4.28972
H 0.47627 0.13417 -4.78791
H 0.92268 1.82301 -4.38736
F -5.35208 -0.24957 -0.57956
C -5.51626 -2.86508 -1.59504
C -2.92820 -4.28528 -1.99030
F -0.60341 -2.91033 -1.25200
C -4.72059 4.45639 0.54029
C -1.84491 5.27896 0.81692
H -0.20684 3.10278 0.56372
H -0.59674 -0.90333 5.27285
H -1.89620 0.30916 5.04133
H -2.21513 -1.42215 4.70399
H -0.06721 -2.91606 1.17211
C 1.44386 -5.13057 1.70221
C 4.34060 -4.58528 1.13507
H 4.68431 -1.96770 0.37276
F 4.73744 -0.34668 -1.39281
C 5.48478 2.31884 -1.89407
C 3.93886 4.42869 -0.46076
F 1.88508 3.49273 1.26618
H 1.53727 0.95415 1.59074
H -5.70388 3.97940 0.41092
H -4.70360 4.95244 1.52729
H -4.62256 5.25693 -0.21485
H -2.19578 5.95006 0.01262
H -2.28724 5.65268 1.75778
H -0.75112 5.38044 0.89158
H -3.55090 -4.98605 -1.40635
H -3.32794 -4.28794 -3.02044
H -1.90212 -4.67540 -2.01976
H -5.68598 -3.79459 -1.02262
H -6.35874 -2.18688 -1.40202
H -5.53557 -3.14062 -2.66453
H 5.34363 -4.20595 0.88837
H 4.35668 -4.95683 2.17533
H 4.13257 -5.45932 0.49234
H 1.93402 -5.41279 2.65074
H 0.35503 -5.13679 1.86474
H 1.68483 -5.92313 0.97142
H 3.27127 5.05497 0.14616
H 4.98106 4.64938 -0.17088
H 3.82907 4.72760 -1.51800
H 5.12168 2.97011 -2.70802
H 6.27720 2.87760 -1.36563
H 5.93390 1.42215 -2.34099

81
TS($12a^F$-$13c^F$)
SCF(BP86)= -2123.62439295
G(298 K) = -2123.083766
SCF(PBE) = -4682.38825865
Low Freq. = -81.7865cm-1,
14.6233cm-1
N -2.04172 0.50332 -0.60346
C -2.89593 0.05185 0.38298
C -4.29039 0.17949 0.21548
C -4.84249 0.79667 -0.91673
C -3.95101 1.30780 -1.89742
C -2.57891 1.12936 -1.68221
C -2.18669 -0.46815 1.55471
C -0.74661 -0.37973 1.49107
C -0.08001 -0.67710 2.69882
C -0.68727 -1.14746 3.87539
C -2.09910 -1.31193 3.88911
C -2.79829 -0.95667 2.73028
Ru -0.01778 0.07639 -0.30541
N 1.99109 -0.27032 0.10121
C 2.79354 0.85287 0.13711
C 4.18986 0.71564 0.28379

```
C   4.78705 -0.54060  0.46254
C   3.93966 -1.68019  0.51322
C   2.56743 -1.47771  0.32449
C   2.02028  2.09576  0.11481
C   0.58892  1.92106  0.12395
C  -0.15720  3.09829  0.33832
C   0.37539  4.39491  0.43313
C   1.78491  4.55753  0.33419
C   2.55673  3.40036  0.18564
Br  0.58774  0.19833 -3.09441
C   0.27136 -1.55334 -2.24881
C  -1.05586 -1.99981 -2.02170
C  -1.27183 -3.33879 -1.66110
C  -0.19881 -4.25211 -1.60025
C   1.10777 -3.81767 -1.93300
C   1.33800 -2.48055 -2.27449
O  -0.52334 -5.52968 -1.24536
C   0.53278 -6.50533 -1.21351
H  -4.95114 -0.20289  0.99342
C  -6.33786  0.92339 -1.07673
C  -4.44084  2.01673 -3.13702
H  -1.85619  1.49826 -2.41559
C  -2.82051 -1.82946  5.11201
C   0.13447 -1.46989  5.10278
F   1.28654 -0.50257  2.75581
H   1.87875 -2.32637  0.34429
C   4.47753 -3.07072  0.75171
C   6.28240 -0.67050  0.62302
H   4.81077  1.61175  0.27825
F  -1.52712  2.99501  0.46009
C  -0.52286  5.59475  0.63206
C   2.42694  5.92401  0.40243
H  -1.90825 -1.33763 -2.17204
H   2.34635 -2.15375 -2.53855
H  -2.28595 -3.69729 -1.46209
H   1.94969 -4.51347 -1.93495
F   3.92235  3.56466  0.11144
F  -4.16836 -1.09518  2.76473
H   0.05698 -7.44607 -0.90490
H   0.99117 -6.63478 -2.21021
H   1.31245 -6.23002 -0.48083
H   6.77810  0.31076  0.56232
H   6.54441 -1.12819  1.59427
H   6.71527 -1.32309 -0.15686
H   5.21065 -3.36061 -0.02278
H   4.99967 -3.14214  1.72306
H   3.66774 -3.81756  0.74749
H  -1.57801  5.29485  0.68557
H  -0.26939  6.13419  1.56257
H  -0.41024  6.31959 -0.19440
H   2.05760  6.58469 -0.40274
H   2.19957  6.43046  1.35796
H   3.51928  5.85056  0.30833
H  -6.68595  0.42907 -2.00185
H  -6.87099  0.47224 -0.22564
H  -6.64566  1.98204 -1.15296
H  -3.59990  2.33934 -3.77131
H  -5.09436  1.36494 -3.74457
H  -5.03634  2.91184 -2.88171
H  -2.46587 -2.83746  5.39370
H  -2.65063 -1.17703  5.98765
H  -3.90355 -1.88654  4.93563
H   1.20383 -1.30080  4.91868
H  -0.17047 -0.84679  5.96303
H  -0.00274 -2.52227  5.41027
```

72
15a^{F} radical
SCF(BP86)= -1910.29245872
G(298 K) = -1909.822863
SCF(PBE) = -4469.21669387
Low Freq. = 17.4586cm-1, 21.2399cm-1

```
C   2.74623  2.47087  1.18720
C   2.14426  1.37866  0.52857
C   0.71601  1.36354  0.36071
C   0.02499  2.45535  0.91688
C   0.61589  3.54933  1.57019
C   2.03055  3.55734  1.70917
C   2.84502  0.21038 -0.00355
N   1.99555 -0.71595 -0.56953
C   2.52316 -1.85045 -1.09316
C   3.88852 -2.15596 -1.09607
C   4.77835 -1.20942 -0.51900
C   4.23329 -0.03558  0.01801
Ru -0.01098 -0.21293 -0.67951
N  -0.61793 -1.97046 -1.75908
C  -0.99850 -2.91960 -2.33286
C  -1.45788 -4.09754 -3.05414
C  -0.69477 -1.00009  1.05956
C  -2.11856 -0.93697  1.24554
C  -2.68122 -1.52766  2.39541
C  -1.93096 -2.17641  3.38793
C  -0.52126 -2.23179  3.22664
C   0.03431 -1.63276  2.08311
C  -2.85947 -0.21583  0.20624
C  -4.25189 -0.00220  0.15231
C  -4.84131  0.72069 -0.89438
C  -3.99277  1.23335 -1.91073
C  -2.61923  0.98015 -1.80319
N  -2.05077  0.28617 -0.78832
Br  0.65009  0.92560 -3.03521
H  -4.87953 -0.40784  0.94522
C  -6.33266  0.94495 -0.93439
C  -4.52731  2.02798 -3.07700
H  -1.91799  1.34550 -2.56046
C  -2.60397 -2.78698  4.59366
C   0.34948 -2.90632  4.26040
F   1.40159 -1.69221  1.96987
H   1.80154 -2.54623 -1.52875
C   4.37599 -3.45105 -1.69814
C   6.26606 -1.45725 -0.48496
H   4.89234  0.70860  0.46456
F  -1.34671  2.47708  0.81220
C  -0.21964  4.68335  2.11607
C   2.74169  4.69480  2.40252
F   4.11102  2.49504  1.34242
F  -4.04035 -1.47897  2.58249
H  -1.32437 -4.99537 -2.42918
H  -2.52507 -3.99433 -3.30796
H  -0.87774 -4.21952 -3.98312
H   6.80063 -0.62150 -0.00817
```

```
H   6.50449 -2.38174  0.07129
H   6.67227 -1.59062 -1.50377
H   0.06589  5.64599  1.65529
H  -1.28784  4.51375  1.92621
H  -0.07497  4.79705  3.20539
H   3.82805  4.53367  2.41615
H   2.54168  5.65675  1.89760
H   2.39610  4.80532  3.44596
H   5.08474 -3.26811 -2.52562
H   4.91121 -4.06556 -0.95217
H   3.53938 -4.04839 -2.09337
H  -3.71646  2.33467 -3.75609
H  -5.25927  1.44225 -3.66173
H  -5.05045  2.93979 -2.73689
H  -6.77470  0.52719 -1.85680
H  -6.83183  0.47842 -0.07141
H  -6.57409  2.02316 -0.93161
H   0.06678 -3.96652  4.38807
H   1.40860 -2.86337  3.97379
H   0.23857 -2.42611  5.24909
H  -2.23103 -2.33443  5.52973
H  -3.69255 -2.64734  4.55424
H  -2.39649 -3.86997  4.65916
```

77
7a^{F} radical
SCF(BP86)= -2029.45650772
G(298 K) = -2028.943441
SCF(PBE) = -2027.91132670
Low Freq. = 19.0087cm-1, 23.2950cm-1

```
C   2.77804 -2.06747 -1.75271
C   2.16673 -1.18850 -0.83774
C   0.73585 -1.23289 -0.67970
C   0.05340 -2.14919 -1.50252
C   0.65643 -3.03258 -2.41292
C   2.07084 -2.99073 -2.54029
C   2.85939 -0.17815 -0.03273
N   2.00693  0.57105  0.74655
C   2.52116  1.54967  1.52885
C   3.88387  1.86346  1.60009
C   4.77903  1.09738  0.80605
C   4.24422  0.08227  0.00024
Ru -0.00008 -0.00183  0.74253
N  -0.61595  1.35396  2.30011
C  -1.00063  2.07835  3.13714
C  -1.47216  2.98002  4.17692
C  -0.73373  1.23734 -0.67385
C  -2.16438  1.19423 -0.83431
C  -2.77406  2.07838 -1.74535
C  -2.06543  3.00574 -2.52682
C  -0.65121  3.04647 -2.39711
C  -0.04980  2.15794 -1.49064
C  -2.85851  0.17959 -0.03600
C  -4.24354 -0.08021 -0.00618
C  -4.77991 -1.09958  0.79318
C  -3.88612 -1.87077  1.58378
C  -2.52313 -1.55729  1.51605
N  -2.00740 -0.57447  0.74003
N   0.61233 -1.36782  2.29227
C   0.99164 -2.10043  3.12460
C   1.45373 -3.01554  4.15682
H  -1.76285  2.40083  5.06817
H  -0.67488  3.68837  4.45375
H  -2.34700  3.54428  3.81608
H  -4.90775  0.52614 -0.62130
C  -6.26420 -1.36670  0.80966
C  -4.36356 -2.99226  2.47248
H  -1.79670 -2.12111  2.10706
F  -4.13579  2.05221 -1.90197
C  -2.78747  3.92241 -3.48349
C   0.17351  4.01048 -3.21601
F   1.31825  2.20981 -1.40292
H   2.48219 -2.75837  4.45688
H   0.79446 -2.95401  5.03746
H   1.44077 -4.04779  3.77112
H   1.79359  2.10966  2.12215
C   4.35970  2.97991  2.49599
C   6.26311  1.36545  0.82562
H   4.90952 -0.52013 -0.61758
F  -1.31478 -2.20191 -1.41711
C  -0.16676 -3.99240 -3.23825
C   2.79458 -3.90200 -3.50084
F   4.14000 -2.04009 -1.90709
H  -3.52404 -3.46638  3.00447
H  -5.08404 -2.62815  3.22654
H  -4.88254 -3.77351  1.88891
H  -6.48620 -2.40176  0.49360
H  -6.67847 -1.25365  1.82761
H  -6.80268 -0.67909  0.14006
H  -0.12837  5.05519 -3.02248
H   1.24234  3.91099 -2.98522
H   0.03434  3.83363 -4.29743
H  -2.44018  3.76700 -4.52046
H  -3.87215  3.75404 -3.45452
H  -2.59426  4.98212 -3.23905
H   5.07892  2.61151  3.24917
H   4.87961  3.76452  1.91779
H   3.51924  3.45087  3.02926
H   6.80281  0.68187  0.15290
H   6.48467  2.40237  0.51539
H   6.67637  1.24723  1.84339
H   0.13510 -5.03805 -3.04986
H  -1.23598 -3.89443 -3.00858
H  -0.02598 -3.80973 -4.31849
H   3.87919 -3.73372 -3.46910
H   2.60102 -4.96306 -3.26268
H   2.44904 -3.74080 -4.53752
```

62
TS(14b^{F}-16c^{F})
SCF(BP86)= -1650.40643118
G(298 K) = -1650.009206
SCF(PBE) = -4209.53709489
Low Freq. = -156.3386cm-1, 18.9302cm-1

```
C   4.67497 -0.53063 -0.96832
C   4.04005 -1.77639 -0.75364
C   2.65881 -1.90920 -0.90976
C   1.88289 -0.76905 -1.22721
C   2.51473  0.45229 -1.53301
C   3.90369  0.58196 -1.36829
```

Br 0.07827 -1.11028 -2.16253
Ru 0.02225 -0.60177 0.47265
O -0.16535 -0.26481 2.51286
C 1.08398 -0.23535 2.89446
C 1.35549 -0.04294 4.37331
C -1.93283 -0.39582 0.09065
C -2.37624 0.95614 -0.12037
C -3.73713 1.19945 -0.40595
C -4.70760 0.19491 -0.50334
C -4.28404 -1.14594 -0.30870
C -2.92540 -1.38563 -0.03096
C -1.33927 1.98282 0.01440
N -0.09507 1.45292 0.28125
C 0.93757 2.29657 0.53566
C 0.83894 3.69270 0.49460
C -0.42439 4.25465 0.16773
C -1.49844 3.38192 -0.06044
N 0.07906 -2.55336 0.85343
C 0.15444 -3.68251 1.17406
C 0.22317 -5.08734 1.55507
O 2.00959 -0.38399 2.04811
F -4.15749 2.49322 -0.60718
H -2.48375 3.78875 -0.28679
C -0.61451 5.74916 0.08451
C 2.04723 4.54827 0.78810
H 1.88211 1.80535 0.78420
C -6.14919 0.52871 -0.80985
C -5.27190 -2.28588 -0.40399
F -2.56655 -2.70386 0.13345
H -0.72152 -5.59975 1.30737
H 1.04720 -5.59146 1.02327
H 0.39926 -5.18059 2.63954
H 0.45153 0.27941 4.91005
H 1.70529 -0.99552 4.80685
H 2.15760 0.69863 4.51321
H 2.18258 -2.88037 -0.76286
H 1.93026 1.30683 -1.88162
H 4.65130 -2.64059 -0.47535
H 4.36950 1.54825 -1.57596
O 6.03323 -0.51715 -0.78847
H -1.65256 6.00762 -0.17486
H -0.36660 6.23789 1.04400
H 0.05233 6.19486 -0.67551
H 1.86590 5.22232 1.64448
H 2.92675 3.92886 1.02386
H 2.30456 5.19171 -0.07251
H -4.78069 -3.25124 -0.22342
H -6.08751 -2.16868 0.33204
H -5.74715 -2.32010 -1.40089
H -6.82197 0.18119 -0.00542
H -6.28717 1.61246 -0.92571
H -6.48625 0.03864 -1.74095
C 6.72017 0.72079 -1.02659
H 7.78049 0.51805 -0.82153
H 6.60602 1.05150 -2.07510
H 6.36164 1.51832 -0.35033

52
$3a^{F}_{OAc}$ radical
SCF(BP86)= -1423.46408129
G(298 K) = -1423.134477
SCF(PBE) = -1422.39619576
Low Freq. = 18.0911cm-1,
25.5137cm-1
C 0.80541 0.91045 -0.06199
C -0.39524 1.71229 -0.13383
C -0.26924 3.10291 -0.26004
C 0.97079 3.77235 -0.32405
C 2.16198 2.99964 -0.25582
C 2.03619 1.60664 -0.13854
C -1.65295 0.96610 -0.06639
C -2.96332 1.47858 -0.08878
C -4.07707 0.62867 -0.00310
C -3.85245 -0.77059 0.10636
C -2.52472 -1.21978 0.11718
N -1.45723 -0.39161 0.03432
Ru 0.49676 -1.04167 0.05251
N 2.39019 -1.74703 0.01969
C 3.44326 -2.25872 -0.00586
C 4.76195 -2.87207 -0.03987
O -0.11168 -3.08002 -0.74557
C 0.01466 -2.60067 -1.92685
O 0.36079 -1.35280 -2.03130
N 0.45166 -1.12428 2.04387
C 0.40603 -1.23038 3.21064
C 0.35693 -1.35983 4.65590
C -0.19173 -3.43383 -3.16114
H -3.10994 2.55512 -0.17246
H 0.84742 -2.29648 4.96668
H 0.88391 -0.50962 5.11962
H -0.68952 -1.36390 5.00083
H 5.38413 -2.47100 0.77659
H 4.67623 -3.96426 0.07878
H 5.25223 -2.65388 -1.00248
F -1.39225 3.87481 -0.32976
C 1.02437 5.27084 -0.46219
C 3.51803 3.65937 -0.30993
F 3.19021 0.88825 -0.07337
C -5.47545 1.19013 -0.02807
C -4.98627 -1.75882 0.20578
H -2.28815 -2.28556 0.18302
H -0.44773 -2.79863 -4.02111
H 0.74294 -3.97350 -3.39259
H -0.98101 -4.17998 -2.98785
H -6.02835 0.91759 0.88848
H -5.46698 2.28685 -0.11446
H -6.05118 0.77938 -0.87661
H -5.61531 -1.55610 1.09074
H -5.64833 -1.69667 -0.67603
H -4.61235 -2.79131 0.28121
H 4.32273 2.91623 -0.23792
H 3.64623 4.22048 -1.25225
H 3.63792 4.38377 0.51463
H 1.56297 5.56080 -1.38179
H 0.01777 5.70696 -0.49660
H 1.57339 5.72315 0.38269

47
$15a^{F}$
SCF(BP86)= -1304.30518364
G(298 K) = -1304.019543
SCF(PBE) = -3863.70593617

Low Freq. = 18.9323cm-1, 23.6439cm-1

C	0.75841	1.01228	-0.09185
C	-0.46208	1.77757	-0.15930
C	-0.38116	3.17631	-0.27547
C	0.83130	3.88568	-0.32626
C	2.04317	3.14782	-0.25364
C	1.96073	1.74940	-0.14307
C	-1.69560	0.99254	-0.08906
C	-3.02264	1.46371	-0.09650
C	-4.10911	0.58062	-0.00356
C	-3.83979	-0.81132	0.09853
C	-2.49907	-1.21789	0.09469
N	-1.45497	-0.35924	0.00353
Ru	0.50845	-0.95894	-0.01808
Br	0.44835	-1.26729	2.45784
O	-0.04876	-3.04051	-0.84017
C	0.02517	-2.53425	-2.01146
O	0.31497	-1.27182	-2.11358
N	2.40829	-1.60361	-0.08364
C	3.48013	-2.07708	-0.13216
C	4.82094	-2.64134	-0.19148
C	-0.17955	-3.35518	-3.25835
H	-3.20261	2.53595	-0.17270
H	5.49103	-2.09754	0.49430
H	4.80135	-3.70431	0.09917
H	5.22021	-2.56051	-1.21572
F	-1.53492	3.91032	-0.34594
C	0.84016	5.38902	-0.45083
C	3.37939	3.85026	-0.29231
F	3.14494	1.07046	-0.06556
C	-5.52505	1.09950	-0.01074
C	-4.94175	-1.83537	0.20796
H	-2.22907	-2.27590	0.15829
H	-0.48549	-2.71775	-4.10044
H	0.76979	-3.85131	-3.52515
H	-0.93235	-4.13704	-3.07919
H	-5.56546	-1.65829	1.10231
H	-5.61779	-1.79221	-0.66454
H	-4.53376	-2.85584	0.27376
H	-6.09942	0.67577	-0.85397
H	-6.05984	0.80915	0.91125
H	-5.55012	2.19653	-0.09334
H	4.20634	3.13039	-0.23429
H	3.49274	4.43580	-1.22180
H	3.47829	4.56074	0.54754
H	1.36852	5.85347	0.40057
H	1.36979	5.70750	-1.36629
H	-0.18071	5.79185	-0.48509

16. References

[1] M. Simonetti, D. M. Cannas, X. Just-Baringo, I. J. Vitorica-Yrezabal, I. Larrosa, *Nat. Chem*. **2018**, *10*,724–731.

[2] P. Coppo, E. A. Plummer, L. De Cola, *Chem. Commun.* **2004**, *15*, 1774-1775.

[3] G. McArthur, J. H. Docherty, M. D. Hareram, M. Simonetti, I. J. Vitorica-Yrezabal, J. J. Douglas, I. Larrosa, *Nat. Chem.* **2024**, 16, 1141–1150.

[4] J. Burés, *Angew. Chem., Int. Ed.* **2016**, *55*, 2028– 2031.

[5] J. Burés, *Angew. Chem. Int. Ed.* **2016**, *55*, 16084– 16087.

[6] C. D.-T. Nielsen, J. Burés, *Chem. Sci.* **2019**, *10*, 348– 353.

[7] M. J. Frisch, G. W. Trucks, H. B. Schlegel, G. E. Scuseria, M. A. Robb, J. R. Cheeseman, G. Scalmani, V. Barone, B. Mennucci, G. A. Petersson, H. Nakatsuji, M. Caricato, X. Li, H. P. Hratchian, A. F. Izmaylov, J. Bloino, G. Zheng, J. L. Sonnenberg, M. Hada, M. Ehara, K. Toyota, R. Fukuda, J. Hasegawa, M. Ishida, T. Nakajima, Y. Honda, O. Kitao, H. Nakai, T. Vreven, J. A. Montgomery, J. E. Peralta, F. Ogliaro, M. Bearpark, J. J. Heyd, E. Brothers, K. N. Kudin, V. N. Staroverov, T. Keith, R. Kobayashi, J. Normand, K. Raghavachari, A. Rendell, J. C. Burant, S. S. Iyengar, J. Tomasi, M. Cossi, N. Rega, J. M. Millam, M. Klene, J. E. Knox, J. B. Cross, V. Bakken, C. Adamo, J. Jaramillo, R. Gomperts, R. E. Stratmann, O. Yazyev, A. J. Austin, R. Cammi, C. Pomelli, J. W. Ochterski, R. L. Martin, K. Morokuma, V. G. Zakrzewski, G. A. Voth, P. Salvador, J. J. Dannenberg, S. Dapprich, A. D. Daniels, O. Farkas, J. B. Foresman, J. V. Ortiz, J. Cioslowski, D. J. Fox, *Gaussian 16, Revision A.03*; Gaussian, Inc: Wallingford CT, 2016.

[8] A. D. Becke, *Phys. Rev. A* **1988**, *38*, 3098-3100.

[9] J. P. Perdew, *Phys. Rev. B* **1986**, *33*, 8822-8824.

[10] J. Tomasi, B. Mennucci, R. Cammi, *Chem. Rev.* **2005**, *105*, 2999-3094.

[11] D. Andrae, U. Häußermann, M. Dolg, H. Stoll, H. Preuß, *Theor. Chim. Acta* **1990**, *77*, 123-141.

[12] P. C. Hariharan, J. A. Pople, *Theor. Chim. Acta* **1973**, *28*, 213-222.

[13] W. J. Hehre, R. Ditchfield, J. A. Pople, *J. Chem. Phys.* **1972**, *56*, 2257-2261.

[14] R. F. Ribeiro, A. V. Marenich, C. J. Cramer, D. G. Truhlar, *J. Phys. Chem. B* **2011**, *115*, 14556-14562.

[15] G. Luchini, J. V. Alegre-Requena, I. Funes-Ardoiz, R. S. Paton, *F1000Research*, **2020**, *9*, 291.

[16] J. P. Perdew, K. Burke, M. Ernzerhof, *Phys. Rev. Lett.* **1996**, *77*, 3865-3868.

[17] F. Weigend, R. Ahlrichs, *Phys. Chem. Chem. Phys.* **2005**, *7*, 3297-3305.

[18] F. Weigend, *Phys. Chem. Chem. Phys.* **2006**, *8*, 1057-1065.

[19] S. Grimme, J. Antony, S. Ehrlich, H. Krieg, *J. Chem. Phys.* **2010**, *132*, 154104.

[20] S. Grimme, S. Ehrlich, L. Goerigk, *J. Comput. Chem.* **2011**, *32*, 1456-1465.

[21] C. Lee, W. Yang, R. G. Parr, *Phys. Rev. B* **1988**, *37*, 785-789.

[22] A. D. Becke, *J. Chem. Phys.* **1993**, *98*, 5648-5652.

[23] C. Adamo, V. Barone, *J. Chem. Phys.* **1999**, *110*, 6158-6170.

[24] S. Grimme, *J. Comput. Chem.* **2006**, *27*, 1787-1799.

[25] J. -D. Chai, M. Head-Gordon, *Phys. Chem. Chem. Phys.* **2008**, *10*, 6615-6620.

[26] Y. Zhao, D. G. Truhlar, *Theor. Chem. Acc.* **2008**, *120*, 215-241.

[27] J. Tao, J. P. Perdew, V. N. Staroverov, G. E. Scuseria, *Phys. Rev. Lett.* **2003**, *91*, 146401.
